# Complex Interplay of Genes Underlies Invasiveness in Fibrosarcoma Progression Model

**DOI:** 10.3390/jcm10112297

**Published:** 2021-05-25

**Authors:** Michaela Kripnerová, Hamendra Singh Parmar, Jiří Šána, Alena Kopková, Lenka Radová, Sieghart Sopper, Krzysztof Biernacki, Jan Jedlička, Michaela Kohoutová, Jitka Kuncová, Jan Peychl, Emil Rudolf, Miroslav Červinka, Zbyněk Houdek, Pavel Dvořák, Kateřina Houfková, Martin Pešta, Zdeněk Tůma, Martina Dolejšová, Filip Tichánek, Václav Babuška, Martin Leba, Ondřej Slabý, Jiří Hatina

**Affiliations:** 1 Institute of Biology, Faculty of Medicine in Pilsen, Charles University, 323 00 Plzen, Czech Republic; Michaela.Kripnerova@lfp.cuni.cz (M.K.); hamendrasingh999@yahoo.co.in (H.S.P.); Zbynek.Houdek@lfp.cuni.cz (Z.H.); Pavel.Dvorak@lfp.cuni.cz (P.D.); Katerina.Houfkova@lfp.cuni.cz (K.H.); Martin.Pesta@lfp.cuni.cz (M.P.); 2 Central European Institute of Technology (CEITEC), Masaryk University, 625 00 Brno, Czech Republic; jiri.sana@ceitec.muni.cz (J.Š.); alena.kopkova@ceitec.muni.cz (A.K.); avodar@gmail.com (L.R.); 3 Department of Comprehensive Cancer Care, Masaryk Memorial Cancer Institute, 602 00 Brno, Czech Republic; 4 Department of Pathology, University Hospital Brno, 625 00 Brno, Czech Republic; 5 Internal Medicine V, Medical University of Innsbruck, 6020 Innsbruck, Austria; sieghart.sopper@i-med.ac.at; 6 Tyrolean Cancer Research Institute, 6020 Innsbruck, Austria; 7 Department of Medical and Molecular Biology, Faculty of Medical Sciences in Zabrze, Medical University of Silesia in Katowice, 41-808 Zabrze, Poland; kbiernacki@sum.edu.pl; 8 Institute of Physiology, Faculty of Medicine in Pilsen, Charles University, 323 00 Plzen, Czech Republic; Jan.Jedlicka@lfp.cuni.cz (J.J.); Michaela.Markova@lfp.cuni.cz (M.K.); Jitka.Kuncova@lfp.cuni.cz (J.K.); 9 Department of Medical Biology and Genetics, Faculty of Medicine in Hradec Kralove, Charles University, 500 03 Hradec Kralove, Czech Republic; peychl@mpi-cbg.de (J.P.); Rudolf@lfhk.cuni.cz (E.R.); cervinka@lfhk.cuni.cz (M.Č.); 10 Biomedical Center, Faculty of Medicine in Pilsen, Charles University, 323 00 Plzen, Czech Republic; zdenek.tuma@lfp.cuni.cz (Z.T.); martina.dolejsova@lfp.cuni.cz (M.D.); 11 Institute of Pathological Physiology, Faculty of Medicine in Pilsen, Charles University, 323 00 Plzen, Czech Republic; tichanef@lfp.cuni.cz; 12 Institute of Medical Chemistry and Biochemistry, Faculty of Medicine in Pilsen, Charles University, 301 66 Plzen, Czech Republic; vaclav.babuska@lfp.cuni.cz; 13 Department of Cybernetics, Faculty of Applied Sciences, University of West Bohemia in Pilsen, 301 00 Plzen, Czech Republic; lebam@students.zcu.cz; 14 Department of Biology, Faculty of Medicine, Masaryk University, 625 00 Brno, Czech Republic

**Keywords:** fibrosarcoma, progression series, invasiveness, transcriptome, Ccl8

## Abstract

Sarcomas are a heterogeneous group of mesenchymal tumours, with a great variability in their clinical behaviour. While our knowledge of sarcoma initiation has advanced rapidly in recent years, relatively little is known about mechanisms of sarcoma progression. JUN-murine fibrosarcoma progression series consists of four sarcoma cell lines, JUN-1, JUN-2, JUN-2fos-3, and JUN-3. JUN-1 and -2 were established from a single tumour initiated in a *H2K/v-jun* transgenic mouse, JUN-3 originates from a different tumour in the same animal, and JUN-2fos-3 results from a targeted in vitro transformation of the JUN-2 cell line. The JUN-1, -2, and -3 cell lines represent a linear progression from the least transformed JUN-2 to the most transformed JUN-3, with regard to all the transformation characteristics studied, while the JUN-2fos-3 cell line exhibits a unique transformation mode, with little deregulation of cell growth and proliferation, but pronounced motility and invasiveness. The invasive sarcoma sublines JUN-2fos-3 and JUN-3 show complex metabolic profiles, with activation of both mitochondrial oxidative phosphorylation and glycolysis and a significant increase in spared respiratory capacity. The specific transcriptomic profile of invasive sublines features very complex biological relationships across the identified genes and proteins, with accentuated autocrine control of motility and angiogenesis. Pharmacologic inhibition of one of the autocrine motility factors identified, Ccl8, significantly diminished both motility and invasiveness of the highly transformed fibrosarcoma cell. This progression series could be greatly valuable for deciphering crucial aspects of sarcoma progression and defining new prognostic markers and potential therapeutic targets.

## 1. Introduction

Sarcomas represent a very heterogeneous group of tumours of mesenchymal origin, accounting for ~1% of human malignancies, affecting about 200,000 people per year across the globe. At the genomic level, sarcomas can be classified into two broad categories. The first group, especially prevailing among paediatric cases, involves tumours of near diploid karyotypes, featuring well-defined focused mutational changes, like specific translocations, amplifications, or activating point mutations in crucial driver oncogenes or inactivating mutations in key tumour suppressor genes. The second group comprises adult-type sarcomas including adult fibrosarcoma, osteosarcoma, chondrosarcoma, most liposarcomas, angiosarcoma, leiomyosarcoma, and undifferentiated pleomorphic sarcoma, with complex and unbalanced karyotypes and extensive genomic instability [1,2,3,4,5]. The clinical behaviour of sarcomas can be highly variable, ranging from indolent to highly aggressive invasive and metastatic tumours. Whereas localised tumours can frequently be successfully treated by surgery alone or in combination with radiotherapy, the prognosis of advanced stage metastatic sarcomas is poor, with a median overall survival of only 8–12 months [2,6]. While the issue of sarcoma initiation has experienced substantial progress in recent years, both regarding the identification of a plethora of driver fusion oncogenes in translocation-dependent sarcomas [3,7] and concerning mesenchymal stem cells as a probable cell of origin for most sarcomas [8], relatively little progress has been achieved in deciphering the mechanisms of sarcoma progression. This issue is of utmost importance, nevertheless, the metastatic dissemination is the principal cause of sarcoma-related death according to all the available evidence [9]; interestingly, a similar prognostic impact on patient survival as the metastatic dissemination seems to be the already existent presence of histologic invasion, suggesting that invasiveness might be a rate-limiting step of the entire metastatic cascade [10]. Moreover, a survey of recent soft tissue sarcoma phase III clinical trials revealed that up to 50% of enrolled patients experience rapid progression, which might obscure any effect of an investigational agent if it is not dramatic enough. Such rapid progressors are seen across histopathological subtypes, suggesting that a yet to be identified general aspect of disease biology might underlie them [11]. The CINSARC gene expression signature has been identified with the implicit assumption that metastatic ability is intimately connected to genome destabilisation, and there is convincing evidence that it can significantly outperform available clinical parameters in forecasting sarcoma cases with propensity to metastasise [12]. However, all the CINSARC-genes function primarily within the context of cell division, mitotic regulation, and chromosome integrity, and while some of the genes identified might have additional functions in classical progression-coupled traits like motility and invasiveness [13], the CINSARC signature as such probably reflects the mere proclivity to progress that is greatly facilitated by genome destabilisation in order to accumulate further mutations, rather than revealing the very biological mechanisms of progression. Indeed, the CINSARC signature is able to predict progression in tumour types that are as biologically different as sarcomas, carcinomas, lymphomas, and leukaemias [14]. Important insights into the mechanistic basis of sarcoma progression could be achieved by analysing several experimental model systems. A remarkable rat fibrosarcoma progression series has been instrumental in unravelling an important mechanistic basis of motility, invasiveness, and metastatic competence in terms of cytoskeletal dynamics [15,16]. Another interesting animal sarcoma progression series derived from Syrian hamster embryonic fibroblasts upon Rous sarcoma virus infection and in vivo selection disclosed an essential role of the cellular retinoic acid-binding protein 1 for in vivo tumour growth, both in terms of primary tumour inoculation and metastatic growth [17]. Two important entirely in vitro-based human sarcoma progression series have been reported as well, consisting in step-wise in vitro transduction of telomerase, viral, and cellular oncogenes into adult human fibroblasts and primary mesenchymal stem cells, respectively [18,19]. They have been especially instrumental in unravelling metabolic basis of sarcoma progression, with a pronounced cell-of-origin-specific variability in metabolic reprogramming. While the BJ-finite fibroblasts strain-based in vitro-derived sarcoma cells underwent a glycolytic switch during the terminal transformation step, the primary human bone marrow-originated mesenchymal stem cells and in vitro-derived sarcoma cells relied entirely upon mitochondrial oxidative phosphorylation, with glycolysis serving only as a temporal resort in response to hypoxia. It remains to be explored which of these two different and largely antithetical scenarios apply for genuine sarcomas. In addition, a regulatory or signalling framework, in which sarcoma progression could take place, has to our knowledge not been adequately addressed until now. To this end, we extended our previously published murine fibrosarcoma progression model [20], which allowed us to sharpen the focus of the subsequent expression analysis. This allowed us to disclose the strikingly complex signalling and metabolic contexts that potentially operate the sarcoma invasiveness.

## 2. Materials and Methods

### 2.1. Cell Culture

The JUN-1, -2, and -3 cell lines were described previously [20]. The cell lines were derived from a fibrosarcoma formed in response to tail biopsy in *H2-K/v-jun* transgenic mouse [21]. Cells were cultured in a high glucose (4500 mg/L) Dulbecco’s modified Eagle’s medium (Gibco/Invitrogen, Waltham, MA, USA) supplemented with 10% foetal calf serum (Sigma, Prague, CZ) and the antibiotics penicillin (final concentration 100 U/mL) and streptomycin (final concentration 100 μg/mL) (Sigma, Prague, CZ) at 37 °C in a humidified atmosphere containing 5% CO_2_. For subculturing, the cells were washed with 0.02% EDTA in phosphate-buffered saline (PBS, Sigma, Prague, CZ) and then briefly incubated with 0.05% trypsin/0.02% EDTA in PBS (Sigma, Prague, CZ).

### 2.2. Stable Transfection and Derivation of JUN-2fos-3 Cell Subline

JUN-2 cells were transfected by the calcium phosphate precipitation method, using the CalPhos Maximizer (TM) Transfection Kit (Clontech, San Francisco, CA, USA) according to the manufacturer’s instructions. The transfection mixture consisted of the CMV-*c-fos* expression vector (a generous gift from Dr. Thomas Curran, St. Jude Children’s Research Hospital, Memphis, TN, USA) and the pSTneoB vector [22] (a generous gift from Dr. Petr Draber, Institute of Molecular Genetics, Academy of Sciences of The Czech Republic, Prague, Czech Republic) at a 10:1 ratio. Stably transfected colonies were selected by cultivation in the culture medium containing G418 (Sigma, Prague, CZ) at the final concentration of 500 μg/mL. Clones were established by picking up individual colonies with a yellow micropipette tip under an inverted microscope. Clones were screened for the *c-fos* expression by Northern analysis. Total cellular RNA was prepared using the acid guanidium phenol-chloroform extraction method [23]. Total RNA (20 µg) was resolved on 0.9% agarose-formaldehyde gel electrophoresis overnight and transferred to a Hybond-N1 (Amersham) nylon membrane using 20× SSC blotting. Membranes were sequentially hybridised with *c-fos* cDNA and *α-tubulin* cDNA probes that were labelled with α32P-dCTP using the Megaprime™ DNA labelling system (Amersham, UK). Hybridisation was done for 24 h at 42 °C in 5× SSC, 5× Denhardt solution (Calbiochem, San Diego, CA, USA), 1% SDS, 100 mg/mL denatured sonicated salmon sperm DNA (Sigma, Prague, CZ), and 50% formamide (Calbiochem). Blots were washed with increasing stringency, followed by autoradiography at −70 °C using intensifying screens [24].

A single clone expressing the highest level of the *c-fos* transcript (clone 3, Figure S1) was designated as JUN-2fos-3 and used for all other analyses.

### 2.3. Expression of Fos and Jun Genes in Fibrosarcoma Cell Lines

Total RNA was isolated from cell lines using the RNeasy mini kit (Qiagen, Hilden, Germany). Then, 250 ng of total RNA were reverse transcribed using SuperScriptIII reverse transcriptase (Invitrogen), with random hexamer primers in a reaction volume of 20 µL. The *c-fos* transcript was amplified by the pair of primers with the following sequences: C-FOS forward 5′-GAC TCC TTC TCC AGC ATG GGC TC-3′; C-FOS reverse, 5′-GCT CTG GTC TGC GAT GGG GCC ACG-3′; the primer sequences are conserved between the murine (i.e., endogenous) and human (i.e., transfected) *c-fos* gene and the PCR amplicon was of 173 bp in length. The *jun* transcript was amplified by the pair of primers with the following sequences: JUN forward 5′-CAT CCA CGG CCA ACA TGC-3′; JUN reverse, 5′-TCA AAA CGT TTG CAA CTG-3′; the primer sequences are conserved between the murine *c-jun* and the *v-jun* genes [25] and the PCR amplicon was of 113 bp in length. The analysis was performed in technical duplicates on the Stratagene M×3005P apparatus (Agilent Technologies, Santa Clara, CA, USA) according to the manufacturer’s protocol. The amplification included initial denaturation at 95 °C for 10 min, followed by 40 cycles of 95 °C (10 s), 95 °C (30 s), 55 °C (1 min), and 72 °C (1 min). The qualitative PCR was performed by iTaq Universal SYBR^®^ Green SuperMix (Bio-Rad, Hercules, CA, USA). The 2 ΔΔCq method was used for the quantification of qPCR data; the expression was normalised to GAPDH gene with the following sequences: GAPDH forward 5′-AGG TCG GTG TGA ACG GAT TTG-3′; GAPDH reverse, 5′-TGT AGA CCA TGT AGT TGA GGT CA-3′.

### 2.4. Indirect Immunofluorescence

Cells were sparsely seeded on a coverslip and grown to 70% confluence, fixed with 100% ice-cold methanol for 30 min, permeabilised in 0.1% saponin-TBS solution, and blocked in 2% normal goat serum in PBS for 60 min. Cells were then sequentially incubated with the primary polyclonal rabbit anti-v-Jun antibody (Antibodies–online ABIN1109458, cross reactivity for avian and mammalian c-Jun) or primary polyclonal rabbit anti-human c-Fos antibody (Sigma F7799, cross reactivity for mouse, rat, and pig c-Fos, Sigma, Prague, CZ) (1% in PBS for 90 min at room temperature) and Atto 488–labelled goat anti-rabbit IgG secondary antibody (Sigma 18772—0.5% in PBS, 60 min at room temperature in dark, Sigma, Prague, CZ), with extensive washing after each incubation. The negative control staining wascarried out by omitting the respective primary antibody (Figure S2). The coverslips were mounted in Vectashield mounting medium (Vector Laboratories, Burlingame, CA, USA) and analysed using the Olympus AX70 fluorescent microscope equipped with the Olympus DP71 camera system.

### 2.5. Analysis of Cell Morphology

Basic morphological evaluation was carried out by routine phase contrast microscopic observation. The cell size analysis was described by a published procedure [26]. Briefly, phase contrast photographs of growing cells were taken at a 10× magnification by the Hamamatsu Orca-ER camera mounted on the Olympus IX 70 inverted microscope (Olympus, Tokyo, Japan). The QuickPHOTO Industrial 2.3 (Promicra Ltd., Prague, Czech Republic) software was used for the photo evaluations. The area of the cells in each clone (*n* = 15) was calculated based on the polygon surface created by tracing the contour of cells.

### 2.6. Evaluation of Growth Characteristics

The characteristics related to deregulated proliferation were analysed with the aid of the xCELLigence system (Roche, Basel, Switzerland) [27]. The xCELLigence system monitors the cellular events in real time by measuring electrical impedance using microelectrodes at the bottom of each cell culture plate well. The RTCA software calculates the cell index (CI) as a relative change in measured impedance. Two major parameters were assessed—the slope of the linear phase of the growth curve describing the steepness incline, and the doubling time (DT), i.e., the period of time required for a given quantity to double in size or value; assuming an exponential growth, the relationship between these parameters is: slope = log2/DT. The procedure was performed according to the instructions provided by the manufacturer. Briefly, 5000 cells in 100 μL DMEM per well were seeded in triplicate (E plate 16), in the final volume of 200 μL of growth medium and maintained in a culture under standard conditions. Dynamic cell proliferation and growth were monitored every 15 min for 77 h. Each cell line produced a distinct profile with the RTCA HT Instrument, corresponding to differences in growth rate, cell morphology or doubling time, and the values of the cell index were calculated and plotted on the graph. Experiments were performed in triplicates and repeated at least twice with similar results.

### 2.7. Anchorage-Independent Growth

The ability of anchorage-independent growth was quantified as clonogenicity in 15% methylcellulose-containing full growth medium [28]. Cells were harvested by routine trypsinisation, dissolved in 15% methylcellulose-containing full growth medium and partitioned in triplicates onto ultra-low attachment surface 6-well plates (Corning); 20,000 cells per well were used. Colonies were counted after four weeks of culture, with supplementation with the normal growth medium every week. Photographic documentation was taken by the Olympus IX 70 inverted microscope equipped with the Hamamatsu Orca-ER camera. Experiments were performed in triplicates and repeated four times with similar results.

### 2.8. Sphere Formation Assay and Side Population Assay

The ability to grow in sarcospheres was assessed as described [29,30]. Briefly, cells were harvested by routine trypsinisation at ~80% confluence. Then, 100,000 cells were seeded per one well in ultra-low attachment surface 6-well plates (Corning, NY, USA) in serum-free DMEM/1% methylcellulose medium supplemented with the recombinant mouse FGF2 (Sigma SRP4038–10 ng/mL, Sigma, Prague, CZ) EGF from murine submaxillary gland (Sigma E4127–10 ng/mL, Sigma, Prague, CZ) and 1× N2 supplement (Gibco/Invitrogen), with regular addition of FGF-2 and EGF every other day. Following 10–14 days in culture, colonies that contained >10 cells were quantitated by inverted phase contrast microscopy. Experiments were performed in triplicates and repeated a minimum of twice with similar results. The side population was analysed as DyeCycle™ Violet (ThermoFischer Scientific, Carslbad, CA, USA) dim cells, as described previously [31].

### 2.9. Motility Assay

The motility assay was performed as the in vitro wound-healing assay as described previously [20]. Briefly, cells were plated onto plastic Petri dishes (60 mm diameter) and grown to confluence. The confluent monolayers were wounded using white plastic micropipette tips, washed with culture medium, and returned to the incubator. The course of the healing was followed for 48 h in Olympus IX70 phase-contrast microscope, and photo-documented at several time points using the Olympus C-35AD-4 camera.

### 2.10. Assessment of Invasive Ability

Two independent approaches were applied to assess the invasiveness of JUN- sarcoma cell lines. First, the BD BioCoatTM MatrigelTM Invasion Chamber (Becton Dickinson, Franklin Lakes, NJ, USA) was used, following the instructions of the manufacturer, with minor modifications, as described previously [20]. Briefly, after trypsinisation and harvesting, 50,000 cells were seeded onto 8 μm pore Matrigel-coated invasion chambers. The invasion test was left for 24 h, with complete, serum-supplemented culture medium in both the lower and upper compartments of the chamber. At the conclusion of the incubation time, cells attached to the upper surface of the membrane (i.e., non-invading) were mechanically removed. The invading cells were fixed using the Carnoy fixative and Giemsa (Sigma, Prague, CZ)-stained. Cell invasion was evaluated in triplicate and repeated twice and expressed as an absolute average number of invading cells from five randomly chosen fields using an Olympus IX 70 inverted microscope.

Second, we adopted a recently described method of three-dimensional invasion assay [32]. Multicell tumour spheroids were generated by a liquid overlay technique [33] by placing 200,000 cells into agarose-covered 6-well plates; spheroids were harvested after 10 days. The spheroids were embedded into type I collagen (Sigma, Prague, CZ); stock solution was made by diluting type I collagen in 0.02 M acetic acid to the final concentration of 8.5 mg/mL at 4 °C overnight. The experimental collagen type I solution was made by quickly mixing equal volumes of the stock solution and sterile neutralizing buffer (100 mM HEPES in 2× PBS). Next, 200 µL of the experimental collagen solution was quickly pipetted per well into a 24-well plate and allowed to solidify for 2 h in a standard CO_2_ incubator at 37 °C, 95% humidity, and 5% CO_2_. Afterwards, the single spheroids were carefully transferred onto the top of the gels and overlaid by 100 µL of experimental collagen solution and again incubated at 37 °C, 95% humidity, and 5% CO_2_ for an additional 2 h to allow for the solidification of the upper collagen layer. Finally, 50 µL of full growth medium was added to the top of the sandwich and the plate was returned into the CO_2_ incubator. After embedding, changes in the spheroids’ shape reflecting the active movement and invasion of tumour cells out of an embedded spheroid into the surrounding collagen matrix were monitored by phase contrast microscopy over a period of two weeks and photo-documented as described above. This experiment was repeated twice.

### 2.11. Analysis of Cellular Energy Metabolism

Oxygen consumption by JUN-1 (*n* = 8), JUN-2 (*n* = 8), JUN-3 (*n* = 11), and JUN-2fos3 (*n* = 9) cells was analysed by high-resolution respirometry in 2 mL glass chambers of oxygraph Oroboros at 37 °C using DatLab software for data acquisition and analysis (Oroboros, Innsbruck, Austria). The oxygen flux was calculated online as a negative time derivative of the oxygen concentration and its values were corrected for instrumental background measured in separate experiments performed in the same medium without cells. After equilibration, the cells were injected into the chambers, mixed, and counted. Respiratory activity of cells was assessed as routine respiration (ROUT; R). State LEAK (L) reflecting intrinsic mitochondrial uncoupling due to the proton leak, proton and electron slip, and cation cycling [34] was measured after inhibition of ATP synthesis by oligomycin (2 µg/mL). Uncoupler trifluorocarbonylcyanide phenylhydrazone (FCCP; 0.05 µmol/L titration steps) was used to induce the state ETS capacity (E), i.e., the maximum capacity of the electron-transporting system. The residual oxygen consumption (ROX) remaining after the inhibition of ETS was determined by antimycin A injection (2.5 µmol/L). In the results, oxygen fluxes recorded in the individual titration steps were corrected for ROX. The results were expressed per IU of citrate synthase (CS) activity reflecting mitochondrial content in cells, assayed in the samples aspirated from each oxygraph chamber [35,36]. The assay medium consisted of 0.1 mmol/L 5,5-dithio-bis-(2-nitrobenzoic) acid, 0.25% Triton-X, 0.5 mmol/L oxalacetate, 0.31 mmol/L acetyl coenzyme A, 5 µmo/L EDTA, 5 mmol/L triethanolamine hydrochloride, and 0.1 mol/L Tris-HCl, pH 8.1 [35]. Then, 200 µL of the mixed and homogenised chamber content was added to 800 µL of the medium. The enzyme activity was measured spectrophotometrically at 412 nm and 30 °C for 200 s and expressed in mIU per 10^6^ cells.

The cell culture medium glucose measurement was performed according to method using a glucose assay kit (GAHK-20, Sigma, Prague, CZ) [37]. Briefly, with this method, glucose is phosphorylated by hexokinase (HK) in the presence of adenosine triphosphate (ATP) to produce glucose-6-phosphate (G-6-P) and adenosine diphosphate (ADP). Glucose-6-phosphate dehydrogenase (G-6-PDH) specifically oxidises G-6-P to 6-phosphogluconate with the concurrent reduction of nicotinamide adenine dinucleotide (NAD) to nicotinamide adenine dinucleotide reduced (NADH). The NADH absorbs light at 340 nm and can be detected spectrophotometrically as an increased absorbance.

Lactate secretory activity was determined using the enzymatic colourimetric assay of conditioned culture media. Medium from each treatment was collected and tested for lactate concentration using an L-lactate Assay Kit (#1200012002, Eton Biosciences Inc., San Diego, CA, USA) [38]. Briefly, L-lactate is oxidised to pyruvate by the specific enzyme lactate oxidase (LOD) and hydrogen peroxide (H_2_O_2_). In the next reaction, enzyme peroxidase (POD) is used to generate a colour dye using the hydrogen peroxide created in the first reaction. The intensity of the colour formed is directly proportional to the L-lactate concentration. It is determined by measuring the increase in absorbance at 552 nm wavelength. Experiments were performed in triplicates and repeated a minimum of two times with similar results.

### 2.12. Transcriptomic Profiling

Total cellular RNA were extracted from dry pellets of JUN-2, JUN-2fos-3, and JUN-3 cells (each cell line in biological triplicates; Figure S3) by mirVana™ miRNA Isolation Kit (Invitrogen™; ThermoFisher Scientific, Carslbad, CA, USA ) according to manufacturer protocol. DNase treatment of extracted RNA was performed by DNA-free™ DNA Removal Kit (Invitrogen™; ThermoFisher Scientific, Carslbad, CA, USA) to remove possible genomic contaminations. Quality and integrity of purified RNA was determined by NanoDrop 2000c (ThermoFisher Scientific, Carslbad, CA, USA) and TapeStation RNA ScreenTape (Agilent Technologies, Santa Clara, CA, USA) to select appropriate RNA samples for analysis. The subsequent synthesis of labelled and fragmented cRNA was performed by GeneChip™ 3’ IVT PLUS Reagent Kit (Applied Biosystems™; ThermoFisher Scientific, Carslbad, CA, USA) according to the manufacturer protocol. Subsequent high-throughput gene expression analyses were performed using the hybridisation technology GeneChip Mouse Genome 430 2.0 Array (ThermoFisher Scientific, Carslbad, CA, USA) according to the manufacturer protocol; intensity values for each probe cell (.cel file) were calculated using Affymetrix GeneChip Command Console (AGCC) software. These arrays cover over 39,000 mouse transcripts. All data were pre-processed and further analysed by the software packages included in the R/Bioconductor, and pre-processing was performed by the RMA method [39]. Complete linkage clustering (farthest neighbour clustering) with Euclidean distance measurements were applied for the visualisation of sample similarities and clusters. As it was impossible to visualise the heatmap structure of all the genes included in the full data matrix of expression data consisting of 22,690 genes, the heatmap was based on expression data of 2000 randomly selected genes. To identify differentially expressed transcripts, the LIMMA approach was applied with additional Benjamini–Hochberg correction of *p* values. Gene set over-representation analysis on upregulated and downregulated gene groups was performed using ConsensusPathDB-mouse (Max Planck Institute for Molecular Genetics in Berlin, Germany, Retrieved from http://cpdb.molgen.mpg.de/MCPDB, (Accessed date on 24 July 2020)) using default settings to detect pathways across various databases (KEGG, MouseCyc, Reactome, and Wikipathways) connected to our gene groups. If the same pathway was detected in various databases, the one with the lowest *p*-value was used. All pathways were sorted in ascending order according to their *p*-value.

### 2.13. Pharmacologic Inhibition of CCL8 Activity

JUN-3 cells were used to test consequences of pharmacologic inhibition of both the ligand CCL8 by Bindarit [40,41] (Abcam ab143292) and of its major receptor CCR5 by Maraviroc [42,43] (Sigma, Prague, CZ), either individually or in combination. The drugs were dissolved in DMSO (Sigma, Prague, CZ) with 50 mM stock solutions. The effects of CCL8–CCR5 inhibition on cell motility was analysed using special migration plate with 8 μm pore membranes (CIM-Plate^®^; used with the xCELLigence^®^ RTCA DP system from ACEA Biosciences, USA; contains electronically integrated Boyden chambers) [44]. Briefly, 20,000 cells in 90 μL of serum-free medium were seeded to each well of the upper chamber, then 10 μL of drug solutions was added (final concentration of inhibitors: 10 μM Maraviroc and 250 μM Bindarit). The wells of the bottom chamber were filled with 160 mL of 10% serum-containing media. The DP instrument with CIM-Plate 16 was placed in a standard CO_2_ incubator. The xCELLigence software was set to collect impendence data (reported as cell index—CI) at every 15 min for 30 h. Finally, we analysed the cell index curves and determined the cell migration activity. Experiments were performed in triplicates and repeated a minimum of two times with similar results. The consequences of either inhibitor on cell invasiveness were evaluated by 3D collagen invasion assay as described above, with the addition of either inhibitor directly into the type I collagen gel (final concentration of inhibitors: 10 μM Maraviroc and 250 μM Bindarit), and monitoring of the invasiveness of JUN-3 spheroids by phase contrast microscopy over a period of two weeks. This experiment was repeated twice.

### 2.14. Additional Statistical Analysis

Statistical analyses and data visualisations were performed in R statistical software [45]. Parametric statistical analyses were extended by permutational (hypothesis testing) or bias-corrected and accelerated bootstrap [46] (estimation of 95% confidence intervals) techniques (10,000–21,000 permutations/resamplings), which do not rely on assumptions of parametric methods and give reliable results even for small sample sizes. Comparisons between groups were performed by permutational ANOVA followed by permutational t-test (exact if N < 11, Monte-Carlo otherwise) with false discovery rate correction for multiple comparisons [47] as a post hoc, both using predictmeans‘ R package [48] and our previously used and published R scripts [49,50]. Heteroscedastic residuals were stabilised by appropriate data transformation. Plots were created using ‘beeswarm’ [51] and ‘vioplot’ [52] R packages.

## 3. Results

### 3.1. Extension of JUN-Sarcoma Progression Series for the JUN-2fos-3 Cell Line

**Figure 1:**
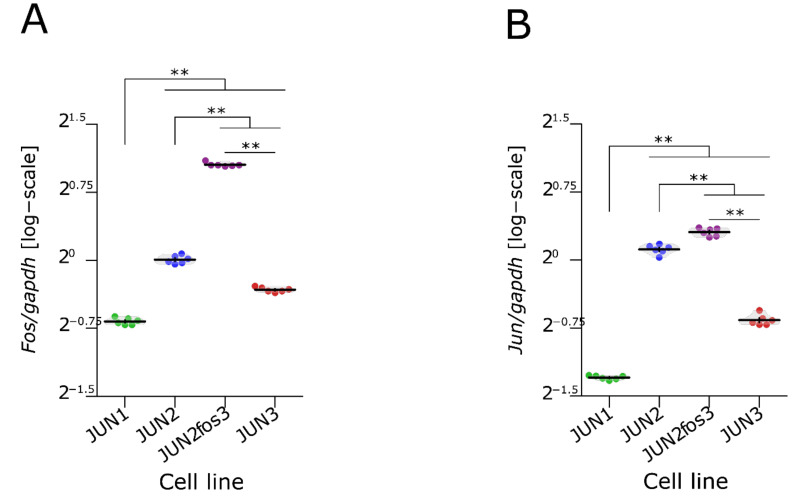
*Fos* and *Jun* expression in fibrosarcoma cell lines. (**A**,**B**) Comparison of *jun* and *fos* expression in different JUN-sarcoma cell lines. Results of permutational ANOVA: F_3,20_ = 1531, ** *p* < 0.01 (*jun* expression) and F_3,20_ = 2948, ** *p* < 0.01 (*fos* expression). See Table S1 for results of contrasts between pairs of lines, their confidence intervals, and exact *p*-values. Each point represents an individual experiment (*n* = 6). Violin plots with means ± SEM are shown.

**Figure 2:**
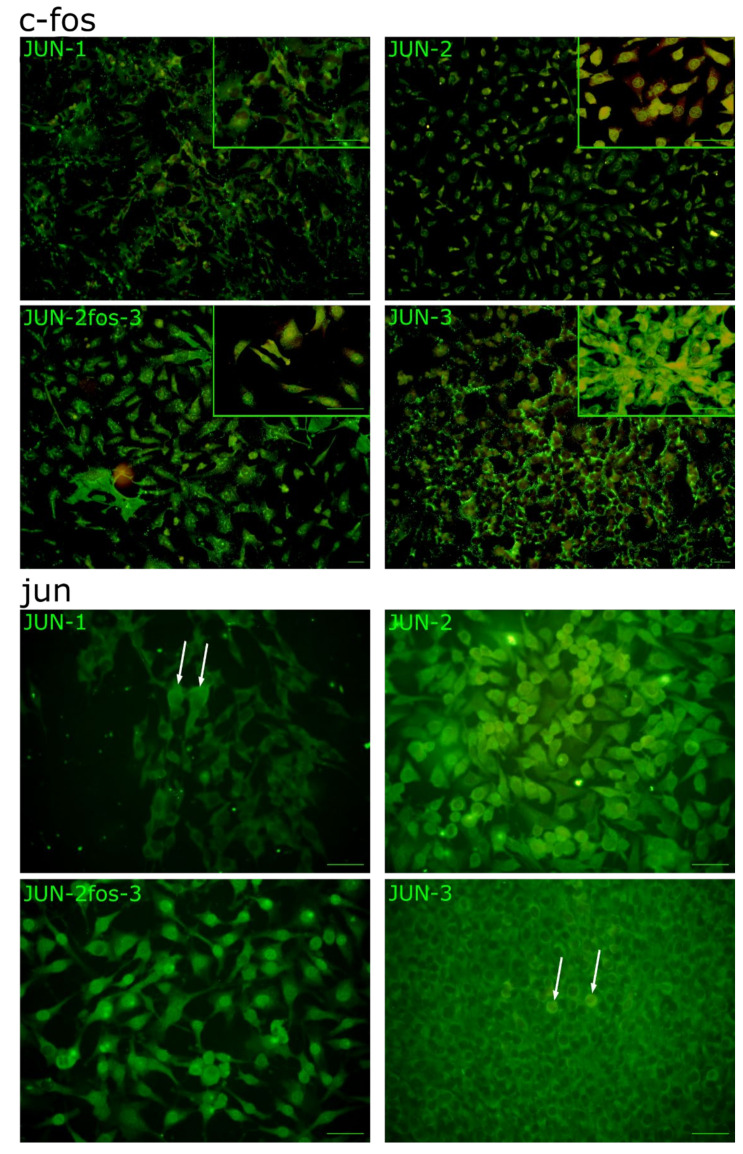
Fos and Jun oncoproteins expression in fibrosarcoma cell lines. Indirect immunofluorescence analysis. Notice the high nuclear expression of both oncoproteins in JUN-2 as well as JUN-2fos-3 cells and their low expression levels in JUN-1 cells. Fos seems to be quite highly expressed in JUN-3 cells as well, with an unusual perinuclear localisation. Both JUN-1 and JUN-3 seem to be generally devoid of appreciable nuclear Jun, except some individual scattered cells (arrows). Pictures were taken by Olympus IX70 fluorescent microscope equipped with the Olympus DP71 camera system (Bar: 100 µm) Negative controls are shown in Figure S2.

The JUN-fibrosarcoma progression series described previously [20] featured a linear gradation of all the transformation-related traits from relatively non-transformed JUN-2 cells, through JUN-1 with an intermediate transformation status to the highly transformed JUN-3 cells. We also noticed an inverse relationship between the *v-jun* oncogene expression and the transformation grade. This relatively high and ubiquitous *v-jun* expression in JUN-2 cells presenting a low transformation status encouraged us to address the possibility of their targeted in vitro transformation by virtue of overexpressing the cooperating oncogene *c-fos*; indeed, we were able to establish a derivative cell line, JUN-2fos-3, with a high level of *c-fos* expression (Figure S1, Table S1). We could verify that both JUN-2 and its new daughter cell line expressed a high level of *jun* oncogenes (*v-jun* and *c-jun*) (Figure 1). As both *jun* and *fos* oncogenes code for nuclear transcription factors, we were also interested in subcellular localisation of the respective oncoproteins. Both JUN-2 and its derivative daughter JUN-2fos-3 displayed prominent high nuclear expression of both fos and jun oncoproteins. Remarkably, we could not see any nuclear c-fos signal in JUN-1 cells, and we saw very unusual subcellular localisation of the c-fos protein in JUN-3 cells, with a very prominent perinuclear localisation, as opposed to the diffuse pan-nuclear staining in JUN-2, and especially in JUN-2fos-3 cells. With regard to the jun expression, our immunofluorescence analysis showed, in addition to the high nuclear expression in both JUN-2 and JUN-2fos-3 cell lines, a low but evident expression in JUN-1 cells, and minimal expression in the bulk of the JUN-3 cells; interestingly, we noticed individual scattered JUN-1 as well as JUN-3 cells showing appreciably high nuclear jun oncoprotein expression, whose identity remains to be established (Figure 2, cells are marked with arrows; Figure S2).

**Figure 3:**
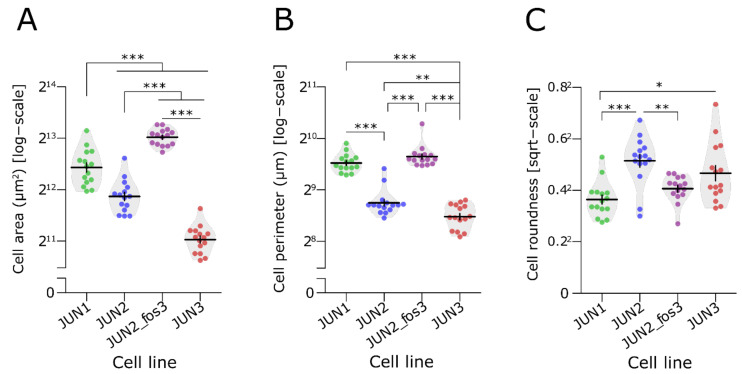
Morphological characteristics of JUN-sarcoma cell lines. (**A**–**C**) Size analysis of single adherent cells of JUN-sarcoma cell lines. Three parameters were evaluated—the total cell area of an adherent cell (μm^2^; permutational ANOVA: F_3,34_ = 3.4, *p* = 0.021), the cell perimeter (μm; permutational ANOVA: F_3,34_ = 45, *p* < 0.001), and the cell roundness (permutational ANOVA: F_3,34_ = 3.8, *p* = 0.018). The JUN-2-fos-3 cell line presented the largest cell size with prominent lamellipodia. * *p* < 0.05, ** *p* < 0.01, *** *p* < 0.001. The statistical significances are based on permutational t-test with FDR correction. See Table S2 for effect sizes, their confidence intervals, and exact *p*-values. Each point represents an individual cell (*n* = 15). Violin plots with means ± SEM are shown.

Morphologically, isolated JUN-2fos-3 cells presented with prominent lamellipodia and the largest cell size, whereas the JUN-3 cells represented the opposite size phenotype, being the smallest of the series (Figure 2 and Figure 3A–C, Table S2).

### 3.2. Proliferation Characteristics

**Figure 4:**
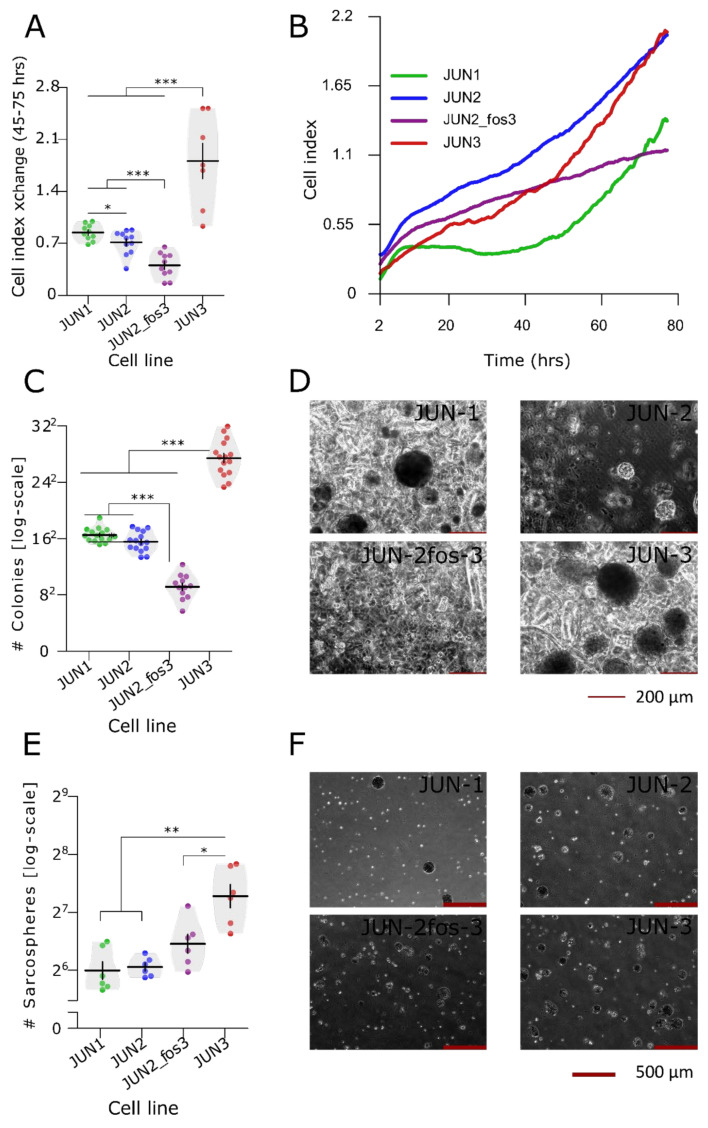
Proliferation and stemness-related characteristics of JUN-sarcoma cell line. (**A**) Proliferation of JUN-sarcoma cell lines. The doubling time and slope analysis (Table 1) were performed on linear growth phase, set arbitrarily as growth curve interval between 45 and 75 h (permutational ANOVA: F_3,34_ = 33, *p* < 0.001). The JUN-3 cell line showed the fastest growth. The proliferative activity of the newly established derivative JUN-2fos-3 was inferior to all the other sarcoma cell lines of the series, even below the proliferative activity of its parental cell line JUN-2. (**B**) Growth curve of the JUN-sarcoma cell lines. The proliferative activity of the newly established derivative JUN-2fos-3 was inferior to all the other sarcoma cell lines of the series, even below the proliferative activity of its parental cell line JUN-2. (**C**) Clonogenicity in semisolid media differ among JUN-sarcoma cell lines (permutational ANOVA: F_3,53_ = 252, *p* < 0.001). JUN-3 was the most active cell line, whereas the JUN-2fos-3 showed the weakest clonogenicity. Representative pictures of colonies formed in 15% methylcellulose are shown in (**D**). The pictures were taken by the Olympus IX 70 inverted microscope equipped with the Hamamatsu Orca-ER camera at 100× magnification. (**E**) Sarcosphere formation capacity differed among JUN-sarcoma cell lines (permutational ANOVA: F_3,20_ = 14.8, *p* < 0.001). Both JUN-2fos-3 and JUN-3 presented rather high sarcosphere formation activity. Representative pictures of spheres are shown in (**F**). The pictures were taken by the Olympus IX 70 inverted microscope equipped with the Hamamatsu Orca-ER camera at 40× magnification. * *p* < 0.05, ** *p* < 0.01, *** *p* <0.001 (A,C,E, respectively). The statistical significances are based on a permutational *t*-test with FDR correction. See Table S3 for effect sizes, their confidence intervals, and exact *p*-values. Each point represents an individual well. Violin plots with means ± SEM are shown.

We applied two assays to evaluate the proliferative activity of the cells. Proliferation in a two-dimensional culture setting was quantified with the aid of two variables—the doubling time and the slope of the exponential growth phase. The results of this analysis are shown in Figure 4A,B, Table 1 and Table S3. We verified the previous results obtained with simpler methodology for JUN-1, -2, and -3 cell lines [20], with JUN-3 being the fastest growing cell line and JUN-1 and JUN-2 showing an intermediary growth intensity. The proliferative activity of the newly established derivative JUN-2fos-3 was inferior to all the other sarcoma cell lines of the series, even below the proliferative activity of its parental cell line JUN-2.

Proliferation in three-dimensional culture was analysed as anchorage-independent growth by evaluating clonogenicity in methylcellulose-containing medium. We evaluated two characteristics—the total number of colonies and their size. We saw the same distribution of sarcoma cell lines as in the two-dimensional culture. JUN-3 was by far the most active cell line in both the total colony number and their size (Figure 4C,D, Table S3), whereas JUN-2fos-3 showed the weakest proliferation activity in the methylcellulose clonogenicity assay. JUN-1 and JUN-2 reached practically identical colony numbers, with distinctly larger colonies produced by the JUN-1 cell line.

### 3.3. Sarcosphere Formation and Clonogenic Activity Is Not Associated with Apparent Side Population

The results of the anchorage-independent growth can be interpreted in two ways. Apparently, the clonogenicity in a semisolid medium may reflect a combination of the proliferative activity and evasion from anoikis, and the overall excellent correlation between the steepness of growth curves in a classical two-dimensional culture and the clonogenicity in methylcellulose suggests that the general proliferative activity could indeed be essential for the anchorage-independent growth of the JUN-sarcoma cell lines. On the other hand, clonogenicity in semisolid media is also increasingly viewed as an assay for cancer stem cells [53,54], and in this case, differences in the clonogenicity in methylcellulose among the JUN-sarcoma cell lines could be more indicative of differences in a relative frequencies of sarcoma stem cells. To resolve this question, we performed the sarcosphere assay (Figure 4E,F, Table S3). Remarkably, the JUN-2fos-3 cell line showed the second highest sarcosphere formation efficiency, indicating that its poor clonogenicity in methylcellulose could be rather attributed to its overall low proliferation activity. The significantly highest sarcosphere formation efficiency was observed in the JUN-3 cell line, which thus couples a high proliferation intensity with the relatively highest frequency of sarcoma stem cells (Figure 4E, Table S3).

On the other hand, we were unable to evidence any appreciable side population in JUN-3 cell line (data not shown); the biological underpinnings of clonogenic and sarcosphere-founding cells thus remains to be further characterised.

### 3.4. JUN-2fos-3 and JUN-3 Cell Lines Are Highly Motile and Invasive

**Figure 5:**
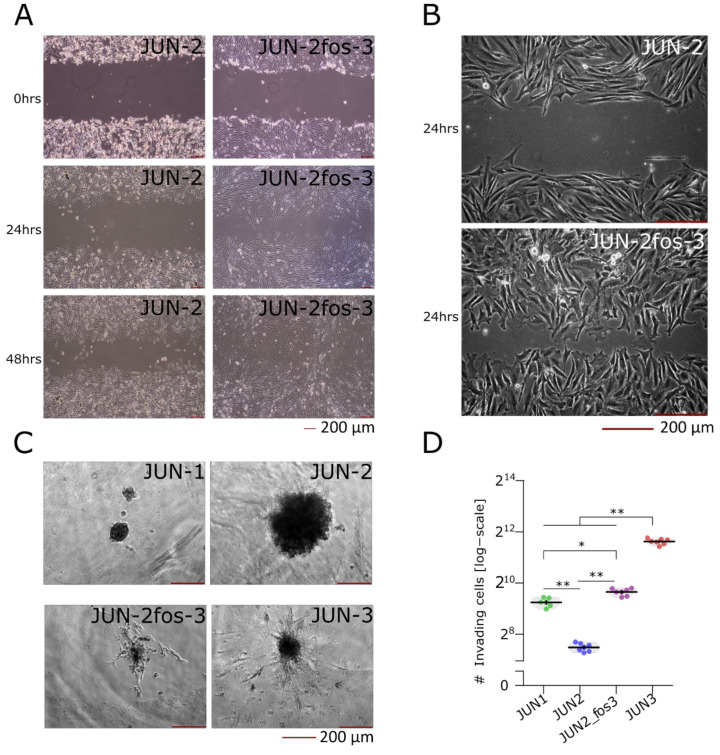
Invasion-related characteristics of JUN-sarcoma cell line. (**A**–**D**) JUN-2fos-3 cells are highly motile and invasive. (**A**) Cell motility after 24 and 48 h in vitro wound-healing test. The newly established JUN-2fos-3 cell line was highly motile in the in vitro wound-healing assay compared to its mother cell line JUN-2. Representative pictures were taken by the Olympus IX 70 inverted microscope at 40× magnification (at 100× magnification in detail (**B**)). (**C**) Invasion of multicell tumour spheroids of JUN-sarcoma cell lines embedded into type I collagen at 100× magnification. JUN-3 and JUN-2fos-3 cell lines showed comparatively intensive invasion, whereas the JUN-2 cell line was completely non-invasive. JUN-1 cell line displayed minimal invasiveness in type I collagen. (**D**) Matrigel in vitro invasion assay differed among JUN-sarcoma cell lines (permutational ANOVA: F_3,23_ = 910, *p* < 0.001). Both the JUN-2fos-3 and the JUN-3 cell lines showed comparatively intensive invasion in this assay. * *p* < 0.05, ** *p* < 0.01 The statistical significances are based on permutational *t*-test with FDR correction. See Table S4 for effect sizes, their confidence intervals, and exact *p*-values. Each point represents an individual well. Violin plots with means ± SEM are shown.

Besides the growth characteristics, we were also interested in the progression characteristics of JUN-sarcoma cell lines; as noted above, our initial characterisation of JUN-1, -2, and -3 cell lines revealed a perfect correlation between growth and progression-related transformation characteristics [20]. A surprising finding was that the newly established JUN-2fos-3 cell line was highly motile in the in vitro wound-healing assay (Figure 5A,B). Although the invasiveness expressed as the total number of cells invading the Matrigel-coated insert and adhering on its bottom in the Matrigel invasion assay were 3.92 times lower than for the highly invasive JUN-3 cell line (Figure 5C,D, Table S4), we noticed that for both the JUN-2fos-3 and JUN-3, the bottom of the Matrigel insert was covered by a confluent cell layer, and indeed, this coefficient was practically identical to the size relation between the JUN-2fos-3 and JUN-3 cells (adherent cell area of JUN-2fos-3 is 3.85 times larger than that of JUN-3 cells). Therefore, we hypothesised that the observed difference in the Matrigel invasion was due to the difference in the cell size of JUN-3 and JUN-2fos-3. To corroborate this notion, we performed an independent invasion assay that could not be influenced by differences in the cell size, namely the three-dimensional invasion assay of spheroids embedded in type I collagen. Both the JUN-2fos-3 and the JUN-3 cell lines showed comparatively intensive invasion in this assay (Figure 5C). The JUN-1 cell line displayed an intermediate invasion in the Matrigel invasion assay and minimal invasiveness in spheroids embedded in type I collagen, whereas the JUN-2 cell line was completely non-invasive in both the invasion assays.

### 3.5. Invasive Cell Lines Have Different Metabolic Profiles

**Figure 6:**
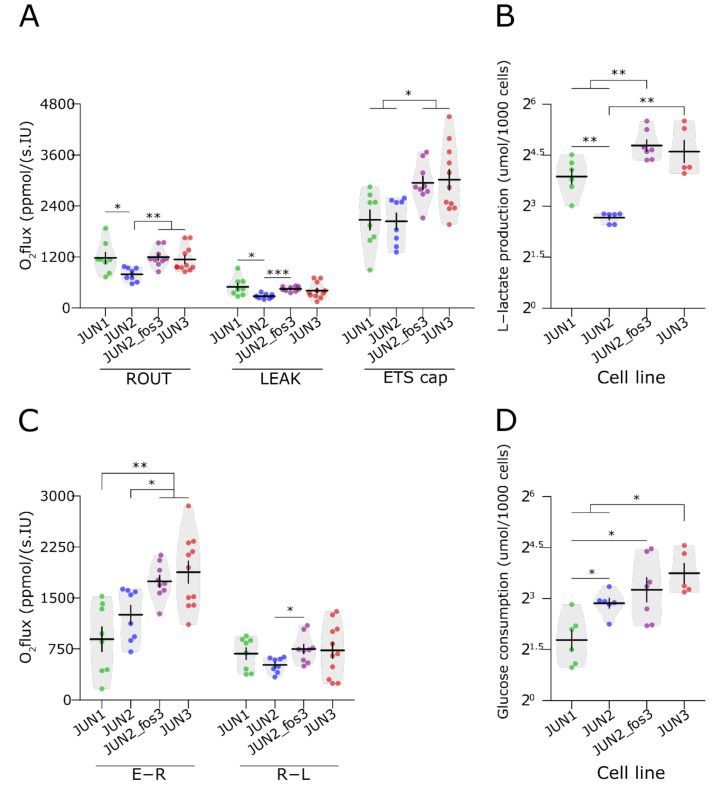
Metabolic analysis of JUN-sarcoma cell lines. (**A**,**C**) Mitochondrial oxygen consumption in JUN-sarcoma cell lines. Cell lines differed in oxygen consumptions in state R (permutational ANOVA: F_3,32_ = 4, *p* = 0.015), L (permutational ANOVA: F_3,32_ = 3.2, *p* < 0.032), and E (permutational ANOVA: F_3,32_ = 6, *p* = 0.0021), and also differed in E-R capacity (permutational ANOVA: F_3,32_ = 9.5, *p* < 0.001) but not R-L capacity (permutational ANOVA: F_3,32_ = 1.3, *p* = 0.3). The JUN-2fos-3 cell line displayed significantly higher oxygen consumption in the states R, L, and E compared to the least transformed non-invasive, non-motile JUN-2 cells. The spare respiratory capacity (excess E-R capacity) was higher in both invasive and motile cell lines, JUN-3 and JUN-2fos-3, than in cells with limited motility and invasiveness, JUN-1 and JUN-2. (**B**) The production of L-lactate differed among JUN-sarcoma cell lines (µmol/1000 cells; (permutational ANOVA: F_3,20_ = 25, *p* < 0.001). The JUN-2 cell line had a relatively high capacity of oxidative phosphorylation and the lowest production of lactate, suggesting that ATP is generated especially aerobically through the respiratory chain. (**D**) The consumption of glucose differed among JUN-sarcoma cell lines (µmol/1000 cells; (permutational ANOVA: F_3,23_ = 9.9, *p* < 0.001). Both the invasive cell lines JUN-2fos-3 and JUN-3 combined relatively high oxphos parameters with the highest production of lactate and consumption of glucose, taking advantage of both pathways of energy production. * *p* < 0.05, ** *p* < 0.01, *** *p* < 0.001. The statistical significances are based on permutational *t*-test with FDR correction. See Table S5 for effect sizes, their confidence intervals, and exact *p*-values. Each point represents an individual experiment. Violin plots with means ± SEM are shown. (ROUT: (R)—resting respiration of intact cells, LEAK (L)—oxygen consumption essential for compensation for the proton leakage, ETS cap (E)—uncoupled respiration, i.e., maximum capacity of the electron-transporting system. R-L—ATP-linked oxygen consumption, E-R—spare respiratory capacity).

Figure 6A,C and Table S5 depict the values of respiratory states of all cells under investigation. Interestingly, JUN-2fos-3 cells displayed significantly higher oxygen consumption in the states R, L, and E compared to the least transformed non-invasive, non-motile JUN-2 cells. Accordingly, their respiration related to mitochondrial ATP production calculated as a difference between routine and leak respiration (R-L; free routine activity) did not differ from that measured in the most transformed JUN-3 cells. In addition, the spare respiratory capacity (excess E-R capacity), i.e., the difference between the fully uncoupled and routine cellular oxygen consumption, was higher in invasive and motile cell lines (JUN-3 and JUN-2fos-3) than in cells with limited motility and invasiveness (JUN-1 and JUN-2).

If expressed per 10^6^ cells, the JUN-2 cell line had a relatively high capacity of oxidative phosphorylation and the lowest production of lactate (Figure 6B, Table S5), suggesting that ATP is generated, especially aerobically, through the respiratory chain. In contrast, JUN-2fos-3 cell line displayed high oxphos and electron-transporting capacities and a high production of lactate in parallel. The most transformed JUN-3 cell line combined relatively high oxphos parameters with the highest production of lactate and consumption of glucose, taking advantage of both pathways of energy production. Accordingly, the glucose consumption rate was also significantly higher in both invasive sarcoma sublines (Figure 6D, Table S5).

Citrate synthase activity was the highest in JUN-2fos-3 cell line (77.2 ± 10.9 mIU/10^6^ cells), comparable in JUN-2 and JUN-3 cells (62.1 ± 25.2 and 51.3 ± 20.6 mIU/10^6^ cells, respectively), and the lowest in JUN-1 cells (27.4 ± 16.7 mIU/10^6^) with intermediary proliferation, motility, and invasiveness characteristics.

### 3.6. Distribution of Transformation Traits among JUN Fibrosarcoma Cell Lines Allows for the Straightforward Identification of Genes Potentially Responsible for Sarcoma Cell Proliferation and Motility/Invasiveness

**Figure 7:**
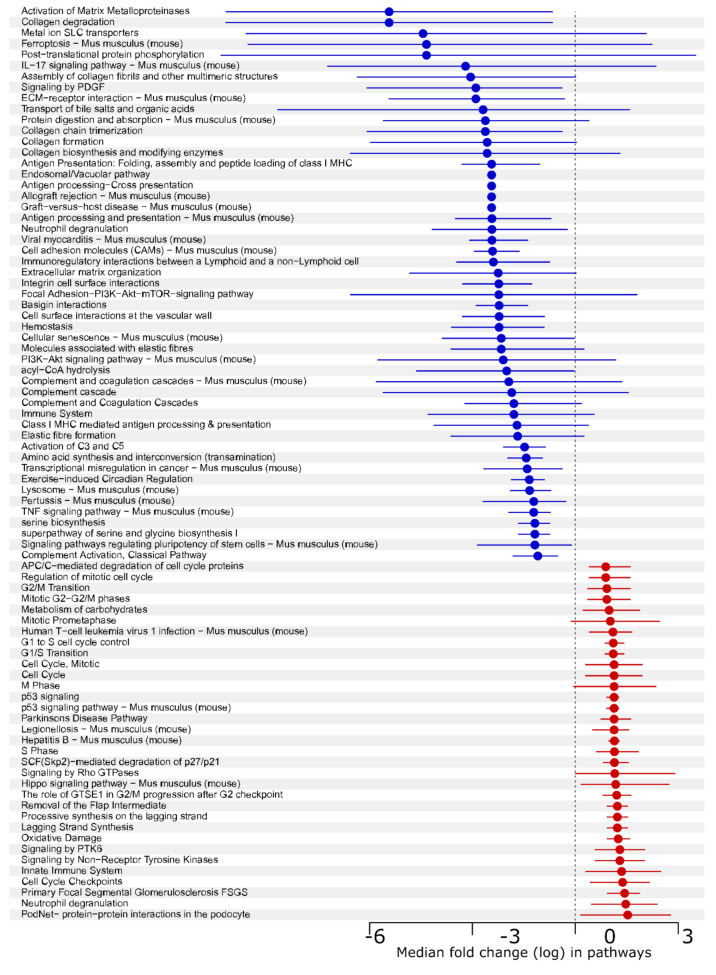
The gene set enrichment analysis of JUN-sarcoma cell lines. Median log (fold change) with 95% confidence interval in pathways sorted by fold change. Downregulated pathways are shown in blue, whereas upregulated pathways are shown in red colours. Analysis revealed that the downregulated genes are dominated by extracellular matrix and cell adhesion, as well as antigen presentation, whereas among upregulated pathways, those related to cell cycle regulation and DNA replication are particularly frequent (see Table S7 The gene set enrichment analysis for complete list of genes in each pathway).

These unique combinations of transformation-related traits made it possible for us to identify two separate groups of genes tentatively involved in sarcoma progression in a single transcriptomic analysis—on the one hand, proliferation-related genes could be identified by their differential expression in JUN-3 compared to both JUN-2 and JUN-2fos3, and, on the other hand, motility and invasiveness-related genes could be identified by their common expression pattern in JUN-2fos3 and JUN-3 cells compared to JUN-2 (Table 2). The former will be addressed in a separate article, and for the remainder of this article, we focus on genes potentially underlying the invasive character of JUN-2fos3 and JUN-3 cell lines. Starting with individual comparisons of each of the invasive sarcoma cell lines with the reference cell line JUN-2 (Tables S9 and S10), we finally arrived at a common transcriptomic profile in both the invasive cell lines. In total, we identified 126 genes that were significantly upregulated and 249 genes that were significantly downregulated in the motile/invasive fibrosarcoma cell lines JUN-2fos3 and JUN-3 compared to JUN-2 (Table S6). The gene set enrichment analysis (Figure 7 and Table S7) revealed that the downregulated genes are dominated by extracellular matrix and cell adhesion, as well as antigen presentation, whereas upregulated pathways, surprisingly, involve an unexpected number of molecular pathways related to cell cycle regulation and DNA replication.

### 3.7. CCL8 Represents a Druggable Target to Curtail Motility and Invasion

**Figure 8:**
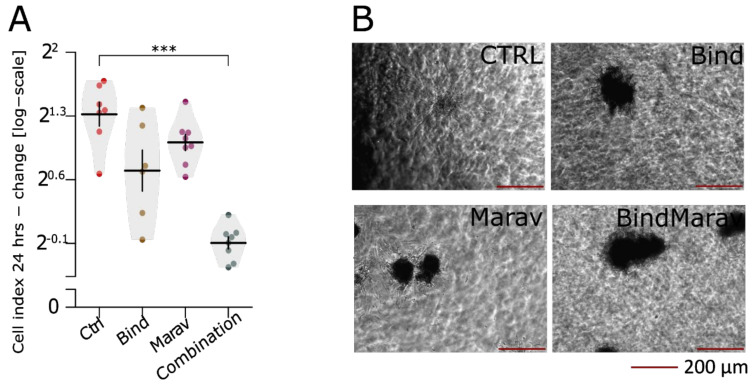
Pharmacological inhibition of Ccl8 - Ccr5 signalling entails a significant decline in invasive capacity. Cells were treated with Bindarit (Ccl8 inhibitor, 250 µM), Maraviroc (Ccr5 inhibitor, 10 µM), or their combination and their invasiveness was analysed by xCelligence motility assay (**A**) and by the invasion of multicell tumour spheroids of JUN-sarcoma cell lines embedded into type I collagen identically to or. (**B**) Linear model revealed that both Bindarit (ẞ = −0.89 (95% CI: −1.15, −0.69), *p* < 0.001) and Maraviroc (ẞ = −0.54 (−0.82, −0.26), *p* < 0.001) significantly decreased the motility of JUN-3 cells, but we were not able to detect significant effect of their interaction (ẞ = −0.48 (−1.04, 0.08), *p* = 0.072). *** *p* < 0.001. The statistical significances are based on permutational *t*-test with FDR correction. See Table S8 for effect sizes, their confidence intervals, and exact *p*-values. Each point represents an individual well. Violin plots with means ± SEM are shown.

As an initial proof of conceptual correctness and usefulness of our transcriptomic screen, we chose CCL8 to test for pharmacological targeting of motility and invasiveness. We were lead in our choice mainly by the availability of clinically applicable pharmacologic inhibitors for both the ligand by Bindarit [55], and its major receptor CCR5 by Maraviroc [56]. Both inhibitors were able to substantially decrease both the motility of JUN-3 cells in the real-time xCELLigence^®^ RTCA DP system assay (Figure 8A, Table S8) and the invasion of JUN-3-derived multicellular spheroids into type I collagen gels (Figure 8B), with an indication for their additive effects upon drug combination.

## 4. Discussion

In the previous report [20], we established three sarcoma cell lines from two consecutive sarcomas initiated in a single female *H2-K/v-jun* transgenic mouse. These cell lines—JUN-1, -2, and -3—exhibited a gradual level of transformation, with JUN-2 being the least transformed cell line, JUN-3 being highly transformed, and JUN-1 presenting with an intermediate transformation status; strikingly, both proliferation and progression (motility, invasiveness) characteristics followed this distribution in each cell line. The expression of the *v-jun* oncogene displayed an inverse relationship to the transformation, which prompted us to speculate that the *v-jun* transgene expression merely provided an initial trigger for sarcoma development in this transgenic model system.

In an attempt to further develop this progression series, we reasoned that the least transformed cell line JUN-2 could be, by virtue of its high *v-jun* expression [20], an excellent candidate for a further in vitro transformation by the *c-fos* oncogene. Oncoproteins of the jun and fos families, together forming the oncogenic transcription factor AP-1, have a particular relevance in sarcoma biology [57,58]. Both oncogene families were founded by viral oncogenes of acutely transforming retroviral strains, and both of them initiated sarcomagenesis in susceptible animal hosts (chicken fibrosarcoma in the case of *v-jun* and murine osteosarcoma for *v-fos*). This predilection for transformation of mesenchymal lineage was preserved in the respective transgenic mice; transgenic *c-fos* overexpression resulted in osteosarcomas [59], whereas *v-jun* overexpressing transgenic mice developed fibrosarcomas secondary to deep wounding [21]. The *c-jun* has been indeed verified as a bona fide human oncogene, by discovering its amplification in a non-negligible proportion of aggressive dedifferentiated liposarcomas [60,61].

Indeed, we were able to establish a derivative cell line, JUN-2fos-3, with a high level of c-fos oncoprotein expression. Overall, we could see a very good correlation between respective quantitative mRNA expression level and a more qualitatively conceived immunofluorescence analysis (Figure 1 and Figure 2), indicating that transcription is the primary regulatory level for both oncogenes. Nevertheless, we also found indications for the existence of additional regulatory mechanisms. Especially conspicuous is the observation of the prominent perinuclear localisation of c-fos in JUN-3 cells. Strikingly in this respect, c-fos has been described as a lamin A-interaction protein, leading to its affinity towards nuclear lamina [62], which can be modulated by mitogenic signalling [62,63]; whether this can explain our immunofluorescence finding and inasmuch as this could impact the high transformation status of JUN-3 sarcoma cells awaits further analyses. Another point deserving a short discussion is the heterogeneity within the sarcoma cell population, especially remarkable for the jun oncoprotein in JUN-1 and JUN-3 cells. As for the latter, we have preliminary evidence that the frequency of nuclear jun-positive cells significantly increases in cells treated with the leukaemia inhibitory factor and connective tissue growth factor, respectively, in both cases accompanied by marked increase in anchorage-independent clonogenicity. This suggests that the JUN-3 cells featuring high nuclear jun expression could correspond to clonogenic stem-like cells.

The newly derived JUN-2fos-3 sarcoma cell subline is especially remarkable by its uncoupling of proliferation on the one hand, and its motility/invasiveness on the other hand, which was essential to our aim to unravel a complex molecular basis of sarcoma motility/invasiveness, as a crucial and rate-limiting step in sarcoma progression and metastatic dissemination. Two specific aspects stand out in this respect—the specific metabolic adaptation and a specific signalling context as revealed by the specific transcriptomic profile.

As for the former, the targeted overexpression of c-fos in JUN-2fos-3 cells resulted in a markedly affected pattern of the cellular energy metabolism compared to the relatively non-transformed JUN-2 cells. Besides enhanced mitochondrial oxygen consumption, the cells also featured increased dependence on glycolytic energy production. The combination of the two ATP-generating pathways approached the metabolic profile of JUN-2fos-3 cells to the most transformed JUN-3 cell line. The deregulation of cellular energetics with changing patterns of glycolytic and mitochondrial contributions in relation to the degree of transformation is not a new finding, although the precise role of these alterations in the chemoresistance, metastasis, and cancer aggressiveness is yet not fully understood [64].

In any case, the JUN progression series is somewhat reminiscent of a previously analysed entirely in vitro-based fibroblastic progression, where an increase in tumourigenic potential was initially associated with the increasing levels of markers of mitochondrial biogenesis and citric-acid cycle metabolites to switch over to increased dependency on glycolysis for energy production in highly transformed sarcoma cells [19]. Analysis of an analogical transformation series of human mesenchymal stem cells (MSCs) suggested that during transformation, not only did MSCs not undergo a similar metabolic reprogramming, but their dependency on oxidative phosphorylation was even increased, and glycolysis served only as a backup energy supply in case of hypoxia [18]. Our data obtained from largely genuine sarcoma cell lines draw a more complex picture, with concurrent activation of both the glycolytic pathway and mitochondrial respiratory chain in highly invasive sarcoma cells.

The most remarkable metabolic commonality of both the invasive sarcoma cell lines JUN-2fos-3 and JUN-3 is the increased spare respiratory capacity. There are just a few reports, and none in the sarcoma field, connecting increased spare respiratory capacity and invasiveness. Such observations have been reported in invasive and metastatic ovarian cancer cell lines [65], as well as a specific aspect of Krüpel-like factor 4-induced invasiveness of glioblastoma cells [66,67]. There is still a vivid debate about the exact physiological interpretation of the spare respiratory capacity [68]. It may reflect an ability to increase energy production in response to a sudden need, such as stress, and it can also be interpreted as a synthetic expression of the bioenergetics fitness of the cell. Indeed, active cell locomotion is unthinkable without an immediate supply of energy, and this correlation between enhanced spare respiratory capacity and motility and/or invasiveness could be biologically meaningful. As is the case in other experimental systems, it is rather difficult to relate this complex metabolic phenotype with particular genes, as revealed in our transcriptomic analysis. On the one hand, the transcriptomic profile of invasive sarcoma cells revealed increased expression of both hexokinase-1 (*Hk-1*) and phosphofructokinase-P (*Pfk-p*), two important enzymes of glycolytic pathway, in good agreement with their extra glycolytic proficiency. On the other hand, the downregulation of *Nupr1*, *Psph,* and *Tgfb* genes was observed in their transcriptome; these genes are part of a specific 10 gene signature of defect mitochondria that has been recently reported in hepatocellular carcinoma [69], which could be interpreted as a reflexion of greater mitochondrial fitness exhibited by invasive sarcoma cell lines. Together with the pyruvate supplied by the activated glycolytic pathway, this provides a plausible mechanistic explanation for their enhanced spare respiratory capacity. Even less clear is their regulatory context. Taking a lesson from the glioblastoma experimental system cited above, as well as from the largely complementing and overlapping metabolic impact of all the pluripotency transcription factors [70], we can hypothesise that the increased expression of *Sox-2* found in our invasive sarcoma cell lines could be the crucial regulatory factor. On the other hand, our pathway analysis (Figure 6 and Table S6) disclosed a downregulation of pluripotency stemness along with a concomitant upregulation of Hippo-pathway stemness, and indeed, *Sox-2* can be viewed as a crucial factor in both these stemness circuits [71,72]. Supporting this notion, both *Hk-1* and *Pfk-p* have also been described as Yap-downstream genes [73].

Indeed, the transcriptomic profile of fibrosarcoma cell invasiveness that emerged from our analysis of the JUN-progression series indicates a far more complex interplay involving a plethora of factors, as opposed to a great part of previous studies aimed at deciphering the molecular framework of cancer (especially sarcoma) cell invasiveness, which concentrated each on a small number of “strong” factors, either taken a priori by a candidate gene approach, or extracted from genomic profiling analyses. Interestingly, we have little evidence for an essential role of any proteolytic enzymatic activity, in concert with findings reported independently on a rat sarcoma progression series [16] that strongly argued that it is the cell motility that constitutes a rate-limiting ability of the invasive phenotype in sarcoma; in fact, the gene most profoundly downregulated in the invasive sarcoma sublines codes for the matrix metalloproteinase 13, and *Mmp-3* expression is diminished as well. Among the genes overexpressed in our invasive transcriptomic profile, we can find several known activators of cell motility/invasiveness and/or genes independently associated with progression in soft tissue sarcoma (*BIRC5* coding for surviving [74], *RHAMM* [75]) or other cancer types, such as *CCL8* (breast carcinoma [76], melanoma [77]), *Tetraspanin 2* (lung carcinoma [78]), Protein Phosphatase and Actin Regulator 1 (breast carcinoma [79]), *Semaphorin 3A* (glioblastoma [80]), or *FOXD1* (osteosarcoma [81], lung carcinoma [82], melanoma [83]); importantly, in the context of the overall strategy of our transcriptomic screen, at least for melanoma, FOXD1 was described as a pure motility/invasiveness factor, with little impact on cell proliferation [83].

With regard to the CCL8, the major difference in our results and the results from the other studies cited above is that, whereas in both breast carcinoma [76] and melanoma [77] this activating chemokine is provided by activated cancer stroma, this paracrine motility regulation switched to autocrine in our invasive sarcoma cells. Indeed, the tumour microenvironment can be both the source of the inflammatory chemokines including CCL8, as well as the major recipient of their signals, as revealed by analysis of a chemically induced fibrosarcoma model, where increased chemokine expression could be correlated with the recruitment of regulatory T-cells and local immunosuppression [84]. Likewise, effects of the pharmacologic inhibition of the CCL8-CCR5 signalling pathway in various cancer models, either with the CCL8 inhibitor Bindarit [85,86], or by inhibiting its major receptor CCR5 by Maraviroc [42,87,88], has been thus far, for the most part, attributed to their complex effects on the tumour microenvironment, e.g., by diminution of cancer-related inflammation and myeloid or suppressor T-cell cell infiltration, or by limiting accumulation of cancer-associated fibroblasts. Both the inflammatory chemokines and the CCR5 receptor have recently been attributed to cancer cell-autonomous effects, nevertheless, they are notable as activators of cell motility and invasiveness, and, accordingly, both Bindarit and Maraviroc exert direct effects on these transformation traits [89,90,91,92]. Our results are, to our knowledge, the first showing similar cell-autonomous effects of both drugs in sarcoma and, thus, warrant further experimental and translational research effort in this direction.

Semaphorin 3A is another autocrine motility factor, as already described in glioblastoma [80]; it is worth noting that the initial identification of the Semaphorin 3A–Neuropilin-1 (a canonical constituent of the Semaphorin 3A receptor) signalling system as an activator of cell motility in glioblastoma resulted initially from a systematic proteomic screen performed in the HT1080 human fibrosarcoma cells [80], providing an independent confirmation of our results. In fact, the Semaphorin 3A has been described as a motility factor even for normal mesenchymal cells, like vascular smooth muscle cells [93].

On the other hand, signalling consequences of the Semaphorin 3A are remarkably pleiotropic, and one of its strongest effects is a pronounced angiogenesis inhibition [93]. It would seem to be counterintuitive that any cancer cell line progression series would activate a strong antiangiogenic program, and indeed, we can find in our invasive transcriptomic profile at least two established angiogenic activators—*Angiopoietin-2* [94] and *c-fos–*induced growth factor coding for vascular endothelial growth factor D [95]. The latter has been traditionally regarded as an activator of lymphangiogenesis, which is presumed to have a marginal impact (albeit significant within a small fraction of cases [96]) in sarcoma biology, as sarcomas preferentially disseminate via blood vasculature. Recent findings have somewhat questioned this traditional view, nevertheless, they have shown that VEGFD is also involved in vascular angiogenesis and, moreover, one of the VEGFD receptors, VEGFR3, has been found to be expressed on the very tumour cells in a fraction of soft tissue sarcomas, with a significant negative prognostic relevance [97]. Indeed, VEGFD has been repeatedly described as a direct motility factor for both activated fibroblasts [98] and various sarcoma cells, like chondrosarcoma [99] or Kaposi sarcoma [95].

Intriguingly, the same dual role, i.e., angiogenic activator counterbalancing the Sema 3A-induced antiangiogenic action and being a direct motility/invasiveness activator, has been ascribed to the Angiopoietin-2 as well. Ang-2, by engaging the specific receptor tyrosine kinase Tie-2, is crucial for vascular remodelling at sites of active vessel sprouting; this activity relies on simultaneous presence of VEGFs and its specific therapeutic inhibition is actually actively pursued and clinically tested [100]. At the same time, nevertheless, Ang-2 can directly stimulate motility/invasiveness of both monocytes/macrophages [101] and tumour cells [102], by serving as an adhesion ligand to various integrins. Ang-2-activated macrophage motility/invasion is directed towards fibrin clots that may result from the vascular sprouting itself, and it generates a specific fibrin degradation product, D-dimer, whose negative prognostic relevance has been described for many cancer types, including sarcomas [103,104]. The Ang-2 receptor mediating this fibrinolytic activity of macrophages is Integrin β_2_, which is traditionally viewed as a specialised leukocyte integrin. Quite unexpectedly in this respect, and not without interest, the Integrin β_2_ is a part of our sarcoma invasiveness transcriptomic profile as well.

All in all, this suggests a remarkable network of cooperating, antagonizing, and compensating biological activities, co-opted from various normal as well as transformed cell types, which collectively underlie sarcoma cell motility and invasion in our model, and which call for a similar paradigmatic change in viewing this process, from individual “strong” factors regarded in isolation to such molecular networks.

Interestingly, among the genes downregulated in invasive sarcoma cells, the gene showing the second highest fold diminution of expression was *Xist*, coding for a long non-coding RNA, which is crucial for X-chromosome inactivation in female mammals. The role for long non-coding RNAs in tumourigenesis is increasingly appreciated [105], and as for Xist, strikingly conflicting results have been reported, even within the context of a single tumour type like osteosarcoma [106,107]. Importantly, Xist-targeted deletion in foetal haematopoietic stem cells is dramatically protumourigenic [108], resulting in fully penetrant, female-specific carcinogenic transformation encompassing chronic myelomonocytic leukaemia, myeloproliferative disease, and a rare haematologically derived sarcoma—histiocytic sarcoma. It is tempting to speculate that we witness a milder version of a similar protumourigenic effect in our sarcoma model resulting from a diminished expression rather than a hard mutation; it should be noticed in this regard that the entire JUN-fibrosarcoma progression series originated from a single female mouse [20]. On the other hand, we did not see a widespread activation of X-linked oncogenes in our model, a mechanism proposed for the leukaemogenic mouse model cited above; in fact, from the overexpressed genes discussed above, only the gene coding for VEGFD is X-linked, suggesting another molecular mechanism. Another interesting point is the mechanism of *Xist* downregulation. It has been reported that pluripotency transcription factors are major *Xist* repressors in embryonic stem cells [109]; our transcriptomic screen revealed Sox-2 among the activated genes in invasive sarcoma cell lines and, possibly, a similar role of a stemness factor could also be ascribed to FOXD1, at least in the context of induced pluripotent stem cells [110]. On the other hand, as noted above, the pathway analysis suggested a general downregulation of the pluripotency stemness pathway; thus, the biological relevance of the mechanism suggested above remains uncertain.

## 5. Conclusions

In conclusion, we believe that our fibrosarcoma progression model and the differentially expressed genes identified by its transcriptomic analysis can provide important new information on biology of soft tissue sarcoma progression. We showed that motility/invasiveness is a druggable target, fitting into the current concept of migrastatics as a new class of pharmacological weapons to combat metastasizing cancer [111,112]. Importantly, in addition to CCL8 inhibitors used paradigmatically here, there are pharmacologic inhibitors available for several other molecules identified in our transcriptomic screen, like Sema3A [113], Ang-2 [100], or survivin [114]. Moreover, our results strongly suggest that any pharmacological intervention must take into account the complex relationship between the different signalling molecules; the Sema3A inhibition would thus probably only be thinkable if combined with an antiangiogenic therapy. In any case, we believe that the presently studied series of sarcoma progression cell lines will be an elegant model to explore novel therapeutic targets, potential drug candidates, and prognostic markers in the near future. On the other hand, the current analysis is based entirely on in vitro experimental approaches, and an extension to the in vivo system would be desirable and is intended in future research. As mentioned above, this article focuses exclusively on exploiting the JUN-fibrosarcoma progression series for the identification of potential invasiveness markers and therapeutic targets, and we have good indications that the complementary part of our transcriptomic analysis resulting in the identification of proliferation-related genes (Table 2) bears a great potential for improving our understanding of complex sarcoma biology as well.

## Figures and Tables

**Table 1:** Growth characteristics of the JUN-sarcoma cell lines.

Cell Line	Doubling Time (h)	Slope of the Exponential Growth Phase
JUN-1	9.5	0.032
JUN-2	10.4	0.029
JUN-2fos-3	30.9	0.010
JUN-3	7.4	0.041

**Table 2:** Overall strategy of transcriptomic analysis. The distribution of transformation traits among JUN-2, JUN-2fos-3 and JUN-3 fibrosarcoma cell lines made us possible to identify separate sets of genes responsible for motility/invasiveness and proliferation, respectively, in a single transcriptomic analysis *.

**Motility and invasiveness-related genes**	JUN3↑ JUN2f3↑ JUN2↓ and JUN3↓ JUN2f3↓ JUN2↑ (activators of motility) (suppressors of motility)
**Proliferation-related genes**	JUN3↑ JUN2f3↓ JUN2↓ and JUN3↓ JUN2f3↑ JUN2↑ (activators of proliferation) (suppressors of proliferation)

* Arrows indicate expression change of a hypothetical gene from the two biological groups in individual sarcoma cell lines.

## Data Availability

Not applicable.

## Supplementary Materials

The following are available online at https://www.mdpi.com/article/10.3390/jcm10112297/s1: Figure S1. Indirect immunofluorescence analysis—negative control (NC). Figure S2. Northern analysis of *c-fos* for JUN-2fos-X cell line clones. Figure S3. Cluster of dendrogram (transcriptomic analyses were performed in three biological replicates per cell line.). Table S1. Table of contrasts from comparisons of *jun* (a) and c-*fos* (b) expressions in different JUN-sarcoma cell lines. Table S2. Table of contrasts (post hoc tests) from comparison of cell area (a), cell perimeter (b), and cell roundness (c) of different lines of sarcoma cells. Table S3. Table of contrasts (post hoc tests) from comparison of proliferative capacity (a; measured as cell index), clonogenicity (b), sarcosphere formation (c), and invasiveness (d) of different lines of sarcoma cells. Table S4. Table of contrasts (post hoc tests) from comparison of invasiveness (a) and motility (b) of JUN-3 sarcoma cells treated with Bindarit (Bind), Maraviroc (Marav), or their combination (comb) compared to untreated control. Table S5. Table of contrasts (post hoc tests) from comparison of metabolic measures across different lines of sarcoma cells. Table S6. Genes with significantly changed expression in invasive sarcoma cell lines JUN-3 and JUN-2fos-3 compared to JUN-2 (available in xls.). Table S7. The gene set enrichment analysis (available in xls.). Table S8. Table of contrasts (post hoc tests) from comparison of invasiveness of JUN-3 sarcoma cells treated with Bindarit (Bind), Maraviroc (Marav), or their combination (comb) compared to untreated control. Table S9. Genes with significantly changed expression in JUN-2 compared to JUN-3 (available in xls.). Table S10. Genes with significantly changed expression in JUN-2fos3 compared to JUN-2 (available in xls.). Supplementary Tables of genes (Tables S6, S7, S9 and S10 in xls.)

## References

[1] Oda Y., Yamamoto H., Kohashi K., Yamada Y., Iura K., Ishii T., Maekawa A., Bekki H. (2017). Soft Tissue Sarcomas: From a Morphological to a Molecular Biological Approach. Pathol. Internat..

[2] Quesada J., Amato R. (2012). The Molecular Biology of Soft-Tissue Sarcomas and Current Trends in Therapy. Sarcoma.

[3] Sbaraglia M., Dei Tos A.P. (2019). The Pathology of Soft Tissue Sarcomas. Radiol. Med..

[4] Skubitz K.M., D’Adamo D.R. (2007). Sarcoma. Mayo Clin. Proc..

[5] Taylor B.S., Barretina J., Maki R.G., Antonescu C.R., Singer S., Ladanyi M. (2011). Advances in Sarcoma Genomics and New Therapeutic Targets. Nat. Rev. Cancer.

[6] Riedel R.F. (2012). Systemic Therapy for Advanced Soft Tissue Sarcomas. Cancer.

[7] Mertens F., Antonescu C.R., Mitelman F. (2016). Gene Fusions in Soft Tissue Tumors: Recurrent and Overlapping Pathogenetic Themes. Genes Chromosomes Cancer.

[8] Hatina J., Kripnerova M., Houfkova K., Pesta M., Kuncova J., Sana J., Slaby O., Rodríguez R. (2019). Sarcoma Stem Cell Heterogeneity. Adv. Exp. Med. Biol..

[9] Pennacchioli E., Tosti G., Barberis M., De Pas T.M., Verrecchia F., Menicanti C., Testori A., Mazzarol G. (2012). Sarcoma Spreads Primarily through the Vascular System: Are There Biomarkers Associated with Vascular Spread?. Clin. Exp. Metastasis.

[10] Tsukushi S., Nishida Y., Urakawa H., Kozawa E., Ishiguro N. (2014). Prognostic Significance of Histological Invasion in High Grade Soft Tissue Sarcomas. SpringerPlus.

[11] Lee A.T.J., Pollack S.M., Huang P., Jones R.L. (2017). Phase III Soft Tissue Sarcoma Trials: Success or Failure?. Curr. Treat. Options Oncol..

[12] Chibon F., Lagarde P., Salas S., Pérot G., Brouste V., Tirode F., Lucchesi C., de Reynies A., Kauffmann A., Bui B. (2010). Validated Prediction of Clinical Outcome in Sarcomas and Multiple Types of Cancer on the Basis of a Gene Expression Signature Related to Genome Complexity. Nat. Med..

[13] Jemaà M., Abdallah S., Lledo G., Perrot G., Lesluyes T., Teyssier C., Roux P., van Dijk J., Chibon F., Abrieu A. (2016). Heterogeneity in Sarcoma Cell Lines Reveals Enhanced Motility of Tetraploid versus Diploid Cells. Oncotarget.

[14] Chibon F., Lesluyes T., Valentin T., Guellec S.L. (2019). CINSARC Signature as a Prognostic Marker for Clinical Outcome in Sarcomas and Beyond. Genes Chromosomes Cancer.

[15] Cavanna T., Pokorna E., Vesely P., Gray C., Zicha D. (2007). Evidence for Protein 4.1B Acting as a Metastasis Suppressor. J. Cell Sci..

[16] Rosel D., Brabek J., Tolde O., Mierke C.T., Zitterbart D.P., Raupach C., Bicanova K., Kollmannsberger P., Pankova D., Vesely P. (2008). Up-Regulation of Rho/ROCK Signaling in Sarcoma Cells Drives Invasion and Increased Generation of Protrusive Forces. Mol. Cancer Res..

[17] Kainov Y., Favorskaya I., Delektorskaya V., Chemeris G., Komelkov A., Zhuravskaya A., Trukhanova L., Zueva E., Tavitian B., Dyakova N. (2014). CRABP1 Provides High Malignancy of Transformed Mesenchymal Cells and Contributes to the Pathogenesis of Mesenchymal and Neuroendocrine Tumors. Cell Cycle.

[18] Funes J.M., Quintero M., Henderson S., Martinez D., Qureshi U., Westwood C., Clements M.O., Bourboulia D., Pedley R.B., Moncada S. (2007). Transformation of Human Mesenchymal Stem Cells Increases Their Dependency on Oxidative Phosphorylation for Energy Production. Proc. Natl. Acad. Sci. USA.

[19] Ramanathan A., Wang C., Schreiber S.L. (2005). Perturbational Profiling of a Cell-Line Model of Tumorigenesis by Using Metabolic Measurements. Proc. Natl. Acad. Sci. USA.

[20] Hatina J., Hajkova L., Peychl J., Rudolf E., Finek J., Cervinka M., Reischig J. (2003). Establishment and Characterization of Clonal Cell Lines Derived from a Fibrosarcoma of the *H2-K/v-Jun* Transgenic Mouse. Tumor Biol..

[21] Schuh A.C., Keating S.J., Monteclaro F.S., Vogt P.K., Breitman M.L. (1990). Obligatory Wounding Requirement for Tumorigenesis in V- Jun Transgenic Mice. Nature.

[22] Katoh K., Takahashi Y., Hayashi S., Kondoh H. (1987). Improved Mammalian Vectors for High Expression of G418 Resistance. Cell Struct. Funct..

[23] Chomczynski P., Sacchi N. (2006). The Single-Step Method of RNA Isolation by Acid Guanidinium Thiocyanate-Phenol-Chloroform Extraction: Twenty-Something Years On. Nat. Protoc..

[24] Ausubel F.M., Brent R., Kingston R.E., Moore D.D., Seidman J.G., Smith J.A., Struhl K. (1995). Current Protocols in Molecular Biology.

[25] Maki Y., Bos T.J., Davis C., Starbuck M., Vogt P.K. (1987). Avian Sarcoma Virus 17 Carries the Jun Oncogene. Proc. Natl. Acad. Sci. USA.

[26] Holubova M., Leba M., Sedmikova M., Vannucci L., Horak V. (2012). Characterization of Three Newly Established Rat Sarcoma Cell Clones. In Vitro Cell Dev. Biol. Anim..

[27] Ke N., Wang X., Xu X., Abassi Y.A. (2011). The XCELLigence System for Real-Time and Label-Free Monitoring of Cell Viability. Methods Mol. Biol..

[28] Mori S., Chang J.T., Andrechek E.R., Matsumura N., Baba T., Yao G., Kim J.W., Gatza M., Murphy S., Nevins J.R. (2009). Anchorage-Independent Cell Growth Signature Identifies Tumors with Metastatic Potential. Oncogene.

[29] Fujii H., Honoki K., Tsujiuchi T., Kido A., Yoshitani K., Takakura Y. (2009). Sphere-Forming Stem-like Cell Populations with Drug Resistance in Human Sarcoma Cell Lines. Int. J. Oncol..

[30] Liu W.-D., Zhang T., Wang C.-L., Meng H.-M., Song Y.-W., Zhao Z., Li Z.-M., Liu J.-K., Pan S.-H., Wang W.-B. (2012). Sphere-Forming Tumor Cells Possess Stem-like Properties in Human Fibrosarcoma Primary Tumors and Cell Lines. Oncol. Lett..

[31] Boesch M., Reimer D., Rumpold H., Zeimet A.G., Sopper S., Wolf D. (2012). DyeCycle Violet Used for Side Population Detection Is a Substrate of P-Glycoprotein. Cytometry A.

[32] Quail D.F., Maciel T.J., Rogers K., Postovit L.M. (2012). A Unique 3D in Vitro Cellular Invasion Assay. J. Biomol. Screen..

[33] Santini M.T., Rainaldi G., Indovina P.L. (1999). Multicellular Tumour Spheroids in Radiation Biology. Int. J. Radiat. Biol..

[34] Pesta D., Gnaiger E. (2012). High-Resolution Respirometry: OXPHOS Protocols for Human Cells and Permeabilized Fibers from Small Biopsies of Human Muscle. Methods Mol. Biol..

[35] Kuznetsov A.V., Strobl D., Ruttmann E., Königsrainer A., Margreiter R., Gnaiger E. (2002). Evaluation of Mitochondrial Respiratory Function in Small Biopsies of Liver. Anal. Biochem..

[36] Larsen S., Nielsen J., Hansen C.N., Nielsen L.B., Wibrand F., Stride N., Schroder H.D., Boushel R., Helge J.W., Dela F. (2012). Biomarkers of Mitochondrial Content in Skeletal Muscle of Healthy Young Human Subjects. J. Physiol..

[37] Chen J.-R., Lazarenko O.P., Blackburn M.L., Rose S., Frye R.E., Badger T.M., Andres A., Shankar K. (2016). Maternal Obesity Programs Senescence Signaling and Glucose Metabolism in Osteo-Progenitors From Rat and Human. Endocrinology.

[38] Zhuang Y., Chan D.K., Haugrud A.B., Miskimins W.K. (2014). Mechanisms by Which Low Glucose Enhances the Cytotoxicity of Metformin to Cancer Cells Both in Vitro and in Vivo. PLoS ONE.

[39] Huber W., Carey V.J., Gentleman R., Anders S., Carlson M., Carvalho B.S., Bravo H.C., Davis S., Gatto L., Girke T. (2015). Orchestrating High-Throughput Genomic Analysis with Bioconductor. Nat. Methods.

[40] Mirolo M., Fabbri M., Sironi M., Vecchi A., Guglielmotti A., Mangano G., Biondi G., Locati M., Mantovani A. (2008). Impact of the Anti-Inflammatory Agent Bindarit on the Chemokinome: Selective Inhibition of the Monocyte Chemotactic Proteins. Eur. Cytokine Netw..

[41] Paccosi S., Giachi M., Di Gennaro P., Guglielmotti A., Parenti A. (2016). The Chemokine (C-C Motif) Ligand Protein Synthesis Inhibitor Bindarit Prevents Cytoskeletal Rearrangement and Contraction of Human Mesangial Cells. Cytokine.

[42] Halvorsen E.C., Hamilton M.J., Young A., Wadsworth B.J., LePard N.E., Lee H.N., Firmino N., Collier J.L., Bennewith K.L. (2016). Maraviroc Decreases CCL8-Mediated Migration of CCR5 ^+^ Regulatory T Cells and Reduces Metastatic Tumor Growth in the Lungs. OncoImmunology.

[43] Sicoli D., Jiao X., Ju X., Velasco-Velazquez M., Ertel A., Addya S., Li Z., Ando S., Fatatis A., Paudyal B. (2014). CCR5 Receptor Antagonists Block Metastasis to Bone of V-Src Oncogene-Transformed Metastatic Prostate Cancer Cell Lines. Cancer Res..

[44] Scrace S., O’Neill E., Hammond E.M., Pires I.M., Coutts A.S. (2013). Use of the xCELLigence System for Real-Time Analysis of Changes in Cellular Motility and Adhesion in Physiological Conditions. Adhesion Protein Protocols.

[45] R Core Team (2020)—European Environment Agency. https://www.eea.europa.eu/data-and-maps/indicators/oxygen-consuming-substances-in-rivers/r-development-core-team-2006.

[46] DiCiccio T.J., Efron B. (1996). Bootstrap Confidence Intervals. Statist. Sci..

[47] Benjamini Y., Hochberg Y. (1995). Controlling the False Discovery Rate: A Practical and Powerful Approach to Multiple Testing. J. R. Stat. Soc. Series B Stat. Methodol..

[48] Luo D., Koolaard S.G.J. Predictmeans: Calculate Predicted Means for Linear Models. https://cran.r-project.org/web/packages/predictmeans/predictmeans.pdf.

[49] Tichanek F., Salomova M., Jedlicka J., Kuncova J., Pitule P., Macanova T., Petrankova Z., Tuma Z., Cendelin J. (2020). Hippocampal Mitochondrial Dysfunction and Psychiatric-Relevant Behavioral Deficits in Spinocerebellar Ataxia 1 Mouse Model. Sci. Rep..

[50] Cendelin J., Tichanek F. (2020). Cerebellar Degeneration Averts Blindness-Induced Despaired Behavior during Spatial Task in Mice. Neurosci. Lett..

[51] Eklund A. Beeswarm: The Bee Swarm Plot, an Alternative to Stripchart. https://rdrr.io/cran/beeswarm/.

[52] Kelly T. TomKellyGenetics/Vioplot. https://cran.r-project.org/web/packages/vioplot/vioplot.pdf.

[53] Hatina J., Fernandes M.I., Hoffmann M.J., Zeimet A.G. (2013). Cancer Stem Cells—Basic Biological Properties and Experimental Approaches. eLS.

[54] Trucco M., Loeb D. (2012). Sarcoma Stem Cells: Do We Know What We Are Looking For?. Sarcoma.

[55] Colombo A., Basavarajaiah S., Limbruno U., Picchi A., Lettieri Valgimigli M., Sciahbasi A., Prati F., Calabresi M., Pierucci D., Guglielmotti A. (2015). A Double-Blind Randomised Study to Evaluate the Efficacy and Safety of Bindarit in Preventing Coronary Stent Restenosis. EuroIntervention.

[56] Parra J., Portilla J., Pulido F., Sánchez-de la Rosa R., Alonso-Villaverde C., Berenguer J., Blanco J.L., Domingo P., Dronda F., Galera C. (2011). Clinical Utility of Maraviroc. Clin. Drug Investig..

[57] Ozanne B.W., McGarry L., Spence H.J., Johnston I., Winnie J., Meagher L., Stapleton G. (2000). Transcriptional Regulation of Cell Invasion: AP-1 Regulation of a Multigenic Invasion Programme. Eur. J. Cancer.

[58] Ozanne B.W., Spence H.J., McGarry L.C., Hennigan R.F. (2007). Transcription Factors Control Invasion: AP-1 the First among Equals. Oncogene.

[59] Wang Z.-Q., Liang J., Schellander K., Wagner E.F., Grigoriadis A.E. (1995). C-Fos-Induced Osteosarcoma Formation in Transgenic Mice: Cooperativity with c-Jun and the Role of Endogenous c-Fos. Cancer Res..

[60] Mariani O., Brennetot C., Coindre J.-M., Gruel N., Ganem C., Delattre O., Stern M.-H., Aurias A. (2007). JUN Oncogene Amplification and Overexpression Block Adipocytic Differentiation in Highly Aggressive Sarcomas. Cancer Cell.

[61] Snyder E.L., Sandstrom D.J., Law K., Fiore C., Sicinska E., Brito J., Bailey D., Fletcher J.A., Loda M., Rodig S.J. (2009). C-Jun Amplification and Overexpression Are Oncogenic in Liposarcoma but Not Always Sufficient to Inhibit the Adipocytic Differentiation Programme. J. Pathol..

[62] Ivorra C., Kubicek M., González J.M., Sanz-González S.M., Alvarez-Barrientos A., O’Connor J.-E., Burke B., Andrés V. (2006). A Mechanism of AP-1 Suppression through Interaction of c-Fos with Lamin A/C. Genes Dev..

[63] Mohamood A.S., Gyles P., Balan K.V., Hollis V.W., Eckberg W.R., Asseffa A., Han Z., Wyche J.H., Anderson W.A. (1997). Estrogen Receptor, Growth Factor Receptor and Protooncogene Protein Activities and Possible Signal Transduction Crosstalk in Estrogen Dependent and Independent Breast Cancer Cell Lines. J. Submicrosc. Cytol. Pathol..

[64] La Vecchia S., Sebastián C. (2020). Metabolic Pathways Regulating Colorectal Cancer Initiation and Progression. Semin. Cell Dev. Biol..

[65] Caneba C.A., Bellance N., Yang L., Pabst L., Nagrath D. (2012). Pyruvate Uptake Is Increased in Highly Invasive Ovarian Cancer Cells under Anoikis Conditions for Anaplerosis, Mitochondrial Function, and Migration. Am. J. Physiol. Endocrinol. Metab..

[66] Wan J., Su Y., Song Q., Tung B., Oyinlade O., Liu S., Ying M., Ming G., Song H., Qian J. (2017). Methylated Cis-Regulatory Elements Mediate KLF4-Dependent Gene Transactivation and Cell Migration. eLife.

[67] Wang S., Shi X., Wei S., Ma D., Oyinlade O., Lv S.-Q., Ying M., Zhang Y.A., Claypool S.M., Watkins P. (2018). Krüppel-like Factor 4 (KLF4) Induces Mitochondrial Fusion and Increases Spare Respiratory Capacity of Human Glioblastoma Cells. J. Biol. Chem..

[68] Marchetti P., Fovez Q., Germain N., Khamari R., Kluza J. (2020). Mitochondrial Spare Respiratory Capacity: Mechanisms, Regulation, and Significance in Non-Transformed and Cancer Cells. FASEB J..

[69] Lee Y.-K., Jee B.A., Kwon S.M., Yoon Y.-S., Xu W.G., Wang H.-J., Wang X.W., Thorgeirsson S.S., Lee J.-S., Woo H.G. (2015). Identification of a Mitochondrial Defect Gene Signature Reveals NUPR1 as a Key Regulator of Liver Cancer Progression. Hepatology.

[70] Machida K. (2018). Pluripotency Transcription Factors and Metabolic Reprogramming of Mitochondria in Tumor-Initiating Stem-like Cells. Antioxid. Redox Signal..

[71] Basu-Roy U., Bayin N.S., Rattanakorn K., Han E., Placantonakis D.G., Mansukhani A., Basilico C. (2015). Sox2 Antagonizes the Hippo Pathway to Maintain Stemness in Cancer Cells. Nat. Commun..

[72] Maurizi G., Verma N., Gadi A., Mansukhani A., Basilico C. (2018). Sox2 Is Required for Tumor Development and Cancer Cell Proliferation in Osteosarcoma. Oncogene.

[73] Yamaguchi H., Taouk G.M. (2020). A Potential Role of YAP/TAZ in the Interplay Between Metastasis and Metabolic Alterations. Front. Oncol..

[74] Würl P., Kappler M., Meye A., Bartel F., Köhler T., Lautenschläger C., Bache M., Schmidt H., Taubert H. (2002). Co-Expression of Survivin and TERT and Risk of Tumour-Related Death in Patients with Soft-Tissue Sarcoma. Lancet.

[75] Nikitovic D., Kouvidi K., Karamanos N.K., Tzanakakis G.N. (2013). The Roles of Hyaluronan/RHAMM/CD44 and Their Respective Interactions along the Insidious Pathways of Fibrosarcoma Progression. BioMed Res. Int..

[76] Farmaki E., Chatzistamou I., Kaza V., Kiaris H. (2016). A CCL8 Gradient Drives Breast Cancer Cell Dissemination. Oncogene.

[77] Barbai T., Fejős Z., Puskas L.G., Tímár J., Rásó E. (2015). The Importance of Microenvironment: The Role of CCL8 in Metastasis Formation of Melanoma. Oncotarget.

[78] Otsubo C., Otomo R., Miyazaki M., Matsushima-Hibiya Y., Kohno T., Iwakawa R., Takeshita F., Okayama H., Ichikawa H., Saya H. (2014). TSPAN2 Is Involved in Cell Invasion and Motility during Lung Cancer Progression. Cell Rep..

[79] Fils-Aimé N., Dai M., Guo J., El-Mousawi M., Kahramangil B., Neel J.-C., Lebrun J.-J. (2013). MicroRNA-584 and the Protein Phosphatase and Actin Regulator 1 (PHACTR1), a New Signaling Route through Which Transforming Growth Factor-β Mediates the Migration and Actin Dynamics of Breast Cancer Cells. J. Biol. Chem..

[80] Bagci T., Wu J.K., Pfannl R., Ilag L.L., Jay D.G. (2009). Autocrine Semaphorin 3A Signaling Promotes Glioblastoma Dispersal. Oncogene.

[81] Tao J., Cong H., Wang H., Zhang D., Liu C., Chu H., Qing Q., Wang K. (2018). MiR-30a-5p Inhibits Osteosarcoma Cell Proliferation and Migration by Targeting FOXD1. Biochem. Biophys. Res. Commun..

[82] Li D., Fan S., Yu F., Zhu X., Song Y., Ye M., Fan L., Lv Z. (2018). FOXD1 Promotes Cell Growth and Metastasis by Activation of Vimentin in NSCLC. Cell Physiol. Biochem..

[83] Wu H., Larribère L., Sun Q., Novak D., Sachindra S., Granados K., Umansky V., Utikal J. (2018). Loss of Neural Crest-Associated Gene FOXD1 Impairs Melanoma Invasion and Migration via RAC1B Downregulation. Int. J. Cancer.

[84] Ondondo B., Colbeck E., Jones E., Smart K., Lauder S.N., Hindley J., Godkin A., Moser B., Ager A., Gallimore A. (2015). A Distinct Chemokine Axis Does Not Account for Enrichment of Foxp3(+) CD4(+) T Cells in Carcinogen-Induced Fibrosarcomas. Immunology.

[85] Gazzaniga S., Bravo A.I., Guglielmotti A., van Rooijen N., Maschi F., Vecchi A., Mantovani A., Mordoh J., Wainstok R. (2007). Targeting Tumor-Associated Macrophages and Inhibition of MCP-1 Reduce Angiogenesis and Tumor Growth in a Human Melanoma Xenograft. J. Investig. Dermatol..

[86] Liu S., Gao H., Gao C., Liu W., Xing D. (2018). Bindarit Attenuates Pain and Cancer-Related Inflammation by Influencing Myeloid Cells in a Model of Bone Cancer. Arch. Immunol. Ther. Exp..

[87] Ward S.T., Li K.K., Hepburn E., Weston C.J., Curbishley S.M., Reynolds G.M., Hejmadi R.K., Bicknell R., Eksteen B., Ismail T. (2015). The Effects of CCR5 Inhibition on Regulatory T-Cell Recruitment to Colorectal Cancer. Br. J. Cancer..

[88] Tanabe Y., Sasaki S., Mukaida N., Baba T. (2016). Blockade of the Chemokine Receptor, CCR5, Reduces the Growth of Orthotopically Injected Colon Cancer Cells via Limiting Cancer-Associated Fibroblast Accumulation. Oncotarget.

[89] Zollo M., Di Dato V., Spano D., De Martino D., Liguori L., Marino N., Vastolo V., Navas L., Garrone B., Mangano G. (2012). Targeting Monocyte Chemotactic Protein-1 Synthesis with Bindarit Induces Tumor Regression in Prostate and Breast Cancer Animal Models. Clin. Exp. Metastasis.

[90] Singh S.K., Mishra M.K., Eltoum I.-E.A., Bae S., Lillard J.W., Singh R. (2018). CCR5/CCL5 Axis Interaction Promotes Migratory and Invasiveness of Pancreatic Cancer Cells. Sci. Rep..

[91] Pervaiz A., Zepp M., Mahmood S., Ali D.M., Berger M.R., Adwan H. (2019). CCR5 Blockage by Maraviroc: A Potential Therapeutic Option for Metastatic Breast Cancer. Cell. Oncol..

[92] Yang J., Sontag D., Gong Y., Minuk G.Y. (2021). Alterations in Chemokine Receptor CCR5 Activity Influence Tumor Cell Biology in Human Cholangiocarcinoma Cell Lines. Ann. Hepatol..

[93] Maione F., Molla F., Meda C., Latini R., Zentilin L., Giacca M., Seano G., Serini G., Bussolino F., Giraudo E. (2009). Semaphorin 3A Is an Endogenous Angiogenesis Inhibitor That Blocks Tumor Growth and Normalizes Tumor Vasculature in Transgenic Mouse Models. J. Clin. Investig..

[94] Hu B., Cheng S.-Y. (2009). Angiopoietin-2: Development of Inhibitors for Cancer Therapy. Curr. Oncol. Rep..

[95] Marconcini L., Marchio S., Morbidelli L., Cartocci E., Albini A., Ziche M., Bussolino F., Oliviero S. (1999). C-Fos-Induced Growth Factor/Vascular Endothelial Growth Factor D Induces Angiogenesis in Vivo and in Vitro. Proc. Natl. Acad. Sci. USA.

[96] Yanagawa T., Shinozaki T., Watanabe H., Saito K., Raz A., Takagishi K. (2012). Vascular Endothelial Growth Factor-D Is a Key Molecule That Enhances Lymphatic Metastasis of Soft Tissue Sarcomas. Exp. Cell Res..

[97] Kilvaer T.K., Valkov A., Sorbye S., Smeland E., Bremnes R.M., Busund L.-T., Donnem T. (2010). Profiling of VEGFs and VEGFRs as Prognostic Factors in Soft Tissue Sarcoma: VEGFR-3 Is an Independent Predictor of Poor Prognosis. PLoS ONE.

[98] Zhao T., Zhao W., Meng W., Liu C., Chen Y., Bhattacharya S.K., Sun Y. (2016). Vascular Endothelial Growth Factor-D Mediates Fibrogenic Response in Myofibroblasts. Mol. Cell Biochem..

[99] Wang P., Chen S.-H., Hung W.-C., Paul C., Zhu F., Guan P.-P., Huso D.L., Kontrogianni-Konstantopoulos A., Konstantopoulos K. (2015). Fluid Shear Promotes Chondrosarcoma Cell Invasion by Activating Matrix Metalloproteinase 12 via IGF-2 and VEGF Signaling Pathways. Oncogene.

[100] Siemann N.M., Siemann D.W. Angiopoietin-2 Axis Inhibitors: Current Status and Future Considerations for Cancer Therapy. https://www.eurekaselect.com/115040/article.

[101] Bezuidenhout L., Zilla P., Davies N. (2009). Association of Ang-2 with Integrin Beta 2 Controls Ang-2/PDGF-BB-Dependent Upregulation of Human Peripheral Blood Monocyte Fibrinolysis. Inflammation.

[102] Hu B., Jarzynka M.J., Guo P., Imanishi Y., Schlaepfer D.D., Cheng S.-Y. (2006). Angiopoietin 2 Induces Glioma Cell Invasion by Stimulating Matrix Metalloprotease 2 Expression through the Alphavbeta1 Integrin and Focal Adhesion Kinase Signaling Pathway. Cancer Res..

[103] Morii T., Mochizuki K., Tajima T., Ichimura S., Satomi K. (2011). D-Dimer Levels as a Prognostic Factor for Determining Oncological Outcomes in Musculoskeletal Sarcoma. BMC Musculoskelet. Disord..

[104] Raj S.D., Zhou X., Bueso-Ramos C.E., Ravi V., Patel S., Benjamin R.S., Vadhan-Raj S. (2012). Prognostic Significance of Elevated D-Dimer for Survival in Patients with Sarcoma. Am. J. Clin. Oncol..

[105] Bure I.V., Kuznetsova E.B., Zaletaev D.V. (2018). Long Noncoding RNAs and Their Role in Oncogenesis. Mol. Biol..

[106] Zhang R., Xia T. (2017). Long Non-Coding RNA XIST Regulates PDCD4 Expression by Interacting with MiR-21-5p and Inhibits Osteosarcoma Cell Growth and Metastasis. Int. J. Oncol..

[107] Lv G.-Y., Miao J., Zhang X.-L. (2018). Long Noncoding RNA XIST Promotes Osteosarcoma Progression by Targeting Ras-Related Protein RAP2B via MiR-320b. Oncol. Res..

[108] Yildirim E., Kirby J.E., Brown D.E., Mercier F.E., Sadreyev R.I., Scadden D.T., Lee J.T. (2013). Xist RNA Is a Potent Suppressor of Hematologic Cancer in Mice. Cell.

[109] Navarro P., Chambers I., Karwacki-Neisius V., Chureau C., Morey C., Rougeulle C., Avner P. (2008). Molecular Coupling of Xist Regulation and Pluripotency. Science.

[110] Koga M., Matsuda M., Kawamura T., Sogo T., Shigeno A., Nishida E., Ebisuya M. (2014). Foxd1 Is a Mediator and Indicator of the Cell Reprogramming Process. Nat. Commun..

[111] Gandalovičová A., Rosel D., Fernandes M., Veselý P., Heneberg P., Čermák V., Petruželka L., Kumar S., Sanz-Moreno V., Brábek J. (2017). Migrastatics-Anti-Metastatic and Anti-Invasion Drugs: Promises and Challenges. Trends Cancer.

[112] Rosel D., Fernandes M., Sanz-Moreno V., Brábek J. (2019). Migrastatics: Redirecting R&D in Solid Cancer Towards Metastasis?. Trends Cancer.

[113] Kikuchi K., Kishino A., Konishi O., Kumagai K., Hosotani N., Saji I., Nakayama C., Kimura T. (2003). In Vitro and in Vivo Characterization of a Novel Semaphorin 3A Inhibitor, SM-216289 or Xanthofulvin. J. Biol. Chem..

[114] Martínez-García D., Manero-Rupérez N., Quesada R., Korrodi-Gregório L., Soto-Cerrato V. (2019). Therapeutic Strategies Involving Survivin Inhibition in Cancer. Med. Res. Rev..

