# Supplementary material for: Complex Interplay of Genes Underlies Invasiveness in Fibrosarcoma Progression Model
Source: J Clin Med. 2021 May 25;10(11):2297. doi: 10.3390/jcm10112297 (PMC8197499; doi:10.3390/jcm10112297)
Supplement: Supplementary file 1 [file jcm-10-02297-s001.zip › Supplementary Materials.pdf]

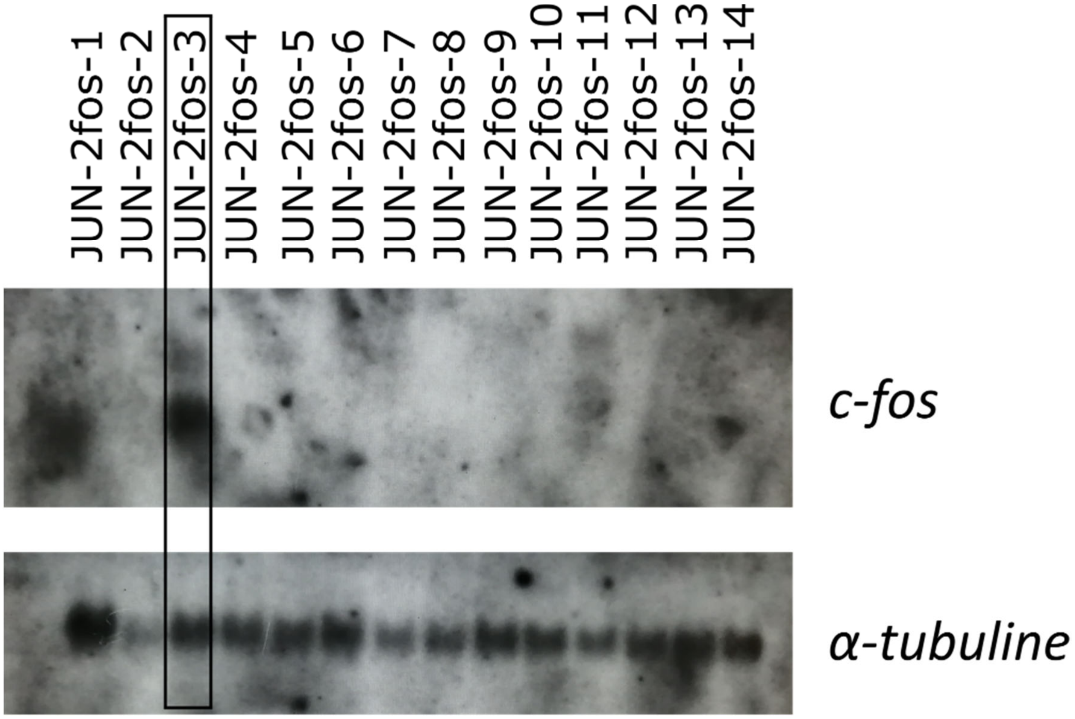

Figure S1 Northern analysis of *c-fos* for JUN-2fos-x cell line clones
Northern analysis of *c-fos* gene expression after transfection of the CMV-c-fos expression vector. A single clone expressing the highest level of the *c-fos* transcript (clone 3) was designated JUN-2fos-3 and used for all other analyses.

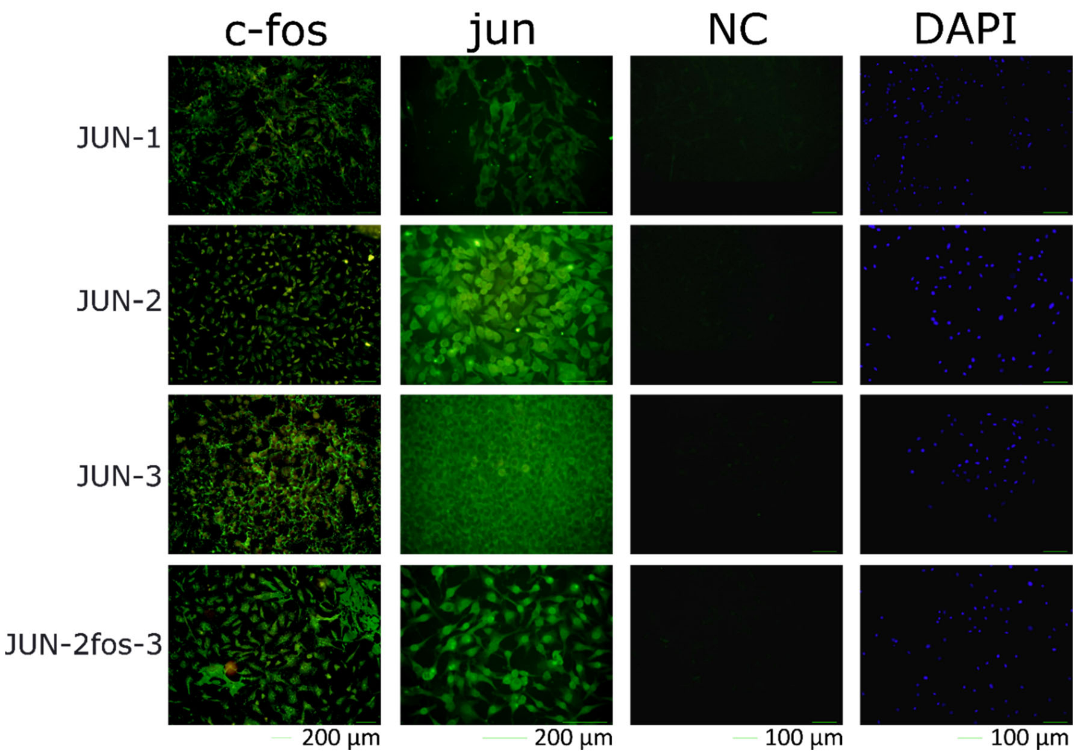

Figure S2 Indirect immunofluorescence analysis - Fos and Jun oncoproteins expression and negative control (NC) staining, which has been carried out by omitting the respective primary antibody and the same field has been stained with DAPI staining. Pictures were taken by Olympus IX70 fluorescent microscope equipped with the Olympus DP71 camera system.

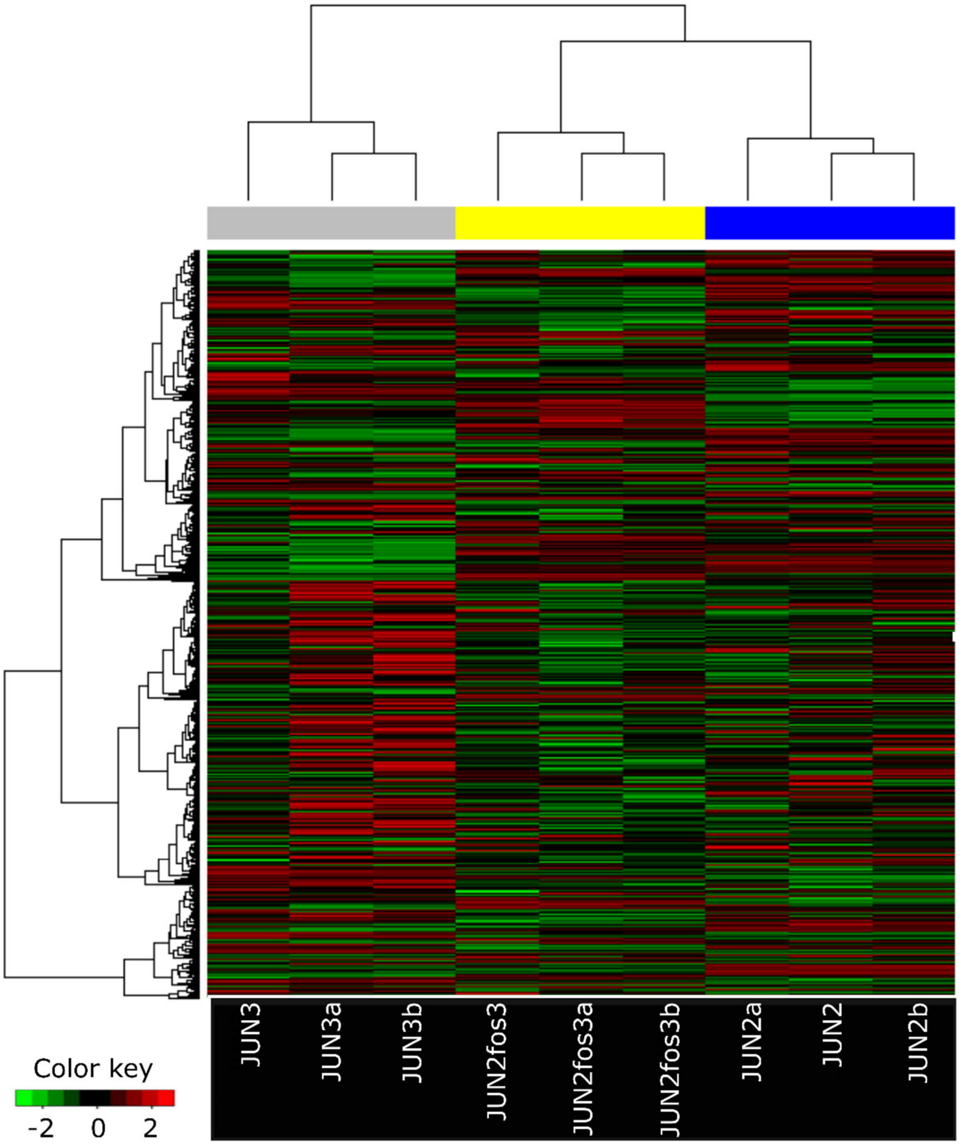

Figure S3 Cluster dendrogram of biological replicates of sarcoma cell lines used for the transcriptomic analysis. Clustering has been carried out based on the full data matrix of expression data consisting of 22690 genes, whereas the heatmap relies on expression data of randomly selected 2000 genes.

Table S1. Table of contrasts from comparisons of *jun* (a) and c-*fos* (b) expressions in different JUN sarcoma cell lines. Beta= effect size – the difference between cell lines with respect to the log2-transformed relative expression of jun (a) or *fos* (b). CI_L and CI_U= limits of 95% confidence intervals for the beta, based on Bias-Corrected and Accelerated (BCa) bootstrap simulations. p= raw p-value from permutational t-test. fdr_p= p-value corrected by False Discovery Rate correction for multiple comparisons.

(a) *Jun* expression	Beta	CI_L	CI_U	p	fdr_p
JUN1 vs. JUN2	-1.41	-1.45	-1.37	0.0022	0.0026
JUN1 vs. JUN2fos3	-1.60	-1.64	-1.56	0.0023	0.0026
JUN1 vs. JUN3	-0.63	-0.69	-0.59	0.0021	0.0026
JUN2 vs. JUN2fos3	-0.19	-0.25	-0.14	0.0020	0.0026
JUN2 vs. JUN3	0.78	0.71	0.83	0.0019	0.0026
JUN2fos3 vs. JUN3	0.97	0.90	1.02	0.0026	0.0026
(b) *Fos* expression	Beta	CI_L	CI_U	p	fdr_p
JUN1 vs. JUN2	-0.68	-0.73	-0.64	0.0026	0.0028
JUN1 vs. JUN2fos3	-1.73	-1.76	-1.70	0.0020	0.0028
JUN1 vs. JUN3	-0.35	-0.39	-0.31	0.0028	0.0028
JUN2 vs. JUN2fos3	-1.05	-1.09	-1.01	0.0021	0.0028
JUN2 vs. JUN3	0.33	0.29	0.37	0.0024	0.0028
JUN2fos3 vs. JUN3	1.38	1.35	1.41	0.0027	0.0028

Table S2. Table of contrasts (post-hoc tests) from comparison of cell area (a), cell perimeter (b) and cell roundness (c) of different lines of sarcoma cells. Beta= effect size – the difference between cell lines with respect to given evaluated measure. CI_L and CI_U= limits of the 95% confidence intervals for the beta, based on Bias-Corrected and Accelerated (BCa) bootstrap simulations. p= raw p-value from permutational t-test. fdr_p= p-value corrected by False Discovery Rate correction for multiple comparison.

(a) Cell area	Beta	CI_L	CI_U	p	fdr_p
JUN1 vs. JUN2	0.56	0.32	0.79	<0.001	<0.001
JUN1 vs. JUN2fos3	-0.59	-0.77	-0.39	<0.001	<0.001
JUN1 vs. JUN3	1.40	1.18	1.62	<0.001	<0.001
JUN2 vs. JUN2fos3	-1.15	-1.32	-0.960	<0.001	<0.001
JUN2 vs. JUN3	0.84	0.64	1.05	<0.001	<0.001
JUN2fos3 vs. JUN3	1.999	1.83	2.15	<0.001	<0.001
(b) Cell perimeter	Beta	CI_L	CI_U	p	fdr_p
JUN1 vs. JUN2	0.77	0.60	0.91	<0.001	<0.001
JUN1 vs. JUN2fos3	-0.13	-0.27	-0.002	0.35	0.35
JUN1 vs. JUN3	1.04	0.907	1.20	<0.001	0.0013
JUN2 vs. JUN2fos3	-0.90	-1.05	-0.73	<0.001	<0.001
JUN2 vs. JUN3	0.27	0.11	0.45	0.01	0.012
JUN2fos3 vs. JUN3	1.17	1.02	1.33	<0.001	<0.001
(c) Cell roundness	Beta	CI_L	CI_U	p	fdr_p
JUN1 vs. JUN2	-0.15	-0.21	-0.09	0.011	0.033
JUN1 vs. JUN2fos3	-0.04	-0.08	0.004	0.648	0.648
JUN1 vs. JUN3	-0.10	-0.17	-0.04	0.162	0.325
JUN2 vs. JUN2fos3	0.11	0.05	0.16	0.002	0.014
JUN2 vs. JUN3	0.05	-0.04	0.12	0.632	0.648
JUN2fos3 vs. JUN3	-0.06	-0.13	-0.002	0.221	0.331

Table S3. Table of contrasts (post-hoc tests) from comparison of proliferative capacity (a; measured as cell index), clonogenicity (b) and sarcosphere formation (c) of different lines of sarcoma cells. *Beta*= effect size – the difference between cell lines with respect to given evaluated measure. *CI_L* and *CI_U*= limits of 95% confidence intervals for the *beta*, based on Bias-Corrected and Accelerated (BCa) bootstrap simulations. *p*= raw p-value from permutational t-test. *fdr_p*= p-value corrected by False Discovery Rate correction for multiple comparison.

(a) Cell index	Beta	CI_L	CI_U	p	fdr_p
JUN1 vs. JUN2	0.13	0.03	0.26	0.03177	0.03177
JUN1 vs. JUN2fos3	0.44	0.32	0.56	<0.001	<0.001
JUN1 vs. JUN3	-0.96	-1.40	-0.48	<0.001	<0.001
JUN2 vs. JUN2fos3	0.31	0.16	0.45	<0.001	<0.001
JUN2 vs. JUN3	-1.10	-1.55	-0.61	<0.001	<0.001
JUN2fos3 vs. JUN3	-1.41	-1.86	-0.92	<0.001	<0.001
(c) Clonogenicity	Beta	CI_L	CI_U	p	fdr_p
JUN1 vs. JUN2	0.97	0.13	1.87	0.03790	0.03790
JUN1 vs. JUN2fos3	7.36	6.28	8.50	<0.001	<0.001
JUN1 vs. JUN3	-10.92	-12.31	-9.56	<0.001	<0.001
JUN2 vs. JUN2fos3	6.40	5.18	7.60	<0.001	<0.001
JUN2 vs. JUN3	-11.89	-13.40	-10.41	<0.001	<0.001
JUN2fos3 vs. JUN3	-18.28	-19.92	-16.69	<0.001	<0.001
(d) Sarcospheres	Beta	CI_L	CI_U	p	fdr_p
JUN1 vs. JUN2	-0.06	-0.37	0.28	0.7208	0.7208
JUN1 vs. JUN2fos3	-0.46	-0.88	-0.01	0.0671	0.0805
JUN1 vs. JUN3	-1.28	-1.75	-0.78	0.0022	0.0065
JUN2 vs. JUN2fos3	-0.40	-0.78	-0.07	0.0368	0.0552
JUN2 vs. JUN3	-1.22	-1.62	-0.80	0.0022	0.0065
JUN2fos3 vs. JUN3	-0.82	-1.31	-0.29	0.0130	0.0260

Table S4. Table of contrasts (post-hoc tests) from comparison of invasiveness (a) and motility (b) of JUN-3 sarcoma cells treated with Bindarit (Bind), Maraviroc (Marav) or their combination (Comb) compared to untreated control. Beta= effect size – the difference between cell lines with respect to given evaluated measure. CI_L and CI_U= limits of 95% confidence intervals for the beta, based on Bias-Corrected and Accelerated (BCa) bootstrap simulations. p= raw p-value from permutational t-test. fdr_p= p-value corrected by False Discovery Rate correction for multiple comparison. See methods for definition of the measures states and capacities.

(b) Invasiveness	Beta	CI_L	CI_U	p	fdr_p
JUN1 vs. JUN2	1.76	1.57	1.94	<0.001	<0.001
JUN1 vs. JUN2fos3	-0.40	-0.58	-0.23	0.0029	0.0029
JUN1 vs. JUN3	-2.38	-2.55	-2.21	<0.001	<0.001
JUN2 vs. JUN2fos3	-2.17	-2.32	-2.01	<0.001	<0.001
JUN2 vs. JUN3	-4.14	-4.28	-3.99	<0.001	<0.001
JUN2fos3 vs. JUN3	-1.97	-2.11	-1.84	<0.001	<0.001

Table S5. Table of contrasts (post-hoc tests) from comparison of metabolic measures across different lines of sarcoma cells. The metabolic measures include mitochondrial oxygen consumption in several states of high-resolution respirometry, including ROUT (a), ETS (b) and LEAK (c), spare respiratory (E-R) capacity (d), L-lactate production (e) and consumption of glucose (f). Beta= effect size – the difference between cell lines with respect to given evaluated measure. CI_L and CI_U= limits of 95% confidence intervals for the beta, based on Bias-Corrected and Accelerated (BCa) bootstrap simulations. p= raw p-value from permutational t-test. fdr_p= p-value corrected by False Discovery Rate correction for multiple comparison. See methods for definition of the measures states and capacities.

(a) ROUT	Beta	CI_L	CI_U	p	fdr_p
JUN1 vs. JUN2	389	128	685	0.0096	0.0193
JUN1 vs. JUN2fos3	-17	-287	296	0.8984	0.8984
JUN1 vs. jun3	40	-260	368	0.8014	0.8984
JUN2 vs. JUN2fos3	-406	-587	-229	<0.001	0.0030
JUN2 vs. jun3	-349	-569	-155	0.0025	0.0074
JUN2fos3 vs. jun3	57	-179	272	0.6247	0.8984
(b) ETS	Beta	CI_L	CI_U	p	fdr_p
JUN1 vs. JUN2	30	-599	602	0.924	0.924
JUN1 vs. JUN2fos3	-869	-1455	-337	0.007	0.015
JUN1 vs. jun3	-946	-1635	-327	0.012	0.018
JUN2 vs. JUN2fos3	-899	-1404	-429	0.003	0.015
JUN2 vs. jun3	-976	-1596	-398	0.006	0.015
JUN2fos3 vs. jun3	-77	-654	468	0.793	0.924
(c) LEAK	Beta	CI_L	CI_U	p	fdr_p
JUN1 vs. JUN2	224	82	402	0.0047	0.0140
JUN1 vs. JUN2fos3	51	-88	231	0.5883	0.5883
JUN1 vs. jun3	89	-88	291	0.3684	0.5526
JUN2 vs. JUN2fos3	-173	-227	-116	<0.001	0.0010
JUN2 vs. jun3	-135	-262	-27	0.0411	0.0822
JUN2fos3 vs. jun3	38	-86	145	0.5270	0.5883
(d) E-R	Beta	CI_L	CI_U	p	fdr_p
JUN1 vs. JUN2	-359	-811	84	0.1305	0.1566
JUN1 vs. JUN2fos3	-852	-1242	-458	<0.001	0.0025
JUN1 vs. jun3	-987	-1477	-530	<0.001	0.0025
JUN2 vs. JUN2fos3	-493	-812	-174	0.0093	0.0145
JUN2 vs. jun3	-628	-1052	-235	0.0097	0.0145
JUN2fos3 vs. jun3	-135	-504	207	0.4743	0.4743
(e) L-Lactate	Beta	CI_L	CI_U	p	fdr_p
JUN1 vs. JUN2	1.21	0.75	1.63	0.0022	0.0043
JUN1 vs. JUN2fos3	-0.91	-1.50	-0.42	0.0040	0.0061
JUN1 vs. JUN3	-0.74	-1.59	-0.04	0.1082	0.1298
JUN2 vs. JUN2fos3	-2.12	-2.51	-1.81	0.0006	0.0035
JUN2 vs. JUN3	-1.95	-2.72	-1.34	0.0022	0.0043
JUN2fos3 vs. JUN3	0.18	-0.61	0.85	0.6301	0.6301
(f) glucose	Beta	CI_L	CI_U	p	fdr_p
JUN1 vs. JUN2	-0.75	-1.18	-0.28	0.0043	0.0130
JUN1 vs. JUN2fos3	-1.03	-1.63	-0.04	0.0117	0.0175
JUN1 vs. JUN3	-1.36	-1.93	-0.82	0.0043	0.0130
JUN2 vs. JUN2fos3	-0.28	-2.79	0.23	0.3368	0.3368

JUN2 vs. JUN3	-0.61	-1.10	-0.21	0.0087	0.0173
JUN2fos3 vs. JUN3	-0.34	-0.96	0.25	0.3157	0.3368

Table S6. Genes with significantly changed expression in invasive sarcoma cell lines JUN-3 and JUN-2fos-3 compared to JUN-2

No.	Gene.title	Gene.Symbol	2^logFC	adj.p.val	Chromosome
1	Lymphocyte antigen 6 complex, locus A	*Ly6a*	11.10	0.000652	15
2	Forkhead box D1	*Foxd1*	9.67	0.000083	13
3	Tetraspanin 2	*Tspan2*	9.11	0.000150	3
4	Myosin VB	*Myo5b*	8.26	0.000159	18
5	SRY (sex determining region Y)-box 2	*Sox2*	7.78	0.000001	3
6	Teneurin transmembrane protein 3	*Tenm3*	7.52	0.000435	8
7	CD244 natural killer cell receptor 2B4	*Cd244*	6.07	0.000229	1
8	Sema domain, immunoglobulin domain (Ig), short basic domain, secreted, (semaphorin) 3A	*Sema3a*	6.00	0.000097	5
9	C-fos induced growth factor	*Figf*	5.85	0.000138	X
10	Protein kinase, cgmp-dependent, type II	*Prkg2*	5.82	0.000579	5
11	Keratin 13	*Krt13*	5.70	0.000071	11
12	Matrilin 2	*Matn2*	5.32	0.000026	15
13	Collagen, type VI, alpha 3	*Col6a3*	5.22	0.000001	1
14	Carbohydrate sulfotransferase 11 /// phosphatase and actin regulator 1	*Chst11 /// Phactr1*	4.80	0.000010	10///13
15	Max dimerization protein 3	*Mxd3*	4.78	0.000919	13
16	Spindle and kinetochore associated complex subunit 1	*Ska1*	4.74	0.000018	18
17	Calponin 2	*Cnn2*	4.60	0.000003	10
18	Arylsulfatase i	*Arsi*	4.41	0.000297	18
19	Chemokine (C-C motif) ligand 8	*Ccl8*	4.34	0.000003	11
20	CD244 natural killer cell receptor 2B4 /// natural killer cell receptor 2B4-like	*Cd244 /// LOC677008*	3.96	0.000903	1///???
21	GLI pathogenesis-related 1 (glioma)	*Glipr1*	3.96	0.000194	10
22	Star-related lipid transfer (START) domain containing 4	*Stard4*	3.94	0.000158	18
23	Methyltransferase like 7A1	*Mettl7a1*	3.84	0.000114	15
24	Rho gtpase activating protein 18	*Arhgap18*	3.67	0.000021	10
25	Cytochrome b-561	*Cyb561*	3.65	0.000071	11
26	Protein kinase C, delta binding protein	*Prkcdbp*	3.63	0.000122	7
27	Ras homolog gene family, member J	*Rhoj*	3.52	0.000136	12
28	Reproductive homeobox 5	*Rhox5*	3.50	0.000006	X
29	Chromodomain helicase DNA binding protein 3, opposite strand	*Chd3os*	3.47	0.000138	11
30	WW, C2 and coiled-coil domain containing 1	*Wwc1*	3.43	0.000248	11
31	MACRO domain containing 2	*Macrod2*	3.42	0.000161	2
32	Dynamin 3, opposite strand /// microrna 214	*Dnm3os /// Mir214*	3.38	0.000215	1///1
33	CD109 antigen	*Cd109*	3.34	0.000071	9
34	Family with sequence similarity 13, member C	*Fam13c*	3.33	0.000572	10
35	PDZ and LIM domain 2	*Pdlim2*	3.25	0.000043	14
36	Dynamin 1	*Dnm1*	3.22	0.000216	2
37	Testis derived transcript	*Tes*	3.22	0.000015	6
38	Progressive ankylosis	*Ank*	3.18	0.000006	15

39	Vanin 1	*Vnn1*	3.18	0.000882	10
40	Fyn-related kinase	*Frk*	3.13	0.000470	10
41	Fibrinogen-like protein 2	*Fgl2*	3.07	0.000249	5
42	Death-associated protein	*Dap*	2.96	0.000171	15
43	Phosphatase and actin regulator 1	*Phactr1*	2.96	0.000340	13
44	Kinesin family member 20A	*Kif20a*	2.92	0.000132	18
45	RAB32, member RAS oncogene family	*Rab32*	2.92	0.000061	10
46	Doublecortin-like kinase 1	*Dclk1*	2.91	0.000860	3
47	Angiopoietin 2	*Angpt2*	2.89	0.000005	8
48	KH domain containing, RNA binding, signal transduction associated 3	*Khdrbs3*	2.88	0.000484	15
49	Phosphofructokinase, platelet	*Pfkp*	2.86	0.000003	13
50	Methyltransferase like 7A2	*Mettl7a2*	2.76	0.000235	15
51	Leucine-rich repeat kinase 2	*Lrrk2*	2.76	0.000123	15
52	CD80 antigen	*Cd80*	2.76	0.000152	16
53	Aldehyde dehydrogenase 1 family, member L1	*Aldh1l1*	2.73	0.000014	6
54	Cyclin-dependent kinase inhibitor 1A (P21)	*Cdkn1a*	2.67	0.000296	17
55	DNA replication helicase 2 homolog (yeast)	*Dna2*	2.61	0.000772	10
56	Ectopic ossification 1	*Etos1*	2.59	0.000174	7
57	Ring finger protein 130	*Rnf130*	2.58	0.000084	11
58	Dual specificity phosphatase 6	*Dusp6*	2.55	0.000129	10
59	Sulfatase 2	*Sulf2*	2.55	0.000782	2
60	NAD(P) dependent steroid dehydrogenase-like	*Nsdhl*	2.53	0.000015	X
61	Hexokinase 1	*Hk1*	2.52	0.000330	10
62	Complement component 3a receptor 1	*C3ar1*	2.39	0.000782	6
63	Tripartite motif-containing 24	*Trim24*	2.38	0.000111	6
64	Integrin beta 2	*Itgb2*	2.31	0.000378	10
65	Thrombospondin 2	*Thbs2*	2.29	0.000168	17
66	Family with sequence similarity 19, member A5	*Fam19a5*	2.28	0.000252	15
67	Pleckstrin homology-like domain, family A, member 1	*Phlda1*	2.27	0.000824	10
68	Ral guanine nucleotide dissociation stimulator,-like 1	*Rgl1*	2.27	0.000867	1
69	Camp responsive element binding protein 3-like 2	*Creb3l2*	2.26	0.000304	6
70	Hematological and neurological expressed sequence 1	*Hn1*	2.25	0.000229	11
71	Epithelial membrane protein 3	*Emp3*	2.25	0.000269	7
72	Non-SMC condensin II complex, subunit H2	*Ncaph2*	2.24	0.000657	15
73	Sorcin	*Sri*	2.23	0.000118	5
74	Baculoviral IAP repeat-containing 5	*Birc5*	2.23	0.000929	11
75	Inositol (myo)-1(or 4)-monophosphatase 2	*Impa2*	2.19	0.000702	18
76	Zinc finger homeodomain 4	*Zfhx4*	2.18	0.000936	3
77	Apoptotic peptidase activating factor 1	*Apaf1*	2.18	0.000968	10
78	Vps20-associated 1 homolog (S. Cerevisiae)	*Vta1*	2.18	0.000151	10

79	Sulfiredoxin 1 homolog (S. Cerevisiae)	*Srxn1*	2.18	0.000253	2
80	Lamin B1	*Lmnb1*	2.16	0.000229	18
81	Potassium channel tetramerisation domain containing 10	*Kctd10*	2.16	0.000320	5
82	FERM domain containing 6	*Frmd6*	2.15	0.000689	12
83	Dnaj (Hsp40) homolog, subfamily C, member 9	*Dnajc9*	2.15	0.000659	14
84	Biogenesis of lysosomal organelles complex-1, subunit 1 /// retinol dehydrogenase 5	*Bloc1s1 /// Rdh5*	2.14	0.000284	10///10
85	Brain-specific angiogenesis inhibitor 1-associated protein 2	*Baiap2*	2.14	0.000169	11
86	Cyclin D1	*Ccnd1*	2.14	0.000898	7
87	Intraflagellar transport 27	*Ift27*	2.14	0.000913	15
88	IQ motif containing gtpase activating protein 3	*Iqgap3*	2.13	0.000203	3
89	Potassium channel, subfamily K, member 2	*Kcnk2*	2.12	0.000265	1
90	DNA primase, p49 subunit	*Prim1*	2.11	0.000955	10
91	Ubiquitin-conjugating enzyme E2G 2	*Ube2g2*	2.04	0.000248	10
92	DNA segment, Chr 10, Wayne State University 102, expressed	*D10Wsu102e*	2.03	0.000248	10
93	Transcription factor 4	*Tcf4*	2.03	0.000533	18
94	G two S phase expressed protein 1	*Gtse1*	2.02	0.000440	15
95	Single-pass membrane protein with aspartate rich tail 1	*Smdt1*	2.00	0.000124	15
96	Histone deacetylase 5	*Hdac5*	1.99	0.000382	11
97	Solute carrier family 25 (mitochondrial carrier, dicarboxylate transporter), member 10	*Slc25a10*	1.99	0.000292	11
98	Jerky	*Jrk*	1.98	0.000980	15
99	Choline kinase beta	*Chkb*	1.96	0.000867	15
100	RIKEN cdna 1110007C09 gene	*1110007C09Rik*	1.95	0.000419	13
101	Trafficking protein particle complex 1	*Trappc1*	1.93	0.000389	17
102	Dedicator of cytokinesis 9	*Dock9*	1.91	0.000640	14
103	Casein kinase 1, gamma 2	*Csnk1g2*	1.91	0.000480	10
104	Protein inhibitor of activated STAT 3	*Pias3*	1.89	0.000540	3
105	Solute carrier family 48 (heme transporter), member 1	*Slc48a1*	1.89	0.000150	15
106	Centrin 3	*Cetn3*	1.88	0.000109	13
107	PQ loop repeat containing	*Pqlc3*	1.88	0.000818	12
108	Myosin, heavy polypeptide 9, non-muscle	*Myh9*	1.86	0.000686	15
109	Budding uninhibited by benzimidazoles 1 homolog, beta (S. Cerevisiae)	*Bub1b*	1.85	0.000901	2
110	Coatomer protein complex, subunit zeta 1	*Copz1*	1.83	0.000284	15
111	WD repeat domain 1	*Wdr1*	1.81	0.000688	5
112	Mediator complex subunit 30	*Med30*	1.81	0.000463	15
113	Tubulin, alpha 4A	*Tuba4a*	1.78	0.000671	1
114	Hyaluronan mediated motility receptor (RHAMM)	*Hmmr*	1.77	0.000570	11

115	Katanin p60 (atpase-containing) subunit A1	*Katna1*	1.77	0.000892	10
116	Pituitary tumor-transforming gene 1	*Pttg1*	1.77	0.000393	11
117	SEC63-like (S. Cerevisiae)	*Sec63*	1.74	0.000617	10
118	Nuclear factor of activated T cells, cytoplasmic, calcineurin dependent 1	*Nfatc1*	1.70	0.000980	18
119	RIKEN cdna 2700094K13 gene	*2700094K13Rik*	1.70	0.000931	2
120	Proteasome (prosome, macropain) activator subunit 4	*Psme4*	1.69	0.000688	11
121	Mckusick-Kaufman syndrome	*Mkks*	1.69	0.000980	2
122	Family with sequence similarity 114, member A1	*Fam114a1*	1.64	0.000819	5
123	Ankyrin repeat domain 13a	*Ankrd13a*	1.64	0.000737	5
124	SAR1 gene homolog A (S. Cerevisiae)	*Sar1a*	1.61	0.000572	10
125	Tubulin, beta 4B class IVB	*Tubb4b*	1.60	0.000704	2
126	N-deacetylase/N-sulfotransferase (heparan glucosaminyl) 1	*Ndst1*	1.58	0.000877	18
1	Matrix metallopeptidase 13	*Mmp13*	-223.43	0.000000	9
2	Inactive X specific transcripts	*Xist*	-99.33	0.000000	X
3	Serum amyloid A 3	*Saa3*	-59.95	0.000265	7
4	Trypsin 4 /// trypsin 5	*Try4 /// Try5*	-43.01	0.000000	6///6
5	SRY (sex determining region Y)-box 11	*Sox11*	-36.01	0.000000	12
6	Insulin-like growth factor binding protein 5	*Igfbp5*	-35.54	0.000867	1
7	Acyl-coa thioesterase 1	*Acot1*	-32.16	0.000000	12
8	Ceruloplasmin	*Cp*	-29.55	0.000168	3
9	Interleukin 13 receptor, alpha 2	*Il13ra2*	-24.74	0.000000	X
10	Cholinergic receptor, nicotinic, beta polypeptide 1 (muscle)	*Chrnb1*	-22.38	0.000001	11
11	Transmembrane protein 176B	*Tmem176b*	-22.31	0.000000	6
12	Regulator of calcineurin 2	*Rcan2*	-22.14	0.000002	17
13	Fibroblast growth factor 7	*Fgf7*	-22.02	0.000000	2
14	ATP-binding cassette, sub-family G (WHITE), member 2	*Abcg2*	-21.13	0.000000	6
15	Solute carrier family 24 (sodium/potassium/calcium exchanger), member 3	*Slc24a3*	-21.00	0.000001	2
16	Guanine deaminase	*Gda*	-20.04	0.000000	19
17	Lysophosphatidic acid receptor 4	*Lpar4*	-19.84	0.000008	X
18	Epidermal growth factor-containing fibulin-like extracellular matrix protein 1	*Efemp1*	-19.12	0.000000	11
19	Leucine rich repeat protein 1, neuronal	*Lrrn1*	-18.34	0.000002	6
20	Transmembrane protein 176A	*Tmem176a*	-17.55	0.000003	6
21	Aldo-keto reductase family 1, member C18	*Akr1c18*	-16.91	0.000005	13
22	Cadherin 15	*Cdh15*	-16.72	0.000000	8
23	Clusterin	*Clu*	-15.86	0.000002	14
24	Immunoglobulin superfamily, DCC subclass, member 4	*Igdcc4*	-15.85	0.000002	9
25	Collagen, type IV, alpha 2	*Col4a2*	-15.39	0.000000	8
26	Haptoglobin	*Hp*	-15.18	0.000000	8
27	Bradykinin receptor, beta 1	*Bdkrb1*	-15.08	0.000003	12

28	Heparan sulfate 6-O-sulfotransferase 2	*Hs6st2*	-14.08	0.000001	X
29	Sphingomyelin phosphodiesterase, acid-like 3B	*Smpdl3b*	-13.69	0.000163	4
30	S100 calcium binding protein A8 (calgranulin A)	*S100a8*	-13.53	0.000001	3
31	A disintegrin-like and metallopeptidase (reprolysin type) with thrombospondin type 1 motif, 5 (aggrecanase-2)	*Adamts5*	-12.70	0.000168	16
32	Protein kinase D1	*Prkd1*	-12.46	0.000082	12
33	PDZ and LIM domain 3	*Pdlim3*	-12.12	0.000000	8
34	Lipocalin 2	*Lcn2*	-12.07	0.000001	2
35	Acyl-coa thioesterase 1 /// acyl-coa thioesterase 2	*Acot1 /// Acot2*	-11.51	0.000000	12///12
36	Ankyrin repeat domain 1 (cardiac muscle)	*Ankrd1*	-10.93	0.000522	19
37	Collagen, type IV, alpha 1	*Col4a1*	-10.79	0.000424	8
38	Desmocollin 2	*Dsc2*	-10.37	0.000000	18
39	Chemokine (C-X-C motif) ligand 5	*Cxcl5*	-10.16	0.000069	5
40	Connective tissue growth factor	*Ctgf*	-9.85	0.000001	10
41	Starch binding domain 1	*Stbd1*	-9.31	0.000007	5
42	Nuclear paraspeckle assembly transcript 1 (non-protein coding)	*Neat1*	-8.87	0.000347	19
43	Titin	*Ttn*	-8.76	0.000145	2
44	Ectonucleotide pyrophosphatase/phosphodiesterase 5	*Enpp5*	-8.71	0.000106	17
45	Nephronectin	*Npnt*	-8.34	0.000242	3
46	Signal-regulatory protein alpha	*Sirpa*	-8.32	0.000472	2
47	RIKEN cdna D430019H16 gene	*D430019H16Rik*	-8.27	0.000001	12
48	Matrix metallopeptidase 3	*Mmp3*	-8.26	0.000529	9
49	Scavenger receptor class A, member 3	*Scara3*	-8.21	0.000000	14
50	CDC42 effector protein (Rho gtpase binding) 3	*Cdc42ep3*	-7.96	0.000250	17
51	CCAAT/enhancer binding protein (C/EBP), delta	*Cebpd*	-7.94	0.000009	16
52	Serum deprivation response	*Sdpr*	-7.90	0.000504	1
53	Armadillo repeat containing, X-linked 4	*Armcx4*	-7.83	0.000001	9
54	Iroquois related homeobox 3	*Irx3*	-7.48	0.000000	8
55	Protocadherin 18	*Pcdh18*	-7.47	0.000011	3
56	Thrombospondin 3	*Thbs3*	-7.44	0.000003	3
57	SH3 domain binding glutamic acid-rich protein like 2	*Sh3bgrl2*	-6.99	0.000092	9
58	Atpase, Na+/K+ transporting, alpha 3 polypeptide	*Atp1a3*	-6.69	0.000997	7
59	Interleukin 1 receptor antagonist	*Il1rn*	-6.67	0.000027	2
60	Very low density lipoprotein receptor	*Vldlr*	-6.54	0.000097	19
61	Solute carrier family 44, member 2	*Slc44a2*	-6.46	0.000275	9
62	Adenosine A2b receptor	*Adora2b*	-6.43	0.000005	11
63	Paired box 7	*Pax7*	-6.37	0.000338	4
64	Solute carrier family 16 (monocarboxylic acid transporters), member 1	*Slc16a1*	-6.21	0.000473	3
65	Oligodendrocyte transcription factor 1	*Olig1*	-6.19	0.000001	16
66	Collagen, type V, alpha 2	*Col5a2*	-6.15	0.000015	1

67	Frizzled homolog 4 (Drosophila)	*Fzd4*	-5.96	0.000006	7
68	Family with sequence similarity 132, member A	*Fam132a*	-5.95	0.000175	1
69	Abhydrolase domain containing 6	*Abhd6*	-5.89	0.000007	14
70	Zinc finger protein, multitype 2	*Zfpm2*	-5.86	0.000064	15
71	CDC42 effector protein (Rho gtpase binding) 5	*Cdc42ep5*	-5.78	0.000011	7
72	Glycoprotein 49 A /// leukocyte immunoglobulin-like receptor, subfamily B, member 4	*Gp49a /// Lilrb4*	-5.71	0.000027	10
73	Family with sequence similarity 213, member A	*Fam213a*	-5.71	0.000236	10
74	WAP four-disulfide core domain 2	*Wfdc2*	-5.65	0.000008	2
75	Membrane bound O-acyltransferase domain containing 2	*Mboat2*	-5.63	0.000021	12
76	Histocompatibility 2, D region locus 1	*H2-D1*	-5.59	0.000870	17
77	Cdna sequence BC002189	*BC002189*	-5.46	0.000480	3
78	Leptin receptor	*Lepr*	-5.38	0.000704	4
79	Junction adhesion molecule 3	*Jam3*	-5.32	0.000014	9
80	Protein tyrosine phosphatase, receptor type, D	*Ptprd*	-5.26	0.000048	4
81	Nucleolar protein 3 (apoptosis repressor with CARD domain)	*Nol3*	-5.19	0.000311	8
82	Secretory leukocyte peptidase inhibitor	*Slpi*	-5.13	0.000009	2
83	Cathepsin F	*Ctsf*	-5.04	0.000530	19
84	Kruppel-like factor 2 (lung)	*Klf2*	-4.96	0.000008	8
85	Nidogen 2	*Nid2*	-4.81	0.000002	14
86	Transmembrane protein 38B	*Tmem38b*	-4.75	0.000435	4
87	Histocompatibility 2, K1, K region	*H2-K1*	-4.72	0.000254	17
88	Ankyrin repeat domain 10	*Ankrd10*	-4.67	0.000110	8
89	Integrin alpha 6	*Itga6*	-4.67	0.000012	2
90	Solute carrier family 40 (iron-regulated transporter), member 1	*Slc40a1*	-4.54	0.000621	1
91	Transforming growth factor, beta 2	*Tgfb2*	-4.48	0.000045	1
92	Scavenger receptor class A, member 5 (putative)	*Scara5*	-4.41	0.000010	14
93	Distal-less homeobox 4	*Dlx4*	-4.26	0.000037	11
94	Histocompatibility 13	*H13*	-4.06	0.000275	2
95	S100 calcium binding protein A16	*S100a16*	-3.98	0.000004	3
96	MACRO domain containing 1	*Macrod1*	-3.98	0.000403	19
97	Oncostatin M receptor	*Osmr*	-3.96	0.000104	15
98	Zinc finger protein of the cerebellum 2	*Zic2*	-3.95	0.000016	14
99	Coxsackie virus and adenovirus receptor	*Cxadr*	-3.94	0.000034	16
100	3-hydroxybutyrate dehydrogenase, type 1	*Bdh1*	-3.91	0.000042	16
101	Plasminogen activator, tissue	*Plat*	-3.90	0.000621	8
102	Estrogen related receptor, beta	*Esrrb*	-3.90	0.000162	12
103	Myomesin 2	*Myom2*	-3.89	0.000091	8
104	Arrestin domain containing 4	*Arrdc4*	-3.88	0.000980	7
105	Acyl-coa thioesterase 4	*Acot4*	-3.87	0.000114	12
106	Basonuclin 1	*Bnc1*	-3.86	0.000014	7
107	RAR-related orphan receptor gamma	*Rorc*	-3.82	0.000083	3

108	CD59a antigen	*Cd59a*	-3.79	0.000621	2
109	Ferredoxin 1-like /// Raver1-Fdx1l readthrough	*Fdx1l /// Raver1-fdx1l*	-3.75	0.000005	9
110	Schlafen 2	*Slfn2*	-3.69	0.000248	11
111	Serglycin	*Srgn*	-3.68	0.000113	10
112	Junction adhesion molecule 2	*Jam2*	-3.68	0.000213	16
113	Brain expressed myelocytomatosis oncogene	*Bmyc*	-3.51	0.000229	2
114	Protocadherin 1	*Pcdh1*	-3.50	0.000931	18
115	Interferon gamma inducible protein 30	*Ifi30*	-3.49	0.000049	8
116	Acyl-coa thioesterase 2	*Acot2*	-3.47	0.000080	12
117	Zinc finger protein of the cerebellum 5	*Zic5*	-3.46	0.000044	14
118	Complement component 3	*C3*	-3.45	0.000097	17
119	Solute carrier family 6 (neurotransmitter transporter, glycine), member 9	*Slc6a9*	-3.44	0.000011	4
120	Dnaj (Hsp40) homolog, subfamily B, member 9	*Dnajb9*	-3.41	0.000158	12
121	F-box protein 32	*Fbxo32*	-3.41	0.000558	15
122	Spla/ryanodine receptor domain and SOCS box containing 1	*Spsb1*	-3.39	0.000588	4
123	Tripartite motif-containing 30A	*Trim30a*	-3.37	0.000463	7
124	Muscle, skeletal, receptor tyrosine kinase	*Musk*	-3.37	0.000010	4
125	Selenium binding protein 1	*Selenbp1*	-3.35	0.000303	3
126	Ectonucleotide pyrophosphatase/phosphodiesterase 2	*Enpp2*	-3.35	0.000572	15
127	Presenilin 2	*Psen2*	-3.33	0.000913	1
128	Sulfide quinone reductase-like (yeast)	*Sqrdl*	-3.31	0.000478	2
129	Sialic acid acetylesterase	*Siae*	-3.30	0.000336	9
130	RIKEN cdna 1190002N15 gene	*1190002N15Rik*	-3.29	0.000473	9
131	B cell leukemia/lymphoma 3	*Bcl3*	-3.29	0.000248	7
132	Deiodinase, iodothyronine, type II	*Dio2*	-3.26	0.000042	12
133	Zinc finger and BTB domain containing 46	*Zbtb46*	-3.22	0.000047	2
134	Ring finger protein 149	*Rnf149*	-3.21	0.000065	1
135	Frizzled homolog 6 (Drosophila)	*Fzd6*	-3.20	0.000570	15
136	Nuclear factor of kappa light polypeptide gene enhancer in B cells inhibitor, zeta	*Nfkbiz*	-3.17	0.000008	16
137	Prolactin family 7, subfamily c, member 1	*Prl7c1*	-3.14	0.000027	13
138	Family with sequence similarity 195, member A	*Fam195a*	-3.12	0.000027	17
139	Ribosomal protein L22	*Rpl22*	-3.11	0.000023	4
140	Prostaglandin reductase 1	*Ptgr1*	-3.09	0.000090	4
141	Phosphoenolpyruvate carboxykinase 2 (mitochondrial)	*Pck2*	-3.05	0.000354	14
142	Phosphoribosyl pyrophosphate synthetase 1-like 3	*Prps1l3*	-3.05	0.000354	12
143	Paired box 8	*Pax8*	-3.04	0.000123	2
144	Aldehyde dehydrogenase 18 family, member A1	*Aldh18a1*	-2.99	0.000080	19
145	Transmembrane protein 132A	*Tmem132a*	-2.98	0.000104	19
146	Activating transcription factor 3	*Atf3*	-2.98	0.000129	1

147	Follistatin	*Fst*	-2.91	0.000063	13
148	Gap junction protein, beta 3	*Gjb3*	-2.87	0.000801	4
149	Ribosomal protein S6	*Rps6*	-2.86	0.000015	4
150	Sortilin 1	*Sort1*	-2.84	0.000229	3
151	Rho guanine nucleotide exchange factor (GEF) 5	*Arhgef5*	-2.84	0.000229	6
152	Quaking	*Qk*	-2.83	0.000275	17
153	Pseudouridine synthase 3	*Pus3*	-2.82	0.000615	9
154	Glucuronyl C5-epimerase	*Glce*	-2.82	0.000128	9
155	Coiled-coil domain containing 80	*Ccdc80*	-2.79	0.000439	16
156	NADH dehydrogenase (ubiquinone) 1 alpha subcomplex, assembly factor 4	*Ndufaf4*	-2.79	0.000078	4
157	Oxidative stress induced growth inhibitor family member 2	*Osgin2*	-2.77	0.000194	4
158	Glutamic pyruvate transaminase (alanine aminotransferase) 2	*Gpt2*	-2.73	0.000446	8
159	Predicted gene 13139	*Gm13139*	-2.71	0.000056	4
160	Phosphoserine phosphatase	*Psph*	-2.71	0.000042	5
161	Zinc finger protein 703	*Zfp703*	-2.67	0.000584	8
162	4-aminobutyrate aminotransferase	*Abat*	-2.66	0.000598	16
163	Insulin-like growth factor binding protein 3	*Igfbp3*	-2.64	0.000186	11
164	Zinc and ring finger 1	*Znrf1*	-2.61	0.000407	8
165	Cathepsin Z	*Ctsz*	-2.58	0.000019	2
166	RAB20, member RAS oncogene family	*Rab20*	-2.57	0.000353	8
167	STEAP family member 4	*Steap4*	-2.56	0.000055	5
168	Kruppel-like factor 9	*Klf9*	-2.56	0.000110	19
169	Procollagen lysine, 2-oxoglutarate 5-dioxygenase 2	*Plod2*	-2.56	0.000621	9
170	Ribosomal protein L5 /// ribisomal protein L5, pseudogene 2	*Rpl5 /// Rpl5-ps2*	-2.51	0.000044	5///2
171	Spla/ryanodine receptor domain and SOCS box containing 4	*Spsb4*	-2.51	0.000638	9
172	H19, imprinted maternally expressed transcript /// microrna 675	*H19 /// Mir675*	-2.51	0.000265	7///7
173	TGFB-induced factor homeobox 1	*Tgif1*	-2.48	0.000064	17
174	YTH domain family 2	*Ythdf2*	-2.47	0.000653	4
175	Ring finger protein 11	*Rnf11*	-2.45	0.000859	4
176	Influenza virus NS1A binding protein	*Ivns1abp*	-2.43	0.000653	1
177	DNA segment, Chr 16, ERATO Doi 472, expressed	*D16Ertd472e*	-2.42	0.000368	16
178	Fin bud initiation factor homolog (zebrafish)	*Fibin*	-2.42	0.000812	2
179	Zinc finger protein 566	*Zfp566*	-2.41	0.000859	7
180	Nuclear protein transcription regulator 1	*Nupr1*	-2.38	0.000325	7
181	Nicotinamide N-methyltransferase	*Nnmt*	-2.36	0.000677	9
182	TEA domain family member 2	*Tead2*	-2.35	0.000325	7
183	Pecanex-like 4 (Drosophila)	*Pcnxl4*	-2.32	0.000514	12
184	CCAAT/enhancer binding protein (C/EBP), beta	*Cebpb*	-2.32	0.000534	2
185	Cytochrome c oxidase assembly factor 7	*Coa7*	-2.32	0.000148	4
186	Caspase 7	*Casp7*	-2.31	0.000407	19

187	SR-related CTD-associated factor 8	*Scaf8*	-2.31	0.000786	17
188	Latent transforming growth factor beta binding protein 3	*Ltbp3*	-2.30	0.000517	19
189	C-type lectin domain family 4, member e	*Clec4e*	-2.29	0.000454	6
190	Isovaleryl coenzyme A dehydrogenase	*Ivd*	-2.29	0.000360	2
191	Palladin, cytoskeletal associated protein	*Palld*	-2.29	0.000066	8
192	Disabled 2 interacting protein	*Dab2ip*	-2.28	0.000136	2
193	Sorting nexin 18	*Snx18*	-2.28	0.000435	13
194	Zinc finger protein 639	*Zfp639*	-2.28	0.000083	3
195	Camello-like 1	*Cml1*	-2.28	0.000714	6
196	Mitogen-activated protein kinase 12	*Mapk12*	-2.27	0.000285	15
197	Fibrillin 1	*Fbn1*	-2.26	0.000126	2
198	Complement component 2 (within H-2S)	*C2*	-2.26	0.000229	17
199	Rho GDP dissociation inhibitor (GDI) gamma	*Arhgdig*	-2.26	0.000311	17
200	Phospholipase A2, group XV	*Pla2g15*	-2.25	0.000248	8
201	Solute carrier family 3 (activators of dibasic and neutral amino acid transport), member 2	*Slc3a2*	-2.25	0.000027	19
202	Post-GPI attachment to proteins 2	*Pgap2*	-2.24	0.000125	7
203	ATP5S-like	*Atp5sl*	-2.23	0.000378	7
204	Apurinic/apyrimidinic endonuclease 1	*Apex1*	-2.23	0.000505	14
205	General transcription factor IIA, 1-like	*Gtf2a1l*	-2.23	0.000826	17
206	Collagen, type V, alpha 1	*Col5a1*	-2.22	0.000316	2
207	FK506 binding protein-like	*Fkbpl*	-2.21	0.000901	17
208	Myelocytomatosis oncogene	*Myc*	-2.21	0.000310	15
209	Golgi associated, gamma adaptin ear containing, ARF binding protein 2	*Gga2*	-2.21	0.000107	7
210	Phosphatidylinositol glycan anchor biosynthesis, class K	*Pigk*	-2.20	0.000355	3
211	Histocompatibility 2, Q region locus 4 /// histocompatibility 2, Q region locus 6 /// histocompatibility 2, Q region locus 8 /// histocompatibility 2, Q region locus 6-like	*H2-Q4 /// H2-Q6 /// H2-Q8 /// LOC68395*	-2.20	0.000538	17
212	Interferon (alpha and beta) receptor 2	*Ifnar2*	-2.19	0.000060	16
213	Chemokine (C-C motif) ligand 25	*Ccl25*	-2.18	0.000288	8
214	SET domain, bifurcated 1	*Setdb1*	-2.18	0.000709	3
215	Expressed sequence AI837181	*AI837181*	-2.18	0.000287	19
216	Protein phosphatase 1, regulatory (inhibitor) subunit 14c	*Ppp1r14c*	-2.18	0.000539	10
217	SKI-like	*Skil*	-2.17	0.000548	3
218	WD repeat domain 20	*Wdr20*	-2.13	0.000463	12
219	3-phosphoglycerate dehydrogenase	*Phgdh*	-2.12	0.000597	3
220	Diacylglycerol O-acyltransferase 2	*Dgat2*	-2.11	0.000812	7
221	Cleavage stimulation factor, 3' pre-RNA subunit 2, tau	*Cstf2t*	-2.10	0.000186	19
222	Transmembrane protein 11	*Tmem11*	-2.09	0.000470	11
223	RAS p21 protein activator 3	*Rasa3*	-2.03	0.000202	8
224	Mannan-binding lectin serine peptidase 1	*Masp1*	-2.00	0.000460	16

225	Leptin receptor overlapping transcript-like 1	*Leprotl1*	-2.00	0.000913	8
226	Solute carrier family 39 (zinc transporter), member 14	*Slc39a14*	-1.99	0.000730	14
227	Heat shock protein 5	*Hspa5*	-1.99	0.000616	2
228	Bicaudal D homolog 1 (Drosophila)	*Bicd1*	-1.98	0.000837	6
229	Iroquois related homeobox 1 (Drosophila)	*Irx1*	-1.97	0.000208	13
230	Dual specificity phosphatase 16	*Dusp16*	-1.95	0.000210	6
231	Interleukin 4 receptor, alpha	*Il4ra*	-1.95	0.000329	7
232	Queuine trna-ribosyltransferase 1	*Qtrt1*	-1.95	0.000341	9
233	Rosbin, round spermatid basic protein 1	*Rsbn1*	-1.92	0.000737	3
234	Chloride channel 5	*Clcn5*	-1.88	0.000326	X
235	Cytotoxic T lymphocyte-associated protein 2 beta	*Ctla2b*	-1.86	0.000702	13
236	Methylenetetrahydrofolate dehydrogenase (NAD+ dependent), methenyltetrahydrofolate cyclohydrolase	*Mthfd2*	-1.86	0.000980	6
237	Complement component 1, q subcomponent binding protein	*C1qbp*	-1.83	0.000287	11
238	Carbohydrate (N-acetylgalactosamine 4-0) sulfotransferase 14	*Chst14*	-1.82	0.000228	2
239	Peroxiredoxin 5	*Prdx5*	-1.78	0.000389	19
240	Zinc finger protein 777	*Zfp777*	-1.77	0.000660	6
241	Feminization 1 homolog b (C. Elegans)	*Fem1b*	-1.76	0.000799	9
242	Carnitine palmitoyltransferase 2	*Cpt2*	-1.76	0.000686	4
243	Seven in absentia 2	*Siah2*	-1.76	0.000702	3
244	RB1-inducible coiled-coil 1	*Rb1cc1*	-1.74	0.000311	1
245	Centrosomal protein 68	*Cep68*	-1.71	0.000653	11
246	Ariadne homolog 2 (Drosophila)	*Arih2*	-1.71	0.000540	9
247	Ring finger protein 113A1	*Rnf113a1*	-1.67	0.000968	X
248	Lysosomal-associated membrane protein 2	*Lamp2*	-1.66	0.000473	X
249	60S ribosomal protein L23-like /// 60S ribosomal protein L23-like /// ribosomal protein L23	*LOC100044627 /// LOC100862455 /// Rpl23*	-1.57	0.000980	17

Table S7. The Gene Set Enrichment analysis

Pathway	Source of data	Down-regulated genes		
		P. val	Q.val	Genes involved
Extracellular matrix organization	Reactome	3.02E-07	9.42E-05	*Adamts5; Try5; Ltbp3; Jam2; Jam3; Efemp1; Try4; Mmp13; Plod2; Tgfb2; Itga6; Fbn1; Col5a2; Nid2; Col5a1; Col4a2; Col4a1; Mmp3*
Protein digestion and absorption - Mus musculus (mouse)	KEGG	4.25E-05	6.63E-03	*Try5; Try4; Col5a2; Slc3a2; Col5a1; Atp1a3; Col4a2; Col4a1*
Activation of C3 and C5	Reactome	1.97E-03	3.08E-02	*C3; C2*
Activation of Matrix Metalloproteinases	Reactome	2.04E-03	3.08E-02	*Try5; Try4; Mmp3; Mmp13*
Acyl-coa hydrolysis	Mousecyc	4.35E-04	1.36E-02	*Acot4; Acot2; Acot1*
Allograft rejection - Mus musculus (mouse)	KEGG	8.83E-03	5.74E-02	*H2-Q4; H2-K1; H2-D1; H2-Q6*
Amino acid synthesis and interconversion (transamination)	Reactome	9.38E-04	2.66E-02	*Aldh18a1; Gpt2; Phgdh; Psph*
Antigen Presentation: Folding, assembly and peptide loading of class I MHC	Reactome	1.29E-04	1.01E-02	*H2-Q4; H2-K1; H2-D1; H2-Q6; Hspa5*
Antigen processing and presentation - Mus musculus (mouse)	KEGG	1.35E-03	2.73E-02	*H2-K1; H2-D1; Hspa5; H2-Q4; H2-Q6; Ifi30*
Antigen processing-Cross presentation	Reactome	2.24E-03	3.08E-02	*H2-Q4; H2-K1; H2-D1; H2-Q6*
Assembly of collagen fibrils and other multimeric structures	Reactome	1.40E-03	2.73E-02	*Mmp3; Col4a2; Itga6; Col4a1; Mmp13*
Basigin interactions	Reactome	5.23E-03	4.41E-02	*Slc16a1; Slc3a2; Itga6*
Cell adhesion molecules (cams) - Mus musculus (mouse)	KEGG	2.27E-03	3.08E-02	*H2-K1; H2-D1; Cdh15; Jam2; Jam3; H2-Q4; H2-Q6; Itga6*
Cell surface interactions at the vascular wall	Reactome	7.33E-03	5.08E-02	*Cxadr; Jam2; Sirpa; Jam3; Slc3a2; Itga6; Slc16a1*
Cellular senescence - Mus musculus (mouse)	KEGG	4.32E-03	4.22E-02	*H2-K1; H2-D1; H2-Q4; H2-Q6; Tgfb2; Igfbp3; Mapk12; Myc*
Class I MHC mediated antigen processing & presentation	Reactome	6.43E-03	4.78E-02	*H2-K1; H2-D1; Znrf1; Hspa5; Siah2; H2-Q4; H2-Q6; Arih2; Fbxo32; Spsb1; Spsb4*
Collagen biosynthesis and modifying enzymes	Reactome	2.49E-03	3.11E-02	*Col5a2; Plod2; Col5a1; Col4a2; Col4a1*
Collagen chain trimerization	Reactome	3.22E-03	3.35E-02	*Col5a2; Col5a1; Col4a2; Col4a1*
Collagen degradation	Reactome	3.22E-03	3.35E-02	*Try5; Try4; Mmp3; Mmp13*
Collagen formation	Reactome	9.03E-05	9.40E-03	*Mmp13; Plod2; Itga6; Col5a2; Col5a1; Col4a2; Col4a1; Mmp3*
Complement Activation, Classical Pathway	Wikipathways	1.68E-03	2.92E-02	*C3; C2; Masp1*

Complement and Coagulation Cascades	Wikipathways	2.27E-04	1.01E-02	*Bdkrb1; Cd59a; Masp1; Plat; C3; C2*
Complement and coagulation cascades - Mus musculus (mouse)	KEGG	2.59E-04	1.01E-02	*Bdkrb1; Cd59a; Masp1; Plat; Clu; C3; C2*
Complement cascade	Reactome	5.71E-03	4.69E-02	*C3; Clu; Cd59a; Masp1; C2*
ECM-receptor interaction - Mus musculus (mouse)	KEGG	6.66E-03	4.83E-02	*Npnt; Itga6; Col4a2; Col4a1; Thbs3*
Elastic fibre formation	Reactome	1.49E-03	2.74E-02	*Efemp1; Tgfb2; Ltbp3; Fbn1*
Endosomal/Vacuolar pathway	Reactome	1.99E-04	1.01E-02	*H2-Q4; H2-K1; H2-D1; H2-Q6*
Exercise-induced Circadian Regulation	Wikipathways	5.17E-03	4.41E-02	*Cebpb; Qk; Stbd1; Klf9*
Ferroptosis - Mus musculus (mouse)	KEGG	2.47E-03	3.11E-02	*Slc3a2; Cp; Slc40a1; Slc39a14*
Focal Adhesion-PI3K-Akt-mtor-signaling pathway	Wikipathways	2.22E-03	3.08E-02	*Ifnar2; Osmr; Il4ra; Thbs3; Itga6; Col5a2; Rps6; Col5a1; Col4a2; Col4a1; Fgf7; Lpar4*
Graft-versus-host disease - Mus musculus (mouse)	KEGG	9.96E-03	5.85E-02	*H2-Q4; H2-K1; H2-D1; H2-Q6*
Hemostasis	Reactome	9.67E-03	5.85E-02	*Cxadr; Tgfb2; Sirpa; Jam2; Jam3; Zfpm2; Itga6; Plat; Slc3a2; Slc16a1; c1qbp; Lamp2; Srgn; Clu; Abhd6; Ttn*
IL-17 signaling pathway - Mus musculus (mouse)	KEGG	3.19E-04	1.11E-02	*Cebpb; Cxcl5; Mmp13; Lcn2; S100a8; Mapk12; Mmp3*
Immune System	Reactome	9.72E-03	5.85E-02	*Il1rn; Znrf1; Sirpa; Try5; Try4; Masp1; Ifnar2; Clu; Prl7c1; Slc44a2; Arih2; Hspa5; Il4ra; Cd59a; Hp; Ctsf; Ctsz; Stbd1; Mapk12; C3; C2; Spsb1; Spsb4; Slpi; Il13ra2; H2-D1; H2-Q4; H2-Q6; H2-K1; Osmr; S100a8; Fbxo32; Cxadr; Siah2; Lamp2*
Immunoregulatory interactions between a Lymphoid and a non-Lymphoid cell	Reactome	3.08E-03	3.35E-02	*Cxadr; H2-K1; H2-D1; H2-Q4; H2-Q6; C3*
Innate Immune System	Reactome			
Integrin cell surface interactions	Reactome	2.48E-04	1.01E-02	*Jam2; Jam3; Itga6; Fbn1; Col4a2; Col4a1*
Lysosome - Mus musculus (mouse)	KEGG	8.72E-03	5.74E-02	*Pla2g15; Ctsf; Ctsz; Gga2; Lamp2; Sort1*
Metal ion SLC transporters	Reactome	3.16E-03	3.35E-02	*Cp; Slc40a1; Slc39a14*
Molecules associated with elastic fibres	Reactome	9.61E-03	5.85E-02	*Efemp1; Tgfb2; Ltbp3*
Neutrophil degranulation	Reactome	5.97E-03	4.69E-02	*Slpi; Slc44a2; H2-D1; Sirpa; Try5; H2-Q4; Hp; H2-Q6; Cd59a; H2-K1; Ctsz; Lamp2; Stbd1; Try4; C3; S100a8*
Pertussis - Mus musculus (mouse)	KEGG	4.59E-03	4.34E-02	*C3; Casp7; Cxcl5; Mapk12; C2*

PI3K-Akt signaling pathway - Mus musculus (mouse)	KEGG	5.12E-03	4.41E-02	*Pck2; Il4ra; Thbs3; Itga6; Ifnar2; Rps6; Osmr; Col4a2; Col4a1; Fgf7; Myc; Lpar4*
Post-translational protein phosphorylation	Reactome	8.08E-03	5.48E-02	*Fbn1; Igfbp3; Igfbp5; C3; Cp; Tmem132a*
Serine biosynthesis	Mousecyc	1.20E-03	2.70E-02	*Phgdh; Psph*
Serine biosynthesis	Reactome	6.84E-03	4.85E-02	*Phgdh; Psph*
Signaling by PDGF	Reactome	6.40E-03	4.78E-02	*Col4a2; Thbs3; Col4a1; Plat*
Signaling pathways regulating pluripotency of stem cells - Mus musculus (mouse)	KEGG	3.50E-03	3.53E-02	*Fzd4; Fzd6; Esrrb; Skil; Mapk12; Myc; Setdb1*
Superpathway of serine and glycine biosynthesis I	Mousecyc	2.93E-03	3.35E-02	*Phgdh; Psph*
TNF signaling pathway - Mus musculus (mouse)	KEGG	4.92E-03	4.41E-02	*Casp7; Cebpb; Dab2ip; Mapk12; Mmp3; Bcl3*
Transcriptional misregulation in cancer - Mus musculus (mouse)	KEGG	1.21E-03	2.70E-02	*Cebpb; Nupr1; Plat; Nfkbiz; Igfbp3; Pax7; Mmp3; Myc; Pax8*
Transport of bile salts and organic acids, metal ions and amine compounds	Reactome	1.05E-03	2.70E-02	*Slc44a2; Slc40a1; Slc39a14; Slc16a1; Slc6a9; Cp*
Viral myocarditis - Mus musculus (mouse)	KEGG	6.01E-03	4.69E-02	*H2-Q4; H2-K1; H2-D1; H2-Q6; Cxadr*
**Pathway**	**Source of data**		**Up-regulated genes**	
		P. val	Q.val	Genes involved
APC/C-mediated degradation of cell cycle proteins	Reactome	3,06E-03	3,60E-02	*Pttg1; Cdkn1a; Bub1b*
Cell Cycle	Reactome	4,47E-07	5,17E-05	*Hmmr; Ncaph2; Lmnb1; Bub1b; Ccnd1; Tubb4b; Cdkn1a; Gtse1; Ska1; Dna2; Prim1; Tuba4a; Pttg1; Phlda1; Birc5; Kif20a*
Cell cycle	Wikipathways	3,12E-03	3,60E-02	*Pttg1; Cdkn1a; Hdac5; Bub1b*
Cell Cycle Checkpoints	Reactome	5,84E-03	5,19E-02	*Dna2; Bub1b; Birc5; Gtse1; Ska1; Cdkn1a*
Cell Cycle, Mitotic	Reactome	7,89E-08	1,82E-05	*Hmmr; Ncaph2; Lmnb1; Bub1b; Ccnd1; Tubb4b; Cdkn1a; Gtse1; Ska1; Dna2; Prim1; Tuba4a; Pttg1; Phlda1; Birc5; Kif20a*
G1 to S cell cycle control	Wikipathways	8,72E-03	6,10E-02	*Ccnd1; Cdkn1a; Prim1*
G1/S Transition	Reactome	8,72E-03	6,10E-02	*Ccnd1; Cdkn1a; Prim1*
G2/M Transition	Reactome	2,10E-04	1,32E-02	*Hmmr; Tubb4b; Cdkn1a; Gtse1; Tuba4a; Phlda1*
Graft-versus-host disease - Mus musculus (mouse)	KEGG			
Hemostasis	Reactome			
Hepatitis B - Mus musculus (mouse)	KEGG	4,72E-04	1,56E-02	*Nfatc1; Ccnd1; Birc5; Creb3l2; Cdkn1a; Apaf1*
Hippo signaling pathway - Mus musculus (mouse)	KEGG	6,98E-04	2,02E-02	*Itgb2; Ccnd1; Birc5; Sox2; Frmd6; Wwc1*
Human T-cell leukemia virus 1 infection - Mus musculus (mouse)	KEGG	1,38E-03	2,75E-02	*Nfatc1; Itgb2; Bub1b; Ccnd1; Cdkn1a; Creb3l2; Pttg1*

Innate Immune System	Reactome	2,97E-03	3,60E-02	*Nfatc1; Itgb2; Glipr1; Trappc1; c3ar1; Vnn1; Fgl2; Frk; Mettl7a1; Dnm1; Tubb4b; Baiap2; Mettl7a2; Dusp6; Apaf1*
Lagging Strand Synthesis	Reactome	9,19E-03	6,25E-02	*Dna2; Prim1*
Legionellosis - Mus musculus (mouse)	KEGG	7,23E-03	5,97E-02	*Sar1a; Itgb2; Apaf1*
M Phase	Reactome	4,06E-04	1,56E-02	*Ncaph2; Lmnb1; Bub1b; Tubb4b; Birc5; Ska1; Pttg1; Tuba4a; Kif20a*
Metabolism of carbohydrates	Reactome	7,98E-03	6,10E-02	*Hmmr; Chst11; Ndst1; Hk1; Pfkp; Slc25a10*
Mitotic G2-G2/M phases	Reactome	2,29E-04	1,32E-02	*Hmmr; Tubb4b; Cdkn1a; Gtse1; Tuba4a; Phlda1*
Mitotic Prometaphase	Reactome	8,69E-03	6,10E-02	*Ska1; Tuba4a; Birc5; Tubb4b; Bub1b*
Molecules associated with elastic fibres	Reactome			
Neutrophil degranulation	Reactome	1,55E-03	2,75E-02	*Itgb2; Glipr1; Trappc1; c3ar1; Vnn1; Fgl2; Frk; Mettl7a2; Tubb4b; Mettl7a1; Apaf1*
Oxidative Damage	Wikipathways	2,85E-03	3,60E-02	*C3ar1; Cdkn1a; Apaf1*
P53 signaling	Wikipathways	1,26E-03	2,75E-02	*Ccnd1; Cdkn1a; Gtse1; Apaf1*
P53 signaling pathway - Mus musculus (mouse)	KEGG	1,48E-03	2,75E-02	*Ccnd1; Cdkn1a; Gtse1; Apaf1*
Parkinsons Disease Pathway	Wikipathways	2,29E-03	3,60E-02	*Lrrk2; Apaf1; Ube2g2*
Podnet- protein-protein interactions in the podocyte	Wikipathways	3,30E-04	1,53E-02	*Sema3a; Birc5; Ccnd1; Wwc1; Myh9; Khdrbs3; Dnm1; Baiap2; Cdkn1a*
Primary Focal Segmental Glomerulosclerosis FSGS	Wikipathways	1,48E-03	2,75E-02	*Cd80; Cdkn1a; Myh9; Dnm1*
Processive synthesis on the lagging strand	Reactome	5,37E-03	5,09E-02	*Dna2; Prim1*
Regulation of mitotic cell cycle	Reactome	3,06E-03	3,60E-02	*Pttg1; Cdkn1a; Bub1b*
Removal of the Flap Intermediate	Reactome	4,72E-03	5,04E-02	*Dna2; Prim1*
S Phase	Reactome	5,51E-03	5,09E-02	*Ccnd1; Cdkn1a; Dna2; Prim1*
SCF(Skp2)-mediated degradation of p27/p21	Reactome	6,06E-03	5,19E-02	*Ccnd1;*
Signaling by Non-Receptor Tyrosine Kinases	Reactome	5,02E-03	5,04E-02	*Ccnd1; Khdrbs3; Lrrk2*
Signaling by PTK6	Reactome	5,02E-03	5,04E-02	*Ccnd1; Khdrbs3; Lrrk2*
Signaling by Rho gtpases	Reactome	8,11E-03	6,10E-02	*Bub1b; Birc5; Myh9; Ska1; Rhoj; Baiap2; Arhgap18; Iqgap3*
The role of GTSE1 in G2/M progression after G2 checkpoint	Reactome	2,52E-03	3,60E-02	*Cdkn1a; Gtse1*

Table S8. Table of contrasts (post-hoc tests) from comparison of invasiveness of JUN-3 sarcoma cells treated with Bindarit (Bind), Maraviroc (Marav) or their combination (Comb) compared to untreated control. Beta= effect size – the difference between cell lines with respect to given evaluated measure. CI_L and CI_U= limits of 95% confidence intervals for the beta, based on Bias-Corrected and Accelerated (BCa) bootstrap simulations. p= raw p-value from permutational t-test. fdr_p= p-value corrected by False Discovery Rate correction for multiple comparison. See methods for definition of the measures states and capacities.

(e) Motility	Beta	CI_L	CI_U	p	fdr_p
ctrl vs. bind	0.62	0.11	1.14	0.0396	0.0594
ctrl vs. marav	0.31	-0.03	0.59	0.0730	0.0730
ctrl vs.comb	0.48	1.10	1.67	<0.001	<0.001

Table S9. Genes with significantly changed expression in JUN-2 compared to JUN-3

Gene.title	Gene.symbol	2^logFC	adj.p.val
Carbonic anhydrase 3	*Car3*	676,834106	9,66e-13
Ankyrin repeat and socs box-containing 5	*Asb5*	337,553761	9,47e-12
Protease, serine 23	*Prss23*	304,523051	4,05e-11
Myogenic factor 5	*Myf5*	294,864431	2,69e-12
Paternally expressed 3	*Peg3*	263,804858	1,20e-12
Inactive x specific transcripts	*Xist*	239,132145	5,47e-12
Matrix metallopeptidase 13	*Mmp13*	233,791469	9,66e-13
Zinc finger protein of the cerebellum 1	*Zic1*	173,50496	4,02e-12
Serine (or cysteine) peptidase inhibitor, clade b, member 9g	*Serpinb9g*	133,606134	5,46e-11
Serum amyloid a 3	*Saa3*	121,599123	1,63e-11
Predicted gene 11397 /// serine (or cysteine) peptidase inhibitor, clade b, member 9e /// serine (or cysteine) peptidase inhibitor, clade b, member 9f /// serine (or cysteine) peptidase inhibitor, clade b, member 9g	*Gm11397 /// serpinb9e /// serpinb9f /// serpinb9g*	106,20245	1,65e-11
Histocompatibility 2, class ii antigen a, beta 1	*H2-ab1*	105,269376	4,05e-11
Serine (or cysteine) peptidase inhibitor, clade b, member 2	*Serpinb2*	104,947275	2,37e-10
Ceruloplasmin	*Cp*	104,150741	5,46e-11
Armadillo repeat containing, x-linked 2	*Armcx2*	84,8545918	3,34e-09
Insulin-like growth factor binding protein 5	*Igfbp5*	75,8920762	1,62e-11
Coagulation factor xiii, a1 subunit	*F13a1*	73,0606745	1,14e-09
Atpase, na+/k+ transporting, beta 1 polypeptide	*Atp1b1*	70,8491081	3,44e-10
Paired-like homeodomain transcription factor 2	*Pitx2*	68,7412513	6,17e-10
Angiopoietin 4	*Angpt4*	57,2838178	5,01e-11
Four and a half lim domains 1	*Fhl1*	52,4798963	9,24e-11
Cap, adenylate cyclase-associated protein, 2 (yeast)	*Cap2*	46,1767584	2,06e-10
Cell adhesion molecule 1	*Cadm1*	45,0476616	1,63e-11
Trypsin 4 /// trypsin 5	*Try4 /// try5*	41,3346923	2,05e-10
Regulator of calcineurin 2	*Rcan2*	41,2553498	4,46e-11
Cd28 antigen	*Cd28*	40,6468893	4,03e-08
Very low density lipoprotein receptor	*Vldlr*	38,7069108	9,73e-09
Cysteine-rich c-terminal 1	*Crct1*	37,4506103	2,81e-11
Sry (sex determining region y)-box 11	*Sox11*	35,3978927	5,67e-11
Cholinergic receptor, nicotinic, alpha polypeptide 1	*Chrna1*	35,2366587	1,79e-11

(muscle)			
Acyl-coa thioesterase 1	*Acot1*	34,8703717	1,88e-10
Clusterin	*Clu*	34,5149922	1,63e-11
Reticulon 2 (z-band associated protein)	*Rtn2*	34,2196428	1,67e-09
Chloride channel calcium activated 2	*Clca2*	30,8436168	2,80e-10
Chloride channel calcium activated 1 /// chloride channel calcium activated 2	*Clca1 /// clca2*	30,432735	3,67e-09
Cyclin-dependent kinase inhibitor 2a	*Cdkn2a*	30,4273865	1,23e-09
Tetraspanin 6	*Tspan6*	29,6280931	4,77e-10
Annexin a8	*Anxa8*	29,2637369	4,53e-11
Glutamine repeat protein 1	*Glrp1*	26,9530482	6,80e-11
Synaptotagmin-like 2	*Sytl2*	26,8613638	2,71e-10
Thrombomodulin	*Thbd*	25,3993111	4,83e-08
Cytidine monophospho-n-acetylneuraminic acid hydroxylase	*Cmah*	24,5808017	2,90e-09
Cugbp, elav-like family member 4	*Celf4*	24,5515189	1,88e-10
Cholinergic receptor, nicotinic, beta polypeptide 1 (muscle)	*Chrnb1*	24,5506363	3,63e-10
Musculoskeletal, embryonic nuclear protein 1	*Mustn1*	24,2400543	4,91e-10
Malignant t cell amplified sequence 2	*Mcts2*	23,1971414	1,38e-09
Interleukin 13 receptor, alpha 2	*Il13ra2*	22,5996109	4,71e-09
Transmembrane protein 176a	*Tmem176a*	22,1960788	4,00e-09
Speg complex locus	*Speg*	21,7891441	9,46e-09
Fibroblast growth factor 7	*Fgf7*	21,7320426	8,55e-10
Leucine rich repeat protein 1, neuronal	*Lrrn1*	21,1626076	4,27e-09
Heparan sulfate 6-o-sulfotransferase 2	*Hs6st2*	21,0560197	5,76e-10
Nestin	*Nes*	21,0290075	2,06e-10
Transmembrane protein 176b	*Tmem176b*	20,7454349	2,71e-10
Cystathionase (cystathionine gamma-lyase)	*Cth*	20,4368186	5,01e-08
Insulin-like growth factor binding protein 2	*Igfbp2*	20,2420668	3,07e-09
Atp-binding cassette, sub-family g (white), member 2	*Abcg2*	20,2400042	2,50e-09
Solute carrier family 24 (sodium/potassium/calcium exchanger), member 3	*Slc24a3*	20,1144808	7,47e-09
Receptor-associated protein of the synapse	*Rapsn*	19,9791182	9,24e-11
Kelch-like 13	*Klhl13*	19,8810909	1,20e-09
Integral membrane protein 2a	*Itm2a*	19,4645515	2,18e-07
Guanine deaminase	*Gda*	19,4573961	1,66e-09

Dachshund 2 (drosophila)	*Dach2*	19,1745837	2,32e-07
Epidermal growth factor-containing fibulin-like extracellular matrix protein 1	*Efemp1*	18,8296611	1,06e-09
Trichohyalin	*Tchh*	18,6951815	5,14e-10
Bradykinin receptor, beta 1	*Bdkrb1*	18,5137027	5,01e-11
Ankyrin repeat domain 1 (cardiac muscle)	*Ankrd1*	17,8995751	1,47e-10
Family with sequence similarity 46, member c	*Fam46c*	17,4504879	2,83e-09
Lysophosphatidic acid receptor 4	*Lpar4*	17,224062	1,34e-07
Titin	*Ttn*	16,8926218	1,88e-10
Chloride channel calcium activated 1	*Clca1*	16,6305265	2,98e-09
Atpase, na+/k+ transporting, alpha 2 polypeptide	*Atp1a2*	16,434359	6,22e-08
Collagen, type iv, alpha 1	*Col4a1*	16,412644	1,09e-09
Collagen, type iv, alpha 2	*Col4a2*	16,1873903	2,62e-09
Cadherin 15	*Cdh15*	15,6326906	1,14e-09
Family with sequence similarity 129, member a	*Fam129a*	15,300274	1,19e-08
Selectin, platelet	*Selp*	15,0521292	3,54e-08
Lactate dehydrogenase b	*Ldhb*	15,0094095	1,34e-08
Beta-site app-cleaving enzyme 2	*Bace2*	14,9730405	3,14e-09
Serine (or cysteine) peptidase inhibitor, clade b, member 6b	*Serpinb6b*	14,820259	9,73e-09
Haptoglobin	*Hp*	14,8194664	1,88e-10
Retinoic acid early transcript 1, alpha /// retinoic acid early transcript beta /// retinoic acid early transcript gamma /// retinoic acid early transcript delta /// retinoic acid early transcript 1e	*Raet1a /// raet1b /// raet1c /// raet1d /// raet1e*	14,4447039	9,46e-09
Insulin-like growth factor 1	*Igf1*	13,940747	1,06e-08
Aldo-keto reductase family 1, member c18	*Akr1c18*	13,6754521	1,54e-09
Immunoglobulin superfamily, dcc subclass, member 4	*Igdcc4*	13,6532624	4,25e-10
Chemokine (c-x-c motif) ligand 5	*Cxcl5*	13,283812	2,85e-09
Death-associated protein kinase 2	*Dapk2*	13,2594715	2,06e-10
Neural proliferation, differentiation and control 1	*Npdc1*	13,0635648	5,14e-10
Thrombospondin 1	*Thbs1*	12,8465296	1,48e-08
Interleukin 1 receptor antagonist	*Il1rn*	12,806676	1,08e-09
Nuclear factor, interleukin 3, regulated	*Nfil3*	12,6842313	2,32e-10
Pdz and lim domain 3	*Pdlim3*	12,5886121	1,28e-10
Myelin basic protein	*Mbp*	12,5475217	1,91e-08
Nuclear paraspeckle assembly transcript 1 (non-	*Neat1*	12,5351803	5,28e-08

protein coding)			
Cd302 antigen	*Cd302*	12,3971077	4,98e-09
Autophagy related 9b	*Atg9b*	12,2738403	1,92e-08
Cdc42 effector protein (rho gtpase binding) 3	*Cdc42ep3*	12,2446451	2,23e-09
Cd200 antigen	*Cd200*	12,2046065	9,72e-08
S100 calcium binding protein a8 (calgranulin a)	*S100a8*	12,0700383	4,14e-09
Histocompatibility 2, class ii, locus mb1 /// histocompatibility 2, class ii, locus mb2 /// uncharacterized loc102642102	*H2-dmb1 /// h2-dmb2 /// loc102642102*	11,9846237	3,34e-07
Nerve growth factor receptor (tnfr superfamily, member 16)	*Ngfr*	11,9220499	7,34e-09
Lipocalin 2	*Lcn2*	11,8533772	9,53e-09
Gm2 ganglioside activator protein	*Gm2a*	11,7880585	5,38e-09
Late cornified envelope 1h	*Lce1h*	11,7880263	2,13e-07
Protocadherin 18	*Pcdh18*	11,4467091	4,08e-09
Annexin a3	*Anxa3*	11,4289549	2,69e-09
Acyl-coa thioesterase 1 /// acyl-coa thioesterase 2	*Acot1 /// acot2*	11,2970927	3,89e-09
Serum deprivation response	*Sdpr*	11,236382	1,05e-08
G0/g1 switch gene 2	*G0s2*	11,1398907	4,23e-10
Mesothelin	*Msln*	11,1255229	3,63e-10
Cugbp, elav-like family member 2	*Celf2*	11,1164678	1,19e-08
Starch binding domain 1	*Stbd1*	11,0624749	5,14e-10
Macrophage stimulating 1 (hepatocyte growth factor-like)	*Mst1*	11,0203291	2,80e-10
Popeye domain containing 3	*Popdc3*	10,9878919	7,13e-10
Bai1-associated protein 2-like 1	*Baiap2l1*	10,9837463	2,39e-10
A disintegrin-like and metallopeptidase (reprolysin type) with thrombospondin type 1 motif, 5 (aggrecanase-2)	*Adamts5*	10,9726393	8,33e-10
Synaptotagmin xvii	*Syt17*	10,6265575	4,77e-10
Chemokine (c-x-c motif) ligand 1	*Cxcl1*	10,3416451	1,53e-08
Serine (or cysteine) peptidase inhibitor, clade b, member 1b	*Serpinb1b*	10,339474	3,64e-09
Catenin (cadherin associated protein), alpha-like 1	*Ctnnal1*	10,2965725	1,42e-08
Zinc finger protein 105	*Zfp105*	10,2378927	1,40e-09
Solute carrier family 9 (sodium/hydrogen exchanger), member 3 regulator 1	*Slc9a3r1*	10,0674235	5,42e-07
Ectonucleotide pyrophosphatase/phosphodiesterase	*Enpp5*	10,0577013	3,79e-07

5			
Solute carrier family 43, member 3	*Slc43a3*	9,96545905	6,11e-09
Desmocollin 2	*Dsc2*	9,94883984	4,88e-09
Heat shock protein 2	*Hspb2*	9,82876492	4,14e-09
Connective tissue growth factor	*Ctgf*	9,57441142	4,39e-09
Tribbles homolog 3 (drosophila)	*Trib3*	9,53697454	1,64e-09
Nephronectin	*Npnt*	9,46130568	2,52e-07
Sphingomyelin phosphodiesterase, acid-like 3b	*Smpdl3b*	9,37370116	9,58e-09
Ubiquitin specific peptidase 17-like a	*Usp17la*	9,36072912	9,36e-08
Tripartite motif-containing 25	*Trim25*	9,29259936	1,05e-08
Sh3 domain binding glutamic acid-rich protein like 2	*Sh3bgrl2*	9,0220444	2,69e-09
Annexin a6	*Anxa6*	8,99258429	4,50e-07
Glutathione peroxidase 3	*Gpx3*	8,87433641	2,91e-08
Family with sequence similarity 20, member c	*Fam20c*	8,80749077	3,44e-08
Family with sequence similarity 134, member b	*Fam134b*	8,78932439	1,31e-08
Protein kinase d1	*Prkd1*	8,77336041	4,83e-10
Solute carrier family 25 (mitochondrial carrier; phosphate carrier), member 23	*Slc25a23*	8,69847613	2,30e-06
Myocardial zonula adherens protein	*Myzap*	8,66693724	2,14e-07
Nebulette	*Nebl*	8,61877963	1,12e-06
Serine (or cysteine) peptidase inhibitor, clade b, member 7	*Serpinb7*	8,61561834	1,12e-06
Interleukin 6	*Il6*	8,61330577	4,01e-08
Chemokine (c-x-c motif) ligand 2	*Cxcl2*	8,60423268	1,48e-06
Growth arrest specific 6	*Gas6*	8,59505808	1,50e-07
Cd93 antigen	*Cd93*	8,5757243	8,36e-08
Insulin receptor substrate 1	*Irs1*	8,45853578	4,26e-09
Solute carrier family 16 (monocarboxylic acid transporters), member 1	*Slc16a1*	8,42594253	1,55e-08
Glycerophosphodiester phosphodiesterase domain containing 2	*Gdpd2*	8,3904879	1,23e-09
Phosphatidylinositol-4-phosphate 5-kinase, type 1 beta	*Pip5k1b*	8,37876708	1,02e-09
Suppressor of cytokine signaling 2	*Socs2*	8,33656316	8,28e-08
Glycine receptor, beta subunit	*Glrb*	8,20036137	9,96e-06
Solute carrier family 44, member 2	*Slc44a2*	8,19686952	4,71e-07
Adipocyte-related x-chromosome expressed sequence 1 /// adipocyte-related x-chromosome	*Arxes1 /// arxes2*	8,10228786	1,51e-07

expressed sequence 2			
B cell linker	*Blnk*	7,95303684	2,77e-08
Collagen, type iv, alpha 5	*Col4a5*	7,94933318	3,50e-08
Calcium channel, voltage-dependent, gamma subunit 6	*Cacng6*	7,93096642	1,26e-08
Rwd domain containing 3	*Rwdd3*	7,89589827	3,31e-08
Ccaat/enhancer binding protein (c/ebp), delta	*Cebpd*	7,86146106	6,34e-08
Scavenger receptor class a, member 3	*Scara3*	7,77173329	5,45e-09
Cyclin-dependent kinase 18	*Cdk18*	7,64628151	1,27e-07
Histocompatibility 2, class ii, locus mb1 /// histocompatibility 2, class ii, locus mb2	*H2-dmb1 /// h2-dmb2*	7,60869351	1,30e-06
Cytoplasmic polyadenylation element binding protein 1	*Cpeb1*	7,57315096	2,27e-08
Riken cdna d430019h16 gene	*D430019h16rik*	7,53217179	2,23e-09
Chloride channel calcium activated 4	*Clca4*	7,50820533	5,21e-06
Sorbitol dehydrogenase	*Sord*	7,46151838	1,00e-09
Armadillo repeat containing, x-linked 4	*Armcx4*	7,42627595	2,98e-09
Pentraxin related gene	*Ptx3*	7,35517447	7,04e-08
Argininosuccinate synthetase 1 /// argininosuccinate synthase pseudogene	*Ass1 /// gm5424*	7,24946014	0,00013394
Zinc finger protein, multitype 2	*Zfpm2*	7,23172412	1,38e-09
Laminin, alpha 2	*Lama2*	7,19131726	5,96e-08
Syncoilin	*Sync*	7,14666344	2,97e-09
Serine (or cysteine) peptidase inhibitor, clade b, member 9c	*Serpinb9c*	7,13487199	4,14e-09
4-nitrophenylphosphatase domain and non-neuronal snap25-like protein homolog 1 (c. Elegans)	*Nipsnap1*	7,09989621	9,73e-09
Iroquois related homeobox 3	*Irx3*	7,09889691	2,34e-09
Histocompatibility 2, class ii antigen a, alpha	*H2-aa*	7,07783565	2,30e-07
Cysteine-rich with egf-like domains 1	*Creld1*	7,05459206	2,85e-08
Major facilitator superfamily domain containing 6	*Mfsd6*	7,0328259	2,01e-08
Colony stimulating factor 1 receptor	*Csf1r*	7,02538882	5,37e-09
Frizzled homolog 7 (drosophila)	*Fzd7*	7,00319613	9,39e-08
Adenosine a2b receptor	*Adora2b*	6,87035716	7,77e-09
Tenascin c	*Tnc*	6,83855937	1,31e-07
Leptin receptor	*Lepr*	6,78236926	2,43e-07
Myelin protein zero-like 2	*Mpzl2*	6,78060013	2,60e-08
Thrombospondin 3	*Thbs3*	6,77420917	2,47e-08

Sperm associated antigen 1	*Spag1*	6,76178582	3,57e-06
Transmembrane protein 38b	*Tmem38b*	6,65823654	2,78e-07
Cytochrome b-245, alpha polypeptide	*Cyba*	6,6243535	3,73e-09
Corneodesmosin	*Cdsn*	6,61225033	0,00014979
Proteoglycan 4 (megakaryocyte stimulating factor, articular superficial zone protein)	*Prg4*	6,60577436	1,60e-07
Bh3 interacting domain death agonist	*Bid*	6,60426871	6,11e-08
Frizzled homolog 6 (drosophila)	*Fzd6*	6,5764071	5,16e-08
Crystallin, beta a4	*Cryba4*	6,56556825	9,30e-07
Solute carrier organic anion transporter family, member 1a5	*Slco1a5*	6,55230996	6,42e-09
Phosphodiesterase 2a, cgmp-stimulated	*Pde2a*	6,3984059	2,38e-08
Phosphatidic acid phosphatase type 2 domain containing 3	*Ppapdc3*	6,39715452	7,40e-08
Tubulin, beta 2b class iib	*Tubb2b*	6,30517352	2,58e-08
Carboxymethylenebutenolidase-like (pseudomonas)	*Cmbl*	6,27022941	1,23e-08
A disintegrin-like and metallopeptidase (reprolysin type) with thrombospondin type 1 motif, 4	*Adamts4*	6,22780265	4,09e-09
Arginine/serine rich protein 1	*Rsrp1*	6,18309528	2,27e-08
Tubulin, beta 2a, pseudogene 2 /// tubulin, beta 2b class iib	*Tubb2a-ps2 /// tubb2b*	6,15880646	1,31e-07
Cdna sequence bc002189	*Bc002189*	6,1559937	1,27e-05
Microtubule associated monooxygenase, calponin and lim domain containing 1	*Mical1*	6,14099325	3,69e-07
Sialic acid acetylesterase	*Siae*	6,13351655	1,50e-07
Collagen, type viii, alpha 1	*Col8a1*	6,13261464	4,57e-09
Atpase, na+/k+ transporting, alpha 3 polypeptide	*Atp1a3*	6,13079538	2,26e-05
Spla/ryanodine receptor domain and socs box containing 1	*Spsb1*	6,1148469	1,60e-08
Calponin 1	*Cnn1*	6,10293608	1,15e-08
Homeobox b13	*Hoxb13*	6,05221811	2,48e-08
Family with sequence similarity 213, member a	*Fam213a*	6,05082732	1,71e-06
Nucleic acid binding protein 1	*Nabp1*	6,03694432	1,23e-05
Lysyl oxidase-like 4	*Loxl4*	5,95325429	1,91e-07
Glucosaminyl (n-acetyl) transferase 2, i-branching enzyme	*Gcnt2*	5,92555258	8,62e-08
Troponin i, skeletal, slow 1	*Tnni1*	5,91874753	4,39e-09
Sema domain, immunoglobulin domain (ig), short	*Sema3b*	5,84929325	2,18e-09

basic domain, secreted, (semaphorin) 3b			
Transporter 2, atp-binding cassette, sub-family b (mdr/tap)	*Tap2*	5,84246079	5,23e-08
Nicotinamide nucleotide adenylyltransferase 3	*Nmnat3*	5,8335704	1,50e-07
Cyclin-dependent kinase inhibitor 2b (p15, inhibits cdk4)	*Cdkn2b*	5,82254027	1,19e-06
Riken cdna 2210016f16 gene	*2210016f16rik*	5,80380166	1,54e-09
Oligodendrocyte transcription factor 1	*Olig1*	5,79893517	8,94e-09
Chymotrypsin-like elastase family, member 1	*Cela1*	5,79036335	3,02e-07
Lectin, galactose binding, soluble 3	*Lgals3*	5,78710731	3,25e-09
Collagen, type v, alpha 2	*Col5a2*	5,74609547	4,27e-08
Nebulin-related anchoring protein	*Nrap*	5,73403984	7,82e-09
Ankyrin repeat domain 10	*Ankrd10*	5,72663546	4,74e-08
F-box protein 17	*Fbxo17*	5,6993703	2,77e-07
Frizzled homolog 4 (drosophila)	*Fzd4*	5,67857564	9,36e-08
Matrix metallopeptidase 3	*Mmp3*	5,67235928	1,51e-07
Abhydrolase domain containing 6	*Abhd6*	5,66243903	2,03e-07
Membrane bound o-acyltransferase domain containing 2	*Mboat2*	5,65243602	1,28e-07
Glycoprotein 49 a /// leukocyte immunoglobulin-like receptor, subfamily b, member 4	*Gp49a /// lilrb4*	5,63720139	5,77e-07
Desmin	*Des*	5,6326621	9,73e-08
Signal-regulatory protein alpha	*Sirpa*	5,6119533	6,46e-09
Glucosidase, alpha, acid	*Gaa*	5,53710561	6,92e-09
Paired box 7	*Pax7*	5,52061382	3,88e-06
Cdc42 effector protein (rho gtpase binding) 5	*Cdc42ep5*	5,51930655	6,24e-08
Diaphanous homolog 2 (drosophila)	*Diap2*	5,44676132	5,19e-08
Dipeptidylpeptidase 7	*Dpp7*	5,43888167	1,95e-08
Interleukin 13 receptor, alpha 1	*Il13ra1*	5,42166816	6,39e-07
Odd-skipped related 1 (drosophila)	*Osr1*	5,41156846	2,63e-07
Protein tyrosine phosphatase, receptor type, d	*Ptprd*	5,38522737	1,92e-07
Phosphoprotein associated with glycosphingolipid microdomains 1	*Pag1*	5,37214032	2,42e-08
Lim and cysteine-rich domains 1	*Lmcd1*	5,32703934	1,55e-07
G protein-coupled receptor 155	*Gpr155*	5,32676384	7,47e-08
Junction adhesion molecule 3	*Jam3*	5,27362029	4,91e-07
Trimethyllysine hydroxylase, epsilon	*Tmlhe*	5,26236433	1,92e-07
Enolase 3, beta muscle	*Eno3*	5,25445053	1,00e-08

Transforming growth factor, beta 2	*Tgfb2*	5,2467938	7,09e-06
Methyltransferase like 20	*Mettl20*	5,24389571	4,46e-07
Aldo-keto reductase family 1, member c12 /// aldo-keto reductase family 1, member c13	*Akr1c12 /// akr1c13*	5,24230714	2,40e-07
Kruppel-like factor 2 (lung)	*Klf2*	5,23894391	2,56e-08
Zinc finger protein 37	*Zfp37*	5,22924084	2,02e-07
Absent in melanoma 1	*Aim1*	5,2056271	3,29e-08
Oncostatin m receptor	*Osmr*	5,20494858	5,19e-08
Dep domain containing mtor-interacting protein	*Deptor*	5,20194988	4,46e-07
Wap four-disulfide core domain 2	*Wfdc2*	5,19683973	3,29e-08
Cbp/p300-interacting transactivator, with glu/asp-rich carboxy-terminal domain, 2	*Cited2*	5,1790888	0,00016919
Lysyl oxidase	*Lox*	5,16636401	3,54e-08
Zinc finger protein 422	*Zfp422*	5,13890194	4,42e-07
Nlr family, apoptosis inhibitory protein 2	*Naip2*	5,1355031	5,55e-09
Transcobalamin 2	*Tcn2*	5,08744073	2,16e-08
Phytanoyl-coa hydroxylase	*Phyh*	5,05931391	3,54e-07
Expressed sequence ai464131	*Ai464131*	5,04212891	1,36e-08
Histocompatibility 13	*H13*	5,02698964	1,19e-08
Abhydrolase domain containing 3	*Abhd3*	5,0010698	6,89e-06
Sh3-domain grb2-like 3	*Sh3gl3*	4,98341561	2,97e-06
Coagulation factor iii	*F3*	4,95303317	1,65e-06
Cytochrome b5 reductase 1	*Cyb5r1*	4,94475954	2,40e-09
Coxsackie virus and adenovirus receptor	*Cxadr*	4,94050154	9,21e-07
Serine (or cysteine) peptidase inhibitor, clade b, member 9	*Serpinb9*	4,92746	1,16e-07
Paired box 6	*Pax6*	4,919596	6,02e-07
Ribosomal protein s6 kinase, polypeptide 2	*Rps6ka2*	4,91152128	8,49e-09
Aldo-keto reductase family 1, member c13	*Akr1c13*	4,91011754	1,27e-07
Family with sequence similarity 132, member a	*Fam132a*	4,89962897	7,02e-07
Arrestin domain containing 4	*Arrdc4*	4,89608684	1,97e-07
Slit-robo rho gtpase activating protein 3	*Srgap3*	4,86392163	3,67e-09
Paraoxonase 3	*Pon3*	4,83640802	4,34e-07
Star-related lipid transfer (start) domain containing 5	*Stard5*	4,81921467	2,77e-08
Carbohydrate (n-acetylgalactosamine 4-sulfate 6-o) sulfotransferase 15	*Chst15*	4,81914725	8,04e-07
Nidogen 2	*Nid2*	4,8113096	3,21e-08
Secretory leukocyte peptidase inhibitor	*Slpi*	4,80523642	5,80e-08

Alpha-kinase 2	*Alpk2*	4,79585048	1,51e-07
Avian reticuloendotheliosis viral (v-rel) oncogene related b	*Relb*	4,79003124	8,84e-09
Notch 4	*Notch4*	4,76915719	1,92e-07
Transmembrane protein with egf-like and two follistatin-like domains 1	*Tmeff1*	4,73624182	1,48e-06
Cd59a antigen	*Cd59a*	4,73555908	1,36e-07
Ephrin b2	*Efnb2*	4,73444866	4,98e-09
Pyruvate dehydrogenase kinase, isoenzyme 4	*Pdk4*	4,69310715	1,50e-08
Retinol dehydrogenase 10 (all-trans)	*Rdh10*	4,6572334	8,15e-07
Armadillo repeat containing, x-linked 3	*Armcx3*	4,63489283	3,33e-08
Glutathione s-transferase, alpha 4	*Gsta4*	4,63156367	8,57e-08
Wolfram syndrome 1 homolog (human)	*Wfs1*	4,63122293	1,91e-09
F-box protein 32	*Fbxo32*	4,62714996	1,68e-06
Ras association (ralgds/af-6) domain family member 2	*Rassf2*	4,60934949	7,72e-08
Spry domain containing 7	*Spryd7*	4,58925856	8,51e-07
Epidermal growth factor receptor	*Egfr*	4,58894804	3,19e-07
Integrin alpha 6	*Itga6*	4,57817942	2,53e-07
Nedd4 binding protein 2-like 1	*N4bp2l1*	4,57486826	8,95e-07
Interleukin-1 receptor-associated kinase 1 binding protein 1	*Irak1bp1*	4,56514276	7,33e-06
Solute carrier family 29 (nucleoside transporters), member 1	*Slc29a1*	4,55091759	1,34e-08
Early b cell factor 3	*Ebf3*	4,51436097	4,49e-07
Protocadherin beta 9	*Pcdhb9*	4,50882213	3,71e-05
Brain derived neurotrophic factor	*Bdnf*	4,49506893	4,58e-07
Spermatogenesis associated glutamate (e)-rich protein 4a	*Speer4a*	4,48628169	1,30e-05
Phosphatase domain containing, paladin 1 /// thrombospondin 1	*Pald1 /// thbs1*	4,46977616	1,31e-08
Phosphodiesterase 1a, calmodulin-dependent	*Pde1a*	4,41000871	7,64e-09
Myomesin 2	*Myom2*	4,40803791	1,75e-07
Riken cdna c920025e04 gene /// histocompatibility 2, t region locus 23 /// h-2 class i histocompatibility antigen, d-37 alpha chain-like	*C920025e04rik /// h2-t23 /// loc102641046*	4,37294094	8,34e-08
Paraoxonase 2	*Pon2*	4,35396448	7,95e-06
Transcription factor ap-2, gamma	*Tfap2c*	4,34765705	6,71e-06

Interferon induced transmembrane protein 10	*Ifitm10*	4,34611919	1,40e-07
Histocompatibility 2, k1, k region	*H2-k1*	4,34413063	3,96e-07
Laminin, beta 2	*Lamb2*	4,33883166	6,84e-08
Hepatic leukemia factor	*Hlf*	4,32416788	4,44e-06
Phospholipase c, beta 4	*Plcb4*	4,31898961	4,66e-05
Histocompatibility 2, d region locus 1	*H2-d1*	4,28840606	4,75e-06
Adenylate kinase 3	*Ak3*	4,28115987	5,26e-07
Chac, cation transport regulator 1	*Chac1*	4,27893561	8,23e-09
Aldehyde dehydrogenase 18 family, member a1	*Aldh18a1*	4,2739759	2,02e-06
Secretogranin v	*Scg5*	4,26699148	2,87e-06
Dysbindin (dystrobrevin binding protein 1) domain containing 2	*Dbndd2*	4,25488728	5,33e-07
Stathmin-like 2	*Stmn2*	4,25254338	1,89e-07
Histone cluster 1, h3b /// histone cluster 1, h3c /// histone cluster 1, h3d /// histone cluster 1, h3e /// histone cluster 1, h3f /// histone cluster 2, h3b /// histone cluster 2, h3c1 /// histone cluster 2, h3c2	*Hist1h3b /// hist1h3c /// hist1h3d /// hist1h3e /// hist1h3f /// hist2h3b /// hist2h3c1 /// hist2h3c2*	4,23469253	3,69e-07
Nhs-like 1	*Nhsl1*	4,22979457	1,31e-05
Distal-less homeobox 4	*Dlx4*	4,20102902	3,72e-07
Acyl-coa thioesterase 4	*Acot4*	4,20080419	1,10e-05
Nucleolar protein 3 (apoptosis repressor with card domain)	*Nol3*	4,19311634	3,25e-06
Stanniocalcin 2	*Stc2*	4,1513433	1,21e-07
Gene regulated by estrogen in breast cancer protein	*Greb1*	4,14370896	1,79e-06
Rho gtpase activating protein 12	*Arhgap12*	4,13656083	1,13e-06
Histocompatibility 2, d region locus 1 /// histocompatibility 2, d region locus l	*H2-d1 /// h2-l*	4,13256601	1,22e-07
Nuclear factor of kappa light polypeptide gene enhancer in b cells inhibitor, zeta	*Nfkbiz*	4,12791581	2,16e-07
Lmbr1 domain containing 1	*Lmbrd1*	4,11422426	2,24e-06
Phosphoprotein enriched in astrocytes 15a	*Pea15a*	4,1001268	1,43e-08
Scavenger receptor class a, member 5 (putative)	*Scara5*	4,09568202	1,92e-07
Cysteine rich protein 61	*Cyr61*	4,0854476	0,00021573
Presenilin 2	*Psen2*	4,07580014	3,87e-07
Peroxisomal biogenesis factor 11 alpha	*Pex11a*	4,06667325	3,85e-07
Histone cluster 1, h2bc /// histone cluster 1, h2be ///	*Hist1h2bc ///*	4,06089085	1,51e-08

histone cluster 1, h2bg	*hist1h2be /// hist1h2bg*		
Basonuclin 1	*Bnc1*	4,05812254	1,36e-07
Chitinase domain containing 1	*Chid1*	4,05488187	4,43e-07
Glutathione s-transferase, alpha 2 (yc2)	*Gsta2*	4,04051591	1,22e-06
Regulator of g-protein signaling 16	*Rgs16*	4,01509719	2,96e-07
Sulfide quinone reductase-like (yeast)	*Sqrdl*	4,00820527	3,36e-08
Chromobox 4	*Cbx4*	4,00115982	5,64e-07
Nudix (nucleoside diphosphate linked moiety x)-type motif 7	*Nudt7*	3,99598398	4,29e-07
Rho family gtpase 3	*Rnd3*	3,98660737	1,27e-05
Spermatid perinuclear rna binding protein	*Strbp*	3,98464535	1,99e-05
Inad-like (drosophila)	*Inadl*	3,96495803	5,14e-08
Interferon activated gene 204	*Ifi204*	3,9549975	6,85e-05
Ubiquitin specific peptidase 11	*Usp11*	3,9437928	4,63e-07
Cxxc finger 5	*Cxxc5*	3,94333733	1,02e-07
Integrin alpha 7	*Itga7*	3,91995842	1,48e-08
Junction adhesion molecule 2	*Jam2*	3,91947843	1,63e-06
Schlafen 2	*Slfn2*	3,91420101	1,27e-06
Coronin 6	*Coro6*	3,8999345	2,38e-05
Complement component 3	*C3*	3,8930827	1,23e-07
Protein tyrosine phosphatase, receptor type, k	*Ptprk*	3,89263761	7,82e-09
Plexin d1	*Plxnd1*	3,88784549	3,75e-07
Homeodomain interacting protein kinase 2	*Hipk2*	3,88525545	1,63e-06
Retinoic acid receptor, beta	*Rarb*	3,86674274	2,97e-06
Cathepsin h	*Ctsh*	3,86634296	3,35e-08
Ajuba lim protein	*Ajuba*	3,85323289	2,46e-08
Nadh dehydrogenase (ubiquinone) 1 alpha subcomplex, assembly factor 1	*Ndufaf1*	3,84897563	1,43e-08
Torsin a interacting protein 2	*Tor1aip2*	3,83997571	4,87e-08
Carbonyl reductase 3	*Cbr3*	3,8365699	3,06e-07
Ubiquitin specific peptidase 53	*Usp53*	3,83071714	2,07e-06
Tuftelin 1	*Tuft1*	3,81101508	2,14e-08
Transgelin 3	*Tagln3*	3,80111166	1,86e-07
Midline 2	*Mid2*	3,79354692	7,28e-08
Zinc finger protein of the cerebellum 2	*Zic2*	3,78115133	2,10e-07
Toll-like receptor 2	*Tlr2*	3,77234306	9,90e-08
Bmp and activin membrane-bound inhibitor	*Bambi*	3,76828002	5,24e-05

Tropomyosin 3, gamma	*Tpm3*	3,76316031	3,19e-05
Zinc and ring finger 2	*Znrf2*	3,75124192	2,47e-07
Phosphoenolpyruvate carboxykinase 2 (mitochondrial)	*Pck2*	3,74763885	2,89e-08
Sperm autoantigenic protein 17	*Spa17*	3,7358548	2,53e-06
Forkhead box o1	*Foxo1*	3,73047325	3,30e-05
Cathepsin f	*Ctsf*	3,72617381	1,51e-08
Zinc finger and btb domain containing 18	*Zbtb18*	3,72329806	1,42e-08
Zinc finger protein of the cerebellum 5	*Zic5*	3,7193544	4,81e-07
Immunoglobulin superfamily, member 8	*Igsf8*	3,69573683	1,25e-05
Trna methyltransferase 10c	*Trmt10c*	3,69197755	2,62e-06
Ferm domain containing 4b	*Frmd4b*	3,6916088	2,90e-05
Xin actin-binding repeat containing 1	*Xirp1*	3,67610474	6,63e-05
Guanine nucleotide binding protein (g protein), beta 5	*Gnb5*	3,67463917	1,28e-07
S100 calcium binding protein a16	*S100a16*	3,66152551	8,49e-09
Protocadherin 1	*Pcdh1*	3,65714613	7,45e-06
Serglycin	*Srgn*	3,65184969	3,60e-06
Arsenic (+3 oxidation state) methyltransferase	*As3mt*	3,64124535	1,32e-06
Atp-binding cassette, sub-family a (abc1), member 1	*Abca1*	3,63463152	2,00e-05
Zinc finger and btb domain containing 46	*Zbtb46*	3,62069651	2,87e-08
Solute carrier family 6 (neurotransmitter transporter, glycine), member 9	*Slc6a9*	3,61969718	8,36e-08
Disabled 2, mitogen-responsive phosphoprotein	*Dab2*	3,60856986	1,48e-06
Ferredoxin 1-like /// raver1-fdx1l readthrough	*Fdx1l /// raver1-fdx1l*	3,60571795	1,01e-07
Expressed sequence aw209491	*Aw209491*	3,60076532	1,23e-07
Tetraspanin 7	*Tspan7*	3,58309406	1,32e-06
Dtw domain containing 1	*Dtwd1*	3,58062492	2,32e-07
Estrogen related receptor, beta	*Esrrb*	3,58025051	6,64e-06
Rab27b, member ras oncogene family	*Rab27b*	3,56939698	3,28e-06
Rar-related orphan receptor gamma	*Rorc*	3,56921269	3,34e-06
Peroxisomal biogenesis factor 13	*Pex13*	3,56827028	7,42e-05
Frizzled homolog 8 (drosophila)	*Fzd8*	3,56578204	1,40e-07
Interleukin 1 receptor accessory protein	*Il1rap*	3,55698089	1,57e-05
Selenium binding protein 1	*Selenbp1*	3,55530538	4,18e-06
Paternally expressed 12	*Peg12*	3,53526339	1,19e-08
Complement component 1, s subcomponent 1	*C1s1*	3,53413627	1,24e-06
Dnaj (hsp40) homolog, subfamily b, member 9	*Dnajb9*	3,51609552	7,98e-06

Lysm, putative peptidoglycan-binding, domain containing 2	*Lysmd2*	3,51595981	8,80e-07
Solute carrier family 40 (iron-regulated transporter), member 1	*Slc40a1*	3,51111637	1,39e-06
Receptor (tnfrsf)-interacting serine-threonine kinase 2	*Ripk2*	3,50395711	6,32e-08
3-hydroxybutyrate dehydrogenase, type 1	*Bdh1*	3,49697997	1,07e-07
Protein o-glucosyltransferase 1	*Poglut1*	3,4962652	2,74e-08
Solute carrier family 6 (neurotransmitter transporter, betaine/gaba), member 12	*Slc6a12*	3,48752769	3,85e-07
Hemopoietic cell kinase	*Hck*	3,4863987	3,67e-08
Glucan (1,4-alpha-), branching enzyme 1	*Gbe1*	3,48384681	0,00024892
Brain expressed myelocytomatosis oncogene	*Bmyc*	3,47744879	1,31e-06
Kelch-like 24	*Klhl24*	3,47479011	2,57e-05
Interferon regulatory factor 9	*Irf9*	3,47260074	1,25e-07
Solute carrier family 27 (fatty acid transporter), member 1	*Slc27a1*	3,46881447	4,47e-08
Pleckstrin homology-like domain, family b, member 2	*Phldb2*	3,44361414	2,44e-07
Dna segment, chr 1, erato doi 622, expressed	*D1ertd622e*	3,43690909	1,62e-06
Leucine rich repeat containing 16a	*Lrrc16a*	3,43521192	6,09e-07
Poly(rc) binding protein 4	*Pcbp4*	3,43118532	3,97e-05
Cardiotrophin-like cytokine factor 1	*Clcf1*	3,42953876	9,53e-07
Toll-like receptor 3	*Tlr3*	3,42741558	2,19e-06
Cyclin-dependent kinase 14	*Cdk14*	3,4221883	1,53e-06
Neurofilament, medium polypeptide	*Nefm*	3,42118372	4,90e-07
Retinol dehydrogenase 5	*Rdh5*	3,41988669	2,04e-07
Glutamic pyruvate transaminase (alanine aminotransferase) 2	*Gpt2*	3,40851839	8,75e-07
Paired box 8	*Pax8*	3,40809625	2,51e-07
Mutl homolog 3 (e coli)	*Mlh3*	3,39951155	2,28e-07
Low density lipoprotein receptor-related protein 4	*Lrp4*	3,3801029	2,36e-07
Adp-ribosylation factor-like 4c	*Arl4c*	3,37791191	1,53e-08
Peptidylprolyl isomerase c	*Ppic*	3,37605874	8,34e-08
Histocompatibility 2, d region locus 1 /// histocompatibility 2, k1, k region /// h-2 class i histocompatibility antigen, k-d alpha chain-like	*H2-d1 /// h2-k1 /// loc101056305*	3,37526569	4,53e-05
Solute carrier family 25, member 33	*Slc25a33*	3,36540332	1,28e-07

6-phosphofructo-2-kinase/fructose-2,6-biphosphatase 3	*Pfkfb3*	3,36473325	4,20e-06
Phospholipid scramblase 2	*Plscr2*	3,36370998	8,34e-08
Sarcoglycan, beta (dystrophin-associated glycoprotein)	*Sgcb*	3,35402339	9,81e-07
Coiled-coil domain containing 80	*Ccdc80*	3,35342464	1,92e-07
Par-6 family cell polarity regulator beta	*Pard6b*	3,35262801	1,15e-06
Cd68 antigen	*Cd68*	3,34380819	3,37e-07
Prostate transmembrane protein, androgen induced 1	*Pmepa1*	3,33377725	3,99e-07
Morn repeat containing 4	*Morn4*	3,32430948	1,45e-06
Adp-ribosylation factor-like 14 effector protein	*Arl14ep*	3,31475996	7,87e-07
Riken cdna 1700017b05 gene	*1700017b05rik*	3,30968781	2,05e-07
Lipoyltransferase 1	*Lipt1*	3,29863705	7,56e-08
Carbonic anhydrase 5b, mitochondrial	*Car5b*	3,2891462	9,35e-06
Family with sequence similarity 195, member a	*Fam195a*	3,28491931	3,98e-07
Coproporphyrinogen oxidase	*Cpox*	3,28234478	5,31e-05
Ribonuclease p 40 subunit	*Rpp40*	3,28102679	4,71e-07
Atpase, aminophospholipid transporter (aplt), class i, type 8a, member 1	*Atp8a1*	3,27982541	3,99e-06
Sacsin	*Sacs*	3,27851267	3,65e-07
Riken cdna 6720475j19 gene	*6720475j19rik*	3,27814744	3,99e-08
B cell leukemia/lymphoma 3	*Bcl3*	3,26734691	6,48e-06
Choline kinase alpha	*Chka*	3,26069212	9,30e-07
Glutamate receptor, ionotropic, ampa1 (alpha 1)	*Gria1*	3,25935898	1,29e-05
P21 protein (cdc42/rac)-activated kinase 3	*Pak3*	3,25869648	3,22e-07
Galactosidase, beta 1	*Glb1*	3,25512601	4,42e-07
Interferon activated gene 202b	*Ifi202b*	3,25221434	2,04e-07
Muscle, skeletal, receptor tyrosine kinase	*Musk*	3,25182016	1,74e-07
Solute carrier family 1 (glutamate/neutral amino acid transporter), member 4	*Slc1a4*	3,25024087	5,31e-05
Nerve growth factor	*Ngf*	3,23570912	0,00387026
St6 (alpha-n-acetyl-neuraminyl-2,3-beta-galactosyl-1,3)-n-acetylgalactosaminide alpha-2,6-sialyltransferase 4	*St6galnac4*	3,23470962	5,93e-07
Gamma-glutamyl hydrolase	*Ggh*	3,23452823	3,34e-06
Sphingosine kinase 1	*Sphk1*	3,21869185	1,33e-06
Microsomal triglyceride transfer protein	*Mttp*	3,21433892	2,04e-06

Enoyl coenzyme a hydratase domain containing 3	*Echdc3*	3,21331464	1,48e-06
Thiosulfate sulfurtransferase, mitochondrial	*Tst*	3,20709517	0,00011906
Ribosomal protein l22	*Rpl22*	3,20510921	8,34e-08
Myosin, light chain 10, regulatory	*Myl10*	3,20213635	5,63e-06
Branched chain ketoacid dehydrogenase e1, beta polypeptide	*Bckdhb*	3,20046427	1,35e-07
Amyloid beta (a4) precursor protein	*App*	3,19301325	5,30e-07
Macro domain containing 1	*Macrod1*	3,19032634	4,67e-07
Wd repeat domain 77	*Wdr77*	3,1896369	8,78e-07
Ribonuclease t2a /// ribonuclease t2b	*Rnaset2a /// rnaset2b*	3,1895074	9,07e-07
Glycoprotein m6b	*Gpm6b*	3,18354114	4,73e-05
Plasminogen activator, tissue	*Plat*	3,18163526	3,99e-06
Riken cdna 1190002n15 gene	*1190002n15rik*	3,16957775	4,73e-06
Endothelin converting enzyme 2	*Ece2*	3,16159792	5,19e-08
Muscle-related coiled-coil protein	*Murc*	3,16051535	0,0001424
Activating transcription factor 6	*Atf6*	3,15634079	1,64e-07
Mterf domain containing 2	*Mterfd2*	3,15041819	2,01e-07
Quaking	*Qk*	3,14762195	9,25e-05
Dedicator of cytokinesis 5	*Dock5*	3,14562283	8,81e-08
Scleraxis	*Scx*	3,1423088	2,64e-06
Glucuronyl c5-epimerase	*Glce*	3,14180309	8,81e-08
Guanine nucleotide binding protein (g protein), gamma 2	*Gng2*	3,13827653	2,40e-05
Threonine synthase-like 1 (bacterial)	*Thnsl1*	3,13727069	9,71e-06
Late cornified envelope 1i	*Lce1i*	3,13717994	4,01e-05
Tripartite motif-containing 30a	*Trim30a*	3,13158889	6,44e-06
N-acylethanolamine acid amidase	*Naaa*	3,11691892	5,16e-07
Riken cdna 4930529m08 gene	*4930529m08rik*	3,11522518	0,00055958
Cathepsin b	*Ctsb*	3,11261178	4,86e-07
Aldehyde dehydrogenase family 6, subfamily a1	*Aldh6a1*	3,10431153	7,27e-06
Prolactin family 7, subfamily c, member 1	*Prl7c1*	3,10278465	6,32e-07
Translocation associated membrane protein 1-like 1	*Tram1l1*	3,10160163	4,92e-05
O-linked n-acetylglucosamine (glcnac) transferase (udp-n-acetylglucosamine:polypeptide-n-acetylglucosaminyl transferase)	*Ogt*	3,08817319	0,00027753
A disintegrin-like and metallopeptidase (reprolysin type) with thrombospondin type 1 motif, 1	*Adamts1*	3,08706911	3,08e-06
Ectonucleotide pyrophosphatase/phosphodiesterase	*Enpp2*	3,08578802	5,41e-06

2			
Chloride channel 2	*Clcn2*	3,08466751	1,69e-05
Ring finger protein 149	*Rnf149*	3,08443128	8,95e-07
Sel-1 suppressor of lin-12-like (c. Elegans)	*Sel1l*	3,08351488	5,58e-07
Eh-domain containing 4	*Ehd4*	3,07214324	3,44e-08
Interferon gamma inducible protein 30	*Ifi30*	3,05624769	6,46e-08
Acyl-coa thioesterase 2	*Acot2*	3,05497296	1,38e-07
Tsc22 domain family, member 3	*Tsc22d3*	3,05364514	3,53e-06
Riken cdna 1110012l19 gene	*1110012l19rik*	3,05164766	3,89e-07
Coagulation factor ii (thrombin) receptor	*F2r*	3,04856525	9,21e-07
Deiodinase, iodothyronine, type ii	*Dio2*	3,04842407	1,03e-06
Paired box 3	*Pax3*	3,04334969	7,35e-05
Heat shock protein family b (small), member 11	*Hspb11*	3,02304472	4,85e-07
Protein tyrosine phosphatase, non-receptor type 2	*Ptpn2*	3,02139087	1,20e-05
Stt3, subunit of the oligosaccharyltransferase complex, homolog a (s. Cerevisiae)	*Stt3a*	3,01966253	6,41e-05
Cysteine rich protein 2	*Crip2*	3,00898743	5,94e-06
Mannosidase 2, alpha b2	*Man2b2*	3,00820029	3,31e-05
Ccr4 carbon catabolite repression 4-like (s. Cerevisiae)	*Ccrn4l*	3,00491129	8,75e-07
Gamma-glutamyl cyclotransferase	*Ggct*	3,00165539	6,32e-07
G patch domain containing 4	*Gpatch4*	2,9957431	4,25e-06
Calpain 5	*Capn5*	2,99537549	8,33e-07
Selenoprotein p, plasma, 1	*Sepp1*	2,99368752	2,98e-07
Sortilin 1	*Sort1*	2,98887141	3,98e-06
Metastasis associated lung adenocarcinoma transcript 1 (non-coding rna)	*Malat1*	2,98682629	0,000981
Family with sequence similarity 32, member a	*Fam32a*	2,98434992	9,40e-06
Napsin a aspartic peptidase	*Napsa*	2,97450165	1,38e-05
Tsr2 20s rrna accumulation	*Tsr2*	2,97349982	2,01e-07
Prune homolog (drosophila)	*Prune*	2,95923225	0,00024039
Retinoblastoma binding protein 6	*Rbbp6*	2,94665756	6,50e-06
Rho/rac guanine nucleotide exchange factor (gef) 2	*Arhgef2*	2,94625288	1,93e-06
D-tyrosyl-trna deacylase 1	*Dtd1*	2,94526632	6,87e-08
Kruppel-like factor 9	*Klf9*	2,94029177	2,72e-07
Glutathione s-transferase, mu 5	*Gstm5*	2,9377689	3,89e-07
Myosin viia	*Myo7a*	2,927515	1,49e-06
Methylmalonyl coa epimerase	*Mcee*	2,92656715	1,18e-06

Secernin 3	*Scrn3*	2,92482854	2,83e-05
Cdna sequence bc022960	*Bc022960*	2,92298735	7,20e-05
Prkc, apoptosis, wt1, regulator	*Pawr*	2,9215201	2,99e-05
Ddb1 and cul4 associated factor 8	*Dcaf8*	2,92061564	4,42e-07
3-hydroxyisobutyryl-coenzyme a hydrolase	*Hibch*	2,91022766	0,0002902
Predicted gene 3776 /// glutathione s-transferase, alpha 1 (ya) /// glutathione s-transferase, alpha 2 (yc2)	*Gm3776 /// gsta1 /// gsta2*	2,9040713	2,42e-06
Gap junction protein, beta 3	*Gjb3*	2,89798803	6,46e-06
Phosphoserine phosphatase	*Psph*	2,89588686	7,89e-08
Signal transducer and activator of transcription 5a	*Stat5a*	2,89401742	0,00028721
Platelet-derived growth factor, c polypeptide	*Pdgfc*	2,88829067	6,33e-07
Ependymin related protein 1 (zebrafish)	*Epdr1*	2,88230431	0,00041264
Glucocorticoid induced transcript 1	*Glcci1*	2,8798497	9,64e-07
Cubilin (intrinsic factor-cobalamin receptor)	*Cubn*	2,87973839	9,74e-07
Phosphatase, orphan 2	*Phospho2*	2,8785443	7,75e-06
Oxidative stress induced growth inhibitor family member 2	*Osgin2*	2,87313337	8,70e-07
Chondroitin polymerizing factor	*Chpf*	2,87038715	9,19e-07
Spo11 meiotic protein covalently bound to dsb homolog (s. Cerevisiae)	*Spo11*	2,86837947	0,00025069
Acyl-coa thioesterase 6	*Acot6*	2,86669295	2,18e-07
Acyl-coa synthetase bubblegum family member 1	*Acsbg1*	2,86445981	1,72e-06
Ribosomal protein s6	*Rps6*	2,85667982	2,74e-07
Neural cell adhesion molecule 1	*Ncam1*	2,85304324	2,59e-05
Thyroid hormone receptor interactor 12	*Trip12*	2,84867324	4,12e-07
Influenza virus ns1a binding protein	*Ivns1abp*	2,84852316	8,35e-06
Growth differentiation factor 15	*Gdf15*	2,84555765	4,27e-07
Mannosidase, beta a, lysosomal	*Manba*	2,84355119	0,00012483
Cugbp, elav-like family member 1	*Celf1*	2,83782906	6,26e-07
Phosphoribosyl pyrophosphate synthetase 1-like 3	*Prps1l3*	2,83450732	1,30e-06
Hairy/enhancer-of-split related with yrpw motif 1	*Hey1*	2,8328213	3,87e-07
Predicted gene 1976	*Gm1976*	2,82802051	1,78e-05
Annexin a4	*Anxa4*	2,8270422	9,97e-07
Fidgetin	*Fign*	2,81549148	1,75e-05
Prostaglandin reductase 1	*Ptgr1*	2,8089211	7,82e-07
Fatty acid binding protein 5, epidermal	*Fabp5*	2,80888333	8,97e-07
Blood vessel epicardial substance	*Bves*	2,80513657	9,53e-06

Cytochrome p450, family 4, subfamily v, polypeptide 3	*Cyp4v3*	2,80172871	4,48e-06
Zinc finger protein 62	*Zfp62*	2,80060101	2,68e-05
4-aminobutyrate aminotransferase	*Abat*	2,7956161	6,89e-06
Histocompatibility 2, m region locus 3	*H2-m3*	2,79365066	2,87e-05
Pyrroline-5-carboxylate reductase 1	*Pycr1*	2,7901965	1,38e-06
Ubx domain protein 4	*Ubxn4*	2,78880095	1,73e-06
B cell cll/lymphoma 9	*Bcl9*	2,7834666	0,00100405
Rab9 effector protein with kelch motifs	*Rabepk*	2,78280705	2,22e-06
Acyl-coenzyme a dehydrogenase family, member 11	*Acad11*	2,78222739	1,99e-05
Run and sh3 domain containing 1	*Rusc1*	2,78082112	0,00015667
Polo-like kinase 2	*Plk2*	2,77787331	2,16e-07
Dna topoisomerase 1, mitochondrial	*Top1mt*	2,77736473	2,12e-06
Paroxysmal nonkinesiogenic dyskinesia	*Pnkd*	2,77713557	1,54e-05
Dna segment, chr 16, erato doi 472, expressed	*D16ertd472e*	2,77557691	1,92e-06
Forkhead box c1	*Foxc1*	2,77400899	5,30e-07
Carbonic anhydrase 6	*Car6*	2,77231373	1,38e-05
Glutamate-rich wd repeat containing 1	*Grwd1*	2,76760665	7,60e-07
Vesicle-associated membrane protein 4	*Vamp4*	2,7666147	3,00e-06
Cd99 antigen-like 2	*Cd99l2*	2,76453261	6,06e-07
Opioid growth factor receptor-like 1	*Ogfrl1*	2,75940426	1,26e-05
Start domain containing 10	*Stard10*	2,7592835	2,34e-06
Wd repeat containing planar cell polarity effector	*Wdpcp*	2,75369433	9,20e-06
Tumor protein d52-like 1	*Tpd52l1*	2,74959564	5,05e-07
G protein-coupled receptor 137b	*Gpr137b*	2,74907096	3,66e-07
Apolipoprotein l 9a /// apolipoprotein l 9b	*Apol9a /// apol9b*	2,74333374	2,27e-06
2-4-dienoyl-coenzyme a reductase 2, peroxisomal	*Decr2*	2,7424251	3,75e-06
Solute carrier family 12, member 7	*Slc12a7*	2,73339258	5,10e-07
Transmembrane protein 132a	*Tmem132a*	2,73319489	1,59e-06
Actin related protein 2/3 complex, subunit 5-like	*Arpc5l*	2,7296185	3,46e-07
Citrate lyase beta like	*Clybl*	2,72684514	7,24e-05
Solute carrier family 16 (monocarboxylic acid transporters), member 2	*Slc16a2*	2,72407292	2,27e-05
Dipeptidylpeptidase 6	*Dpp6*	2,72398818	1,59e-05
Immunity-related gtpase family m member 2	*Irgm2*	2,71880953	1,35e-05
Queuine trna-ribosyltransferase domain containing 1	*Qtrtd1*	2,70863427	0,00807268
Sema domain, immunoglobulin domain (ig), tm domain, and short cytoplasmic domain	*Sema4f*	2,70567103	3,56e-05

Allograft inflammatory factor 1-like	*Aif1l*	2,70554214	1,55e-05
Phospholipase d1	*Pld1*	2,70270471	2,66e-07
Aldehyde dehydrogenase family 1, subfamily a3	*Aldh1a3*	2,70249863	1,40e-06
Seryl-aminoacyl-trna synthetase	*Sars*	2,70017508	2,53e-06
Galactokinase 2	*Galk2*	2,69731801	6,12e-05
Histone cluster 1, h2bc /// histone cluster 1, h2be /// histone cluster 1, h2bf /// histone cluster 1, h2bg /// histone cluster 1, h2bj /// histone cluster 1, h2bl /// histone cluster 1, h2bm /// histone cluster 1, h2bn /// histone cluster 1, h2bp /// histone cluster 1, h2bq /// histone cluster 1 h2br	*Hist1h2bc /// hist1h2be /// hist1h2bf /// hist1h2bg /// hist1h2bj /// hist1h2bl /// hist1h2bm /// hist1h2bn /// hist1h2bp /// hist1h2bq /// hist1h2br*	2,69586172	1,34e-07
Zinc and ring finger 1	*Znrf1*	2,69286164	2,67e-05
Cathepsin z	*Ctsz*	2,68907963	1,70e-07
Zinc finger protein 566	*Zfp566*	2,68801779	1,81e-06
Melanoma cell adhesion molecule	*Mcam*	2,68757359	3,63e-05
Yippee-like 3 (drosophila)	*Ypel3*	2,68535863	1,64e-06
3-phosphoglycerate dehydrogenase pseudogene /// 3-phosphoglycerate dehydrogenase	*Gm8096 /// phgdh*	2,68197111	8,42e-08
Activating transcription factor 3	*Atf3*	2,68127776	9,33e-07
Methyltransferase like 22	*Mettl22*	2,67791009	4,36e-06
Follistatin	*Fst*	2,67682158	5,07e-07
Solute carrier family 35, member f5	*Slc35f5*	2,67427956	2,34e-07
Aryl-hydrocarbon receptor	*Ahr*	2,67424666	6,29e-05
Four jointed box 1 (drosophila)	*Fjx1*	2,67364993	9,17e-05
Inhibitor of kappab kinase epsilon	*Ikbke*	2,67364459	7,57e-06
Suppressor of cytokine signaling 6	*Socs6*	2,67266604	0,00015311
Riken cdna 1110058l19 gene	*1110058l19rik*	2,66836568	8,64e-07
Single-stranded dna binding protein 2	*Ssbp2*	2,66739218	2,36e-07
Peroxisome proliferative activated receptor, gamma, coactivator 1 beta	*Ppargc1b*	2,65839542	5,16e-07
Cyclin h	*Ccnh*	2,65617129	4,17e-06
Protein phosphatase 1, regulatory (inhibitor) subunit 15a	*Ppp1r15a*	2,65163814	9,16e-07

Riken cdna 1700019g17 gene	*1700019g17rik*	2,64502272	6,44e-06
Nucleobindin 2	*Nucb2*	2,6427909	1,76e-06
Latent transforming growth factor beta binding protein 3	*Ltbp3*	2,63906687	6,24e-07
Necdin-like 2	*Ndnl2*	2,63886079	6,01e-07
Predicted gene 13139	*Gm13139*	2,63826919	1,00e-06
Calcium channel, voltage-dependent, p/q type, alpha 1a subunit	*Cacna1a*	2,63776007	2,35e-06
Tryptophanyl-trna synthetase	*Wars*	2,63764892	6,37e-06
Rab20, member ras oncogene family	*Rab20*	2,63635187	9,36e-06
Fibrillin 1	*Fbn1*	2,62876191	8,52e-06
Sclerostin domain containing 1	*Sostdc1*	2,62779123	1,06e-05
Pseudouridine synthase 3	*Pus3*	2,62371375	1,55e-05
Pleckstrin	*Plek*	2,61955615	0,00106339
Fibulin 2	*Fbln2*	2,61924348	1,28e-07
Nadh dehydrogenase (ubiquinone) 1 alpha subcomplex, assembly factor 4	*Ndufaf4*	2,61487042	1,07e-06
Swi/snf related, matrix associated, actin dependent regulator of chromatin, subfamily d, member 3	*Smarcd3*	2,61432352	2,04e-06
Integrin beta 5	*Itgb5*	2,61365332	8,89e-07
Udp-glucose ceramide glucosyltransferase	*Ugcg*	2,61091338	3,37e-06
Serine (or cysteine) peptidase inhibitor, clade e, member 1	*Serpine1*	2,60592534	2,69e-07
Peroxisomal trans-2-enoyl-coa reductase	*Pecr*	2,6056587	1,49e-06
Tetratricopeptide repeat domain 30b	*Ttc30b*	2,60140648	0,00196444
Transmembrane protein 121	*Tmem121*	2,59640051	1,46e-06
Leukemia inhibitory factor	*Lif*	2,59404635	1,51e-05
Multivesicular body subunit 12b	*Mvb12b*	2,59105499	2,42e-07
Phospholipase d family, member 3	*Pld3*	2,58425815	8,03e-07
Nemo like kinase	*Nlk*	2,58170589	2,10e-06
H19, imprinted maternally expressed transcript /// microrna 675	*H19 /// mir675*	2,57831655	5,77e-06
Rhomboid domain containing 1	*Rhbdd1*	2,57751687	2,24e-07
Cytochrome c oxidase subunit via polypeptide 2	*Cox6a2*	2,57697677	6,08e-06
Thioesterase superfamily member 4	*Them4*	2,57576429	1,75e-05
Ubia prenyltransferase domain containing 1	*Ubiad1*	2,57349797	1,49e-06
3-phosphoglycerate dehydrogenase	*Phgdh*	2,5734408	1,89e-07
Fyn proto-oncogene	*Fyn*	2,5679522	2,78e-06

Dead (asp-glu-ala-asp) box polypeptide 18	*Ddx18*	2,56475856	2,72e-05
Transmembrane protein 123	*Tmem123*	2,56464201	2,72e-06
Creb3 regulatory factor	*Crebrf*	2,55920941	5,16e-07
Keratin 80	*Krt80*	2,55911558	5,66e-06
Ras p21 protein activator 4	*Rasa4*	2,55499509	5,55e-07
Aldo-keto reductase family 1, member e1	*Akr1e1*	2,55447856	3,64e-06
Melanoma antigen, family a, 1 /// melanoma antigen, family a, 2 /// melanoma antigen, family a, 3 /// melanoma antigen, family a, 5 /// melanoma antigen, family a, 6 /// melanoma antigen, family a, 8	*Magea1 /// magea2 /// magea3 /// magea5 /// magea6 /// magea8*	2,55242949	5,21e-05
Interferon gamma receptor 2	*Ifngr2*	2,5477434	7,09e-07
Purinergic receptor p2x, ligand-gated ion channel 4	*P2rx4*	2,54548995	0,00036758
Taxilin gamma	*Txlng*	2,54199559	0,00026162
Tensin 1	*Tns1*	2,54043416	9,34e-06
Galactosidase, alpha	*Gla*	2,53854266	1,40e-07
Coronin, actin binding protein, 2b	*Coro2b*	2,53728245	0,00045757
Fucosidase, alpha-l- 2, plasma	*Fuca2*	2,53503139	5,49e-07
N-acetyltransferase 2 (arylamine n-acetyltransferase)	*Nat2*	2,53305264	0,00034849
Sh3-domain binding protein 5 (btk-associated)	*Sh3bp5*	2,5327384	3,20e-05
Chaperonin containing tcp1, subunit 4 (delta)	*Cct4*	2,53266596	0,00016874
Pleckstrin homology domain containing, family o member 1	*Plekho1*	2,53000979	8,90e-06
Guanylate binding protein 2	*Gbp2*	2,5256308	8,20e-05
Tea domain family member 2	*Tead2*	2,52465518	2,77e-06
Dipeptidylpeptidase 8	*Dpp8*	2,52216309	0,00293086
Serine/threonine kinase 39	*Stk39*	2,52053006	2,38e-05
Glucosidase, beta, acid	*Gba*	2,51972857	1,37e-06
Ras association (ralgds/af-6) domain family member 5	*Rassf5*	2,51801535	3,22e-05
Fk506 binding protein-like	*Fkbpl*	2,51021301	9,56e-07
Histone cluster 1, h1c	*Hist1h1c*	2,50989039	8,29e-05
Placental growth factor	*Pgf*	2,50915782	2,25e-05
Steap family member 4	*Steap4*	2,50753277	1,20e-06
Tumor protein d52	*Tpd52*	2,50684123	1,96e-07
Prostaglandin f receptor	*Ptgfr*	2,50664028	2,55e-05
Ero1-like (s. Cerevisiae)	*Ero1l*	2,50650093	2,36e-07
Insulin-like growth factor binding protein 3	*Igfbp3*	2,50432685	7,46e-06
Lim domain only 1	*Lmo1*	2,50423054	1,24e-06

G protein-coupled receptor associated sorting protein 1	*Gprasp1*	2,50235514	1,07e-06
Caspase 7	*Casp7*	2,50200962	1,56e-06
Ccaat/enhancer binding protein (c/ebp), beta	*Cebpb*	2,49886413	4,10e-05
Rho guanine nucleotide exchange factor (gef) 5	*Arhgef5*	2,4985319	2,36e-06
Host cell factor c1 regulator 1 (xpo1-dependent)	*Hcfc1r1*	2,49694206	2,72e-06
Regulator of g-protein signaling 4	*Rgs4*	2,495675	8,62e-07
Nuclear factor, erythroid derived 2, like 2	*Nfe2l2*	2,49393364	3,10e-05
Synaptosomal-associated protein, 47	*Snap47*	2,48950369	9,53e-07
Isovaleryl coenzyme a dehydrogenase	*Ivd*	2,48946452	2,91e-06
Mannan-binding lectin serine peptidase 1	*Masp1*	2,48345524	7,00e-05
Solute carrier family 25, member 37	*Slc25a37*	2,48302841	1,79e-06
Ubiquitin-conjugating enzyme e2d 3	*Ube2d3*	2,48164883	1,36e-05
Transmembrane protein 53	*Tmem53*	2,47875979	9,66e-07
Podocalyxin-like	*Podxl*	2,47494918	2,56e-05
Ribosomal protein l5 /// ribisomal protein l5, pseudogene 2	*Rpl5 /// rpl5-ps2*	2,47411582	5,04e-07
Acyl-coenzyme a dehydrogenase family, member 10	*Acad10*	2,46945088	2,32e-07
Phospholipase a2, group xv	*Pla2g15*	2,4682578	3,88e-07
Fast kinase domains 1	*Fastkd1*	2,46604312	7,69e-06
Zinc finger protein 639	*Zfp639*	2,46302251	1,07e-07
Chitobiase, di-n-acetyl-	*Ctbs*	2,46192255	7,82e-05
Beta-1,4-n-acetyl-galactosaminyl transferase 1	*B4galnt1*	2,46156038	4,10e-06
Nicotinamide n-methyltransferase	*Nnmt*	2,45930379	5,66e-06
Otu domain, ubiquitin aldehyde binding 2	*Otub2*	2,45912031	4,65e-06
Myozenin 2	*Myoz2*	2,45585984	1,04e-06
Heparan sulfate 6-o-sulfotransferase 1	*Hs6st1*	2,45253686	0,0003961
Alcohol dehydrogenase, iron containing, 1	*Adhfe1*	2,44956172	9,75e-06
Guanine nucleotide binding protein, alpha o	*Gnao1*	2,44852583	0,00016926
Tgfb-induced factor homeobox 1	*Tgif1*	2,44721353	1,52e-06
Unc-13 homolog b (c. Elegans)	*Unc13b*	2,44357062	5,24e-05
S100 calcium binding protein a1	*S100a1*	2,44193103	5,27e-05
Radical s-adenosyl methionine domain containing 2	*Rsad2*	2,44098351	8,49e-05
Branched chain aminotransferase 2, mitochondrial	*Bcat2*	2,43711922	3,06e-06
Ski-like	*Skil*	2,43696615	2,34e-07
Guanylate binding protein 3	*Gbp3*	2,43431924	1,31e-05
Clock interacting protein, circadian	*Cipc*	2,43424439	2,67e-07
Splicing factor 3b, subunit 1	*Sf3b1*	2,43247355	2,81e-05

Unc-119 homolog (c. Elegans)	*Unc119*	2,42696093	1,28e-06
Dapper homolog 1, antagonist of beta-catenin (xenopus)	*Dact1*	2,42611057	1,27e-05
Deoxynucleotidyltransferase, terminal, interacting protein 2	*Dnttip2*	2,42528222	2,18e-05
Poly (a) polymerase alpha	*Papola*	2,42438734	1,01e-05
5-hydroxytryptamine (serotonin) receptor 2b	*Htr2b*	2,42340699	1,95e-05
Brca1/brca2-containing complex, subunit 3	*Brcc3*	2,42220081	0,00193485
Sh3-domain grb2-like b1 (endophilin)	*Sh3glb1*	2,42169348	3,58e-05
Wd repeat domain 45	*Wdr45*	2,41982784	4,42e-07
Zinc finger homeobox 3	*Zfhx3*	2,41773875	0,00134991
Growth hormone inducible transmembrane protein	*Ghitm*	2,41485734	2,79e-06
Hexose-6-phosphate dehydrogenase (glucose 1-dehydrogenase)	*H6pd*	2,41288039	2,31e-06
Solute carrier family 19 (folate transporter), member 1	*Slc19a1*	2,40894865	7,87e-07
Ptk7 protein tyrosine kinase 7	*Ptk7*	2,40862646	2,24e-07
Sterile alpha motif domain containing 8	*Samd8*	2,4075565	3,33e-06
Phospholipase a2, group xvi	*Pla2g16*	2,40739254	2,40e-06
Non-catalytic region of tyrosine kinase adaptor protein 1	*Nck1*	2,40635702	9,31e-06
Ribonuclease p 14 subunit	*Rpp14*	2,40256409	1,25e-06
Ubiquitin specific peptidase 36	*Usp36*	2,40141683	4,26e-05
Glutathione transferase zeta 1 (maleylacetoacetate isomerase)	*Gstz1*	2,39966273	3,96e-05
Phosphatidylinositol glycan anchor biosynthesis, class k	*Pigk*	2,39703642	4,50e-07
Sine oculis-related homeobox 1	*Six1*	2,39392898	6,71e-06
Zinc finger protein 703	*Zfp703*	2,39216217	1,01e-05
Elav (embryonic lethal, abnormal vision, drosophila)-like 2 (hu antigen b)	*Elavl2*	2,39010592	2,68e-05
Zinc finger protein 960 /// zinc finger protein 97	*Zfp960 /// zfp97*	2,38994716	8,61e-06
Obg-like atpase 1	*Ola1*	2,38874129	6,39e-07
Eph receptor a2	*Epha2*	2,38662554	9,90e-07
Wd repeat domain, phosphoinositide interacting 1	*Wipi1*	2,38656987	2,68e-05
Thioredoxin reductase 3	*Txnrd3*	2,37697689	4,68e-05
Myeloma overexpressed 2	*Myeov2*	2,37576791	1,21e-06
Rho gtpase activating protein 20	*Arhgap20*	2,37539378	5,10e-05

Potassium inwardly-rectifying channel, subfamily j, member 15	*Kcnj15*	2,37321356	0,00011629
Endonuclease domain containing 1	*Endod1*	2,37306248	1,46e-06
Rab3d, member ras oncogene family	*Rab3d*	2,37179007	5,69e-05
Camello-like 1	*Cml1*	2,36951781	4,37e-05
Catechol-o-methyltransferase domain containing 1	*Comtd1*	2,36771921	1,49e-06
Torsin family 3, member a	*Tor3a*	2,36530858	3,52e-05
Forkhead box p1	*Foxp1*	2,36431637	1,70e-05
Solute carrier family 46, member 1	*Slc46a1*	2,36011802	5,40e-05
Sec11 homolog c (s. Cerevisiae)	*Sec11c*	2,35416773	6,55e-06
Set domain, bifurcated 1	*Setdb1*	2,35295087	2,06e-06
Family with sequence similarity 210, member b	*Fam210b*	2,35182343	1,17e-05
Procollagen lysine, 2-oxoglutarate 5-dioxygenase 2	*Plod2*	2,3500749	0,00027066
Wd repeat domain 20	*Wdr20*	2,3494245	6,56e-07
La ribonucleoprotein domain family, member 6	*Larp6*	2,34929009	8,84e-06
Arfgap with rhogap domain, ankyrin repeat and ph domain 2	*Arap2*	2,34924029	6,71e-06
Golgi coiled coil 1	*Gcc1*	2,34449959	1,22e-05
Fas apoptotic inhibitory molecule	*Faim*	2,34253898	1,43e-06
Abhydrolase domain containing 14b	*Abhd14b*	2,34252805	0,0006562
Zinc finger e-box binding homeobox 2	*Zeb2*	2,34233033	5,23e-06
Pr domain containing 5	*Prdm5*	2,33994606	2,19e-06
Discoidin domain receptor family, member 1	*Ddr1*	2,33909863	3,94e-05
Protein kinase inhibitor, alpha	*Pkia*	2,33782321	0,0004957
Transient receptor potential cation channel, subfamily v, member 2	*Trpv2*	2,33730535	9,35e-06
Adhesion molecule with ig like domain 1	*Amigo1*	2,33621441	1,11e-05
Trafficking protein particle complex 2	*Trappc2*	2,33571215	6,23e-05
Phosphatidylinositol 3-kinase, c2 domain containing, alpha polypeptide	*Pik3c2a*	2,33362046	9,63e-06
Phospholipase a2, group iie	*Pla2g2e*	2,33176897	1,15e-06
Family with sequence similarity 174, member a	*Fam174a*	2,33123171	0,00025352
Fibronectin type iii domain containing 1	*Fndc1*	2,33026521	8,74e-05
Lymphocyte antigen 75	*Ly75*	2,32953741	6,87e-05
Mastermind like 1 (drosophila)	*Maml1*	2,32854448	2,46e-05
Nodal modulator 1	*Nomo1*	2,32374188	6,20e-05
Interleukin 10 receptor, beta	*Il10rb*	2,32317057	1,18e-06
Ngg1 interacting factor 3-like 1 (s. Pombe)	*Nif3l1*	2,32237989	3,77e-07

Protocadherin beta 7	*Pcdhb7*	2,32179397	8,90e-05
Myomesin 1	*Myom1*	2,31886233	3,29e-06
Lim domain containing preferred translocation partner in lipoma	*Lpp*	2,31833345	1,77e-06
Pre b cell leukemia transcription factor interacting protein 1	*Pbxip1*	2,31757969	1,77e-06
Histocompatibility 2, q region locus 4 /// histocompatibility 2, q region locus 6 /// histocompatibility 2, q region locus 8 /// histocompatibility 2, q region locus 6-like	*H2-q4 /// h2-q6 /// h2-q8 /// loc68395*	2,3152285	4,71e-06
Tnfrsf1a-associated via death domain	*Tradd*	2,31504293	0,00065953
Palladin, cytoskeletal associated protein	*Palld*	2,3145407	1,46e-06
Cytochrome p450, family 4, subfamily f, polypeptide 13	*Cyp4f13*	2,31055356	7,91e-06
Zinc finger protein 930	*Zfp930*	2,30721496	0,00224688
Bassoon	*Bsn*	2,30720273	0,00106284
Spla/ryanodine receptor domain and socs box containing 4	*Spsb4*	2,3056904	2,65e-05
Nucleus accumbens associated 2, ben and btb (poz) domain containing	*Nacc2*	2,30522683	1,37e-05
Inhibin beta-a	*Inhba*	2,30187671	2,70e-05
Golgi associated, gamma adaptin ear containing, arf binding protein 2	*Gga2*	2,29750135	6,69e-07
Cryptochrome 2 (photolyase-like)	*Cry2*	2,29686118	4,74e-05
Tripartite motif-containing 13	*Trim13*	2,28759392	0,00019454
Chemokine (c-c motif) ligand 25	*Ccl25*	2,28733239	3,05e-06
Rad52 homolog (s. Cerevisiae)	*Rad52*	2,2853705	7,95e-05
Non-catalytic region of tyrosine kinase adaptor protein 2	*Nck2*	2,28485487	1,33e-06
Predicted gene 13363 /// protein tyrosine phosphatase 4a1	*Gm13363 /// ptp4a1*	2,27957913	0,00379532
Transmembrane protein 41a	*Tmem41a*	2,27503794	2,07e-05
Acid phosphatase 2, lysosomal	*Acp2*	2,27492595	5,77e-06
Kinesin family member c3	*Kifc3*	2,27184475	1,36e-05
Membrane magnesium transporter 2	*Mmgt2*	2,26990786	5,00e-06
Aldehyde dehydrogenase 2, mitochondrial	*Aldh2*	2,26897652	3,70e-07
Protein-o-mannose kinase	*Pomk*	2,26747856	0,00030688
Selenoprotein m	*Selm*	2,26737428	0,00012314

Cellular repressor of e1a-stimulated genes 1	*Creg1*	2,2670189	1,88e-05
Udp-glcnac:betagal beta-1,3-n-acetylglucosaminyltransferase 2	*B3gnt2*	2,26658075	5,69e-05
General transcription factor iia, 1-like	*Gtf2a1l*	2,26601713	9,17e-06
Sema domain, immunoglobulin domain (ig), and gpi membrane anchor, (semaphorin) 7a	*Sema7a*	2,26414655	6,31e-05
Tetratricopeptide repeat domain 17	*Ttc17*	2,263579	3,00e-05
Interleukin 3 receptor, alpha chain	*Il3ra*	2,26347806	0,00019028
Solute carrier family 3 (activators of dibasic and neutral amino acid transport), member 2	*Slc3a2*	2,26320456	4,26e-07
Potassium channel tetramerisation domain containing 1	*Kctd1*	2,26109762	1,34e-05
Cap methyltransferase 2	*Cmtr2*	2,26052531	0,00156991
Fin bud initiation factor homolog (zebrafish)	*Fibin*	2,25744586	9,51e-05
Uroporphyrinogen iii synthase	*Uros*	2,25709594	1,06e-06
Riken cdna 2810408m09 gene /// transformation related protein 53 regulating kinase	*2810408m09rik /// trp53rk*	2,25438398	0,00010476
Post-gpi attachment to proteins 2	*Pgap2*	2,25277748	0,00010701
Calsyntenin 1	*Clstn1*	2,25167753	1,17e-06
Rb1-inducible coiled-coil 1	*Rb1cc1*	2,25125218	1,34e-06
Endoplasmic reticulum lectin 1	*Erlec1*	2,25101061	0,00015654
Nfkb activating protein-like	*Nkapl*	2,25054469	1,38e-05
Integrin beta 6	*Itgb6*	2,24959741	3,24e-05
Mitochondrial ribosomal protein l50	*Mrpl50*	2,24767275	3,00e-05
Ectonucleotide pyrophosphatase/phosphodiesterase 4	*Enpp4*	2,24672842	8,24e-06
Mitochondrial ribosomal protein s2	*Mrps2*	2,24629292	6,44e-07
Peroxiredoxin 6	*Prdx6*	2,24546341	1,74e-06
Glutamyl-trna synthetase 2 (mitochondrial)(putative)	*Ears2*	2,245029	1,54e-05
Transmembrane protein 115	*Tmem115*	2,24473829	3,62e-05
Phosphohistidine phosphatase 1	*Phpt1*	2,24272188	0,00504549
Creatine kinase, brain	*Ckb*	2,24215339	1,50e-06
Dual specificity phosphatase 14	*Dusp14*	2,2418213	2,66e-06
Sec62 homolog (s. Cerevisiae)	*Sec62*	2,24118266	0,00121725
Zinc finger protein 937	*Zfp937*	2,23853567	1,01e-06
Baculoviral iap repeat-containing 3	*Birc3*	2,23733482	6,82e-05
Mitogen-activated protein kinase 12	*Mapk12*	2,23680764	1,67e-05

Solute carrier family 35, member c1	*Slc35c1*	2,23547978	0,00048157
Male-specific lethal 3-like 2 (drosophila)	*Msl3l2*	2,23509564	2,77e-06
Kinesin family member 1b	*Kif1b*	2,23504608	8,35e-06
Translation machinery associated 16 homolog (s. Cerevisiae)	*Tma16*	2,23440802	1,23e-05
Neurotrimin	*Ntm*	2,23407324	0,00061557
T cell lymphoma invasion and metastasis 1	*Tiam1*	2,23363551	0,00013815
Phosphorylase kinase alpha 1	*Phka1*	2,2325548	6,87e-05
Disabled 2 interacting protein	*Dab2ip*	2,23252662	3,47e-06
Thump domain containing 1	*Thumpd1*	2,23084124	2,64e-06
Bcs1-like (yeast)	*Bcs1l*	2,22973551	7,26e-07
Calcium channel, voltage-dependent, l type, alpha 1c subunit	*Cacna1c*	2,22835669	6,71e-06
Expressed sequence ai503316 /// heterogeneous nuclear ribonucleoprotein u	*Ai503316 /// hnrnpu*	2,22623383	2,04e-05
Phosphodiesterase 4d interacting protein (myomegalin)	*Pde4dip*	2,22338597	0,00034899
G elongation factor, mitochondrial 1	*Gfm1*	2,22315982	3,40e-07
Solute carrier family 20, member 1	*Slc20a1*	2,22234249	1,75e-06
Zinc finger, an1-type domain 1	*Zfand1*	2,22183643	5,84e-05
2,4-dienoyl coa reductase 1, mitochondrial	*Decr1*	2,22113496	2,22e-06
Neuraminidase 1	*Neu1*	2,21943377	2,82e-05
Transmembrane protein 30a	*Tmem30a*	2,21938215	1,44e-05
Protein phosphatase 1, regulatory (inhibitor) subunit 14c	*Ppp1r14c*	2,21820972	3,79e-05
Syndecan 4	*Sdc4*	2,21671326	1,77e-05
Pelota homolog (drosophila)	*Pelo*	2,21440346	1,69e-05
Nuclear protein transcription regulator 1	*Nupr1*	2,21345342	9,55e-06
Sr-related ctd-associated factor 8	*Scaf8*	2,21183569	9,23e-06
Lectin, galactoside-binding, soluble, 3 binding protein	*Lgals3bp*	2,20999232	6,87e-05
Complement component 2 (within h-2s)	*C2*	2,2084382	5,51e-06
Yip1 domain family, member 2	*Yipf2*	2,20544623	0,00015361
L-3-hydroxyproline dehydratase (trans-)	*L3hypdh*	2,20530651	0,00026304
Riken cdna 2610301b20 gene	*2610301b20rik*	2,20303878	7,35e-05
Transcription elongation factor, mitochondrial	*Tefm*	2,20053348	2,73e-05
Phosphatidylinositol glycan anchor biosynthesis, class s	*Pigs*	2,19507177	1,87e-06

Feminization 1 homolog b (c. Elegans)	*Fem1b*	2,19408733	9,47e-05
Udp-glcnac:betagal beta-1,3-n-acetylglucosaminyltransferase 3	*B3gnt3*	2,1931716	0,00013216
Ribosomal rna processing 15 homolog (s. Cerevisiae)	*Rrp15*	2,19288807	1,44e-05
Major histocompatibility complex, class i-related	*Mr1*	2,19085091	0,00018825
Bone morphogenetic protein 1	*Bmp1*	2,19047322	8,38e-05
P21 protein (cdc42/rac)-activated kinase 1	*Pak1*	2,18812955	5,10e-07
Tripeptidyl peptidase i	*Tpp1*	2,18757917	3,37e-06
Phenylalanine-trna synthetase 2 (mitochondrial)	*Fars2*	2,18720485	0,00091472
Density-regulated protein	*Denr*	2,18702026	1,48e-06
Biphenyl hydrolase-like (serine hydrolase, breast epithelial mucin-associated antigen)	*Bphl*	2,18442724	4,14e-06
Membrane associated guanylate kinase, ww and pdz domain containing 3	*Magi3*	2,1842783	8,13e-05
Fibroblast growth factor binding protein 1	*Fgfbp1*	2,18409123	0,00018861
Expressed sequence ai837181	*Ai837181*	2,18314085	1,57e-05
Rho gtpase activating protein 21	*Arhgap21*	2,18296459	3,96e-06
Lanc (bacterial lantibiotic synthetase component c)-like 1	*Lancl1*	2,17949811	6,81e-07
Ataxin 7-like 1	*Atxn7l1*	2,17936215	5,11e-06
Prostaglandin e synthase	*Ptges*	2,17512493	3,46e-06
C-type lectin domain family 4, member e	*Clec4e*	2,17476957	9,04e-06
Glutaredoxin	*Glrx*	2,17475829	4,74e-07
Solute carrier family 39 (zinc transporter), member 14	*Slc39a14*	2,17462659	1,79e-06
Histone deacetylase 11	*Hdac11*	2,1734078	3,54e-05
Dph5 homolog (s. Cerevisiae)	*Dph5*	2,17280921	9,93e-07
Transmembrane protein 11	*Tmem11*	2,17221865	2,87e-06
Eh domain binding protein 1-like 1	*Ehbp1l1*	2,17075678	8,39e-06
Cleavage stimulation factor, 3' pre-rna subunit 2, tau	*Cstf2t*	2,16984923	1,30e-06
Cytochrome c oxidase assembly factor 7	*Coa7*	2,16883354	2,24e-06
5'-nucleotidase, cytosolic iiib	*Nt5c3b*	2,16855386	1,26e-05
Nucleobindin 1	*Nucb1*	2,16821625	5,76e-06
Angel homolog 2 (drosophila)	*Angel2*	2,16736055	0,00087444
Isoamyl acetate-hydrolyzing esterase 1 homolog (s. Cerevisiae)	*Iah1*	2,16688368	4,80e-06
Cd38 antigen	*Cd38*	2,16655521	1,20e-05
Dynein light chain tctex-type 3	*Dynlt3*	2,16517531	4,18e-06

Poliovirus receptor-related 2	*Pvrl2*	2,1614209	1,23e-05
Phospholipase a2 receptor 1	*Pla2r1*	2,15873453	2,79e-06
Myelocytomatosis oncogene	*Myc*	2,15572063	1,21e-05
Yth domain family 2	*Ythdf2*	2,15434177	3,43e-06
Tetratricopeptide repeat domain 14	*Ttc14*	2,15399662	7,98e-05
Coiled-coil domain containing 107	*Ccdc107*	2,15375358	5,36e-06
Autophagy related 10	*Atg10*	2,15180228	1,30e-05
Fragile x mental retardation 1 neighbor	*Fmr1nb*	2,15136528	6,52e-06
Tubulin polymerization-promoting protein family member 3	*Tppp3*	2,15038002	6,22e-05
Colony stimulating factor 1 (macrophage)	*Csf1*	2,15037652	2,94e-06
Transmembrane protein 70	*Tmem70*	2,14892102	3,30e-06
Filamin binding lim protein 1	*Fblim1*	2,14775846	3,87e-06
Arfgap with gtpase domain, ankyrin repeat and ph domain 1	*Agap1*	2,14501505	1,27e-05
3-phosphoglycerate dehydrogenase pseudogene /// 3-phosphoglycerate dehydrogenase	*Gm6756 /// phgdh*	2,14459185	4,56e-07
Cell division cycle 73, paf1/rna polymerase ii complex component	*Cdc73*	2,14420446	3,56e-05
Hypoxia up-regulated 1	*Hyou1*	2,14396155	0,00094405
Cell cycle associated protein 1	*Caprin1*	2,1392139	3,60e-05
Proline-rich nuclear receptor coactivator 1	*Pnrc1*	2,13911202	4,15e-06
Ras p21 protein activator 3	*Rasa3*	2,13849049	8,57e-07
Brain and acute leukemia, cytoplasmic	*Baalc*	2,13839045	2,07e-06
Golgi phosphoprotein 3-like	*Golph3l*	2,1367046	3,11e-06
A kinase (prka) anchor protein 2	*Akap2*	2,13650447	4,43e-06
Pseudouridylate synthase 10	*Pus10*	2,13307637	0,00053519
Neuroblastoma ras oncogene	*Nras*	2,13291134	4,05e-06
Mitogen-activated protein kinase kinase kinase 8	*Map3k8*	2,13216597	0,00067565
Heat shock protein 5	*Hspa5*	2,13168275	3,78e-06
Solute carrier family 25, member 45	*Slc25a45*	2,12743682	7,16e-05
Zinc finger swim-type containing 1	*Zswim1*	2,1266814	2,07e-05
Fumarylacetoacetate hydrolase	*Fah*	2,12547217	3,07e-06
Runt related transcription factor 1	*Runx1*	2,12545477	2,05e-05
Interleukin 17 receptor c	*Il17rc*	2,12391206	1,06e-05
Nudix (nucleoside diphosphate linked moiety x)-type motif 16	*Nudt16*	2,12385207	1,46e-05
Astrotactin 1	*Astn1*	2,12307439	8,85e-05

Neugrin, neurite outgrowth associated	*Ngrn*	2,12232385	0,00022217
Activin receptor iia	*Acvr2a*	2,12134341	2,05e-06
Egf-like repeats and discoidin i-like domains 3	*Edil3*	2,11894727	0,0022412
Methyltransferase like 6	*Mettl6*	2,11700661	2,77e-05
Myosin id	*Myo1d*	2,11652524	0,00197423
Diacylglycerol o-acyltransferase 2	*Dgat2*	2,11583638	2,00e-05
Golgi membrane protein 1	*Golm1*	2,11439917	1,63e-06
Atp5s-like	*Atp5sl*	2,11297203	2,11e-05
Transmembrane protein 191c	*Tmem191c*	2,11280678	0,00021368
Adipocyte plasma membrane associated protein	*Apmap*	2,1127467	6,74e-06
Exostoses (multiple)-like 2	*Extl2*	2,10988729	2,88e-06
Sh3-domain binding protein 4	*Sh3bp4*	2,10935926	3,97e-06
Inhibin beta-b	*Inhbb*	2,10883144	0,00044776
Vesicle-associated membrane protein 2	*Vamp2*	2,10860127	0,00568917
Glutaryl-coenzyme a dehydrogenase	*Gcdh*	2,10809041	1,98e-05
Cd320 antigen	*Cd320*	2,1064965	1,68e-06
Atm interactor	*Atmin*	2,10592914	6,10e-06
Eukaryotic translation initiation factor 1a, x-linked	*Eif1ax*	2,10432486	4,41e-05
Receptor-type tyrosine-protein phosphatase eta-like /// protein tyrosine phosphatase, receptor type, j	*Loc102641972 /// ptprj*	2,1042178	0,00408396
Superoxide dismutase 2, mitochondrial	*Sod2*	2,10420787	4,37e-07
Shroom family member 3	*Shroom3*	2,10381393	0,00275905
Transmembrane emp24-like trafficking protein 10 (yeast)	*Tmed10*	2,10341017	5,33e-07
Rho gdp dissociation inhibitor (gdi) gamma	*Arhgdig*	2,10269951	2,81e-06
Interferon (alpha and beta) receptor 2	*Ifnar2*	2,10246131	7,20e-07
Zinc finger, cchc domain containing 18	*Zcchc18*	2,10207063	0,00018938
Sorting nexin 18	*Snx18*	2,10146771	1,93e-06
Cleavage stimulation factor, 3' pre-rna subunit 2	*Cstf2*	2,10075518	2,20e-05
Polyribonucleotide nucleotidyltransferase 1	*Pnpt1*	2,0990668	8,20e-05
Ubiquitin-conjugating enzyme e2 variant 2	*Ube2v2*	2,09743182	0,00120974
Tnfaip3 interacting protein 1	*Tnip1*	2,09510015	5,67e-06
Apolipoprotein e	*Apoe*	2,09374935	4,01e-05
Tyrosine kinase, non-receptor, 2	*Tnk2*	2,09311555	0,00021114
Dalr anticodon binding domain containing 3	*Dalrd3*	2,08596676	6,11e-06
Radial spoke head 9 homolog (chlamydomonas)	*Rsph9*	2,08570777	2,71e-05
Riken cdna 1110008p14 gene	*1110008p14rik*	2,08525103	9,08e-06
Methyltransferase like 8	*Mettl8*	2,08376194	2,82e-05

Z-dna binding protein 1	*Zbp1*	2,08312076	6,08e-06
Zinc finger and btb domain containing 21	*Zbtb21*	2,08064497	9,59e-05
Crystallin, zeta	*Cryz*	2,07924831	1,26e-06
Cingulin-like 1	*Cgnl1*	2,07908743	5,36e-07
Protein phosphatase 1b, magnesium dependent, beta isoform	*Ppm1b*	2,07736289	1,43e-06
Ubiquitin-associated protein 1	*Ubap1*	2,0769676	8,01e-07
Zinc finger protein 830	*Zfp830*	2,07668428	0,00062507
Egf domain-specific o-linked n-acetylglucosamine (glcnac) transferase	*Eogt*	2,07605574	1,56e-05
Purinergic receptor p2x, ligand-gated ion channel, 3	*P2rx3*	2,07577038	0,00034361
Glucosamine-phosphate n-acetyltransferase 1	*Gnpnat1*	2,0757461	2,20e-06
Component of oligomeric golgi complex 8	*Cog8*	2,07504663	2,36e-06
Ring finger protein 11	*Rnf11*	2,0745438	8,57e-07
Pecanex-like 4 (drosophila)	*Pcnxl4*	2,07096509	4,15e-06
Zinc finger protein interacting with k protein 1	*Zik1*	2,07021054	9,59e-05
Catechol-o-methyltransferase	*Comt*	2,06963523	0,00205738
Potassium voltage-gated channel, subfamily h (eag-related), member 2	*Kcnh2*	2,0694996	0,00012766
Afg3-like aaa atpase 2	*Afg3l2*	2,0683958	4,80e-05
Iron-sulfur cluster assembly 2 homolog (s. Cerevisiae)	*Isca2*	2,06738283	2,79e-06
Proteasome (prosome, macropain) 26s subunit, non-atpase, 10	*Psmd10*	2,06642615	6,77e-06
Phosphodiesterase 10a	*Pde10a*	2,06439519	0,00057784
Hect, uba and wwe domain containing 1	*Huwe1*	2,06358013	4,43e-06
Zinc finger protein 2	*Zfp2*	2,06240781	1,79e-05
Transcription elongation factor b (siii), polypeptide 1	*Tceb1*	2,06079121	3,66e-06
Transforming growth factor beta regulated gene 1	*Tbrg1*	2,06012118	1,35e-06
Solute carrier family 30 (zinc transporter), member 4	*Slc30a4*	2,05900552	2,00e-05
Ventral anterior homeobox 2	*Vax2*	2,05894997	1,75e-05
Translocase of inner mitochondrial membrane 8a1	*Timm8a1*	2,05876915	1,34e-05
Glycogenin	*Gyg*	2,05795145	6,18e-06
Protein-l-isoaspartate (d-aspartate) o-methyltransferase domain containing 2	*Pcmtd2*	2,05769587	0,00100164
Mitochondrial ribosomal protein l44	*Mrpl44*	2,05760726	5,05e-06
Heat shock protein 9	*Hspa9*	2,05691826	9,05e-07
Creb/atf bzip transcription factor	*Crebzf*	2,05450508	0,00473611

Complement component 1, r subcomponent a	*C1ra*	2,0532636	2,31e-06
Friend leukemia integration 1	*Fli1*	2,04707482	0,00014722
Snail family zinc finger 2	*Snai2*	2,04684495	2,66e-05
Dna-damage regulated autophagy modulator 2	*Dram2*	2,0452602	5,48e-05
Androgen receptor	*Ar*	2,04402996	0,00050185
Insulin receptor substrate 2	*Irs2*	2,04269037	3,80e-05
Predicted gene, 20559	*Gm20559*	2,04026771	3,82e-06
Karyopherin (importin) alpha 1	*Kpna1*	2,03961125	0,00052265
Poliovirus receptor	*Pvr*	2,03939655	4,38e-05
Zinc finger protein 281	*Zfp281*	2,03938974	4,21e-06
Intraflagellar transport 81	*Ift81*	2,03880198	4,08e-06
Translocase of outer mitochondrial membrane 70 homolog a (yeast)	*Tomm70a*	2,03764639	0,00012857
Cytotoxic t lymphocyte-associated protein 2 beta	*Ctla2b*	2,03686918	7,87e-07
Guanine nucleotide binding protein (g protein), beta polypeptide 1-like	*Gnb1l*	2,03599126	1,90e-05
Meteorin, glial cell differentiation regulator-like	*Metrnl*	2,03583879	4,65e-06
Chemokine (c-c motif) ligand 2	*Ccl2*	2,03568083	5,80e-05
Riken cdna 1700010i14 gene	*1700010i14rik*	2,03453407	0,00010202
Translocase of inner mitochondrial membrane 10	*Timm10*	2,03424505	1,86e-05
Udp-glucuronate decarboxylase 1	*Uxs1*	2,03421243	0,00014195
Coenzyme q3 homolog, methyltransferase (yeast)	*Coq3*	2,03366775	4,77e-05
Rap1 interacting factor 1 homolog (yeast)	*Rif1*	2,03157737	0,00067701
Ww domain-containing oxidoreductase	*Wwox*	2,03114818	1,22e-05
Phosphoribosyl pyrophosphate synthetase 1 /// phosphoribosyl pyrophosphate synthetase 1-like 3	*Prps1 /// prps1l3*	2,03079917	0,00078912
Wd repeat domain 33	*Wdr33*	2,0307778	0,0003787
Ubiquitin-conjugating enzyme e2w (putative)	*Ube2w*	2,02946204	0,00119469
Znrd1 antisense	*Znrd1as*	2,02938293	7,15e-05
Coiled-coil-helix-coiled-coil-helix domain containing 4	*Chchd4*	2,02816368	1,81e-05
Olfactomedin-like 2b	*Olfml2b*	2,02799102	5,95e-06
Family with sequence similarity 63, member a	*Fam63a*	2,02778687	1,19e-05
Neogenin	*Neo1*	2,02566059	5,46e-06
Collagen, type v, alpha 1	*Col5a1*	2,02543844	2,63e-06
Lysine rich nucleolar protein 1	*Knop1*	2,02536668	1,72e-06
Family with sequence similarity 118, member b	*Fam118b*	2,02375183	3,11e-06
Taxilin beta	*Txlnb*	2,02301698	0,0035914

Protein kinase, dna activated, catalytic polypeptide	*Prkdc*	2,0228925	1,84e-05
Guanylate binding protein 2b	*Gbp2b*	2,02240429	3,53e-06
At rich interactive domain 5b (mrf1-like)	*Arid5b*	2,02226187	4,07e-06
G protein-coupled receptor 137b /// g protein-coupled receptor 137b, pseudogene	*Gpr137b /// gpr137b-ps*	2,02224936	6,32e-07
Hydroxysteroid (17-beta) dehydrogenase 10	*Hsd17b10*	2,02173204	1,74e-05
Fyn binding protein	*Fyb*	2,01999313	2,31e-05
Aftiphilin	*Aftph*	2,01964717	6,32e-06
Bystin-like	*Bysl*	2,01898372	5,53e-05
Methylthioadenosine phosphorylase	*Mtap*	2,01676177	1,11e-06
Apolipoprotein a-i binding protein	*Apoa1bp*	2,01362882	8,73e-07
Interferon-induced protein with tetratricopeptide repeats 1	*Ifit1*	2,01188892	6,18e-05
Methylenetetrahydrofolate dehydrogenase (nad+ dependent), methenyltetrahydrofolate cyclohydrolase	*Mthfd2*	2,01074902	4,80e-06
Glypican 6	*Gpc6*	2,00989888	4,17e-05
Mitogen-activated protein kinase 8 interacting protein 1	*Mapk8ip1*	2,00984945	0,00021457
Cytidine and dcmp deaminase domain containing 1	*Cdadc1*	2,00904847	0,00053742
Atp-binding cassette, sub-family c (cftr/mrp), member 5	*Abcc5*	2,00847681	1,74e-06
Mesoderm development candidate 2	*Mesdc2*	2,00670061	9,85e-07
Gtp cyclohydrolase 1	*Gch1*	2,00620721	0,00193333
Cytochrome p450, family 39, subfamily a, polypeptide 1	*Cyp39a1*	2,00435146	0,0004947
Ddhd domain containing 1	*Ddhd1*	2,00421194	0,00043874
Progesterone receptor membrane component 2	*Pgrmc2*	2,00350011	2,57e-05
Bridging integrator 1	*Bin1*	2,0032385	1,68e-06
Protein kinase c, delta	*Prkcd*	2,0029586	4,62e-06
Trna methyltransferase 10b	*Trmt10b*	2,00292894	0,00011142
Ankyrin repeat domain 13c	*Ankrd13c*	2,00284857	1,52e-05
2-oxoglutarate and iron-dependent oxygenase domain containing 3	*Ogfod3*	2,0018096	2,89e-05
Xylulokinase homolog (h. Influenzae)	*Xylb*	2,00107536	0,0004947
Cadherin 2	*Cdh2*	2,00100155	1,87e-05
Phosphatidylserine decarboxylase, pseudogene 1 /// phosphatidylserine decarboxylase, pseudogene 3	*Pisd-ps1 /// pisd-ps3*	2,00090175	7,89e-05

Cysteine and glycine-rich protein 1	*Csrp1*	2,00067512	0,00016953
Ganglioside-induced differentiation-associated protein 1-like 1	*Gdap1l1*	1,99735191	0,00032218
Carbonic anhydrase 11	*Car11*	1,99687838	3,06e-05
Wd repeat domain 12	*Wdr12*	1,99678572	6,18e-05
Insulin-like growth factor 2 mrna binding protein 2	*Igf2bp2*	1,9966438	1,89e-06
Slit-robo rho gtpase activating protein 2	*Srgap2*	1,99638521	4,02e-06
Serine (or cysteine) peptidase inhibitor, clade i, member 2	*Serpini2*	1,99507196	8,45e-05
Solute carrier family 7 (cationic amino acid transporter, y+ system), member 2	*Slc7a2*	1,99503049	0,00018189
Rab9, member ras oncogene family	*Rab9*	1,99446692	6,08e-06
Rrs1 ribosome biogenesis regulator homolog (s. Cerevisiae)	*Rrs1*	1,99402261	6,73e-06
Complement component (3b/4b) receptor 1-like	*Cr1l*	1,99270214	7,81e-06
Regulator of chromosome condensation (rcc1) and btb (poz) domain containing protein 2	*Rcbtb2*	1,99245846	0,00038093
Bicaudal d homolog 1 (drosophila)	*Bicd1*	1,99125968	4,37e-05
Dopey family member 2	*Dopey2*	1,99123522	3,19e-05
Suppression of tumorigenicity 5	*St5*	1,99107738	8,24e-06
Iroquois related homeobox 1 (drosophila)	*Irx1*	1,98950279	4,37e-06
Six transmembrane epithelial antigen of the prostate 1	*Steap1*	1,98890401	8,98e-07
Dual specificity phosphatase 16	*Dusp16*	1,98797408	3,31e-06
Chloride channel 3	*Clcn3*	1,98682918	3,40e-05
Xenotropic and polytropic retrovirus receptor 1	*Xpr1*	1,98660019	1,34e-05
Ras homolog gene family, member c	*Rhoc*	1,98497739	0,00112429
Solute carrier organic anion transporter family, member 1a6	*Slco1a6*	1,98374661	0,00147251
Limb region 1	*Lmbr1*	1,98353719	5,10e-05
Baculoviral iap repeat-containing 2	*Birc2*	1,98332023	4,21e-06
Queuine trna-ribosyltransferase 1	*Qtrt1*	1,98301447	9,13e-06
Rna methyltransferase like 1	*Rnmtl1*	1,9829129	1,12e-06
Calmin	*Clmn*	1,98231377	0,00025643
Membrane-spanning 4-domains, subfamily a, member 4d	*Ms4a4d*	1,98219587	8,16e-05
Keratinocyte associated protein 2	*Krtcap2*	1,98171599	6,59e-06
Programmed cell death 6 interacting protein	*Pdcd6ip*	1,9809943	0,00043658

Htra serine peptidase 3	*Htra3*	1,98077334	5,08e-05
Low density lipoprotein receptor-related protein associated protein 1	*Lrpap1*	1,98028666	7,39e-06
Ring finger protein 38	*Rnf38*	1,97846691	2,64e-06
Rab gtpase activating protein 1	*Rabgap1*	1,97694454	0,00603852
Coiled-coil domain containing 120	*Ccdc120*	1,97612518	0,00012539
Methylcrotonoyl-coenzyme a carboxylase 1 (alpha)	*Mccc1*	1,97601267	9,70e-06
Ly6/neurotoxin 1	*Lynx1*	1,97504325	1,30e-05
Tnf receptor-associated protein 1	*Trap1*	1,97502914	1,74e-06
Orm1-like 1 (s. Cerevisiae)	*Ormdl1*	1,97493393	0,00345037
Junction-mediating and regulatory protein	*Jmy*	1,97233387	0,0048985
Apurinic/apyrimidinic endonuclease 1	*Apex1*	1,97126885	1,33e-06
U2af homology motif (uhm) kinase 1	*Uhmk1*	1,96973632	1,33e-05
Ferric-chelate reductase 1	*Frrs1*	1,9691449	3,70e-05
Natriuretic peptide receptor 2	*Npr2*	1,96911902	5,07e-05
Son of sevenless homolog 2 (drosophila)	*Sos2*	1,96878859	0,000191
Sry (sex determining region y)-box 12	*Sox12*	1,96776043	5,60e-05
Ral gef with ph domain and sh3 binding motif 2	*Ralgps2*	1,96768853	2,32e-06
Prefoldin 2	*Pfdn2*	1,9662006	1,17e-05
Methylmalonyl-coenzyme a mutase	*Mut*	1,96596954	2,17e-05
Tight junction associated protein 1	*Tjap1*	1,9653483	6,08e-06
Purine-rich element binding protein g	*Purg*	1,96424172	0,00012279
Eyes absent 1 homolog (drosophila)	*Eya1*	1,96199828	4,17e-05
Yth domain family 3	*Ythdf3*	1,96045188	3,76e-05
Serine hydroxymethyltransferase 2 (mitochondrial)	*Shmt2*	1,95867778	4,53e-05
Prostaglandin reductase 2	*Ptgr2*	1,9572713	0,00028524
Inhibitor of kappab kinase gamma	*Ikbkg*	1,956831	1,37e-06
Riken cdna 0610031j06 gene	*0610031j06rik*	1,95567584	1,45e-06
Intercellular adhesion molecule 1	*Icam1*	1,95566615	0,00013768
X-linked myotubular myopathy gene 1	*Mtm1*	1,95491138	0,00070926
Fun14 domain containing 2	*Fundc2*	1,95442194	0,00047929
Patatin-like phospholipase domain containing 7	*Pnpla7*	1,95351366	3,15e-05
Rho-related btb domain containing 2	*Rhobtb2*	1,95180752	5,03e-05
Cyclin pas1/pho80 domain containing 1	*Cnppd1*	1,95113151	2,58e-06
Sorting nexin 19	*Snx19*	1,94904987	1,14e-05
Coiled-coil domain containing 90b	*Ccdc90b*	1,9467359	2,19e-05
Interleukin 4 receptor, alpha	*Il4ra*	1,9464604	5,21e-06
Proteasome (prosome, macropain) 26s subunit,	*Psmc6*	1,94559311	0,00778278

atpase, 6			
Polymerase (rna) iii (dna directed) polypeptide g like	*Polr3gl*	1,9448916	0,00074718
Nuclear receptor subfamily 2, group f, member 2	*Nr2f2*	1,94329474	0,00314983
Teneurin transmembrane protein 4	*Tenm4*	1,94313681	2,01e-05
Sap30 binding protein	*Sap30bp*	1,94280117	1,21e-05
Polymerase (rna) iii (dna directed) polypeptide e	*Polr3e*	1,94267623	1,03e-05
Nme/nm23 nucleoside diphosphate kinase 4	*Nme4*	1,94198739	0,00100154
Adp-ribosylation factor guanine nucleotide-exchange factor 1(brefeldin a-inhibited)	*Arfgef1*	1,94117471	1,75e-06
Pq loop repeat containing 1	*Pqlc1*	1,94081941	9,50e-06
Growth arrest specific 5	*Gas5*	1,9408025	6,01e-06
Complement component 1, q subcomponent binding protein	*C1qbp*	1,94058793	1,19e-06
Phosphatidylinositol-5-phosphate 4-kinase, type ii, alpha	*Pip4k2a*	1,94056101	3,94e-06
Aar2 splicing factor homolog (s. Cerevisiae)	*Aar2*	1,93691175	0,00170951
Cyclin m3	*Cnnm3*	1,9348957	5,43e-06
Nucleolin	*Ncl*	1,93452379	3,85e-06
Phospholipase a1 member a	*Pla1a*	1,9338301	4,23e-06
Protein inhibitor of activated stat 1	*Pias1*	1,93273309	0,00088501
Bro1 domain and caax motif containing	*Brox*	1,9316694	2,22e-06
Pdz and lim domain 7	*Pdlim7*	1,93129751	0,00017177
Mitochondrial ribosomal protein s18b	*Mrps18b*	1,92984794	4,73e-06
Riken cdna 5430417l22 gene	*5430417l22rik*	1,9288781	7,98e-06
Interferon gamma induced gtpase	*Igtp*	1,92887056	0,00048463
Yes-associated protein 1	*Yap1*	1,92749939	5,51e-06
Torsin family 4, member a	*Tor4a*	1,92749801	8,85e-06
Motile sperm domain containing 1	*Mospd1*	1,92667318	1,42e-05
Quiescin q6 sulfhydryl oxidase 1	*Qsox1*	1,92409803	5,19e-06
Vacuolar protein sorting 53 (yeast)	*Vps53*	1,92247784	3,02e-06
Zinc finger and btb domain containing 44	*Zbtb44*	1,92108558	1,40e-05
Asparagine synthetase domain containing 1	*Asnsd1*	1,92019418	1,72e-06
Regulation of nuclear pre-mrna domain containing 1a	*Rprd1a*	1,9197731	6,84e-06
Charged multivesicular body protein 1b	*Chmp1b*	1,91885164	2,41e-05
Prolyl-trna synthetase (mitochondrial)(putative)	*Pars2*	1,91842823	2,51e-05
Re1-silencing transcription factor	*Rest*	1,91798163	0,00386607
Glycerophosphodiester phosphodiesterase domain	*Gdpd1*	1,91782324	0,00024488

containing 1			
Solute carrier family 44, member 1	*Slc44a1*	1,91693794	4,18e-05
Mitochondrial ribosomal protein s14	*Mrps14*	1,91626769	4,09e-05
Rab8b, member ras oncogene family	*Rab8b*	1,91368277	2,07e-05
Transmembrane channel-like gene family 6	*Tmc6*	1,91288485	1,51e-05
Yy1 transcription factor	*Yy1*	1,91192192	4,93e-06
Interferon gamma inducible protein 47	*Ifi47*	1,91134944	1,01e-05
Formin 1	*Fmn1*	1,91124189	0,00012715
Reticulocalbin 2	*Rcn2*	1,9099458	0,00117018
Wingless-type mmtv integration site family, member 5b	*Wnt5b*	1,90953957	0,00174471
Protein-l-isoaspartate (d-aspartate) o-methyltransferase domain containing 1	*Pcmtd1*	1,90953288	0,00010298
Emopamil binding protein-like	*Ebpl*	1,90948251	5,68e-05
Asparagine synthetase	*Asns*	1,90891014	9,02e-06
Solute carrier family 25, member 51	*Slc25a51*	1,9073312	3,02e-06
Homeodomain interacting protein kinase 3	*Hipk3*	1,90708481	2,29e-05
Chromobox 8	*Cbx8*	1,90624227	7,46e-06
Ras-like, family 12	*Rasl12*	1,90418528	1,36e-05
Solute carrier family 12, member 5	*Slc12a5*	1,90343786	4,92e-06
Zinc finger protein 568	*Zfp568*	1,90279833	0,00711548
Kelch-like 26	*Klhl26*	1,90229357	1,07e-05
Marcks-like 1	*Marcksl1*	1,90214214	2,83e-05
Rab interacting lysosomal protein-like 1	*Rilpl1*	1,90102094	0,00032569
Von willebrand factor a domain containing 8	*Vwa8*	1,90011103	0,00170951
Cytosolic iron-sulfur protein assembly 1	*Ciao1*	1,89928847	3,10e-06
Mitochondrial ribosomal protein l46	*Mrpl46*	1,89755174	7,81e-06
Tgf-beta activated kinase 1/map3k7 binding protein 3	*Tab3*	1,89732504	2,90e-05
Wingless-type mmtv integration site family, member 5a	*Wnt5a*	1,89699176	3,67e-05
Ubiquitin carboxyl-terminal esterase l5	*Uchl5*	1,89670625	0,00013448
Tripartite motif-containing 44	*Trim44*	1,89617679	2,52e-06
Dnaj (hsp40) homolog, subfamily c, member 27	*Dnajc27*	1,89507105	0,00030648
Methyltransferase like 21a	*Mettl21a*	1,89409923	7,63e-05
Transmembrane protein 141	*Tmem141*	1,89399347	3,98e-06
Islet cell autoantigen 1	*Ica1*	1,8935146	0,00128686
Calpain 1	*Capn1*	1,89335381	0,00078186

Tudor domain containing 7	*Tdrd7*	1,89296003	1,12e-05
Activating transcription factor 5	*Atf5*	1,89227153	0,0003796
Cytochrome c oxidase subunit vib polypeptide 2	*Cox6b2*	1,89215999	0,00116435
Laminin, alpha 5	*Lama5*	1,89116309	2,91e-05
Ubiquilin 4	*Ubqln4*	1,88998809	0,00088277
Glutamine and serine rich 1	*Qser1*	1,88931555	0,00023654
Cathepsin a	*Ctsa*	1,88856256	1,62e-06
Bifunctional apoptosis regulator	*Bfar*	1,88845523	6,58e-05
Notch 2	*Notch2*	1,88767149	3,22e-05
Porcupine homolog (drosophila)	*Porcn*	1,8863939	7,11e-05
Growth arrest and dna-damage-inducible 45 gamma	*Gadd45g*	1,8863859	2,30e-05
Mitochondrial ribosomal protein l38	*Mrpl38*	1,88573554	5,01e-05
N(alpha)-acetyltransferase 15, nata auxiliary subunit	*Naa15*	1,88531369	5,40e-05
Zinc finger protein 51	*Zfp51*	1,88458488	0,00098041
Angiomotin-like 2	*Amotl2*	1,88395137	4,32e-06
Microfibrillar-associated protein 3	*Mfap3*	1,88331003	7,22e-05
Myosin va	*Myo5a*	1,88286796	3,15e-06
Vasohibin 2	*Vash2*	1,88220488	3,31e-06
Guanine nucleotide binding protein, alpha 14	*Gna14*	1,88183149	0,00016674
Leptin receptor overlapping transcript-like 1	*Leprotl1*	1,88140221	5,26e-05
Triggering receptor expressed on myeloid cells 2	*Trem2*	1,88126401	2,79e-05
Clusterin associated protein 1	*Cluap1*	1,87865722	8,09e-06
Predicted gene 561	*Gm561*	1,87857639	1,64e-05
Cytohesin 1	*Cyth1*	1,87808912	8,90e-06
Ribulose-5-phosphate-3-epimerase	*Rpe*	1,87755326	3,91e-05
Riken cdna 2310061i04 gene	*2310061i04rik*	1,87752043	0,00017109
Special at-rich sequence binding protein 1	*Satb1*	1,8767446	0,00033854
Zinc finger and scan domain containing 26	*Zscan26*	1,87620657	6,16e-05
Riken cdna 9430016h08 gene	*9430016h08rik*	1,87592476	4,52e-05
Expressed sequence c79468	*C79468*	1,87553705	9,57e-05
Trinucleotide repeat containing 6c	*Tnrc6c*	1,87441889	6,16e-05
Sphingomyelin phosphodiesterase 1, acid lysosomal	*Smpd1*	1,87386504	2,74e-06
Carnitine deficiency-associated gene expressed in ventricle 3	*Cdv3*	1,87358895	3,89e-06
Cleavage and polyadenylation specific factor 2	*Cpsf2*	1,87243912	1,32e-05
Reticulocalbin 3, ef-hand calcium binding domain	*Rcn3*	1,87203228	6,22e-06
Leucine-rich repeat-containing g protein-coupled receptor 6	*Lgr6*	1,87191785	0,0001142

Chloride channel 5	*Clcn5*	1,87174038	6,30e-06
Dual serine/threonine and tyrosine protein kinase	*Dstyk*	1,87170824	0,00021367
Adrenergic receptor, beta 2	*Adrb2*	1,87156247	0,00141359
Serine (or cysteine) peptidase inhibitor, clade f, member 1	*Serpinf1*	1,8709312	0,00010729
Bisphosphate 3'-nucleotidase 1	*Bpnt1*	1,86787628	0,00040509
Nima (never in mitosis gene a)-related expressed kinase 6	*Nek6*	1,86713	3,19e-05
Pdz and lim domain 5	*Pdlim5*	1,86650222	3,69e-05
Ccaat/enhancer binding protein (c/ebp), gamma	*Cebpg*	1,86536064	1,81e-05
Discoidin, cub and lccl domain containing 1	*Dcbld1*	1,86529668	1,47e-05
Galactosidase, beta 1-like	*Glb1l*	1,8643815	0,00038509
Lysophosphatidylglycerol acyltransferase 1	*Lpgat1*	1,86432765	5,41e-06
Predicted gene 16340 /// interferon activated gene 203 /// flocculation protein flo11-like	*Gm16340 /// ifi203 /// loc100862473*	1,86416651	0,0008688
Sec16 homolog a (s. Cerevisiae)	*Sec16a*	1,86408865	2,31e-06
Coiled-coil serine rich 2	*Ccser2*	1,86202329	1,60e-05
Phospholipid scramblase 1	*Plscr1*	1,86154583	6,54e-05
Factor 8-associated gene a	*F8a*	1,86033692	8,01e-05
Family with sequence similarity 102, member a	*Fam102a*	1,86017053	5,48e-05
Intermediate filament family orphan 1	*Iffo1*	1,85870504	0,00038508
Related ras viral (r-ras) oncogene homolog 2	*Rras2*	1,85802581	0,00020545
Beta-2 microglobulin	*B2m*	1,85740118	8,47e-06
Heparan sulfate (glucosamine) 3-o-sulfotransferase 1	*Hs3st1*	1,85723606	9,03e-05
Tetratricopeptide repeat domain 13	*Ttc13*	1,85669977	2,30e-06
Tcf3 (e2a) fusion partner	*Tfpt*	1,85653159	0,00140843
Udp-glucose glycoprotein glucosyltransferase 2	*Uggt2*	1,85554195	8,49e-05
Lim domains containing 1	*Limd1*	1,85506911	5,41e-05
Patched homolog 1	*Ptch1*	1,85486794	6,70e-05
Phosphatidylinositol 3-kinase, regulatory subunit, polypeptide 1 (p85 alpha)	*Pik3r1*	1,85475965	7,96e-05
Glucosamine (udp-n-acetyl)-2-epimerase/n-acetylmannosamine kinase	*Gne*	1,85454355	0,00014786
F-box protein 22	*Fbxo22*	1,8539993	1,03e-05
Mitochondrial trans-2-enoyl-coa reductase	*Mecr*	1,85356583	0,00014138
Sorbin and sh3 domain containing 1	*Sorbs1*	1,85283526	8,82e-06
Rho gtpase activating protein 39	*Arhgap39*	1,85258602	9,06e-05
Pinin	*Pnn*	1,85192948	0,00303645

Coiled-coil domain containing 58	*Ccdc58*	1,85186026	4,55e-06
Mitochondrial ribosomal protein l15	*Mrpl15*	1,85044292	4,22e-06
Neural precursor cell expressed, developmentally down-regulated gene 9	*Nedd9*	1,84864534	0,00315988
Tubulin folding cofactor e-like	*Tbcel*	1,84864194	4,76e-05
Protein phosphatase 4, regulatory subunit 2	*Ppp4r2*	1,84715366	1,42e-05
Zinc finger protein 772	*Zfp772*	1,84654622	0,00058675
Atpase, cu++ transporting, alpha polypeptide	*Atp7a*	1,84546566	2,93e-05
Immunoglobulin superfamily containing leucine-rich repeat	*Islr*	1,84444764	0,00095311
Phosphatidylinositol glycan anchor biosynthesis, class n	*Pign*	1,84409844	7,59e-05
Signal transducing adaptor molecule (sh3 domain and itam motif) 1	*Stam*	1,84332543	9,17e-05
Saccharopine dehydrogenase (putative)	*Sccpdh*	1,8429117	0,00048315
Syntaxin 6	*Stx6*	1,84265917	5,52e-06
Carboxypeptidase d	*Cpd*	1,84227762	0,00697106
Casitas b-lineage lymphoma-like 1	*Cbll1*	1,84106779	0,00029412
Histocompatibility 2, t region locus 10 /// histocompatibility 2, t region locus 22 /// histocompatibility 2, t region locus 9	*H2-t10 /// h2-t22 /// h2-t9*	1,84082007	9,47e-06
Mitochondrial fission factor	*Mff*	1,83983293	4,38e-05
Transmembrane protein 140	*Tmem140*	1,83966802	3,13e-05
Carbohydrate (n-acetylgalactosamine 4-0) sulfotransferase 14	*Chst14*	1,83911699	4,65e-06
Gata binding protein 3	*Gata3*	1,8390663	0,00019889
Nephronophthisis 1 (juvenile) homolog (human)	*Nphp1*	1,83774295	2,82e-05
Dead (asp-glu-ala-asp) box polypeptide 10	*Ddx10*	1,837632	0,00029506
Zinc finger protein 955b	*Zfp955b*	1,83745527	0,00027516
Rna polymerase ii associated protein 2	*Rpap2*	1,83685827	5,24e-05
Fucose mutarotase	*Fuom*	1,83593404	1,32e-05
Talin 1	*Tln1*	1,83588629	0,00382046
Orosomucoid 1	*Orm1*	1,8345976	0,00010753
Mitochondrial carrier homolog 2 (c. Elegans)	*Mtch2*	1,83457681	0,0002958
Ring finger and fyve like domain containing protein	*Rffl*	1,83345278	5,99e-05
Fast kinase domains 2	*Fastkd2*	1,83266135	1,29e-05
Oxidoreductase nad-binding domain containing 1	*Oxnad1*	1,83198746	0,00010752
Protein-tyrosine sulfotransferase 2	*Tpst2*	1,82939128	2,26e-06

Tumor necrosis factor receptor superfamily, member 22	*Tnfrsf22*	1,82904944	0,00234243
Solute carrier family 25, member 36	*Slc25a36*	1,82851535	0,00038753
S-adenosylhomocysteine hydrolase-like 2	*Ahcyl2*	1,82755199	9,02e-06
Zinc finger protein x-linked	*Zfx*	1,82707453	3,12e-05
Tetraspanin 12	*Tspan12*	1,82688018	0,00016344
Ubiquitin-conjugating enzyme e2f (putative)	*Ube2f*	1,82616718	1,45e-05
Calcium and integrin binding family member 2	*Cib2*	1,82609522	4,14e-05
Rhotekin 2	*Rtkn2*	1,82549461	0,00010706
Phosphatidylserine decarboxylase, pseudogene 3	*Pisd-ps3*	1,82506731	6,53e-06
Carboxypeptidase x 2 (m14 family)	*Cpxm2*	1,82363822	0,00210494
Riken cdna 4933434e20 gene /// predicted gene 7664	*4933434e20rik /// gm7664*	1,82289942	0,00025207
Phosducin-like	*Pdcl*	1,82262245	0,00020372
Lim motif-containing protein kinase 2	*Limk2*	1,82260956	9,31e-06
Family with sequence similarity 13, member b	*Fam13b*	1,82201376	5,81e-05
Protein phosphatase 1, regulatory (inhibitor) subunit 8	*Ppp1r8*	1,82180715	0,00783975
Aarf domain containing kinase 4	*Adck4*	1,82170795	2,42e-05
Degenerative spermatocyte homolog 1 (drosophila)	*Degs1*	1,82170137	0,00025367
Polymerase (rna) i polypeptide b	*Polr1b*	1,82144937	1,75e-05
Ubx domain protein 2b	*Ubxn2b*	1,82141218	0,00059713
Cdk5 regulatory subunit associated protein 1	*Cdk5rap1*	1,82097184	0,00026947
Branched chain aminotransferase 1, cytosolic	*Bcat1*	1,82064344	3,09e-05
Syntaxin 17	*Stx17*	1,82029433	7,90e-05
Fibronectin 1	*Fn1*	1,82023237	0,00011428
Protein kinase, x-linked	*Prkx*	1,81981683	9,31e-05
Major vault protein	*Mvp*	1,81921022	0,00036285
Asparagine-linked glycosylation 2 (alpha-1,3-mannosyltransferase)	*Alg2*	1,81891788	0,0081993
Biregional cell adhesion molecule-related/down-regulated by oncogenes (cdon) binding protein	*Boc*	1,817702	0,00024448
Eukaryotic translation initiation factor 1a	*Eif1a*	1,81748451	0,00015892
Gtpase activating protein (sh3 domain) binding protein 1	*G3bp1*	1,81725838	4,44e-05
Zinc finger with krab and scan domains 6	*Zkscan6*	1,81562063	3,56e-05
Nischarin	*Nisch*	1,81506545	0,00196462
Cytochrome c oxidase subunit viia polypeptide 2-	*Cox7a2l*	1,81495039	3,69e-06

like			
A disintegrin and metallopeptidase domain 12 (meltrin alpha)	*Adam12*	1,81415906	0,00083349
Family with sequence similarity 122, member a	*Fam122a*	1,81378683	0,00087704
Dehydrogenase/reductase (sdr family) member 3	*Dhrs3*	1,81367905	6,75e-05
Aspartyl aminopeptidase	*Dnpep*	1,81367042	4,18e-06
Eukaryotic translation initiation factor 3, subunit c	*Eif3c*	1,81257343	6,68e-06
Leukemia inhibitory factor receptor	*Lifr*	1,81096424	0,00068855
Hig1 domain family, member 1a	*Higd1a*	1,81069341	0,00013414
Endoplasmic reticulum chaperone sil1 homolog (s. Cerevisiae)	*Sil1*	1,81016832	4,42e-06
Protein phosphatase 1, regulatory (inhibitor) subunit 7	*Ppp1r7*	1,80898532	3,40e-05
Sulfatase modifying factor 1	*Sumf1*	1,80812974	1,49e-05
Armadillo repeat containing 10	*Armc10*	1,80778399	7,73e-06
Xylosyltransferase ii	*Xylt2*	1,80741995	0,00093722
Suppressor apc domain containing 1	*Sapcd1*	1,80727509	0,00020322
Mad2 mitotic arrest deficient-like 2	*Mad2l2*	1,80704001	0,00032962
Heparan-alpha-glucosaminide n-acetyltransferase	*Hgsnat*	1,80688867	1,81e-05
Makorin, ring finger protein, 3	*Mkrn3*	1,80668265	0,00035831
Udp-n-acetyl-alpha-d-galactosamine:polypeptide n-acetylgalactosaminyltransferase 10	*Galnt10*	1,80634677	8,71e-06
Matrix metallopeptidase 11	*Mmp11*	1,80552405	5,03e-05
Lectin, galactoside binding-like	*Lgalsl*	1,8054807	3,11e-05
Myod family inhibitor domain containing	*Mdfic*	1,80479581	0,00011501
Utp6, small subunit (ssu) processome component, homolog (yeast)	*Utp6*	1,8044762	9,15e-06
Nlr family, apoptosis inhibitory protein 1	*Naip1*	1,80424224	0,0001519
Protein phosphatase 3, catalytic subunit, alpha isoform	*Ppp3ca*	1,804229	1,42e-05
Carnitine palmitoyltransferase 2	*Cpt2*	1,80331818	1,00e-05
Cysteine rich transmembrane bmp regulator 1 (chordin like)	*Crim1*	1,80132176	0,00010268
Ga repeat binding protein, beta 1	*Gabpb1*	1,80063343	0,00599417
Ring finger protein 7	*Rnf7*	1,79968767	8,90e-06
Mature t cell proliferation 1	*Mtcp1*	1,79952895	8,71e-06
Sestrin 2	*Sesn2*	1,79946126	0,00070766
Par-3 family cell polarity regulator	*Pard3*	1,79931216	0,00179308

Prolyl endopeptidase-like	*Prepl*	1,79885694	6,99e-06
Guanine nucleotide binding protein, alpha 13	*Gna13*	1,79884035	0,00015361
Udp-gal:betaglcnac beta 1,4-galactosyltransferase, polypeptide 4	*B4galt4*	1,79819451	0,00017091
Aldehyde oxidase 1	*Aox1*	1,79711723	0,00098955
Ethylmalonic encephalopathy 1	*Ethe1*	1,79658025	1,65e-05
Atpase, ca++ transporting, cardiac muscle, slow twitch 2	*Atp2a2*	1,79647409	0,00010901
Retinoblastoma binding protein 9	*Rbbp9*	1,7961037	0,00197014
Sestrin 3	*Sesn3*	1,79490424	6,73e-06
Rho gtpase activating protein 5	*Arhgap5*	1,79483879	9,02e-05
Cytochrome c oxidase assembly factor 5	*Coa5*	1,79398618	1,97e-05
Cytochrome p450, family 27, subfamily a, polypeptide 1	*Cyp27a1*	1,79333547	0,00803329
T cell lymphoma invasion and metastasis 2	*Tiam2*	1,79292947	0,00062397
Phospholipase a2, group v	*Pla2g5*	1,79244675	0,00323344
Lipase maturation factor 1	*Lmf1*	1,7916897	6,53e-05
Arp1 actin-related protein 1b, centractin beta	*Actr1b*	1,79164824	0,00675883
Discs, large homolog 4 (drosophila)	*Dlg4*	1,79079946	0,00082852
N-terminal xaa-pro-lys n-methyltransferase 1	*Ntmt1*	1,79055196	2,34e-05
Pre b cell leukemia homeobox 1	*Pbx1*	1,78915495	0,00024741
Proteasome (prosome, macropain) activator subunit 1 (pa28 alpha)	*Psme1*	1,78867308	5,86e-05
Elk4, member of ets oncogene family	*Elk4*	1,78861744	1,32e-05
Atpase, h+ transporting, lysosomal v1 subunit b2	*Atp6v1b2*	1,78834674	2,97e-05
Arrestin, beta 2	*Arrb2*	1,78783694	0,00186682
Dual-specificity tyrosine-(y)-phosphorylation regulated kinase 3	*Dyrk3*	1,78774332	3,79e-05
Xanthine dehydrogenase	*Xdh*	1,78694504	2,44e-05
Praja ring finger 1, e3 ubiquitin protein ligase	*Pja1*	1,78682968	0,00015253
Guanine nucleotide binding protein-like 2 (nucleolar)	*Gnl2*	1,78680011	0,00030921
Mitochondrial ribosomal protein s34	*Mrps34*	1,78669681	1,16e-05
Glucuronidase, beta	*Gusb*	1,78563022	0,00039128
Peroxiredoxin 5	*Prdx5*	1,78552158	1,45e-05
Tsukushi	*Tsku*	1,7852994	0,00474787
Bbsome interacting protein 1	*Bbip1*	1,78445935	0,00047534
N(alpha)-acetyltransferase 30, natc catalytic subunit	*Naa30*	1,78403966	0,00128437

Homeodomain interacting protein kinase 1	*Hipk1*	1,78338909	9,04e-06
Uncharacterized loc102643023 /// microfibrillar-associated protein 1a /// microfibrillar-associated protein 1b	*Loc102643023 /// mfap1a /// mfap1b*	1,78337887	4,50e-06
Predicted gene 15776 /// guanine nucleotide binding protein (g protein), gamma 5	*Gm15776 /// gng5*	1,78259967	8,52e-06
Angio-associated migratory protein	*Aamp*	1,78234121	5,60e-06
Rosbin, round spermatid basic protein 1	*Rsbn1*	1,78086119	1,42e-05
Galactose-1-phosphate uridyl transferase	*Galt*	1,78058857	0,00011418
Zinc finger, ran-binding domain containing 2	*Zranb2*	1,77948637	3,46e-05
Solute carrier family 35, member a5	*Slc35a5*	1,77943275	4,18e-05
Inhibitor of dna binding 1	*Id1*	1,77930679	0,00028336
Sid1 transmembrane family, member 2	*Sidt2*	1,77858222	2,15e-05
Glycosyltransferase 25 domain containing 1	*Glt25d1*	1,77835577	0,00066438
Ets variant 3	*Etv3*	1,777748	0,0002542
Coenzyme q9 homolog (yeast)	*Coq9*	1,77709783	1,80e-05
Myb/sant-like dna-binding domain containing 4 with coiled-coils	*Msantd4*	1,7760209	0,00025499
Ankyrin repeat domain 40	*Ankrd40*	1,77597872	9,88e-06
Small integral membrane protein 8	*Smim8*	1,77592186	0,00016264
Amyloid beta (a4) precursor-like protein 1	*Aplp1*	1,77568568	0,00437335
Trna methyltransferase 1	*Trmt1*	1,77531822	0,00011634
Transporter 1, atp-binding cassette, sub-family b (mdr/tap)	*Tap1*	1,77459885	9,02e-05
Mpv17 transgene, kidney disease mutant-like	*Mpv17l*	1,77432086	8,20e-05
Rho gtpase activating protein 24	*Arhgap24*	1,77348129	0,0019343
Ef-hand calcium binding domain 2	*Efcab2*	1,77151079	0,00010168
Thrombospondin, type i, domain 1	*Thsd1*	1,77046546	0,00071858
Transcription factor b2, mitochondrial	*Tfb2m*	1,77013125	5,74e-06
Low density lipoprotein receptor class a domain containing 4	*Ldlrad4*	1,76925933	0,0004006
G protein-coupled receptor 137b, pseudogene	*Gpr137b-ps*	1,76908677	1,04e-05
Demethyl-q 7	*Coq7*	1,76770622	8,37e-06
Berardinelli-seip congenital lipodystrophy 2 homolog (seipin)	*Bscl2*	1,76765507	8,69e-05
Actin, alpha 2, smooth muscle, aorta	*Acta2*	1,76467939	0,0001946
Transmembrane protein 62	*Tmem62*	1,76464719	0,0001288
Pseudouridine synthase 1	*Pus1*	1,7640122	1,48e-05

Coactosin-like 1 (dictyostelium)	*Cotl1*	1,76397667	0,00294866
Rfad1, flavin adenine dinucleotide synthetase, homolog (yeast)	*Flad1*	1,76347623	1,83e-05
Methyltransferase like 18	*Mettl18*	1,76315411	2,68e-05
Protein tyrosine phosphatase, mitochondrial 1	*Ptpmt1*	1,76297815	4,23e-05
Rna binding motif protein 43	*Rbm43*	1,76257015	0,00760219
Riken cdna 2610201a13 gene	*2610201a13rik*	1,76248783	5,37e-05
Leucine rich repeat containing 1	*Lrrc1*	1,76123208	0,00010476
Dystroglycan 1	*Dag1*	1,76042878	2,97e-05
Atpase, h+ transporting, lysosomal accessory protein 1	*Atp6ap1*	1,76016897	8,91e-06
Riken cdna 4632428n05 gene	*4632428n05rik*	1,75934085	0,00018801
Peptide deformylase (mitochondrial)	*Pdf*	1,75924988	5,40e-05
Predicted gene 12538 /// orm1-like 3 (s. Cerevisiae)	*Gm12538 /// ormdl3*	1,75841955	1,49e-05
Zinc finger protein 30	*Zfp30*	1,75759168	0,00012548
Cyclin-dependent kinase 9 (cdc2-related kinase)	*Cdk9*	1,7573405	9,59e-06
Histone cluster 2, h2aa2	*Hist2h2aa2*	1,75727845	2,14e-05
Methionyl aminopeptidase type 1d (mitochondrial)	*Metap1d*	1,75726596	5,54e-05
Rar-related orphan receptor alpha	*Rora*	1,75705319	0,00018822
Ph domain and leucine rich repeat protein phosphatase 1	*Phlpp1*	1,75674029	2,30e-05
Zinc finger protein 189	*Zfp189*	1,75668133	0,00021987
Cklf-like marvel transmembrane domain containing 6	*Cmtm6*	1,75667664	0,00024416
Like-glycosyltransferase	*Large*	1,75525205	0,00135264
Zinc finger protein 940	*Zfp940*	1,75476649	0,0039072
Plexin a2	*Plxna2*	1,75475041	3,50e-05
Riken cdna 2700062c07 gene /// predicted gene 9182	*2700062c07rik /// gm9182*	1,75453046	0,00017161
Phosphodiesterase 4b, camp specific	*Pde4b*	1,75449635	0,00064201
Interleukin enhancer binding factor 2	*Ilf2*	1,75435544	0,00013448
Synaptotagmin vii	*Syt7*	1,75361497	7,07e-05
Vasorin	*Vasn*	1,7528329	0,00010363
Snf related kinase	*Snrk*	1,75275646	2,78e-05
Solute carrier family 22, member 23	*Slc22a23*	1,75274832	1,61e-05
Fggy carbohydrate kinase domain containing	*Fggy*	1,75256411	3,68e-05
Granulin	*Grn*	1,75190893	9,42e-06
Kinase suppressor of ras 1	*Ksr1*	1,75115012	0,00146883

Ccaat/enhancer binding protein zeta	*Cebpz*	1,75066836	0,00011025
Tetraspanin 3	*Tspan3*	1,75061077	4,14e-06
Mitogen-activated protein kinase associated protein 1	*Mapkap1*	1,7505302	0,00049273
Zinc finger protein 148	*Zfp148*	1,74983794	0,00010831
Neuron derived neurotrophic factor	*Nenf*	1,74933964	0,00021421
Ribosomal protein s6 kinase polypeptide 1	*Rps6kc1*	1,74885145	0,00055001
Sarcospan	*Sspn*	1,7482252	0,00012518
F-box and wd-40 domain protein 5	*Fbxw5*	1,7475671	0,00471572
Histocompatibility 2, class ii antigen e beta	*H2-eb1*	1,74746935	0,0004935
Tm2 domain containing 2	*Tm2d2*	1,74671256	3,08e-05
Bone morphogenetic protein receptor, type ii (serine/threonine kinase)	*Bmpr2*	1,74665097	0,00224421
Atp-binding cassette, sub-family b (mdr/tap), member 7	*Abcb7*	1,74598937	9,14e-05
Cdna sequence bc002059	*Bc002059*	1,74430177	0,00010696
Imp3, u3 small nucleolar ribonucleoprotein, homolog (yeast)	*Imp3*	1,74359069	2,45e-05
Rna binding motif protein 18	*Rbm18*	1,74356854	3,79e-05
Adp-dependent glucokinase	*Adpgk*	1,74298975	4,81e-05
Interleukin 18	*Il18*	1,74287579	9,74e-06
Protein tyrosine phosphatase, receptor type, g	*Ptprg*	1,74285853	4,71e-05
Male enhanced antigen 1	*Mea1*	1,74235919	0,0001614
F-box protein 16	*Fbxo16*	1,74228159	0,00076844
Mitochondrial ribosomal protein l24	*Mrpl24*	1,74219089	2,77e-05
Chromodomain helicase dna binding protein 1-like	*Chd1l*	1,74086251	0,00011343
Human immunodeficiency virus type i enhancer binding protein 1	*Hivep1*	1,74080273	0,00010882
Heat shock protein 4	*Hspa4*	1,73948344	0,00145685
Ariadne homolog 2 (drosophila)	*Arih2*	1,73942037	7,45e-06
Cdna sequence u90926	*U90926*	1,73802519	0,00696161
Riken cdna 2810474o19 gene	*2810474o19rik*	1,73760827	0,00040371
Yme1-like 1 (s. Cerevisiae)	*Yme1l1*	1,73641234	0,00019225
Src homology 2 domain containing f	*Shf*	1,73638699	0,00112402
Tumor necrosis factor receptor superfamily, member 9	*Tnfrsf9*	1,73586137	2,17e-05
Interferon-related developmental regulator 2	*Ifrd2*	1,73472373	4,60e-05
Ring finger protein, lim domain interacting	*Rlim*	1,73418755	2,08e-05

Transmembrane 7 superfamily member 3	*Tm7sf3*	1,73408138	7,98e-05
Chemokine (c-c motif) ligand 5	*Ccl5*	1,73330443	0,00033082
Serine (or cysteine) peptidase inhibitor, clade b, member 9b	*Serpinb9b*	1,73236347	4,93e-05
Sgt1, suppressor of g2 allele of skp1 (s. Cerevisiae)	*Sugt1*	1,73230952	4,67e-06
Tetraspanin 17	*Tspan17*	1,73210529	3,09e-05
Swa-70 protein	*Swap70*	1,73084219	0,00180087
Ral guanine nucleotide dissociation stimulator-like 3	*Rgl3*	1,73055921	0,00034849
Creb binding protein	*Crebbp*	1,73013646	1,89e-05
Dnaj (hsp40) homolog, subfamily b, member 11	*Dnajb11*	1,7293318	2,97e-05
Exostoses (multiple) 2	*Ext2*	1,7292992	1,23e-05
Isocitrate dehydrogenase 3 (nad+) beta	*Idh3b*	1,72889661	2,61e-05
Kruppel-like factor 7 (ubiquitous)	*Klf7*	1,72865898	0,0002542
Solute carrier family 31, member 1	*Slc31a1*	1,72848062	2,37e-05
Centrosomal protein 19	*Cep19*	1,72759506	0,0004203
Splicing factor 3b, subunit 4	*Sf3b4*	1,72748986	0,00020014
Dynein cytoplasmic 1 light intermediate chain 1	*Dync1li1*	1,72709217	6,86e-06
Low density lipoprotein receptor-related protein 2	*Lrp2*	1,72631858	0,0007696
Swi/snf related, matrix associated, actin dependent regulator of chromatin, subfamily e, member 1	*Smarce1*	1,7260019	0,00046059
Phd finger protein 20	*Phf20*	1,72551099	5,33e-06
Zinc finger protein 26	*Zfp26*	1,72493768	7,06e-05
Cop9 (constitutive photomorphogenic) homolog, subunit 2 (arabidopsis thaliana)	*Cops2*	1,72448817	0,00135188
Map kinase-interacting serine/threonine kinase 1	*Mknk1*	1,72362319	0,0001919
Sh3-domain binding protein 1	*Sh3bp1*	1,72354339	0,00015584
Ww domain containing adaptor with coiled-coil	*Wac*	1,72336508	6,38e-05
Ubiquinol-cytochrome c reductase, complex iii subunit vii	*Uqcrq*	1,72293924	2,57e-05
Kinesin-associated protein 3	*Kifap3*	1,72136311	0,00010371
Family with sequence similarity 188, member a	*Fam188a*	1,72067451	2,33e-05
Misato homolog 1 (drosophila)	*Msto1*	1,7198325	6,82e-05
Glucosamine-6-phosphate deaminase 1 pseudogene /// glucosamine-6-phosphate deaminase 1	*Gm8615 /// gnpda1*	1,71936284	6,22e-05
Fibronectin type iii domain containing 4	*Fndc4*	1,71935555	0,0017778
Peroxisomal biogenesis factor 11 beta	*Pex11b*	1,71868515	0,000348
Nima (never in mitosis gene a)-related expressed kinase 3	*Nek3*	1,71815112	0,0002211

Yth domain family 1	*Ythdf1*	1,71746141	2,73e-05
Leucine rich repeat containing 58	*Lrrc58*	1,7165575	0,00167796
Dna-damage-inducible transcript 4	*Ddit4*	1,71594491	0,00012549
Riken cdna 0610009b22 gene	*0610009b22rik*	1,71492486	7,94e-05
Seven in absentia 2	*Siah2*	1,7145363	2,14e-05
Riken cdna 1190005i06 gene	*1190005i06rik*	1,71422912	9,73e-05
Solute carrier family 7 (cationic amino acid transporter, y+ system), member 5	*Slc7a5*	1,71412702	0,00089199
Cerebral cavernous malformation 2	*Ccm2*	1,71391805	7,86e-05
Stam binding protein like 1	*Stambpl1*	1,71363205	3,28e-05
Membrane-spanning 4-domains, subfamily a, member 4b	*Ms4a4b*	1,71325662	0,00034511
Prp31 pre-mrna processing factor 31 homolog (yeast)	*Prpf31*	1,71266338	9,60e-05
Testis specific protein kinase 1	*Tesk1*	1,71204496	8,00e-05
Herpud family member 2	*Herpud2*	1,71204479	7,69e-05
Clip associating protein 1	*Clasp1*	1,71156251	2,21e-05
Poliovirus receptor-related 1	*Pvrl1*	1,71116937	0,00263603
Integrin alpha v	*Itgav*	1,71090074	1,19e-05
Basic leucine zipper and w2 domains 1	*Bzw1*	1,7100609	0,00175769
E26 avian leukemia oncogene 2, 3' domain	*Ets2*	1,70988922	2,71e-05
Phosphotriesterase related	*Pter*	1,70968061	0,00034723
Tetratricopeptide repeat, ankyrin repeat and coiled-coil containing 1	*Tanc1*	1,70948955	8,71e-06
Dnaj (hsp40) homolog, subfamily a, member 2	*Dnaja2*	1,70885555	2,23e-05
Asparaginyl-trna synthetase	*Nars*	1,70881097	9,66e-06
Ring finger protein 113a1	*Rnf113a1*	1,70733338	3,83e-05
Fc receptor, ige, low affinity ii, alpha polypeptide	*Fcer2a*	1,70728343	0,00106069
F-box and leucine-rich repeat protein 8	*Fbxl8*	1,7072213	0,00120496
Rho guanine nucleotide exchange factor (gef) 25	*Arhgef25*	1,70667041	0,00029909
Lysosomal-associated membrane protein 2	*Lamp2*	1,70656643	6,50e-06
Serum response factor binding protein 1	*Srfbp1*	1,7062255	0,00013227
Nme/nm23 family member 5	*Nme5*	1,70589096	0,00526766
Suppressor of ty 20	*Supt20*	1,70533274	0,00072923
Myb/sant-like dna-binding domain containing 3	*Msantd3*	1,70455235	0,00037947
Poly(rc) binding protein 3	*Pcbp3*	1,70448794	0,00087678
Potassium voltage-gated channel, subfamily q, member 5	*Kcnq5*	1,70428287	0,00040488
Zinc finger protein 68	*Zfp68*	1,70422451	0,00021139

Casein kinase 1, gamma 1	*Csnk1g1*	1,70413785	9,66e-05
Leucine rich repeat containing 42	*Lrrc42*	1,70282261	0,00039132
Tubulointerstitial nephritis antigen-like 1	*Tinagl1*	1,7027364	0,0033596
Laminin, gamma 1	*Lamc1*	1,70265319	0,00116435
Esf1, nucleolar pre-rrna processing protein, homolog (s. Cerevisiae)	*Esf1*	1,70247735	4,14e-05
Asparagine-linked glycosylation 1 (beta-1,4-mannosyltransferase)	*Alg1*	1,70152431	0,00022947
Zinc finger protein 777	*Zfp777*	1,70140251	4,22e-05
Histone cluster 1, h4a /// histone cluster 1, h4b /// histone cluster 1, h4c /// histone cluster 1, h4d /// histone cluster 1, h4f /// histone cluster 1, h4h /// histone cluster 1, h4i /// histone cluster 1, h4j /// histone cluster 1, h4k /// histone cluster 1, h4m /// histone cluster 1, h4n /// histone cluster 2, h4 /// histone cluster 4, h4	*Hist1h4a /// hist1h4b /// hist1h4c /// hist1h4d /// hist1h4f /// hist1h4h /// hist1h4i /// hist1h4j /// hist1h4k /// hist1h4m /// hist1h4n /// hist2h4 /// hist4h4*	1,7013617	0,00017967
Sorting nexin 16	*Snx16*	1,70041033	4,62e-05
Traf family member-associated nf-kappa b activator	*Tank*	1,69848508	0,00827998
Short chain dehydrogenase/reductase family 42e, member 1	*Sdr42e1*	1,69818663	0,00045725
Neutrophil cytosolic factor 4	*Ncf4*	1,69812586	0,00064446
Retinol dehydrogenase 9	*Rdh9*	1,69785976	5,03e-05
Enoyl-coenzyme a, hydratase/3-hydroxyacyl coenzyme a dehydrogenase	*Ehhadh*	1,69781774	0,00028296
Centrosomal protein 68	*Cep68*	1,69780775	1,54e-05
Transmembrane protein with metallophosphoesterase domain	*Tmppe*	1,69773327	0,00165421
Transducin-like enhancer of split 1, homolog of drosophila e(spl)	*Tle1*	1,69762907	2,62e-05
Zinc finger protein 82	*Zfp82*	1,69689278	0,00024444
Forkhead box o3	*Foxo3*	1,69685754	2,12e-05
Deah (asp-glu-ala-his) box polypeptide 32	*Dhx32*	1,69622242	2,09e-05
Monoglyceride lipase	*Mgll*	1,69567867	0,00037891
Crystallin, lambda 1	*Cryl1*	1,69509024	4,64e-05
Inositol 1,4,5-triphosphate receptor 2	*Itpr2*	1,69439739	0,00054641
Myoneurin	*Mynn*	1,69420899	0,00489794
Glycogen synthase 1, muscle	*Gys1*	1,69419032	0,00423417

Hyaluronic acid binding protein 4	*Habp4*	1,69416908	9,07e-05
Atp-binding cassette, sub-family a (abc1), member 2	*Abca2*	1,69360597	0,00088922
Tropomyosin 1, alpha	*Tpm1*	1,6929794	0,00218845
Transforming growth factor, beta receptor i	*Tgfbr1*	1,69253346	0,00053227
Rhophilin, rho gtpase binding protein 2	*Rhpn2*	1,69219317	0,00126234
Tetratricopeptide repeat domain 7b	*Ttc7b*	1,69200743	0,00345784
Leucine rich repeat (in flii) interacting protein 1	*Lrrfip1*	1,69197251	0,00085105
R3h domain and coiled-coil containing 1	*R3hcc1*	1,6917971	0,0001015
Nedd4 family interacting protein 1	*Ndfip1*	1,69156121	1,21e-05
Riken cdna 2510039o18 gene	*2510039o18rik*	1,69146332	9,18e-05
Mckusick-kaufman syndrome	*Mkks*	1,69130934	0,00085799
Deah (asp-glu-ala-his) box polypeptide 40	*Dhx40*	1,69114088	1,00e-05
Ring finger protein 135	*Rnf135*	1,69113496	0,00183379
Enhancer of yellow 2 homolog (drosophila)	*Eny2*	1,690676	0,00014138
Pyruvate dehydrogenase (lipoamide) beta	*Pdhb*	1,68996332	0,00545462
Eukaryotic translation initiation factor 2, subunit 2 (beta)	*Eif2s2*	1,68849256	9,28e-06
Minichromosome maintenance deficient 3 (s. Cerevisiae) associated protein	*Mcm3ap*	1,68825168	0,0001005
Phosphatidylinositol transfer protein, membrane-associated 1	*Pitpnm1*	1,68758188	6,58e-05
Transmembrane protein 45a	*Tmem45a*	1,68592715	0,00032962
Cullin 3	*Cul3*	1,68515387	0,00016264
Atpase, ca++-sequestering	*Atp2c1*	1,68515336	0,00792987
N-ethylmaleimide sensitive fusion protein attachment protein beta	*Napb*	1,68508292	0,00789226
Fibroblast growth factor (acidic) intracellular binding protein	*Fibp*	1,68470035	5,91e-05
Dolichyl-phosphate (udp-n-acetylglucosamine) acetylglucosaminephosphotransferase 1 (glcnac-1-p transferase)	*Dpagt1*	1,68454369	1,45e-05
Nadh dehydrogenase (ubiquinone) fe-s protein 2	*Ndufs2*	1,68431027	2,65e-05
Spermatogenesis associated 5	*Spata5*	1,6832105	2,25e-05
Insulin induced gene 2	*Insig2*	1,68228942	9,12e-05
Rna binding motif protein 39	*Rbm39*	1,68154491	0,00014434
Integrin alpha fg-gap repeat containing 1	*Itfg1*	1,68124273	0,00344739
Protein tyrosine phosphatase, receptor type, o	*Ptpro*	1,68083494	8,25e-05
Alcohol dehydrogenase 7 (class iv), mu or sigma	*Adh7*	1,68052465	0,00418478

polypeptide			
Transglutaminase 2, c polypeptide	*Tgm2*	1,68049094	5,94e-05
Zinc finger protein 191	*Zfp191*	1,68008871	0,00223007
Ras p21 protein activator 2	*Rasa2*	1,67983535	0,00191318
Growth factor independent 1	*Gfi1*	1,67939746	9,61e-05
Carboxypeptidase q	*Cpq*	1,67891193	1,38e-05
Zinc finger protein 65 /// zinc finger protein 738	*Zfp65 /// zfp738*	1,67879186	0,00046119
Transducin-like enhancer of split 3, homolog of drosophila e(spl)	*Tle3*	1,67869063	0,00650527
Oviductal glycoprotein 1	*Ovgp1*	1,67755857	0,00126305
Zinc finger with krab and scan domains 1	*Zkscan1*	1,67753933	4,43e-05
Pleckstrin homology domain containing, family b (evectins) member 2	*Plekhb2*	1,67726657	0,00038275
Zinc finger protein 106	*Zfp106*	1,67704166	2,12e-05
Harvey rat sarcoma oncogene, subgroup r	*Rras*	1,67690405	6,71e-05
Histocompatibility 2, class ii, locus dma	*H2-dma*	1,67631424	0,00059713
Succinate dehydrogenase complex, subunit c, integral membrane protein	*Sdhc*	1,67598716	0,006106
Mitochondrial ribosomal protein l1	*Mrpl1*	1,67570982	5,86e-06
St3 beta-galactoside alpha-2,3-sialyltransferase 5	*St3gal5*	1,67492313	0,00026657
Sequestosome 1	*Sqstm1*	1,67473504	6,78e-06
Kelch-like 22	*Klhl22*	1,67466915	5,48e-05
Zinc finger protein of the cerebellum 4	*Zic4*	1,67383533	0,00017598
Lin-7 homolog c (c. Elegans)	*Lin7c*	1,67381135	0,00463563
Sumo/sentrin specific peptidase 6	*Senp6*	1,67352806	0,00074601
Udp-glcnac:betagal beta-1,3-n-acetylglucosaminyltransferase 1	*B3gnt1*	1,67329609	0,00026135
Growth arrest specific 2	*Gas2*	1,67329211	0,0024417
Jumping translocation breakpoint	*Jtb*	1,67299518	1,35e-05
A kinase (prka) anchor protein 17b	*Akap17b*	1,67295961	0,00023079
Suppressor of variegation 4-20 homolog 1 (drosophila)	*Suv420h1*	1,6729011	0,00014358
Proteasome (prosome, macropain) assembly chaperone 4	*Psmg4*	1,67260858	9,95e-05
Protocadherin 10	*Pcdh10*	1,67182839	0,00766757
Riken cdna 1700021f05 gene	*1700021f05rik*	1,67101487	3,36e-05
Phd finger protein 21a	*Phf21a*	1,67088778	0,00034049
Corepressor interacting with rbpj, 1	*Cir1*	1,67066321	0,00044372

Interleukin 33	*Il33*	1,67057624	1,61e-05
G-protein signalling modulator 1 (ags3-like, c. Elegans)	*Gpsm1*	1,67019335	0,00088559
U7 snrnp-specific sm-like protein lsm10	*Lsm10*	1,66988478	2,65e-05
Hemk methyltransferase family member 1	*Hemk1*	1,6690779	9,91e-05
Mannoside acetylglucosaminyltransferase 4, isoenzyme b	*Mgat4b*	1,66848064	0,00014623
Autism susceptibility candidate 2	*Auts2*	1,66788415	0,0030599
Cartilage associated protein	*Crtap*	1,66775162	1,99e-05
U2 small nuclear ribonucleoprotein b	*Snrpb2*	1,66715056	0,00158215
Small edrk-rich factor 1	*Serf1*	1,66703768	0,00011033
Vacuolar protein sorting 4b (yeast)	*Vps4b*	1,6661104	0,00019843
Lectin, mannose-binding 2	*Lman2*	1,66578228	0,00020694
Glutaredoxin 2 (thioltransferase)	*Glrx2*	1,66504897	3,32e-05
Fukutin	*Fktn*	1,6647452	0,00079949
Glutamyl-prolyl-trna synthetase	*Eprs*	1,66459355	3,98e-05
Rna binding motif protein 15	*Rbm15*	1,66448265	0,00078092
Mannosidase 2, alpha b1	*Man2b1*	1,66379814	2,53e-05
Poly (adp-ribose) polymerase family, member 3	*Parp3*	1,66338798	9,03e-05
Myeloid/lymphoid or mixed-lineage leukemia (trithorax homolog, drosophila); translocated to, 11	*Mllt11*	1,66299565	0,00010472
Olfactomedin 1	*Olfm1*	1,662806	0,00061003
Cdna sequence bc003331	*Bc003331*	1,66262287	0,00014205
Neuropilin 1	*Nrp1*	1,66257163	0,00022227
Retinitis pigmentosa gtpase regulator	*Rpgr*	1,66241696	0,00100536
Pescadillo homolog 1, containing brct domain (zebrafish)	*Pes1*	1,66202114	0,00024726
Serum amyloid a 1	*Saa1*	1,66200977	0,00021895
Propionyl-coenzyme a carboxylase, alpha polypeptide	*Pcca*	1,66121759	5,98e-05
Ras suppressor protein 1	*Rsu1*	1,66121348	5,15e-05
Nuclear receptor co-repressor 1	*Ncor1*	1,660688	6,08e-05
Thioredoxin interacting protein	*Txnip*	1,6603891	6,25e-05
Dimethylarginine dimethylaminohydrolase 2	*Ddah2*	1,65984188	9,41e-05
Small proline-rich protein 2h	*Sprr2h*	1,65972739	0,00019227
Expressed sequence ai413582	*Ai413582*	1,65895067	0,00165405
Thymocyte nuclear protein 1	*Thyn1*	1,6586819	1,54e-05
Programmed cell death 4	*Pdcd4*	1,65857423	2,87e-05

Platelet derived growth factor, alpha	*Pdgfa*	1,65780994	1,97e-05
Haus augmin-like complex, subunit 2	*Haus2*	1,65779878	0,00044208
Transmembrane protein 203	*Tmem203*	1,65757683	0,00012417
Nuclear factor of kappa light polypeptide gene enhancer in b cells inhibitor, epsilon	*Nfkbie*	1,6571846	0,0008499
Gtp binding protein 2	*Gtpbp2*	1,65716441	0,00063618
Camp-regulated phosphoprotein 19	*Arpp19*	1,65708592	6,10e-05
Low-density lipoprotein receptor-related protein 10	*Lrp10*	1,65703737	0,00044944
Enoyl-coenzyme a delta isomerase 2	*Eci2*	1,65696705	3,00e-05
Killer cell lectin-like receptor subfamily g, member 1	*Klrg1*	1,65639945	0,0005005
Zinc finger protein 426	*Zfp426*	1,65605991	6,44e-05
Kti12 homolog, chromatin associated (s. Cerevisiae)	*Kti12*	1,65602875	0,00018153
Family with sequence similarity 173, member a	*Fam173a*	1,65601345	2,72e-05
Inhibitor of growth family, member 1	*Ing1*	1,65598297	1,46e-05
Alcohol dehydrogenase 5 (class iii), chi polypeptide	*Adh5*	1,65533409	4,09e-05
Zinc finger ccch type containing 12a	*Zc3h12a*	1,65514955	0,00387369
Mitogen-activated protein kinase 6	*Mapk6*	1,65473598	0,00275052
Riken cdna 4930453n24 gene	*4930453n24rik*	1,65392046	2,46e-05
Signal peptidase complex subunit 1 homolog (s. Cerevisiae)	*Spcs1*	1,65348557	0,00014471
B cell translocation gene 2, anti-proliferative	*Btg2*	1,65259581	0,00052108
Signal recognition particle receptor, b subunit	*Srprb*	1,65228533	0,00836148
Prokineticin receptor 1	*Prokr1*	1,65221878	0,00019762
Lysine-rich coiled-coil 1	*Krcc1*	1,64944902	0,00783068
Nadh dehydrogenase (ubiquinone) fe-s protein 1	*Ndufs1*	1,64923917	0,00022429
Coiled-coil domain containing 86	*Ccdc86*	1,64821902	0,00039479
Ufm1-specific peptidase 1	*Ufsp1*	1,64817757	0,00021987
Pet112 homolog (s. Cerevisiae)	*Pet112*	1,64775139	0,00070305
Er degradation enhancer, mannosidase alpha-like 3	*Edem3*	1,6470641	8,54e-05
Breast cancer anti-estrogen resistance 3	*Bcar3*	1,646697	6,06e-05
Set domain containing 6	*Setd6*	1,64661711	0,00024488
Cyclin-dependent kinase 7	*Cdk7*	1,64614588	0,0002902
Membrane-associated ring finger (c3hc4) 7	*Iii.07*	1,64564955	0,00027514
Zinc finger protein 955a	*Zfp955a*	1,64562851	0,00018976
Transmembrane protein 245	*Tmem245*	1,64562774	5,25e-05
Run and sh3 domain containing 2	*Rusc2*	1,64522886	0,00016083
Heat shock protein 1 (chaperonin 10)	*Hspe1*	1,64506706	8,03e-05
Cullin 9	*Cul9*	1,64454443	0,00039902

Family with sequence similarity 134, member a	*Fam134a*	1,64450244	2,68e-05
Ras homolog gene family, member g	*Rhog*	1,64439299	0,00033229
Tripartite motif-containing 32	*Trim32*	1,64395766	0,00071844
Pyrophosphatase (inorganic) 2	*Ppa2*	1,64350081	5,06e-05
Hairy and enhancer of split 6	*Hes6*	1,64196116	0,00059526
Spry domain containing 4	*Spryd4*	1,64175267	0,00010627
Vacuolar protein sorting 18 (yeast)	*Vps18*	1,64105318	6,82e-05
Cdna sequence bc048403	*Bc048403*	1,64064091	0,00022333
Glypican 4	*Gpc4*	1,64043142	3,38e-05
Kinesin family member 3b	*Kif3b*	1,64001818	0,00019963
Signal recognition particle 14	*Srp14*	1,63969852	2,69e-05
Protein sfi1 homolog /// sfi1 homolog, spindle assembly associated (yeast)	*Loc100861749 /// sfi1*	1,63919943	0,0001145
Ubiquitin-conjugating enzyme e2d 2a	*Ube2d2a*	1,63868999	1,14e-05
N-myristoyltransferase 1	*Nmt1*	1,63813482	3,25e-05
Dcn1, defective in cullin neddylation 1, domain containing 1 (s. Cerevisiae)	*Dcun1d1*	1,63781016	0,00075176
Transmembrane protein 205	*Tmem205*	1,63735356	8,74e-05
Ribosomal protein s24	*Rps24*	1,63663996	0,00337027
Trafficking protein particle complex 6a	*Trappc6a*	1,63646037	3,20e-05
Cysteine-rich transmembrane module containing 1	*Cystm1*	1,63629896	0,00034511
Neuropeptide y receptor y1	*Npy1r*	1,6360367	0,00011165
Uri1, prefoldin-like chaperone	*Uri1*	1,6352951	0,00018976
Transmembrane protein 209	*Tmem209*	1,63386937	0,00265477
Active bcr-related gene	*Abr*	1,63341946	4,20e-05
Zinc finger protein 455	*Zfp455*	1,63330676	0,0043561
Btb (poz) domain containing 1	*Btbd1*	1,63315371	2,68e-05
Phosphatidic acid phosphatase type 2c	*Ppap2c*	1,63305384	1,67e-05
Renin binding protein	*Renbp*	1,6329074	4,37e-05
Ankyrin repeat and zinc finger domain containing 1	*Ankzf1*	1,63257105	2,81e-05
Tripartite motif-containing 21	*Trim21*	1,63156686	0,0001004
Plakophilin 3	*Pkp3*	1,63074463	0,00048876
Glioblastoma amplified sequence	*Gbas*	1,63069086	0,00087915
Acid phosphatase 6, lysophosphatidic	*Acp6*	1,63041618	5,53e-05
Tnf receptor-associated factor 5	*Traf5*	1,63004899	3,97e-05
Cyclin c	*Ccnc*	1,6299198	0,00940912
Tribbles homolog 2 (drosophila)	*Trib2*	1,62935617	0,00023761
Cold shock domain containing e1, rna binding	*Csde1*	1,62922842	5,45e-05

Aprataxin	*Aptx*	1,62906858	0,00023738
Lactamase, beta	*Lactb*	1,62843702	0,0006036
Phosphatidylserine decarboxylase /// phosphatidylserine decarboxylase, pseudogene 1 /// phosphatidylserine decarboxylase, pseudogene 3	*Pisd /// pisd-ps1 /// pisd-ps3*	1,62834179	2,90e-05
Alpha-n-acetylglucosaminidase (sanfilippo disease iiib)	*Naglu*	1,6282772	0,00031434
Solute carrier family 26, member 6	*Slc26a6*	1,62800821	0,00095432
Transducer of erbb-2.1	*Tob1*	1,62732519	0,0091882
Protein phosphatase 2, regulatory subunit b, alpha	*Ppp2r2a*	1,62692791	4,38e-05
Adp-ribose/cdp-alcohol diphosphatase, manganese dependent	*Adprm*	1,62688332	0,00080273
Wd repeat domain 26	*Wdr26*	1,62685157	0,00218502
Cystinosis, nephropathic	*Ctns*	1,62682997	0,00038699
Orai calcium release-activated calcium modulator 1	*Orai1*	1,62613911	0,00415459
Selenophosphate synthetase 1	*Sephs1*	1,62546048	0,00185763
Dihydrolipoamide s-acetyltransferase (e2 component of pyruvate dehydrogenase complex)	*Dlat*	1,62492897	0,00110392
1-acylglycerol-3-phosphate o-acyltransferase 3	*Agpat3*	1,62489811	9,82e-05
Poliovirus receptor-related 3	*Pvrl3*	1,62445915	0,00299908
Sperm specific antigen 2	*Ssfa2*	1,62424531	0,0001612
Isoleucine-trna synthetase 2, mitochondrial	*Iars2*	1,62400669	0,00016586
Mitochondrial fission process 1	*Mtfp1*	1,62393252	3,75e-05
Epithelial membrane protein 2	*Emp2*	1,62309622	0,00208966
Mitochondrial methionyl-trna formyltransferase	*Mtfmt*	1,62257456	0,00055208
Ecto-nox disulfide-thiol exchanger 2	*Enox2*	1,62255862	0,0019343
Acyl-coenzyme a binding domain containing 6	*Acbd6*	1,62105058	0,00023039
Superoxide dismutase 1, soluble	*Sod1*	1,62086893	0,00026641
Nlr family member x1	*Nlrx1*	1,62051515	0,00088747
Ngfi-a binding protein 1	*Nab1*	1,61886014	0,00660289
Monoamine oxidase a	*Maoa*	1,61884022	2,38e-05
Single immunoglobulin and toll-interleukin 1 receptor (tir) domain	*Sigirr*	1,61825184	8,09e-05
Protein tyrosine phosphatase, non-receptor type 4	*Ptpn4*	1,618215	0,00083623
Phosphoribosylglycinamide formyltransferase	*Gart*	1,61795968	3,53e-05
Uhrf1 (icbp90) binding protein 1	*Uhrf1bp1*	1,61730256	0,00011258
Notch 3	*Notch3*	1,61715522	0,00210465
Eukaryotic translation initiation factor 4e binding	*Eif4ebp1*	1,61711913	8,95e-05

protein 1			
Zinc finger protein 207	*Zfp207*	1,61703591	0,00602624
Tyrosyl-trna synthetase	*Yars*	1,6168948	2,71e-05
Wilms tumour 1-associating protein	*Wtap*	1,61623773	6,02e-05
Solute carrier family 33 (acetyl-coa transporter), member 1	*Slc33a1*	1,61601052	0,00223585
Leucine-rich ppr-motif containing	*Lrpprc*	1,61556128	0,00023654
Serine/threonine/tyrosine interaction protein	*Styx*	1,61493198	0,00131005
Smad family member 4	*Smad4*	1,61453659	9,39e-05
Hiv tat specific factor 1	*Htatsf1*	1,61397481	8,09e-05
Ergic and golgi 3	*Ergic3*	1,61371141	0,00015262
Riken cdna 3110043o21 gene	*3110043o21rik*	1,61318721	0,00029425
Transmembrane protein 218	*Tmem218*	1,61286579	0,0009961
Zinc finger protein 959	*Zfp959*	1,6124552	0,00094959
Death effector domain-containing	*Dedd*	1,61199771	0,00033924
S100p binding protein	*S100pbp*	1,61140527	0,00135376
Butyrophilin, subfamily 1, member a1	*Btn1a1*	1,61060434	0,00173407
Zinc finger protein 445	*Zfp445*	1,61041997	0,00054515
Ribosomal protein s20	*Rps20*	1,61032801	0,00064732
Hydroxysteroid dehydrogenase like 2	*Hsdl2*	1,60989575	0,00084484
Breast cancer anti-estrogen resistance 1	*Bcar1*	1,60941743	0,00015524
Activity regulated cytoskeletal-associated protein	*Arc*	1,60913752	0,00167217
Forkhead box c2	*Foxc2*	1,60878408	0,00144955
Plexin a3	*Plxna3*	1,60844577	3,52e-05
Calmodulin regulated spectrin-associated protein 1	*Camsap1*	1,60800496	7,59e-05
Trub pseudouridine (psi) synthase homolog 2 (e. Coli)	*Trub2*	1,60760357	0,00012661
Riken cdna 1110054m08 gene	*1110054m08rik*	1,60724927	0,00775427
Bcl2-associated athanogene 5	*Bag5*	1,60692305	8,32e-05
Wntless homolog (drosophila)	*Wls*	1,6066287	3,84e-05
Las1-like (s. Cerevisiae)	*Las1l*	1,6057998	5,36e-05
Transformation related protein 53 binding protein 1	*Trp53bp1*	1,60552099	0,00010805
Cd151 antigen	*Cd151*	1,60517455	4,80e-05
Prickle homolog 4 (drosophila) /// translocase of outer mitochondrial membrane 6 homolog (yeast)	*Prickle4 /// tomm6*	1,60493042	0,00906764
Regulator of g-protein signaling 19	*Rgs19*	1,60379967	0,00055891
Protein tyrosine phosphatase-like (proline instead of catalytic arginine), member b	*Ptplb*	1,60334459	0,00020832

Asteroid homolog 1 (drosophila)	*Aste1*	1,60258297	0,0003394
Guanylate kinase 1	*Guk1*	1,60240841	0,00016042
Sulfite oxidase	*Suox*	1,60153437	2,65e-05
Rio kinase 3	*Riok3*	1,6013518	0,0001214
Angiotensin ii, type i receptor-associated protein	*Agtrap*	1,60118062	0,00051421
Dual specificity phosphatase 13	*Dusp13*	1,60051783	0,00019796
Heme binding protein 2	*Hebp2*	1,60032344	0,00085632
Udp-glucose glycoprotein glucosyltransferase 1	*Uggt1*	1,60016999	0,00090748
Hras-like suppressor	*Hrasls*	1,59968398	0,00062142
Dicer 1, ribonuclease type iii	*Dicer1*	1,59856677	0,00010969
Coiled-coil-helix-coiled-coil-helix domain containing 5	*Chchd5*	1,59829953	0,00018622
At hook containing transcription factor 1	*Ahctf1*	1,59800347	0,00020104
Dead (asp-glu-ala-asp) box polypeptide 24	*Ddx24*	1,59794708	2,60e-05
Ring finger protein 44	*Rnf44*	1,59751709	0,0001026
Brx1, biogenesis of ribosomes, homolog (s. Cerevisiae)	*Brix1*	1,59725363	0,00040063
Riken cdna 1700123o20 gene	*1700123o20rik*	1,59715748	0,00017388
Atypical chemokine receptor 3	*Ackr3*	1,59669983	0,00036519
Zinc finger protein 202	*Zfp202*	1,59625952	6,72e-05
Wd repeat domain 75	*Wdr75*	1,59602543	3,37e-05
Pregnancy upregulated non-ubiquitously expressed cam kinase	*Pnck*	1,59580745	0,00285236
Nima (never in mitosis gene a)-related expressed kinase 7	*Nek7*	1,59521461	0,00012737
General transcription factor ii i repeat domain-containing 1	*Gtf2ird1*	1,59468566	0,00019829
Atpase, na+/k+ transporting, beta 3 polypeptide	*Atp1b3*	1,59457328	0,00032528
Cell adhesion molecule 4	*Cadm4*	1,59385664	0,00112655
Nuclear receptor coactivator 4	*Ncoa4*	1,59371726	0,00134629
Gid complex subunit 4, vid24 homolog (s. Cerevisiae)	*Gid4*	1,59370386	0,00127126
Glutamate receptor, ionotropic, kainate 5 (gamma 2)	*Grik5*	1,59364143	0,00137815
Matrix metallopeptidase 12	*Mmp12*	1,59336148	0,00126663
Leucine zipper protein 1	*Luzp1*	1,59294852	0,00084351
Glypican 1	*Gpc1*	1,59238461	2,43e-05
Secretory carrier membrane protein 3	*Scamp3*	1,59201436	1,77e-05
Lim domain only 4	*Lmo4*	1,59161726	5,34e-05

Peptidylglycine alpha-amidating monooxygenase	*Pam*	1,59111006	0,00460184
Atpase, h+ transporting, lysosomal v1 subunit f	*Atp6v1f*	1,59082359	7,39e-05
Phosphorylated adaptor for rna export	*Phax*	1,59076085	0,00165433
Lim and senescent cell antigen like domains 2	*Lims2*	1,59060915	0,00257224
Deah (asp-glu-ala-his) box polypeptide 36	*Dhx36*	1,59004201	0,00028347
Serta domain containing 2	*Sertad2*	1,58994529	2,81e-05
Serine racemase	*Srr*	1,58988138	7,23e-05
Multiple inositol polyphosphate histidine phosphatase 1	*Minpp1*	1,58918363	0,00154933
Zinc finger protein 768	*Zfp768*	1,5887981	0,00089716
Cullin 2	*Cul2*	1,58842593	0,00158779
M phase phosphoprotein 6	*Mphosph6*	1,58804225	9,39e-05
Leucyl-trna synthetase	*Lars*	1,58772261	3,99e-05
Acyl-coenzyme a dehydrogenase, medium chain	*Acadm*	1,58740644	3,86e-05
Mus musculus zinc finger protein 697 (zfp697), mrna.	*Zfp697*	1,58731838	0,00070048
Dystrobrevin alpha	*Dtna*	1,58700068	6,92e-05
Interleukin 11	*Il11*	1,5866137	0,00036709
F-box and wd-40 domain protein 11	*Fbxw11*	1,58578674	9,70e-05
Ephrin a4	*Efna4*	1,58549751	0,00069195
Gem (nuclear organelle) associated protein 7	*Gemin7*	1,58484746	0,00078119
Telomeric repeat binding factor 2, interacting protein	*Terf2ip*	1,58447059	0,00162572
Poly (adp-ribose) polymerase family, member 10 /// plectin	*Parp10 /// plec*	1,58438561	0,00236934
Musashi rna-binding protein 1	*Msi1*	1,58391308	0,00096983
Mitochondrial ribosomal protein l39	*Mrpl39*	1,58377168	0,00010445
Deah (asp-glu-ala-his) box polypeptide 30	*Dhx30*	1,58334322	0,00019478
Zyg-ll family member b, cell cycle regulator	*Zyg11b*	1,58303731	0,00451346
Latent transforming growth factor beta binding protein 1	*Ltbp1*	1,58251494	0,00038133
Tata box binding protein (tbp)-associated factor, rna polymerase i, c	*Taf1c*	1,58240081	9,14e-05
Solute carrier family 22 (organic cation transporter), member 5	*Slc22a5*	1,58180361	0,00020322
Rab14, member ras oncogene family	*Rab14*	1,58139837	0,00115844
R3h domain containing 1	*R3hdm1*	1,58103261	5,03e-05
Slu7 splicing factor homolog (s. Cerevisiae)	*Slu7*	1,58055626	0,00054025
Protocadherin alpha 1 /// protocadherin alpha 10 ///	*Pcdha1 /// pcdha10*	1,58046509	0,00016674

protocadherin alpha 11 /// protocadherin alpha 12 /// protocadherin alpha 2 /// protocadherin alpha 3 /// protocadherin alpha 4 /// protocadherin alpha 5 /// protocadherin alpha 6 /// protocadherin alpha 7 /// protocadherin alpha 8 /// protocadherin alpha 9 /// protocadherin alpha subfamily c, 1 /// protocadherin alpha subfamily c, 2	*/// pcdha11 /// pcdha12 /// pcdha2 /// pcdha3 /// pcdha4 /// pcdha5 /// pcdha6 /// pcdha7 /// pcdha8 /// pcdha9 /// pcdhac1 /// pcdhac2*		
Transmembrane protein 135	*Tmem135*	1,58014179	0,00044312
Procollagen-lysine, 2-oxoglutarate 5-dioxygenase 3	*Plod3*	1,57998676	8,08e-05
Oxysterol binding protein-like 11	*Osbpl11*	1,5796321	0,00058662
Melanoma antigen, family d, 2	*Maged2*	1,57925343	2,71e-05
Large tumor suppressor 2	*Lats2*	1,57860158	0,00053282
Zinc finger with krab and scan domains 14	*Zkscan14*	1,57841349	0,00053003
Spermatogenesis associated 6	*Spata6*	1,57785282	0,001489
Rna terminal phosphate cyclase-like 1	*Rcl1*	1,57572451	1,69e-05
Proline-serine-threonine phosphatase-interacting protein 2	*Pstpip2*	1,5757237	0,00046938
Asparagine-linked glycosylation 3 (alpha-1,3-mannosyltransferase)	*Alg3*	1,57557846	0,00027314
Protein phosphatase 1, regulatory (inhibitor) subunit 2	*Ppp1r2*	1,5753526	0,00010472
Family with sequence similarity 117, member b	*Fam117b*	1,57432354	4,47e-05
Myocyte enhancer factor 2d	*Mef2d*	1,57408113	0,00011744
Gram domain containing 1a	*Gramd1a*	1,57339935	0,00019657
Cytoplasmic polyadenylation element binding protein 3	*Cpeb3*	1,57251725	0,00032263
Vacuolar protein sorting 37a (yeast)	*Vps37a*	1,57244013	0,00043222
Ras homolog enriched in brain like 1	*Rhebl1*	1,57198503	0,0002669
Tetratricopeptide repeat domain 28	*Ttc28*	1,57155719	0,0019757
Protoporphyrinogen oxidase	*Ppox*	1,57127239	0,00852111
Acylpeptide hydrolase	*Apeh*	1,5710519	8,70e-05
Fatty acid desaturase 3	*Fads3*	1,57037026	6,29e-05
Axin interactor, dorsalization associated	*Aida*	1,57008196	0,00014372
Zinc finger ccch type containing 8	*Zc3h8*	1,56993888	0,00104308
Forty-two-three domain containing 1	*Fyttd1*	1,56965096	0,0004049
Signal recognition particle 9	*Srp9*	1,56903414	0,00012549
Unc-50 homolog (c. Elegans)	*Unc50*	1,56787187	3,28e-05
Aminolevulinic acid synthase 1	*Alas1*	1,56688158	8,20e-05

Early b cell factor 1	*Ebf1*	1,5666488	0,00039417
Ga repeat binding protein, alpha	*Gabpa*	1,56646796	0,00244525
Adaptor-related protein complex 3, mu 2 subunit	*Ap3m2*	1,56628331	0,00743909
Heparin-binding egf-like growth factor	*Hbegf*	1,56600367	0,0001781
Dual-specificity tyrosine-(y)-phosphorylation regulated kinase 1a	*Dyrk1a*	1,56507484	0,00330167
Hyaluronoglucosaminidase 2	*Hyal2*	1,56497575	6,43e-05
Flt3 interacting zinc finger protein 1	*Fiz1*	1,56415683	5,04e-05
Atpase, h+ transporting, lysosomal accessory protein 2	*Atp6ap2*	1,5636925	0,00059382
Toll-like receptor 4	*Tlr4*	1,56360016	0,00458512
Component of oligomeric golgi complex 8 /// peptide deformylase (mitochondrial)	*Cog8 /// pdf*	1,56357638	8,58e-05
Histidine triad nucleotide binding protein 2	*Hint2*	1,56353725	8,23e-05
Bromodomain adjacent to zinc finger domain, 2b	*Baz2b*	1,56348541	0,00025181
Hydroxysteroid (17-beta) dehydrogenase 4	*Hsd17b4*	1,56339817	0,00043978
Solute carrier organic anion transporter family, member 3a1	*Slco3a1*	1,56261125	0,00027602
Solute carrier family 25 (mitochondrial carrier ornithine transporter), member 15	*Slc25a15*	1,56247613	0,00067972
Protein-kinase, interferon-inducible double stranded rna dependent inhibitor, repressor of (p58 repressor)	*Prkrir*	1,56202768	0,0008736
Zinc finger protein 503	*Zfp503*	1,56174082	0,00577634
Frataxin	*Fxn*	1,56113678	0,00020562
Thyroid hormone receptor interactor 4	*Trip4*	1,55980458	7,51e-05
Cytosolic thiouridylase subunit 1 homolog (s. Pombe)	*Ctu1*	1,55912745	0,00053018
Nuclear factor, erythroid derived 2,-like 1	*Nfe2l1*	1,55835858	0,00012962
Dnaj (hsp40) homolog, subfamily c, member 5	*Dnajc5*	1,55813972	0,00213033
Uba-like domain containing 1	*Ubald1*	1,55787088	0,00581513
Homer homolog 1 (drosophila)	*Homer1*	1,55767041	0,00424025
Thioredoxin domain containing 17	*Txndc17*	1,55743398	0,00361946
Ptk2 protein tyrosine kinase 2 beta	*Ptk2b*	1,55730616	0,00143644
Choline/ethanolaminephosphotransferase 1	*Cept1*	1,55691029	7,50e-05
Riken cdna 4931406c07 gene	*4931406c07rik*	1,55669904	8,22e-05
Oxidoreductase like domain containing 1	*Oxld1*	1,55645161	0,00144407
Transmembrane 9 superfamily protein member 4	*Tm9sf4*	1,55578142	0,00078039
Mitochondrial ribosomal protein l43	*Mrpl43*	1,55568067	0,00018953

Interleukin 6 signal transducer	*Il6st*	1,55506359	0,00289939
Carcinoembryonic antigen-related cell adhesion molecule 1	*Ceacam1*	1,5550346	0,00173721
Phosphorylase kinase, gamma 2 (testis)	*Phkg2*	1,55468627	0,00013698
Tumor suppressor candidate 2	*Tusc2*	1,55453949	9,21e-05
Activating signal cointegrator 1 complex subunit 2	*Ascc2*	1,55442468	0,0001869
Pseudouridylate synthase 7 homolog (s. Cerevisiae)	*Pus7*	1,55440504	0,00014709
Microfibrillar-associated protein 1a /// microfibrillar-associated protein 1b	*Mfap1a /// mfap1b*	1,55396023	4,76e-05
Wd repeat domain 46	*Wdr46*	1,55380629	0,00139061
Exosome component 9	*Exosc9*	1,55375418	5,09e-05
Ankyrin 3, epithelial	*Ank3*	1,55314444	0,00113725
Sjogren's syndrome/scleroderma autoantigen 1 homolog (human)	*Sssca1*	1,55299856	0,00905587
Cttnbp2 n-terminal like	*Cttnbp2nl*	1,55270745	0,00590892
Peroxisomal biogenesis factor 26	*Pex26*	1,55264991	0,00033075
Gamma-glutamylamine cyclotransferase	*Ggact*	1,55261605	0,00636678
Sema domain, transmembrane domain (tm), and cytoplasmic domain, (semaphorin) 6a	*Sema6a*	1,5525168	0,00614469
Sema domain, immunoglobulin domain (ig), transmembrane domain (tm) and short cytoplasmic domain, (semaphorin) 4a	*Sema4a*	1,55234632	0,00021034
Cytidine 5'-triphosphate synthase 2	*Ctps2*	1,55209339	6,00e-05
Poly (adp-ribose) polymerase family, member 4	*Parp4*	1,55175539	0,00146826
Ceroid-lipofuscinosis, neuronal 5	*Cln5*	1,55163897	0,00050719
Zinc finger and btb domain containing 22	*Zbtb22*	1,55158161	7,36e-05
Dnaj (hsp40) homolog, subfamily c, member 11	*Dnajc11*	1,55157151	6,09e-05
Imp4, u3 small nucleolar ribonucleoprotein, homolog (yeast)	*Imp4*	1,55124911	0,00151513
Family with sequence similarity 222, member b	*Fam222b*	1,55086899	0,00045797
Regulator of calcineurin 1	*Rcan1*	1,55055999	5,12e-05
Inhibitor of kappa light polypeptide enhancer in b cells, kinase complex-associated protein	*Ikbkap*	1,55007231	0,0001118
Coilin	*Coil*	1,5499295	0,00085296
Serine incorporator 3	*Serinc3*	1,54991749	0,00191971
Hexosaminidase b	*Hexb*	1,5498362	0,00127865
Tnf receptor-associated factor 6	*Traf6*	1,54924647	0,00017142
Actin, alpha 1, skeletal muscle	*Acta1*	1,54905677	0,00042157

Tumor protein d52-like 2	*Tpd52l2*	1,54884053	0,00010356
Insulin-like 6	*Insl6*	1,54869908	0,0030599
Riken cdna 0610010b08 gene /// predicted gene 14308 /// predicted gene 14326 /// predicted gene 14391 /// predicted gene 14430 /// predicted gene 14434 /// predicted gene 4724 /// uncharacterized loc102639426	*0610010b08rik /// gm14308 /// gm14326 /// gm14391 /// gm14430 /// gm14434 /// gm4724 /// loc102639426*	1,54824258	0,00945271
Hepatitis a virus cellular receptor 2	*Havcr2*	1,54822708	0,00246164
Camp responsive element binding protein 3	*Creb3*	1,54776171	0,00047482
Nuclear import 7 homolog (s. Cerevisiae)	*Nip7*	1,54771828	0,00054193
Family with sequence similarity 58, member b	*Fam58b*	1,5474881	0,00035855
Lysophospholipase-like 1	*Lyplal1*	1,54655151	0,00040426
Zeta-chain (tcr) associated protein kinase	*Zap70*	1,54623321	0,00027923
Sprouty homolog 1 (drosophila)	*Spry1*	1,54445985	0,00022021
Transmembrane protein 185b	*Tmem185b*	1,54435392	0,0007579
Sperm antigen with calponin homology and coiled-coil domains 1	*Specc1*	1,54399771	0,00013726
Phospholipase c, delta 1	*Plcd1*	1,54389739	0,00035634
Ubiquitin specific peptidase 38	*Usp38*	1,5437893	0,00130086
Coiled-coil domain containing 22	*Ccdc22*	1,54362634	0,00100432
Laminin, gamma 2	*Lamc2*	1,54351774	4,62e-05
Leucine carboxyl methyltransferase 2	*Lcmt2*	1,54349834	0,00020231
60s ribosomal protein l23-like /// 60s ribosomal protein l23-like /// ribosomal protein l23	*Loc100044627 /// loc100862455 /// rpl23*	1,543147	3,87e-05
Aldo-keto reductase family 7, member a5 (aflatoxin aldehyde reductase)	*Akr7a5*	1,54301078	0,0002046
Solute carrier family 10 (sodium/bile acid cotransporter family), member 3	*Slc10a3*	1,54297152	3,78e-05
Polymerase (rna) ii (dna directed) polypeptide h	*Polr2h*	1,54283607	9,02e-05
Pax3 and pax7 binding protein 1	*Paxbp1*	1,54161637	0,00037301
Abhydrolase domain containing 10	*Abhd10*	1,54069081	0,00149289
Myosin xviiia	*Myo18a*	1,54055019	0,00163859
Thioredoxin domain containing 9	*Txndc9*	1,54015674	0,00033502
Ribonucleotide reductase m2 b (tp53 inducible)	*Rrm2b*	1,54012332	0,00065903
Ariadne ubiquitin-conjugating enzyme e2 binding	*Arih1*	1,53861868	0,00086467

protein homolog 1 (drosophila)			
Ribosomal protein l31 /// ribosomal protein l31, pseudogene 1 2	*Rpl31 /// rpl31-ps12*	1,5380016	0,00047397
Smg-8 homolog, nonsense mediated mrna decay factor (c. Elegans)	*Smg8*	1,53797463	0,0006113
Tea domain family member 4	*Tead4*	1,53727823	0,00038625
Sec14-like 2 (s. Cerevisiae)	*Sec14l2*	1,5366997	0,0013034
Zinc finger protein 160	*Zfp160*	1,53626342	0,0001192
Testis expressed gene 264	*Tex264*	1,53593795	0,00014329
Polymerase (rna) i polypeptide d	*Polr1d*	1,53547394	0,00016413
Solute carrier family 6 (neurotransmitter transporter), member 17	*Slc6a17*	1,53537663	0,00146111
Small arfgap 1	*Smap1*	1,53528431	0,00136682
Family with sequence similarity 96, member a	*Fam96a*	1,53493962	0,00350161
N-myc (and stat) interactor	*Nmi*	1,53480121	0,0001587
Cytochrome c oxidase assembly protein 17	*Cox17*	1,53471204	0,00031276
Listerin e3 ubiquitin protein ligase 1	*Ltn1*	1,53456838	0,0006646
Cd47 antigen (rh-related antigen, integrin-associated signal transducer)	*Cd47*	1,53440465	7,36e-05
Hydroxysteroid (17-beta) dehydrogenase 2	*Hsd17b2*	1,5341482	8,91e-05
Synaptopodin 2	*Synpo2*	1,53405496	6,28e-05
Rab34, member ras oncogene family	*Rab34*	1,53405393	4,46e-05
Mitochondrial ribosomal protein s9	*Mrps9*	1,53363679	0,00029104
N-acetyltransferase 9 (gcn5-related, putative)	*Nat9*	1,53359412	0,00030604
Protein kinase domain containing, cytoplasmic	*Pkdcc*	1,53332187	0,00539783
Yae1 domain containing 1	*Yae1d1*	1,53271329	0,0024532
Ring finger protein 185	*Rnf185*	1,53178693	4,95e-05
Angiopoietin 1	*Angpt1*	1,53156621	0,00466856
S100 calcium binding protein a13	*S100a13*	1,53133705	0,0001516
Flavin containing monooxygenase 5	*Fmo5*	1,5309132	0,00070349
A disintegrin and metallopeptidase domain 15 (metargidin)	*Adam15*	1,53079177	0,00028242
Twinfilin, actin-binding protein, homolog 2 (drosophila)	*Twf2*	1,53075174	0,00214566
Catenin beta interacting protein 1	*Ctnnbip1*	1,53063076	0,00057168
Zinc finger protein 275	*Zfp275*	1,5303659	0,00011857
Hephaestin	*Heph*	1,53035592	0,00282073
Inhibitor of bruton agammaglobulinemia tyrosine	*Ibtk*	1,53022292	0,00014212

kinase			
6-pyruvoyl-tetrahydropterin synthase	*Pts*	1,53017307	0,00090366
Autophagy related 12	*Atg12*	1,52963298	0,00020773
Small integral membrane protein 7	*Smim7*	1,5282908	0,00103471
Mitochondrial antiviral signaling protein	*Mavs*	1,52813997	0,00301709
Lps-induced tn factor	*Litaf*	1,52802906	0,00017428
Thioredoxin domain containing 11	*Txndc11*	1,5274415	0,00135376
Phd finger protein 23	*Phf23*	1,526988	0,00012159
Atpase family, aaa domain containing 1	*Atad1*	1,52681544	0,0041216
Excision repair cross-complementing rodent repair deficiency, complementation group 6 like 2	*Ercc6l2*	1,52651532	0,00392162
F-box protein 28	*Fbxo28*	1,52601218	0,00019226
Solute carrier family 25, member 44	*Slc25a44*	1,52587468	0,00029936
Leucine rich repeat containing 51	*Lrrc51*	1,52477877	0,00218402
Acid phosphatase 5, tartrate resistant	*Acp5*	1,52468593	0,00254197
Rab interacting factor	*Rabif*	1,52465423	0,00050463
Cathepsin d	*Ctsd*	1,52408503	3,50e-05
Diphthamine biosynthesis 6	*Dph6*	1,52405578	0,00011062
B cell leukemia/lymphoma 6	*Bcl6*	1,52389858	0,0056631
Tight junction protein 2	*Tjp2*	1,52265445	0,00035472
Nadh dehydrogenase (ubiquinone) 1 alpha subcomplex 10	*Ndufa10*	1,52258854	7,40e-05
Elongation protein 4 homolog (s. Cerevisiae)	*Elp4*	1,52253862	0,00087754
Synaptotagmin xi	*Syt11*	1,52167129	0,00802901
Period circadian clock 2	*Per2*	1,52092354	0,00083337
Mediator complex subunit 12	*Med12*	1,5198952	0,00462923
Cgg triplet repeat binding protein 1	*Cggbp1*	1,5193337	5,76e-05
Sideroflexin 4	*Sfxn4*	1,51892509	0,0009309
Clp1, cleavage and polyadenylation factor i subunit	*Clp1*	1,51833086	0,00029096
Small proline-rich protein 2d	*Sprr2d*	1,51819843	0,00196454
Mob family member 4, phocein	*Mob4*	1,51772094	0,00088338
Dual specificity phosphatase 10	*Dusp10*	1,51759686	0,00022812
Gram domain containing 3	*Gramd3*	1,51727122	0,0014965
Protein phosphatase 2, regulatory subunit b', gamma	*Ppp2r5c*	1,51722288	0,00030688
Myeloid/lymphoid or mixed-lineage leukemia (trithorax homolog, drosophila); translocated to, 10	*Mllt10*	1,51715188	0,00055804
Solute carrier family 37 (glycerol-3-phosphate transporter), member 2	*Slc37a2*	1,5159484	0,00269469

Interferon activated gene 205 /// myeloid cell nuclear differentiation antigen	*Ifi205 /// mnda*	1,51563533	0,00015992
Sirtuin 4	*Sirt4*	1,51434	0,00144734
Kat8 regulatory nsl complex subunit 1-like	*Kansl1l*	1,51409521	0,00050857
Riken cdna b230118h07 gene	*B230118h07rik*	1,51381354	0,00019969
Gem (nuclear organelle) associated protein 8	*Gemin8*	1,51356793	0,00151963
Leucine rich repeat containing 8a	*Lrrc8a*	1,51235354	0,00018976
Ubiquitin specific peptidase 3	*Usp3*	1,51106199	0,00031735
Protein arginine n-methyltransferase 7	*Prmt7*	1,51092281	0,00102691
Anoctamin 10	*Ano10*	1,51076161	0,00015905
Transmembrane protein 9	*Tmem9*	1,51074128	0,00082102
Ubiquitin-conjugating enzyme e2b	*Ube2b*	1,51027994	6,69e-05
Polymerase (rna) iii (dna directed) polypeptide c	*Polr3c*	1,50967538	0,00039022
Amylase 1, salivary	*Amy1*	1,50925042	0,00368662
Nop56 ribonucleoprotein	*Nop56*	1,50920384	0,00178373
Thap domain containing 4	*Thap4*	1,50918017	0,00017406
Hydroxysteroid (17-beta) dehydrogenase 12	*Hsd17b12*	1,50885753	0,00159766
Family with sequence similarity 160, member a2	*Fam160a2*	1,5086878	0,00517831
Dnaj (hsp40) homolog, subfamily c, member 1	*Dnajc1*	1,50867323	0,00197649
Zinc finger protein 354a	*Zfp354a*	1,50829753	0,00451104
Vma21 vacuolar h+-atpase homolog (s. Cerevisiae)	*Vma21*	1,50821642	0,00703088
Brain abundant, membrane attached signal protein 1	*Basp1*	1,50808584	4,73e-05
Heme oxygenase (decycling) 1	*Hmox1*	1,50786228	0,00018601
Tyrosyl-dna phosphodiesterase 1	*Tdp1*	1,50767415	0,00043214
Growth arrest and dna-damage-inducible 45 alpha	*Gadd45a*	1,50761023	0,00055804
Rfad1, flavin adenine dinucleotide synthetase, homolog (yeast) /// lens epithelial protein	*Flad1 /// lenep*	1,50747882	0,0022412
Glycogen synthase kinase 3 beta	*Gsk3b*	1,50565372	0,00243789
Involucrin	*Ivl*	1,50496401	0,00058664
Rna (guanine-7-) methyltransferase	*Rnmt*	1,5047874	0,00120367
Ubiquitin-conjugating enzyme e2r 2	*Ube2r2*	1,50470308	0,0008432
Rab33b, member ras oncogene family	*Rab33b*	1,50453531	0,00129999
Fibrous sheath-interacting protein 1	*Fsip1*	1,50435087	0,00174671
Predicted gene 3650 /// spastic paraplegia 11	*Gm3650 /// spg11*	1,50408991	0,0001677
Zinc finger protein 451	*Zfp451*	1,50406715	0,00068684
Mitochondria localized glutamic acid rich protein	*Mgarp*	1,50388358	0,00230525
Rab gtpase activating protein 1-like	*Rabgap1l*	1,50355053	0,00043674
Signal transducer and activator of transcription 1	*Stat1*	1,5035331	0,00705267

Crystallin, zeta (quinone reductase)-like 1	*Cryzl1*	1,50333808	0,00011412
Mitofusin 2	*Mfn2*	1,50254442	0,00026795
Kelch domain containing 3	*Klhdc3*	1,50223617	0,00203213
Surfeit gene 1	*Surf1*	1,50185861	0,00125748
Transmembrane protein 219	*Tmem219*	1,50166037	0,00253175
Phosphatidylinositol glycan anchor biosynthesis, class y-like	*Pigyl*	1,50155184	0,00040139
Filamin, beta	*Flnb*	1,50154266	0,00090981
Regulator of microtubule dynamics 3	*Rmdn3*	1,49957297	0,00123057
Speckle-type poz protein	*Spop*	1,49821068	0,00042042
Phosphatidylinositol 3 kinase, regulatory subunit, polypeptide 4, p150	*Pik3r4*	1,49820798	0,0015981
Ubiquitin specific peptidase 20	*Usp20*	1,49797417	0,00278018
Developmental pluripotency associated 2	*Dppa2*	1,49783961	0,00279318
Sorting nexin 1	*Snx1*	1,49780667	9,10e-05
Nfkb inhibitor interacting ras-like protein 1	*Nkiras1*	1,49708983	0,0011378
Acetyl-coenzyme a acyltransferase 1a /// acetyl-coenzyme a acyltransferase 1b	*Acaa1a /// acaa1b*	1,49628798	0,00140911
Wd repeat and fyve domain containing 1	*Wdfy1*	1,49612829	0,00051108
Prion protein	*Prnp*	1,49606978	0,0001168
Protein phosphatase 6, catalytic subunit	*Ppp6c*	1,49574682	0,00404118
Glucosamine-6-phosphate deaminase 2	*Gnpda2*	1,49564154	0,00030071
Sodium channel, voltage-gated, type v, alpha	*Scn5a*	1,49555156	0,00103659
Adenosine monophosphate deaminase 3	*Ampd3*	1,49540704	0,00030492
Nuclear transcription factor, x-box binding 1	*Nfx1*	1,4951037	0,00377901
Transmembrane protein 173	*Tmem173*	1,49468758	0,00317001
Zinc finger protein 605	*Zfp605*	1,49461044	0,00404319
Leprecan 1	*Lepre1*	1,49421104	8,49e-05
Histocompatibility 2, m region locus 9	*H2-m9*	1,49389536	0,00042063
Zinc finger protein 932	*Zfp932*	1,49369617	0,00041346
Magnesium-dependent phosphatase 1	*Mdp1*	1,49361048	0,00048071
Diacylglycerol kinase zeta	*Dgkz*	1,49338923	0,00178926
Autophagy related 13	*Atg13*	1,49310841	0,00159179
Nuclear receptor interacting protein 1	*Nrip1*	1,4930513	0,00014319
Notchless homolog 1 (drosophila)	*Nle1*	1,49286454	0,00463611
Zinc finger, mym-type 5	*Zmym5*	1,49274891	0,00025171
Riken cdna 2410016o06 gene	*2410016o06rik*	1,49216662	7,64e-05
B9 protein domain 1	*B9d1*	1,49214361	0,00068275

Nadh dehydrogenase (ubiquinone) 1 beta subcomplex 3	*Ndufb3*	1,49208688	0,00021293
Microtubule-associated protein 1 light chain 3 beta	*Map1lc3b*	1,49132483	0,00030297
Oxidase assembly 1-like	*Oxa1l*	1,49109326	0,00157098
Hypoxanthine guanine phosphoribosyl transferase	*Hprt*	1,49056398	0,0004345
Caspase 4, apoptosis-related cysteine peptidase	*Casp4*	1,49015312	0,00035798
Xeroderma pigmentosum, complementation group a	*Xpa*	1,48905546	0,00515466
Uridine monophosphate synthetase	*Umps*	1,48873308	0,00017064
Dynamin 1-like	*Dnm1l*	1,48849828	0,00012314
Seven in absentia 1a	*Siah1a*	1,48842233	0,00056426
Rhomboid, veinlet-like 3 (drosophila)	*Rhbdl3*	1,48779536	0,00194837
Solute carrier family 39 (metal ion transporter), member 11	*Slc39a11*	1,48721444	0,00018563
Glycolipid transfer protein domain containing 1	*Gltpd1*	1,48720144	0,00042556
Selenophosphate synthetase 2	*Sephs2*	1,48675186	0,00011765
Mus musculus 8 days embryo whole body cdna, riken full-length enriched library, clone:5730407p16 product:unclassifiable, full insert sequence.	*Ak077428*	1,48673407	0,00097806
Bcl2-like 11 (apoptosis facilitator)	*Bcl2l11*	1,48608499	0,00022539
Rho guanine nucleotide exchange factor (gef) 3	*Arhgef3*	1,48555402	0,00171562
Macrophage scavenger receptor 1	*Msr1*	1,48550181	0,00368974
Cystathionine beta-synthase	*Cbs*	1,48542217	0,00093994
Eukaryotic translation initiation factor 3, subunit a	*Eif3a*	1,48536549	0,00590597
Suppressor of ikbke 1	*Sike1*	1,48522647	0,00048315
Branched chain ketoacid dehydrogenase e1, alpha polypeptide	*Bckdha*	1,48503426	0,00151449
Expressed sequence c78339	*C78339*	1,48503315	6,22e-05
Hermansky-pudlak syndrome 4 homolog (human)	*Hps4*	1,48471623	0,00064255
Glycerophosphodiester phosphodiesterase 1	*Gde1*	1,48401059	7,34e-05
Ligand dependent nuclear receptor corepressor-like	*Lcorl*	1,48381712	0,00149755
Muscleblind-like 1 (drosophila)	*Mbnl1*	1,48379254	5,84e-05
Retinitis pigmentosa 9 (human)	*Rp9*	1,48361683	0,00019889
Predicted pseudogene 8069 /// translocase of outer mitochondrial membrane 5 homolog (yeast)	*Gm8069 /// tomm5*	1,48354705	0,00014138
Tubulin-specific chaperone c	*Tbcc*	1,48199265	0,00017918
Coiled-coil domain containing 84	*Ccdc84*	1,48185849	0,0031789
Interferon induced transmembrane protein 6	*Ifitm6*	1,48149545	0,00153057
Solute carrier family 39 (metal ion transporter),	*Slc39a13*	1,48138412	0,00071181

member 13			
Mucolipin 1	*Mcoln1*	1,48122812	0,00021706
Acyl-coenzyme a dehydrogenase family, member 8	*Acad8*	1,48096417	0,00147084
Ring finger protein 141	*Rnf141*	1,48069075	0,00142643
Caax box 1a /// caax box 1b	*Cxx1a /// cxx1b*	1,47965779	0,00657537
Cyclin d-type binding-protein 1	*Ccndbp1*	1,47906656	0,00037068
Growth arrest and dna-damage-inducible, gamma interacting protein 1	*Gadd45gip1*	1,4789371	0,00112702
Polymerase (rna) iii (dna directed) polypeptide f	*Polr3f*	1,47872662	0,00746014
Suppressor of ty 5	*Supt5*	1,47868367	0,00023792
Rab3 gtpase activating protein subunit 2	*Rab3gap2*	1,47825995	0,00235134
Atpase, ca++ transporting, type 2c, member 2	*Atp2c2*	1,47785494	0,00917625
Adaptor protein complex ap-1, gamma 2 subunit	*Ap1g2*	1,47761675	0,00037417
Melanoma inhibitory activity 3	*Mia3*	1,47688971	0,00083941
Protein tyrosine phosphatase, non-receptor type 21	*Ptpn21*	1,47676448	0,00402692
Glis family zinc finger 2	*Glis2*	1,47669929	0,00014982
P21 protein (cdc42/rac)-activated kinase 2	*Pak2*	1,47612656	0,00503722
Breast carcinoma amplified sequence 2	*Bcas2*	1,47560375	0,00073102
Coiled-coil-helix-coiled-coil-helix domain containing 6	*Chchd6*	1,47545941	0,00236693
Rho gtpase activating protein 29	*Arhgap29*	1,47543984	0,00515711
Rab, member ras oncogene family-like 3	*Rabl3*	1,4752037	0,00082538
Ubiquinol cytochrome c reductase core protein 2	*Uqcrc2*	1,47493837	0,00020335
Udp-gal:betagal beta 1,3-galactosyltransferase, polypeptide 6	*B3galt6*	1,47483072	0,00204334
Kinesin family member 3a	*Kif3a*	1,47474676	0,00026186
Mucolipin 3	*Mcoln3*	1,47448618	0,00297093
Gtp binding protein 4	*Gtpbp4*	1,47435224	0,00070305
Mitochondrial amidoxime reducing component 2	*Iii.02*	1,47430975	6,22e-05
Fumarylacetoacetate hydrolase domain containing 1	*Fahd1*	1,47414654	0,00044372
Stt3, subunit of the oligosaccharyltransferase complex, homolog b (s. Cerevisiae)	*Stt3b*	1,47413443	0,00011034
Nitrilase family, member 2	*Nit2*	1,47357375	0,00042702
Riken cdna 3830406c13 gene	*3830406c13rik*	1,47320234	0,00019419
Tubulin, alpha 8	*Tuba8*	1,47308583	0,00164292
Predicted gene 12942 /// zinc finger, mym-type 6	*Gm12942 /// zmym6*	1,47307356	0,00099971
Surfeit gene 6	*Surf6*	1,47283601	0,00065445
Er membrane protein complex subunit 9	*Emc9*	1,47239602	0,00068735

Origin recognition complex, subunit 4	*Orc4*	1,47217369	0,00705718
Zinc finger and btb domain containing 3	*Zbtb3*	1,47206187	0,00232066
Zinc finger, zz domain containing 3	*Zzz3*	1,47203055	0,00199665
Exosome component 5	*Exosc5*	1,47167506	0,00057618
Tuberous sclerosis 1	*Tsc1*	1,47112859	0,00012226
Phosphatidylinositol glycan anchor biosynthesis, class t	*Pigt*	1,47095916	0,00304291
Sphingomyelin phosphodiesterase, acid-like 3a	*Smpdl3a*	1,47050842	0,00067708
Rev1 homolog (s. Cerevisiae)	*Rev1*	1,47035224	0,00253485
Mitotic spindle organizing protein 2	*Mzt2*	1,46947407	0,00031784
Zinc finger protein 120	*Zfp120*	1,4694362	0,00712289
Lactamase, beta 2	*Lactb2*	1,46931251	0,00020242
Transmembrane protein 147	*Tmem147*	1,46913522	0,00021646
Zinc finger protein 94	*Zfp94*	1,46900327	0,00477749
Clustered mitochondria (clua/clu1) homolog	*Cluh*	1,46888305	0,00022516
F-box protein 6	*Fbxo6*	1,46878093	0,00016604
Ubiquitin protein ligase e3 component n-recognin 3	*Ubr3*	1,46872992	0,00831731
Ceramide synthase 2	*Cers2*	1,46833838	7,92e-05
Protein tyrosine phosphatase, receptor type, n	*Ptprn*	1,46823992	0,0047043
Dehydrogenase/reductase (sdr family) member 11	*Dhrs11*	1,46801956	0,00548837
Wd repeat domain 81	*Wdr81*	1,46762517	0,00204672
Expressed sequence aa408650	*Aa408650*	1,46757679	0,00058706
Peroxiredoxin 3	*Prdx3*	1,46728387	0,0002812
Dnaj (hsp40) homolog, subfamily a, member 1	*Dnaja1*	1,46690011	0,00260485
Tripeptidyl peptidase ii	*Tpp2*	1,46643798	0,0016196
Aldo-keto reductase family 1, member b3 (aldose reductase) /// akr1b3 pseudogene	*Akr1b3 /// gm6644*	1,46641127	0,00014092
Zinc finger protein 131	*Zfp131*	1,4659758	0,00131054
Nadh dehydrogenase (ubiquinone) 1 alpha subcomplex, assembly factor 3	*Ndufaf3*	1,46560109	0,00040356
Regulatory factor x, 5 (influences hla class ii expression)	*Rfx5*	1,46520024	0,0003624
Golgi apparatus protein 1	*Glg1*	1,4651609	0,00112044
Syntaxin 8	*Stx8*	1,46436742	0,00041339
Transcription factor b1, mitochondrial	*Tfb1m*	1,46392954	0,00746919
Transmembrane protein 189	*Tmem189*	1,46392866	0,00025533
Vasodilator-stimulated phosphoprotein	*Vasp*	1,46385754	0,00071427
Immediate early response 5	*Ier5*	1,46363097	0,00780667

Myb binding protein (p160) 1a	*Mybbp1a*	1,46250522	0,00187457
Bcl2/adenovirus e1b interacting protein 3	*Bnip3*	1,46184574	0,00028887
Cdk2-associated protein 2	*Cdk2ap2*	1,46182746	0,0003064
G protein-coupled receptor kinase 4	*Grk4*	1,46175848	0,00237486
Splicing regulatory glutamine/lysine-rich protein 1interacting protein 1	*Srek1ip1*	1,46159494	0,00117866
Deah (asp-glu-ala-his) box polypeptide 8	*Dhx8*	1,46136919	0,00021947
Adipogenesis associated mth938 domain containing	*Aamdc*	1,46096083	0,00031474
Aarf domain containing kinase 3	*Adck3*	1,4607637	0,00831481
Potassium voltage-gated channel, shal-related family, member 2	*Kcnd2*	1,4606275	0,00052169
Cell death inducing trp53 target 1	*Cdip1*	1,46042033	0,00091723
Leucine zipper transcription factor-like 1	*Lztfl1*	1,46022661	0,00255489
Nop14 nucleolar protein	*Nop14*	1,45908417	0,00062058
Myc induced nuclear antigen	*Mina*	1,45904638	0,0026253
Neurolysin (metallopeptidase m3 family)	*Nln*	1,45832409	0,00046182
Synaptotagmin binding, cytoplasmic rna interacting protein	*Syncrip*	1,45802507	0,00385776
Interleukin 7	*Il7*	1,45771183	0,00470153
Upstream binding protein 1	*Ubp1*	1,45725535	0,00089508
Ubiquitin-like 3	*Ubl3*	1,45722177	0,00012082
Ataxia telangiectasia and rad3 related	*Atr*	1,45716319	0,00029173
Family with sequence similarity 69, member b	*Fam69b*	1,45683088	0,00061076
Niemann-pick type c2	*Npc2*	1,45681308	0,0008714
Centrin 2	*Cetn2*	1,45667734	0,0004848
Glutamate-ammonia ligase (glutamine synthetase)	*Glul*	1,45656929	0,0039432
Reticulon 4 interacting protein 1	*Rtn4ip1*	1,45630981	0,00496079
Cellular retinoic acid binding protein ii	*Crabp2*	1,45594334	0,00010987
Cytochrome c oxidase subunit vb	*Cox5b*	1,45526143	0,00033754
Ribonuclease l (2', 5'-oligoisoadenylate synthetase-dependent)	*Rnasel*	1,45474782	0,00603405
Arginyl aminopeptidase (aminopeptidase b)-like 1	*Rnpepl1*	1,45458294	0,00056935
Protein kinase inhibitor, gamma	*Pkig*	1,45393043	0,00279606
Abhydrolase domain containing 17c	*Abhd17c*	1,45371074	0,00015341
Copper chaperone for superoxide dismutase	*Ccs*	1,45363372	0,00019306
Poly(a) binding protein, nuclear 1	*Pabpn1*	1,45166029	0,00963445
Udp-glcnac:betagal beta-1,3-n-acetylglucosaminyltransferase 9	*B3gnt9*	1,45139625	0,00429808

Helicase with zinc finger 2, transcriptional coactivator	*Helz2*	1,45107065	0,00646695
Tafazzin	*Taz*	1,45094688	0,00029303
Nitrilase 1	*Nit1*	1,4499046	0,00114474
Coiled-coil domain containing 93	*Ccdc93*	1,44988773	0,0003813
Advillin	*Avil*	1,44971498	0,00299983
Sorting nexin 5	*Snx5*	1,44970198	0,00021192
Syntaxin 3	*Stx3*	1,44914069	0,0031957
B cell leukemia/lymphoma 10	*Bcl10*	1,44894666	0,00032784
Protein kinase c substrate 80k-h	*Prkcsh*	1,44829913	0,00041622
Mitochondrial ribosomal protein s12	*Mrps12*	1,44807013	0,00015141
Kdel (lys-asp-glu-leu) containing 1	*Kdelc1*	1,44786378	0,00031143
Proline-rich polypeptide 3	*Prr3*	1,44775494	0,0048668
Nop58 ribonucleoprotein	*Nop58*	1,44755306	0,00061185
Multiple coagulation factor deficiency 2	*Mcfd2*	1,44724004	0,00129907
Methionyl aminopeptidase 1	*Metap1*	1,44675787	0,00358613
Septin 8	*Ix.08*	1,44635788	0,00053885
Tousled-like kinase 2 (arabidopsis)	*Tlk2*	1,44616369	0,00099756
Acyl-coenzyme a dehydrogenase family, member 9	*Acad9*	1,44616167	0,00622774
Expressed sequence ai597479	*Ai597479*	1,44598848	0,00071604
Hexosaminidase (glycosyl hydrolase family 20, catalytic domain) containing	*Hexdc*	1,44590496	0,0004183
Ubiquitin specific peptidase 8	*Usp8*	1,44576757	0,00053085
Adenylate cyclase 9	*Adcy9*	1,44526753	0,00564885
Trna methyltransferase 1 like	*Trmt1l*	1,44515793	0,00165666
Golgi autoantigen, golgin subfamily a, 4	*Golga4*	1,44439908	0,00495329
Immunoglobulin (cd79a) binding protein 1	*Igbp1*	1,44312345	0,00032912
Ninjurin 1	*Ninj1*	1,44310814	0,0010901
Adiponectin receptor 1	*Adipor1*	1,44283297	0,0020248
Atp-binding cassette, sub-family c (cftr/mrp), member 1	*Abcc1*	1,44279027	0,00045622
Erythrocyte protein band 4.1-like 4a	*Epb4.1l4a*	1,44271925	0,00071567
Glutamate-cysteine ligase, catalytic subunit	*Gclc*	1,44252159	0,00138529
Late endosomal/lysosomal adaptor, mapk and mtor activator 2	*Lamtor2*	1,44220395	0,00036264
Predicted gene 13152	*Gm13152*	1,44220221	0,00198154
Vesicle-associated membrane protein, associated protein b and c	*Vapb*	1,44124719	0,00093881

Ubiquitin specific peptidase 14	*Usp14*	1,44124185	0,0002518
Magnesium transporter 1	*Magt1*	1,44119722	0,0079557
Nucleoporin 62	*Nup62*	1,44102372	0,00023221
Metaxin 1	*Mtx1*	1,44091427	0,00178998
Dual specificity phosphatase 8	*Dusp8*	1,44091138	0,00111299
Proteasome (prosome, macropain) 26s subunit, non-atpase, 8	*Psmd8*	1,44087797	0,00154224
Fos-like antigen 2	*Fosl2*	1,44035769	0,00801247
Aspartyl-trna synthetase	*Dars*	1,43980843	0,00151174
Set nuclear oncogene	*Set*	1,43974981	0,00010356
Mitogen-activated protein kinase 9	*Mapk9*	1,4395704	0,00041946
Dnaj (hsp40) homolog, subfamily a, member 3	*Dnaja3*	1,43946856	0,00016843
Isocitrate dehydrogenase 3 (nad+) alpha	*Idh3a*	1,43899171	0,00233842
Ubiquitin-conjugating enzyme e2a	*Ube2a*	1,43893159	0,00375543
Mitochondrial ribosomal protein l40	*Mrpl40*	1,43891782	0,00052928
Riken cdna 0610010b08 gene /// predicted gene 14308 /// predicted gene 14325 /// predicted gene 14326 /// predicted gene 14430 /// predicted gene 14432 /// predicted gene 14434 /// predicted gene 4724 /// predicted gene 6710 /// zinc finger protein 91-like /// zinc finger protein 709-like	*0610010b08rik /// gm14308 /// gm14325 /// gm14326 /// gm14430 /// gm14432 /// gm14434 /// gm4724 /// gm6710 /// loc102639412 /// loc628147*	1,43861123	0,00082984
Tsc22 domain family, member 1	*Tsc22d1*	1,43799294	0,0001559
Platelet-activating factor acetylhydrolase, isoform 1b, subunit 2	*Pafah1b2*	1,43789438	0,00051163
Mesenteric estrogen dependent adipogenesis	*Medag*	1,43778962	0,00265689
Cdk2 associated, cullin domain 1	*Cacul1*	1,43774123	0,00191698
Dolichol kinase	*Dolk*	1,43736786	0,00012289
Ubiquitin specific peptidase 47	*Usp47*	1,43735559	0,00023733
Ankyrin repeat and socs box-containing 8	*Asb8*	1,43711383	0,00070341
Mitochondrial ribosomal protein s17	*Mrps17*	1,43708526	9,17e-05
Mitochondrial pyruvate carrier 2	*Mpc2*	1,43691862	0,00043395
Trans-golgi network protein /// trans-golgi network protein 2	*Tgoln1 /// tgoln2*	1,43686235	0,00015182
Solute carrier family 30 (zinc transporter), member 1	*Slc30a1*	1,43685059	0,00050296

St3 beta-galactoside alpha-2,3-sialyltransferase 2	*St3gal2*	1,43654253	0,00451346
Neuroblastoma, suppression of tumorigenicity 1	*Nbl1*	1,43594025	0,00342007
Myosin vi	*Myo6*	1,4349729	0,0042027
Dead (asp-glu-ala-asp) box polypeptide 52	*Ddx52*	1,43495489	0,00059594
A kinase (prka) anchor protein 1	*Akap1*	1,43491116	0,00039599
Riken cdna 1810030o07 gene	*1810030o07rik*	1,43486966	0,00026705
Alpha thalassemia/mental retardation syndrome x-linked homolog (human)	*Atrx*	1,43455258	0,00959363
Pyruvate dehydrogenase kinase, isoenzyme 1	*Pdk1*	1,43414771	0,00099402
Protein s (alpha)	*Pros1*	1,43377886	0,00051865
Cub domain containing protein 1	*Cdcp1*	1,43350899	0,00053365
Spastic paraplegia 20, spartin (troyer syndrome) homolog (human)	*Spg20*	1,43237068	0,0007671
Major facilitator superfamily domain containing 5	*Mfsd5*	1,43218357	0,00015703
Phosphatidylinositol glycan anchor biosynthesis, class x	*Pigx*	1,43183009	0,00160843
Isocitrate dehydrogenase 3 (nad+), gamma	*Idh3g*	1,43181476	0,00012289
Solute carrier family 39 (zinc transporter), member 7	*Slc39a7*	1,43163781	0,00017663
Phosphodiesterase 12	*Pde12*	1,4315164	0,00388956
Eukaryotic translation termination factor 1	*Etf1*	1,43142289	0,00082966
Homocysteine-inducible, endoplasmic reticulum stress-inducible, ubiquitin-like domain member 1	*Herpud1*	1,43122444	0,00185486
Mitochondrial ribosomal protein l16	*Mrpl16*	1,43118449	0,0001712
Mitochondrial ribosomal protein s31	*Mrps31*	1,43101125	0,0005881
Glucose-fructose oxidoreductase domain containing 2	*Gfod2*	1,43061048	0,00015239
Bone morphogenetic protein receptor, type 1b	*Bmpr1b*	1,42989792	0,00755455
Williams beuren syndrome chromosome region 27 (human)	*Wbscr27*	1,42976895	0,00018799
Proteasome (prosome, macropain) subunit, beta type 8 (large multifunctional peptidase 7)	*Psmb8*	1,42965463	0,00401785
Peptidyl-trna hydrolase 1 homolog (s. Cerevisiae)	*Ptrh1*	1,42944556	0,00024452
Denn/madd domain containing 1a	*Dennd1a*	1,42914834	0,0006499
Ring finger protein 138	*Rnf138*	1,42909948	0,00548634
Er membrane protein complex subunit 4	*Emc4*	1,42872003	0,00336687
Ae binding protein 1	*Aebp1*	1,42870945	0,00218402
Acyl-coa thioesterase 13	*Acot13*	1,42838474	0,0019336
Expressed sequence au020206	*Au020206*	1,42837811	0,00047172

Mitochondrial ribosomal protein s30	*Mrps30*	1,42812699	0,00044466
Rna binding motif protein 15b	*Rbm15b*	1,42811595	0,00204348
Nima (never in mitosis gene a)-related expressed kinase 4	*Nek4*	1,42810152	0,00243301
Solute carrier family 5, member 4a	*Slc5a4a*	1,4279135	0,00127089
F-box and leucine-rich repeat protein 12	*Fbxl12*	1,42758038	0,00353199
Nadh dehydrogenase (ubiquinone) fe-s protein 3	*Ndufs3*	1,4271308	0,0007931
Endoplasmic reticulum-golgi intermediate compartment (ergic) 1	*Ergic1*	1,42664041	0,00021081
Er membrane protein complex subunit 6	*Emc6*	1,42642826	0,00043944
Ntf2-related export protein 1	*Nxt1*	1,42604352	0,00025182
Nudix (nucleoside diphosphate linked moiety x)-type motif 16-like 1	*Nudt16l1*	1,42585066	0,00964137
Heat shock 105kda/110kda protein 1	*Hsph1*	1,42582666	0,00126531
Asparagine-linked glycosylation 14	*Alg14*	1,42558497	0,00493237
Cyclin t2	*Ccnt2*	1,42515538	0,00574236
Calcium binding protein 39	*Cab39*	1,42495748	0,00018424
Ubiquinol-cytochrome c reductase core protein 1	*Uqcrc1*	1,42480507	0,00057898
Riken cdna 1600012h06 gene	*1600012h06rik*	1,42476867	0,00114882
V-crk sarcoma virus ct10 oncogene homolog (avian)-like	*Crkl*	1,42448218	0,00024056
Zinc finger ccch-type containing 15	*Zc3h15*	1,42377247	0,00146186
Dna segment, chr 19, brigham & women's genetics 1357 expressed	*D19bwg1357e*	1,42371166	0,00232006
Mitogen-activated protein kinase kinase 4	*Map2k4*	1,42365144	0,00079041
Transducin (beta)-like 1 x-linked	*Tbl1x*	1,42355507	0,00887273
Ash1 (absent, small, or homeotic)-like (drosophila)	*Ash1l*	1,42354501	0,00076303
Rogdi homolog (drosophila)	*Rogdi*	1,42344726	0,00106139
Translocase of inner mitochondrial membrane 10b	*Timm10b*	1,42334138	0,00085307
Ubiquitin specific peptidase 19	*Usp19*	1,42325711	0,00030614
Differentially expressed in b16f10 1	*Deb1*	1,42317249	0,00179834
Heat shock protein 3	*Hspb3*	1,42286499	0,00068461
Parkinson disease (autosomal recessive, early onset) 7	*Park7*	1,42275932	0,0008837
Coiled-coil domain containing 186	*Ccdc186*	1,42244777	0,00225756
Mid1 interacting protein 1 (gastrulation specific g12-like (zebrafish))	*Mid1ip1*	1,42171335	0,00766017
Hook homolog 2 (drosophila)	*Hook2*	1,42144059	0,00117245

Stromal interaction molecule 1	*Stim1*	1,42142963	0,00125127
Telo2 interacting protein 1	*Tti1*	1,42110845	0,00285222
Otu domain containing 7a	*Otud7a*	1,42076437	0,00197293
Septin 2	*Ix.02*	1,42063361	0,00380244
Mitchondrial ribosomal protein s7	*Mrps7*	1,42030095	0,00035551
Centrosomal protein 112	*Cep112*	1,42017318	0,00112429
V-crk sarcoma virus ct10 oncogene homolog (avian)	*Crk*	1,41976171	0,00318924
Adp-ribosylation factor related protein 1	*Arfrp1*	1,41946838	0,00184128
Biogenesis of lysosomal organelles complex-1, subunit 6, pallidin	*Bloc1s6*	1,41944471	0,00055891
Trna methyltransferase 10a	*Trmt10a*	1,41922326	0,00109716
Cytochrome p450, family 2, subfamily c, polypeptide 44	*Cyp2c44*	1,4191071	0,00111778
Maturin, neural progenitor differentiation regulator homolog (xenopus)	*Mturn*	1,41865214	0,00531082
K(lysine) acetyltransferase 2a	*Kat2a*	1,41839233	0,00089571
H2-k region expressed gene 6	*H2-ke6*	1,41836245	0,00223882
Mediator complex subunit 9	*Med9*	1,41833288	0,00125243
Coiled-coil domain containing 22 /// protein phosphatase 1, regulatory (inhibitor) subunit 3f	*Ccdc22 /// ppp1r3f*	1,41800689	0,00060179
Nitrogen fixation gene 1 (s. Cerevisiae)	*Nfs1*	1,4179614	0,00038282
Expressed sequence au040320	*Au040320*	1,41674886	0,00387651
Cyclin k	*Ccnk*	1,4164429	0,00564071
Tsr3 20s rrna accumulation	*Tsr3*	1,41622829	0,00169939
Anthrax toxin receptor 1	*Antxr1*	1,41608438	0,00280739
Myosin, light polypeptide 1	*Myl1*	1,41589458	0,00689224
Inhibitor of cdk, cyclin a1 interacting protein 1	*Inca1*	1,41576883	0,00364846
Microtubule-associated protein 1 light chain 3 alpha	*Map1lc3a*	1,41562436	0,00021188
Cytochrome c oxidase assembly factor 6	*Coa6*	1,41520507	0,00125855
Autophagy related 14	*Atg14*	1,41511907	0,00965095
Golgi snap receptor complex member 1	*Gosr1*	1,41503836	0,00017575
Ddrgk domain containing 1	*Ddrgk1*	1,41498338	0,00048979
Phenylalanyl-trna synthetase, beta subunit	*Farsb*	1,41381535	0,00133921
General transcription factor iif, polypeptide 2	*Gtf2f2*	1,41344448	0,0039072
Poly(a) binding protein interacting protein 2b	*Paip2b*	1,41342911	0,00022947
Yth domain containing 1	*Ythdc1*	1,41327843	0,00050446
Transforming, acidic coiled-coil containing protein 2	*Tacc2*	1,41284331	0,00056989
Prolactin family 7, subfamily a, member 2	*Prl7a2*	1,41204786	0,00136492

Thiamine triphosphatase	*Thtpa*	1,41203627	0,00031185
A disintegrin and metallopeptidase domain 17	*Adam17*	1,41178328	0,00324803
Zinc finger protein 644	*Zfp644*	1,41176238	0,00711704
Zinc finger rna binding protein	*Zfr*	1,41145647	0,00718133
Hydroxyacyl-coenzyme a dehydrogenase	*Hadh*	1,41144279	0,0003235
Taf5-like rna polymerase ii, p300/cbp-associated factor (pcaf)-associated factor	*Taf5l*	1,41141578	0,00062964
Smad family member 1	*Smad1*	1,41120226	0,00215395
Family with sequence similarity 126, member b	*Fam126b*	1,41106429	0,00690286
Origin recognition complex, subunit 2	*Orc2*	1,4107686	0,00016529
Carbonyl reductase 1	*Cbr1*	1,41060719	0,00372023
Mitogen-activated protein kinase kinase 1	*Map2k1*	1,41057425	0,00043985
Dead (asp-glu-ala-asp) box polypeptide 5	*Ddx5*	1,41030915	0,00636647
N-acetylneuraminic acid phosphatase	*Nanp*	1,40952918	0,00121043
Cysteinyl-trna synthetase	*Cars*	1,40872023	0,00070224
Pantothenate kinase 1	*Pank1*	1,40862765	0,00506402
Nucleolar protein interacting with the fha domain of mki67	*Nifk*	1,40856188	0,00118127
Spindlin 1	*Spin1*	1,4085218	0,00016008
Arfgap with fg repeats 1	*Agfg1*	1,40848444	0,00071024
Small integral membrane protein 12	*Smim12*	1,40831135	0,00055043
Lysine (k)-specific demethylase 5b	*Kdm5b*	1,4081519	0,00323844
Serine carboxypeptidase 1	*Scpep1*	1,40809196	0,00560058
Mau2 chromatid cohesion factor homolog (c. Elegans)	*Mau2*	1,40773062	0,00256811
Scavenger receptor class b, member 2	*Scarb2*	1,40739651	0,00042425
Predicted gene 10015 /// predicted gene 10145 /// predicted gene 10705 /// predicted gene 5858 /// ubiquitin-conjugating enzyme e2l 3	*Gm10015 /// gm10145 /// gm10705 /// gm5858 /// ube2l3*	1,4067822	0,00127089
Adenylosuccinate synthetase, non muscle	*Adss*	1,40585982	0,00176696
Dual specificity phosphatase 12	*Dusp12*	1,40560288	0,00049485
Chromodomain helicase dna binding protein 6	*Chd6*	1,40543631	0,00278018
Acidic (leucine-rich) nuclear phosphoprotein 32 family, member a	*Anp32a*	1,40524449	0,00124546
F-box protein 36	*Fbxo36*	1,40499477	0,00032811
Dual specificity phosphatase 7	*Dusp7*	1,40438803	0,00443919
Cyclin-dependent kinase 2 interacting protein	*Cinp*	1,40437553	0,00017135

Sub1 homolog (s. Cerevisiae)	*Sub1*	1,40413531	0,00485167
Transcriptional adaptor 2b	*Tada2b*	1,40390542	0,00204484
Mediator complex subunit 8	*Med8*	1,4038172	0,00065939
Mitogen-activated protein kinase binding protein 1	*Mapkbp1*	1,4033377	0,00059321
Inversin	*Invs*	1,40315639	0,00807889
Brain protein i3	*Bri3*	1,40259394	0,00179075
Intercellular adhesion molecule 4, landsteiner-wiener blood group	*Icam4*	1,40244214	0,00199321
Exosome component 7	*Exosc7*	1,40226046	0,00124507
Follistatin-like 1	*Fstl1*	1,40217531	0,00025914
Cytochrome c oxidase assembly protein 18	*Cox18*	1,40158317	0,00165304
Interferon (alpha and beta) receptor 1	*Ifnar1*	1,40114188	0,00148473
Carbonyl reductase 2	*Cbr2*	1,40112621	0,00232323
Zinc finger protein 40	*Zfp40*	1,40090747	0,00869205
Poly (adp-ribose) polymerase family, member 6	*Parp6*	1,40075368	0,00292467
Amyotrophic lateral sclerosis 2 (juvenile)	*Als2*	1,4007504	0,00137532
Acidic (leucine-rich) nuclear phosphoprotein 32 family, member b	*Anp32b*	1,400736	0,00014138
Atpase, h+ transporting, lysosomal v1 subunit h	*Atp6v1h*	1,40045457	0,00117138
Udp-galnac:betaglcnac beta 1,3-galactosaminyltransferase, polypeptide 2	*B3galnt2*	1,40042833	0,00816213
Predicted gene 4604 /// ribosomal protein l36	*Gm4604 /// rpl36*	1,40014529	0,00228473
Insulin degrading enzyme	*Ide*	1,40000274	0,00068855
Sh3-domain grb2-like endophilin b2	*Sh3glb2*	1,39986929	0,00044367
Autophagy/beclin 1 regulator 1	*Ambra1*	1,3997454	0,0012534
Rap1 gtpase-activating protein	*Rap1gap*	1,39929992	0,00153433
Fc receptor, igg, low affinity iv	*Fcgr4*	1,39845767	0,00086384
Pest proteolytic signal containing nuclear protein	*Pcnp*	1,39832306	0,00025288
Predicted pseudogene 3244 /// predicted gene 3873 /// nadh dehydrogenase (ubiquinone) 1 beta subcomplex 4	*Gm3244 /// gm3873 /// ndufb4*	1,39820744	0,00532929
Riken cdna d030056l22 gene	*D030056l22rik*	1,39808413	0,00072287
Er membrane protein complex subunit 2	*Emc2*	1,39803259	0,00031162
Sorting nexin 10	*Snx10*	1,39801796	0,00064675
Amme chromosomal region gene 1-like	*Ammecr1l*	1,39725366	0,00079435
Vinculin	*Vcl*	1,39719936	0,00128582
Solute carrier family 39 (metal ion transporter), member 6	*Slc39a6*	1,39644208	0,00085707

Solute carrier family 12, member 2	*Slc12a2*	1,39628896	0,00061003
Tripartite motif-containing 8	*Trim8*	1,39625622	0,0031059
Kinectin 1	*Ktn1*	1,39566203	0,00154503
Replication termination factor 2 domain containing 1	*Rtfdc1*	1,3953981	0,00379062
Atpase, class vi, type 11b	*Atp11b*	1,39455326	0,00048065
General transcription factor ii h, polypeptide 1	*Gtf2h1*	1,39420383	0,00070305
Hcls1 associated x-1	*Hax1*	1,39341707	0,00053529
Cdna sequence bc003965	*Bc003965*	1,39311345	0,00065884
Sec22 vesicle trafficking protein homolog b (s. Cerevisiae)	*Sec22b*	1,39308174	0,00057573
Predicted gene 5633 /// predicted gene 9178 /// mrt4, mrna turnover 4, homolog (s. Cerevisiae)	*Gm5633 /// gm9178 /// mrto4*	1,39301153	0,00204262
Spectrin alpha, non-erythrocytic 1	*Sptan1*	1,39299598	0,00262457
Prefoldin subunit 6	*Pfdn6*	1,39297725	0,0019559
Phosphatidylinositol glycan anchor biosynthesis, class a	*Piga*	1,39271783	0,00932937
Tm2 domain containing 3	*Tm2d3*	1,39222738	0,00361726
Tectonic family member 3	*Tctn3*	1,39190426	0,00176371
Nadh dehydrogenase (ubiquinone) 1 alpha subcomplex, 1	*Ndufa1*	1,39177482	0,00058675
Eukaryotic translation initiation factor 3, subunit g	*Eif3g*	1,39152629	0,0005295
Tectonin beta-propeller repeat containing 1	*Tecpr1*	1,39132281	0,00538024
Dna segment, chr 2, wayne state university 81, expressed	*D2wsu81e*	1,39128153	0,00027647
Purine rich element binding protein a	*Pura*	1,3907496	0,00289999
Zinc finger protein 282	*Zfp282*	1,39063774	0,00539328
Poly (adp-ribose) polymerase family, member 8	*Parp8*	1,39042241	0,00158651
Presequence translocase-asssociated motor 16 homolog (s. Cerevisiae)	*Pam16*	1,39035627	0,0010426
Sec22 vesicle trafficking protein homolog a (s. Cerevisiae)	*Sec22a*	1,3903536	0,00609055
Protein tyrosine phosphatase, non-receptor type 6	*Ptpn6*	1,3902227	0,00195128
Protein disulfide isomerase associated 4	*Pdia4*	1,38971098	0,00194091
Interleukin enhancer binding factor 3	*Ilf3*	1,38961331	0,00514263
Phosphatidylserine synthase 2	*Ptdss2*	1,38957187	0,00071401
Bmp2 inducible kinase	*Bmp2k*	1,38954955	0,00274214
Ubiquitin fusion degradation 1 like	*Ufd1l*	1,38950135	0,00131254
Dph1 homolog (s. Cerevisiae) /// candidate tumor	*Dph1 /// ovca2*	1,38942817	0,00021938

suppressor in ovarian cancer 2			
Trophoblast glycoprotein	*Tpbg*	1,3882963	0,00299983
Star-related lipid transfer (start) domain containing 6	*Stard6*	1,38818298	0,00037451
Jun dimerization protein 2	*Jdp2*	1,38738687	0,00053113
Mitochondrial ubiquitin ligase activator of nfkb 1	*Mul1*	1,38724303	0,00451346
Wd repeat domain 55	*Wdr55*	1,38723635	0,00199321
Serine/threonine kinase 17b (apoptosis-inducing)	*Stk17b*	1,38705451	0,0021129
Meteorin, glial cell differentiation regulator	*Metrn*	1,38696411	0,00061741
Negative regulator of ubiquitin-like proteins 1	*Nub1*	1,3868764	0,00318333
Torsin family 1, member a (torsin a)	*Tor1a*	1,38679562	0,00376803
Growth factor, erv1 (s. Cerevisiae)-like (augmenter of liver regeneration)	*Gfer*	1,38600452	0,0018523
Heme oxygenase (decycling) 2	*Hmox2*	1,3852929	0,00042846
Secis binding protein 2-like	*Secisbp2l*	1,38505253	0,00165295
Eukaryotic translation elongation factor 1 epsilon 1	*Eef1e1*	1,38502764	0,00362601
Discs, large homolog 1 (drosophila)	*Dlg1*	1,38435127	0,00091952
Interleukin 17d	*Il17d*	1,38419024	0,00242118
Mob kinase activator 2	*Mob2*	1,38386746	0,00072934
Guanosine diphosphate (gdp) dissociation inhibitor 1	*Gdi1*	1,38381174	0,00547171
Ctage family, member 5	*Ctage5*	1,38330371	0,00316921
Protein tyrosine phosphatase-like (proline instead of catalytic arginine), member a	*Ptpla*	1,38320892	0,00075951
Potassium channel tetramerisation domain containing 20	*Kctd20*	1,38289063	0,00296321
Pten induced putative kinase 1	*Pink1*	1,38276756	0,00064752
Fumarate hydratase 1	*Fh1*	1,38224409	0,0004848
Translocase of inner mitochondrial membrane 17a	*Timm17a*	1,38217229	0,00151857
Autophagy related 16-like 1 (s. Cerevisiae)	*Atg16l1*	1,38204208	0,0055567
Inositol polyphosphate-1-phosphatase	*Inpp1*	1,38163842	0,00147198
Insulin-like growth factor i receptor	*Igf1r*	1,38136784	0,00394722
Yippee-like 1 (drosophila)	*Ypel1*	1,38119235	0,00746014
Smad family member 6	*Smad6*	1,38004098	0,0016062
Tankyrase, trf1-interacting ankyrin-related adp-ribose polymerase 2	*Tnks2*	1,37919987	0,00057269
Torsin family 1, member b	*Tor1b*	1,37878173	0,0062114
Mus musculus 8 days embryo whole body cdna, riken full-length enriched library, clone:5730407p16	*Ak077428 /// gm16536 ///*	1,37866218	0,00928364

product:unclassifiable, full insert sequence. /// --- /// predicted gene 16536	*gm16536*		
Ferritin light chain 1 /// predicted gene, 20746 /// ferritin light chain 1-like	*Ftl1 /// gm20746 /// loc100862446*	1,37833587	0,00018834
Dead (asp-glu-ala-asp) box polypeptide 19b	*Ddx19b*	1,3780859	0,00293635
Proteasome (prosome, macropain) 26s subunit, non-atpase, 5	*Psmd5*	1,37806851	0,00645871
Solute carrier family 25, member 28	*Slc25a28*	1,37784131	0,00277732
Transmembrane protein 42	*Tmem42*	1,3776077	0,00046633
Phosphatidic acid phosphatase type 2b	*Ppap2b*	1,37732063	0,00146111
Xylosylprotein beta1,4-galactosyltransferase, polypeptide 7 (galactosyltransferase i)	*B4galt7*	1,37715108	0,00546333
Nuclear cap binding protein subunit 2	*Ncbp2*	1,37620497	0,0008092
5-methyltetrahydrofolate-homocysteine methyltransferase reductase	*Mtrr*	1,37608391	0,00079891
Tetratricopeptide repeat domain 33	*Ttc33*	1,37531225	0,00650189
Phosphatidylinositol glycan anchor biosynthesis, class q	*Pigq*	1,37517841	0,00162828
Mesencephalic astrocyte-derived neurotrophic factor	*Manf*	1,37475371	0,00047195
Carcinoembryonic antigen-related cell adhesion molecule 1 /// carcinoembryonic antigen-related cell adhesion molecule 2	*Ceacam1 /// ceacam2*	1,37446629	0,00069895
Riken cdna 2410015m20 gene	*2410015m20rik*	1,3744155	0,00042666
Kri1 homolog (s. Cerevisiae)	*Kri1*	1,37406077	0,00210919
Mitochondrial ribosomal protein l45	*Mrpl45*	1,37378403	0,00106204
Transmembrane emp24 protein transport domain containing 5	*Tmed5*	1,3735133	0,00746014
Intraflagellar transport 172	*Ift172*	1,37270671	0,00090512
Ubiquitin specific peptidase 10	*Usp10*	1,37216292	0,00732239
Aminoacyl trna synthetase complex-interacting multifunctional protein 1	*Aimp1*	1,37183287	0,00086907
Solute carrier family 35 (udp-n-acetylglucosamine (udp-glcnac) transporter), member 3	*Slc35a3*	1,3714697	0,00203335
Reduced expression 2	*Rex2*	1,37145731	0,00231183
Prolylcarboxypeptidase (angiotensinase c)	*Prcp*	1,37120924	0,00822584
Presenilin associated, rhomboid-like	*Parl*	1,37101077	0,00571392
C-type lectin domain family 2, member h	*Clec2h*	1,37066801	0,00133778
Leucine rich repeat containing 8a /// phytanoyl-coa	*Lrrc8a /// phyhd1*	1,37037111	0,00240372

dioxygenase domain containing 1			
Mannose phosphate isomerase	*Mpi*	1,37027154	0,0006222
Hexosaminidase a	*Hexa*	1,37010029	0,00035066
Apoptosis-inducing factor, mitochondrion-associated 1	*Aifm1*	1,36981959	0,00121652
Cytidine deaminase	*Cda*	1,36968147	0,00121958
Solute carrier family 11 (proton-coupled divalent metal ion transporters), member 1	*Slc11a1*	1,36954326	0,00458178
Pyroglutamyl-peptidase i	*Pgpep1*	1,36953052	0,00100019
Zinc finger protein 280c	*Zfp280c*	1,36943181	0,0040226
Pro-platelet basic protein	*Ppbp*	1,36927065	0,00346648
Elac homolog 2 (e. Coli)	*Elac2*	1,36903717	0,00114401
Signal sequence receptor, beta	*Ssr2*	1,36872201	0,00063821
Glutamine-rich 1	*Qrich1*	1,36809934	0,00047417
Serine/threonine kinase 16	*Stk16*	1,36786159	0,00370534
Casp8 and fadd-like apoptosis regulator	*Cflar*	1,36765636	0,00076633
Taf13 rna polymerase ii, tata box binding protein (tbp)-associated factor	*Taf13*	1,36709555	0,00113347
Secretory carrier membrane protein 1	*Scamp1*	1,36706852	0,00154139
Mitochondrial ribosomal protein l41	*Mrpl41*	1,36696221	0,00383083
Zinc finger protein 654	*Zfp654*	1,36656669	0,00780233
Sr-related ctd-associated factor 4	*Scaf4*	1,36628966	0,00677835
Adp-ribosylation factor-like 6	*Arl6*	1,36621408	0,00858393
Metastasis associated 1	*Mta1*	1,36604619	0,00728953
Eukaryotic translation initiation factor 2b, subunit 2 beta	*Eif2b2*	1,36574456	0,00367869
Tsr1 20s rrna accumulation	*Tsr1*	1,36531258	0,00109963
Myotubularin related protein 1	*Mtmr1*	1,36504983	0,00023477
Diablo homolog (drosophila)	*Diablo*	1,36441226	0,00083349
Grb10 interacting gyf protein 2	*Gigyf2*	1,36439776	0,00071844
Diphthamine biosynethesis 7	*Dph7*	1,36410553	0,00102691
Ribosomal protein l35	*Rpl35*	1,3635973	0,00071343
Eukaryotic translation initiation factor 3, subunit j1 /// eukaryotic translation initiation factor 3, subunit j2	*Eif3j1 /// eif3j2*	1,36321704	0,00668498
Wd repeat domain 48	*Wdr48*	1,36320212	0,00168108
Riken cdna 6330416g13 gene	*6330416g13rik*	1,36224362	0,0064342
Predicted gene 7285 /// ribosomal protein s7	*Gm7285 /// rps7*	1,36217145	0,0026667

Predicted pseudogene 9385 /// ribosomal protein l24	*Gm9385 /// rpl24*	1,36216923	0,00028524
Upf2 regulator of nonsense transcripts homolog (yeast)	*Upf2*	1,36189298	0,00071427
Polymerase (rna) i polypeptide e	*Polr1e*	1,36185197	0,00612849
Coordinator of prmt5, differentiation stimulator	*Coprs*	1,36166451	0,00086182
Transmembrane protein 161a	*Tmem161a*	1,36147637	0,00043582
Methyl cpg binding protein 2	*Mecp2*	1,36145459	0,00127993
Casein kinase 1, delta	*Csnk1d*	1,3608109	0,00442723
N-acylsphingosine amidohydrolase 1	*Asah1*	1,36002106	0,0017004
Atp-binding cassette, sub-family f (gcn20), member 3	*Abcf3*	1,35986394	0,00322906
Slit-robo rho gtpase activating protein 1	*Srgap1*	1,35977121	0,00551035
Rna-binding region (rnp1, rrm) containing 3	*Rnpc3*	1,35968812	0,00722468
Family with sequence similarity 50, member a	*Fam50a*	1,35955073	0,00103352
Inositol 1,4,5-trisphosphate receptor 1	*Itpr1*	1,35913414	0,00066273
Rna binding motif protein 4b	*Rbm4b*	1,35862653	0,0050737
Stomatin (epb7.2)-like 2	*Stoml2*	1,3585339	0,00119162
At rich interactive domain 5a (mrf1-like)	*Arid5a*	1,3583953	0,00209079
Fyve and coiled-coil domain containing 1	*Fyco1*	1,35813557	0,00241148
Protein phosphatase 1, regulatory (inhibitor) subunit 15b	*Ppp1r15b*	1,35803156	0,00060066
Predicted gene 2808 /// tubby like protein 4	*Gm2808 /// tulp4*	1,35798806	0,00347495
Testis expressed gene 10	*Tex10*	1,35765945	0,00045089
Required for meiotic nuclear division 1 pseudogene /// required for meiotic nuclear division 1 homolog (s. Cerevisiae)	*Gm5512 /// rmnd1*	1,35750853	0,00183074
Expressed sequence ai314180	*Ai314180*	1,35730239	0,00212096
Trimethylguanosine synthase homolog (s. Cerevisiae)	*Tgs1*	1,35683294	0,00608003
Acyl-coenzyme a dehydrogenase, short/branched chain	*Acadsb*	1,35652406	0,00214542
M-phase phosphoprotein 10 (u3 small nucleolar ribonucleoprotein)	*Mphosph10*	1,35647494	0,00203965
Epidermal growth factor-containing fibulin-like extracellular matrix protein 2	*Efemp2*	1,35613734	0,00099441
Nadh dehydrogenase (ubiquinone) fe-s protein 4	*Ndufs4*	1,35549853	0,00344105
Surfeit gene 2	*Surf2*	1,35545771	0,00257835
Phosphomannomutase 1	*Pmm1*	1,35513618	0,0034256
Coenzyme q4 homolog (yeast)	*Coq4*	1,35497069	0,00082966

Hippocampus abundant gene transcript 1	*Hiat1*	1,35491609	0,00715539
Sumo/sentrin specific peptidase 2	*Senp2*	1,35488282	0,00322182
Ring finger protein 214	*Rnf214*	1,35413751	0,00685461
Cadherin 13	*Cdh13*	1,35411394	0,0013628
Akirin 2	*Akirin2*	1,35403734	0,00100232
Udp-gal:betaglcnac beta 1,4-galactosyltransferase, polypeptide 3	*B4galt3*	1,35380046	0,00129647
Cdp-diacylglycerol--inositol 3-phosphatidyltransferase (phosphatidylinositol synthase)	*Cdipt*	1,35343241	0,00080379
Heat shock protein 1 (chaperonin)	*Hspd1*	1,35290744	0,0036955
Sterile alpha motif domain containing 4	*Samd4*	1,35134103	0,00397338
Flotillin 1	*Flot1*	1,35079662	0,00323719
Nth (endonuclease iii)-like 1 (e.coli)	*Nthl1*	1,35063177	0,0008837
Spectrin beta, non-erythrocytic 1	*Sptbn1*	1,35048233	0,0010338
Tox high mobility group box family member 4	*Tox4*	1,35030056	0,00148071
Golgi associated, gamma adaptin ear containing, arf binding protein 1	*Gga1*	1,35020091	0,00326354
Sorting nexin 14	*Snx14*	1,34957442	0,00961606
Heat shock factor binding protein 1	*Hsbp1*	1,3495631	0,00046654
Small nuclear ribonucleoprotein 48 (u11/u12)	*Snrnp48*	1,34918866	0,00570279
Breast carcinoma amplified sequence 3	*Bcas3*	1,34904912	0,0032547
Mis12 homolog (yeast)	*Mis12*	1,34889131	0,001871
Rna polymerase ii associated protein 1	*Rpap1*	1,34882186	0,00070798
Glucosaminyl (n-acetyl) transferase 3, mucin type	*Gcnt3*	1,34879519	0,00299983
Riken cdna 1810026j23 gene	*1810026j23rik*	1,34864646	0,00057618
Transducin-like enhancer of split 6, homolog of drosophila e(spl)	*Tle6*	1,34814766	0,00150814
Family with sequence similarity 220, member a	*Fam220a*	1,34808948	0,00120974
Glutathione s-transferase, mu 2	*Gstm2*	1,34807212	0,00116459
Basic leucine zipper nuclear factor 1	*Blzf1*	1,34784837	0,00743821
Vacuolar protein sorting 54 (yeast)	*Vps54*	1,3477572	0,00375628
Oxidative-stress responsive 1	*Oxsr1*	1,34773006	0,00739893
Cell division cycle associated 7	*Cdca7*	1,34765519	0,00789283
Ciliary neurotrophic factor /// zinc finger protein 91 /// zfp91-cntf readthrough transcript (nmd candidate)	*Cntf /// zfp91 /// zfp91cntf*	1,34713261	0,00284465
Glycine cleavage system protein h (aminomethyl	*Gcsh*	1,34682104	0,00071401

carrier)			
Dynein cytoplasmic 1 heavy chain 1	*Dync1h1*	1,34661058	0,00176349
Cyclin-dependent kinase 5	*Cdk5*	1,34638289	0,00268569
Neurofibromatosis 1	*Nf1*	1,34632647	0,00582227
Chromatin target of prmt1	*Chtop*	1,34629261	0,00061967
Eukaryotic translation initiation factor 4a2	*Eif4a2*	1,34619061	0,00740536
Start domain containing 3	*Stard3*	1,34605371	0,00333333
Male-specific lethal 2 homolog (drosophila)	*Msl2*	1,34604871	0,00741413
Phospholipase a2, group xiia	*Pla2g12a*	1,34531022	0,00289939
Gtf2i repeat domain containing 2	*Gtf2ird2*	1,34528582	0,00104673
Wd repeat domain 61	*Wdr61*	1,34524698	0,00119433
Serine (or cysteine) peptidase inhibitor, clade h, member 1	*Serpinh1*	1,34515521	0,00178485
Nudix (nucleoside diphosphate linked moiety x)-type motif 22	*Nudt22*	1,34511345	0,00183586
Interleukin 18 receptor accessory protein	*Il18rap*	1,3446796	0,00564447
Small integral membrane protein 19	*Smim19*	1,34422467	0,00096218
Protein kinase, camp dependent, catalytic, beta	*Prkacb*	1,34420818	0,0008665
Wd repeat, sam and u-box domain containing 1	*Wdsub1*	1,34398165	0,00322906
Nol1/nop2/sun domain family, member 4	*Nsun4*	1,34337264	0,0045636
Numb-like	*Numbl*	1,34293187	0,0041639
Elongator acetyltransferase complex subunit 3	*Elp3*	1,34287548	0,00252493
Golgi integral membrane protein 4	*Golim4*	1,3424914	0,00632128
High mobility group 20a	*Hmg20a*	1,34227102	0,00088457
Adaptor-related protein complex 1 associated regulatory protein	*Ap1ar*	1,34214388	0,00228295
Intersectin 2	*Itsn2*	1,34205856	0,00723581
Proteasome (prosome, macropain) subunit, beta type 10	*Psmb10*	1,34198717	0,00675416
Nmda receptor synaptonuclear signaling and neuronal migration factor	*Nsmf*	1,34198164	0,00357393
Predicted pseudogene 10913 /// predicted gene 12447 /// predicted gene 12508 /// predicted pseudogene 8210 /// 60s ribosomal protein l29-like /// 60s ribosomal protein l29-like /// ribosomal protein l29	*Gm10913 /// gm12447 /// gm12508 /// gm8210 /// loc100503055 /// loc102642233 /// rpl29*	1,34178569	0,00089286
Importin 4	*Ipo4*	1,3417503	0,00714285

Set and mynd domain containing 2	*Smyd2*	1,34172532	0,00243102
Proviral integration site 1	*Pim1*	1,34157145	0,0012748
Opioid growth factor receptor	*Ogfr*	1,3403252	0,0023667
Nme/nm23 nucleoside diphosphate kinase 6	*Nme6*	1,34027979	0,00142936
Ferredoxin reductase	*Fdxr*	1,34027034	0,00050063
Eukaryotic translation initiation factor 1 /// predicted pseudogene 5471	*Eif1 /// gm5471*	1,33988727	0,0021332
Poly (adp-ribose) polymerase family, member 16	*Parp16*	1,33976139	0,00509173
Solute carrier family 11 (proton-coupled divalent metal ion transporters), member 2	*Slc11a2*	1,33970316	0,00129693
Nima (never in mitosis gene a)-related expressed kinase 9	*Nek9*	1,33952721	0,00055117
Zinc finger, an1-type domain 3	*Zfand3*	1,33952712	0,00543047
Brain and reproductive organ-expressed protein	*Bre*	1,33941798	0,00175026
Elongator acetyltransferase complex subunit 2	*Elp2*	1,33914252	0,00131306
Inhibitor of kappab kinase beta	*Ikbkb*	1,3386815	0,00317157
Coagulation factor viii	*F8*	1,33867984	0,00212155
F-box and wd-40 domain protein 7	*Fbxw7*	1,33857151	0,00714958
Acireductone dioxygenase 1	*Adi1*	1,33850927	0,00323663
Rna binding motif protein 5	*Rbm5*	1,33846294	0,00863109
Cell division cycle 37-like 1	*Cdc37l1*	1,33833892	0,00176696
Mitochondrial ribosomal protein l3	*Mrpl3*	1,33819054	0,00372104
Peptidylprolyl isomerase (cyclophilin)-like 2	*Ppil2*	1,33793261	0,00934565
Protein tyrosine phosphatase-like a domain containing 1	*Ptplad1*	1,3378043	0,0022835
Tissue inhibitor of metalloproteinase 1	*Timp1*	1,33773532	0,00101601
Melanoma antigen, family d, 1	*Maged1*	1,33764496	0,00106339
Phosphodiesterase 6d, cgmp-specific, rod, delta	*Pde6d*	1,33752183	0,0015058
Matrix metallopeptidase 19	*Mmp19*	1,33728578	0,00580171
Ik cytokine	*Ik*	1,33727791	0,00077909
Grpe-like 2, mitochondrial	*Grpel2*	1,33705431	0,00160462
Cdna sequence bc023829	*Bc023829*	1,33600386	0,00087432
Btb (poz) domain containing 10	*Btbd10*	1,33518842	0,00261928
Intraflagellar transport 57	*Ift57*	1,334164	0,00073651
Cytotoxic t lymphocyte-associated protein 2 alpha	*Ctla2a*	1,33401975	0,0013697
Mannoside acetylglucosaminyltransferase 1	*Mgat1*	1,33394746	0,00305086
A disintegrin and metallopeptidase domain 10	*Adam10*	1,3338209	0,0047717
Membrane-associated ring finger (c3hc4) 5	*Iii.05*	1,33375374	0,000737

Huntingtin interacting protein k /// small edrk-rich factor 2	*Hypk /// serf2*	1,33284231	0,00086467
Stress-associated endoplasmic reticulum protein 1	*Serp1*	1,3326115	0,00444626
Run and fyve domain containing 3	*Rufy3*	1,3325576	0,0098848
Eukaryotic translation initiation factor 5b	*Eif5b*	1,33246952	0,00897907
Rna pseudouridylate synthase domain containing 4	*Rpusd4*	1,33210662	0,00087353
Mediator complex subunit 1	*Med1*	1,33186007	0,00144375
Ubiquitin-like modifier activating enzyme 5	*Uba5*	1,33179003	0,00087241
Fic domain containing	*Ficd*	1,33110618	0,00789226
Endoplasmic reticulum metallopeptidase 1	*Ermp1*	1,33105243	0,00688392
Mitochondrial ribosomal protein l12	*Mrpl12*	1,33059872	0,00075387
Fasciculation and elongation protein zeta 2 (zygin ii)	*Fez2*	1,33019344	0,0005663
Uv radiation resistance associated gene	*Uvrag*	1,32985685	0,0013697
Ribosomal protein s7	*Rps7*	1,32977338	0,00109963
Late cornified envelope 1f	*Lce1f*	1,32957725	0,00650609
Large subunit gtpase 1 homolog (s. Cerevisiae)	*Lsg1*	1,32930392	0,00689096
Solute carrier family 25, member 30	*Slc25a30*	1,32922516	0,00603852
Atp-binding cassette, sub-family b (mdr/tap), member 9	*Abcb9*	1,32918034	0,00518708
Acyl-coenzyme a oxidase 1, palmitoyl	*Acox1*	1,32887028	0,00063584
Secreted phosphoprotein 1	*Spp1*	1,32882477	0,00321777
Abhydrolase domain containing 4	*Abhd4*	1,32850013	0,00296321
Histone cluster 1, h2bp	*Hist1h2bp*	1,32815163	0,00391278
Zinc finger protein 354b	*Zfp354b*	1,32808415	0,00094885
Peroxisomal biogenesis factor 6	*Pex6*	1,32789455	0,00996641
Myosin, light chain 12b, regulatory	*Myl12b*	1,32785904	0,00104396
Wd repeat domain, phosphoinositide interacting 2	*Wipi2*	1,32725188	0,0018571
Polyadenylate-binding protein-interacting protein 2	*Paip2*	1,32721851	0,00395219
Retinol dehydrogenase 13 (all-trans and 9-cis)	*Rdh13*	1,32698051	0,00278018
Dynactin 3	*Dctn3*	1,32680366	0,0031207
Alkb, alkylation repair homolog 4 (e. Coli)	*Alkbh4*	1,32633273	0,00522702
Prosaposin	*Psap*	1,32631186	0,0023667
Thap domain containing 7	*Thap7*	1,3260543	0,00830914
Oxidative stress responsive serine rich 1	*Oser1*	1,32580303	0,00131508
Membrane metallo endopeptidase	*Mme*	1,32532324	0,00459943
Ran, member ras oncogene family	*Ran*	1,32508419	0,00639988
Max binding protein	*Mnt*	1,32478279	0,00781797
Mindbomb homolog 2 (drosophila)	*Mib2*	1,32461466	0,0063252

Sh3-domain binding protein 2	*Sh3bp2*	1,324575	0,00480858
Cytochrome b-561 domain containing 2	*Cyb561d2*	1,32425875	0,00386891
Transmembrane protein 230	*Tmem230*	1,3241962	0,00169184
Dnl-type zinc finger	*Dnlz*	1,32372173	0,00193858
Riken cdna 4932438a13 gene	*4932438a13rik*	1,32367207	0,00910443
Riken cdna 1600014c10 gene	*1600014c10rik*	1,32357434	0,00268684
Arginyl-trna synthetase	*Rars*	1,32305343	0,00187838
Metal response element binding transcription factor 1	*Mtf1*	1,3226859	0,00402692
Cdna sequence bc017643	*Bc017643*	1,32239282	0,00228085
Zinc finger protein 318	*Zfp318*	1,32224208	0,0014909
Gc-rich promoter binding protein 1	*Gpbp1*	1,32223625	0,00344679
Zinc finger protein 287	*Zfp287*	1,32202545	0,00700704
Rwd domain containing 4a	*Rwdd4a*	1,32199684	0,00831392
Riken cdna 2810403a07 gene	*2810403a07rik*	1,32194554	0,0068421
Vacuolar protein sorting 11 (yeast)	*Vps11*	1,32181555	0,00357032
Inositol hexaphosphate kinase 1	*Ip6k1*	1,32160084	0,00958554
Ribonuclease p/mrp 25 subunit-like	*Rpp25l*	1,32141339	0,00158779
Dual specificity phosphatase 3 (vaccinia virus phosphatase vh1-related)	*Dusp3*	1,32135001	0,0088987
Jagunal homolog 1 (drosophila)	*Jagn1*	1,32132492	0,00330018
Nadh dehydrogenase (ubiquinone) 1 alpha subcomplex, 13	*Ndufa13*	1,32124036	0,00307471
Translocated promoter region, nuclear basket protein	*Tpr*	1,3208708	0,00344966
Vacuolar protein sorting 45 (yeast)	*Vps45*	1,32079887	0,00326618
Isoleucine-trna synthetase	*Iars*	1,319945	0,00187073
Programmed cell death 2	*Pdcd2*	1,31940475	0,00244794
Protein kinase, interferon inducible double stranded rna dependent activator	*Prkra*	1,31932777	0,00315256
Nme/nm23 nucleoside diphosphate kinase 2	*Nme2*	1,31930706	0,00100432
Atpase, h+ transporting, lysosomal v1 subunit g1	*Atp6v1g1*	1,31904027	0,00108424
Rna binding motif protein 28	*Rbm28*	1,31869214	0,00807134
Zinc finger protein 746	*Zfp746*	1,31851434	0,00156806
Reticulocalbin 1	*Rcn1*	1,31836252	0,00368403
Ubiquitin-conjugating enzyme e2h	*Ube2h*	1,31800774	0,0050093
Riken cdna 1110037f02 gene	*1110037f02rik*	1,31782726	0,00181209
Swi/snf related, matrix associated, actin dependent	*Smarcc1*	1,31738651	0,00207384

regulator of chromatin, subfamily c, member 1			
Mediator complex subunit 19	*Med19*	1,31737694	0,00496726
Gar1 ribonucleoprotein homolog (yeast)	*Gar1*	1,31729581	0,00082966
Zinc finger, ran-binding domain containing 1	*Zranb1*	1,31684059	0,00564447
Family with sequence similarity 45, member a	*Fam45a*	1,31678701	0,00382877
Dnaj (hsp40) homolog, subfamily b, member 2	*Dnajb2*	1,31665652	0,00306167
Signal transducer and activator of transcription 3	*Stat3*	1,31665263	0,00308547
Tripartite motif-containing 11	*Trim11*	1,31662195	0,00201162
Zinc finger protein 280d	*Zfp280d*	1,3161894	0,00444119
Cystatin b	*Cstb*	1,31492341	0,00267199
Tip41, tor signalling pathway regulator-like (s. Cerevisiae)	*Tiprl*	1,31491152	0,00829076
Rna binding motif protein 8a	*Rbm8a*	1,31467352	0,00820786
High mobility group box transcription factor 1	*Hbp1*	1,31455557	0,0068216
Enoyl coenzyme a hydratase 1, peroxisomal	*Ech1*	1,31427921	0,00299985
Dihydrouridine synthase 1-like (s. Cerevisiae)	*Dus1l*	1,31405999	0,0027707
Proteasome (prosome, macropain) 26s subunit, non-atpase, 4	*Psmd4*	1,31226165	0,00283158
Glia maturation factor, beta	*Gmfb*	1,31218208	0,00178537
Coiled-coil domain containing 71	*Ccdc71*	1,31207935	0,00563344
Activator of basal transcription 1	*Abt1*	1,31199089	0,00339483
Start domain containing 7	*Stard7*	1,31196152	0,00162045
Synovial sarcoma, x breakpoint 2 interacting protein	*Ssx2ip*	1,31134157	0,00184137
Predicted pseudogene 10913 /// predicted gene 12508 /// predicted pseudogene 8210 /// 60s ribosomal protein l29-like /// 60s ribosomal protein l29-like /// ribosomal protein l29	*Gm10913 /// gm12508 /// gm8210 /// loc100503055 /// loc102642233 /// rpl29*	1,31121963	0,00157806
Nrde-2 necessary for rna interference, domain containing	*Nrde2*	1,31093039	0,00237451
Midasin homolog (yeast)	*Mdn1*	1,3108304	0,00589619
Predicted pseudogene 4024 /// transmembrane emp24-like trafficking protein 10 (yeast)	*Gm4024 /// tmed10*	1,31019259	0,00445772
Mitochondrial ribosomal protein l19	*Mrpl19*	1,31010358	0,00243102
Atp synthase, h+ transporting, mitochondrial f0 complex, subunit b1	*Atp5f1*	1,30987497	0,00147043
Charged multivesicular body protein 5	*Chmp5*	1,30975186	0,00331761
Zinc finger and btb domain containing 2	*Zbtb2*	1,30761014	0,00257969

Autophagy related 4b, cysteine peptidase	*Atg4b*	1,30691541	0,00236225
Leucine rich repeat containing 2	*Lrrc2*	1,30668406	0,00839668
Haloacid dehalogenase-like hydrolase domain containing 3	*Hdhd3*	1,30667513	0,00577514
Catenin (cadherin associated protein), beta 1	*Ctnnb1*	1,3062004	0,00660416
Ribosomal l1 domain containing 1	*Rsl1d1*	1,30555532	0,00392701
Riken cdna 2900097c17 gene	*2900097c17rik*	1,3053543	0,00087703
Lon peptidase 1, mitochondrial	*Lonp1*	1,30435038	0,00557272
T-box 15	*Tbx15*	1,30423841	0,0044755
Acyl-coenzyme a dehydrogenase, very long chain	*Acadvl*	1,30410098	0,00415926
Poly(a)-specific ribonuclease (deadenylation nuclease)	*Parn*	1,30384167	0,00275905
Protein phosphatase 2 (formerly 2a), regulatory subunit b'', delta	*Ppp2r3d*	1,30376274	0,0077299
Mediator complex subunit 25	*Med25*	1,30373901	0,00785176
Nad(p)h dehydrogenase, quinone 1	*Nqo1*	1,30283513	0,00246621
Smt3 suppressor of mif two 3 homolog 1 (yeast)	*Sumo1*	1,30235527	0,00111027
N-terminal asn amidase	*Ntan1*	1,30181727	0,00696034
Atpase, h+ transporting, lysosomal v0 subunit a2	*Atp6v0a2*	1,30163351	0,00321351
Suppressor of variegation 3-9 homolog 2 (drosophila)	*Suv39h2*	1,30146981	0,00292661
Gene rich cluster, c10 gene	*Grcc10*	1,30139597	0,00422972
Glial cell line derived neurotrophic factor	*Gdnf*	1,30100633	0,00144965
Phd finger protein 13	*Phf13*	1,30080819	0,00279617
Zinc finger protein 623	*Zfp623*	1,29992666	0,00322537
Serine palmitoyltransferase, long chain base subunit 2	*Sptlc2*	1,29919894	0,0036052
Ring finger and wd repeat domain 2	*Rfwd2*	1,29912006	0,00276416
Tetratricopeptide repeat domain 39c	*Ttc39c*	1,29869361	0,0033647
Serine/arginine-rich splicing factor 6	*Srsf6*	1,29868439	0,00336526
Dnaj (hsp40) homolog, subfamily b, member 4	*Dnajb4*	1,29837026	0,00609195
Ankyrin repeat domain 24	*Ankrd24*	1,29831723	0,00663128
Smek homolog 2, suppressor of mek1 (dictyostelium)	*Smek2*	1,2982152	0,00453693
Predicted gene 15483 /// 40s ribosomal protein s13-like /// ribosomal protein s13 /// ribosomal protein s13, pseudogene 4	*Gm15483 /// loc102642137 /// rps13 /// rps13-ps4*	1,29814073	0,00173715
Succinyl-coa glutarate-coa transferase	*Sugct*	1,29754042	0,00402368

Proline, glutamic acid and leucine rich protein 1	*Pelp1*	1,29747701	0,00757378
Shisa homolog 5 (xenopus laevis)	*Shisa5*	1,29714865	0,00140663
Riken cdna 2600001m11 gene /// nudix (nucleoside diphosphate linked moiety x)-type motif 5	*2600001m11rik /// nudt5*	1,29691913	0,00630771
Coiled-coil domain containing 50	*Ccdc50*	1,29614873	0,00168846
Oxysterol binding protein-like 2	*Osbpl2*	1,29599091	0,00184756
Serine (or cysteine) peptidase inhibitor, clade d, member 1	*Serpind1*	1,29560365	0,00165171
Apoptosis enhancing nuclease	*Aen*	1,295433	0,00760943
Heterogeneous nuclear ribonucleoprotein a3 pseudogene /// heterogeneous nuclear ribonucleoprotein a3	*Gm6793 /// hnrnpa3*	1,29522663	0,00282018
Integral membrane protein 2c	*Itm2c*	1,29491563	0,00404609
Mannosidase, alpha, class 1a, member 2	*Man1a2*	1,29458483	0,0055567
Nudix (nucleoside diphosphate linked moiety x)-type motif 19	*Nudt19*	1,29439987	0,00210643
Bola-like 2 (e. Coli)	*Bola2*	1,29427614	0,00604758
Digeorge syndrome critical region gene 14	*Dgcr14*	1,29321654	0,00799805
Transmembrane emp24 domain containing 3	*Tmed3*	1,29318344	0,00197423
Atp synthase, h+ transporting, mitochondrial f0 complex, subunit s	*Atp5s*	1,29158664	0,00447937
Coiled-coil domain containing 23	*Ccdc23*	1,29143385	0,00118179
Predicted pseudogene 6238 /// mitochondrial ribosomal protein l30	*Gm6238 /// mrpl30*	1,29130468	0,00251601
Ketohexokinase	*Khk*	1,29088678	0,00859242
Trypsin domain containing 1	*Tysnd1*	1,29075301	0,00636516
Mechanistic target of rapamycin (serine/threonine kinase)	*Mtor*	1,28996695	0,00872072
Sh2 domain protein 1b1 /// sh2 domain protein 1b2	*Sh2d1b1 /// sh2d1b2*	1,28968046	0,00907893
Predicted gene 4705 /// predicted gene 6404 /// ribosomal protein l34 /// ribosomal protein l34, pseudogene 1	*Gm4705 /// gm6404 /// rpl34 /// rpl34-ps1*	1,28905394	0,00279933
Retinoic acid induced 1	*Rai1*	1,28903642	0,00562609
Stromal cell derived factor 2	*Sdf2*	1,28875605	0,00433927
Mitogen-activated protein kinase kinase 5	*Map2k5*	1,28869391	0,00629459
Dehydrogenase/reductase (sdr family) member 7	*Dhrs7*	1,28864819	0,00380244
Suppression of tumorigenicity 7-like	*St7l*	1,28822825	0,00679931
Predicted gene 3258 /// suppressor of ty 4a	*Gm3258 /// supt4a*	1,28780112	0,00202726

Mediator complex subunit 31	*Med31*	1,28735245	0,00765435
Asparaginase like 1	*Asrgl1*	1,28702632	0,00588691
Grpe-like 1, mitochondrial	*Grpel1*	1,28696046	0,00244918
Lysine (k)-specific demethylase 4c	*Kdm4c*	1,28685735	0,0034812
Serologically defined colon cancer antigen 3	*Sdccag3*	1,28644843	0,00437688
Regulator of microtubule dynamics 1	*Rmdn1*	1,28625069	0,00824927
Peroxisomal biogenesis factor 14	*Pex14*	1,28613076	0,00609537
Perilipin 2	*Plin2*	1,28611306	0,00174965
Hcls1 binding protein 3	*Hs1bp3*	1,28590844	0,00434701
Glucosamine-6-phosphate deaminase 1	*Gnpda1*	1,28515553	0,00397039
Suppressor of cytokine signaling 3	*Socs3*	1,28500155	0,00593663
Protein kinase c and casein kinase substrate in neurons 3	*Pacsin3*	1,28478347	0,00315291
Zinc finger, ccch-type with g patch domain	*Zgpat*	1,28460648	0,00243075
Activating transcription factor 2	*Atf2*	1,28451922	0,00247977
Complement component factor h	*Cfh*	1,28448752	0,00378658
Autophagy related 101	*Atg101*	1,28408528	0,00704237
Aquarius	*Aqr*	1,28386916	0,00269146
Nucleolar and coiled-body phosphoprotein 1	*Nolc1*	1,28386764	0,00486256
Archaelysin family metallopeptidase 2	*Amz2*	1,28305864	0,00331808
Endoglin	*Eng*	1,28213291	0,00562412
Nadh dehydrogenase (ubiquinone) flavoprotein 1	*Ndufv1*	1,28211377	0,00928935
Predicted pseudogene 10913 /// predicted gene 11449 /// predicted gene 12508 /// predicted gene 13213 /// predicted pseudogene 8210 /// 60s ribosomal protein l29-like /// 60s ribosomal protein l29-like /// ribosomal protein l29	*Gm10913 /// gm11449 /// gm12508 /// gm13213 /// gm8210 /// loc100503055 /// loc102642233 /// rpl29*	1,28205984	0,00130387
Adnp homeobox 2	*Adnp2*	1,28195435	0,00552804
Cd2 antigen (cytoplasmic tail) binding protein 2	*Cd2bp2*	1,28161226	0,00185621
Sumo/sentrin specific peptidase 3	*Senp3*	1,27952714	0,00495453
Inositol polyphosphate phosphatase-like 1	*Inppl1*	1,27871777	0,00726507
Polysaccharide biosynthesis domain containing 1	*Pbdc1*	1,27859763	0,00197579
Trafficking protein particle complex 12	*Trappc12*	1,27855446	0,00196601
Glutaminyl-trna synthetase	*Qars*	1,27835869	0,00171886
Serine/arginine-rich protein specific kinase 2	*Srpk2*	1,27789505	0,00404603
Charged multivesicular body protein 7	*Chmp7*	1,27769662	0,00515954

Far upstream element (fuse) binding protein 1	*Fubp1*	1,27652452	0,00627459
General transcription factor iiic, polypeptide 6, alpha	*Gtf3c6*	1,27612644	0,00841383
Ubiquitin pseudogene /// ubiquitin b	*Gm1821 /// ubb*	1,27605357	0,00128034
Nadh dehydrogenase (ubiquinone) 1, subcomplex unknown, 1	*Ndufc1*	1,27528551	0,00558873
Ubiquitin-fold modifier 1	*Ufm1*	1,2740525	0,00904432
Thioredoxin-like 4a	*Txnl4a*	1,27395574	0,0069058
Protein tyrosine phosphatase, receptor type, a	*Ptpra*	1,27329301	0,00766852
Er degradation enhancer, mannosidase alpha-like 2	*Edem2*	1,27311189	0,00871834
Deoxyhypusine synthase	*Dhps*	1,27283255	0,00317671
Nmd3 homolog (s. Cerevisiae)	*Nmd3*	1,27272837	0,00265942
Pyruvate dehydrogenase e1 alpha 1	*Pdha1*	1,26960471	0,00928689
Cyclin d2	*Ccnd2*	1,26959276	0,00293086
Karyopherin (importin) alpha 4	*Kpna4*	1,26938552	0,00889584
Protein arginine n-methyltransferase 1	*Prmt1*	1,26933688	0,00404118
Smu-1 suppressor of mec-8 and unc-52 homolog (c. Elegans)	*Smu1*	1,26881284	0,00243727
Peptidase (mitochondrial processing) alpha	*Pmpca*	1,26850572	0,00219738
Adp-ribosylarginine hydrolase	*Adprh*	1,2682769	0,00226074
V-myc myelocytomatosis viral related oncogene, neuroblastoma derived (avian)	*Mycn*	1,26794892	0,00735966
Aldo-keto reductase family 1, member c12	*Akr1c12*	1,26771613	0,00536704
Tripartite motif-containing 33	*Trim33*	1,26751168	0,00388256
Ring finger protein 25	*Rnf25*	1,26696372	0,00547938
Acyl-coa thioesterase 9	*Acot9*	1,26658607	0,00279367
Ccr4-not transcription complex, subunit 8	*Cnot8*	1,26651858	0,0048778
Set and mynd domain containing 5	*Smyd5*	1,26633746	0,00370742
Topoisomerase (dna) iii beta	*Top3b*	1,26618008	0,00534191
Nadh dehydrogenase (ubiquinone) 1 beta subcomplex, 6	*Ndufb6*	1,26599341	0,00919588
General transcription factor iiic, polypeptide 4	*Gtf3c4*	1,26572056	0,006999
Adp-ribosylation factor-like 5a	*Arl5a*	1,26559053	0,00295627
Myo-inositol 1-phosphate synthase a1	*Isyna1*	1,26543168	0,00392362
Heat shock protein 8	*Hspb8*	1,26433066	0,00242546
Seminal vesicle secretory protein 5	*Svs5*	1,26426383	0,00716981
Small nucleolar rna host gene 1	*Snhg1*	1,26342423	0,00527165
Dihydrolipoamide s-succinyltransferase (e2 component of 2-oxo-glutarate complex)	*Dlst*	1,26334517	0,00534191

Eukaryotic translation initiation factor 4e	*Eif4e*	1,26324927	0,00501542
Predicted gene 16340 /// interferon activated gene 203 /// flocculation protein flo11-like /// interferon-activable protein 203-like	*Gm16340 /// ifi203 /// loc100862473 /// loc102641031*	1,26314866	0,00905442
Apoptosis inhibitor 5	*Api5*	1,26312198	0,00310377
Predicted gene 10349 /// predicted pseudogene 9386 /// nuclear transport factor 2 /// nuclear transport factor 2, pseudogene 1	*Gm10349 /// gm9386 /// nutf2 /// nutf2-ps1*	1,26295615	0,00663325
Riken cdna 1110059g10 gene	*1110059g10rik*	1,26283949	0,00208592
Growth arrest specific 1	*Gas1*	1,2619253	0,00432707
Mitochondrial ribosomal protein s22	*Mrps22*	1,26101167	0,00721126
Tumor suppressing subtransferable candidate 1	*Tssc1*	1,25971773	0,00425834
Sirtuin 3	*Sirt3*	1,25969915	0,00450913
Zinc finger protein 319	*Zfp319*	1,25958452	0,00583585
Ras-related protein-1a	*Rap1a*	1,25888829	0,00749589
Actinin, alpha 1	*Actn1*	1,25839753	0,00409827
Filamin, alpha	*Flna*	1,25839212	0,00557115
Aminopeptidase-like 1	*Npepl1*	1,25824696	0,0045286
Moesin	*Msn*	1,25820399	0,00673293
Late endosomal/lysosomal adaptor, mapk and mtor activator 5	*Lamtor5*	1,2579041	0,00347187
U box domain containing 5	*Ubox5*	1,25765684	0,00497456
Riken cdna 1110051m20 gene	*1110051m20rik*	1,25719386	0,00437615
Ribosomal protein l12	*Rpl12*	1,25601562	0,00732606
Rna binding motif protein 17	*Rbm17*	1,2551658	0,00362873
Exostoses (multiple) 1	*Ext1*	1,25499983	0,00548599
Proteasome (prosome, macropain) subunit, beta type 5	*Psmb5*	1,25494679	0,00368154
Ep300 interacting inhibitor of differentiation 1	*Eid1*	1,25458968	0,00692395
Solute carrier family 23 (nucleobase transporters), member 2	*Slc23a2*	1,25433956	0,00837017
Interactor of little elongation complex ell subunit 1	*Ice1*	1,25423303	0,00985702
Ero1-like beta (s. Cerevisiae)	*Ero1lb*	1,25377305	0,00449424
Bleomycin hydrolase	*Blmh*	1,25287171	0,00896318
Glycerol-3-phosphate acyltransferase, mitochondrial	*Gpam*	1,25280786	0,00766459
Atp synthase, h+ transporting, mitochondrial f1 complex, alpha subunit 1	*Atp5a1*	1,25253473	0,00216188
Atp synthase, h+ transporting, mitochondrial f1	*Atp5c1*	1,25222143	0,00563636

complex, gamma polypeptide 1			
Set domain containing 3	*Setd3*	1,25204058	0,0033832
Mediator complex subunit 17	*Med17*	1,25196967	0,00661655
Serum amyloid a-like 1	*Saal1*	1,25155551	0,00894522
Nitrogen permease regulator-like 2	*Nprl2*	1,25132838	0,00998376
Potassium channel tetramerisation domain containing 5	*Kctd5*	1,25131679	0,00727452
Chromodomain helicase dna binding protein 1	*Chd1*	1,2510701	0,00770132
Receptor (tnfrsf)-interacting serine-threonine kinase 1	*Ripk1*	1,25049007	0,00828813
Syntrophin, acidic 1	*Snta1*	1,25047908	0,00493576
N-ethylmaleimide sensitive fusion protein attachment protein gamma	*Napg*	1,25017678	0,00559407
Phosphatidylinositol-4-phosphate 5-kinase, type 1 alpha	*Pip5k1a*	1,25011042	0,00814442
Integral membrane protein 2b	*Itm2b*	1,2497418	0,00578719
Stip1 homology and u-box containing protein 1	*Stub1*	1,24973162	0,00493827
Serum response factor	*Srf*	1,24951341	0,00541439
Myotubularin related protein 9	*Mtmr9*	1,24904884	0,00599541
Mterf domain containing 1	*Mterfd1*	1,24850827	0,00789642
Grancalcin	*Gca*	1,24813404	0,00954046
60s ribosomal protein l12-like /// uncharacterized loc102635609 /// 60s ribosomal protein l12-like /// ribosomal protein l12	*Loc102635048 /// loc102635609 /// loc102640707 /// rpl12*	1,24811127	0,00236549
Procollagen-lysine, 2-oxoglutarate 5-dioxygenase 1	*Plod1*	1,2467632	0,00835563
Ubx domain protein 6	*Ubxn6*	1,24649378	0,00563527
Cyclin-dependent kinase 8	*Cdk8*	1,24625926	0,00667898
Mitochondrial ribosomal protein s26	*Mrps26*	1,24535076	0,00428664
Iscu iron-sulfur cluster scaffold homolog (e. Coli)	*Iscu*	1,24515673	0,00500222
Cap methyltransferase 1	*Cmtr1*	1,2451115	0,00419103
Aconitase 1	*Aco1*	1,24443581	0,00766757
Crystallin, alpha b	*Cryab*	1,24431799	0,00847789
Niemann-pick type c1	*Npc1*	1,24413217	0,00977387
Ftsj homolog 3 (e. Coli)	*Ftsj3*	1,24400339	0,00712895
Decapping enzyme, scavenger	*Dcps*	1,24369073	0,00464106
Copine iii	*Cpne3*	1,2424283	0,00487395
Microsomal glutathione s-transferase 1	*Mgst1*	1,24170235	0,00596145

Threonyl-trna synthetase	*Tars*	1,24163665	0,00347187
Bcdin3 domain containing	*Bcdin3d*	1,24163011	0,00792987
Pyridoxal-dependent decarboxylase domain containing 1	*Pdxdc1*	1,24093899	0,00525021
Phosphoglucomutase 2	*Pgm2*	1,24072675	0,00503287
60s ribosomal protein l12-like /// ribosomal protein l12	*Loc102635048 /// rpl12*	1,24036336	0,00646379
Zinc finger, mynd domain containing 19	*Zmynd19*	1,23994729	0,00372728
Rabaptin, rab gtpase binding effector protein 1	*Rabep1*	1,23978498	0,0051258
F-box and wd-40 domain protein 2	*Fbxw2*	1,2388298	0,00444813
Microtubule-associated protein 4	*Map4*	1,23877369	0,00857601
Prp40 pre-mrna processing factor 40 homolog a (yeast)	*Prpf40a*	1,23834955	0,00773879
Atpase, na+/k+ transporting, alpha 1 polypeptide	*Atp1a1*	1,23784391	0,00439604
Au rna binding protein/enoyl-coenzyme a hydratase	*Auh*	1,23759904	0,00754828
Dynein cytoplasmic 1 intermediate chain 2	*Dync1i2*	1,2375268	0,00413255
Thyroid hormone receptor interactor 11	*Trip11*	1,23600078	0,00937722
Ribosomal protein l14 /// ribosomal protein l14, pseudogene 1	*Rpl14 /// rpl14-ps1*	1,23446651	0,00743297
Acyl-coa thioesterase 10 /// acyl-coa thioesterase 9	*Acot10 /// acot9*	1,23416335	0,00471975
Histidine triad nucleotide binding protein 1	*Hint1*	1,23385037	0,00574229
Ribosomal protein l7-like 1	*Rpl7l1*	1,23298191	0,00479767
Solute carrier family 35, member b2	*Slc35b2*	1,23090419	0,00804123
Nadh dehydrogenase subunit 5	*Nd5*	1,22877484	0,00723211
Transaldolase 1	*Taldo1*	1,22821563	0,00746489
Cdna sequence bc094435 /// ccr4 carbon catabolite repression 4-like (s. Cerevisiae) /// component of oligomeric golgi complex 6 /// sh3-domain grb2-like (endophilin) interacting protein 1	*Bc094435 /// ccrn4l /// cog6 /// sgip1*	1,22500268	0,00609447
Eukaryotic translation initiation factor 3, subunit f	*Eif3f*	1,22450815	0,00466856
Predicted pseudogene 10051 /// ribosomal protein l28	*Gm10051 /// rpl28*	1,22355636	0,00698251
Dnaj (hsp40) homolog, subfamily c, member 10	*Dnajc10*	1,22310492	0,00558055
Anaphase promoting complex subunit 1	*Anapc1*	1,22261719	0,00876681
Cdkn2a interacting protein n-terminal like	*Cdkn2aipnl*	1,22222985	0,00545178
Ribosomal protein l26 pseudogene /// ribosomal protein l26	*Gm15772 /// rpl26*	1,22185956	0,00450913
Spastic paraplegia 21 homolog (human)	*Spg21*	1,2173007	0,00623352

Milk fat globule-egf factor 8 protein	*Mfge8*	1,21725721	0,00714958
Upstream transcription factor 1	*Usf1*	1,21593776	0,00858677
Protein kinase c, iota	*Prkci*	1,21459141	0,00766459
Enabled homolog (drosophila)	*Enah*	1,2143244	0,0082449
Ras homolog enriched in brain	*Rheb*	1,21399176	0,00985702
Clptm1-like	*Clptm1l*	1,21147376	0,00986861
Predicted gene 5621 /// ribosomal protein l10	*Gm5621 /// rpl10*	1,20876146	0,00658493
Mitochondrial ribosomal protein l37	*Mrpl37*	1,20853579	0,00703218
Vacuole membrane protein 1	*Vmp1*	1,19816015	0,00862064

**Gene.title**	**Gene.symbol**	**2^logFC**	**Adj.p.val**
Ribosomal protein l39-like	*Rpl39l*	-400,5481904	1,20e-12
Solute carrier family 14 (urea transporter), member 1	*Slc14a1*	-321,1120696	7,86e-13
Cellular retinoic acid binding protein i	*Crabp1*	-216,5619959	4,42e-12
Growth differentiation factor 10	*Gdf10*	-192,97278	7,59e-12
Collagen, type i, alpha 2	*Col1a2*	-142,1145043	4,53e-11
Dermatopontin	*Dpt*	-133,548562	4,42e-12
Dickkopf homolog 2 (xenopus laevis)	*Dkk2*	-111,9209627	8,21e-12
Keratin 18	*Krt18*	-74,34212582	1,39e-11
Retinoic acid receptor responder (tazarotene induced) 2	*Rarres2*	-72,66093304	4,42e-12
Steroidogenic acute regulatory protein	*Star*	-72,03597655	1,30e-12
Ribosomal protein s4-like	*Rps4l*	-69,51935064	3,13e-11
Integrin, beta-like 1	*Itgbl1*	-69,24566902	7,44e-11
Insulin-like growth factor binding protein 6	*Igfbp6*	-60,7465229	1,79e-11
Sparc-like 1	*Sparcl1*	-56,38569317	3,45e-11
T-cell receptor beta-2 chain c region-like /// t cell receptor beta, joining region /// t cell receptor beta, variable 1	*Loc665506 /// tcrb-j /// trbv1*	-52,37462236	2,39e-10
T-cell receptor beta-2 chain c region-like	*Loc665506*	-47,32007098	3,62e-11
24-dehydrocholesterol reductase	*Dhcr24*	-46,74936692	1,63e-11
Udp-galnac:betaglcnac beta 1,3-galactosaminyltransferase, polypeptide 1	*B3galnt1*	-45,54976344	2,06e-10
Protein kinase c, beta	*Prkcb*	-45,24078391	1,15e-11
Hiv-1 tat interactive protein 2, homolog (human)	*Htatip2*	-40,89403322	2,02e-10
Platelet derived growth factor receptor, alpha polypeptide	*Pdgfra*	-38,84815134	4,23e-10
Cd34 antigen	*Cd34*	-34,46710621	1,02e-09
Regulator of cell cycle	*Rgcc*	-28,06528422	1,26e-09
Cathepsin k	*Ctsk*	-25,48921282	2,50e-10
Mannose receptor, c type 2	*Mrc2*	-24,54813727	9,90e-11
Phospholipase a2, group vii (platelet-activating factor acetylhydrolase, plasma)	*Pla2g7*	-23,88831214	2,06e-10
Dickkopf homolog 3 (xenopus laevis)	*Dkk3*	-23,69451917	7,67e-10
Twist basic helix-loop-helix transcription factor 2	*Twist2*	-21,64706034	1,86e-10
T-box 20	*Tbx20*	-21,01140448	5,67e-09

Enhancer trap locus 4	*Etl4*	-20,29084198	2,82e-10
Fibroblast growth factor 13	*Fgf13*	-19,88860811	2,40e-09
Isopentenyl-diphosphate delta isomerase	*Idi1*	-18,94223506	9,76e-10
Riken cdna 1810011o10 gene	*1810011o10rik*	-18,63362293	5,01e-11
Lymphocyte antigen 6 complex, locus a	*Ly6a*	-18,02874992	1,69e-11
Transforming growth factor, beta induced	*Tgfbi*	-17,68355238	8,76e-11
Phosphodiesterase 8a	*Pde8a*	-15,93958332	2,40e-10
Cadherin 11	*Cdh11*	-15,57801226	7,56e-08
Bone morphogenetic protein 2	*Bmp2*	-14,74115989	1,47e-09
Homeobox a11	*Hoxa11*	-14,7409036	4,50e-07
Immunoglobulin superfamily, member 5	*Igsf5*	-14,49456825	2,18e-07
Fibulin 5	*Fbln5*	-14,48538399	3,26e-09
Receptor tyrosine kinase-like orphan receptor 2	*Ror2*	-14,0048787	3,85e-10
Interleukin 1 receptor-like 1	*Il1rl1*	-13,71960359	2,15e-09
Family with sequence similarity 84, member a	*Fam84a*	-13,68843273	8,00e-09
Family with sequence similarity 198, member b	*Fam198b*	-13,29773682	9,46e-09
Sarcoglycan, epsilon	*Sgce*	-13,08567786	1,75e-09
Inhibitor of dna binding 4	*Id4*	-12,99409284	8,49e-09
Transient receptor potential cation channel, subfamily c, member 6	*Trpc6*	-12,70732337	1,25e-09
Cell division cycle associated 7 like	*Cdca7l*	-12,46830198	1,56e-08
T cell receptor beta, joining region	*Tcrb-j*	-12,21756544	1,54e-09
Epiregulin	*Ereg*	-12,06306865	4,14e-09
Adrenomedullin	*Adm*	-11,91748309	7,01e-09
Ribonuclease p/mrp 25 subunit	*Rpp25*	-11,86044747	4,84e-08
Plexin domain containing 1	*Plxdc1*	-11,79264505	9,24e-11
Homeobox a11, opposite strand	*Hoxa11os*	-11,25743289	1,88e-09
Myosin vb	*Myo5b*	-11,15332895	1,09e-08
R-spondin homolog (xenopus laevis)	*Rspo1*	-10,7644114	2,13e-07
Collagen, type xviii, alpha 1	*Col18a1*	-10,75869485	1,86e-10
Tetraspanin 2	*Tspan2*	-10,74480104	5,92e-07
Pleiomorphic adenoma gene-like 1	*Plagl1*	-10,40040376	5,38e-09
Egl-9 family hypoxia-inducible factor 3	*Egln3*	-10,17815125	4,20e-08
Mediator complex subunit 12-like	*Med12l*	-10,11039423	7,95e-08
S100 calcium binding protein a4	*S100a4*	-9,859360113	2,37e-10
Cd1d1 antigen	*Cd1d1*	-9,7232075	3,58e-09
Betacellulin, epidermal growth factor family member	*Btc*	-9,697580818	9,75e-10
Guanine nucleotide binding protein (g protein), gamma 4	*Gng4*	-9,666230337	5,50e-10
St8 alpha-n-acetyl-neuraminide alpha-2,8-sialyltransferase 1	*St8sia1*	-9,404553957	1,36e-07
Homeobox d9	*Hoxd9*	-9,372529921	8,94e-09
Calpain 6	*Capn6*	-9,306057622	1,14e-08
Homeobox d10	*Hoxd10*	-9,084680342	1,47e-08
Decorin	*Dcn*	-9,038199933	5,99e-08

Spondin 2, extracellular matrix protein	*Spon2*	-9,022340379	2,20e-09
Map/microtubule affinity-regulating kinase 1	*Mark1*	-8,798951824	2,01e-07
Glutathione peroxidase 7	*Gpx7*	-8,569638963	2,14e-08
Procollagen c-endopeptidase enhancer 2	*Pcolce2*	-8,529142513	2,80e-10
Norrie disease (pseudoglioma) (human)	*Ndp*	-8,505119507	2,60e-09
Sry (sex determining region y)-box 2	*Sox2*	-8,342027423	6,43e-10
Pleiotrophin	*Ptn*	-8,260657304	6,92e-10
Nidogen 1	*Nid1*	-8,162801235	2,61e-07
Protein kinase, cgmp-dependent, type ii	*Prkg2*	-8,11185278	3,34e-09
Solute carrier family 8 (sodium/calcium exchanger), member 1	*Slc8a1*	-8,008996125	3,56e-08
Hemochromatosis	*Hfe*	-7,93700752	1,33e-09
Echinoderm microtubule associated protein like 5	*Eml5*	-7,902685392	4,83e-09
Calcium/calmodulin-dependent protein kinase id	*Camk1d*	-7,739057781	3,47e-08
Cysteine and glycine-rich protein 2	*Csrp2*	-7,714788766	8,55e-10
Cd9 antigen	*Cd9*	-7,540784699	4,23e-10
Forkhead box d1	*Foxd1*	-7,286482854	6,44e-09
Spindle and kinetochore associated complex subunit 1	*Ska1*	-7,123906213	3,14e-09
Murine tcr cbeta2 chain cdna. /// t cell receptor beta, joining region	*Tcr-beta chain /// tcrb-j*	-7,089580635	1,05e-08
C-fos induced growth factor	*Figf*	-6,842240673	1,42e-08
Cd244 natural killer cell receptor 2b4	*Cd244*	-6,822514118	2,63e-06
Gata binding protein 2	*Gata2*	-6,808585121	1,78e-09
Neuro-oncological ventral antigen 1	*Nova1*	-6,667628514	1,09e-08
Max dimerization protein 3	*Mxd3*	-6,59240573	2,97e-09
Sema domain, immunoglobulin domain (ig), short basic domain, secreted, (semaphorin) 3a	*Sema3a*	-6,45989867	1,13e-06
Plasmacytoma expressed transcript 2	*Pet2*	-6,311566387	2,01e-07
Secreted frizzled-related protein 2	*Sfrp2*	-6,29429014	4,26e-08
Zw10 interactor	*Zwint*	-6,185478502	4,26e-09
Cytochrome p450, family 51	*Cyp51*	-6,092070341	5,09e-09
T-box18	*Tbx18*	-6,07032006	1,67e-06
3-hydroxy-3-methylglutaryl-coenzyme a synthase 1	*Hmgcs1*	-6,048454109	2,77e-08
Phytanoyl-coa hydroxylase interacting protein-like	*Phyhipl*	-6,019849665	0,00011103
Lysyl oxidase-like 3	*Loxl3*	-5,937390529	1,85e-08
Elovl family member 6, elongation of long chain fatty acids (yeast)	*Elovl6*	-5,881633406	2,44e-09
Rna binding protein gene with multiple splicing	*Rbpms*	-5,862267616	5,39e-07
Synaptogyrin 1	*Syngr1*	-5,836488867	1,28e-09
Mevalonate (diphospho) decarboxylase	*Mvd*	-5,730996606	3,34e-08
Cannabinoid receptor 1 (brain)	*Cnr1*	-5,675768578	1,56e-07
Cysteine-rich protein 1 (intestinal)	*Crip1*	-5,671672276	3,67e-08
Phorbol-12-myristate-13-acetate-induced protein 1	*Pmaip1*	-5,660690013	5,67e-06
Teneurin transmembrane protein 3	*Tenm3*	-5,620193711	1,48e-06
Arylsulfatase i	*Arsi*	-5,571430594	7,01e-09
Hepatocyte growth factor	*Hgf*	-5,483273239	8,26e-06

Fms-like tyrosine kinase 1	*Flt1*	-5,432922266	5,39e-07
Farnesyl diphosphate synthetase	*Fdps*	-5,328946575	5,38e-09
Hydroxyacid oxidase 1, liver	*Hao1*	-5,274984472	1,72e-06
Meiosis 1 associated protein	*M1ap*	-5,270928691	2,70e-08
Myosin 1h	*Myo1h*	-5,226923822	0,00894691
Acetyl-coenzyme a acetyltransferase 2	*Acat2*	-5,20432886	1,36e-09
Collagen, type vi, alpha 3	*Col6a3*	-5,194262	5,09e-09
Sam and sh3 domain containing 1	*Sash1*	-5,155006359	9,52e-09
Stearoyl-coenzyme a desaturase 1	*Scd1*	-5,150018711	2,07e-07
Raftlin family member 2	*Rftn2*	-5,102439239	1,70e-08
Matrilin 2	*Matn2*	-5,022511747	1,98e-07
Sorting nexin 6	*Snx6*	-4,952573507	1,24e-07
Fibroblast growth factor 10	*Fgf10*	-4,937630467	6,84e-06
Methyltransferase like 7a1 /// methyltransferase like 7a2	*Mettl7a1 /// mettl7a2*	-4,900947799	6,11e-08
Cd244 natural killer cell receptor 2b4 /// natural killer cell receptor 2b4-like	*Cd244 /// loc677008*	-4,853000947	8,95e-07
Leprecan-like 2	*Leprel2*	-4,78343056	1,26e-06
Protein tyrosine phosphatase, non-receptor type 13	*Ptpn13*	-4,760209631	3,08e-06
Transforming growth factor alpha	*Tgfa*	-4,753554497	4,49e-06
Keratin 13	*Krt13*	-4,720649949	3,19e-07
Heat shock protein 1b	*Hspa1b*	-4,704320095	1,13e-06
Family with sequence similarity 117, member a	*Fam117a*	-4,695198419	1,27e-06
Runt-related transcription factor 1; translocated to, 1 (cyclin d-related)	*Runx1t1*	-4,66133348	2,12e-06
Chemokine (c-c motif) ligand 8	*Ccl8*	-4,647990398	9,26e-09
Methyltransferase like 7a1	*Mettl7a1*	-4,623290306	3,16e-08
Phosphoglycerate mutase 2	*Pgam2*	-4,580461574	5,53e-08
Slit homolog 2 (drosophila)	*Slit2*	-4,578395443	2,33e-06
Sparc/osteonectin, cwcv and kazal-like domains proteoglycan 3	*Spock3*	-4,568666098	9,02e-06
Centriole, cilia and spindle associated protein	*Ccsap*	-4,513438104	1,92e-08
Chondroitin sulfate n-acetylgalactosaminyltransferase 1	*Csgalnact1*	-4,51310407	1,19e-08
Arylsulfatase a	*Arsa*	-4,510103448	5,67e-09
Pituitary tumor-transforming 1 interacting protein	*Pttg1ip*	-4,464308094	0,00540013
Fxyd domain-containing ion transport regulator 6	*Fxyd6*	-4,459267692	1,25e-07
Calponin 2	*Cnn2*	-4,414845556	5,80e-08
5'-nucleotidase domain containing 2	*Nt5dc2*	-4,386839026	5,27e-08
Carbohydrate sulfotransferase 11 /// phosphatase and actin regulator 1	*Chst11 /// phactr1*	-4,386755669	5,11e-08
Sh3/ankyrin domain gene 3	*Shank3*	-4,385867809	2,18e-07
Star-related lipid transfer (start) domain containing 4	*Stard4*	-4,363734775	3,66e-07
Cell division cycle associated 3	*Cdca3*	-4,361485213	4,05e-09
Farnesyl diphosphate farnesyl transferase 1	*Fdft1*	-4,350435664	4,05e-09
Adenylate cyclase 7	*Adcy7*	-4,336253192	1,95e-08
Sterol regulatory element binding factor 2	*Srebf2*	-4,333436097	1,80e-08

Riken cdna 3110007f17 gene	*3110007f17rik*	-4,331537902	6,68e-06
Solute carrier family 41, member 2	*Slc41a2*	-4,324723006	6,64e-08
Cell growth regulator with ef hand domain 1	*Cgref1*	-4,309324357	6,05e-07
Amphiregulin	*Areg*	-4,276157085	7,03e-08
Microsomal glutathione s-transferase 3	*Mgst3*	-4,228622324	3,18e-08
Serum/glucocorticoid regulated kinase 2	*Sgk2*	-4,220274372	1,98e-07
Dynamin 3, opposite strand /// microrna 214	*Dnm3os /// mir214*	-4,192521835	6,84e-08
Apolipoprotein b receptor	*Apobr*	-4,191620883	2,26e-08
Cd40 antigen	*Cd40*	-4,17755525	1,84e-05
Ww, c2 and coiled-coil domain containing 1	*Wwc1*	-4,134566143	8,34e-08
Wingless-type mmtv integration site family, member 7b	*Wnt7b*	-4,131321194	4,52e-08
Aurora kinase b	*Aurkb*	-4,116137896	8,36e-08
Mannosidase 1, alpha	*Man1a*	-4,079152084	8,34e-08
Methylsterol monoxygenase 1	*Msmo1*	-4,064992921	2,54e-06
3-hydroxy-3-methylglutaryl-coenzyme a reductase	*Hmgcr*	-4,045851339	1,22e-08
Lanosterol synthase	*Lss*	-4,044419964	1,37e-08
Loricrin	*Lor*	-3,954602468	5,55e-08
Homeobox b2	*Hoxb2*	-3,943754751	1,46e-07
Family with sequence similarity 13, member c	*Fam13c*	-3,902772844	6,53e-07
Adducin 2 (beta)	*Add2*	-3,884356481	2,82e-07
Vanin 1	*Vnn1*	-3,878181258	4,34e-07
Procollagen c-endopeptidase enhancer protein	*Pcolce*	-3,873678428	1,33e-07
Squalene epoxidase	*Sqle*	-3,847996701	2,74e-08
Receptor accessory protein 1	*Reep1*	-3,830554014	6,79e-07
Transmembrane channel-like gene family 4	*Tmc4*	-3,816284517	1,26e-06
Biglycan	*Bgn*	-3,811710794	5,17e-07
Solute carrier family 5 (inositol transporters), member 3	*Slc5a3*	-3,790679669	1,71e-06
Twist basic helix-loop-helix transcription factor 1	*Twist1*	-3,755855684	4,87e-08
Thioredoxin reductase 2	*Txnrd2*	-3,74683134	1,13e-08
Proline rich 13	*Prr13*	-3,727814568	2,62e-07
Protocadherin 7	*Pcdh7*	-3,71391568	2,74e-07
Replication factor c (activator 1) 5	*Rfc5*	-3,670455578	1,30e-08
Non-smc condensin ii complex, subunit h2	*Ncaph2*	-3,654569737	6,43e-07
Testis derived transcript	*Tes*	-3,631776178	7,03e-08
T-box 3	*Tbx3*	-3,599845114	3,14e-08
Left right determination factor 1	*Lefty1*	-3,592617851	6,94e-08
Pdz and lim domain 2	*Pdlim2*	-3,588069676	9,90e-08
Homeobox b9	*Hoxb9*	-3,581858712	2,30e-06
Death-associated protein	*Dap*	-3,570524764	1,48e-08
Collapsin response mediator protein 1	*Crmp1*	-3,566910493	8,40e-08
Solute carrier family 2 (facilitated glucose transporter), member 3	*Slc2a3*	-3,565340151	1,60e-07
Non-smc condensin i complex, subunit g	*Ncapg*	-3,543617172	5,37e-05
Predicted gene 13889	*Gm13889*	-3,528915439	4,79e-08

Homeobox d3 /// homeobox d4	*Hoxd3 /// hoxd4*	-3,514374435	5,59e-07
Platelet derived growth factor receptor, beta polypeptide	*Pdgfrb*	-3,505341663	1,12e-07
Riken cdna 2610524h06 gene	*2610524h06rik*	-3,503724375	4,01e-07
Mannosidase 2, alpha 1	*Man2a1*	-3,499207089	2,63e-07
Transmembrane protein 63a	*Tmem63a*	-3,493767193	8,40e-08
Progestin and adipoq receptor family member iv	*Paqr4*	-3,491582862	5,51e-08
Centromere protein a	*Cenpa*	-3,481290375	2,89e-08
Asporin	*Aspn*	-3,470223056	0,00031877
Periostin, osteoblast specific factor	*Postn*	-3,46453447	4,42e-08
Fatty acid desaturase 2	*Fads2*	-3,452926338	2,38e-08
Latrophilin 1	*Lphn1*	-3,437313483	3,56e-06
Riken cdna 2810417h13 gene	*2810417h13rik*	-3,435521867	1,19e-08
Expressed sequence au016916	*Au016916*	-3,435392936	0,00278018
Ribosome binding protein 1	*Rrbp1*	-3,435299336	4,58e-07
Reproductive homeobox 5	*Rhox5*	-3,431389025	1,54e-07
Phosphate regulating endopeptidase homolog, x-linked	*Phex*	-3,427926995	2,41e-05
Nuclear autoantigenic sperm protein (histone-binding)	*Nasp*	-3,419522357	1,68e-06
Cytochrome b-561	*Cyb561*	-3,416983564	4,15e-07
Kh domain containing, rna binding, signal transduction associated 3	*Khdrbs3*	-3,401486129	1,54e-07
Cd248 antigen, endosialin	*Cd248*	-3,388804963	2,37e-06
Rho gtpase activating protein 18	*Arhgap18*	-3,378836089	2,90e-07
Stearoyl-coenzyme a desaturase 2	*Scd2*	-3,377657616	6,28e-05
Ribonucleotide reductase m2	*Rrm2*	-3,376405658	2,34e-05
Homeobox d8	*Hoxd8*	-3,362733757	8,62e-08
Gli-kruppel family member gli2	*Gli2*	-3,361387347	1,90e-06
Deoxycytidine kinase	*Dck*	-3,326908076	2,44e-06
Ornithine decarboxylase, structural 1	*Odc1*	-3,324439797	7,40e-08
Ste20-like kinase	*Slk*	-3,318422757	2,82e-07
Kit ligand	*Kitl*	-3,286651581	0,00024714
Fyn-related kinase	*Frk*	-3,265839746	6,84e-05
Gli pathogenesis-related 1 (glioma)	*Glipr1*	-3,265285681	9,83e-08
Plastin 1 (i-isoform)	*Pls1*	-3,253078701	1,65e-05
Arfgap with fg repeats 2	*Agfg2*	-3,239709732	2,95e-07
Rab2a, member ras oncogene family	*Rab2a*	-3,238063939	0,00656849
Prolactin family 6, subfamily a, member 1	*Prl6a1*	-3,23364391	2,66e-06
Progressive ankylosis	*Ank*	-3,2281287	1,02e-07
Atpase, class v, type 10a	*Atp10a*	-3,227550902	2,53e-05
Spermine oxidase	*Smox*	-3,216273286	1,28e-07
Oaf homolog (drosophila)	*Oaf*	-3,206145685	1,97e-08
Tlr4 interactor with leucine-rich repeats	*Tril*	-3,161396429	1,36e-06
Fatty acid synthase	*Fasn*	-3,156395267	7,89e-08
Coronin, actin binding protein 1c /// predicted gene 17122 /// ---	*Coro1c /// gm17122 /// gm17122*	-3,14072544	0,00670182

Acetyl-coenzyme a acetyltransferase 2 /// acetyl-coenzyme a acetyltransferase 3	*Acat2 /// acat3*	-3,135548713	8,05e-07
Polymerase (dna directed), epsilon 2 (p59 subunit)	*Pole2*	-3,134917725	3,77e-08
Rac gtpase-activating protein 1	*Racgap1*	-3,129503792	1,23e-07
Phosphate cytidylyltransferase 2, ethanolamine	*Pcyt2*	-3,126555111	1,84e-07
Stathmin 1	*Stmn1*	-3,124987636	4,43e-07
Ras related protein 2a	*Rap2a*	-3,114674117	8,59e-08
Chromodomain helicase dna binding protein 3, opposite strand	*Chd3os*	-3,101140984	7,89e-06
Neuropeptide y receptor y5	*Npy5r*	-3,089932686	8,70e-05
Cd109 antigen	*Cd109*	-3,086479029	5,79e-07
Protein kinase c, delta binding protein	*Prkcdbp*	-3,079564051	1,22e-07
Methyltransferase like 7a2	*Mettl7a2*	-3,073074729	6,33e-07
Non-smc condensin i complex, subunit d2	*Ncapd2*	-3,070850835	2,50e-07
Mevalonate kinase	*Mvk*	-3,061124583	3,46e-07
E2f transcription factor 8	*E2f8*	-3,049208984	7,03e-07
Myeloid leukemia factor 1	*Mlf1*	-3,034442929	5,69e-07
Thrombospondin 2	*Thbs2*	-3,03268006	4,73e-08
Sushi-repeat-containing protein	*Srpx*	-3,028818857	1,27e-07
Anoctamin 1, calcium activated chloride channel	*Ano1*	-3,02875015	3,78e-07
Structural maintenance of chromosomes 6	*Smc6*	-3,025576301	1,17e-05
Phd finger protein 19	*Phf19*	-3,025167166	2,50e-07
Acetoacetyl-coa synthetase	*Aacs*	-3,020864675	1,50e-07
Rab32, member ras oncogene family	*Rab32*	-3,011231264	7,35e-07
Tumor necrosis factor receptor superfamily, member 11b (osteoprotegerin)	*Tnfrsf11b*	-3,003292145	5,86e-05
Heterogeneous nuclear ribonucleoprotein a1 pseudogene /// heterogeneous nuclear ribonucleoprotein a1 pseudogene /// predicted gene 5803 /// heterogeneous nuclear ribonucleoprotein a1	*Gm10052 /// gm5643 /// gm5803 /// hnrnpa1*	-3,000488992	4,42e-06
Macro domain containing 2	*Macrod2*	-2,999114042	1,34e-06
Potassium channel, subfamily k, member 5	*Kcnk5*	-2,984737696	2,26e-06
Ligase i, dna, atp-dependent	*Lig1*	-2,977108639	4,73e-08
Ras homolog gene family, member j	*Rhoj*	-2,964281667	4,59e-08
Neuronal guanine nucleotide exchange factor	*Ngef*	-2,946249876	9,85e-08
Dna replication helicase 2 homolog (yeast)	*Dna2*	-2,941056599	9,44e-07
Transketolase	*Tkt*	-2,940636055	4,36e-07
Ca2+-dependent secretion activator	*Cadps*	-2,929154653	1,39e-07
Rho gtpase activating protein 8	*Arhgap8*	-2,926573445	1,25e-07
Rad51 associated protein 1	*Rad51ap1*	-2,925585806	6,68e-07
Angiopoietin 2	*Angpt2*	-2,913893314	5,14e-08
Ellis van creveld gene syndrome	*Evc*	-2,910741293	1,75e-05
Galanin receptor 3 /// glycine c-acetyltransferase (2-amino-3-ketobutyrate-coenzyme a ligase)	*Galr3 /// gcat*	-2,898737311	2,71e-07
Kinesin family member 20a	*Kif20a*	-2,896096584	3,56e-06
Snail family zinc finger 1	*Snai1*	-2,885062529	3,57e-06
Protein phosphatase 6, regulatory subunit 3	*Ppp6r3*	-2,884055054	1,06e-06

Glutathione s-transferase, theta 1	*Gstt1*	-2,883553103	1,38e-05
Centromere protein m	*Cenpm*	-2,86592853	2,49e-06
Dynamin 1	*Dnm1*	-2,864633206	2,97e-06
Fibroblast growth factor receptor 2	*Fgfr2*	-2,863622316	5,28e-06
Antisense igf2r rna	*Airn*	-2,861082936	4,17e-05
Family with sequence similarity 109, member b	*Fam109b*	-2,858508581	0,00034551
Fibrinogen-like protein 2	*Fgl2*	-2,857533301	7,45e-06
Low density lipoprotein receptor	*Ldlr*	-2,855592611	1,25e-07
Family with sequence similarity 111, member a	*Fam111a*	-2,85521501	4,95e-08
Tubulin, alpha 1c pseudogene /// tubulin, alpha 1c	*Gm6682 /// tuba1c*	-2,854493887	4,08e-05
Lymphocyte specific 1	*Lsp1*	-2,854218658	2,08e-06
Heterogeneous nuclear ribonucleoprotein u-like 2	*Hnrnpul2*	-2,847030821	1,14e-06
Hydroxysteroid (17-beta) dehydrogenase 11	*Hsd17b11*	-2,843348612	3,72e-06
Siva1, apoptosis-inducing factor	*Siva1*	-2,832673421	1,03e-07
Shc sh2-domain binding protein 1	*Shcbp1*	-2,824927432	3,81e-07
Udp-glcnac:betagal beta-1,3-n-acetylglucosaminyltransferase 5	*B3gnt5*	-2,824634123	0,00011634
Heat shock protein 1a	*Hspa1a*	-2,823380942	7,86e-06
Phosphofructokinase, platelet	*Pfkp*	-2,796248462	4,02e-08
Procollagen-proline, 2-oxoglutarate 4-dioxygenase (proline 4-hydroxylase), alpha 1 polypeptide	*P4ha1*	-2,790009537	6,43e-06
Expressed sequence aa517023	*Aa517023*	-2,786082952	2,42e-05
Phosphatase and actin regulator 1	*Phactr1*	-2,784081759	1,00e-05
Growth arrest specific 7	*Gas7*	-2,765685206	0,000283
Protein phosphatase 2, regulatory subunit b, delta	*Ppp2r2d*	-2,760783861	0,00509182
Stanniocalcin 1	*Stc1*	-2,753526888	4,33e-06
Netrin 1	*Ntn1*	-2,747047681	3,62e-06
Transcription factor ap-2, alpha	*Tfap2a*	-2,745691031	3,71e-06
Fatty acid binding protein 4, adipocyte	*Fabp4*	-2,739046261	6,17e-07
Cyclin a2	*Ccna2*	-2,733487358	9,08e-06
Predicted gene 5481 /// predicted gene 6109 /// predicted gene 6570 /// predicted pseudogene 7429 /// ribosomal protein l30	*Gm5481 /// gm6109 /// gm6570 /// gm7429 /// rpl30*	-2,728464309	3,58e-07
Netrin g1	*Ntng1*	-2,72561976	0,0009973
Small g protein signaling modulator 3	*Sgsm3*	-2,722120033	4,12e-08
Rad54 like (s. Cerevisiae)	*Rad54l*	-2,718817736	5,17e-07
Dual specificity phosphatase 6	*Dusp6*	-2,717460546	3,52e-07
Transmembrane protein 41b	*Tmem41b*	-2,711591425	2,02e-05
Aldehyde dehydrogenase 1 family, member l1	*Aldh1l1*	-2,698995054	2,17e-07
Glucosaminyl (n-acetyl) transferase 1, core 2	*Gcnt1*	-2,690451571	6,05e-06
Mitochondrial ribosomal protein s25	*Mrps25*	-2,679450332	4,80e-07
Antigen identified by monoclonal antibody ki 67	*Mki67*	-2,679344977	6,89e-08
Lumican	*Lum*	-2,676163136	6,73e-06
Rar-related orphan receptor beta	*Rorb*	-2,665590381	1,00e-05
Collagen, type i, alpha 1	*Col1a1*	-2,663316305	2,87e-06
Sumo1/sentrin specific peptidase 1	*Senp1*	-2,652639712	1,43e-06

Centromere protein h	*Cenph*	-2,652171036	3,48e-06
Cyclin b1	*Ccnb1*	-2,645806352	0,00575329
Mitogen-activated protein kinase kinase kinase 12	*Map3k12*	-2,64345971	0,00803329
Vac14 homolog (s. Cerevisiae)	*Vac14*	-2,634427295	4,06e-06
Cyclin e2	*Ccne2*	-2,631380429	0,00782356
Nad(p) dependent steroid dehydrogenase-like	*Nsdhl*	-2,631078292	9,94e-08
Mad2 mitotic arrest deficient-like 1	*Mad2l1*	-2,628604711	1,07e-07
Leucine rich repeat protein 1	*Lrr1*	-2,626822007	2,16e-06
Polymerase (dna directed), mu	*Polm*	-2,626776992	9,02e-06
Solute carrier family 7 (cationic amino acid transporter, y+ system), member 6	*Slc7a6*	-2,625603596	8,75e-07
Chemokine (c-c motif) ligand 27a /// chemokine (c-c motif) ligand 27b /// predicted gene 13306 /// predicted gene 2506 /// c-c motif chemokine 27-like	*Ccl27a /// ccl27b /// gm13306 /// gm2506 /// loc100861978*	-2,625458963	3,10e-06
Complement component 3a receptor 1	*C3ar1*	-2,614418531	3,66e-06
Ndc1 transmembrane nucleoporin	*Ndc1*	-2,613950047	3,97e-05
Kinesin family member 2c	*Kif2c*	-2,611378214	1,14e-06
Thymic stromal lymphopoietin	*Tslp*	-2,610613393	0,00016436
Lymphocyte antigen 6 complex, locus c1 /// lymphocyte antigen 6 complex, locus c2	*Ly6c1 /// ly6c2*	-2,609343519	3,01e-07
Ecotropic viral integration site 2a	*Evi2a*	-2,609017825	4,18e-07
Ring finger protein 130	*Rnf130*	-2,606446261	8,04e-07
Arfgap with sh3 domain, ankyrin repeat and ph domain1	*Asap1*	-2,581156104	6,34e-08
Tubulin, alpha 3a /// tubulin, alpha 3b	*Tuba3a /// tuba3b*	-2,580281268	5,38e-07
Osteoclast stimulating factor 1	*Ostf1*	-2,580170208	6,24e-08
Serine (or cysteine) peptidase inhibitor, clade e, member 2	*Serpine2*	-2,577522825	3,06e-07
Ral guanine nucleotide dissociation stimulator,-like 1	*Rgl1*	-2,571920714	3,54e-07
Leucine-rich repeat kinase 2	*Lrrk2*	-2,571900438	1,31e-06
Ectopic ossification 1	*Etos1*	-2,566023142	2,14e-06
Transient receptor potential cation channel, subfamily c, member 1	*Trpc1*	-2,564655046	0,00017968
Laminin, alpha 4	*Lama4*	-2,562846246	0,00011373
Kinesin family member 21a	*Kif21a*	-2,561315963	1,24e-06
3-oxoacid coa transferase 1	*Oxct1*	-2,558266995	2,70e-05
Sterol regulatory element binding transcription factor 1	*Srebf1*	-2,553320694	2,56e-07
N-myc downstream regulated gene 4	*Ndrg4*	-2,548770182	2,81e-07
Baculoviral iap repeat-containing 5	*Birc5*	-2,546941449	2,94e-07
Kinesin family member 23	*Kif23*	-2,541118575	0,00013278
Insulin-like growth factor binding protein 4	*Igfbp4*	-2,540947669	4,41e-06
Downstream of stk11	*Dos*	-2,531527598	7,25e-05
Suppression of tumorigenicity 13	*St13*	-2,522216963	2,86e-07
High mobility group at-hook 2	*Hmga2*	-2,516452234	4,02e-06
Tubulin, beta 3 class iii	*Tubb3*	-2,516419794	6,12e-05
Sorcin	*Sri*	-2,515032489	1,81e-07

Cyclin-dependent kinase inhibitor 2d (p19, inhibits cdk4)	*Cdkn2d*	-2,511751811	2,04e-07
Eh-domain containing 3	*Ehd3*	-2,511741717	2,74e-05
Ephrin b1	*Efnb1*	-2,511006158	1,20e-05
Enhancer of zeste homolog 2 (drosophila)	*Ezh2*	-2,506944908	7,88e-07
Hypermethylated in cancer 1	*Hic1*	-2,504112547	2,61e-06
Aldo-keto reductase family 1, member b8	*Akr1b8*	-2,500611284	1,35e-07
Protease-associated domain containing 1	*Pradc1*	-2,498247381	7,30e-07
Galactose-4-epimerase, udp	*Gale*	-2,491645898	2,24e-07
Tripartite motif-containing 24	*Trim24*	-2,488248788	1,75e-06
Ephrin a2	*Efna2*	-2,477206253	8,77e-07
Predicted gene 2808 /// tubby like protein 4	*Gm2808 /// tulp4*	-2,474053296	5,92e-06
Epithelial membrane protein 3	*Emp3*	-2,473820554	4,86e-07
Tropomyosin 1, alpha	*Tpm1*	-2,473728545	1,04e-06
Transcription factor 4	*Tcf4*	-2,472305216	1,01e-05
Angiopoietin-like 4	*Angptl4*	-2,471794109	5,31e-05
Lymphoid enhancer binding factor 1	*Lef1*	-2,470372697	2,04e-05
Serine/threonine kinase 3	*Stk3*	-2,468743684	8,75e-07
Camp responsive element binding protein 3-like 2	*Creb3l2*	-2,468609617	5,16e-07
Mitochondrial ribosomal protein s6	*Mrps6*	-2,467888542	4,56e-07
Histone cluster 1, h2ab /// histone cluster 1, h2ac /// histone cluster 1, h2ad /// histone cluster 1, h2ae /// histone cluster 1, h2ag /// histone cluster 1, h2ah /// histone cluster 1, h2ai /// histone cluster 1, h2an /// histone cluster 1, h2ao /// histone cluster 1, h2ap	*Hist1h2ab /// hist1h2ac /// hist1h2ad /// hist1h2ae /// hist1h2ag /// hist1h2ah /// hist1h2ai /// hist1h2an /// hist1h2ao /// hist1h2ap*	-2,465615291	3,87e-07
Cd80 antigen	*Cd80*	-2,462940112	5,58e-07
Annexin a11 /// predicted gene 2260 /// predicted gene 2274	*Anxa11 /// gm2260 /// gm2274*	-2,46286027	0,00253088
Thymidylate synthase /// thymidylate synthase, pseudogene	*Tyms /// tyms-ps*	-2,459370332	1,24e-07
Sema domain, seven thrombospondin repeats (type 1 and type 1-like), transmembrane domain (tm) and short cytoplasmic domain, (semaphorin) 5a	*Sema5a*	-2,458711958	1,09e-06
Fer (fms/fps related) protein kinase, testis specific 2	*Fert2*	-2,456311225	6,30e-06
Nuclear factor i/b	*Nfib*	-2,455298801	2,71e-05
Hexokinase 1	*Hk1*	-2,454703065	3,10e-05
Sema domain, immunoglobulin domain (ig), transmembrane domain (tm) and short cytoplasmic domain, (semaphorin) 4g	*Sema4g*	-2,451222625	3,66e-06
Fucosyltransferase 8	*Fut8*	-2,450385033	6,53e-07
Maternal embryonic leucine zipper kinase	*Melk*	-2,44927786	1,25e-05
Collagen, type vi, alpha 1	*Col6a1*	-2,447123572	6,29e-06
Inositol (myo)-1(or 4)-monophosphatase 2	*Impa2*	-2,44702782	5,56e-07
Cytoskeleton associated protein 2	*Ckap2*	-2,444768812	1,14e-07

Epithelial membrane protein 1	*Emp1*	-2,443046585	1,86e-06
Pleckstrin homology-like domain, family a, member 1	*Phlda1*	-2,441801371	1,21e-05
Isl1 transcription factor, lim/homeodomain	*Isl1*	-2,440824397	5,32e-06
Syntrophin, basic 2	*Sntb2*	-2,439115095	1,75e-05
Docking protein 1	*Dok1*	-2,438995427	4,63e-07
Occludin	*Ocln*	-2,437583243	2,60e-05
Dna primase, p49 subunit	*Prim1*	-2,430503063	2,11e-07
Dnaj (hsp40) homolog, subfamily c, member 9	*Dnajc9*	-2,427319311	1,64e-07
Spectrin repeat containing, nuclear envelope 2	*Syne2*	-2,42711997	6,42e-06
Zinc finger protein 518b	*Zfp518b*	-2,426478634	1,06e-06
Family with sequence similarity 64, member a	*Fam64a*	-2,424942792	7,33e-07
Inner centromere protein	*Incenp*	-2,424904674	3,83e-07
H2a histone family, member y2 /// h2a histone family, member y3	*H2afy2 /// h2afy3*	-2,420684284	0,00091066
Transmembrane protein 229b	*Tmem229b*	-2,419053051	6,64e-06
Riken cdna 6430548m08 gene	*6430548m08rik*	-2,419020241	1,33e-06
Doublecortin-like kinase 1	*Dclk1*	-2,417432994	5,43e-06
Rna binding motif protein 14	*Rbm14*	-2,41126864	9,36e-07
Citron	*Cit*	-2,407895368	2,04e-06
Mas-related gpr, member f	*Mrgprf*	-2,40575826	0,00064105
Serum/glucocorticoid regulated kinase 3	*Sgk3*	-2,401483499	2,31e-06
Ccr4-not transcription complex, subunit 6	*Cnot6*	-2,394878336	1,91e-05
Acyl-coa synthetase long-chain family member 5	*Acsl5*	-2,391844142	1,52e-07
Cyclin-dependent kinase inhibitor 1a (p21)	*Cdkn1a*	-2,390823901	2,48e-06
Ras association (ralgds/af-6) domain family (n-terminal) member 8	*Rassf8*	-2,38605618	2,23e-07
Cell division cycle 20	*Cdc20*	-2,381237864	1,85e-06
Fibroblast growth factor 18	*Fgf18*	-2,37897454	1,95e-06
Apoptotic peptidase activating factor 1	*Apaf1*	-2,378805927	1,79e-06
Salvador homolog 1 (drosophila)	*Sav1*	-2,376966301	1,07e-06
Myocyte enhancer factor 2c	*Mef2c*	-2,37394173	5,43e-06
Matrix metallopeptidase 16	*Mmp16*	-2,372152633	5,16e-07
H2a histone family, member y2	*H2afy2*	-2,365870471	1,67e-05
Riken cdna 5031439g07 gene	*5031439g07rik*	-2,365585411	6,59e-07
Sh3-binding kinase 1	*Sbk1*	-2,364258044	7,56e-06
Fragile x mental retardation syndrome 1	*Fmr1*	-2,363437936	0,00011227
Potassium channel tetramerisation domain containing 10	*Kctd10*	-2,362615881	6,33e-07
Zinc finger protein 101	*Zfp101*	-2,36205226	9,37e-07
Methylenetetrahydrofolate dehydrogenase (nadp+ dependent), methenyltetrahydrofolate cyclohydrolase, formyltetrahydrofolate synthase	*Mthfd1*	-2,362022078	3,85e-07
Marvel (membrane-associating) domain containing 1	*Marveld1*	-2,360885525	1,26e-06
Transmembrane 7 superfamily member 2	*Tm7sf2*	-2,355191044	5,10e-05
Glutamate receptor, ionotropic, kainate 2 (beta 2)	*Grik2*	-2,351922128	3,49e-06
Synaptonemal complex central element protein 2	*Syce2*	-2,346154542	7,34e-07

Cyclin m2	*Cnnm2*	-2,341462782	1,67e-05
Transcription factor 19	*Tcf19*	-2,340058359	1,78e-07
Trio and f-actin binding protein	*Triobp*	-2,335449495	2,12e-05
Kinesin family member 11	*Kif11*	-2,335229052	2,20e-06
Synaptopodin	*Synpo*	-2,332887616	4,23e-07
Mitogen-activated protein kinase kinase 6	*Map2k6*	-2,332857898	5,43e-06
Mesoderm specific transcript	*Mest*	-2,331973201	0,00012909
Eyes absent 4 homolog (drosophila)	*Eya4*	-2,329264634	3,96e-05
Dead (asp-glu-ala-asp) box polypeptide 39	*Ddx39*	-2,32704926	9,34e-06
Oxidation resistance 1	*Oxr1*	-2,321931981	2,28e-06
Leukotriene a4 hydrolase	*Lta4h*	-2,318622657	3,40e-07
S100 calcium binding protein a10 (calpactin)	*S100a10*	-2,318272439	2,14e-07
Thymidine kinase 1	*Tk1*	-2,31502236	6,02e-07
Odd-skipped related 2	*Osr2*	-2,314851557	1,34e-05
Ectonucleoside triphosphate diphosphohydrolase 7	*Entpd7*	-2,310964658	9,60e-05
Zinc finger homeodomain 4	*Zfhx4*	-2,310076726	4,78e-06
Achalasia, adrenocortical insufficiency, alacrimia	*Aaas*	-2,309615941	2,96e-06
Fused in sarcoma	*Fus*	-2,309278327	0,00010383
Riken cdna 2610507b11 gene	*2610507b11rik*	-2,303596893	2,41e-05
Small proline-rich protein 2a1 /// small proline-rich protein 2a2	*Sprr2a1 /// sprr2a2*	-2,302520361	1,72e-06
Minichromosome maintenance deficient 5, cell division cycle 46 (s. Cerevisiae)	*Mcm5*	-2,299256334	9,21e-07
G protein-coupled receptor 153	*Gpr153*	-2,296611432	2,87e-05
Predicted gene 9840 /// ring-box 1	*Gm9840 /// rbx1*	-2,296377542	1,79e-05
Smoothened homolog (drosophila)	*Smo*	-2,294660089	2,54e-05
Chromobox 5	*Cbx5*	-2,29193546	0,00583869
Nei like 3 (e. Coli)	*Neil3*	-2,289273704	1,48e-06
Ubiquitin carboxy-terminal hydrolase l1	*Uchl1*	-2,28837722	2,29e-05
Anillin, actin binding protein	*Anln*	-2,286254633	4,82e-06
Dihydropyrimidinase	*Dpys*	-2,283210158	1,62e-06
Phosphatase and actin regulator 2	*Phactr2*	-2,282791039	8,71e-05
Gap junction protein, alpha 1	*Gja1*	-2,28215314	0,001153
Dip2 disco-interacting protein 2 homolog b (drosophila)	*Dip2b*	-2,279954773	1,67e-06
Actin related protein 2/3 complex, subunit 4	*Arpc4*	-2,279421823	2,79e-06
Transcription factor 7 like 2, t cell specific, hmg box	*Tcf7l2*	-2,27260327	2,07e-06
Riken cdna 4933413g19 gene /// forkhead box m1 /// phosphatidylethanolamine binding protein 1	*4933413g19rik /// foxm1 /// pebp1*	-2,271684296	3,14e-05
Riken cdna 2310039h08 gene	*2310039h08rik*	-2,270474905	5,61e-06
Nuclear factor of activated t cells, cytoplasmic, calcineurin dependent 4	*Nfatc4*	-2,27026727	1,16e-05
Prolactin regulatory element binding	*Preb*	-2,269088463	7,43e-05
Cell division cycle 7 (s. Cerevisiae)	*Cdc7*	-2,267561846	3,66e-06
Solute carrier family 2 (facilitated glucose transporter), member 5	*Slc2a5*	-2,266264455	5,41e-06
Sulfiredoxin 1 homolog (s. Cerevisiae)	*Srxn1*	-2,265217446	0,00362022

Lamin b1	*Lmnb1*	-2,262020893	4,46e-06
Cyclin f	*Ccnf*	-2,259240824	1,55e-05
Epoxide hydrolase 1, microsomal	*Ephx1*	-2,258446983	8,58e-07
Thymopoietin	*Tmpo*	-2,258070876	0,00101906
G protein-coupled receptor kinase 6	*Grk6*	-2,257140237	2,34e-06
Eh-domain containing 1	*Ehd1*	-2,246675106	4,06e-06
3'-phosphoadenosine 5'-phosphosulfate synthase 2	*Papss2*	-2,24341301	2,51e-06
Kdel (lys-asp-glu-leu) endoplasmic reticulum protein retention receptor 2	*Kdelr2*	-2,238390855	4,11e-05
Src homology three (sh3) and cysteine rich domain	*Stac*	-2,235955268	2,74e-06
Transmembrane protein 144	*Tmem144*	-2,235089917	0,00010505
Phosphatidylinositol 4-kinase type 2 beta	*Pi4k2b*	-2,233941959	0,00069632
Cklf-like marvel transmembrane domain containing 7	*Cmtm7*	-2,233443246	5,70e-06
Predicted gene, 17748 /// peptidyl prolyl isomerase h	*Gm17748 /// ppih*	-2,231690879	1,77e-06
Vps20-associated 1 homolog (s. Cerevisiae)	*Vta1*	-2,225197014	2,03e-06
Poly (adp-ribose) polymerase family, member 12	*Parp12*	-2,223229059	0,00045224
Glutamate receptor, ionotropic, ampa4 (alpha 4)	*Gria4*	-2,22110785	0,00010874
7-dehydrocholesterol reductase	*Dhcr7*	-2,218273637	0,00033278
Predicted gene 7040 /// prolactin family 2, subfamily c, member 2 /// prolactin family 2, subfamily c, member 3 /// prolactin family 2, subfamily c, member 4	*Gm7040 /// prl2c2 /// prl2c3 /// prl2c4*	-2,216600544	3,11e-07
Formin-like 3	*Fmnl3*	-2,215824332	2,52e-06
Jerky	*Jrk*	-2,214719472	5,92e-07
Hop homeobox	*Hopx*	-2,212871533	9,57e-05
Run and fyve domain containing 1	*Rufy1*	-2,206003993	1,48e-06
H2a histone family, member y	*H2afy*	-2,204515657	2,77e-06
Er degradation enhancer, mannosidase alpha-like 1	*Edem1*	-2,204256237	2,83e-06
Solute carrier family 48 (heme transporter), member 1	*Slc48a1*	-2,201255241	2,53e-06
Ribonucleotide reductase m1	*Rrm1*	-2,200789928	0,00042666
Cxadr-like membrane protein	*Clmp*	-2,200490947	0,00156595
Centlein, centrosomal protein	*Cntln*	-2,197752252	3,19e-06
Lim domain only 7	*Lmo7*	-2,197130047	5,11e-07
Cell division cycle associated 5	*Cdca5*	-2,195704143	2,48e-06
Family with sequence similarity 220, member a	*Fam220a*	-2,195700201	5,50e-06
Sh3 domain ysc-like 1	*Sh3yl1*	-2,195636535	1,31e-06
Guanylate cyclase activator 1a (retina)	*Guca1a*	-2,195268635	0,00025026
Neuropeptide y	*Npy*	-2,194647299	4,43e-06
Alpha fetoprotein	*Afp*	-2,193988061	0,00165405
Cyclin b2	*Ccnb2*	-2,193527468	1,06e-06
Sulfatase 2	*Sulf2*	-2,190974349	4,57e-06
Stem-loop binding protein	*Slbp*	-2,189730695	2,12e-05
Choline kinase beta	*Chkb*	-2,188915186	4,50e-07
Abhydrolase domain containing 8	*Abhd8*	-2,18447413	6,85e-06
Regulating synaptic membrane exocytosis 2	*Rims2*	-2,183261531	0,0064031

Dachshund 1 (drosophila)	*Dach1*	-2,182372486	3,82e-06
Evi2a-evi2b readthrough /// ecotropic viral integration site 2b	*Evi2a-evi2b /// evi2b*	-2,176310294	0,00032218
Cyclin g1	*Ccng1*	-2,175935911	0,00504489
Mus musculus cdna, clone:y1g0142j18, strand:unspecified. /// minichromosome maintenance deficient 3 (s. Cerevisiae)	*Ak205147 /// mcm3*	-2,175920283	0,00940433
Interferon-induced protein with tetratricopeptide repeats 2	*Ifit2*	-2,169897722	7,30e-07
Contactin 3	*Cntn3*	-2,169375776	0,00019399
Cap, adenylate cyclase-associated protein 1 (yeast)	*Cap1*	-2,168944005	0,00010445
Peripheral myelin protein 22	*Pmp22*	-2,166773636	5,48e-07
Retinol dehydrogenase 11	*Rdh11*	-2,163735533	7,19e-05
Guanine nucleotide binding protein, alpha 11	*Gna11*	-2,15957789	2,60e-06
Myosin ie	*Myo1e*	-2,158500827	7,56e-06
Pleckstrin homology domain-containing, family a (phosphoinositide binding specific) member 2	*Plekha2*	-2,158032547	0,00014039
Dual specificity phosphatase 9	*Dusp9*	-2,157645952	3,46e-05
Integrin beta 2	*Itgb2*	-2,155548418	1,15e-05
Egf-like domain 7	*Egfl7*	-2,155302121	0,00032461
Epidermal growth factor receptor pathway substrate 8	*Eps8*	-2,146607783	5,82e-06
Histidine triad nucleotide binding protein 3	*Hint3*	-2,14485539	1,16e-05
Keratin 8	*Krt8*	-2,141919839	4,66e-06
Nurim (nuclear envelope membrane protein)	*Nrm*	-2,139979358	2,53e-06
Aurora kinase a	*Aurka*	-2,138493147	9,15e-06
Colony stimulating factor 2 receptor, beta 2, low-affinity (granulocyte-macrophage)	*Csf2rb2*	-2,137836821	9,33e-05
Early growth response 1	*Egr1*	-2,134356818	0,00736325
Chondroitin sulfate n-acetylgalactosaminyltransferase 2	*Csgalnact2*	-2,132736744	8,63e-06
Transformed mouse 3t3 cell double minute 1	*Mdm1*	-2,131972441	0,00050326
Delta-like 1 (drosophila)	*Dll1*	-2,128991362	2,31e-06
Growth associated protein 43	*Gap43*	-2,122597492	0,00127089
Gap junction protein, gamma 1	*Gjc1*	-2,122576237	3,34e-06
Geminin	*Gmnn*	-2,122334839	1,75e-06
Biogenesis of lysosomal organelles complex-1, subunit 1 /// retinol dehydrogenase 5	*Bloc1s1 /// rdh5*	-2,122115487	1,05e-05
Chemokine (c-x-c motif) ligand 12	*Cxcl12*	-2,119443526	1,19e-06
Melanocyte proliferating gene 1	*Myg1*	-2,119093455	4,57e-06
Grp1 (general receptor for phosphoinositides 1)-associated scaffold protein	*Grasp*	-2,116459658	4,47e-05
Dna segment, chr 17, human d6s56e 5	*D17h6s56e-5*	-2,116005551	1,16e-06
Retinitis pigmentosa gtpase regulator interacting protein 1	*Rpgrip1*	-2,115369318	0,00607812
Muscleblind-like 3 (drosophila)	*Mbnl3*	-2,114596938	4,73e-05
Atp citrate lyase	*Acly*	-2,114276344	1,42e-06
Hematological and neurological expressed sequence 1	*Hn1*	-2,114011392	3,56e-06

Solute carrier family 25 (mitochondrial carrier, dicarboxylate transporter), member 10	*Slc25a10*	-2,113771658	1,11e-05
2,3-bisphosphoglycerate mutase	*Bpgm*	-2,111509574	0,00054193
A disintegrin and metallopeptidase domain 8	*Adam8*	-2,10977261	5,27e-06
Collagen, type vi, alpha 2	*Col6a2*	-2,107457805	0,00049942
Peptidase inhibitor 15	*Pi15*	-2,106369162	2,30e-05
Replication protein a3	*Rpa3*	-2,104901925	5,18e-06
Cyclin-dependent kinase 2	*Cdk2*	-2,104876788	6,95e-05
Arginine vasopressin-induced 1	*Avpi1*	-2,102451112	6,73e-06
Growth factor receptor bound protein 14	*Grb14*	-2,100184079	0,00083685
Calmodulin 1 /// calmodulin 2 /// calmodulin 3	*Calm1 /// calm2 /// calm3*	-2,099860603	1,72e-06
Predicted gene 12816 /// predicted gene 5879 /// ribosomal protein l3	*Gm12816 /// gm5879 /// rpl3*	-2,099760641	0,00010356
Interferon induced with helicase c domain 1	*Ifih1*	-2,098914205	0,0004947
Methionine sulfoxide reductase b2	*Msrb2*	-2,096901543	1,38e-05
Sprouty homolog 2 (drosophila)	*Spry2*	-2,096825856	2,88e-05
Map7 domain containing 1	*Map7d1*	-2,095981123	5,04e-06
Aspartate-beta-hydroxylase	*Asph*	-2,095465815	5,30e-05
Vacuolar protein sorting 53 (yeast)	*Vps53*	-2,094888365	0,00024075
Dna segment, chr 10, wayne state university 102, expressed	*D10wsu102e*	-2,094846649	6,71e-06
Myosin, heavy polypeptide 9, non-muscle	*Myh9*	-2,09389625	0,00041294
Kruppel-like factor 5	*Klf5*	-2,093339831	4,01e-05
Plectin	*Plec*	-2,093039251	4,29e-05
Histone deacetylase 5	*Hdac5*	-2,091972284	6,71e-06
Neural precursor cell expressed, developmentally down-regulated gene 1	*Nedd1*	-2,090730397	0,00336632
Ethanolamine kinase 1	*Etnk1*	-2,088374009	0,00299293
Cyclin d1	*Ccnd1*	-2,084936644	6,32e-06
Glycoprotein (transmembrane) nmb	*Gpnmb*	-2,084646733	7,68e-07
G two s phase expressed protein 1	*Gtse1*	-2,082132158	1,36e-05
Dnaj (hsp40) homolog, subfamily a, member 4	*Dnaja4*	-2,081955908	9,02e-06
Osteomodulin	*Omd*	-2,081828464	1,13e-05
Ring finger protein 145	*Rnf145*	-2,080074725	1,87e-06
Transforming, acidic coiled-coil containing protein 3	*Tacc3*	-2,078548223	2,96e-06
Pannexin 1	*Panx1*	-2,078464226	9,89e-07
Small proline-rich protein 2a1 /// small proline-rich protein 2a2 /// small proline-rich protein 2a3	*Sprr2a1 /// sprr2a2 /// sprr2a3*	-2,077171246	8,90e-06
Mitogen-activated protein kinase kinase kinase 7	*Map3k7*	-2,076158187	0,00518708
Intraflagellar transport 27	*Ift27*	-2,075300777	2,00e-05
Tubulin, alpha 1a	*Tuba1a*	-2,073178439	4,42e-06
Isocitrate dehydrogenase 1 (nadp+), soluble	*Idh1*	-2,067174841	0,00016874
Sec24 related gene family, member d (s. Cerevisiae)	*Sec24d*	-2,066482452	2,87e-06
Predicted gene 9817	*Gm9817*	-2,066240946	0,00792638
Protein-tyrosine sulfotransferase 1	*Tpst1*	-2,064974853	6,53e-07
Transferrin receptor	*Tfrc*	-2,061967437	0,00310664

Isochorismatase domain containing 1	*Isoc1*	-2,059528527	6,46e-05
Peroxiredoxin 1 pseudogene /// peroxiredoxin 1	*Gm21399 /// prdx1*	-2,057054633	0,00010632
Nucleoporin 107	*Nup107*	-2,055016337	8,40e-07
Acetyl-coenzyme a acetyltransferase 1	*Acat1*	-2,053054222	4,43e-06
G protein-coupled receptor kinase 5	*Grk5*	-2,051203045	8,87e-05
Expressed sequence aa408251	*Aa408251*	-2,051024175	0,0013272
C-myc binding protein	*Mycbp*	-2,050133537	6,27e-05
Zinc finger protein 68	*Zfp68*	-2,047815262	1,12e-05
Centromere protein k	*Cenpk*	-2,044828064	2,53e-06
Non-smc condensin i complex, subunit h	*Ncaph*	-2,044275959	1,79e-06
Ahnak nucleoprotein (desmoyokin)	*Ahnak*	-2,043444732	1,63e-06
Rad21 homolog (s. Pombe)	*Rad21*	-2,04325496	0,00045025
Membrane protein, palmitoylated 6 (maguk p55 subfamily member 6)	*Mpp6*	-2,041616915	2,70e-06
Family with sequence similarity 19, member a5	*Fam19a5*	-2,040810486	7,02e-07
Stimulated by retinoic acid gene 6	*Stra6*	-2,040244949	8,33e-05
Coiled coil domain containing 88a	*Ccdc88a*	-2,038678171	3,25e-06
Daz associated protein 1	*Dazap1*	-2,038349312	6,03e-06
Splicing factor proline/glutamine rich (polypyrimidine tract binding protein associated)	*Sfpq*	-2,036748317	1,13e-05
Fyve, rhogef and ph domain containing 6	*Fgd6*	-2,035562197	0,00019365
Topoisomerase (dna) ii alpha	*Top2a*	-2,034343961	8,28e-07
Translocator protein	*Tspo*	-2,033718477	2,87e-05
Vacuolar protein sorting 4a (yeast)	*Vps4a*	-2,032170612	2,28e-06
Potassium channel, subfamily k, member 2	*Kcnk2*	-2,03199892	4,10e-06
Collagen, type xii, alpha 1	*Col12a1*	-2,031795172	0,00038123
Brain-specific angiogenesis inhibitor 1-associated protein 2	*Baiap2*	-2,030616616	4,42e-06
Protein phosphatase 1, catalytic subunit, gamma isoform	*Ppp1cc*	-2,027161595	8,24e-06
Excision repair cross-complementing rodent repair deficiency complementation group 6 like	*Ercc6l*	-2,026852662	2,17e-06
Cyclin-dependent kinase 1	*Cdk1*	-2,025929721	8,75e-07
Riken cdna 4930429b21 gene	*4930429b21rik*	-2,025248845	3,02e-05
Spindle and kinetochore associated complex subunit 2	*Ska2*	-2,024605895	2,95e-05
Dual oxidase maturation factor 1	*Duoxa1*	-2,024151185	1,08e-05
S-phase kinase-associated protein 2 (p45)	*Skp2*	-2,024018945	1,87e-05
Ets variant 4	*Etv4*	-2,022072742	1,60e-05
Retinoblastoma-like 1 (p107)	*Rbl1*	-2,021728914	1,95e-05
Transmembrane protein 109	*Tmem109*	-2,021489176	3,02e-06
Essential meiotic endonuclease 1 homolog 1 (s. Pombe)	*Eme1*	-2,020822487	1,51e-05
Homeobox b6	*Hoxb6*	-2,020698434	3,36e-05
Cyclin b1 /// predicted gene 5593	*Ccnb1 /// gm5593*	-2,019734798	3,04e-05
Integrin beta 7	*Itgb7*	-2,018973901	4,23e-06
Hyaluronan mediated motility receptor (rhamm)	*Hmmr*	-2,018682708	1,87e-06

Zinc finger, fyve domain containing 27	*Zfyve27*	-2,016951959	9,71e-06
Homeobox b5	*Hoxb5*	-2,0159237	0,00044908
Zxd family zinc finger c	*Zxdc*	-2,015783866	0,00176434
Schlafen 8	*Slfn8*	-2,015259676	9,12e-06
Calcitonin gene-related peptide-receptor component protein	*Crcp*	-2,014138541	3,37e-06
Glycoprotein ib, beta polypeptide	*Gp1bb*	-2,012554488	0,00018056
Replication factor c (activator 1) 3	*Rfc3*	-2,012509908	3,69e-06
Jun proto-oncogene	*Jun*	-2,011920576	1,36e-05
Atlastin gtpase 3	*Atl3*	-2,008798158	0,00493576
Nucleoporin 85	*Nup85*	-2,007689261	2,17e-05
Nudix (nucleoside diphosphate linked moiety x)-type motif 4	*Nudt4*	-2,007415018	7,44e-06
Proliferating cell nuclear antigen	*Pcna*	-2,005338064	2,67e-06
Tissue inhibitor of metalloproteinase 3	*Timp3*	-2,005033411	2,35e-05
Protein-l-isoaspartate (d-aspartate) o-methyltransferase 1	*Pcmt1*	-2,001967435	4,32e-06
Enolase 2, gamma neuronal	*Eno2*	-2,001347865	1,67e-05
Budding uninhibited by benzimidazoles 1 homolog, beta (s. Cerevisiae)	*Bub1b*	-2,001034588	4,67e-06
Pax interacting (with transcription-activation domain) protein 1	*Paxip1*	-1,99980978	1,17e-05
Ran gtpase activating protein 1	*Rangap1*	-1,999043429	0,00272266
Zinc finger protein 386 (kruppel-like)	*Zfp386*	-1,997624463	2,35e-06
Eomesodermin homolog (xenopus laevis)	*Eomes*	-1,997408603	0,00061623
Dynein, axonemal, heavy chain 11	*Dnah11*	-1,99739658	0,00502083
Ena-vasodilator stimulated phosphoprotein	*Evl*	-1,996816333	0,00018185
Predicted gene 7846 /// lsm5 homolog, u6 small nuclear rna associated (s. Cerevisiae)	*Gm7846 /// lsm5*	-1,993123301	1,17e-05
Coiled-coil domain containing 88c	*Ccdc88c*	-1,991242058	1,31e-05
Forkhead box m1	*Foxm1*	-1,99121071	2,73e-06
Cdc28 protein kinase regulatory subunit 2	*Cks2*	-1,99110451	2,96e-06
Histone deacetylase 2	*Hdac2*	-1,986777395	5,06e-06
Mitochondrial fission regulator 2	*Mtfr2*	-1,986270676	0,00030214
Ubiquitin specific peptidase 2	*Usp2*	-1,985362014	4,74e-05
Spc25, ndc80 kinetochore complex component, homolog (s. Cerevisiae)	*Spc25*	-1,984013203	3,70e-05
Ubiquitin-like, containing phd and ring finger domains, 1	*Uhrf1*	-1,981667385	1,27e-05
Iq motif containing gtpase activating protein 3	*Iqgap3*	-1,978380823	3,37e-06
Stomatin	*Stom*	-1,97832554	6,77e-06
Casein kinase 1, gamma 2	*Csnk1g2*	-1,978029298	8,61e-06
Nucleoporin 37	*Nup37*	-1,975749999	3,39e-06
Dysferlin	*Dysf*	-1,974675545	9,98e-06
High mobility group box 3	*Hmgb3*	-1,973918167	5,47e-05
Cytochrome b5 reductase 3	*Cyb5r3*	-1,972908056	0,00046364
Sec61 alpha 1 subunit (s. Cerevisiae)	*Sec61a1*	-1,972323148	0,00053103
Preli domain containing 1	*Prelid1*	-1,9721468	1,79e-05

Mitogen-activated protein kinase kinase kinase 1	*Map3k1*	-1,971929589	9,35e-05
Zinc finger, cchc domain containing 6	*Zcchc6*	-1,96997726	3,36e-05
Glycerol phosphate dehydrogenase 2, mitochondrial	*Gpd2*	-1,967251732	5,48e-06
Enoyl coenzyme a hydratase domain containing 2	*Echdc2*	-1,966862165	6,95e-05
Solute carrier family 9 (sodium/hydrogen exchanger), member 3 regulator 2	*Slc9a3r2*	-1,9665776	0,00118838
Eyes absent 3 homolog (drosophila)	*Eya3*	-1,966554468	6,05e-06
Heterogeneous nuclear ribonucleoprotein a1	*Hnrnpa1*	-1,96607742	2,68e-05
V-ki-ras2 kirsten rat sarcoma viral oncogene homolog	*Kras*	-1,965527579	7,02e-06
Proteasome (prosome, macropain) activator subunit 4	*Psme4*	-1,962281182	1,84e-05
Basic transcription factor 3	*Btf3*	-1,961325548	0,00024891
Chondroitin polymerizing factor 2	*Chpf2*	-1,954870722	0,00014877
Single-pass membrane protein with aspartate rich tail 1	*Smdt1*	-1,953712143	1,77e-06
Sialophorin, pseudogene	*Spn-ps*	-1,952008198	0,00013394
Transportin 1	*Tnpo1*	-1,94872123	0,00710584
Protein inhibitor of activated stat 3	*Pias3*	-1,947614416	7,13e-06
Protein kinase, amp-activated, gamma 1 non-catalytic subunit	*Prkag1*	-1,947274572	1,14e-06
Adp-ribosylation factor gtpase activating protein 3	*Arfgap3*	-1,946812423	3,29e-06
Eukaryotic translation initiation factor 2 alpha kinase 3	*Eif2ak3*	-1,945575871	0,00030572
Dedicator of cytokinesis 9	*Dock9*	-1,945310346	6,55e-06
Fip1 like 1 (s. Cerevisiae)	*Fip1l1*	-1,943375249	4,10e-05
Lamin b receptor	*Lbr*	-1,942483218	1,96e-05
Diaphanous homolog 3 (drosophila)	*Diap3*	-1,94135826	5,98e-05
Insulin-like growth factor 2 receptor	*Igf2r*	-1,935067132	5,87e-06
C-type lectin domain family 2, member d	*Clec2d*	-1,934506137	8,93e-05
Meiosis-specific nuclear structural protein 1	*Mns1*	-1,93352996	7,03e-06
Polymerase i and transcript release factor	*Ptrf*	-1,933197249	3,58e-06
Vacuolar protein sorting 37b (yeast)	*Vps37b*	-1,933026779	6,57e-05
Potassium channel tetramerisation domain containing 11	*Kctd11*	-1,931088505	4,44e-05
Transcription factor 3	*Tcf3*	-1,930959294	7,15e-05
Male-specific lethal 1 homolog (drosophila)	*Msl1*	-1,929873945	2,57e-05
Ankyrin repeat domain 54	*Ankrd54*	-1,929014248	5,66e-06
Coatomer protein complex, subunit zeta 1	*Copz1*	-1,928455064	1,25e-06
Ubiquitin-like modifier activating enzyme 2	*Uba2*	-1,928016601	0,00222985
Cytochrome c oxidase subunit viic	*Cox7c*	-1,926668631	0,00010696
Mitogen-activated protein kinase kinase 3	*Map2k3*	-1,925872988	3,42e-05
Transcriptional regulator, sin3b (yeast)	*Sin3b*	-1,925116058	1,51e-05
Phosphoribosyl pyrophosphate amidotransferase	*Ppat*	-1,922106327	5,31e-05
Protein regulator of cytokinesis 1	*Prc1*	-1,921406792	1,57e-06
Y box protein 1	*Ybx1*	-1,921296328	1,89e-06
Ubiquitin specific peptidase 15	*Usp15*	-1,919163345	4,30e-06

Ran binding protein 17	*Ranbp17*	-1,917477454	0,00046487
Homeobox b7 /// homeobox b8	*Hoxb7 /// hoxb8*	-1,916966188	0,00900493
Atpase, class vi, type 11a	*Atp11a*	-1,916159521	3,94e-06
Arp1 actin-related protein 1a, centractin alpha	*Actr1a*	-1,915915239	0,00012826
Cyclin-dependent kinase 20	*Cdk20*	-1,91566103	1,87e-06
Udp-n-acetyl-alpha-d-galactosamine:polypeptide n-acetylgalactosaminyltransferase 1	*Galnt1*	-1,915367326	6,42e-06
Polymerase (rna) iii (dna directed) polypeptide k	*Polr3k*	-1,914663948	2,78e-05
Thymoma viral proto-oncogene 1	*Akt1*	-1,914250943	1,21e-05
Choline kinase beta /// chkb-cpt1b readthrough transcript (nmd candidate) /// carnitine palmitoyltransferase 1b, muscle	*Chkb /// chkbcpt1b /// cpt1b*	-1,913549413	5,44e-05
Bromodomain adjacent to zinc finger domain, 1b	*Baz1b*	-1,913456474	0,00798276
Dihydrofolate reductase	*Dhfr*	-1,912707213	4,37e-05
Pq loop repeat containing	*Pqlc3*	-1,911885586	2,13e-05
Riken cdna 1110007c09 gene	*1110007c09rik*	-1,911420575	2,43e-05
Acid phosphatase 1, soluble	*Acp1*	-1,911356307	0,00139588
Riken cdna 2610018g03 gene	*2610018g03rik*	-1,911172353	0,00026609
Breast cancer 1	*Brca1*	-1,910306842	0,00012868
Pdz and lim domain 4	*Pdlim4*	-1,91018287	1,04e-05
Peroxisome proliferative activated receptor, gamma, coactivator 1 alpha	*Ppargc1a*	-1,909816957	0,00055058
Kazal-type serine peptidase inhibitor domain 1	*Kazald1*	-1,909391064	0,00063703
Px domain containing serine/threonine kinase	*Pxk*	-1,907661552	2,29e-05
Parathyroid hormone-like peptide	*Pthlh*	-1,906880418	0,00061938
Solute carrier family 44, member 1	*Slc44a1*	-1,904722339	5,67e-06
Glycine amidinotransferase (l-arginine:glycine amidinotransferase)	*Gatm*	-1,904259656	0,00011112
Calcium channel, voltage-dependent, t type, alpha 1g subunit	*Cacna1g*	-1,903914973	0,00043488
Rna binding protein, fox-1 homolog (c. Elegans) 2	*Rbfox2*	-1,903640687	1,47e-05
Aly/ref export factor	*Alyref*	-1,903546297	8,23e-05
Riken cdna 4932441j04 gene	*4932441j04rik*	-1,903316694	3,01e-05
Centrin 3	*Cetn3*	-1,90298221	2,11e-06
Purine-nucleoside phosphorylase	*Pnp*	-1,901945631	5,43e-05
Minichromosome maintenance deficient 7 (s. Cerevisiae)	*Mcm7*	-1,901504911	1,00e-05
Gipc pdz domain containing family, member 2	*Gipc2*	-1,901042796	5,68e-05
Carbohydrate sulfotransferase 11	*Chst11*	-1,900182575	0,0005717
Interferon regulatory factor 5	*Irf5*	-1,894197948	0,00384007
Heterogeneous nuclear ribonucleoprotein l-like	*Hnrnpll*	-1,891979688	0,00012279
Pan2 polya specific ribonuclease subunit homolog (s. Cerevisiae)	*Pan2*	-1,89195369	0,00039971
Glutamate dehydrogenase 1	*Glud1*	-1,890809323	2,70e-05
Adducin 3 (gamma)	*Add3*	-1,890708779	9,35e-06
Mitochondrial pyruvate carrier 1	*Mpc1*	-1,890706616	4,23e-05
Glutamine fructose-6-phosphate transaminase 1	*Gfpt1*	-1,888837884	0,00041774

Lysm, putative peptidoglycan-binding, domain containing 3	*Lysmd3*	-1,888625398	0,00023995
Ferm domain containing 6	*Frmd6*	-1,887906817	1,64e-06
Calcium/calmodulin-dependent protein kinase ii, delta	*Camk2d*	-1,88715479	0,00014637
Gap junction protein, beta 5	*Gjb5*	-1,887012953	0,00023086
V-ral simian leukemia viral oncogene homolog a (ras related)	*Rala*	-1,884237775	1,69e-05
S100 calcium binding protein a5	*S100a5*	-1,883993623	8,72e-05
Cathepsin e	*Ctse*	-1,882564228	0,00084432
Polypyrimidine tract binding protein 1	*Ptbp1*	-1,881776051	2,92e-06
Flap structure specific endonuclease 1	*Fen1*	-1,881674596	1,08e-05
Ubiquitin-conjugating enzyme e2g 2	*Ube2g2*	-1,881507598	0,00178458
S100 calcium binding protein a3	*S100a3*	-1,88148574	0,00083881
Fanconi anemia, complementation group i	*Fanci*	-1,880305427	7,13e-06
Diacylglycerol kinase, alpha	*Dgka*	-1,880023987	3,79e-05
Bud31 homolog (yeast)	*Bud31*	-1,877479888	0,00115933
Guanylate binding protein 4 /// guanylate-binding protein 8	*Gbp4 /// gbp8*	-1,877346213	0,00567226
Septin 6	*Ix.06*	-1,874788907	0,00026059
Polymerase (dna directed), alpha 2	*Pola2*	-1,872558094	9,72e-06
Riken cdna 4932431p20 gene /// predicted gene, 21596 /// predicted gene 6115 /// high mobility group box 1	*4932431p20rik /// gm21596 /// gm6115 /// hmgb1*	-1,871977255	0,00145522
Transducer of erbb2, 2	*Tob2*	-1,871890199	0,00028651
Rho guanine nucleotide exchange factor (gef) 28	*Arhgef28*	-1,871732448	1,38e-05
Activated leukocyte cell adhesion molecule	*Alcam*	-1,871593655	5,74e-06
Riken cdna 2700089e24 gene	*2700089e24rik*	-1,87136799	2,55e-05
Colony stimulating factor 2 receptor, beta, low-affinity (granulocyte-macrophage)	*Csf2rb*	-1,869224049	0,00017428
Rab21, member ras oncogene family	*Rab21*	-1,86774846	4,25e-05
D site albumin promoter binding protein	*Dbp*	-1,867576703	3,22e-05
Phd finger protein 20-like 1	*Phf20l1*	-1,867264866	8,65e-05
Lamin b2	*Lmnb2*	-1,866625076	0,00012798
Triosephosphate isomerase 1	*Tpi1*	-1,862570482	3,29e-06
Tata box binding protein (tbp)-associated factor, rna polymerase i, a	*Taf1a*	-1,861992049	4,20e-05
Proteasome (prosome, macropain) 26s subunit, atpase 3, interacting protein	*Psmc3ip*	-1,860778262	2,94e-06
Cell division cycle 6	*Cdc6*	-1,857095985	0,00066273
Riken cdna 9130004c02 gene	*9130004c02rik*	-1,85579622	0,00169653
Nudix (nucleoside diphosphate linked moiety x)-type motif 21	*Nudt21*	-1,85483688	2,31e-06
Mus musculus cdna clone mgc:27981 image:3596365, complete cds.	*Bc125002*	-1,854830869	0,00116435
Low density lipoprotein receptor-related protein 8, apolipoprotein e receptor	*Lrp8*	-1,854466736	0,00131005
Ribosomal protein s6 kinase, polypeptide 4	*Rps6ka4*	-1,853495354	0,00640932
Spindle apparatus coiled-coil protein 1	*Spdl1*	-1,852963935	0,00012359

Parathymosin	*Ptms*	-1,852071479	0,00321198
Poly(rc) binding protein 2	*Pcbp2*	-1,849709824	2,61e-06
Homeobox a13	*Hoxa13*	-1,849438534	2,42e-05
Wd repeat domain 1	*Wdr1*	-1,848379429	0,00061278
Recombination signal binding protein for immunoglobulin kappa j region	*Rbpj*	-1,848175266	9,66e-05
Atpase, h+ transporting, lysosomal v0 subunit e2	*Atp6v0e2*	-1,846402537	0,00068927
Hydroxysteroid (17-beta) dehydrogenase 7	*Hsd17b7*	-1,845448055	4,00e-05
Notum pectinacetylesterase homolog (drosophila)	*Notum*	-1,844287821	6,10e-05
Ubiquitin specific peptidase 48	*Usp48*	-1,840774732	4,36e-05
Dna methyltransferase (cytosine-5) 1	*Dnmt1*	-1,840211518	7,01e-06
Kinesin family member 22	*Kif22*	-1,839152079	7,81e-06
Ngfi-a binding protein 2	*Nab2*	-1,838918668	0,00143211
Polymerase (dna directed), delta 1, catalytic subunit	*Pold1*	-1,83794894	0,00019474
Centromere protein n	*Cenpn*	-1,837264041	6,56e-06
Chemokine-like factor	*Cklf*	-1,836816662	0,00025026
Microtubule associated serine/threonine kinase-like	*Mastl*	-1,836800271	0,00188831
Calcium/calmodulin-dependent protein kinase kinase 2, beta	*Camkk2*	-1,833820652	8,20e-05
Integrin beta 3 binding protein (beta3-endonexin)	*Itgb3bp*	-1,833740187	0,00050061
Caspase 3	*Casp3*	-1,832517866	0,00109072
Limb-bud and heart	*Lbh*	-1,832192756	0,00040461
Protein kinase, amp-activated, beta 1 non-catalytic subunit	*Prkab1*	-1,83143569	1,38e-05
Zinc finger and btb domain containing 45	*Zbtb45*	-1,831030863	4,89e-06
Transmembrane protein 184b	*Tmem184b*	-1,830494347	3,29e-06
Atpase, class vi, type 11c	*Atp11c*	-1,829730734	0,00530231
Poly(a) binding protein, cytoplasmic 1	*Pabpc1*	-1,829616692	0,00240098
Dead (asp-glu-ala-asp) box polypeptide 50	*Ddx50*	-1,829015976	3,53e-06
Origin recognition complex, subunit 6	*Orc6*	-1,828686037	4,88e-06
Homeobox a10	*Hoxa10*	-1,828113724	1,30e-05
Contactin associated protein-like 4	*Cntnap4*	-1,827790372	0,0003978
Budding uninhibited by benzimidazoles 1 homolog (s. Cerevisiae)	*Bub1*	-1,827676405	1,27e-05
Adp-ribosylation factor 3	*Arf3*	-1,825145167	7,62e-06
Cysteine-rich hydrophobic domain 1	*Chic1*	-1,824522647	0,00030492
Chondroitin sulfate proteoglycan 4	*Cspg4*	-1,823263472	1,87e-05
Vesicular, overexpressed in cancer, prosurvival protein 1	*Vopp1*	-1,82316902	0,00010309
Minichromosome maintenance deficient 2 mitotin (s. Cerevisiae)	*Mcm2*	-1,820632774	5,12e-06
Tar dna binding protein	*Tardbp*	-1,819872835	0,00077909
Heterogeneous nuclear ribonucleoprotein d-like	*Hnrnpdl*	-1,819828635	6,99e-06
Zinc finger, dhhc domain containing 2	*Zdhhc2*	-1,819810802	0,00538167
Activating transcription factor 7 interacting protein 2	*Atf7ip2*	-1,819797377	0,00218402
Inhibitor of dna binding 2	*Id2*	-1,819597688	0,0002422
Coiled-coil domain containing 25	*Ccdc25*	-1,819281923	0,00537852

Paxillin	*Pxn*	-1,819142037	0,00017533
Homeobox b3	*Hoxb3*	-1,818867647	8,65e-05
Sub1 homolog (s. Cerevisiae)	*Sub1*	-1,818627665	2,45e-06
Cell division cycle 25c	*Cdc25c*	-1,817408336	1,31e-05
Tubby-like protein 3	*Tulp3*	-1,814916316	3,79e-05
Spermatogenesis associated, serine-rich 2	*Spats2*	-1,814571674	7,37e-06
Polymerase (dna directed), epsilon	*Pole*	-1,814168256	8,61e-06
Mis18 kinetochore protein homolog a (s. Pombe)	*Mis18a*	-1,812846371	8,20e-05
Rab1b, member ras oncogene family	*Rab1b*	-1,811507383	0,00015858
Centromere protein o	*Cenpo*	-1,808451577	1,24e-05
Map kinase-interacting serine/threonine kinase 2	*Mknk2*	-1,808148529	1,93e-05
Trafficking protein particle complex 1	*Trappc1*	-1,808126334	1,14e-05
Abhydrolase domain containing 17a	*Abhd17a*	-1,807543603	0,0001326
Rad18 homolog (s. Cerevisiae)	*Rad18*	-1,807419511	0,0016062
Polymerase (dna-directed), delta interacting protein 3	*Poldip3*	-1,807315403	2,71e-05
Nuclear receptor subfamily 1, group d, member 1	*Nr1d1*	-1,807193285	0,00016281
Origin recognition complex, subunit 1	*Orc1*	-1,807106657	0,00223504
Cold inducible rna binding protein	*Cirbp*	-1,807005644	1,73e-05
Nucleolar and spindle associated protein 1	*Nusap1*	-1,806800153	4,27e-05
Nucleoporin 155	*Nup155*	-1,805788032	0,00022323
Chromobox 3 /// predicted pseudogene 5792 /// predicted gene 6901 /// predicted gene 6917	*Cbx3 /// gm5792 /// gm6901 /// gm6917*	-1,805516847	5,31e-05
Dopachrome tautomerase	*Dct*	-1,805058225	0,00039416
Solute carrier family 35, member e1	*Slc35e1*	-1,804947379	2,83e-05
Interleukin 17 receptor a	*Il17ra*	-1,80394716	0,00015992
Sap30-like	*Sap30l*	-1,803778008	8,08e-06
Dek oncogene (dna binding)	*Dek*	-1,801524739	1,39e-05
Angiomotin	*Amot*	-1,801240917	0,00021108
Proteasome (prosome, macropain) subunit, alpha type 7	*Psma7*	-1,800338639	2,79e-05
Serine/arginine-rich splicing factor 4	*Srsf4*	-1,800309823	1,68e-05
Proline-rich coiled-coil 1	*Prrc1*	-1,800275454	5,13e-05
Glycophorin c	*Gypc*	-1,799219693	2,64e-05
Rab29, member ras oncogene family	*Rab29*	-1,798771637	0,00022091
Replication protein a2	*Rpa2*	-1,798714529	0,0009822
High mobility group box 2	*Hmgb2*	-1,798601496	2,21e-05
Cell division cycle 25b	*Cdc25b*	-1,798561259	0,0002077
Matrix metallopeptidase 10	*Mmp10*	-1,798500728	0,00015137
Dis3 mitotic control homolog (s. Cerevisiae)	*Dis3*	-1,797687265	1,03e-05
Proteasome (prosome, macropain) subunit, alpha type 2	*Psma2*	-1,794049036	1,06e-05
Centrosomal protein 41	*Cep41*	-1,791888577	0,00082848
Tubulin tyrosine ligase	*Ttl*	-1,791008353	0,00010992
Polo-like kinase 1	*Plk1*	-1,790519506	4,79e-05
Actinin alpha 4	*Actn4*	-1,790209489	1,11e-05

Absent in melanoma 2	*Aim2*	-1,789429879	0,00033358
Expressed sequence aa409749	*Aa409749*	-1,788974361	0,00170921
Rap guanine nucleotide exchange factor (gef) 3	*Rapgef3*	-1,788970433	0,00339483
Ets variant 1 /// predicted gene 5454	*Etv1 /// gm5454*	-1,786671871	8,20e-06
Hermansky-pudlak syndrome 1 homolog (human)	*Hps1*	-1,785381868	4,43e-06
Thioredoxin-related transmembrane protein 1	*Tmx1*	-1,782842515	5,75e-05
Meningioma expressed antigen 5 (hyaluronidase)	*Mgea5*	-1,782721076	0,00025458
Metastasis suppressor 1	*Mtss1*	-1,782709949	4,29e-05
Ndc80 homolog, kinetochore complex component (s. Cerevisiae)	*Ndc80*	-1,782353933	6,07e-05
Riken cdna c330027c09 gene	*C330027c09rik*	-1,780616107	0,00041153
Laminin b1	*Lamb1*	-1,778728403	2,08e-05
Sperm associated antigen 5	*Spag5*	-1,778564449	0,0002612
Coatomer protein complex, subunit gamma 1	*Copg1*	-1,778280335	3,24e-06
2'-deoxynucleoside 5'-phosphate n-hydrolase 1	*Dnph1*	-1,777040944	6,39e-06
Ribose 5-phosphate isomerase a	*Rpia*	-1,774994871	1,80e-05
Nanos homolog 1 (drosophila)	*Nanos1*	-1,772858293	0,00023954
Transmembrane protein 43	*Tmem43*	-1,772857534	1,75e-05
Cyclin n-terminal domain containing 1	*Cntd1*	-1,772031637	0,0004717
Replication factor c (activator 1) 2	*Rfc2*	-1,770791197	6,09e-06
Paired related homeobox 1	*Prrx1*	-1,770239839	0,00218848
Beta galactoside alpha 2,6 sialyltransferase 1	*St6gal1*	-1,768678561	1,48e-05
Polymerase (dna directed), beta	*Polb*	-1,76696568	1,06e-05
Ptk2 protein tyrosine kinase 2	*Ptk2*	-1,76692431	1,99e-05
Asunder, spermatogenesis regulator	*Asun*	-1,765023502	6,00e-05
Integrator complex subunit 8	*Ints8*	-1,764290859	1,81e-05
Dual specificity phosphatase 11 (rna/rnp complex 1-interacting)	*Dusp11*	-1,761603277	0,00042608
Adenosine deaminase, rna-specific, b1	*Adarb1*	-1,761094836	0,00022539
Trab domain containing	*Trabd*	-1,761001112	6,23e-05
Receptor (calcitonin) activity modifying protein 2	*Ramp2*	-1,759742053	0,00198521
Nudix (nucleoside diphosphate linked moiety x)-type motif 9	*Nudt9*	-1,757598096	1,29e-05
Rab30, member ras oncogene family	*Rab30*	-1,75743558	9,67e-06
Zinc finger and scan domain containing 21	*Zscan21*	-1,756858569	0,00945271
Glutathione s-transferase kappa 1	*Gstk1*	-1,75644151	0,00011588
Family with sequence similarity 76, member a	*Fam76a*	-1,756055251	5,76e-06
Adenylate cyclase activating polypeptide 1	*Adcyap1*	-1,754556738	0,00014109
Chibby homolog 1 (drosophila)	*Cby1*	-1,753749826	4,51e-05
Mediator complex subunit 30	*Med30*	-1,753564668	1,74e-05
Riken cdna 2700029m09 gene	*2700029m09rik*	-1,753488584	6,39e-06
Thioredoxin reductase 1	*Txnrd1*	-1,753204908	0,00751452
O-acyl-adp-ribose deacylase 1	*Oard1*	-1,75253354	0,00014147
Serum/glucocorticoid regulated kinase 1	*Sgk1*	-1,751840763	0,00073724
Membrane protein, palmitoylated	*Mpp1*	-1,751639102	0,00035476
Sphingosine-1-phosphate phosphatase 1	*Sgpp1*	-1,751546509	0,00791706

Sterol-c5-desaturase (fungal erg3, delta-5-desaturase) homolog (s. Cerevisae)	*Sc5d*	-1,751435189	6,16e-05
Mucolipin 2	*Mcoln2*	-1,751223564	0,000537
Serine/arginine-rich splicing factor 3	*Srsf3*	-1,749999633	0,00294339
Pyrroline-5-carboxylate reductase family, member 2	*Pycr2*	-1,749930713	1,03e-05
Ring finger protein 183	*Rnf183*	-1,748477938	8,17e-06
Neuronal pentraxin 1	*Nptx1*	-1,747658949	0,00018589
Serine palmitoyltransferase, small subunit a	*Sptssa*	-1,746029396	4,10e-05
Predicted gene 5553 /// orm1-like 2 (s. Cerevisiae)	*Gm5553 /// ormdl2*	-1,745395429	4,76e-05
Pleckstrin homology domain containing, family g (with rhogef domain) member 2	*Plekhg2*	-1,745368619	0,00080384
Oxysterol binding protein-like 6	*Osbpl6*	-1,745256262	0,00229793
Riken cdna 1700091h14 gene /// predicted gene 8068	*1700091h14rik /// gm8068*	-1,744862738	9,81e-05
Ribosomal protein s17	*Rps17*	-1,742865723	4,79e-05
Ubiquitin-conjugating enzyme e2c	*Ube2c*	-1,742850709	1,85e-05
Citrate synthase	*Cs*	-1,741153956	2,38e-05
Serine palmitoyltransferase, long chain base subunit 1	*Sptlc1*	-1,739935164	4,08e-05
Microspherule protein 1	*Mcrs1*	-1,739263964	4,81e-06
Tubulin, alpha 4a	*Tuba4a*	-1,738609728	5,44e-05
Mis18 binding protein 1	*Mis18bp1*	-1,737812118	2,30e-05
Ect2 oncogene	*Ect2*	-1,737582834	0,00113293
Signal sequence receptor, alpha	*Ssr1*	-1,735080819	0,00170003
Nhp2-like protein 1-like /// nhp2 non-histone chromosome protein 2-like 1 (s. Cerevisiae)	*Loc100862468 /// nhp2l1*	-1,734780025	1,03e-05
Riken cdna 2700094k13 gene	*2700094k13rik*	-1,734774649	3,65e-05
Diras family, gtp-binding ras-like 1	*Diras1*	-1,734563339	0,00022681
Histidine ammonia lyase	*Hal*	-1,733825729	0,00016522
Ubiquitin domain containing 1	*Ubtd1*	-1,733236428	4,60e-05
Kinesin family member c1	*Kifc1*	-1,731027393	0,00146186
Glutamic acid decarboxylase 2	*Gad2*	-1,728391223	7,03e-06
Ataxin 10	*Atxn10*	-1,727536252	0,00118206
Asp (abnormal spindle)-like, microcephaly associated (drosophila)	*Aspm*	-1,727157266	3,74e-05
Letm1 domain containing 1	*Letmd1*	-1,727144665	0,00017213
Calcium/calmodulin-dependent serine protein kinase (maguk family)	*Cask*	-1,725401265	0,00110473
Importin 8	*Ipo8*	-1,724627444	0,00070442
Transmembrane protein 129	*Tmem129*	-1,723730988	7,77e-06
Fatty acid desaturase 1	*Fads1*	-1,72370375	2,62e-05
Family with sequence similarity 83, member g	*Fam83g*	-1,723475838	3,07e-05
Polymerase (dna directed), alpha 1	*Pola1*	-1,722883137	0,00016604
Wnt1 inducible signaling pathway protein 1	*Wisp1*	-1,722250835	9,67e-06
Isy1 splicing factor homolog (s. Cerevisiae)	*Isy1*	-1,722225051	0,00030518
Kdel (lys-asp-glu-leu) endoplasmic reticulum protein retention receptor 3	*Kdelr3*	-1,72183364	5,99e-05
Timeless circadian clock 1	*Timeless*	-1,721218685	1,42e-05

Timeless interacting protein	*Tipin*	-1,720322314	4,88e-05
Tubulin alpha, related sequence 1	*Tuba-rs1*	-1,719147633	0,0003242
H2a histone family, member x	*H2afx*	-1,719136056	2,99e-05
Riken cdna b930041f14 gene	*B930041f14rik*	-1,718808726	5,23e-05
Potassium intermediate/small conductance calcium-activated channel, subfamily n, member 4	*Kcnn4*	-1,718736759	4,18e-05
Gtp binding protein 1	*Gtpbp1*	-1,717806775	2,77e-05
Dynactin 1	*Dctn1*	-1,717421226	0,00013481
Enhancer of yellow 2 homolog (drosophila)	*Eny2*	-1,716723202	7,86e-06
Tho complex 1	*Thoc1*	-1,716078554	0,00512761
Riken cdna 0610007p14 gene	*0610007p14rik*	-1,715485409	5,02e-06
Syntaxin 1a (brain)	*Stx1a*	-1,715317836	0,0022275
Sprouty protein with evh-1 domain 1, related sequence	*Spred1*	-1,714816086	1,36e-05
Checkpoint kinase 2	*Chek2*	-1,714291032	0,00039134
Caveolin 1, caveolae protein	*Cav1*	-1,714268086	6,95e-06
Protein phosphatase 1, catalytic subunit, alpha isoform	*Ppp1ca*	-1,713873928	4,57e-06
Zinc finger protein 467	*Zfp467*	-1,713840079	0,00012863
Msh homeobox 1	*Msx1*	-1,713785	0,0001142
Riken cdna 1110006o24 gene	*1110006o24rik*	-1,713502855	0,00214075
Dopa decarboxylase	*Ddc*	-1,71331246	0,00033641
Acyl-coa synthetase short-chain family member 2	*Acss2*	-1,712706169	0,0003789
Abhydrolase domain containing 5	*Abhd5*	-1,712523155	2,51e-05
Core 1 synthase, glycoprotein-n-acetylgalactosamine 3-beta-galactosyltransferase, 1	*C1galt1*	-1,712448213	0,0001452
Paired related homeobox 2	*Prrx2*	-1,711255169	0,0003276
Ergic and golgi 2	*Ergic2*	-1,710084398	6,99e-05
Activating transcription factor 1 /// predicted pseudogene 1862	*Atf1 /// gm1862*	-1,710040034	2,78e-05
Postmeiotic segregation increased 2 (s. Cerevisiae)	*Pms2*	-1,709618637	6,22e-05
Phenylalkylamine ca2+ antagonist (emopamil) binding protein	*Ebp*	-1,709376282	3,94e-05
Core binding factor beta	*Cbfb*	-1,708798929	0,00030483
Ctf18, chromosome transmission fidelity factor 18	*Chtf18*	-1,70754543	4,96e-05
Cd33 antigen	*Cd33*	-1,707408172	0,00566045
Swi/snf related, matrix associated, actin dependent regulator of chromatin, subfamily d, member 2	*Smarcd2*	-1,70733158	2,75e-05
Tissue inhibitor of metalloproteinase 2	*Timp2*	-1,706572823	0,00699317
Eukaryotic translation initiation factor 2, subunit 1 alpha	*Eif2s1*	-1,706293015	7,20e-05
Myeloid cell leukemia sequence 1	*Mcl1*	-1,706078598	0,00046595
Phosphatidic acid phosphatase type 2a	*Ppap2a*	-1,705881189	0,00015277
High mobility group nucleosomal binding domain 3	*Hmgn3*	-1,703694499	0,0001243
Transmembrane 4 superfamily member 1	*Tm4sf1*	-1,703688804	1,17e-05
Ubiquitin-conjugating enzyme e2j 1	*Ube2j1*	-1,703115137	2,58e-05
Zinc finger, dhhc domain containing 6	*Zdhhc6*	-1,702917658	0,00011103
Lipin 1	*Lpin1*	-1,701983296	3,12e-05

Predicted gene 7040 /// prolactin family 2, subfamily c, member 2 /// prolactin family 2, subfamily c, member 3 /// prolactin family 2, subfamily c, member 4 /// prolactin family 2, subfamily c, member 5	*Gm7040 /// prl2c2 /// prl2c3 /// prl2c4 /// prl2c5*	-1,700037595	0,00039126
Atp/gtp binding protein-like 5	*Agbl5*	-1,699908143	0,00911704
Golgi to er traffic protein 4 homolog (s. Cerevisiae)	*Get4*	-1,699851153	1,34e-05
Methyltransferase like 3	*Mettl3*	-1,699087387	7,41e-05
Lsm3 homolog, u6 small nuclear rna associated (s. Cerevisiae)	*Lsm3*	-1,698725695	2,30e-05
Pituitary tumor-transforming gene 1	*Pttg1*	-1,697733998	1,33e-05
Small nuclear ribonucleoprotein polypeptide a'	*Snrpa1*	-1,697725967	4,95e-05
Budding uninhibited by benzimidazoles 3 homolog (s. Cerevisiae)	*Bub3*	-1,697486179	0,00178975
Translocating chain-associating membrane protein 1	*Tram1*	-1,697037018	7,56e-06
Mitogen-activated protein kinase kinase kinase 5	*Map3k5*	-1,696477984	0,00023079
Hnrnp-associated with lethal yellow	*Raly*	-1,694923967	1,21e-05
Tetratricopeptide repeat domain 8	*Ttc8*	-1,694298633	8,58e-05
Elovl family member 5, elongation of long chain fatty acids (yeast)	*Elovl5*	-1,693502825	0,00018798
Thyroid hormone receptor interactor 13	*Trip13*	-1,693031723	0,00040371
Midnolin	*Midn*	-1,691533773	0,00014358
Predicted gene 13305 /// predicted gene 2002 /// interleukin 11 receptor, alpha chain 1 /// interleukin 11 receptor, alpha chain 2 /// interleukin-11 receptor subunit alpha-2-like	*Gm13305 /// gm2002 /// il11ra1 /// il11ra2 /// loc100861969*	-1,691174111	5,68e-05
Fk506 binding protein 5	*Fkbp5*	-1,691062644	1,60e-05
Ring finger protein 10	*Rnf10*	-1,690706794	0,00011859
Nuclear factor of activated t cells, cytoplasmic, calcineurin dependent 1	*Nfatc1*	-1,690158344	2,56e-05
Ras homolog gene family, member v	*Rhov*	-1,6893374	0,00042846
Unc-119 homolog b (c. Elegans)	*Unc119b*	-1,689074	1,67e-05
Carboxypeptidase n, polypeptide 1	*Cpn1*	-1,688396787	0,00078127
Dnaj (hsp40) homolog, subfamily c, member 12	*Dnajc12*	-1,688393521	0,00033581
Purine-nucleoside phosphorylase /// purine-nucleoside phosphorylase 2	*Pnp /// pnp2*	-1,687950835	0,0016913
Deoxyguanosine kinase	*Dguok*	-1,687856211	0,00016071
Lysosomal-associated protein transmembrane 4b	*Laptm4b*	-1,687605217	6,39e-05
Dna segment, chr 8, wayne state university 26, expressed	*D8wsu26e*	-1,687537786	0,00977387
Adenomatosis polyposis coli down-regulated 1	*Apcdd1*	-1,686979893	9,16e-05
Testicular cell adhesion molecule 1	*Tcam1*	-1,686504801	0,00017207
Golgi-specific brefeldin a-resistance factor 1	*Gbf1*	-1,686404045	9,15e-05
Cox assembly mitochondrial protein 2	*Cmc2*	-1,685747564	1,81e-05
Mkl (megakaryoblastic leukemia)/myocardin-like 1	*Mkl1*	-1,685507676	6,18e-05
3'-phosphoadenosine 5'-phosphosulfate synthase 1	*Papss1*	-1,685242096	5,06e-05
Periplakin	*Ppl*	-1,684490544	0,00396981
Adenosine deaminase, trna-specific 2	*Adat2*	-1,684435699	0,00031185

Solute carrier family 26 (sulfate transporter), member 2	*Slc26a2*	-1,684272372	0,00019419
Karyopherin (importin) alpha 2	*Kpna2*	-1,683977538	6,48e-05
Tgfb-induced factor homeobox 2	*Tgif2*	-1,683843769	1,37e-05
Bromodomain containing 8	*Brd8*	-1,683517912	0,00584484
Kinetochore-localized astrin/spag5 binding	*Knstrn*	-1,683395518	0,00035314
Swi/snf related, matrix associated, actin dependent regulator of chromatin, subfamily a, member 5	*Smarca5*	-1,683238147	7,16e-05
Keratin 75	*Krt75*	-1,682909865	0,00696523
Ttk protein kinase	*Ttk*	-1,681859084	9,83e-05
Ligand of numb-protein x 1	*Lnx1*	-1,68168708	0,00810839
Thymidylate synthase	*Tyms*	-1,681667313	0,00372104
Sec63-like (s. Cerevisiae)	*Sec63*	-1,681625826	3,84e-05
Lim domain containing 2	*Limd2*	-1,681329731	0,00041264
Coiled-coil domain containing 6	*Ccdc6*	-1,681291605	1,20e-05
N-methylpurine-dna glycosylase	*Mpg*	-1,68110162	9,57e-05
Tubulin, beta 5 class i	*Tubb5*	-1,679826992	1,62e-05
Lanc (bacterial lantibiotic synthetase component c)-like 2	*Lancl2*	-1,679817025	5,68e-05
Replication factor c (activator 1) 4	*Rfc4*	-1,679339619	6,23e-05
Zinc finger, cchc domain containing 8	*Zcchc8*	-1,678469032	7,41e-05
Huntingtin	*Htt*	-1,67831506	7,47e-05
Fgfr1 oncogene partner 2	*Fgfr1op2*	-1,678283409	0,00034361
Chromobox 6	*Cbx6*	-1,678007585	0,00010527
Lsm8 homolog, u6 small nuclear rna associated (s. Cerevisiae)	*Lsm8*	-1,67786037	8,27e-06
Adaptor-related protein complex 3, delta 1 subunit	*Ap3d1*	-1,677673087	7,91e-05
Transcription factor dp 1	*Tfdp1*	-1,677556747	0,00029936
Ral guanine nucleotide dissociation stimulator	*Ralgds*	-1,67730998	4,66e-05
Enolase-phosphatase 1	*Enoph1*	-1,677086173	4,81e-05
Tripartite motif-containing 16	*Trim16*	-1,676588178	0,00169653
Dynein light chain tctex-type 1a /// dynein light chain tctex-type 1b /// dynein light chain tctex-type 1c /// dynein light chain tctex-type 1f	*Dynlt1a /// dynlt1b /// dynlt1c /// dynlt1f*	-1,676088542	5,26e-05
Elongation of very long chain fatty acids (fen1/elo2, sur4/elo3, yeast)-like 4	*Elovl4*	-1,675778737	0,0046079
Forkhead box l1	*Foxl1*	-1,675776751	0,00041339
Protein kinase c, alpha	*Prkca*	-1,674570798	5,03e-05
Sr-related ctd-associated factor 11	*Scaf11*	-1,674515852	0,00017977
Anti-silencing function 1b histone chaperone	*Asf1b*	-1,674048268	1,95e-05
Cell division cycle associated 4	*Cdca4*	-1,673559383	0,00062905
Small nuclear ribonucleoprotein 48 (u11/u12)	*Snrnp48*	-1,672265845	0,00285222
Homeobox d13	*Hoxd13*	-1,672186195	0,00014076
Serine (or cysteine) peptidase inhibitor, clade b, member 6a	*Serpinb6a*	-1,671737106	1,07e-05
Star-related lipid transfer (start) domain containing 13	*Stard13*	-1,671361252	4,62e-05
Dead (asp-glu-ala-asp) box polypeptide 21	*Ddx21*	-1,670870613	3,00e-05

Tetraspanin 8	*Tspan8*	-1,670404372	0,00097157
Prp38 pre-mrna processing factor 38 (yeast) domain containing a	*Prpf38a*	-1,670175706	0,00127296
Chloride intracellular channel 1	*Clic1*	-1,669979816	1,25e-05
Hig1 domain family, member 1c /// methyltransferase like 7a1 /// methyltransferase like 7a2 /// mettl7a2-higd1c readthrough transcript /// methyltransferase like 7a3	*Higd1c /// mettl7a1 /// mettl7a2 /// mettl7a2higd1c /// mettl7a3*	-1,669953612	0,00042666
Glutaminyl-peptide cyclotransferase (glutaminyl cyclase)	*Qpct*	-1,669182781	0,00039559
Reticulon 3	*Rtn3*	-1,664186335	7,42e-05
Polymerase (rna) ii (dna directed) polypeptide e	*Polr2e*	-1,663944834	1,39e-05
Adp-ribosylation factor gtpase activating protein 1	*Arfgap1*	-1,663751711	0,00037813
Riken cdna 9030617o03 gene	*9030617o03rik*	-1,663331403	0,00016352
Bicaudal d homolog 2 (drosophila)	*Bicd2*	-1,662340509	1,62e-05
Protein tyrosine phosphatase, receptor type, e	*Ptpre*	-1,662194155	0,00019646
Protein o-fucosyltransferase 2	*Pofut2*	-1,660765743	9,57e-05
Aquaporin 3	*Aqp3*	-1,660612479	0,00057941
Proteasome (prosome, macropain) 26s subunit, non-atpase, 13	*Psmd13*	-1,660427804	2,04e-05
Heterogeneous nuclear ribonucleoprotein l	*Hnrnpl*	-1,660113541	0,00120188
Acylphosphatase 2, muscle type	*Acyp2*	-1,65959485	0,00226221
Pbx/knotted 1 homeobox	*Pknox1*	-1,659489373	0,00020524
Protein tyrosine phosphatase, non-receptor type 1	*Ptpn1*	-1,659483672	0,00010489
Sry (sex determining region y)-box 5	*Sox5*	-1,659397109	0,00108919
Polymerase (dna directed), delta 2, regulatory subunit	*Pold2*	-1,659359104	5,34e-05
Tank-binding kinase 1	*Tbk1*	-1,659339971	0,00017489
Suppressor of defective silencing 3 homolog (s. Cerevisiae)	*Suds3*	-1,659216793	2,93e-05
Small nuclear ribonucleoprotein 40 (u5)	*Snrnp40*	-1,658918828	0,00153556
Nik related kinase	*Nrk*	-1,65876597	0,00102121
Rna binding motif protein, x chromosome	*Rbmx*	-1,657835103	3,77e-05
Endothelin receptor type a	*Ednra*	-1,657683204	0,00890309
Helicase, lymphoid specific	*Hells*	-1,657210026	0,00041328
Aly/ref export factor /// aly/ref export factor 2	*Alyref /// alyref2*	-1,656949257	0,00034511
Tubulin, gamma complex associated protein 3	*Tubgcp3*	-1,65679704	0,00084217
Microrchidia 4	*Morc4*	-1,656537238	0,00018976
Ubiquitin specific peptidase 18	*Usp18*	-1,656082142	1,23e-05
Lysophosphatidylcholine acyltransferase 3	*Lpcat3*	-1,655634714	2,19e-05
Small nuclear ribonucleoprotein d3	*Snrpd3*	-1,655270614	0,00072129
Urate oxidase	*Uox*	-1,655087976	0,00057399
Centrosomal protein 95	*Cep95*	-1,654990456	0,00059321
Wd repeat and hmg-box dna binding protein 1	*Wdhd1*	-1,654386007	0,00014314
Phosphoglycerate mutase 1	*Pgam1*	-1,653641636	2,59e-05
Fanconi anemia, complementation group a	*Fanca*	-1,653615266	7,31e-05
Tubulin, gamma complex associated protein 2	*Tubgcp2*	-1,653319442	1,34e-05

Heterogeneous nuclear ribonucleoprotein a/b	*Hnrnpab*	-1,653005032	0,00028861
Monocyte to macrophage differentiation-associated	*Mmd*	-1,652923821	9,29e-05
Nuclear respiratory factor 1	*Nrf1*	-1,652395379	0,00057573
Eva-1 homolog c (c. Elegans)	*Eva1c*	-1,652358433	0,00045729
Cyclin-dependent kinase 4	*Cdk4*	-1,652276072	1,78e-05
Paxip1 associated glutamate rich protein 1a	*Pagr1a*	-1,652233891	0,00015053
Ankyrin repeat domain 13a	*Ankrd13a*	-1,651073244	2,41e-05
Guanine nucleotide binding protein (g protein), beta 1	*Gnb1*	-1,650616047	0,00453426
Zinc finger protein 36, c3h type-like 1	*Zfp36l1*	-1,650544288	8,22e-05
Zinc finger protein 532	*Zfp532*	-1,649895181	3,31e-05
H2a histone family, member y3	*H2afy3*	-1,649814729	0,0096611
Protein phosphatase 6, regulatory subunit 2	*Ppp6r2*	-1,649636529	0,0033596
Exonuclease 3'-5' domain containing 2	*Exd2*	-1,649245055	0,00015467
Cytohesin 3	*Cyth3*	-1,648627273	2,66e-05
Mau2 chromatid cohesion factor homolog (c. Elegans)	*Mau2*	-1,648492878	4,14e-05
Ganglioside-induced differentiation-associated-protein 10	*Gdap10*	-1,64823117	0,00205521
Centrosomal protein 55	*Cep55*	-1,647872778	0,00418613
Polymerase (dna-directed), delta 3, accessory subunit	*Pold3*	-1,647391205	0,00057497
Achaete-scute complex homolog 1 (drosophila)	*Ascl1*	-1,647057189	0,00218432
Proliferation-associated 2g4	*Pa2g4*	-1,646939669	2,15e-05
Katanin p60 (atpase-containing) subunit a1	*Katna1*	-1,646722468	1,27e-05
Ubx domain protein 8	*Ubxn8*	-1,645349127	0,00059098
Werner syndrome homolog (human)	*Wrn*	-1,644235214	0,00101166
Structural maintenance of chromosomes 3	*Smc3*	-1,643541244	3,66e-05
Riken cdna 2810428i15 gene	*2810428i15rik*	-1,643330446	0,00021979
Serine/arginine-rich splicing factor 7	*Srsf7*	-1,64259895	0,00018262
Ras-related c3 botulinum substrate 1	*Rac1*	-1,642531107	6,29e-05
Mckusick-kaufman syndrome	*Mkks*	-1,642476782	7,06e-05
Haus augmin-like complex, subunit 5	*Haus5*	-1,641895054	0,00029141
Meis homeobox 1	*Meis1*	-1,641266505	0,00016835
Gasdermin c /// gasdermin c2	*Gsdmc /// gsdmc2*	-1,639621626	0,00325642
Tpx2, microtubule-associated protein homolog (xenopus laevis)	*Tpx2*	-1,639435917	2,07e-05
Isoprenylcysteine carboxyl methyltransferase	*Icmt*	-1,639150619	3,76e-05
Histone cluster 1, h4a /// histone cluster 1, h4b /// histone cluster 1, h4c /// histone cluster 1, h4d /// histone cluster 1, h4f /// histone cluster 1, h4h /// histone cluster 1, h4i /// histone cluster 1, h4j /// histone cluster 1, h4k /// histone cluster 1, h4m /// histone cluster 1, h4n /// histone cluster 2, h4 /// histone cluster 4, h4	*Hist1h4a /// hist1h4b /// hist1h4c /// hist1h4d /// hist1h4f /// hist1h4h /// hist1h4i /// hist1h4j /// hist1h4k /// hist1h4m /// hist1h4n /// hist2h4 /// hist4h4*	-1,6391447	0,00890836
Elk3, member of ets oncogene family	*Elk3*	-1,63906684	8,74e-05
Coiled-coil domain containing 77	*Ccdc77*	-1,639020684	0,0030973

Serine/arginine-rich splicing factor 1-like /// serine/arginine-rich splicing factor 1	*Loc102641923 /// srsf1*	-1,638797793	0,00016488
Nucleoporin 133	*Nup133*	-1,638003263	3,02e-05
Transmembrane protein 223	*Tmem223*	-1,637308295	8,33e-05
Neoplastic progression 2	*Npn2*	-1,637161726	0,00035798
Sec14-like 1 (s. Cerevisiae)	*Sec14l1*	-1,635968664	0,00012225
Mediator complex subunit 16	*Med16*	-1,634877433	0,00010803
N-deacetylase/n-sulfotransferase (heparan glucosaminyl) 1	*Ndst1*	-1,63473408	0,00012469
Phosphoglucomutase 1	*Pgm1*	-1,634720478	5,03e-05
Actin related protein 2/3 complex, subunit 3	*Arpc3*	-1,63463819	8,73e-05
Luc7-like 2 (s. Cerevisiae)	*Luc7l2*	-1,634511992	8,95e-05
Ubiquitin-conjugating enzyme e2s	*Ube2s*	-1,634244096	2,01e-05
Kinesin light chain 1	*Klc1*	-1,633658061	8,78e-05
Breast cancer metastasis-suppressor 1	*Brms1*	-1,633620831	0,00020804
Carnitine palmitoyltransferase 1a, liver	*Cpt1a*	-1,633351673	0,00151788
Otu domain containing 7b	*Otud7b*	-1,63257531	0,00069535
Phosphatidylinositol 4-kinase type 2 alpha	*Pi4k2a*	-1,632196121	5,40e-05
Unconventional snare in the er 1 homolog (s. Cerevisiae)	*Use1*	-1,631986015	2,46e-05
Keratin 10	*Krt10*	-1,631813828	0,00048046
Multiple endocrine neoplasia 1	*Men1*	-1,631566986	0,00019715
Homeobox c13	*Hoxc13*	-1,63141069	0,0001344
Nipa-like domain containing 2	*Nipal2*	-1,631408106	0,00180599
Ring finger protein 157	*Rnf157*	-1,630168648	0,00077506
Serine/arginine repetitive matrix 1	*Srrm1*	-1,630131832	0,00017289
Microtubule-associated protein, rp/eb family, member 2	*Mapre2*	-1,629998388	4,58e-05
Tubulin, beta 4b class ivb	*Tubb4b*	-1,629847387	1,67e-05
Poc5 centriolar protein homolog (chlamydomonas)	*Poc5*	-1,629346761	0,00019946
Endoplasmic reticulum-golgi intermediate compartment (ergic) 1	*Ergic1*	-1,628733694	0,00980852
Regulator of g-protein signaling 2	*Rgs2*	-1,628469025	0,00016471
Pou domain, class 6, transcription factor 1	*Pou6f1*	-1,628195303	5,94e-05
Zinc finger protein 521	*Zfp521*	-1,627985037	0,00156806
Dead (asp-glu-ala-asp) box polypeptide 49	*Ddx49*	-1,627622654	0,00061751
F-box protein 31	*Fbxo31*	-1,627242621	0,0005919
Arp10 actin-related protein 10	*Actr10*	-1,627076478	7,33e-05
Ww domain binding protein 1	*Wbp1*	-1,62541383	5,86e-05
Nuclear factor i/x	*Nfix*	-1,624960899	0,00012094
Interleukin 34	*Il34*	-1,624634926	0,00011131
Start domain containing 8	*Stard8*	-1,624230699	0,00013131
Iroquois related homeobox 2 (drosophila)	*Irx2*	-1,623864507	6,39e-05
Wd repeat domain 90	*Wdr90*	-1,623005237	0,00011441
Interferon zeta	*Ifnz*	-1,622271946	0,00411228
Pecanex homolog (drosophila)	*Pcnx*	-1,621869933	0,00038509

Asparagine-linked glycosylation 12 (alpha-1,6-mannosyltransferase)	*Alg12*	-1,621001795	0,00020201
Killer cell lectin-like receptor, subfamily a, member 18	*Klra18*	-1,620800227	0,00150987
Scribbled homolog (drosophila)	*Scrib*	-1,619845575	0,00106286
Riken cdna 2610528a11 gene	*2610528a11rik*	-1,619556179	0,0026901
Testis expressed gene 261	*Tex261*	-1,619188434	0,00010445
Glial cell line derived neurotrophic factor family receptor alpha 2	*Gfra2*	-1,618476381	0,00168846
Dep domain containing 7	*Depdc7*	-1,618432535	3,53e-05
Erythrocyte protein band 4.1-like 1	*Epb4.1l1*	-1,618406796	0,00034231
Squamous cell carcinoma antigen recognized by t cells 3	*Sart3*	-1,618324258	0,00032569
Adaptor-related protein complex ap-1, mu subunit 1	*Ap1m1*	-1,618116662	3,65e-05
Lectin, mannose-binding, 1	*Lman1*	-1,618042277	0,00039141
Fc receptor, igg, low affinity iib	*Fcgr2b*	-1,61709026	0,00637139
Family with sequence similarity 120, member a	*Fam120a*	-1,61612649	0,00304786
Insulin-like growth factor 2 mrna binding protein 1	*Igf2bp1*	-1,61498605	0,00052978
Family with sequence similarity 114, member a1	*Fam114a1*	-1,614149585	3,46e-05
Solute carrier family 4 (anion exchanger), member 8	*Slc4a8*	-1,614003547	0,00056935
Sar1 gene homolog a (s. Cerevisiae)	*Sar1a*	-1,613452738	2,07e-05
Riken cdna 0610037l13 gene	*0610037l13rik*	-1,61254309	0,00015788
Actin related protein 2/3 complex, subunit 1b /// predicted pseudogene 5637	*Arpc1b /// gm5637*	-1,612272048	2,41e-05
Protein tyrosine phosphatase 4a3	*Ptp4a3*	-1,612214364	0,00122812
Proteasome (prosome, macropain) 26s subunit, non-atpase, 3	*Psmd3*	-1,611628503	0,0001136
Diacylglycerol lipase, beta	*Daglb*	-1,610331475	0,000232
Neural precursor cell expressed, developmentally down-regulated gene 4-like	*Nedd4l*	-1,609792412	1,47e-05
Lamin a	*Lmna*	-1,609643191	0,00013496
Mitogen-activated protein kinase 8 interacting protein 2	*Mapk8ip2*	-1,609593429	0,00248499
Sodium channel, voltage-gated, type i, beta	*Scn1b*	-1,608612039	0,0010645
Proteolipid protein 2	*Plp2*	-1,608344577	1,29e-05
Spectrin beta, non-erythrocytic 1	*Sptbn1*	-1,608243073	0,00091147
Wd repeat and socs box-containing 2	*Wsb2*	-1,607041663	0,00012781
Mus musculus adult male lung cdna, riken full-length enriched library, clone:1200016e24 product:unclassifiable, full insert sequence. /// predicted gene, 20186	*Bc057675 /// gm20186*	-1,605153283	0,00256616
Nuclear transcription factor-y beta	*Nfyb*	-1,603935479	4,06e-05
Guanine nucleotide binding protein (g protein), beta 3	*Gnb3*	-1,603095813	0,00887623
Dual specificity phosphatase 22	*Dusp22*	-1,601345528	0,00024206
Neuronal pentraxin chromo domain /// neuronal pentraxin receptor	*Npcd /// nptxr*	-1,600364404	0,0011829
Protein o-fucosyltransferase 1	*Pofut1*	-1,600229621	0,000548
Eukaryotic translation initiation factor 4e binding protein 2	*Eif4ebp2*	-1,600139312	0,00010178

Scavenger receptor class b, member 1	*Scarb1*	-1,600050517	0,00165304
Galactose mutarotase	*Galm*	-1,59968776	0,00092911
Adp-ribosylation factor-like 4d	*Arl4d*	-1,599624828	0,000181
Peroxiredoxin 2	*Prdx2*	-1,599101343	3,97e-05
Splicing factor 1	*Sf1*	-1,598776973	0,00141935
Gene trap rosa 26, philippe soriano	*Gt(rosa)26sor*	-1,598680222	0,00044944
F-box and wd-40 domain protein 4	*Fbxw4*	-1,598592803	0,00043658
Centrobin, centrosomal brca2 interacting protein	*Cntrob*	-1,598574539	1,53e-05
Oligosaccharyltransferase 4 homolog (s. Cerevisiae)	*Ost4*	-1,598423794	0,00022445
Guanine nucleotide binding protein (g protein), beta 2	*Gnb2*	-1,598320573	8,73e-05
Kelch-like 5	*Klhl5*	-1,598164391	5,04e-05
Vacuolar protein sorting 36 (yeast)	*Vps36*	-1,597554282	4,77e-05
Eukaryotic translation initiation factor 4 gamma, 3	*Eif4g3*	-1,597183925	0,00017598
Membrane bound o-acyltransferase domain containing 1	*Mboat1*	-1,597152192	3,09e-05
Tripartite motif-containing 28	*Trim28*	-1,596672044	0,00025035
Ring finger protein 121	*Rnf121*	-1,596563309	0,00051401
Maternally expressed 3	*Meg3*	-1,595827121	6,10e-05
Protein arginine n-methyltransferase 3	*Prmt3*	-1,59543219	0,00310336
Transcription factor 20	*Tcf20*	-1,59537041	0,00019791
Mms19 (met18 s. Cerevisiae)	*Mms19*	-1,595115699	8,73e-05
Fibroblast growth factor 14	*Fgf14*	-1,594490582	0,00059583
Zinc finger, fyve domain containing 21	*Zfyve21*	-1,593845724	0,0014548
Interferon-induced protein with tetratricopeptide repeats 3	*Ifit3*	-1,593613436	0,00019479
Dna segment, chr 10, johns hopkins university 81 expressed	*D10jhu81e*	-1,593001753	3,97e-05
Scm-like with four mbt domains 2	*Sfmbt2*	-1,592714299	0,00291176
Zinc finger protein 821	*Zfp821*	-1,59190824	0,00035951
Pyruvate dehydrogenase kinase, isoenzyme 3	*Pdk3*	-1,591112317	0,00028347
Cyclin i	*Ccni*	-1,591026277	0,00024481
Zinc finger, cchc domain containing 17	*Zcchc17*	-1,590635187	4,61e-05
Btb (poz) domain containing 2	*Btbd2*	-1,58990562	0,00043995
Integrin beta 1 binding protein 1	*Itgb1bp1*	-1,589671327	8,47e-05
Purkinje cell protein 2 (l7)	*Pcp2*	-1,58944056	0,0039072
Excision repair cross-complementing rodent repair deficiency, complementation group 1	*Ercc1*	-1,589302999	8,92e-05
Sterol o-acyltransferase 1	*Soat1*	-1,588732977	9,06e-05
Polymerase (dna-directed), epsilon 4 (p12 subunit)	*Pole4*	-1,588093958	0,00162615
Pyridoxine 5'-phosphate oxidase	*Pnpo*	-1,58647305	0,00019677
High mobility group at-hook 1 /// high mobility group at-hook i, related sequence 1	*Hmga1 /// hmga1-rs1*	-1,586224935	0,0070555
Hippocalcin-like 1	*Hpcal1*	-1,585760765	4,77e-05
Low density lipoprotein receptor-related protein 1	*Lrp1*	-1,585049273	0,00017572
Transmembrane protein 263	*Tmem263*	-1,585026976	0,00783544

Solute carrier family 6 (neurotransmitter transporter, taurine), member 6	*Slc6a6*	-1,583921598	0,0036124
Ebna1 binding protein 2	*Ebna1bp2*	-1,583616124	6,33e-05
Caveolin 2	*Cav2*	-1,58257517	0,00010158
Tubulin, alpha 1c pseudogene /// tubulin, alpha 1a	*Gm6682 /// tuba1a*	-1,582415634	0,00525869
Cysteine and histidine rich 1	*Cyhr1*	-1,582398756	0,00105896
Microtubule-associated protein 1b	*Map1b*	-1,582190332	0,00933079
Plasmacytoma variant translocation 1	*Pvt1*	-1,581878987	3,46e-05
Platelet-activating factor acetylhydrolase, isoform 1b, subunit 1	*Pafah1b1*	-1,581279793	6,42e-05
Histone deacetylase 7	*Hdac7*	-1,58105694	0,00047849
Cd164 antigen	*Cd164*	-1,580814209	0,00023079
Sh3 domain binding glutamic acid-rich protein-like 3	*Sh3bgrl3*	-1,580635245	8,09e-05
Lim and senescent cell antigen-like domains 1	*Lims1*	-1,58053923	0,0004978
Cd1d2 antigen	*Cd1d2*	-1,580384146	0,00027449
Nuf2, ndc80 kinetochore complex component, homolog (s. Cerevisiae)	*Nuf2*	-1,580298752	0,00029896
Proline rich 14	*Prr14*	-1,579327804	0,00013448
Apelin	*Apln*	-1,579080808	0,00092309
Proteasome (prosome, macropain) 26s subunit, non-atpase, 9	*Psmd9*	-1,578837374	0,0035346
Discs, large (drosophila) homolog-associated protein 5	*Dlgap5*	-1,576843685	0,00068416
G protein-coupled receptor kinase-interactor 2	*Git2*	-1,576280218	0,00061846
Erythrocyte protein band 4.2	*Epb4.2*	-1,576039081	0,00474866
Proline-rich nuclear receptor coactivator 2	*Pnrc2*	-1,575458478	0,00111184
Predicted gene 2382 /// 5, 10-methenyltetrahydrofolate synthetase	*Gm2382 /// mthfs*	-1,575420309	0,00017598
Rab10, member ras oncogene family	*Rab10*	-1,57530242	0,00446001
Ankyrin repeat domain 32	*Ankrd32*	-1,574806199	0,00576831
Riken cdna 4930427a07 gene	*4930427a07rik*	-1,573676054	0,00032611
Prostaglandin e receptor 2 (subtype ep2)	*Ptger2*	-1,573658501	0,00019676
Cyclin-dependent kinase 16	*Cdk16*	-1,573467787	0,00018189
Cd22 antigen	*Cd22*	-1,573294487	0,00509182
Haus augmin-like complex, subunit 3	*Haus3*	-1,572803586	4,51e-05
Crm, cramped-like (drosophila)	*Cramp1l*	-1,572701275	0,00522226
Proteasome (prosome, macropain) inhibitor subunit 1	*Psmf1*	-1,572623405	4,81e-05
Solute carrier family 19 (thiamine transporter), member 2	*Slc19a2*	-1,572161611	0,00013272
Taf12 rna polymerase ii, tata box binding protein (tbp)-associated factor	*Taf12*	-1,572038813	4,60e-05
Grainyhead-like 2 (drosophila)	*Grhl2*	-1,571930829	0,00381789
Na+/k+ transporting atpase interacting 1	*Nkain1*	-1,571520893	5,80e-05
Utrophin	*Utrn*	-1,571191554	1,85e-05
Leprecan-like 4	*Leprel4*	-1,570878405	0,00015449
Sphingosine kinase 2	*Sphk2*	-1,570728652	0,00555912
F-box and wd-40 domain protein 8	*Fbxw8*	-1,56995351	0,00093258

Nol1/nop2/sun domain family, member 5	*Nsun5*	-1,569951603	0,0004341
Nucleoporin 43	*Nup43*	-1,56990958	6,79e-05
Fk506 binding protein 11	*Fkbp11*	-1,568732852	0,0010764
Replication factor c (activator 1) 1	*Rfc1*	-1,568415504	0,00012549
Anaphase-promoting complex subunit 5	*Anapc5*	-1,568336706	0,00012286
Thyrotroph embryonic factor	*Tef*	-1,567785631	0,00023477
Msh homeobox 3	*Msx3*	-1,567533657	0,00014853
Glutathione s-transferase, mu 1	*Gstm1*	-1,567162846	0,00225548
Serine/arginine repetitive matrix 3	*Srrm3*	-1,567051458	7,70e-05
Hyaluronan synthase 3	*Has3*	-1,566943124	0,0082979
Sarcoglycan, delta (dystrophin-associated glycoprotein)	*Sgcd*	-1,566782688	0,00036064
Tax1 (human t cell leukemia virus type i) binding protein 1	*Tax1bp1*	-1,566488899	0,00067977
Paired box 9	*Pax9*	-1,56636794	0,00040671
Zinc finger (ccch type), rna binding motif and serine/arginine rich 1	*Zrsr1*	-1,566151199	4,57e-05
Bcl2-associated agonist of cell death	*Bad*	-1,565967169	0,00020398
Mms22-like, dna repair protein	*Mms22l*	-1,565456766	7,54e-05
Dna fragmentation factor, alpha subunit	*Dffa*	-1,565324814	0,0010984
Immediate early response 3	*Ier3*	-1,565289352	6,46e-05
Suppressor of cytokine signaling 5	*Socs5*	-1,5651187	0,00073086
Elastin microfibril interfacer 1	*Emilin1*	-1,564988468	0,00322324
Receptor (calcitonin) activity modifying protein 3	*Ramp3*	-1,564444186	0,00063935
Caspase 6	*Casp6*	-1,563824536	0,00016331
Histone cluster 1, h3a /// histone cluster 1, h3b /// histone cluster 1, h3c /// histone cluster 1, h3d /// histone cluster 1, h3e /// histone cluster 1, h3f /// histone cluster 1, h3g /// histone cluster 1, h3h /// histone cluster 1, h3i /// histone cluster 2, h3b /// histone cluster 2, h3c1 /// histone cluster 2, h3c2	*Hist1h3a /// hist1h3b /// hist1h3c /// hist1h3d /// hist1h3e /// hist1h3f /// hist1h3g /// hist1h3h /// hist1h3i /// hist2h3b /// hist2h3c1 /// hist2h3c2*	-1,562940231	0,00120379
Lsm14 homolog b (scd6, s. Cerevisiae)	*Lsm14b*	-1,562645288	8,49e-05
Nima (never in mitosis gene a)-related expressed kinase 8	*Nek8*	-1,562475948	0,00015885
Gtpase activating protein (sh3 domain) binding protein 2	*G3bp2*	-1,561967527	0,00080865
Interferon-related developmental regulator 1	*Ifrd1*	-1,561936872	0,00014812
Uev and lactate/malate dehyrogenase domains	*Uevld*	-1,561580349	0,00048463
M-phase phosphoprotein 9	*Mphosph9*	-1,561460816	0,00022515
Yip1 interacting factor homolog a (s. Cerevisiae)	*Yif1a*	-1,561428707	0,00028029
Ewing tumor-associated antigen 1	*Etaa1*	-1,561368771	0,00052462
Atpase, h+ transporting, lysosomal v1 subunit d	*Atp6v1d*	-1,560991087	0,00011227
Leucine-rich repeats and wd repeat domain containing 1	*Lrwd1*	-1,560111421	0,00070692
Adrenergic receptor, alpha 1a	*Adra1a*	-1,560110862	0,00053596
Interleukin 6 receptor, alpha	*Il6ra*	-1,559974628	0,00154818
Aprataxin and pnkp like factor	*Aplf*	-1,559932194	0,00086591

Receptor accessory protein 3	*Reep3*	-1,559546606	0,00021656
Extracellular matrix protein 1	*Ecm1*	-1,559369614	0,00069399
Sine oculis-related homeobox 5	*Six5*	-1,55882083	4,03e-05
Enhancer of rudimentary homolog (drosophila) /// enhancer of rudimentary homolog	*Erh /// loc101056061*	-1,558183491	8,61e-05
Hect domain and ankyrin repeat containing, e3 ubiquitin protein ligase 1	*Hace1*	-1,558022893	0,00281414
Denticleless homolog (drosophila)	*Dtl*	-1,557892625	0,00839634
Lymphocyte antigen 6 complex, locus f	*Ly6f*	-1,557419359	0,00274639
Ring finger protein 144b	*Rnf144b*	-1,557099136	4,11e-05
Aspartoacylase	*Aspa*	-1,556855672	0,00018424
Retinol binding protein 1, cellular	*Rbp1*	-1,556560831	0,00261031
Inner membrane protein, mitochondrial	*Immt*	-1,556129983	0,00044084
Suppressor of variegation 3-9 homolog 1 (drosophila)	*Suv39h1*	-1,555988568	1,95e-05
Two pore channel 1	*Tpcn1*	-1,555614111	0,00011767
Mitochondrial ribosomal protein l49	*Mrpl49*	-1,554858432	0,00033027
Tbc1 domain family, member 15	*Tbc1d15*	-1,554715308	0,00270542
Pdz binding kinase	*Pbk*	-1,554216019	0,00016263
Centromere protein e	*Cenpe*	-1,554092409	0,00024481
Polo-like kinase 4	*Plk4*	-1,553734045	0,00049185
Mitogen-activated protein kinase kinase kinase 6	*Map3k6*	-1,55330713	0,00037387
Podoplanin	*Pdpn*	-1,553299918	9,38e-05
Anaphase prompoting complex c subunit 15	*Anapc15*	-1,552842325	9,69e-05
Predicted gene, 21596 /// predicted gene 6115 /// high mobility group box 1 /// high mobility group protein b1-like	*Gm21596 /// gm6115 /// hmgb1 /// loc102635075*	-1,552374366	0,00266535
Translocase of outer mitochondrial membrane 7 homolog (yeast)	*Tomm7*	-1,552151058	0,00459564
Riken cdna 1700020i14 gene	*1700020i14rik*	-1,551463864	0,00060816
Mitochondrial ribosomal protein l42	*Mrpl42*	-1,551116079	7,45e-05
Thioredoxin 1	*Txn1*	-1,550867791	0,0082284
Exonuclease 1	*Exo1*	-1,550663262	0,00022947
Sorting nexin 3	*Snx3*	-1,550660996	4,95e-05
Notch 1	*Notch1*	-1,549505976	0,00037494
Sestrin 1	*Sesn1*	-1,549454973	2,89e-05
Homer homolog 3 (drosophila)	*Homer3*	-1,548911663	0,00016445
Microfibrillar associated protein 5	*Mfap5*	-1,548669585	0,00044081
F-box protein 4	*Fbxo4*	-1,547855906	0,00013646
Immunoglobulin kappa chain variable 28 (v28) /// immunoglobulin kappa variable 6-14 /// immunoglobulin kappa variable 6-23 /// immunoglobulin kappa variable 8-19	*Igk-v28 /// igkv6-14 /// igkv6-23 /// igkv8-19*	-1,547826392	0,00196601
Solute carrier family 7 (cationic amino acid transporter, y+ system), member 4	*Slc7a4*	-1,547578476	0,00204632
Fibulin 1	*Fbln1*	-1,547133402	0,00512051
Angiogenic factor with g patch and fha domains 1	*Aggf1*	-1,547121331	5,65e-05
Calcium binding protein 39-like	*Cab39l*	-1,547021164	0,00163887

Block of proliferation 1	*Bop1*	-1,546415991	9,42e-05
Riken cdna 2700097o09 gene	*2700097o09rik*	-1,546247253	0,00021947
Apurinic/apyrimidinic endonuclease 2	*Apex2*	-1,545735085	0,00019463
Rna binding motif protein 12	*Rbm12*	-1,54555444	0,00556474
Collagen, type xvi, alpha 1	*Col16a1*	-1,544953512	0,00044841
E2f-associated phosphoprotein	*Eapp*	-1,543790162	0,00102306
Ectodysplasin-a	*Eda*	-1,543686479	0,00339865
Ankyrin repeat and socs box-containing 13	*Asb13*	-1,543639646	0,00375221
Paired box 5	*Pax5*	-1,542960891	0,00757378
Microphthalmia-associated transcription factor	*Mitf*	-1,542367705	0,00017637
Acyl-coa thioesterase 7	*Acot7*	-1,542089937	5,02e-05
Ninein	*Nin*	-1,540898709	0,00236411
Leucine rich repeat containing 8 family, member c	*Lrrc8c*	-1,54083846	0,00061003
Heparan sulfate 6-o-sulfotransferase 3	*Hs6st3*	-1,540787309	0,0003211
Nicalin homolog (zebrafish)	*Ncln*	-1,540660786	0,00395828
Tyro3 protein tyrosine kinase 3	*Tyro3*	-1,540360178	0,00089989
Riken cdna b230354o11 gene	*B230354o11rik*	-1,539934281	0,00931584
Guanylyl cyclase domain containing 1	*Gucd1*	-1,539908767	0,00014472
Cytidine monophosphate (ump-cmp) kinase 1	*Cmpk1*	-1,539813907	0,00228754
Adp-ribosylation factor-like 1	*Arl1*	-1,539722422	7,80e-05
Syntaxin 4a (placental)	*Stx4a*	-1,539648903	0,00113639
Rest corepressor 2	*Rcor2*	-1,539599608	0,00070975
Thyroglobulin	*Tg*	-1,538840834	0,00152681
Hydroxymethylbilane synthase	*Hmbs*	-1,538385887	0,00014695
Heart and neural crest derivatives expressed transcript 1	*Hand1*	-1,538213955	0,00021663
T lymphoma oncogene	*Tlm*	-1,538097396	0,00755071
Furry homolog-like (drosophila)	*Fryl*	-1,537599728	7,11e-05
Erythroid differentiation regulatory factor 1	*Edrf1*	-1,537536865	0,0003064
Metallothionein 1	*Mt1*	-1,537429929	0,00088929
Histone deacetylase 1	*Hdac1*	-1,536711308	0,00025582
Kruppel-like factor 6	*Klf6*	-1,536671115	0,00015253
Adaptor-related protein complex 3, mu 2 subunit	*Ap3m2*	-1,536536841	0,00931477
Spermatogenesis associated 13	*Spata13*	-1,536464682	0,00054023
Phospholipase c, beta 1	*Plcb1*	-1,535403463	0,00782274
Polymerase (dna directed), eta (rad 30 related)	*Polh*	-1,535315913	0,00647837
Abhydrolase domain containing 16a	*Abhd16a*	-1,534816431	7,54e-05
Period circadian clock 3	*Per3*	-1,534766782	0,00155474
Ribosomal rna processing 7 homolog a (s. Cerevisiae)	*Rrp7a*	-1,534756575	0,00034275
Clathrin, light polypeptide (lcb)	*Cltb*	-1,534122735	0,00035753
Coatomer protein complex, subunit epsilon	*Cope*	-1,533560924	0,00078478
Rna binding motif protein, x-linked 2	*Rbmx2*	-1,533096862	0,00088124
Sap domain containing ribonucleoprotein	*Sarnp*	-1,532999069	0,0001546
Predicted gene 14277 /// small nuclear ribonucleoprotein d1	*Gm14277 /// snrpd1*	-1,532841869	4,45e-05

Phosphatidylinositol transfer protein, cytoplasmic 1	*Pitpnc1*	-1,532048465	0,00057682
Uracil dna glycosylase	*Ung*	-1,531722944	9,76e-05
Lysine (k)-specific demethylase 2b	*Kdm2b*	-1,531710779	0,0001145
Retinal degeneration 3	*Rd3*	-1,530848652	0,00949943
Non-smc condensin ii complex, subunit g2	*Ncapg2*	-1,530482309	0,00145826
Polymerase (dna directed), kappa	*Polk*	-1,529635272	0,0073413
Leucine rich repeat (in flii) interacting protein 1	*Lrrfip1*	-1,528851144	0,00012094
Annexin a7	*Anxa7*	-1,527663744	0,0001272
Sap30 binding protein	*Sap30bp*	-1,527020018	0,00047971
Progesterone receptor	*Pgr*	-1,526741361	0,00138918
Major facilitator superfamily domain containing 1	*Mfsd1*	-1,52611449	0,00490583
Clarin 3	*Clrn3*	-1,525776212	0,00882747
C-abl oncogene 1, non-receptor tyrosine kinase	*Abl1*	-1,525470379	0,00010637
Cytochrome c, somatic	*Cycs*	-1,525130518	6,92e-05
Tetraspanin 33	*Tspan33*	-1,525095896	0,0072781
Transformed mouse 3t3 cell double minute 2	*Mdm2*	-1,525090014	0,00548875
--- /// predicted gene, 17344	*Gm17344 /// gm17344*	-1,525078061	0,00911704
Pitrilysin metallepetidase 1	*Pitrm1*	-1,525006481	0,00177412
Rotatin	*Rttn*	-1,524768628	0,00018863
Matrix metallopeptidase 24	*Mmp24*	-1,524627551	0,0091575
Nuclear factor i/a	*Nfia*	-1,524207859	0,00129647
Vesicle transport through interaction with t-snares 1b	*Vti1b*	-1,523810953	0,00013401
Chimerin 2	*Chn2*	-1,523689663	0,00135188
Sema domain, transmembrane domain (tm), and cytoplasmic domain, (semaphorin) 6d	*Sema6d*	-1,523646123	7,90e-05
Adenylate cyclase 6	*Adcy6*	-1,523170517	0,00019948
Proteasome (prosome, macropain) subunit, beta type 2	*Psmb2*	-1,52272963	4,91e-05
Unc-51 like kinase 2	*Ulk2*	-1,521668715	0,00555912
Lim homeobox protein 4	*Lhx4*	-1,521376866	0,00185221
Cd276 antigen	*Cd276*	-1,521298762	0,00705355
Diazepam binding inhibitor	*Dbi*	-1,521151648	8,20e-05
Proteasome (prosome, macropain) subunit, alpha type 3	*Psma3*	-1,520608245	3,79e-05
Tetraspanin 9	*Tspan9*	-1,520536008	0,00024448
St3 beta-galactoside alpha-2,3-sialyltransferase 4	*St3gal4*	-1,520403926	0,00353081
Vacuole membrane protein 1	*Vmp1*	-1,519496972	0,0010473
Sorting and assembly machinery component 50 homolog (s. Cerevisiae)	*Samm50*	-1,519442893	0,00010865
Inhibin alpha	*Inha*	-1,519423243	0,00012004
Dnaj (hsp40) homolog, subfamily c, member 14	*Dnajc14*	-1,519365295	0,00013496
Twisted gastrulation homolog 1 (drosophila)	*Twsg1*	-1,519152607	0,00513084
Cdc42 effector protein (rho gtpase binding) 4	*Cdc42ep4*	-1,519113018	0,00035418
Solute carrier family 8 (sodium/lithium/calcium exchanger), member b1	*Slc8b1*	-1,518630691	0,00034231

Ww domain containing e3 ubiquitin protein ligase 1	*Wwp1*	-1,518330139	0,0023667
Mitochondrial ribosomal protein l13	*Mrpl13*	-1,51804331	4,01e-05
Late endosomal/lysosomal adaptor, mapk and mtor activator 1	*Lamtor1*	-1,517973803	0,0004874
Exportin 1, crm1 homolog (yeast)	*Xpo1*	-1,517772742	0,00094959
Syntaxin 3	*Stx3*	-1,517587922	0,00016356
Chromatin assembly factor 1, subunit b (p60)	*Chaf1b*	-1,516720192	0,00022715
Steap family member 3	*Steap3*	-1,516642387	0,00032146
Aconitase 2, mitochondrial	*Aco2*	-1,516626103	8,87e-05
Zyg-11 family member a, cell cycle regulator	*Zyg11a*	-1,516086413	0,00043004
Pre b cell leukemia homeobox 3	*Pbx3*	-1,516021313	6,14e-05
Brick1, scar/wave actin-nucleating complex subunit	*Brk1*	-1,515765623	0,00020646
Pci domain containing 2	*Pcid2*	-1,515590503	0,00070305
Histone cell cycle regulation defective homolog a (s. Cerevisiae)	*Hira*	-1,515345543	0,00087241
Cystatin e/m	*Cst6*	-1,515316081	0,00294544
Centromere protein f	*Cenpf*	-1,515021173	0,00036248
Tao kinase 3	*Taok3*	-1,514904145	0,00567216
Creb/atf bzip transcription factor	*Crebzf*	-1,514351974	0,00350994
Cobw domain containing 1	*Cbwd1*	-1,514296328	0,00013389
Family with sequence similarity 131, member b	*Fam131b*	-1,514223054	0,00049261
Fanconi anemia, complementation group m	*Fancm*	-1,513772111	0,00269641
Ribonuclease, rnase a family 4	*Rnase4*	-1,513693939	0,00689224
Histone deacetylase 6	*Hdac6*	-1,513241147	0,0001192
Pyrimidinergic receptor p2y, g-protein coupled, 6	*P2ry6*	-1,512845242	0,00017289
Coronin, actin binding protein 1c	*Coro1c*	-1,512677404	6,98e-05
Mesoderm induction early response 1, family member 2	*Mier2*	-1,512637167	0,00142643
Synaptophysin-like 2	*Sypl2*	-1,512597825	0,0003207
Spectrin repeat containing, nuclear envelope 1	*Syne1*	-1,51251212	0,0001813
Fibroblast growth factor receptor 1	*Fgfr1*	-1,512154469	0,00294866
Tar (hiv) rna binding protein 2	*Tarbp2*	-1,511889504	0,00014964
Atp-binding cassette, sub-family a (abc1), member 3	*Abca3*	-1,511586999	0,0004036
U2 small nuclear rna auxiliary factor 1-like 4	*U2af1l4*	-1,511560495	0,00035814
Was/wasl interacting protein family, member 1	*Wipf1*	-1,511103289	0,00518371
Lectin, galactose binding, soluble 8	*Lgals8*	-1,511070602	0,00019724
Predicted gene 12693 /// rwd domain containing 1	*Gm12693 /// rwdd1*	-1,510957314	0,00776905
Riken cdna 1110059e24 gene	*1110059e24rik*	-1,510247282	4,96e-05
Integrin alpha 2b	*Itga2b*	-1,509803539	0,00612549
Solute carrier family 39 (metal ion transporter), member 8	*Slc39a8*	-1,508953084	0,000191
Zinc finger, dhhc domain containing 8	*Zdhhc8*	-1,508573991	0,00044153
Zinc finger ccch-type containing 18	*Zc3h18*	-1,507895502	0,00032569
Ctd (carboxy-terminal domain, rna polymerase ii, polypeptide a) small phosphatase 2	*Ctdsp2*	-1,507660585	0,00015708
Predicted gene 12606	*Gm12606*	-1,507571109	0,0005102
Histocompatibility 13	*H13*	-1,5073241	0,00016363

Williams beuren syndrome chromosome region 22	*Wbscr22*	-1,507117187	0,00023733
Dead (asp-glu-ala-asp) box polypeptide 27	*Ddx27*	-1,506752937	0,00041622
Fig4 homolog (s. Cerevisiae)	*Fig4*	-1,506172111	0,00029147
Ras association (ralgds/af-6) domain family member 3	*Rassf3*	-1,505801163	0,00025651
Farnesyltransferase, caax box, alpha	*Fnta*	-1,504690889	0,00018562
Dihydropyrimidinase-like 3	*Dpysl3*	-1,504517832	6,29e-05
Predicted gene 10193 /// zinc finger protein 706	*Gm10193 /// zfp706*	-1,504275662	0,00087353
Serine/arginine-rich splicing factor 10	*Srsf10*	-1,504221979	0,00294384
Rab13, member ras oncogene family	*Rab13*	-1,503709351	0,00113681
Serine/arginine-rich splicing factor 9	*Srsf9*	-1,503074335	9,39e-05
Cdk2 (cyclin-dependent kinase 2)-associated protein 1	*Cdk2ap1*	-1,50276874	7,44e-05
Phospholipase c, gamma 1	*Plcg1*	-1,502203627	0,00147251
Activating transcription factor 7 interacting protein	*Atf7ip*	-1,502062913	0,00154957
Calcium homeostasis endoplasmic reticulum protein	*Cherp*	-1,501818075	0,00012715
Submaxillary gland androgen regulated protein 2	*Smr2*	-1,501460088	0,00055519
Nuclear pore membrane protein 121	*Pom121*	-1,501312411	0,00131045
Anaphase promoting complex subunit 4	*Anapc4*	-1,499905456	8,44e-05
Kinesin family member 13a	*Kif13a*	-1,499608119	0,00011249
Meiotic recombination 11 homolog a (s. Cerevisiae)	*Mre11a*	-1,499367902	0,00147251
Nuclear factor of activated t cells, cytoplasmic, calcineurin dependent 2 interacting protein	*Nfatc2ip*	-1,499341494	0,00086826
Zinc finger, cchc domain containing 14	*Zcchc14*	-1,499209715	0,00042846
Early endosome antigen 1	*Eea1*	-1,49902016	0,00029008
Peroxisomal biogenesis factor 5	*Pex5*	-1,498861657	0,00054464
Eukaryotic translation initiation factor 2d	*Eif2d*	-1,498790631	0,00181705
Solute carrier family 35, member c2	*Slc35c2*	-1,498748754	0,00441103
Cdc16 cell division cycle 16	*Cdc16*	-1,498668475	0,00042063
Predicted gene 15453 /// rna binding motif protein 3	*Gm15453 /// rbm3*	-1,498505013	0,00042666
Olfactory receptor 70	*Olfr70*	-1,497648885	0,00459564
Topoisomerase (dna) ii binding protein 1	*Topbp1*	-1,497351156	0,00099161
Zinc finger protein 787	*Zfp787*	-1,497290477	0,00565027
Predicted gene, 21596 /// predicted gene 6115 /// high mobility group box 1	*Gm21596 /// gm6115 /// hmgb1*	-1,497145705	0,00019463
Membrane associated guanylate kinase, ww and pdz domain containing 1	*Magi1*	-1,495957217	0,00012184
Chemokine (c-c motif) ligand 17	*Ccl17*	-1,495850682	0,00951951
Dysbindin (dystrobrevin binding protein 1) domain containing 1	*Dbndd1*	-1,495689894	0,00882607
Nudix (nucleoside diphosphate linked moiety x)-type motif 1	*Nudt1*	-1,495050362	0,00014345
Predicted gene 6750 /// predicted pseudogene 7931 /// predicted gene 9525 /// high mobility group nucleosomal binding domain 2	*Gm6750 /// gm7931 /// gm9525 /// hmgn2*	-1,494937969	0,00013076
Polyhomeotic-like 2 (drosophila)	*Phc2*	-1,494099794	0,00050436
Ribonuclease/angiogenin inhibitor 1	*Rnh1*	-1,494081613	8,93e-05
Splicing factor 3b, subunit 5	*Sf3b5*	-1,494069725	0,00015262

Riken cdna a030005k14 gene	*A030005k14rik*	-1,493889754	0,00624337
Nucleolar and coiled-body phosphoprotein 1	*Nolc1*	-1,493218158	0,00030904
Solute carrier family 11 (proton-coupled divalent metal ion transporters), member 2	*Slc11a2*	-1,493097958	0,00756131
Eh-domain containing 2	*Ehd2*	-1,493020341	0,00014964
Methyl-cpg binding domain protein 1	*Mbd1*	-1,492754175	0,0052392
Ras related protein 1b	*Rap1b*	-1,492620173	0,00785121
Sex comb on midleg homolog 1	*Scmh1*	-1,492033538	0,00048874
Rab7, member ras oncogene family	*Rab7*	-1,491866256	0,00373823
Mitogen-activated protein kinase kinase 3, opposite strand	*Map2k3os*	-1,491663482	0,00012279
Zinc finger, an1-type domain 2a	*Zfand2a*	-1,491638783	0,00025116
Transducin (beta)-like 2	*Tbl2*	-1,490842604	0,00012225
Signal recognition particle 54a /// signal recognition particle 54b /// signal recognition particle 54c	*Srp54a /// srp54b /// srp54c*	-1,490112867	0,00033201
Glycolipid transfer protein	*Gltp*	-1,489811096	0,00435351
Microtubule associated serine/threonine kinase 2	*Mast2*	-1,488270457	0,00049594
Cdc28 protein kinase 1b	*Cks1b*	-1,488254743	0,00044944
Small proline-rich protein 2f	*Sprr2f*	-1,488180013	0,00025643
Plasminogen activator, urokinase	*Plau*	-1,488068288	0,00018662
Deoxynucleotidyltransferase, terminal, interacting protein 1	*Dnttip1*	-1,487476686	9,75e-05
General transcription factor iiic, polypeptide 2, beta	*Gtf3c2*	-1,487346162	0,00072934
Moloney leukemia virus 10-like 1	*Mov10l1*	-1,486790173	0,00897275
Ubiquitin protein ligase e3c	*Ube3c*	-1,486725489	0,00011394
Ankyrin repeat domain 44	*Ankrd44*	-1,486317945	0,00057467
Claudin 10	*Cldn10*	-1,486243501	0,00795613
Epithelial stromal interaction 1 (breast)	*Epsti1*	-1,48620382	0,00893139
Inhibitor of dna binding 3	*Id3*	-1,485727663	9,02e-05
Proteasome maturation protein	*Pomp*	-1,485425259	0,0001192
Udp-glucose dehydrogenase	*Ugdh*	-1,484914997	0,00075064
Dna segment, chr 4, erato doi 117, expressed	*D4ertd117e*	-1,484710723	0,00600364
Microtubule-associated protein, rp/eb family, member 1	*Mapre1*	-1,484635443	0,00159374
Intermediate filament family orphan 2	*Iffo2*	-1,484633272	0,00279617
Bms1 homolog, ribosome assembly protein (yeast)	*Bms1*	-1,483625661	0,00095873
Sorting nexin 29	*Snx29*	-1,483620785	0,00025733
Tripartite motif-containing 12a	*Trim12a*	-1,48286217	0,00672338
Myeloblastosis oncogene-like 2	*Mybl2*	-1,48278446	8,09e-05
Melanoma associated antigen (mutated) 1	*Mum1*	-1,482563758	0,0003143
Basal cell adhesion molecule	*Bcam*	-1,482563743	0,00244164
Predicted pseudogene 9769 /// prostaglandin e synthase 3 (cytosolic)	*Gm9769 /// ptges3*	-1,482538171	8,22e-05
Spastin	*Spast*	-1,48243704	0,00139483
Predicted gene 15455 /// rna binding motif protein 10	*Gm15455 /// rbm10*	-1,482090856	0,00012345
Cklf-like marvel transmembrane domain containing 8	*Cmtm8*	-1,482001978	0,00021184

Non-smc element 1 homolog (s. Cerevisiae)	*Nsmce1*	-1,481998534	0,00073087
Ceramide synthase 4	*Cers4*	-1,481778916	0,00044084
Ubiquinol-cytochrome c reductase hinge protein	*Uqcrh*	-1,480900652	0,00034511
Cysteine rich, dpf motif domain containing 1	*Cdpf1*	-1,480623762	0,00020322
Atpase, h+/k+ exchanging, gastric, alpha polypeptide	*Atp4a*	-1,480415184	0,00184603
Cadherin 11 pseudogene	*2610005l07rik*	-1,4802921	0,00555378
Brx1, biogenesis of ribosomes, homolog (s. Cerevisiae)	*Brix1*	-1,480251952	0,00022415
Solute carrier family 13 (sodium-dependent dicarboxylate transporter), member 3	*Slc13a3*	-1,480153729	0,0043547
Cdna sequence bc029722	*Bc029722*	-1,480062854	0,00092711
Glutathione synthetase	*Gss*	-1,479931893	0,00014767
Ubiquitin specific peptidase 39	*Usp39*	-1,479921406	0,00027773
Glyceronephosphate o-acyltransferase	*Gnpat*	-1,479193911	6,95e-05
H2a histone family, member j	*H2afj*	-1,479085872	0,00044081
Slc10a3-ubl4 readthrough /// ubiquitin-like 4	*Slc10a3-ubl4 /// ubl4*	-1,478899662	0,00035454
Myosin, light polypeptide 6, alkali, smooth muscle and non-muscle pseudogene /// myosin light polypeptide 6 alkali smooth muscle and non-muscle protein, pseudogene /// myosin light polypeptide 6-like /// myosin, light polypeptide 6, alkali, smooth muscle and non-muscle	*Gm5526 /// gm8894 /// loc102643184 /// myl6*	-1,478774236	0,0001118
Udp-gal:betaglcnac beta 1,4- galactosyltransferase, polypeptide 1	*B4galt1*	-1,47866815	0,00027711
Mediator complex subunit 10	*Med10*	-1,478496832	9,66e-05
Lymphocyte antigen 6 complex, locus i	*Ly6i*	-1,478307488	0,00471572
Claudin 23	*Cldn23*	-1,478283646	0,00079655
Elongation factor tu gtp binding domain containing 2	*Eftud2*	-1,478056237	0,00150893
Riken cdna 2010012o05 gene	*2010012o05rik*	-1,47781782	0,00099311
Karyopherin (importin) alpha 6	*Kpna6*	-1,477760087	0,00109608
Ndc80 homolog, kinetochore complex component pseudogene	*2700099c18rik*	-1,477305211	0,00172354
Protein tyrosine phosphatase, receptor type, m	*Ptprm*	-1,47656057	0,00015524
Follistatin-like 3	*Fstl3*	-1,476494433	0,00087649
Pih1 domain containing 1	*Pih1d1*	-1,476422691	0,00015667
Tyrosine 3-monooxygenase/tryptophan 5-monooxygenase activation protein, zeta polypeptide	*Ywhaz*	-1,475824029	0,00298187
Sec31 homolog a (s. Cerevisiae)	*Sec31a*	-1,475380003	0,00051141
Tubulin, alpha 1b pseudogene /// tubulin, alpha 1b	*Gm5620 /// tuba1b*	-1,475316439	6,31e-05
Serine protease inhibitor 16	*Spi16*	-1,475040925	0,00959363
Fermitin family homolog 2 (drosophila)	*Fermt2*	-1,474899486	0,001299
Centromere protein t	*Cenpt*	-1,474893448	0,00047545
Cytochrome b5 type b	*Cyb5b*	-1,474608889	0,00092647
Optic atrophy 3	*Opa3*	-1,47442509	0,00150385
Tdp-glucose 4,6-dehydratase	*Tgds*	-1,474014591	0,00989754
Calpain 12	*Capn12*	-1,473759113	0,00531904
Metal response element binding transcription factor 1	*Mtf1*	-1,473269414	0,00075341

Riken cdna 1110004e09 gene	*1110004e09rik*	-1,473263504	0,00459917
Prohibitin 2	*Phb2*	-1,473074102	0,00375628
Tho complex 3	*Thoc3*	-1,472858657	0,00189483
Adp-ribosylation factor 5	*Arf5*	-1,472538177	0,0006084
Cleavage and polyadenylation specific factor 3-like	*Cpsf3l*	-1,472065807	0,00017348
Centromere protein c1	*Cenpc1*	-1,471716894	0,00822584
Recq protein-like	*Recql*	-1,471686554	0,00010189
Zinc finger protein 207	*Zfp207*	-1,47144352	0,00234805
Tudor domain containing 3	*Tdrd3*	-1,471378594	0,00195568
Potassium intermediate/small conductance calcium-activated channel, subfamily n, member 2	*Kcnn2*	-1,471365359	0,00517724
C-type lectin domain family 4, member g	*Clec4g*	-1,471103559	0,00316504
D4, zinc and double phd fingers family 1	*Dpf1*	-1,470296726	0,0062333
Cysteine-rich protein 3	*Crip3*	-1,470199355	0,00748619
Riken cdna 2310011j03 gene	*2310011j03rik*	-1,47011342	7,64e-05
B cell cll/lymphoma 7c	*Bcl7c*	-1,4691673	0,00164355
Fizzy/cell division cycle 20 related 1 (drosophila)	*Fzr1*	-1,469135605	0,00419534
Sumo1 activating enzyme subunit 1	*Sae1*	-1,469071044	0,00389022
Pseudopodium-enriched atypical kinase 1	*Peak1*	-1,468543247	0,00200734
Olfactory receptor 66	*Olfr66*	-1,468198474	0,00624628
Heterogeneous nuclear ribonucleoprotein d	*Hnrnpd*	-1,46802343	0,00745607
Ubiquitin protein ligase e3 component n-recognin 2	*Ubr2*	-1,467695656	0,00073086
Glutathione s-transferase, mu 7	*Gstm7*	-1,467442367	0,00176696
Cd7 antigen	*Cd7*	-1,467123257	0,00564962
Predicted gene, 26672	*Gm26672*	-1,466998082	0,00290875
Brain glycogen phosphorylase	*Pygb*	-1,466959421	0,0001516
Transmembrane protein 106b	*Tmem106b*	-1,466862144	0,00256361
N-deacetylase/n-sulfotransferase (heparan glucosaminyl) 3	*Ndst3*	-1,466859989	0,00069102
Potassium voltage-gated channel, shaker-related subfamily, beta member 2	*Kcnab2*	-1,466806284	0,00846077
Glutamate receptor, ionotropic, nmda2b (epsilon 2)	*Grin2b*	-1,466801115	0,00653568
Riken cdna 1110001j03 gene	*1110001j03rik*	-1,466460354	0,00084238
Family with sequence similarity 81, member a	*Fam81a*	-1,466452117	0,00090463
Calcium channel, voltage-dependent, alpha2/delta subunit 1	*Cacna2d1*	-1,466013437	0,00492574
Eukaryotic translation elongation factor 1 delta (guanine nucleotide exchange protein)	*Eef1d*	-1,465532166	0,00017135
Transcription factor 7 like 1 (t cell specific, hmg box)	*Tcf7l1*	-1,465426531	0,00274214
Natriuretic peptide type b	*Nppb*	-1,465426198	0,00043173
Interferon alpha 5	*Ifna5*	-1,465019339	0,0027345
Caprin family member 2	*Caprin2*	-1,464997977	0,00449362
Oxysterol binding protein-like 9	*Osbpl9*	-1,464912306	0,00662825
Pyridoxal (pyridoxine, vitamin b6) phosphatase	*Pdxp*	-1,464244811	0,0003191
Plexin b2	*Plxnb2*	-1,464197908	0,00137812
Ankyrin repeat domain 17	*Ankrd17*	-1,464185098	0,00068139
Rho family gtpase 2	*Rnd2*	-1,463897592	0,0008907

Tam41, mitochondrial translocator assembly and maintenance protein, homolog (s. Cerevisiae)	*Tamm41*	-1,463629815	0,00024709
Mob kinase activator 1b	*Mob1b*	-1,462922442	0,00011744
Ubiquitin interaction motif containing 1	*Uimc1*	-1,462829857	0,00017431
Eps8-like 1	*Eps8l1*	-1,461933252	0,00852365
Ewing sarcoma breakpoint region 1	*Ewsr1*	-1,461849859	0,00046337
Daz associated protein 2 /// predicted gene 2444	*Dazap2 /// gm2444*	-1,461834357	0,00027876
Cd82 antigen	*Cd82*	-1,461550798	0,00830559
Myosin ib	*Myo1b*	-1,461512844	0,00143315
Tripartite motif-containing 60	*Trim60*	-1,461331963	0,00306484
Putative homeodomain transcription factor 2	*Phtf2*	-1,46061258	0,00158844
Gins complex subunit 1 (psf1 homolog)	*Gins1*	-1,460507128	0,00094285
Myosin, heavy polypeptide 6, cardiac muscle, alpha /// myosin, heavy polypeptide 7, cardiac muscle, beta	*Myh6 /// myh7*	-1,460363334	0,00220444
Iq motif containing c	*Iqcc*	-1,460284914	0,00245637
Polymerase (dna-directed), delta 4	*Pold4*	-1,460268752	0,00042846
C-mer proto-oncogene tyrosine kinase	*Mertk*	-1,460085203	0,00050047
Translin-associated factor x	*Tsnax*	-1,460014682	0,00021132
Calpain, small subunit 1	*Capns1*	-1,459918337	0,00053212
Solute carrier family 22 (organic cation transporter), member 17	*Slc22a17*	-1,45926041	0,00395828
Sparc/osteonectin, cwcv and kazal-like domains proteoglycan 1	*Spock1*	-1,459175204	0,00091846
Rna binding motif, single stranded interacting protein 2	*Rbms2*	-1,458819485	0,00068855
Nuclear receptor subfamily 2, group f, member 6	*Nr2f6*	-1,458601814	0,00166707
Myeloid/lymphoid or mixed-lineage leukemia (trithorax homolog, drosophila); translocated to, 1	*Mllt1*	-1,458129759	0,00361887
Succinate-coenzyme a ligase, gdp-forming, beta subunit	*Suclg2*	-1,458099734	0,00656062
Sirtuin 1	*Sirt1*	-1,457091965	0,00186416
Hypoxia inducible factor 1, alpha subunit	*Hif1a*	-1,456769255	0,00021421
Prostate androgen-regulated mucin-like protein 1	*Parm1*	-1,456281296	0,00289745
Sry (sex determining region y)-box 4	*Sox4*	-1,456269103	0,00180612
F11 receptor	*F11r*	-1,455486348	0,00254198
Sphingomyelin phosphodiesterase 4	*Smpd4*	-1,455028008	0,00238059
Polymerase (dna directed), gamma	*Polg*	-1,454228633	0,00044395
Asparagine-linked glycosylation 9 (alpha 1,2 mannosyltransferase)	*Alg9*	-1,453879041	0,00089716
Rad51 homolog	*Rad51*	-1,453417465	0,00822584
General transcription factor ii e, polypeptide 2 (beta subunit)	*Gtf2e2*	-1,45322503	0,00143315
Heterogeneous nuclear ribonucleoprotein h1	*Hnrnph1*	-1,453109132	0,00353396
Heterogeneous nuclear ribonucleoprotein m	*Hnrnpm*	-1,452952933	0,00024589
Alveolar soft part sarcoma chromosome region, candidate 1 (human)	*Aspscr1*	-1,452902898	0,00024006
Minichromosome maintenance deficient 4 homolog (s. Cerevisiae)	*Mcm4*	-1,452577816	0,00656616
Potassium channel tetramerisation domain containing 20	*Kctd20*	-1,452436974	0,00048567

Rab3a interacting protein	*Rab3ip*	-1,451999677	0,00062558
Eukaryotic translation initiation factor 4b	*Eif4b*	-1,451950493	0,00018783
Dnaj (hsp40) homolog, subfamily c, member 17	*Dnajc17*	-1,451470026	0,00105045
Solute carrier family 9 (sodium/hydrogen exchanger), member 1	*Slc9a1*	-1,450783605	0,00063694
Frizzled homolog 5 (drosophila)	*Fzd5*	-1,450782905	0,00544602
Cdp-diacylglycerol synthase (phosphatidate cytidylyltransferase) 2	*Cds2*	-1,450716633	0,00033278
Sh3 binding domain protein 5 like	*Sh3bp5l*	-1,450663388	0,00028272
Fk506 binding protein 3	*Fkbp3*	-1,450330133	0,00722345
H1 histone family, member x	*H1fx*	-1,45031111	0,00044583
Pds5, regulator of cohesion maintenance, homolog a (s. Cerevisiae)	*Pds5a*	-1,449649243	0,00954046
Single-pass membrane protein with coiled-coil domains 4	*Smco4*	-1,449523398	0,00182679
Malonyl coa:acp acyltransferase (mitochondrial)	*Mcat*	-1,44934441	0,0001142
Nucleosome assembly protein 1-like 1	*Nap1l1*	-1,44898206	0,00179513
Gamma-aminobutyric acid (gaba) b receptor, 1	*Gabbr1*	-1,448927854	0,00348581
Phd finger protein 7	*Phf7*	-1,448611234	0,00155955
S100 calcium binding protein a6 (calcyclin)	*S100a6*	-1,447631468	0,00010023
Ubiquitin family domain containing 1	*Ubfd1*	-1,447522089	0,00098955
Protein kinase, membrane associated tyrosine/threonine 1	*Pkmyt1*	-1,446813032	0,00504549
Deoxyribonuclease 1-like 2	*Dnase1l2*	-1,446624214	0,00353331
Angiotensin i converting enzyme (peptidyl-dipeptidase a) 2	*Ace2*	-1,446541225	0,00548131
Charged multivesicular body protein 3	*Chmp3*	-1,446158914	0,00174965
Component of oligomeric golgi complex 1 /// conserved oligomeric golgi complex subunit 1-like	*Cog1 /// loc102641618*	-1,446010766	0,00073313
Arginase type ii	*Arg2*	-1,445765795	0,00238371
Adducin 1 (alpha)	*Add1*	-1,445649804	0,00026609
Transmembrane and coiled-coil domains 6	*Tmco6*	-1,445413795	0,00052334
Apolipoprotein a-ii	*Apoa2*	-1,445398171	0,00642073
Transmembrane protein 97	*Tmem97*	-1,445394889	0,00243614
Transmembrane bax inhibitor motif containing 6	*Tmbim6*	-1,445269631	0,00111027
Stimulated by retinoic acid 13	*Stra13*	-1,445252731	0,00198521
Prolactin receptor	*Prlr*	-1,445182932	0,00839063
Indoleamine 2,3-dioxygenase 1	*Ido1*	-1,444564081	0,0010338
Aquaporin 2	*Aqp2*	-1,444468768	0,00555912
Scl/tal1 interrupting locus	*Stil*	-1,443479432	0,00460778
Adp-ribosylhydrolase like 2	*Adprhl2*	-1,443216742	0,00083667
Fanconi anemia, complementation group c	*Fancc*	-1,443040447	0,00072165
Small nuclear ribonucleoprotein 27 (u4/u6.u5)	*Snrnp27*	-1,44254913	0,00015884
Potassium channel tetramerisation domain containing 9	*Kctd9*	-1,442443674	0,00368403
Charged multivesicular body protein 1a	*Chmp1a*	-1,442220008	0,00034325
Abhydrolase domain containing 1	*Abhd1*	-1,442052019	0,00313654
Ribosomal rna processing 1 homolog (s. Cerevisiae)	*Rrp1*	-1,441876177	0,00226

Family with sequence similarity 43, member a	*Fam43a*	-1,441630839	0,00056301
Neuroguidin, eif4e binding protein	*Ngdn*	-1,441627344	0,00104396
Gephyrin	*Gphn*	-1,441266048	0,00055159
Small glutamine-rich tetratricopeptide repeat (tpr)-containing, beta	*Sgtb*	-1,441229763	0,00053529
Adrenergic receptor kinase, beta 1	*Adrbk1*	-1,440870434	0,00306666
Regulation of nuclear pre-mrna domain containing 1b	*Rprd1b*	-1,440771634	0,00056124
Elongation of very long chain fatty acids (fen1/elo2, sur4/elo3, yeast)-like 1	*Elovl1*	-1,440460634	0,00014982
Nascent polypeptide-associated complex alpha polypeptide	*Naca*	-1,440392293	0,0001276
Ubiquitin specific peptidase 5 (isopeptidase t)	*Usp5*	-1,440249535	0,00060572
Alanyl-trna synthetase domain containing 1	*Aarsd1*	-1,439994513	0,0013628
Cyclin d3	*Ccnd3*	-1,439294175	0,00036798
Osteoglycin	*Ogn*	-1,438721745	0,00678426
Protein phosphatase 5, catalytic subunit	*Ppp5c*	-1,438438323	0,00146407
Histone aminotransferase 1	*Hat1*	-1,43796363	0,00143202
Beta-transducin repeat containing protein	*Btrc*	-1,437762912	0,0002572
Reproductive homeobox 2a /// reproductive homeobox 2e	*Rhox2a /// rhox2e*	-1,437514969	0,00938839
Polymerase (rna) iii (dna directed) polypeptide h	*Polr3h*	-1,436534831	0,00077082
X-prolyl aminopeptidase (aminopeptidase p) 1, soluble	*Xpnpep1*	-1,436409238	0,00060564
Serine/threonine kinase 40	*Stk40*	-1,436290304	0,00028524
Muts homolog 2 (e. Coli)	*Msh2*	-1,436242871	0,00205791
Maf1 homolog (s. Cerevisiae)	*Maf1*	-1,436070732	0,00635301
Gene trap locus 3	*Gtl3*	-1,435587451	0,00028029
Gamma-aminobutyric acid (gaba) a receptor-associated protein-like 1	*Gabarapl1*	-1,435297951	0,0003429
Trans-golgi network protein /// trans-golgi network protein 2	*Tgoln1 /// tgoln2*	-1,435214261	0,00083066
Propionyl coenzyme a carboxylase, beta polypeptide	*Pccb*	-1,435133279	0,00144613
Anaphase promoting complex subunit 16	*Anapc16*	-1,435103264	0,00038396
5-hydroxymethylcytosine (hmc) binding, es cell specific	*Hmces*	-1,434711316	0,00016189
C1galt1-specific chaperone 1	*C1galt1c1*	-1,434645196	0,0009131
Centrosomal protein 152	*Cep152*	-1,434377858	0,0005919
Insulin i	*Ins1*	-1,434362249	0,00837112
Mds1 and evi1 complex locus	*Mecom*	-1,43418122	0,00657866
Relaxin/insulin-like family peptide receptor 2	*Rxfp2*	-1,433761665	0,00132689
Caspase recruitment domain family, member 10	*Card10*	-1,433509402	0,00192881
Zinc finger protein 825	*Zfp825*	-1,433254257	0,00708139
Kv channel interacting protein 3, calsenilin	*Kcnip3*	-1,433096231	0,00642073
Nel-like 2	*Nell2*	-1,433064811	0,00788425
H2a histone family, member v	*H2afv*	-1,432660162	0,0002975
Riken cdna 6430706d22 gene /// riken cdna a730008h23 gene /// holliday junction recognition protein	*6430706d22rik /// a730008h23rik /// hjurp*	-1,432263779	0,00600976

Zinc finger protein 57	*Zfp57*	-1,431940104	0,00317117
Torsin a interacting protein 2	*Tor1aip2*	-1,431478115	0,00041846
Riken cdna 1700001g17 gene	*1700001g17rik*	-1,430913397	0,00112521
Splicing factor 3b, subunit 6	*Sf3b6*	-1,430822398	0,00048344
Pq loop repeat containing 1	*Pqlc1*	-1,430790829	0,0069415
Solute carrier family 15, member 4	*Slc15a4*	-1,430585915	0,00085531
Fun14 domain containing 2	*Fundc2*	-1,429961217	0,00159411
Guanosine monophosphate reductase 2	*Gmpr2*	-1,429862479	0,00168193
Cholecystokinin b receptor	*Cckbr*	-1,429704669	0,00174245
Glutathione peroxidase 8 (putative)	*Gpx8*	-1,429516527	0,00011929
Serum amyloid a 4	*Saa4*	-1,428542927	0,00895368
Fos-like antigen 1	*Fosl1*	-1,428457836	0,00174543
Solute carrier family 22 (organic cation transporter), member 1	*Slc22a1*	-1,427906414	0,00780281
Predicted gene 9744	*Gm9744*	-1,42768687	0,00335751
Protein-o-mannosyltransferase 2	*Pomt2*	-1,427542345	0,00114301
Serine (or cysteine) peptidase inhibitor, clade b, member 5	*Serpinb5*	-1,426969094	0,00160327
Retinol dehydrogenase 14 (all-trans and 9-cis)	*Rdh14*	-1,42677128	0,00294866
Bardet-biedl syndrome 9 (human)	*Bbs9*	-1,426103368	0,0094962
Agrin	*Agrn*	-1,425797546	0,00018582
Naked cuticle 2 homolog (drosophila)	*Nkd2*	-1,425724685	0,00861009
Cytochrome c oxidase assembly protein 15	*Cox15*	-1,425469656	0,00663015
Ubiquitin-associated protein 2-like	*Ubap2l*	-1,425313585	0,00155155
Lon peptidase 2, peroxisomal	*Lonp2*	-1,425312548	0,00053572
Erythrocyte protein band 4.1-like 3	*Epb4.1l3*	-1,424871072	0,00131254
Dexh (asp-glu-x-his) box polypeptide 58	*Dhx58*	-1,424861838	0,00254722
Rab4b, member ras oncogene family	*Rab4b*	-1,424507436	0,00217361
Gata binding protein 6	*Gata6*	-1,424227155	0,00428427
Solute carrier family 37 (glycerol-3-phosphate transporter), member 3	*Slc37a3*	-1,424204951	0,00472244
Coiled-coil domain containing 104	*Ccdc104*	-1,424113167	0,00108313
Pdgfa associated protein 1	*Pdap1*	-1,423870852	0,0077109
Lysyl oxidase-like 1	*Loxl1*	-1,4238641	0,00758388
Keratin associated protein 4-16	*Krtap4-16*	-1,423779574	0,00548837
Gamma-aminobutyric acid (gaba) a receptor, subunit alpha 1	*Gabra1*	-1,423716319	0,00061052
Riken cdna 2010111i01 gene	*2010111i01rik*	-1,423683926	0,00013532
Carbonic anyhydrase 12	*Car12*	-1,42349962	0,00291808
Zinc finger protein 52	*Zfp52*	-1,423027103	0,00079655
Caspase 2	*Casp2*	-1,422937861	0,0006737
Exoribonuclease 1	*Eri1*	-1,422724769	0,00792987
U2 small nuclear ribonucleoprotein auxiliary factor (u2af) 1	*U2af1*	-1,422484601	0,00016008
Neuronal pas domain protein 1	*Npas1*	-1,422227632	0,0005167
Visual system homeobox 2	*Vsx2*	-1,421742689	0,00701957
Testis expressed gene 2	*Tex2*	-1,421707278	0,00254627

Downstream neighbor of son	*Donson*	-1,421638015	0,00193828
Periaxin	*Prx*	-1,421590066	0,00609516
Leucine rich repeat containing 59	*Lrrc59*	-1,421423008	0,00208389
Ww domain containing transcription regulator 1	*Wwtr1*	-1,421193105	0,00089071
Pc4 and sfrs1 interacting protein 1	*Psip1*	-1,420680525	0,00091555
Dicarbonyl l-xylulose reductase	*Dcxr*	-1,420094935	0,00156293
G-protein signalling modulator 2 (ags3-like, c. Elegans)	*Gpsm2*	-1,419998483	0,00053227
Capicua homolog (drosophila)	*Cic*	-1,418607757	0,00415951
Uncharacterized loc102638039	*Loc102638039*	-1,418286606	0,00427759
Structural maintenance of chromosomes 4	*Smc4*	-1,417332793	0,0005919
Udp-n-acetyl-alpha-d-galactosamine: polypeptide n-acetylgalactosaminyltransferase 7	*Galnt7*	-1,417236902	0,00401187
Rna polymerase ii associated protein 3	*Rpap3*	-1,417150659	0,00116435
Mitogen-activated protein kinase kinase kinase 14	*Map3k14*	-1,416899274	0,00101906
Taste receptor, type 1, member 3	*Tas1r3*	-1,416753266	0,00548815
Adenylate cyclase activating polypeptide 1 receptor 1	*Adcyap1r1*	-1,415649415	0,00975858
Prp39 pre-mrna processing factor 39 homolog (yeast)	*Prpf39*	-1,415164066	0,00399465
Adaptor-related protein complex 2, beta 1 subunit	*Ap2b1*	-1,414850911	0,00355638
Sideroflexin 3	*Sfxn3*	-1,414816325	0,00227326
Usher syndrome 2a (autosomal recessive, mild)	*Ush2a*	-1,414775337	0,00732264
Family with sequence similarity 60, member a	*Fam60a*	-1,41460726	0,00097011
Sec13 homolog (s. Cerevisiae)	*Sec13*	-1,414552225	0,00105494
Apolipoprotein c-ii	*Apoc2*	-1,414544017	0,00356246
Centrosomal protein 89	*Cep89*	-1,414446149	0,00785734
Uridine-cytidine kinase 2	*Uck2*	-1,414440535	0,0002593
Trinucleotide repeat containing 6b	*Tnrc6b*	-1,414360989	0,00145685
Riken cdna 4930470p17 gene	*4930470p17rik*	-1,414311327	0,00333492
Zinc finger protein pseudogene	*Loc171588*	-1,41418044	0,00466856
Junction plakoglobin	*Jup*	-1,414011283	0,00116435
Efr3 homolog a (s. Cerevisiae)	*Efr3a*	-1,413929102	0,00218459
Nitric oxide synthase 1, neuronal	*Nos1*	-1,413322632	0,00750638
Trna methyltransferase 11-2	*Trmt112*	-1,413318327	0,00432019
Wd repeat domain 62	*Wdr62*	-1,413288809	0,00254837
Catenin, beta like 1	*Ctnnbl1*	-1,413250147	0,00028096
Udp-glucose pyrophosphorylase 2	*Ugp2*	-1,413226556	0,00117829
Cytochrome c oxidase assembly protein 14 /// cytochrome c oxidase assembly protein cox14-like	*Cox14 /// loc101055854*	-1,412926669	0,00083667
Protein kinase c, zeta	*Prkcz*	-1,412631401	0,00879646
Cytoplasmic fmr1 interacting protein 1	*Cyfip1*	-1,41260184	0,00155408
Cut-like homeobox 1	*Cux1*	-1,412573298	0,00508845
Phosphatidylglycerophosphate synthase 1	*Pgs1*	-1,412540445	0,00044604
Family with sequence similarity 160, member b1	*Fam160b1*	-1,412322949	0,0023616
Integrator complex subunit 1	*Ints1*	-1,41231392	0,00254308
Rap2c, member of ras oncogene family	*Rap2c*	-1,411975917	0,00493644

Syntaxin 2	*Stx2*	-1,411700642	0,00050512
Selenoprotein k	*Selk*	-1,411621088	0,00047172
Collagen, type ii, alpha 1	*Col2a1*	-1,411592006	0,00313677
Ptc7 protein phosphatase homolog (s. Cerevisiae)	*Pptc7*	-1,41100033	0,00050091
N-acetylneuraminic acid synthase (sialic acid synthase)	*Nans*	-1,410945311	0,00328333
Claudin 18	*Cldn18*	-1,410738807	0,00624337
Processing of precursor 5, ribonuclease p/mrp family (s. Cerevisiae)	*Pop5*	-1,410663779	0,00039022
Sphingosine-1-phosphate receptor 3	*S1pr3*	-1,410297338	0,00157814
Was protein family, member 1	*Wasf1*	-1,410236422	0,00154224
Glial cells missing homolog 1 (drosophila)	*Gcm1*	-1,409975161	0,00528704
Hmg box domain containing 4	*Hmgxb4*	-1,409810799	0,00098027
Proteasome (prosome, macropain) subunit, beta type 7 pseudogene	*Gm6787*	-1,409562599	0,00279933
Human immunodeficiency virus type i enhancer binding protein 3	*Hivep3*	-1,409338234	0,00131686
Acetyl-coenzyme a acyltransferase 2 (mitochondrial 3-oxoacyl-coenzyme a thiolase)	*Acaa2*	-1,408387819	0,00017572
St3 beta-galactoside alpha-2,3-sialyltransferase 3	*St3gal3*	-1,40808061	0,00070975
Sjogren's syndrome nuclear autoantigen 1	*Ssna1*	-1,407829656	0,0001862
Myosin, light polypeptide 3	*Myl3*	-1,407822082	0,00608678
Protein tyrosine phosphatase-like a domain containing 2	*Ptplad2*	-1,407747147	0,00173574
Riken cdna 1810037i17 gene /// predicted gene 2036	*1810037i17rik /// gm2036*	-1,407712715	0,001242
Ring finger and chy zinc finger domain containing 1	*Rchy1*	-1,407572533	0,00321777
Nuclear distribution gene e homolog 1 (a nidulans)	*Nde1*	-1,407549405	0,00074449
Thioredoxin-like 1	*Txnl1*	-1,407061709	0,00112558
Methyl-cpg binding domain protein 4	*Mbd4*	-1,406888946	0,0074602
Mechanistic target of rapamycin (serine/threonine kinase)	*Mtor*	-1,406851576	0,00367953
Kinesin family member 16b	*Kif16b*	-1,406552306	0,00806649
Swi/snf related, matrix associated, actin dependent regulator of chromatin, subfamily b, member 1	*Smarcb1*	-1,406529438	0,00130084
Histone cluster 1, h3d	*Hist1h3d*	-1,40642204	0,00154945
Sin3 associated polypeptide	*Sap30*	-1,406094071	0,00129091
Predicted gene, 21540 /// transmembrane emp24 domain trafficking protein 2	*Gm21540 /// tmed2*	-1,406059977	0,00755071
Tetratricopeptide repeat domain 4	*Ttc4*	-1,405993744	0,00178414
Zinc finger and btb domain containing 16	*Zbtb16*	-1,404775557	0,00596549
Receptor (calcitonin) activity modifying protein 2 /// vacuolar protein sorting 25 (yeast)	*Ramp2 /// vps25*	-1,40475293	0,00063958
Cytochrome p450, family 21, subfamily a, polypeptide 1	*Cyp21a1*	-1,404696475	0,00176856
Arylacetamide deacetylase (esterase)	*Aadac*	-1,404420628	0,00960645
Eph receptor b3	*Ephb3*	-1,403955651	0,00029525
Unc-5 homolog b (c. Elegans)	*Unc5b*	-1,403818479	0,0006562
Leucine-rich repeats and immunoglobulin-like domains 1	*Lrig1*	-1,403747	0,00932031

Methylphosphate capping enzyme	*Mepce*	-1,403682196	0,00265057
Max-like protein x	*Mlx*	-1,403667452	0,00047172
Adp-ribosylation factor-like 6 interacting protein 4	*Arl6ip4*	-1,40364862	0,00066524
Pith (c-terminal proteasome-interacting domain of thioredoxin-like) domain containing 1	*Pithd1*	-1,403619023	0,00043441
Corticotropin releasing hormone receptor 1	*Crhr1*	-1,403500509	0,00936858
Cd2 antigen	*Cd2*	-1,403455466	0,00605284
Potassium voltage-gated channel, shaker-related subfamily, beta member 3	*Kcnab3*	-1,403024083	0,00038659
Eukaryotic translation initiation factor 2b, subunit 4 delta	*Eif2b4*	-1,402820041	0,00172073
Guanine nucleotide binding protein (g protein), gamma 11	*Gng11*	-1,402669206	0,00321914
Sorting nexin 12	*Snx12*	-1,402597298	0,00150958
Inducible t cell co-stimulator	*Icos*	-1,402345049	0,0081553
Guanosine monophosphate reductase	*Gmpr*	-1,402312443	0,00445835
O-6-methylguanine-dna methyltransferase	*Mgmt*	-1,402159418	0,00733738
Transmembrane protein 87a	*Tmem87a*	-1,40211044	0,00448224
Ufm1-specific peptidase 2	*Ufsp2*	-1,401896617	0,00031242
Myosin, light polypeptide 6, alkali, smooth muscle and non-muscle pseudogene /// myosin light polypeptide 6 alkali smooth muscle and non-muscle protein, pseudogene /// myosin, light polypeptide 6, alkali, smooth muscle and non-muscle	*Gm5526 /// gm8894 /// myl6*	-1,401689699	0,00060056
Estrogen receptor-binding fragment-associated gene 9	*Ebag9*	-1,40162369	0,00461123
Polymerase (rna) ii (dna directed) polypeptide g	*Polr2g*	-1,401555638	0,00085105
Lysosomal-associated protein transmembrane 4a	*Laptm4a*	-1,401357116	0,0007071
Carbamoyl-phosphate synthetase 2, aspartate transcarbamylase, and dihydroorotase	*Cad*	-1,400926837	0,00101601
Wingless-type mmtv integration site family, member 2	*Wnt2*	-1,400924495	0,00232568
Stathmin-like 3	*Stmn3*	-1,400916237	0,00391427
Von willebrand factor a domain containing 1	*Vwa1*	-1,399518124	0,00731997
Potassium voltage gated channel, shab-related subfamily, member 1 /// protein kinase c and casein kinase substrate in neurons 2	*Kcnb1 /// pacsin2*	-1,399278253	0,00040671
Anaplastic lymphoma kinase	*Alk*	-1,39920703	0,00493499
Solute carrier family 25 (mitochondrial carnitine/acylcarnitine translocase), member 20	*Slc25a20*	-1,399148241	0,00322028
Ddb1 and cul4 associated factor 15	*Dcaf15*	-1,398900916	0,00114845
Malignant t cell amplified sequence 1	*Mcts1*	-1,398891735	0,00023259
Acetyl-coenzyme a carboxylase alpha	*Acaca*	-1,39889085	0,00125197
Testis specific protein-y encoded, pseudogene	*Tspy-ps*	-1,398782713	0,00040232
Pyrophosphatase (inorganic) 1	*Ppa1*	-1,398767726	0,00021699
Myotubularin related protein 14	*Mtmr14*	-1,398687684	0,00210903
Interleukin 31 receptor a	*Il31ra*	-1,398573375	0,00760304
Mitochondrial inner membrane organizing system 1	*Minos1*	-1,398470233	0,00077578
N(alpha)-acetyltransferase 10, nata catalytic subunit	*Naa10*	-1,398455744	0,00117488
Solute carrier family 4 (anion exchanger), member 2	*Slc4a2*	-1,398148184	0,00130583

Microrna 425	*Mir425*	-1,397898295	0,00793964
Predicted gene 6910 /// ring1 and yy1 binding protein	*Gm6910 /// rybp*	-1,397780643	0,00164429
Nuclear distribution gene c homolog (aspergillus)	*Nudc*	-1,397383453	0,0005355
Vimentin	*Vim*	-1,397144777	0,00019825
Interleukin 20	*Il20*	-1,396901642	0,00570311
Origin recognition complex, subunit 3	*Orc3*	-1,396447149	0,0004975
Phosphatidylinositol transfer protein, alpha	*Pitpna*	-1,396134088	0,00249177
Coiled-coil domain containing 166	*Ccdc166*	-1,396039709	0,00582168
Deoxyuridine triphosphatase	*Dut*	-1,394941667	0,00050986
Metastasis-associated gene family, member 2	*Mta2*	-1,394047357	0,00185315
Formin-like 1	*Fmnl1*	-1,393860356	0,00043277
Tropomyosin 3, gamma	*Tpm3*	-1,393822611	0,00119469
Negative elongation factor complex member a, whsc2	*Nelfa*	-1,393765058	0,00021087
Cadherin 5	*Cdh5*	-1,393739972	0,00376514
Serrate rna effector molecule homolog (arabidopsis)	*Srrt*	-1,393728656	0,00310895
Glutamate-cysteine ligase, modifier subunit	*Gclm*	-1,393660813	0,00052878
Ribonuclease p/mrp 30 subunit	*Rpp30*	-1,392486926	0,00218106
Proteasome (prosome, macropain) assembly chaperone 3	*Psmg3*	-1,390144827	0,0012794
Dual adaptor for phosphotyrosine and 3-phosphoinositides 1	*Dapp1*	-1,390138423	0,00188506
Myosin, light polypeptide 4	*Myl4*	-1,389675308	0,00934565
Ataxia, cerebellar, cayman type homolog (human)	*Atcay*	-1,389462625	0,00822207
Protein kinase c, epsilon	*Prkce*	-1,389400243	0,00304291
Zinc finger, c3hc type 1	*Zc3hc1*	-1,388828665	0,00204632
Taspase, threonine aspartase 1	*Tasp1*	-1,388412097	0,00187073
Sema domain, transmembrane domain (tm), and cytoplasmic domain, (semaphorin) 6b	*Sema6b*	-1,38834878	0,0014548
Sh3-domain kinase binding protein 1	*Sh3kbp1*	-1,388321334	0,0008106
Gnas (guanine nucleotide binding protein, alpha stimulating) complex locus	*Gnas*	-1,387660741	0,00451848
Stabilin 1	*Stab1*	-1,387437966	0,00196846
Transgelin 2	*Tagln2*	-1,387129419	0,00023515
B cell translocation gene 1, anti-proliferative	*Btg1*	-1,386941978	0,00338353
Capping protein (actin filament), gelsolin-like	*Capg*	-1,386791913	0,00042556
Tspy-like 4	*Tspyl4*	-1,386622169	0,00629125
E1a binding protein p400	*Ep400*	-1,386570443	0,00198703
Egl-9 family hypoxia-inducible factor 1	*Egln1*	-1,386502594	0,0011819
Eps8-like 2	*Eps8l2*	-1,38641667	0,00261112
Anaphase promoting complex subunit 7	*Anapc7*	-1,386130559	0,00052462
Plastin 3 (t-isoform)	*Pls3*	-1,385811758	0,00140306
Phosphofructokinase, muscle	*Pfkm*	-1,38576203	0,0002138
Family with sequence similarity 207, member a	*Fam207a*	-1,385431402	0,002135
Mitogen-activated protein kinase kinase 2	*Map2k2*	-1,385180636	0,00243169
Nuclear receptor subfamily 2, group e, member 3	*Nr2e3*	-1,384756314	0,00771566

Occludin/ell domain containing 1	*Ocel1*	-1,384439558	0,00183547
Heme binding protein 1	*Hebp1*	-1,384265794	0,00072165
Cue domain containing 2	*Cuedc2*	-1,383928688	0,00932043
Coiled-coil domain containing 71 like	*Ccdc71l*	-1,383905923	0,00395455
Oxysterol binding protein	*Osbp*	-1,383781957	0,00028524
Eukaryotic translation initiation factor 4h	*Eif4h*	-1,382877059	0,00049594
Phospholipase d2	*Pld2*	-1,382321813	0,00227744
Tbc1 domain family, member 22a	*Tbc1d22a*	-1,382254099	0,00058758
Tyrosine 3-monooxygenase/tryptophan 5-monooxygenase activation protein, theta polypeptide	*Ywhaq*	-1,38201679	0,0012441
N(alpha)-acetyltransferase 35, natc auxiliary subunit	*Naa35*	-1,381718882	0,00266959
Wilms tumour 1-associating protein	*Wtap*	-1,381586545	0,0023667
Ectonucleoside triphosphate diphosphohydrolase 5	*Entpd5*	-1,381257405	0,00283548
C-terminal binding protein 1	*Ctbp1*	-1,380826331	0,00026649
Fidgetin-like 1	*Fignl1*	-1,380744098	0,00822663
Mitochondrial ribosomal protein l54	*Mrpl54*	-1,380707184	0,00191521
Rho-associated coiled-coil containing protein kinase 2	*Rock2*	-1,380350107	0,00031703
Ligand dependent nuclear receptor corepressor	*Lcor*	-1,37971778	0,00662711
Purinergic receptor p2y, g-protein coupled 2	*P2ry2*	-1,379706199	0,00186209
Pas domain containing serine/threonine kinase	*Pask*	-1,379703024	0,00101541
Signal-induced proliferation-associated 1 like 2	*Sipa1l2*	-1,379683231	0,00278284
Coiled-coil-helix-coiled-coil-helix domain containing 5	*Chchd5*	-1,379411029	0,00312244
Det1 and ddb1 associated 1	*Dda1*	-1,378983944	0,00275905
Mus musculus, clone image:4481448, mrna.	*Bc049805*	-1,37863624	0,00183483
Aldehyde oxidase 3	*Aox3*	-1,378587649	0,00075735
Solute carrier family 25 (mitochondrial carrier, adenine nucleotide translocator), member 13	*Slc25a13*	-1,378522183	0,00668008
Solute carrier family 2 (facilitated glucose transporter), member 4	*Slc2a4*	-1,378303897	0,00161234
Cullin associated and neddylation disassociated 1	*Cand1*	-1,377641768	0,00123855
Translocase of outer mitochondrial membrane 20 homolog (yeast)	*Tomm20*	-1,377552724	0,00381753
Proteasome (prosome, macropain) subunit, alpha type 6	*Psma6*	-1,376961641	0,00088383
Adp-ribosylation factor-like 4a	*Arl4a*	-1,376801597	0,00185038
Was protein family, member 3	*Wasf3*	-1,376294425	0,00067901
Cerberus 1 homolog (xenopus laevis)	*Cer1*	-1,376104146	0,00356984
Zyxin	*Zyx*	-1,375870542	0,0008495
Autophagy related 5	*Atg5*	-1,375791073	0,00281405
Transmembrane bax inhibitor motif containing 4	*Tmbim4*	-1,375780796	0,0019757
Kelch-like 42	*Klhl42*	-1,375730698	0,0037143
Tgf-beta activated kinase 1/map3k7 binding protein 1	*Tab1*	-1,37569144	0,00466722
Riken cdna 1110035h17 gene	*1110035h17rik*	-1,375195894	0,00221395
N-ethylmaleimide sensitive fusion protein	*Nsf*	-1,374714366	0,00227326

Syntaxin 7	*Stx7*	-1,374630696	0,00083667
Pancreatic polypeptide	*Ppy*	-1,37437412	0,00327323
Aldo-keto reductase family 1, member a1 (aldehyde reductase)	*Akr1a1*	-1,374335317	0,00020409
Putative homeodomain transcription factor 1	*Phtf1*	-1,374268828	0,00053692
Harvey rat sarcoma virus oncogene	*Hras*	-1,373887735	0,0018961
Bromodomain containing 2	*Brd2*	-1,373778108	0,00301651
Potassium large conductance calcium-activated channel, subfamily m, beta member 4	*Kcnmb4*	-1,373253666	0,00128167
Aryl hydrocarbon receptor-interacting protein-like 1	*Aipl1*	-1,373199876	0,00299908
Serine hydroxymethyltransferase 1 (soluble)	*Shmt1*	-1,372844624	0,0004596
Acyl-coa desaturase 1-like /// stearoyl-coenzyme a desaturase 3	*Loc102642893 /// scd3*	-1,372627204	0,00197681
Uncharacterized loc269472	*Loc269472*	-1,372500818	0,00757049
Muts homolog 6 (e. Coli)	*Msh6*	-1,372494583	0,00105687
Profilin 3	*Pfn3*	-1,372310721	0,00666557
Urocanase domain containing 1	*Uroc1*	-1,371963397	0,00400303
B lymphoid kinase	*Blk*	-1,371519576	0,00552804
Aquaporin 7	*Aqp7*	-1,371385454	0,0034639
Malic enzyme 2, nad(+)-dependent, mitochondrial	*Me2*	-1,371349233	0,00068911
Staphylococcal nuclease and tudor domain containing 1	*Snd1*	-1,370999952	0,00028524
Mitochondrial ribosomal protein l18	*Mrpl18*	-1,370926189	0,00668008
Nadh dehydrogenase (ubiquinone) 1 alpha subcomplex, 9	*Ndufa9*	-1,370595456	0,00048627
Kelch domain containing 2	*Klhdc2*	-1,370540791	0,00316584
At rich interactive domain 3a (bright-like)	*Arid3a*	-1,370536137	0,00453944
Nad(p)h dehydrogenase, quinone 2	*Nqo2*	-1,370372854	0,00141645
Anti-silencing function 1a histone chaperone	*Asf1a*	-1,370240291	0,00112702
S-adenosylhomocysteine hydrolase	*Ahcy*	-1,370086587	0,00044734
Rhomboid domain containing 3	*Rhbdd3*	-1,369297091	0,00877622
Non-smc element 2 homolog (mms21, s. Cerevisiae)	*Nsmce2*	-1,369234694	0,00073086
Protein phosphatase 2, regulatory subunit b', gamma	*Ppp2r5c*	-1,368562592	0,00052085
Tumor necrosis factor (ligand) superfamily, member 12 /// tnfsf12-tnfsf13 readthrough transcript /// tumor necrosis factor (ligand) superfamily, member 13	*Tnfsf12 /// tnfsf12tnfsf13 /// tnfsf13*	-1,368156136	0,00367282
Williams-beuren syndrome chromosome region 16 homolog (human)	*Wbscr16*	-1,368112077	0,00109595
Kinesin family member 4	*Kif4*	-1,368089472	0,00114747
Cytochrome p450, family 2, subfamily d, polypeptide 22	*Cyp2d22*	-1,368062453	0,00186002
Disabled 1	*Dab1*	-1,36798637	0,00714958
Trna methyltransferase 44	*Trmt44*	-1,367982652	0,00122165
Spry domain containing 3	*Spryd3*	-1,3679604	0,00273151
Src family associated phosphoprotein 2	*Skap2*	-1,367707773	0,00092418
S100 calcium binding protein a9 (calgranulin b)	*S100a9*	-1,367463809	0,00394251
Haloacid dehalogenase-like hydrolase domain containing 1a	*Hdhd1a*	-1,367380264	0,00350555
Nitrogen permease regulator-like 3	*Nprl3*	-1,367169003	0,00287968

Centromere protein q	*Cenpq*	-1,367113874	0,00451259
Mdm2, transformed 3t3 cell double minute p53 binding protein	*Mtbp*	-1,366465392	0,00033525
Nadh dehydrogenase (ubiquinone) 1 beta subcomplex, 9	*Ndufb9*	-1,366318445	0,00052223
Angiogenin, ribonuclease, rnase a family, 5	*Ang*	-1,36585303	0,00460465
Ferm domain containing 5	*Frmd5*	-1,365693226	0,00257224
Nsfl1 (p97) cofactor (p47)	*Nsfl1c*	-1,365397537	0,00104349
X-ray repair complementing defective repair in chinese hamster cells 6	*Xrcc6*	-1,365175049	0,00059673
D4, zinc and double phd fingers, family 3	*Dpf3*	-1,36505584	0,00581312
Riken cdna 1700001c19 gene	*1700001c19rik*	-1,364709581	0,00843572
Per-pentamer repeat gene	*Ppnr*	-1,364584501	0,00203065
Ankyrin repeat domain 27 (vps9 domain)	*Ankrd27*	-1,364418873	0,00340259
Chemokine (c-x3-c motif) ligand 1	*Cx3cl1*	-1,36411256	0,0008714
Heat shock protein 8	*Hspa8*	-1,363366569	0,00214279
Fibromodulin	*Fmod*	-1,363312304	0,00930115
Copine i	*Cpne1*	-1,363240841	0,00053596
Feminization 1 homolog a (c. Elegans)	*Fem1a*	-1,362619839	0,00186702
Myotubularin related protein 4	*Mtmr4*	-1,3625164	0,00118256
G protein-regulated inducer of neurite outgrowth 1	*Gprin1*	-1,362500909	0,0032648
Kh domain containing, rna binding, signal transduction associated 1	*Khdrbs1*	-1,362414615	0,00992651
Vesicle transport through interaction with t-snares 1a	*Vti1a*	-1,362295714	0,00676254
Contactin 2	*Cntn2*	-1,36208294	0,00808303
Dead (asp-glu-ala-asp) box polypeptide 47	*Ddx47*	-1,361799028	0,00117656
Dead (asp-glu-ala-asp) box polypeptide 6	*Ddx6*	-1,361721514	0,00049753
Daz interacting protein 1	*Dzip1*	-1,361653231	0,00330358
Rhotekin	*Rtkn*	-1,361424263	0,00325122
Predicted gene 4887 /// twinfilin, actin-binding protein, homolog 1 (drosophila)	*Gm4887 /// twf1*	-1,361422461	0,00989754
Integrator complex subunit 6	*Ints6*	-1,361296315	0,00070156
Formin binding protein 4	*Fnbp4*	-1,361204015	0,00248865
Diaphanous homolog 1 (drosophila)	*Diap1*	-1,360825592	0,00328912
Family with sequence similarity 126, member a	*Fam126a*	-1,360732672	0,00859842
Epidermal growth factor receptor pathway substrate 15	*Eps15*	-1,360394115	0,00201559
Family with sequence similarity 96, member b	*Fam96b*	-1,360200936	0,00168846
Ring finger protein 181	*Rnf181*	-1,359993921	0,00084314
Quinoid dihydropteridine reductase	*Qdpr*	-1,359959596	0,00505884
Solute carrier family 25 (mitochondrial carrier, adenine nucleotide translocator), member 4	*Slc25a4*	-1,35992608	0,00595585
Artemin	*Artn*	-1,3595465	0,00421767
Mitochondrial calcium uptake 1	*Micu1*	-1,35892801	0,00169184
Rab24, member ras oncogene family	*Rab24*	-1,358902221	0,00208872
Sry (sex determining region y)-box 21	*Sox21*	-1,358791181	0,00588691
Scm-like with four mbt domains 1	*Sfmbt1*	-1,358735954	0,003192

Gs homeobox 1	*Gsx1*	-1,358335483	0,00682703
Rmi1, recq mediated genome instability 1, homolog (s. Cerevisiae)	*Rmi1*	-1,357453543	0,00061873
Coiled-coil domain containing 109b	*Ccdc109b*	-1,357229665	0,00418989
Negative elongation factor complex member e, rdbp	*Nelfe*	-1,356978969	0,00437069
Mitochondrial ribosomal protein s15	*Mrps15*	-1,356649837	0,00190734
Riken cdna 2610001j05 gene	*2610001j05rik*	-1,356498285	0,00046337
Glutamate receptor, ionotropic, nmda3b	*Grin3b*	-1,356487965	0,00732505
Enhancer of zeste homolog 1 (drosophila)	*Ezh1*	-1,356234111	0,00315256
N(alpha)-acetyltransferase 20, natb catalytic subunit	*Naa20*	-1,356193361	0,00222406
Flavin containing monooxygenase 4	*Fmo4*	-1,355992568	0,00819509
Homeobox a4	*Hoxa4*	-1,355685398	0,00944415
Small proline-rich protein 3	*Sprr3*	-1,355524989	0,0031207
Smg-5 homolog, nonsense mediated mrna decay factor (c. Elegans)	*Smg5*	-1,354176018	0,00270542
Dnaj (hsp40) homolog, subfamily c, member 11	*Dnajc11*	-1,354109885	0,00129693
Erythrocyte protein band 4.1-like 2	*Epb4.1l2*	-1,353771388	0,00089192
Mitogen-activated protein kinase 9	*Mapk9*	-1,352906398	0,00079041
Dnaj (hsp40) homolog, subfamily c, member 8	*Dnajc8*	-1,352537644	0,00192507
Myeloblastosis oncogene	*Myb*	-1,352382889	0,00837838
Tubulin, gamma 1	*Tubg1*	-1,351257976	0,00329181
Lysine (k)-specific methyltransferase 2e	*Kmt2e*	-1,351234876	0,00205494
Rab31, member ras oncogene family	*Rab31*	-1,350568249	0,00081342
Proteasome (prosome, macropain) subunit, alpha type 1	*Psma1*	-1,349820545	0,00192112
Polo-like kinase 3	*Plk3*	-1,349796835	0,00563832
Heterogeneous nuclear ribonucleoprotein u-like 1	*Hnrnpul1*	-1,34975417	0,00173814
Dead (asp-glu-ala-asp) box polypeptide 1	*Ddx1*	-1,349696324	0,0008106
Single-stranded dna binding protein 1	*Ssbp1*	-1,349494347	0,00090205
Rev3-like, catalytic subunit of dna polymerase zeta rad54 like (s. Cerevisiae)	*Rev3l*	-1,349185236	0,0099583
Lsm12 homolog (s. Cerevisiae)	*Lsm12*	-1,349166024	0,00523963
Sam domain and hd domain, 1	*Samhd1*	-1,348840401	0,00527879
Ptprf interacting protein, binding protein 2 (liprin beta 2)	*Ppfibp2*	-1,348748474	0,00711523
Keratin 15	*Krt15*	-1,348513833	0,00083959
Trans-2,3-enoyl-coa reductase	*Tecr*	-1,34800841	0,00389306
Gtpase, imap family member 3	*Gimap3*	-1,347988124	0,00590533
Solute carrier family 35, member a5	*Slc35a5*	-1,347645097	0,00480858
Chromatin accessibility complex 1	*Chrac1*	-1,347519287	0,0006881
Riken cdna 0610009o20 gene	*0610009o20rik*	-1,347400049	0,00531128
Inositol 1,3,4,5,6-pentakisphosphate 2-kinase	*Ippk*	-1,347148928	0,00407246
Serine peptidase inhibitor, kazal type 3	*Spink3*	-1,346737555	0,00931477
Butyrylcholinesterase	*Bche*	-1,346619857	0,00073116
Gap junction protein, delta 4	*Gjd4*	-1,345932061	0,00971606
Rho gtpase activating protein 17	*Arhgap17*	-1,345910833	0,00378658
E2f transcription factor 5	*E2f5*	-1,345723797	0,00080948

Minichromosome maintenance deficient 3 (s. Cerevisiae)	*Mcm3*	-1,345667533	0,0029945
Elastin	*Eln*	-1,345613139	0,00315182
Transmembrane protein 243, mitochondrial	*Tmem243*	-1,345360098	0,00204396
Coenzyme q6 homolog (yeast)	*Coq6*	-1,344839781	0,00298912
Set domain containing (lysine methyltransferase) 8	*Setd8*	-1,344341794	0,00579129
Collagen, type iii, alpha 1	*Col3a1*	-1,344326808	0,00193321
Antigen p97 (melanoma associated) identified by monoclonal antibodies 133.2 and 96.5	*Mfi2*	-1,343625327	0,00337105
Tubby like protein 1	*Tulp1*	-1,343615872	0,00560734
Relt-like 1	*Rell1*	-1,343541111	0,00387519
Transmembrane protein 209	*Tmem209*	-1,343313379	0,0082979
High density lipoprotein (hdl) binding protein	*Hdlbp*	-1,343280791	0,00904891
Ring finger and wd repeat domain 3	*Rfwd3*	-1,343032581	0,00067295
Myotubularin related protein 10	*Mtmr10*	-1,342826097	0,0005145
Solute carrier family 35 (udp-glucuronic acid/udp-n-acetylgalactosamine dual transporter), member d1	*Slc35d1*	-1,342397947	0,00581453
Nuclear receptor binding protein 1	*Nrbp1*	-1,341979119	0,00055189
A disintegrin and metallopeptidase domain 19 (meltrin beta)	*Adam19*	-1,341939341	0,00700387
Zinc finger, mynd-type containing 8	*Zmynd8*	-1,341758236	0,00484394
Muts homolog 3 (e. Coli)	*Msh3*	-1,341672031	0,00270835
Proteasome (prosome, macropain) 26s subunit, atpase 2	*Psmc2*	-1,339791827	0,00075463
Afg3-like aaa atpase 1	*Afg3l1*	-1,338829686	0,00259355
Ctd (carboxy-terminal domain, rna polymerase ii, polypeptide a) phosphatase, subunit 1	*Ctdp1*	-1,338579178	0,00901345
Nuclear receptor subfamily 2, group c, member 1	*Nr2c1*	-1,338236789	0,00299985
Pet100 homolog (s. Cerevisiae)	*Pet100*	-1,338186311	0,00933291
F-box and leucine-rich repeat protein 14	*Fbxl14*	-1,337880835	0,00374285
Circadian locomotor output cycles kaput	*Clock*	-1,337696602	0,00330167
Atpase, h+/k+ transporting, nongastric, alpha polypeptide	*Atp12a*	-1,33769012	0,00247154
Prp4 pre-mrna processing factor 4 homolog b (yeast)	*Prpf4b*	-1,337494461	0,0015523
Elks/rab6-interacting/cast family member 1	*Erc1*	-1,337076013	0,00369759
Otu deubiquitinase with linear linkage specificity	*Otulin*	-1,336859004	0,00098526
Bardet-biedl syndrome 2 (human)	*Bbs2*	-1,336528986	0,00263815
Riken cdna 1200014j11 gene	*1200014j11rik*	-1,336471829	0,00608704
Phosphoribosylaminoimidazole carboxylase, phosphoribosylaminoribosylaminoimidazole, succinocarboxamide synthetase	*Paics*	-1,336441038	0,00168846
Expressed sequence c77080	*C77080*	-1,336026101	0,00074303
Coatomer protein complex, subunit zeta 2	*Copz2*	-1,335273556	0,00316921
Transmembrane 4 superfamily member 4	*Tm4sf4*	-1,334967786	0,00726138
Myocilin	*Myoc*	-1,334777962	0,00110334
Rad50 homolog (s. Cerevisiae)	*Rad50*	-1,334627979	0,00117525
Glutamate receptor, ionotropic, delta 1	*Grid1*	-1,334347351	0,00574236
Family with sequence similarity 193, member b	*Fam193b*	-1,334096435	0,00268641

Mus musculus es cells cdna, riken full-length enriched library, clone:2410038p04 product:suppressor of variegation 3-9 homolog 1 (drosophila), full insert sequence. /// --- /// predicted gene 14820	*Ak010638 /// gm14820 /// gm14820*	-1,333945412	0,00447028
Nadh dehydrogenase (ubiquinone) 1 beta subcomplex, 10	*Ndufb10*	-1,333506904	0,00531904
Predicted gene, 21098 /// reproductive homeobox 4a /// reproductive homeobox 4b /// reproductive homeobox 4c /// reproductive homeobox 4d /// reproductive homeobox 4e /// reproductive homeobox 4f /// reproductive homeobox 4g	*Gm21098 /// rhox4a /// rhox4b /// rhox4c /// rhox4d /// rhox4e /// rhox4f /// rhox4g*	-1,333189185	0,00621877
Ubiquitin carboxyl-terminal esterase l3 (ubiquitin thiolesterase) /// ubiquitin carboxyl-terminal esterase l4	*Uchl3 /// uchl4*	-1,33304537	0,00644936
Calcium regulated heat stable protein 1	*Carhsp1*	-1,332997108	0,00053085
Histone deacetylase 3	*Hdac3*	-1,332749534	0,00053702
Ly1 antibody reactive clone	*Lyar*	-1,332651916	0,00328859
Myeloid zinc finger 1	*Mzf1*	-1,33235776	0,00300368
Mediator complex subunit 6	*Med6*	-1,33233527	0,00741957
Ceramide synthase 5	*Cers5*	-1,331928295	0,00195878
Karyopherin (importin) beta 1	*Kpnb1*	-1,331800152	0,00265343
Poly(a) binding protein, nuclear 1	*Pabpn1*	-1,331729148	0,00573458
Phospholipase b domain containing 2	*Plbd2*	-1,331574051	0,00750228
Predicted gene 5093 /// ribosomal protein l11	*Gm5093 /// rpl11*	-1,331380535	0,00885914
Ww domain binding protein 4	*Wbp4*	-1,330924198	0,00111322
Protein kinase c, theta	*Prkcq*	-1,330645826	0,0097813
Cleavage and polyadenylation specific factor 7	*Cpsf7*	-1,330476938	0,00146186
Cysteine-rich secretory protein lccl domain containing 1	*Crispld1*	-1,330180459	0,00078499
Suppressor of var1, 3-like 1 (s. Cerevisiae)	*Supv3l1*	-1,330143204	0,00100072
Solute carrier family 2 (facilitated glucose transporter), member 1	*Slc2a1*	-1,329984699	0,00126663
Neurocan	*Ncan*	-1,329952116	0,00625487
Lfng o-fucosylpeptide 3-beta-n-acetylglucosaminyltransferase	*Lfng*	-1,329699571	0,00443231
Upregulated during skeletal muscle growth 5	*Usmg5*	-1,329581966	0,00288419
Proline/serine-rich coiled-coil 1	*Psrc1*	-1,329504558	0,00405499
Chromosome segregation 1-like (s. Cerevisiae)	*Cse1l*	-1,329250951	0,00331687
Trans-acting transcription factor 1	*Sp1*	-1,328910774	0,00947937
Recname: full=ig gamma-2a chain c region secreted form; altname: full=b allele; /// recname: full=ig gamma-2a chain c region secreted form; altname: full=b allele; /// immunoglobulin heavy constant gamma 2c /// immunoglobulin heavy constant mu	*Igh /// ighg /// ighg2c /// ighm*	-1,328785633	0,00791706
Cytochrome p450, family 1, subfamily b, polypeptide 1	*Cyp1b1*	-1,328669977	0,00597309
Adaptor-related protein complex 2, sigma 1 subunit	*Ap2s1*	-1,328652076	0,00160831
Max dimerization protein 4	*Mxd4*	-1,328385404	0,00210704
Prominin 1	*Prom1*	-1,328053165	0,0094693
Cytochrome c oxidase assembly protein 16	*Cox16*	-1,327969328	0,0027735

Cysteine-rich with egf-like domains 2	*Creld2*	-1,327965475	0,0028769
Map3k12 binding inhibitory protein 1	*Mbip*	-1,327851962	0,00199928
Double c2, beta	*Doc2b*	-1,327696564	0,00382008
Dna-damage regulated autophagy modulator 1	*Dram1*	-1,327530027	0,00263532
Lectin, galactose binding, soluble 1	*Lgals1*	-1,327477786	0,00062642
Swi/snf related matrix associated, actin dependent regulator of chromatin, subfamily a-like 1	*Smarcal1*	-1,327331406	0,00861009
Microtubule-actin crosslinking factor 1	*Macf1*	-1,327186697	0,00635237
Kell blood group	*Kel*	-1,327058461	0,00487903
Frizzled homolog 2 (drosophila)	*Fzd2*	-1,326601535	0,00236693
Pleckstrin homology-like domain, family b, member 1	*Phldb1*	-1,326230211	0,00480782
Trm2 trna methyltransferase 2a	*Trmt2a*	-1,326196484	0,00142475
Ubx domain protein 1	*Ubxn1*	-1,326190228	0,00155095
Fk506 binding protein 1a	*Fkbp1a*	-1,325544051	0,00325173
Telo2 interacting protein 2	*Tti2*	-1,325292037	0,00439235
Protein phosphatase 1, regulatory subunit 21	*Ppp1r21*	-1,32518526	0,00203975
Wd repeat and fyve domain containing 2	*Wdfy2*	-1,324971696	0,00670683
Purinergic receptor p2x, ligand-gated ion channel, 1	*P2rx1*	-1,324806709	0,00988871
Ccz1 vacuolar protein trafficking and biogenesis associated	*Ccz1*	-1,324791602	0,0017004
Phosphoribosyl pyrophosphate synthetase-associated protein 1	*Prpsap1*	-1,324691664	0,00155474
Myosin x	*Myo10*	-1,324520309	0,00559783
Zinc finger and btb domain containing 25	*Zbtb25*	-1,324114574	0,00174323
Ribophorin i	*Rpn1*	-1,323832903	0,00068684
Ring finger protein 123	*Rnf123*	-1,323491384	0,00689963
Comm domain containing 5	*Commd5*	-1,323324436	0,00237529
Myosin ic	*Myo1c*	-1,323246717	0,0018325
Gli pathogenesis-related 2	*Glipr2*	-1,323226782	0,00142197
Nuclear factor of activated t cells 5	*Nfat5*	-1,323193399	0,0080933
Capping protein (actin filament) muscle z-line, alpha 2	*Capza2*	-1,322842918	0,00050719
Protein tyrosine phosphatase, receptor type, b	*Ptprb*	-1,32268762	0,00263352
Vaccinia related kinase 1	*Vrk1*	-1,322448331	0,00179834
Shroom family member 1	*Shroom1*	-1,322094787	0,00802338
Serine (or cysteine) peptidase inhibitor, clade i, member 1	*Serpini1*	-1,321998371	0,00304206
Mitochondrial carrier homolog 1 (c. Elegans)	*Mtch1*	-1,321546526	0,00466856
Polymerase (rna) ii (dna directed) polypeptide d	*Polr2d*	-1,321544223	0,00273541
Prefoldin 5	*Pfdn5*	-1,321007232	0,00387519
N-myc downstream regulated gene 1	*Ndrg1*	-1,32093686	0,00144734
Nadh dehydrogenase (ubiquinone) 1 alpha subcomplex, 4	*Ndufa4*	-1,320822123	0,00198703
Tissue factor pathway inhibitor 2	*Tfpi2*	-1,32065211	0,00477749
Predicted pseudogene 10651 /// josephin domain containing 1	*Gm10651 /// josd1*	-1,32050493	0,00170921

Regulatory factor x, 2 (influences hla class ii expression)	*Rfx2*	-1,3204287	0,00087214
Charged multivesicular body protein 2a	*Chmp2a*	-1,320395453	0,00288071
Muskelin 1, intracellular mediator containing kelch motifs	*Mkln1*	-1,31990338	0,00090907
Adaptor protein complex ap-1, gamma 1 subunit	*Ap1g1*	-1,319639767	0,00768702
Glutamyl-trna(gln) amidotransferase, subunit c homolog (bacterial)	*Gatc*	-1,318521746	0,00263352
Cdgsh iron sulfur domain 1	*Cisd1*	-1,317771694	0,00157984
Embryonic ectoderm development	*Eed*	-1,317572996	0,00221661
Riken cdna 5430435g22 gene	*5430435g22rik*	-1,316985108	0,00622664
Solute carrier family 22 (organic cation transporter), member 13	*Slc22a13*	-1,316808639	0,00609069
Ran binding protein 10	*Ranbp10*	-1,316704438	0,0021129
Swi/snf related, matrix associated, actin dependent regulator of chromatin, subfamily a, member 4	*Smarca4*	-1,316680966	0,00287786
Tnf receptor-associated factor 7	*Traf7*	-1,31606588	0,00068838
Tumor necrosis factor receptor superfamily, member 14 (herpesvirus entry mediator)	*Tnfrsf14*	-1,315649771	0,00885914
Vaccinia related kinase 2	*Vrk2*	-1,315508226	0,00513211
Tubulin, beta 2a class iia	*Tubb2a*	-1,315437059	0,00121214
Muts homolog 5 (e. Coli)	*Msh5*	-1,315311973	0,00463551
Trafficking protein particle complex subunit 10-like /// trafficking protein particle complex 10	*Loc102641872 /// trappc10*	-1,313890869	0,00174965
Polymerase (rna) ii (dna directed) polypeptide f	*Polr2f*	-1,313791994	0,00485527
Methionine aminopeptidase 2	*Metap2*	-1,313718764	0,00476067
Predicted gene 15583 /// zinc finger, bed domain containing 4	*Gm15583 /// zbed4*	-1,313178073	0,00102691
Fk506 binding protein 2	*Fkbp2*	-1,313104623	0,00212155
Damage specific dna binding protein 1	*Ddb1*	-1,312985441	0,00622694
Gpi anchor attachment protein 1	*Gpaa1*	-1,312877962	0,00167441
Cell cycle activator and apoptosis regulator 2	*Ccar2*	-1,312779326	0,00430995
Atpase, h+ transporting, lysosomal v1 subunit c1	*Atp6v1c1*	-1,31203968	0,00775678
Interleukin 17 receptor d	*Il17rd*	-1,312013044	0,00316584
Polymerase (rna) ii (dna directed) polypeptide c	*Polr2c*	-1,311335557	0,00117525
Dnaj (hsp40) homolog, subfamily c, member 2	*Dnajc2*	-1,310544183	0,00124373
Archain 1	*Arcn1*	-1,309996719	0,00137791
Dynein, axonemal assembly factor 2 /// ribosomal protein l36a /// ribosomal protein l36a-like	*Dnaaf2 /// rpl36a /// rpl36al*	-1,309825571	0,00096137
Eukaryotic translation initiation factor 3, subunit l	*Eif3l*	-1,309688734	0,0033141
Snf8, escrt-ii complex subunit, homolog (s. Cerevisiae)	*Snf8*	-1,309500335	0,00471582
Kelch-like ech-associated protein 1	*Keap1*	-1,309171427	0,00402377
Was protein family homolog	*Wash*	-1,308836488	0,00261044
Component of oligomeric golgi complex 1	*Cog1*	-1,308726666	0,00745746
Uridine-cytidine kinase 1-like 1	*Uckl1*	-1,308378905	0,00169268
Transcription factor 7, t cell specific	*Tcf7*	-1,308190521	0,0021129
Ornithine decarboxylase antizyme 1 /// ornithine decarboxylase antizyme 1, pseudogene	*Oaz1 /// oaz1-ps*	-1,308113645	0,00522148

Sphingomyelin synthase 1	*Sgms1*	-1,307685601	0,00413478
Iq motif containing gtpase activating protein 1	*Iqgap1*	-1,307586695	0,00257224
Leucine rich repeat containing 14	*Lrrc14*	-1,307484038	0,00249518
Angiotensinogen (serpin peptidase inhibitor, clade a, member 8)	*Agt*	-1,307452794	0,00230782
Protein kinase, camp dependent regulatory, type i, alpha	*Prkar1a*	-1,307112901	0,00207238
Prostate tumor over expressed gene 1	*Ptov1*	-1,307102959	0,00567925
Ubiquitin specific peptidase 49	*Usp49*	-1,306417208	0,00172038
Sorting nexin family member 21	*Snx21*	-1,306404451	0,00809986
Nad kinase	*Nadk*	-1,306307769	0,00345
Predicted gene 7008	*Gm7008*	-1,306295584	0,00097157
Triple functional domain (ptprf interacting)	*Trio*	-1,306146432	0,00585119
Atpase, h+ transporting, lysosomal v0 subunit a1	*Atp6v0a1*	-1,306123616	0,00777495
Short stature homeobox 2	*Shox2*	-1,306118348	0,00784472
Gram domain containing 4	*Gramd4*	-1,306016378	0,00254043
Gonadotropin releasing hormone receptor	*Gnrhr*	-1,305523614	0,00992651
Prenylcysteine oxidase 1	*Pcyox1*	-1,305148599	0,00190759
Structural maintenance of chromosomes 1a	*Smc1a*	-1,305121019	0,00182275
Pentatricopeptide repeat domain 2	*Ptcd2*	-1,304451081	0,00236549
Target of myb1-like 2 (chicken)	*Tom1l2*	-1,30437179	0,00772106
Dbf4 homolog (s. Cerevisiae)	*Dbf4*	-1,303694807	0,00652788
Small integral membrane protein 3	*Smim3*	-1,303571651	0,00081184
Bloom syndrome, recq helicase-like	*Blm*	-1,303462993	0,00916158
Mannosidase, beta a, lysosomal-like	*Manbal*	-1,303354305	0,00367869
Immunoglobulin heavy constant mu	*Ighm*	-1,303327395	0,00479781
Brca1 interacting protein c-terminal helicase 1, opposite strand	*Brip1os*	-1,302771668	0,0009038
A kinase (prka) interacting protein 1	*Akip1*	-1,302672476	0,00172821
Intraflagellar transport 74	*Ift74*	-1,301804231	0,00871834
Cytochrome c oxidase assembly factor 6 /// predicted gene, 17296	*Coa6 /// gm17296*	-1,301684675	0,00299983
Inositol 1,3,4-triphosphate 5/6 kinase	*Itpk1*	-1,301266342	0,00400812
Suppression of tumorigenicity 7	*St7*	-1,301171008	0,0032266
Cadherin-related family member 5	*Cdhr5*	-1,300913971	0,00334199
Ring finger protein 41	*Rnf41*	-1,300490866	0,002289
Rna 2',3'-cyclic phosphate and 5'-oh ligase	*Rtcb*	-1,300480028	0,00153182
E74-like factor 5	*Elf5*	-1,300346897	0,0089453
Zinc finger protein 395	*Zfp395*	-1,300251244	0,00846254
Mtor associated protein, lst8 homolog (s. Cerevisiae)	*Mlst8*	-1,300176834	0,00720735
Resistin like beta	*Retnlb*	-1,300106265	0,00638481
Scaffold attachment factor b	*Safb*	-1,300102793	0,00732771
S-adenosylmethionine decarboxylase 1	*Amd1*	-1,300024071	0,0024092
Proacrosin binding protein	*Acrbp*	-1,29962059	0,0049058
Cts telomere maintenance complex component 1	*Ctc1*	-1,299068874	0,00304609
Mitochondrial ribosomal protein s36	*Mrps36*	-1,299055367	0,00094888

Methylmalonic aciduria (cobalamin deficiency) type b homolog (human)	*Mmab*	-1,298846572	0,00998402
Fxyd domain-containing ion transport regulator 5	*Fxyd5*	-1,298693629	0,00581267
Basic leucine zipper transcription factor, atf-like 3	*Batf3*	-1,298324508	0,0059457
Inhibitor of growth family, member 3	*Ing3*	-1,298082979	0,00137683
Profilin 2	*Pfn2*	-1,298071788	0,00485894
G protein-coupled receptor 180	*Gpr180*	-1,297951457	0,00302655
Cyclin e1	*Ccne1*	-1,297594135	0,00301078
Coiled-coil domain containing 34	*Ccdc34*	-1,297450078	0,00233389
Riken cdna b230219d22 gene	*B230219d22rik*	-1,296844335	0,00277407
Coronin, actin binding protein 1b	*Coro1b*	-1,296786066	0,00776905
Unc-45 homolog a (c. Elegans)	*Unc45a*	-1,29674474	0,00220052
Comm domain containing 6	*Commd6*	-1,296679989	0,00534584
Zinc finger protein 91	*Zfp91*	-1,296510442	0,00443919
Cytochrome c-1	*Cyc1*	-1,296222446	0,00218311
Pantothenate kinase 3	*Pank3*	-1,296100998	0,00448965
Comm domain containing 1	*Commd1*	-1,296034476	0,00248953
Germ cell-less homolog 1 (drosophila)	*Gmcl1*	-1,29594052	0,00789226
Tuftelin interacting protein 11	*Tfip11*	-1,295831545	0,00772185
Mediator complex subunit 22	*Med22*	-1,295646252	0,00626979
Syndecan 2	*Sdc2*	-1,295607918	0,00338353
Hyaluronoglucosaminidase 1 /// n-acetyltransferase 6	*Hyal1 /// nat6*	-1,295187601	0,00733648
Myristoylated alanine rich protein kinase c substrate	*Marcks*	-1,295175383	0,00237535
Seminal vesicle secretory protein 3a /// seminal vesicle secretory protein 3b	*Svs3a /// svs3b*	-1,294953598	0,00368974
Surfactant associated protein c	*Sftpc*	-1,294363451	0,00783701
Ruvb-like protein 1	*Ruvbl1*	-1,294158337	0,00162375
Kruppel-like factor 12	*Klf12*	-1,293774807	0,00275989
F-box protein 21	*Fbxo21*	-1,293558175	0,00578939
Wilms tumor 1 homolog	*Wt1*	-1,293261567	0,00732771
Solute carrier family 25 (mitochondrial carrier, phosphate carrier), member 3	*Slc25a3*	-1,2929984	0,00177217
Protease, serine 40	*Prss40*	-1,292365475	0,00867599
Proteasome (prosome, macropain) subunit, beta type 3	*Psmb3*	-1,292202837	0,00915267
Nadh dehydrogenase (ubiquinone) 1 beta subcomplex, 2	*Ndufb2*	-1,29217104	0,00814302
Atp synthase, h+ transporting, mitochondrial f1 complex, delta subunit	*Atp5d*	-1,291637831	0,00378039
Retinol binding protein 3, interstitial	*Rbp3*	-1,291584781	0,0088249
Solute carrier family 30 (zinc transporter), member 5	*Slc30a5*	-1,291160575	0,00902024
Sh3-domain grb2-like 1	*Sh3gl1*	-1,290849622	0,0017069
Predicted gene 10257 /// predicted gene 12657 /// predicted pseudogene 6749 /// h3 histone, family 3a /// h3 histone, family 3b /// h3 histone, family 3c	*Gm10257 /// gm12657 /// gm6749 /// h3f3a /// h3f3b /// h3f3c*	-1,290222761	0,00973926
Tropomyosin 4	*Tpm4*	-1,290028116	0,00103541

Perp, tp53 apoptosis effector	*Perp*	-1,289560589	0,00458591
Dedicator of cytokinesis 6	*Dock6*	-1,289184693	0,00927077
Btb and cnc homology 1	*Bach1*	-1,289174467	0,00488863
Sperm associated antigen 6	*Spag6*	-1,289097276	0,00609537
Signal peptide peptidase 3	*Sppl3*	-1,288781875	0,00202368
Sad1 and unc84 domain containing 2	*Sun2*	-1,28857797	0,00130131
Potassium channel, subfamily k, member 3	*Kcnk3*	-1,288553733	0,00790625
Peptidyl-trna hydrolase domain containing 1	*Ptrhd1*	-1,288443966	0,00800054
Chromobox 1	*Cbx1*	-1,288184963	0,00634871
Ferm, rhogef (arhgef) and pleckstrin domain protein 1 (chondrocyte-derived)	*Farp1*	-1,28779983	0,00771545
Cop9 (constitutive photomorphogenic) homolog, subunit 4 (arabidopsis thaliana)	*Cops4*	-1,287714594	0,00592989
Cystatin 10 (chondrocytes)	*Cst10*	-1,287599009	0,00912545
Biliverdin reductase a	*Blvra*	-1,28691137	0,00367912
Regenerating islet-derived 3 gamma	*Reg3g*	-1,286723769	0,00697073
Ribosomal protein l41	*Rpl41*	-1,286381277	0,00976707
Tachykinin receptor 1	*Tacr1*	-1,286070787	0,00681488
Adrenergic receptor, alpha 2c	*Adra2c*	-1,285738708	0,00860236
Carboxylesterase 4a	*Ces4a*	-1,285256641	0,00750583
Vasoactive intestinal polypeptide	*Vip*	-1,285250465	0,00376049
Tumor necrosis factor receptor superfamily, member 8	*Tnfrsf8*	-1,285212687	0,0089498
Abra c-terminal like /// predicted pseudogene 6314	*Abracl /// gm6314*	-1,28502546	0,00490399
Uncharacterized loc102634352 /// snf2-related crebbp activator protein	*Loc102634352 /// srcap*	-1,284878191	0,00327787
Protein phosphatase 1d magnesium-dependent, delta isoform	*Ppm1d*	-1,284763883	0,0027438
Interleukin 10 receptor, alpha	*Il10ra*	-1,284755923	0,00555912
Megakaryocyte-associated tyrosine kinase	*Matk*	-1,284029231	0,00535424
Transmembrane protein 39a	*Tmem39a*	-1,283954663	0,00595468
Dsn1, mind kinetochore complex component, homolog (s. Cerevisiae)	*Dsn1*	-1,283709866	0,00945271
Transmembrane protein 55a	*Tmem55a*	-1,283685399	0,00927041
Adenylosuccinate lyase	*Adsl*	-1,283609785	0,00308967
Small nuclear ribonucleoprotein polypeptide g	*Snrpg*	-1,283288615	0,00447812
Growth differentiation factor 2	*Gdf2*	-1,283170765	0,00822584
Heterogeneous nuclear ribonucleoprotein h3	*Hnrnph3*	-1,283066236	0,00447648
Coxsackie virus and adenovirus receptor	*Cxadr*	-1,282979839	0,00869216
Frequently rearranged in advanced t cell lymphomas	*Frat1*	-1,282968187	0,0029824
Annexin a1	*Anxa1*	-1,28268253	0,00283106
Protein phosphatase 1, regulatory subunit 10 pseudogene /// protein phosphatase 1, regulatory subunit 10	*Gm8801 /// ppp1r10*	-1,281987898	0,00350555
Dynactin 2	*Dctn2*	-1,28198165	0,00349824
Udp-n-acetyl-alpha-d-galactosamine:polypeptide n-acetylgalactosaminyltransferase 11	*Galnt11*	-1,281660171	0,00558055
Atpase, ca++ transporting, cardiac muscle, fast twitch 1	*Atp2a1*	-1,281531369	0,00570279

Solute carrier family 39 (zinc transporter), member 3	*Slc39a3*	-1,281218148	0,00227737
Ras homolog gene family, member d	*Rhod*	-1,280785478	0,00639226
Radial spoke 3a homolog (chlamydomonas) /// radial spoke 3b homolog (chlamydomonas)	*Rsph3a /// rsph3b*	-1,27999638	0,00268564
Sec24 related gene family, member c (s. Cerevisiae)	*Sec24c*	-1,279856985	0,00374598
Potassium channel modulatory factor 1	*Kcmf1*	-1,279760115	0,00237822
Ino80 complex subunit c	*Ino80c*	-1,279637202	0,0075302
Taurine upregulated gene 1	*Tug1*	-1,279599747	0,00785121
Striatin, calmodulin binding protein 4	*Strn4*	-1,278646138	0,00916016
Replication initiator 1	*Repin1*	-1,278236635	0,00331089
Asparagine-linked glycosylation 2 (alpha-1,3-mannosyltransferase)	*Alg2*	-1,278207037	0,00588691
Tp53rk binding protein	*Tprkb*	-1,27733954	0,00504489
Succinate dehydrogenase complex assembly factor 2	*Sdhaf2*	-1,276838195	0,00232091
Predicted gene 13669 /// proteolipid protein 2	*Gm13669 /// plp2*	-1,276559617	0,00680105
Ddb1 and cul4 associated factor 13	*Dcaf13*	-1,276390458	0,00208389
Transcription factor 25 (basic helix-loop-helix)	*Tcf25*	-1,275564606	0,00212128
Matrix metallopeptidase 17	*Mmp17*	-1,275478435	0,00242877
Small nuclear rna activating complex, polypeptide 3	*Snapc3*	-1,27539835	0,00287369
Dead (asp-glu-ala-asp) box polypeptide 19b	*Ddx19b*	-1,274955157	0,00452839
Small nuclear rna activating complex, polypeptide 2	*Snapc2*	-1,274886672	0,00478068
Nyn domain and retroviral integrase containing	*Nynrin*	-1,274825043	0,00647084
C-type lectin domain family 4, member a2 /// c-type lectin domain family 4, member b1	*Clec4a2 /// clec4b1*	-1,274739638	0,00766852
Eukaryotic translation initiation factor 3, subunit b	*Eif3b*	-1,274379967	0,00413057
Rna binding motif protein 8a	*Rbm8a*	-1,273351667	0,00403954
Heterochromatin protein 1, binding protein 3	*Hp1bp3*	-1,273135296	0,00191154
Translocase of outer mitochondrial membrane 22 homolog (yeast)	*Tomm22*	-1,272735787	0,00200546
Tetraspanin 4	*Tspan4*	-1,272695186	0,00694592
Zinc finger protein 286	*Zfp286*	-1,272017806	0,00582168
Cop9 (constitutive photomorphogenic) homolog, subunit 3 (arabidopsis thaliana)	*Cops3*	-1,271741471	0,00175797
Ubiquitin-conjugating enzyme e2a	*Ube2a*	-1,27090399	0,0082239
Rala binding protein 1	*Ralbp1*	-1,270608367	0,00360257
Solute carrier family 38, member 2	*Slc38a2*	-1,270483443	0,00288048
Adenylosuccinate synthetase like 1	*Adssl1*	-1,270157446	0,00608192
Emg1 nucleolar protein homolog (s. Cerevisiae)	*Emg1*	-1,270125869	0,00609516
Growth differentiation factor 3	*Gdf3*	-1,269936167	0,00937722
Succinate-coa ligase, gdp-forming, alpha subunit	*Suclg1*	-1,268633382	0,00285202
Dpy-30 homolog (c. Elegans)	*Dpy30*	-1,268238353	0,00548668
Sine oculis-binding protein homolog (drosophila)	*Sobp*	-1,267983528	0,00518113
Kaptin	*Kptn*	-1,267951747	0,00695972
Swi/snf related, matrix associated, actin dependent regulator of chromatin, subfamily a, member 2	*Smarca2*	-1,267926806	0,00923893
Huntingtin interacting protein 1 related	*Hip1r*	-1,26783685	0,00822584
Lim domain binding 1	*Ldb1*	-1,267725087	0,00720703

Sorting nexin 15	*Snx15*	-1,267391804	0,00293635
Three prime repair exonuclease 2	*Trex2*	-1,267155879	0,00693519
Predicted pseudogene 10224 /// predicted gene 4581 /// ribosomal protein l15 pseudogene /// ribosomal protein l15	*Gm10224 /// gm4581 /// gm6937 /// rpl15*	-1,266736688	0,00412713
Atpase, h+ transporting, lysosomal v1 subunit e1	*Atp6v1e1*	-1,266650307	0,00916375
Prostaglandin e receptor 4 (subtype ep4)	*Ptger4*	-1,265393941	0,00740536
Lethal giant larvae homolog 1 (drosophila)	*Llgl1*	-1,26465007	0,00336144
Zinc finger, dhhc domain containing 3	*Zdhhc3*	-1,264396085	0,00434732
Max interacting protein 1	*Mxi1*	-1,26406778	0,00695126
Phosphofurin acidic cluster sorting protein 1	*Pacs1*	-1,263976701	0,00531904
Atpase inhibitory factor 1	*Atpif1*	-1,26383981	0,00783224
Signal transducer and activator of transcription 5b	*Stat5b*	-1,263279356	0,00614877
Cytohesin 2	*Cyth2*	-1,262732749	0,00584268
Copine ii	*Cpne2*	-1,262676666	0,00965095
Potassium channel tetramerisation domain containing 2	*Kctd2*	-1,261555995	0,00674746
Actin-like 6a	*Actl6a*	-1,261469968	0,0062189
Chemokine (c-x-c motif) receptor 3	*Cxcr3*	-1,261357117	0,00234648
Solute carrier family 32 (gaba vesicular transporter), member 1	*Slc32a1*	-1,261042002	0,00176696
Gamma-aminobutyric acid (gaba) c receptor, subunit rho 1	*Gabrr1*	-1,261017677	0,00806229
T cell leukemia translocation altered gene	*Tcta*	-1,260881827	0,00498344
Oleoyl-acp hydrolase	*Olah*	-1,260493271	0,00858526
Special at-rich sequence binding protein 2	*Satb2*	-1,260213326	0,00397201
Riken cdna 1810009a15 gene /// uncharacterized loc102308570	*1810009a15rik /// loc102308570*	-1,259864798	0,00588047
Signal peptide peptidase like 2b	*Sppl2b*	-1,259821209	0,00536445
Zinc finger, dhhc domain containing 7	*Zdhhc7*	-1,25932163	0,00298957
Membrane metallo-endopeptidase-like 1	*Mmel1*	-1,259089966	0,00699317
Mediator complex subunit 15	*Med15*	-1,258957429	0,00907424
Polybromo 1	*Pbrm1*	-1,258408236	0,00853597
Regulator of chromosome condensation (rcc1) and btb (poz) domain containing protein 2	*Rcbtb2*	-1,25839039	0,00308771
Vaccinia related kinase 3	*Vrk3*	-1,258119721	0,00587475
Potassium voltage gated channel, shaw-related subfamily, member 3	*Kcnc3*	-1,257624758	0,00348333
Family with sequence similarity 107, member b	*Fam107b*	-1,257176547	0,00846216
Nucleosome assembly protein 1-like 4	*Nap1l4*	-1,25456094	0,00204135
Wingless-type mmtv integration site family, member 4	*Wnt4*	-1,253937725	0,00956963
Ribosomal protein s27-like	*Rps27l*	-1,253578191	0,00659344
Heterogeneous nuclear ribonucleoprotein u	*Hnrnpu*	-1,253564016	0,00511649
Regulator of chromosome condensation 2	*Rcc2*	-1,253303131	0,00983452
Bromodomain and phd finger containing, 1	*Brpf1*	-1,253017365	0,0032912
Phosphatidylethanolamine binding protein 1	*Pebp1*	-1,25284696	0,00254722
Minichromosome maintenance deficient 10 (s. Cerevisiae)	*Mcm10*	-1,252741446	0,00633809

Rad9-hus1-rad1 interacting nuclear orphan 1	*Rhno1*	-1,25257807	0,00953696
4haus augmin-like complex, subunit 8	*Haus8*	-1,25248116	0,00519729
Prp19/pso4 pre-mrna processing factor 19 homolog (s. Cerevisiae)	*Prpf19*	-1,251983802	0,00512705
Eukaryotic translation initiation factor 2b, subunit 1 (alpha)	*Eif2b1*	-1,251833367	0,00444548
Glucose 6 phosphatase, catalytic, 3	*G6pc3*	-1,251797156	0,00926959
Tuberous sclerosis 2	*Tsc2*	-1,250716052	0,00770645
Predicted gene 14680 /// spermine synthase	*Gm14680 /// sms*	-1,250360089	0,00351588
Menage a trois 1	*Mnat1*	-1,248710927	0,00733686
High mobility group box transcription factor 1	*Hbp1*	-1,248626157	0,0082284
Msh homeobox 2	*Msx2*	-1,24815437	0,00767055
Regulator of chromosome condensation 1	*Rcc1*	-1,248018299	0,00947937
Ubiquitin-conjugating enzyme e2k	*Ube2k*	-1,247524969	0,00471475
Peptidase d	*Pepd*	-1,247106573	0,00430021
Wingless-type mmtv integration site family, member 3a	*Wnt3a*	-1,246793106	0,00519914
Tubulin folding cofactor b	*Tbcb*	-1,24661991	0,00724938
Dna-directed rna polymerases i, ii, and iii subunit rpabc4-like /// polymerase (rna) ii (dna directed) polypeptide k	*Loc100862456 /// polr2k*	-1,245673032	0,00910935
Craniofacial development protein 1	*Cfdp1*	-1,244202983	0,00877622
Defensin, alpha 1 /// defensin, alpha, 24 /// defensin, alpha, 6 /// predicted gene 15284	*Defa1 /// defa24 /// defa6 /// gm15284*	-1,244083276	0,00998734
Zinc finger protein 334	*Zfp334*	-1,243776629	0,00820637
Egl-9 family hypoxia-inducible factor 2	*Egln2*	-1,243471243	0,00827594
Coiled-coil-helix-coiled-coil-helix domain containing 2 /// predicted gene 13202 /// zinc finger, bed-type containing 5	*Chchd2 /// gm13202 /// zbed5*	-1,242588608	0,0055019
Ubiquitin-conjugating enzyme e2i	*Ube2i*	-1,2419851	0,00632858
General transcription factor iih, polypeptide 3	*Gtf2h3*	-1,238313393	0,00842948
Pr domain containing 4	*Prdm4*	-1,238043546	0,00887623
Cholinergic receptor, muscarinic 3, cardiac	*Chrm3*	-1,236320986	0,00364285
Predicted gene 13826 /// ribosomal protein l37	*Gm13826 /// rpl37*	-1,23566714	0,00356217
Mitochondrial ribosomal protein l51	*Mrpl51*	-1,23533522	0,00736325
Cklf-like marvel transmembrane domain containing 3	*Cmtm3*	-1,233525254	0,00419857
Polypyrimidine tract binding protein 3	*Ptbp3*	-1,231294014	0,00789226
Denn/madd domain containing 5a	*Dennd5a*	-1,230039802	0,00442372
Poly-u binding splicing factor 60	*Puf60*	-1,227596704	0,00661854
Adp-ribosylation factor-like 2	*Arl2*	-1,227415901	0,00927264
Protease (prosome, macropain) 26s subunit, atpase 5	*Psmc5*	-1,227123339	0,00880016
Transcription elongation regulator 1 (ca150)	*Tcerg1*	-1,226740732	0,00503581
Ankyrin repeat, sam and basic leucine zipper domain containing 1	*Asz1*	-1,225462021	0,00822142
Kallikrein related-peptidase 8	*Klk8*	-1,223364439	0,00910723
Riken cdna 4833439l19 gene	*4833439l19rik*	-1,221033817	0,00612549
Wnk lysine deficient protein kinase 4	*Wnk4*	-1,221006319	0,00746155

Solute carrier family 25 (mitochondrial carrier, adenine nucleotide translocator), member 5	*Slc25a5*	-1,220139831	0,00605377
Wd repeat domain 13	*Wdr13*	-1,214748595	0,00740162
Zinc finger protein 626	*Zfp626*	-1,21449398	0,00756253
Ribosomal protein l8	*Rpl8*	-1,213983673	0,00487537
Dcp1 decapping enzyme homolog a (s. Cerevisiae)	*Dcp1a*	-1,212706335	0,00953689
Small nucleolar rna host gene 5	*Snhg5*	-1,211091976	0,00640516
Vacuolar protein sorting 26 homolog b (yeast)	*Vps26b*	-1,208253187	0,0080769
Ribosomal protein l30 pseudogene /// predicted gene 5481 /// predicted gene 6109 /// predicted gene 6570 /// predicted pseudogene 7429 /// ribosomal protein l30	*Gm12191 /// gm5481 /// gm6109 /// gm6570 /// gm7429 /// rpl30*	-1,207257227	0,00768027
Melanophilin	*Mlph*	-1,205824449	0,00962622
Srebf chaperone	*Scap*	-1,203876941	0,00764528

Table S10. Genes with significantly changed expression in JUN-2fos3 compared to JUN-2

Gene.title	Gene.symbol	2^logFC	adj.p.val
Stathmin-like 2	*Stmn2*	77,2935674	1,69e-10
Plasmacytoma expressed transcript 2	*Pet2*	69,9509256	6,55e-10
Calponin 1	*Cnn1*	47,9372067	1,69e-10
Calcitonin-related polypeptide, beta	*Calcb*	47,2099442	1,14e-10
Small proline-rich protein 2a1 /// small proline-rich protein 2a2	*Sprr2a1 /// sprr2a2*	28,2413931	1,69e-10
Developmental pluripotency associated 4	*Dppa4*	26,805494	2,32e-09
Small proline-rich protein 2a1 /// small proline-rich protein 2a2 /// small proline-rich protein 2a3	*Sprr2a1 /// sprr2a2 /// sprr2a3*	26,0266498	2,88e-10
Solute carrier family 9, subfamily b (nha1, cation proton antiporter 1), member 1	*Slc9b1*	18,0201168	4,21e-10
Melanoma antigen, family a, 1 /// melanoma antigen, family a, 2 /// melanoma antigen, family a, 3 /// melanoma antigen, family a, 5 /// melanoma antigen, family a, 6 /// melanoma antigen, family a, 8	*Magea1 /// magea2 /// magea3 /// magea5 /// magea6 /// magea8*	17,9026106	1,55e-08
Neurofilament, light polypeptide	*Nefl*	17,2423414	1,15e-09
Insulin-like growth factor 2	*Igf2*	14,8363185	2,36e-07
Rab6b, member ras oncogene family	*Rab6b*	14,21816	7,81e-08
Developmental pluripotency associated 2	*Dppa2*	12,8996917	3,39e-09
Forkhead box d1	*Foxd1*	12,8372288	1,88e-09
Reproductive homeobox 2a /// reproductive homeobox 2e	*Rhox2a /// rhox2e*	12,2951675	1,39e-08
Acid phosphatase 5, tartrate resistant	*Acp5*	11,9972844	8,50e-09
Lymphocyte antigen 6 complex, locus c1 /// lymphocyte antigen 6 complex, locus c2	*Ly6c1 /// ly6c2*	10,9417873	6,37e-10
Hemopoietic cell kinase	*Hck*	10,6917211	6,39e-10
Glutathione s-transferase kappa 1	*Gstk1*	10,6478852	3,57e-09
Alpha fetoprotein	*Afp*	10,2930344	8,05e-07
Teneurin transmembrane protein 3	*Tenm3*	10,0738464	2,27e-07
Epoxide hydrolase 1, microsomal	*Ephx1*	10,0113212	6,39e-10
Gap junction protein, beta 5	*Gjb5*	9,4008104	2,72e-08
Myosin viib	*Myo7b*	8,98880198	1,37e-07
Scm-like with four mbt domains 2	*Sfmbt2*	8,68269695	6,08e-08
Small proline-rich protein 2f	*Sprr2f*	8,57157781	1,44e-09
Chemokine (c-x-c motif) receptor 6	*Cxcr6*	8,37785616	4,31e-07
Carboxymethylenebutenolidase-like (pseudomonas)	*Cmbl*	8,11059288	7,87e-09
Tetraspanin 2	*Tspan2*	7,72638388	2,52e-06
Lysozyme 1	*Lyz1*	7,69294233	1,20e-08
Sema domain, immunoglobulin domain (ig), short basic domain, secreted, (semaphorin) 3a	*Sema3a*	7,68063391	9,31e-09
Sry (sex determining region y)-box 2	*Sox2*	7,25141769	2,12e-09
Atpase, ca++ transporting, type 2c, member 2	*Atp2c2*	7,04356126	1,50e-07
Myosin vc	*Myo5c*	7,00020011	3,06e-07
Keratin 13	*Krt13*	6,89409544	9,22e-08
Lymphocyte antigen 6 complex, locus a	*Ly6a*	6,83471808	6,55e-10
Premature ovarian failure 1b	*Pof1b*	6,7664017	3,06e-08

Insulin-like growth factor binding protein 4	*Igfbp4*	6,73256183	2,91e-08
Protein tyrosine phosphatase, non-receptor type 20	*Ptpn20*	6,72901101	2,74e-07
Ankyrin repeat and socs box-containing 17	*Asb17*	6,6353602	1,44e-09
Cd80 antigen	*Cd80*	6,6333538	1,18e-08
Predicted gene 10439 /// predicted gene 15080 /// predicted gene 15085 /// predicted gene 15093 /// predicted gene 15107 /// predicted gene 15114 /// predicted gene 15128 /// leucine zipper protein 4 /// ovary testis transcribed	*Gm10439 /// gm15080 /// gm15085 /// gm15093 /// gm15107 /// gm15114 /// gm15128 /// luzp4 /// ott*	6,62776383	2,63e-06
Matrilin 2	*Matn2*	6,50760295	7,28e-09
Keratin associated protein 4-16	*Krtap4-16*	6,48960376	5,05e-08
C-fos induced growth factor	*Figf*	6,44100772	3,11e-08
Endothelial cell-specific molecule 1	*Esm1*	6,24575694	4,57e-08
Myosin vb	*Myo5b*	6,11135457	1,62e-07
Fragile x mental retardation 1 neighbor	*Fmr1nb*	5,95906853	1,56e-08
Otospiralin	*Otos*	5,94254142	4,52e-07
Spindle and kinetochore associated complex subunit 1	*Ska1*	5,81585493	1,24e-08
Regulator of g-protein signaling 2	*Rgs2*	5,7749453	9,42e-08
Paired immunoglobin-like type 2 receptor alpha	*Pilra*	5,63447953	1,86e-07
Lim and senescent cell antigen like domains 2	*Lims2*	5,56254091	2,72e-07
Potassium voltage-gated channel, shaker-related subfamily, beta member 1	*Kcnab1*	5,45425744	3,10e-07
Cd244 natural killer cell receptor 2b4	*Cd244*	5,40936762	9,13e-06
Predicted gene 10058 /// predicted gene 10096 /// predicted gene 10147 /// predicted gene 10230 /// predicted gene 10486 /// predicted gene 10487 /// predicted gene 10488 /// predicted gene 14525 /// predicted gene 14632 /// predicted gene 14819 /// predicted pseudogene 2005 /// predicted gene 2012 /// predicted gene 2030 /// predicted gene 4297 /// predicted gene 4836 /// predicted gene 5169 /// predicted gene 5934 /// predicted gene 6121	*Gm10058 /// gm10096 /// gm10147 /// gm10230 /// gm10486 /// gm10487 /// gm10488 /// gm14525 /// gm14632 /// gm14819 /// gm2005 /// gm2012 /// gm2030 /// gm4297 /// gm4836 /// gm5169 /// gm5934 /// gm6121*	5,36983152	9,08e-09
Carbohydrate sulfotransferase 11 /// phosphatase and actin regulator 1	*Chst11 /// phactr1*	5,24907438	3,44e-08
Collagen, type vi, alpha 3	*Col6a3*	5,24569248	8,53e-09
Arrestin, beta 1	*Arrb1*	5,17232705	7,41e-08
Formin homology 2 domain containing 3	*Fhod3*	5,1617969	1,52e-08
Family with sequence similarity 198, member b	*Fam198b*	5,12417057	4,86e-07
Chromodomain helicase dna binding protein 3, opposite strand	*Chd3os*	5,10009649	6,72e-07
Neuropeptide y receptor y1	*Npy1r*	5,08969923	2,04e-08
Family with sequence similarity 84, member a	*Fam84a*	5,08187561	4,71e-07

Transglutaminase 2, c polypeptide	*Tgm2*	5,04569562	5,45e-08
Chemokine (c-c motif) ligand 2	*Ccl2*	4,99294583	1,78e-07
Chemokine (c-c motif) ligand 7	*Ccl7*	4,96727777	4,60e-06
Microtubule-associated protein 6	*Map6*	4,91216722	1,54e-08
Synaptopodin	*Synpo*	4,84167084	5,89e-09
Gli pathogenesis-related 1 (glioma)	*Glipr1*	4,80686902	1,76e-08
Calponin 2	*Cnn2*	4,79337664	6,19e-08
Isochorismatase domain containing 1	*Isoc1*	4,75479086	7,28e-09
Tlc domain containing 2	*Tlcd2*	4,68534585	1,51e-08
Collagen, type iv, alpha 5	*Col4a5*	4,63382488	5,19e-07
Histocompatibility 2, m region locus 9	*H2-m9*	4,61605491	2,86e-08
Thrombomodulin	*Thbd*	4,60836247	1,92e-05
Fragile histidine triad gene	*Fhit*	4,59136186	4,27e-06
Actin, alpha 1, skeletal muscle	*Acta1*	4,51696006	6,24e-08
Follistatin-like 3	*Fstl3*	4,47615208	6,17e-08
Ribonuclease p/mrp 25 subunit	*Rpp25*	4,46950604	3,14e-06
Cytochrome p450, family 2, subfamily d, polypeptide 22	*Cyp2d22*	4,40019085	3,06e-08
Transmembrane protein with egf-like and two follistatin-like domains 2	*Tmeff2*	4,37016455	1,00e-07
Melanoma antigen, family a, 5	*Magea5*	4,36851646	3,98e-07
Retinol dehydrogenase 5	*Rdh5*	4,33460162	8,05e-08
Cubilin (intrinsic factor-cobalamin receptor)	*Cubn*	4,31465912	1,41e-07
Chemokine (c-c motif) ligand 11	*Ccl11*	4,3079159	2,84e-07
Gata binding protein 6	*Gata6*	4,30522872	2,23e-07
Protein kinase c, delta binding protein	*Prkcdbp*	4,27037297	2,71e-08
Dna-damage-inducible transcript 4-like	*Ddit4l*	4,26784707	6,63e-08
Transgelin	*Tagln*	4,21718471	7,55e-08
Kallikrein related-peptidase 7 (chymotryptic, stratum corneum)	*Klk7*	4,18382851	3,20e-06
Ras homolog gene family, member j	*Rhoj*	4,18123079	9,16e-09
Msh homeobox 3	*Msx3*	4,17746376	3,75e-08
Protein kinase, cgmp-dependent, type ii	*Prkg2*	4,17653842	1,04e-07
Gene regulated by estrogen in breast cancer protein	*Greb1*	4,10330019	2,56e-06
Guanylate cyclase activator 2a (guanylin)	*Guca2a*	4,09926161	2,98e-06
Adrenergic receptor, alpha 1b	*Adra1b*	4,08247324	5,25e-07
Solute carrier family 26 (sulfate transporter), member 2	*Slc26a2*	4,05840145	1,73e-07
3'-phosphoadenosine 5'-phosphosulfate synthase 2	*Papss2*	4,05440137	5,93e-08
Chemokine (c-c motif) ligand 8	*Ccl8*	4,04350509	3,28e-08
Aldehyde dehydrogenase family 1, subfamily a3	*Aldh1a3*	4,03965569	1,54e-07
Insulin-like growth factor binding protein 6	*Igfbp6*	4,02098359	1,01e-07
Riken cdna 4930529m08 gene	*4930529m08rik*	4,02090185	0,00016329
Rab27b, member ras oncogene family	*Rab27b*	4,00072293	2,31e-06
Rho gtpase activating protein 18	*Arhgap18*	3,99309386	1,62e-07
Steroidogenic acute regulatory protein	*Star*	3,96128261	7,05e-08
Dysferlin	*Dysf*	3,91226553	7,69e-08

Macro domain containing 2	*Macrod2*	3,90662556	3,65e-07
Cytochrome b-561	*Cyb561*	3,89502764	2,73e-07
Inhibitor of dna binding 3	*Id3*	3,8939997	1,23e-08
G1 to s phase transition 2	*Gspt2*	3,89064542	2,90e-07
Colony stimulating factor 1 receptor	*Csf1r*	3,88367657	1,42e-07
Poly(rc) binding protein 3	*Pcbp3*	3,87982807	1,28e-06
Carboxypeptidase z	*Cpz*	3,86420138	1,67e-06
Guanylate binding protein 3	*Gbp3*	3,83727286	7,69e-07
Sema domain, seven thrombospondin repeats (type 1 and type 1-like), transmembrane domain (tm) and short cytoplasmic domain, (semaphorin) 5a	*Sema5a*	3,82595482	7,78e-08
Protein tyrosine phosphatase-like a domain containing 2	*Ptplad2*	3,81842052	1,17e-07
Cyclin g1	*Ccng1*	3,81011495	0,00016818
Spleen tyrosine kinase	*Syk*	3,80431897	1,73e-06
Dystrobrevin alpha	*Dtna*	3,78879385	6,74e-07
Fyn-related kinase	*Frk*	3,77721322	3,66e-05
Advillin	*Avil*	3,77604855	4,20e-07
Potassium intermediate/small conductance calcium-activated channel, subfamily n, member 2	*Kcnn2*	3,77245096	1,09e-06
Betacellulin, epidermal growth factor family member	*Btc*	3,74665178	1,00e-07
Serine (or cysteine) peptidase inhibitor, clade b, member 1a	*Serpinb1a*	3,71143639	1,89e-07
Melanoma antigen, family a, 6	*Magea6*	3,70808239	8,69e-07
Cytochrome b5 reductase 3	*Cyb5r3*	3,6968487	3,65e-07
Dnaj (hsp40) homolog, subfamily a, member 4	*Dnaja4*	3,68232177	1,81e-06
Sperm antigen with calponin homology and coiled-coil domains 1	*Specc1*	3,63527272	5,88e-08
Pleckstrin homology domain containing, family a (phosphoinositide binding specific) member 1	*Plekha1*	3,63360656	1,92e-08
Dynamin 1	*Dnm1*	3,61521611	8,63e-07
Cd109 antigen	*Cd109*	3,61099852	3,05e-07
Reproductive homeobox 5	*Rhox5*	3,56740688	1,83e-07
Star-related lipid transfer (start) domain containing 4	*Stard4*	3,56355674	1,55e-06
Rab3d, member ras oncogene family	*Rab3d*	3,55104863	4,32e-06
Udp-gal:betaglcnac beta 1,3-galactosyltransferase, polypeptide 1	*B3galt1*	3,51742927	1,97e-06
Doublecortin-like kinase 1	*Dclk1*	3,49617264	5,26e-07
Arylsulfatase i	*Arsi*	3,4882318	1,35e-07
Lim domain binding 3	*Ldb3*	3,48557085	2,14e-06
Phosphofructokinase, liver, b-type	*Pfkl*	3,48279138	0,00086227
Tumor necrosis factor receptor superfamily, member 9	*Tnfrsf9*	3,48034844	6,67e-08
D-dopachrome tautomerase	*Ddt*	3,47383247	1,18e-07
Max dimerization protein 3	*Mxd3*	3,46411394	1,22e-07
Insulin-like growth factor 2 mrna binding protein 1	*Igf2bp1*	3,46092006	6,20e-07
Chloride channel calcium activated 4	*Clca4*	3,45929447	0,00022698

Predicted gene 7040 /// prolactin family 2, subfamily c, member 2 /// prolactin family 2, subfamily c, member 3 /// prolactin family 2, subfamily c, member 4	*Gm7040 /// prl2c2 /// prl2c3 /// prl2c4*	3,43330158	1,68e-08
Neurofilament, medium polypeptide	*Nefm*	3,43248482	6,51e-07
Pyridoxal (pyridoxine, vitamin b6) phosphatase	*Pdxp*	3,42989515	7,46e-08
Tubulin polyglutamylase complex subunit 2	*Tpgs2*	3,4253748	2,16e-08
Selenoprotein p, plasma, 1	*Sepp1*	3,41983052	1,86e-07
Methyltransferase like 7a1	*Mettl7a1*	3,41058335	2,61e-07
Ropporin 1-like	*Ropn1l*	3,3994532	7,35e-06
Camp responsive element modulator	*Crem*	3,39311098	9,83e-06
Immunoglobulin superfamily, member 5	*Igsf5*	3,35978771	0,00010537
Cd48 antigen	*Cd48*	3,35059823	5,35e-07
Serine (or cysteine) peptidase inhibitor, clade b, member 8	*Serpinb8*	3,34837404	3,62e-05
Transmembrane protein 158	*Tmem158*	3,3482845	6,63e-08
Predicted gene 3716	*Gm3716*	3,32956205	5,69e-07
Carboxypeptidase x 2 (m14 family)	*Cpxm2*	3,32679593	2,02e-05
Acylphosphatase 2, muscle type	*Acyp2*	3,32590682	6,48e-06
Collagen, type iv, alpha 6	*Col4a6*	3,31809745	0,00012009
Prickle homolog 1 (drosophila)	*Prickle1*	3,31138708	3,72e-07
Gipc pdz domain containing family, member 2	*Gipc2*	3,30076635	7,12e-07
Fibrinogen-like protein 2	*Fgl2*	3,29096894	3,90e-06
Meiosis-specific nuclear structural protein 1	*Mns1*	3,2900455	1,21e-07
Ww, c2 and coiled-coil domain containing 1	*Wwc1*	3,25322111	4,69e-06
Adenylosuccinate synthetase like 1	*Adssl1*	3,23673581	1,00e-07
Cd244 natural killer cell receptor 2b4 /// natural killer cell receptor 2b4-like	*Cd244 /// loc677008*	3,23493633	1,12e-05
Microtubule-associated protein 1b	*Map1b*	3,22297803	2,20e-05
Olfactomedin-like 3	*Olfml3*	3,20081977	1,25e-07
Transforming growth factor, beta induced	*Tgfbi*	3,18321938	8,83e-08
Dicarbonyl l-xylulose reductase	*Dcxr*	3,17930365	3,53e-07
Phosphofructokinase, platelet	*Pfkp*	3,17720959	2,83e-05
Testis derived transcript	*Tes*	3,17587508	2,59e-08
Isopentenyl-diphosphate delta isomerase	*Idi1*	3,16953242	2,36e-07
Solute carrier family 8 (sodium/calcium exchanger), member 1	*Slc8a1*	3,16802676	4,68e-06
Pannexin 1	*Panx1*	3,16493846	4,85e-08
Abhydrolase domain containing 3	*Abhd3*	3,15141233	0,00010614
Opticin	*Optc*	3,14841509	1,88e-07
Phosphatase and actin regulator 1	*Phactr1*	3,14163175	5,89e-06
Riken cdna 9030617o03 gene	*9030617o03rik*	3,14100017	5,19e-07
Plakophilin 3	*Pkp3*	3,13874952	1,24e-06
Progressive ankylosis	*Ank*	3,13873064	1,86e-07
Receptor accessory protein 5	*Reep5*	3,12681332	0,00147998
Cyclin-dependent kinase inhibitor 1a (p21)	*Cdkn1a*	3,12326867	2,01e-05
A disintegrin and metallopeptidase domain 8	*Adam8*	3,10931671	3,07e-07

Coiled-coil domain containing 104	*Ccdc104*	3,08333823	3,00e-07
Neural precursor cell expressed, developmentally down-regulated gene 4-like	*Nedd4l*	3,07976274	3,31e-08
Solute carrier organic anion transporter family, member 2a1	*Slco2a1*	3,06126712	8,60e-07
Glutamate receptor interacting protein 1	*Grip1*	3,05532238	6,71e-06
Transmembrane protein 141	*Tmem141*	3,05098236	8,54e-08
Cysteine conjugate-beta lyase 2	*Ccbl2*	3,05030969	4,97e-06
Impact, rwd domain protein	*Impact*	3,03609358	2,07e-07
Adrenomedullin	*Adm*	3,01009944	5,09e-06
Glycophorin c	*Gypc*	3,00466801	3,16e-07
Solute carrier family 39 (metal ion transporter), member 8	*Slc39a8*	2,98146393	0,00126306
Cyclin d1	*Ccnd1*	2,98027022	4,39e-07
Lim domain only 1	*Lmo1*	2,97606148	4,66e-07
Sulfatase 2	*Sulf2*	2,96798434	5,26e-07
Leucine-rich repeat kinase 2	*Lrrk2*	2,96041702	6,13e-07
Sclerostin domain containing 1	*Sostdc1*	2,95847526	5,98e-06
Phosphodiesterase 1b, ca2+-calmodulin dependent	*Pde1b*	2,95592457	6,98e-06
Myeloma overexpressed 2	*Myeov2*	2,95525738	3,07e-07
Cadherin 17	*Cdh17*	2,95343809	5,76e-07
Pdz and lim domain 2	*Pdlim2*	2,94193504	5,14e-07
Kinesin family member 20a	*Kif20a*	2,94168858	4,29e-06
Sulfiredoxin 1 homolog (s. Cerevisiae)	*Srxn1*	2,94093531	2,63e-06
Clathrin, light polypeptide (lcb)	*Cltb*	2,93727836	1,41e-07
Tumor necrosis factor (ligand) superfamily, member 13b	*Tnfsf13b*	2,93625296	9,27e-07
Tripartite motif-containing 24	*Trim24*	2,93531803	6,59e-07
Histidine triad nucleotide binding protein 3	*Hint3*	2,92981691	1,17e-06
Methyltransferase like 7a1 /// methyltransferase like 7a2	*Mettl7a1 /// mettl7a2*	2,91432958	1,76e-06
Mitogen-activated protein kinase kinase kinase 5	*Map3k5*	2,90721983	1,69e-06
Protocadherin 10	*Pcdh10*	2,89625611	7,84e-05
G protein-coupled receptor 19	*Gpr19*	2,88837043	1,47e-05
Retinoic acid receptor, beta	*Rarb*	2,87197579	2,44e-05
Angiopoietin 2	*Angpt2*	2,86469058	9,22e-08
Lysozyme 2	*Lyz2*	2,86417416	2,69e-06
Glutathione peroxidase 3	*Gpx3*	2,85329516	1,11e-05
Serine (or cysteine) peptidase inhibitor, clade a, member 3n	*Serpina3n*	2,84787122	8,23e-06
Star-related lipid transfer (start) domain containing 6	*Stard6*	2,84581344	9,71e-08
Amino-terminal enhancer of split	*Aes*	2,84202276	0,00171179
Family with sequence similarity 13, member c	*Fam13c*	2,84074793	6,72e-06
Dynamin 3, opposite strand /// microrna 214	*Dnm3os /// mir214*	2,83193069	1,93e-07
Myozenin 2	*Myoz2*	2,83008062	4,76e-07
Brain and acute leukemia, cytoplasmic	*Baalc*	2,82741122	2,69e-07
Rab32, member ras oncogene family	*Rab32*	2,82645274	1,59e-06

Cysteine-rich protein 1 (intestinal)	*Crip1*	2,81277695	2,78e-06
Guanine nucleotide binding protein, alpha 13	*Gna13*	2,81097323	4,91e-07
Family with sequence similarity 101, member b	*Fam101b*	2,81035853	4,26e-07
Synaptotagmin xiii	*Syt13*	2,79397503	2,04e-06
Growth factor receptor bound protein 2-associated protein 3	*Gab3*	2,78851271	1,56e-06
Prostaglandin e receptor 4 (subtype ep4)	*Ptger4*	2,78460759	3,31e-05
Protein kinase inhibitor, alpha	*Pkia*	2,77370021	0,00438091
Death-associated protein	*Dap*	2,77066093	1,36e-07
S100 calcium binding protein a4	*S100a4*	2,76514531	1,90e-07
Aldehyde dehydrogenase 1 family, member l1	*Aldh1l1*	2,76444691	2,59e-07
Hexokinase 1	*Hk1*	2,7609865	0,0003993
Guanine nucleotide binding protein (g protein), alpha inhibiting 1	*Gnai1*	2,75406997	1,01e-05
Neuron navigator 2	*Nav2*	2,74895998	1,63e-05
Chimerin 2	*Chn2*	2,74543399	8,48e-06
Carbonic anhydrase 9	*Car9*	2,7346463	3,04e-05
Serum/glucocorticoid regulated kinase 1	*Sgk1*	2,73375367	1,41e-05
Glutathione s-transferase, mu 2	*Gstm2*	2,72862323	2,19e-07
Lim homeobox protein 6	*Lhx6*	2,72760034	3,47e-05
Microtubule-associated protein, rp/eb family, member 2	*Mapre2*	2,72567256	7,23e-05
Hepatic leukemia factor	*Hlf*	2,72061269	9,50e-05
Predicted gene 13305 /// predicted gene 2002 /// interleukin 11 receptor, alpha chain 1 /// interleukin 11 receptor, alpha chain 2 /// interleukin-11 receptor subunit alpha-2-like	*Gm13305 /// gm2002 /// il11ra1 /// il11ra2 /// loc100861969*	2,70922006	6,12e-07
Htra serine peptidase 3	*Htra3*	2,70785081	4,04e-06
Citrate lyase beta like	*Clybl*	2,70409377	9,54e-05
Calcium channel, voltage-dependent, gamma subunit 6	*Cacng6*	2,70051938	5,18e-06
Zw10 interactor	*Zwint*	2,69137309	1,23e-07
Cytidine deaminase	*Cda*	2,68919926	3,76e-07
Sodium channel, voltage-gated, type ii, alpha 1	*Scn2a1*	2,67958003	5,27e-05
Adp-ribosylation factor gtpase activating protein 3	*Arfgap3*	2,67763872	2,36e-07
Glutathione s-transferase, theta 2	*Gstt2*	2,67684826	3,42e-05
Aspartoacylase	*Aspa*	2,67462108	6,29e-07
Sprouty homolog 2 (drosophila)	*Spry2*	2,67402323	4,60e-06
Acetyl-coenzyme a acyltransferase 2 (mitochondrial 3-oxoacyl-coenzyme a thiolase)	*Acaa2*	2,66426732	8,87e-06
Tripartite motif-containing 54	*Trim54*	2,66241921	4,04e-05
2'-5' oligoadenylate synthetase-like 2	*Oasl2*	2,65109295	0,00036281
Caax box 1c	*Cxx1c*	2,63300255	4,75e-07
Ubiquitin specific peptidase 2	*Usp2*	2,6292211	4,85e-06
Ectopic ossification 1	*Etos1*	2,61370147	2,48e-06
Pituitary tumor-transforming 1 interacting protein	*Pttg1ip*	2,61179899	3,72e-07
Vanin 1	*Vnn1*	2,60959197	8,02e-06
Lysyl oxidase-like 3	*Loxl3*	2,6059592	3,12e-06

Zinc finger (ccch type), rna binding motif and serine/arginine rich 1	*Zrsr1*	2,59856461	2,15e-07
Heparin-binding egf-like growth factor	*Hbegf*	2,59827551	1,34e-06
Solute carrier family 15, member 5	*Slc15a5*	2,59049273	7,02e-05
Nme/nm23 family member 5	*Nme5*	2,58634707	0,0001406
D-aspartate oxidase	*Ddo*	2,58405889	7,48e-06
Cell division cycle 25c	*Cdc25c*	2,58371879	5,35e-07
Diaphanous homolog 1 (drosophila)	*Diap1*	2,57499166	2,77e-07
Nuclear factor i/c	*Nfic*	2,56202292	0,00464296
Ring finger protein 130	*Rnf130*	2,54804268	1,32e-06
Family with sequence similarity 19, member a5	*Fam19a5*	2,54701967	1,36e-07
Start domain containing 8	*Stard8*	2,54625643	1,33e-06
Guanine nucleotide binding protein (g protein), gamma 2	*Gng2*	2,54060994	0,00013146
Ring finger protein 183	*Rnf183*	2,53931384	2,37e-07
G protein-coupled receptor 137b /// g protein-coupled receptor 137b, pseudogene	*Gpr137b /// gpr137b-ps*	2,52744432	1,20e-07
Pregnancy-associated plasma protein a	*Pappa*	2,52567019	1,11e-05
Lps-responsive beige-like anchor	*Lrba*	2,52456969	4,49e-07
Caprin family member 2	*Caprin2*	2,51885921	1,27e-05
Guanylate binding protein 2b	*Gbp2b*	2,50962903	6,20e-07
N-myc downstream regulated gene 3	*Ndrg3*	2,50257473	5,74e-07
Purkinje cell protein 4	*Pcp4*	2,50218694	3,06e-05
Dystrophia myotonica-protein kinase	*Dmpk*	2,50210965	0,00036558
Family with sequence similarity 214, member b	*Fam214b*	2,50208351	7,05e-06
Px domain containing serine/threonine kinase	*Pxk*	2,50067479	2,20e-06
Intraflagellar transport 27	*Ift27*	2,49195083	2,72e-06
Peroxisomal biogenesis factor 3	*Pex3*	2,49193063	0,00042254
Ectonucleoside triphosphate diphosphohydrolase 5	*Entpd5*	2,49157078	6,40e-07
Histidyl-trna synthetase	*Hars*	2,48933413	1,80e-07
Methyltransferase like 7a2	*Mettl7a2*	2,48763584	4,23e-06
Solute carrier family 12, member 5	*Slc12a5*	2,48555327	4,91e-07
Meis homeobox 1	*Meis1*	2,48431668	2,47e-06
Potassium channel, subfamily k, member 13	*Kcnk13*	2,48114186	0,00012829
Fizzy/cell division cycle 20 related 1 (drosophila)	*Fzr1*	2,47907907	1,39e-05
G protein-coupled receptor 137b	*Gpr137b*	2,47865955	1,12e-06
Bcl2-related ovarian killer	*Bok*	2,475733	3,19e-07
Integrin beta 2	*Itgb2*	2,47221716	4,51e-06
Ubiquitin-conjugating enzyme e2g 2	*Ube2g2*	2,47201496	0,00018562
Tetraspanin 15	*Tspan15*	2,4676528	4,43e-07
Ferm domain containing 4b	*Frmd4b*	2,46572004	0,00049508
Utrophin	*Utrn*	2,462692	1,45e-07
Myelin basic protein	*Mbp*	2,46186887	8,46e-07
Cdc23 cell division cycle 23	*Cdc23*	2,46155869	0,00029662
Serine hydrolase-like	*Serhl*	2,45834857	9,05e-05
Tubulin tyrosine ligase-like 1	*Ttll1*	2,45626237	5,81e-06

Adenomatosis polyposis coli	*Apc*	2,45399293	1,21e-05
Riken cdna b230118h07 gene	*B230118h07rik*	2,44835321	8,54e-07
Kh domain containing, rna binding, signal transduction associated 3	*Khdrbs3*	2,44364974	2,34e-06
Vesicle-associated membrane protein 5	*Vamp5*	2,441881	1,40e-06
Rab18, member ras oncogene family	*Rab18*	2,44166221	0,00335377
Nad(p) dependent steroid dehydrogenase-like	*Nsdhl*	2,44066226	2,69e-07
Ferm domain containing 6	*Frmd6*	2,4399466	1,83e-07
Msh homeobox 2	*Msx2*	2,42082106	6,03e-07
Dnaj (hsp40) homolog, subfamily b, member 4	*Dnajb4*	2,42070476	0,00040096
Reticulon 4	*Rtn4*	2,41916658	0,00069779
Integrator complex subunit 6	*Ints6*	2,41716121	4,15e-07
Zinc finger, cchc domain containing 7	*Zcchc7*	2,41564301	2,08e-06
Gap junction protein, alpha 1	*Gja1*	2,40894813	0,0008928
Component of oligomeric golgi complex 1 /// conserved oligomeric golgi complex subunit 1-like	*Cog1 /// loc102641618*	2,40540314	1,71e-06
Hematological and neurological expressed sequence 1	*Hn1*	2,40407012	1,46e-06
Superoxide dismutase 3, extracellular	*Sod3*	2,40387911	2,12e-05
Hepatitis a virus cellular receptor 2	*Havcr2*	2,40316318	2,39e-05
Sorting nexin 2	*Snx2*	2,40216798	5,51e-06
Cdna sequence bc028528	*Bc028528*	2,40081504	3,45e-05
Dual specificity phosphatase 6	*Dusp6*	2,39723338	1,34e-06
Zxd family zinc finger c	*Zxdc*	2,39511181	0,00048171
Triggering receptor expressed on myeloid cells 3	*Trem3*	2,39433008	2,65e-05
Transgelin 3	*Tagln3*	2,39184867	6,42e-06
Fig4 homolog (s. Cerevisiae)	*Fig4*	2,3849217	1,50e-06
Cytochrome c oxidase subunit viia 1	*Cox7a1*	2,37699753	8,93e-05
Amylo-1,6-glucosidase, 4-alpha-glucanotransferase	*Agl*	2,37356007	5,81e-06
Expressed sequence aa408650	*Aa408650*	2,37280948	2,03e-06
Casein kappa	*Csn3*	2,37217654	8,94e-05
Drebrin-like	*Dbnl*	2,36987435	3,09e-05
Map kinase-interacting serine/threonine kinase 2	*Mknk2*	2,36795118	1,10e-05
Complement component 3a receptor 1	*C3ar1*	2,36637508	1,11e-05
Translocase of outer mitochondrial membrane 34	*Tomm34*	2,36452378	2,69e-07
Carbohydrate sulfotransferase 2	*Chst2*	2,36372335	1,98e-05
Echinoderm microtubule associated protein like 6	*Eml6*	2,36265146	6,44e-06
Mannosidase 1, alpha	*Man1a*	2,35863398	2,45e-05
Nucleus accumbens associated 2, ben and btb (poz) domain containing	*Nacc2*	2,35615093	4,00e-06
Ino80 complex subunit c	*Ino80c*	2,34532352	1,74e-06
Kinesin light chain 1	*Klc1*	2,3348809	1,97e-06
Triadin	*Trdn*	2,33210583	1,60e-05
Diaphanous homolog 2 (drosophila)	*Diap2*	2,32991671	1,40e-05
Transducin-like enhancer of split 2, homolog of drosophila e(spl)	*Tle2*	2,32873421	5,64e-06
Ankyrin repeat domain 44	*Ankrd44*	2,3273202	3,00e-06

Dna segment, chr 10, johns hopkins university 81 expressed	*D10jhu81e*	2,32323056	6,03e-07
Methyl-cpg binding domain protein 2	*Mbd2*	2,3207191	6,18e-05
F-box protein 4	*Fbxo4*	2,31675695	1,40e-06
Adenylate kinase 1	*Ak1*	2,31612734	1,32e-06
Sestrin 1	*Sesn1*	2,3130206	8,16e-05
Catenin (cadherin associated protein), alpha-like 1	*Ctnnal1*	2,3128342	0,00027827
Methionine sulfoxide reductase a	*Msra*	2,3127591	4,65e-05
Family with sequence similarity 26, member e	*Fam26e*	2,31259648	7,85e-05
Mannosidase 2, alpha b2	*Man2b2*	2,31230966	0,00028722
Dna replication helicase 2 homolog (yeast)	*Dna2*	2,31066206	8,61e-06
Peroxisomal biogenesis factor 12	*Pex12*	2,30450004	1,60e-05
Lanosterol synthase	*Lss*	2,29942532	2,77e-05
Iq motif containing gtpase activating protein 3	*Iqgap3*	2,2977382	9,93e-07
Small proline-rich protein 1a	*Sprr1a*	2,29550383	1,45e-06
Keratin 15	*Krt15*	2,29428812	6,13e-07
Tripartite motif-containing 2	*Trim2*	2,2934847	5,27e-05
Family with sequence similarity 102, member a	*Fam102a*	2,28978737	1,31e-05
Sorcin	*Sri*	2,28731923	5,69e-07
Dematin actin binding protein	*Dmtn*	2,28348291	1,77e-05
Brain glycogen phosphorylase	*Pygb*	2,28181775	6,47e-07
Adaptor-related protein complex 3, delta 1 subunit	*Ap3d1*	2,27187343	3,37e-06
Serum/glucocorticoid regulated kinase 3	*Sgk3*	2,26995298	0,0040274
Cd99 antigen	*Cd99*	2,2694601	0,00029662
Tripartite motif-containing 12a	*Trim12a*	2,26696023	6,39e-05
Absent in melanoma 1	*Aim1*	2,26582527	9,71e-06
Axin2	*Axin2*	2,26530155	3,83e-05
Collagen, type xviii, alpha 1	*Col18a1*	2,26439469	8,60e-07
Riken cdna 3110002h16 gene	*3110002h16rik*	2,26251912	1,18e-06
Brain-specific angiogenesis inhibitor 1-associated protein 2	*Baiap2*	2,26165368	2,04e-06
Transmembrane and coiled coil domains 3	*Tmcc3*	2,26146761	0,0014497
Guanylate binding protein 2	*Gbp2*	2,26090422	0,00024593
Wd repeat domain 1	*Wdr1*	2,26046763	0,00010181
Phosphoinositide-3-kinase, class 3	*Pik3c3*	2,26039111	3,70e-06
Transformed mouse 3t3 cell double minute 1	*Mdm1*	2,25994736	0,00035815
Inad-like (drosophila)	*Inadl*	2,25847887	6,14e-05
T-box18	*Tbx18*	2,25826596	0,0006818
Transmembrane protein 71	*Tmem71*	2,25531478	1,22e-05
Inhibitor of dna binding 2	*Id2*	2,2539335	3,31e-05
Solute carrier family 37 (glycerol-3-phosphate transporter), member 1	*Slc37a1*	2,25235836	4,67e-06
Slain motif family, member 1	*Slain1*	2,25067213	2,61e-05
Breast carcinoma amplified sequence 3	*Bcas3*	2,2453744	9,16e-05
Potassium channel tetramerisation domain containing 1	*Kctd1*	2,2435253	1,84e-05

Ring finger protein 14	*Rnf14*	2,24332793	0,0046821
Urocortin 2	*Ucn2*	2,24164383	6,07e-05
Mterf domain containing 3	*Mterfd3*	2,24152026	2,37e-06
Proline-rich coiled-coil 1	*Prrc1*	2,24115119	6,43e-06
Bmp and activin membrane-bound inhibitor	*Bambi*	2,23533894	0,00198832
Guanylate binding protein 7	*Gbp7*	2,23532435	2,28e-05
Component of oligomeric golgi complex 1	*Cog1*	2,23228612	5,21e-06
Riken cdna 5430435g22 gene	*5430435g22rik*	2,23061104	4,93e-06
Abhydrolase domain containing 17a	*Abhd17a*	2,22966516	1,80e-05
Prostaglandin i2 (prostacyclin) synthase	*Ptgis*	2,2266828	5,89e-05
Elac homolog 1 (e. Coli)	*Elac1*	2,22602261	0,0001302
Aldehyde dehydrogenase family 7, member a1	*Aldh7a1*	2,22578025	1,59e-06
Testis expressed gene 13	*Tex13*	2,22462541	5,95e-05
Rna binding motif protein, x chromosome	*Rbmx*	2,22461118	1,61e-06
Guanidinoacetate methyltransferase	*Gamt*	2,22422423	1,87e-06
Enoyl coenzyme a hydratase domain containing 1	*Echdc1*	2,22130236	1,70e-05
Rab13, member ras oncogene family	*Rab13*	2,21587047	1,23e-05
Inhibitor of kappab kinase gamma	*Ikbkg*	2,21545211	1,03e-05
Tubulin, beta 3 class iii	*Tubb3*	2,2145971	0,00021518
Vacuolar protein sorting 45 (yeast)	*Vps45*	2,21336725	2,50e-06
Cdna sequence bc031181	*Bc031181*	2,21039217	2,22e-05
Potassium channel, subfamily k, member 2	*Kcnk2*	2,20867466	2,39e-06
Iduronate 2-sulfatase	*Ids*	2,20651993	0,00186513
High-mobility group nucleosome binding domain 5	*Hmgn5*	2,20438878	6,00e-06
Rap guanine nucleotide exchange factor (gef) 3	*Rapgef3*	2,20314589	0,00054829
Ataxin 10	*Atxn10*	2,20010489	1,87e-06
Transcription factor cp2-like 1	*Tfcp2l1*	2,19998438	0,00026222
Pyrimidinergic receptor p2y, g-protein coupled, 6	*P2ry6*	2,19936537	1,93e-06
Myosin, heavy polypeptide 1, skeletal muscle, adult	*Myh1*	2,19395812	0,00011967
Natriuretic peptide type b	*Nppb*	2,19265627	2,89e-06
Dedicator of cytokinesis 9	*Dock9*	2,18899792	2,59e-06
Lectin, mannose-binding, 1	*Lman1*	2,18867025	1,45e-05
Leucine rich repeat (in flii) interacting protein 1	*Lrrfip1*	2,18474241	1,71e-06
Ras association (ralgds/af-6) domain family member 3	*Rassf3*	2,18449884	2,89e-06
Tetraspanin 7	*Tspan7*	2,1840865	2,89e-05
Erythrocyte protein band 4.1-like 1	*Epb4.1l1*	2,1834121	2,48e-06
Hydroxysteroid (17-beta) dehydrogenase 7	*Hsd17b7*	2,18014349	8,64e-06
Atp-binding cassette, sub-family d (ald), member 1	*Abcd1*	2,1793252	0,00014513
F-box protein 16	*Fbxo16*	2,17902065	8,81e-05
Inhibitor of dna binding 1	*Id1*	2,17629811	4,07e-05
Zinc finger e-box binding homeobox 1	*Zeb1*	2,17623552	1,07e-06
Zinc finger, matrin type 2	*Zmat2*	2,17601271	2,10e-06
Bridging integrator 1	*Bin1*	2,17436886	9,59e-07
Adenylate cyclase 9	*Adcy9*	2,1743594	4,15e-05

Inositol 1,4,5-trisphosphate receptor 1	*Itpr1*	2,17398716	0,00022595
Small integral membrane protein 3	*Smim3*	2,17373767	4,10e-07
Adaptor-related protein complex 2, beta 1 subunit	*Ap2b1*	2,1726938	1,68e-05
Leucine-rich repeat-containing g protein-coupled receptor 6	*Lgr6*	2,17156835	3,01e-05
Coiled-coil domain containing 109b	*Ccdc109b*	2,1683645	8,30e-06
Biogenesis of lysosomal organelles complex-1, subunit 1 /// retinol dehydrogenase 5	*Bloc1s1 /// rdh5*	2,16632549	1,13e-05
Bcl2-associated athanogene 2	*Bag2*	2,16240014	9,00e-05
Farnesyl diphosphate synthetase	*Fdps*	2,15800307	3,06e-06
Protein tyrosine phosphatase, receptor type, m	*Ptprm*	2,15108565	1,36e-06
Oaf homolog (drosophila)	*Oaf*	2,15055653	7,19e-07
Special at-rich sequence binding protein 1	*Satb1*	2,14726244	3,04e-06
2-hydroxyacyl-coa lyase 1	*Hacl1*	2,14093006	2,72e-05
Lim-domain containing, protein kinase	*Limk1*	2,14036978	3,92e-05
Vps20-associated 1 homolog (s. Cerevisiae)	*Vta1*	2,13915805	4,00e-06
Phosphofructokinase, muscle	*Pfkm*	2,13582168	5,35e-07
Rho family gtpase 2	*Rnd2*	2,13460329	8,19e-06
Peptidylprolyl isomerase (cyclophilin)-like 4	*Ppil4*	2,13187324	4,93e-06
Transmembrane protein 43	*Tmem43*	2,13142023	2,88e-06
Leucine rich repeat containing 57	*Lrrc57*	2,13131305	1,30e-05
Acyl-coa synthetase short-chain family member 2	*Acss2*	2,12980045	2,85e-06
Rad51 homolog b	*Rad51b*	2,1263448	5,17e-05
Phosphatase and actin regulator 2	*Phactr2*	2,12406581	0,00020156
Small nuclear ribonucleoprotein 25 (u11/u12)	*Snrnp25*	2,12273219	3,39e-06
Fascin homolog 1, actin bundling protein (strongylocentrotus purpuratus)	*Fscn1*	2,12265941	0,00151792
Rna binding motif, single stranded interacting protein 2	*Rbms2*	2,12134277	6,12e-06
Zinc finger, matrin type 5	*Zmat5*	2,12072619	8,49e-06
Transcription factor 4	*Tcf4*	2,11882565	3,50e-05
Breast cancer anti-estrogen resistance 3	*Bcar3*	2,11689582	3,82e-06
Dehydrogenase/reductase (sdr family) member 3	*Dhrs3*	2,11526758	1,57e-05
Rho gtpase activating protein 23	*Arhgap23*	2,11272659	3,28e-06
Kinesin family member 13a	*Kif13a*	2,11117477	1,57e-06
Tropomyosin 2, beta	*Tpm2*	2,11024964	0,00382322
Pleckstrin homology-like domain, family a, member 1	*Phlda1*	2,10484945	5,95e-05
Transmembrane protein 140	*Tmem140*	2,10391051	9,30e-06
Trichohyalin	*Tchh*	2,10244495	2,55e-05
Cytoplasmic fmr1 interacting protein 2	*Cyfip2*	2,10192223	4,07e-05
Pterin 4 alpha carbinolamine dehydratase/dimerization cofactor of hepatocyte nuclear factor 1 alpha (tcf1) 2	*Pcbd2*	2,09990891	3,64e-06
Contactin 6	*Cntn6*	2,09846977	3,43e-05
Wd repeat and fyve domain containing 1	*Wdfy1*	2,09845578	7,92e-06
Dynactin 4	*Dctn4*	2,09362517	0,00029726
Integrin beta 3 binding protein (beta3-endonexin)	*Itgb3bp*	2,09331654	0,00015115
Interleukin 17 receptor c	*Il17rc*	2,09323679	1,59e-05

Riken cdna 2610002m06 gene /// charged multivesicular body protein 1b	*2610002m06rik /// chmp1b*	2,09178123	2,72e-05
Family with sequence similarity 189, member b	*Fam189b*	2,09019885	4,93e-06
Chymotrypsin-like elastase family, member 1	*Cela1*	2,0897211	0,00024476
Spc25, ndc80 kinetochore complex component, homolog (s. Cerevisiae)	*Spc25*	2,08938307	2,69e-05
Thrombospondin 2	*Thbs2*	2,08845624	8,05e-07
Cdp-diacylglycerol synthase (phosphatidate cytidylyltransferase) 2	*Cds2*	2,08556022	1,09e-05
Adenosine deaminase, rna-specific, b1	*Adarb1*	2,08482261	4,28e-05
Cysteine-rich with egf-like domains 1	*Creld1*	2,08438589	6,40e-05
Riken cdna 2010005h15 gene /// predicted gene 5483	*2010005h15rik /// gm5483*	2,08304715	0,00024611
Steroid receptor rna activator 1	*Sra1*	2,0830348	4,18e-06
Riken cdna 2010111i01 gene	*2010111i01rik*	2,08280406	7,56e-07
Monocyte to macrophage differentiation-associated	*Mmd*	2,08173437	8,73e-06
S100 calcium binding protein a1	*S100a1*	2,07874422	9,73e-06
Laminin, gamma 2	*Lamc2*	2,07523039	1,24e-06
Excision repair cross-complementing rodent repair deficiency, complementation group 3	*Ercc3*	2,0750309	7,87e-06
Adaptor-related protein complex 1, sigma 2 subunit	*Ap1s2*	2,0749498	5,04e-05
Glucosidase, alpha; neutral c	*Ganc*	2,0738577	0,00020872
Keratin 19	*Krt19*	2,07216939	7,08e-06
Supervillin	*Svil*	2,0712849	2,44e-06
Suppressor of variegation 3-9 homolog 1 (drosophila)	*Suv39h1*	2,0700919	0,0040523
Gh3 domain containing	*Ghdc*	2,06988223	8,52e-06
Immediate early response 3 interacting protein 1	*Ier3ip1*	2,06958291	3,20e-05
Dihydropyrimidinase-like 3	*Dpysl3*	2,0653754	1,12e-06
Lamin b1	*Lmnb1*	2,06503107	1,42e-05
Serine (or cysteine) peptidase inhibitor, clade b, member 9	*Serpinb9*	2,06383227	5,78e-05
Trafficking protein particle complex 1	*Trappc1*	2,0636294	3,37e-06
Zinc finger homeodomain 4	*Zfhx4*	2,06276804	1,83e-05
Jagged 1	*Jag1*	2,06170026	0,00025432
Fun14 domain containing 2	*Fundc2*	2,06146351	0,00011189
Integrin linked kinase	*Ilk*	2,06118381	0,00212743
Vacuolar protein sorting 25 (yeast)	*Vps25*	2,0602113	0,00029919
Camp responsive element binding protein 3-like 2	*Creb3l2*	2,06017667	3,84e-06
Wingless-type mmtv integration site family, member 4	*Wnt4*	2,05805102	4,48e-06
Homeobox d8	*Hoxd8*	2,0569466	6,33e-06
Cytochrome b5 type a (microsomal)	*Cyb5a*	2,05646598	1,69e-06
Cytochrome p450, family 2, subfamily j, polypeptide 6	*Cyp2j6*	2,05510134	9,86e-05
Epithelial membrane protein 3	*Emp3*	2,05380505	3,82e-06
Rho gtpase activating protein 1	*Arhgap1*	2,05358925	0,00060328
Solute carrier family 31, member 2	*Slc31a2*	2,05226697	8,73e-06
Human immunodeficiency virus type i enhancer binding protein 3	*Hivep3*	2,05202476	8,58e-06

Discs, large homolog 3 (drosophila)	*Dlg3*	2,05123032	2,00e-06
Riken cdna 1810043g02 gene	*1810043g02rik*	2,0488184	4,75e-05
Glutamate-ammonia ligase (glutamine synthetase)	*Glul*	2,04781496	6,14e-05
N-sulfoglucosamine sulfohydrolase (sulfamidase)	*Sgsh*	2,04700859	3,11e-06
Gamma-glutamyltransferase 7	*Ggt7*	2,04653237	2,78e-05
Dual specificity phosphatase 10	*Dusp10*	2,04522323	5,71e-06
Transmembrane protein 19	*Tmem19*	2,04119661	1,69e-05
Homeobox b4	*Hoxb4*	2,04069396	0,00074228
Riken cdna c030006k11 gene	*C030006k11rik*	2,0404248	0,00032809
Myeloid nuclear differentiation antigen like	*Mndal*	2,039439	0,00090306
Sec11 homolog c (s. Cerevisiae)	*Sec11c*	2,03883897	3,31e-05
Abelson helper integration site 1	*Ahi1*	2,03818218	1,78e-05
Single-pass membrane protein with aspartate rich tail 1	*Smdt1*	2,03729639	1,53e-06
Tubulin, beta 2a class iia	*Tubb2a*	2,03696342	1,59e-06
Btb (poz) domain containing 2	*Btbd2*	2,03278492	2,55e-05
Ring finger protein 141	*Rnf141*	2,03011457	0,00016588
Discs, large homolog 5 (drosophila)	*Dlg5*	2,02941793	0,00143944
Rh blood group, d antigen	*Rhd*	2,02904596	1,37e-05
Colony stimulating factor 2 receptor, beta, low-affinity (granulocyte-macrophage)	*Csf2rb*	2,02729002	8,98e-05
Solute carrier family 25 (mitochondrial carrier, dicarboxylate transporter), member 10	*Slc25a10*	2,02690075	2,19e-05
Transmembrane protein 184a	*Tmem184a*	2,02534307	8,26e-05
Ferrochelatase	*Fech*	2,0250255	7,96e-05
Sparc related modular calcium binding 1	*Smoc1*	2,02499161	0,00017179
Low density lipoprotein receptor class a domain containing 4	*Ldlrad4*	2,02329457	0,00010893
Wd repeat domain 33	*Wdr33*	2,02313835	0,00046531
Neurochondrin	*Ncdn*	2,02049767	0,00732446
Transformation related protein 53 inducible nuclear protein 2	*Trp53inp2*	2,01728594	1,88e-05
Sulfite oxidase	*Suox*	2,01505386	1,78e-06
Oxysterol binding protein-like 6	*Osbpl6*	2,01503262	0,00561657
Ribosomal rna processing 1 homolog (s. Cerevisiae)	*Rrp1*	2,01441241	3,04e-05
Ubiquitin specific peptidase 20	*Usp20*	2,01439169	0,00034084
Calcitonin/calcitonin-related polypeptide, alpha	*Calca*	2,01302292	0,0002504
Helicase (dna) b	*Helb*	2,01125506	0,00023024
Taf7 rna polymerase ii, tata box binding protein (tbp)-associated factor	*Taf7*	2,01085688	0,00071665
Tec protein tyrosine kinase	*Tec*	2,00989897	6,22e-05
Zinc finger, nfx1-type containing 1	*Znfx1*	2,00878765	3,61e-05
Cndp dipeptidase 2 (metallopeptidase m20 family)	*Cndp2*	2,00695112	4,33e-06
N-ethylmaleimide sensitive fusion protein attachment protein gamma	*Napg*	2,00386381	2,58e-06
Homeobox c13	*Hoxc13*	2,00279637	1,32e-05
Transformed mouse 3t3 cell double minute 2	*Mdm2*	2,00231965	6,96e-05
Protein tyrosine phosphatase, receptor type, e	*Ptpre*	2,00220628	2,54e-05

Apoptotic peptidase activating factor 1	*Apaf1*	2,00207316	1,26e-05
Swi/snf related, matrix associated, actin dependent regulator of chromatin, subfamily b, member 1	*Smarcb1*	2,00166969	1,05e-05
A disintegrin and metallopeptidase domain 12 (meltrin alpha)	*Adam12*	2,00151693	0,00034898
Rac gtpase-activating protein 1	*Racgap1*	2,00061181	7,35e-06
Polycystic kidney disease 2-like 2	*Pkd2l2*	1,99804079	0,00331437
Ral guanine nucleotide dissociation stimulator,-like 1	*Rgl1*	1,9980263	5,10e-06
Cell division cycle associated 3	*Cdca3*	1,99688373	1,95e-06
Phosphodiesterase 2a, cgmp-stimulated	*Pde2a*	1,99136485	5,98e-05
Syntaxin 7	*Stx7*	1,98903502	4,10e-06
Riken cdna 1110007c09 gene	*1110007c09rik*	1,9885144	1,98e-05
Cap, adenylate cyclase-associated protein, 2 (yeast)	*Cap2*	1,98759836	2,06e-05
Vacuolar protein sorting 53 (yeast)	*Vps53*	1,98565916	0,00021822
Alanyl-trna synthetase domain containing 1	*Aarsd1*	1,98526003	1,93e-05
Ankyrin repeat and ef-hand domain containing 1	*Ankef1*	1,98521112	0,00025226
Spectrin repeat containing, nuclear envelope 1	*Syne1*	1,98469494	2,46e-05
Myotubularin related protein 1	*Mtmr1*	1,98400019	8,71e-07
Sorting nexin 6	*Snx6*	1,98332364	9,41e-05
Mitogen-activated protein kinase kinase kinase 8	*Map3k8*	1,98324949	0,0015479
Glial cell line derived neurotrophic factor family receptor alpha 1	*Gfra1*	1,98207132	0,00487008
Tetratricopeptide repeat domain 39c	*Ttc39c*	1,98172085	5,04e-06
Dep domain containing mtor-interacting protein	*Deptor*	1,98079697	0,00037702
S100 calcium binding protein a13	*S100a13*	1,98074284	6,10e-06
Iq motif and sec7 domain 1	*Iqsec1*	1,97937603	1,85e-05
Ltv1 homolog (s. Cerevisiae)	*Ltv1*	1,97895183	1,31e-05
Ubiquitin-conjugating enzyme e2d 2a	*Ube2d2a*	1,97834756	0,00935618
Solute carrier family 48 (heme transporter), member 1	*Slc48a1*	1,97625402	9,52e-07
Potassium channel, subfamily u, member 1	*Kcnu1*	1,97607769	6,83e-05
Family with sequence similarity 107, member b	*Fam107b*	1,97590473	6,39e-06
Max-like protein x	*Mlx*	1,97569971	1,25e-05
Nestin	*Nes*	1,97477003	2,58e-05
Non-smc condensin ii complex, subunit h2	*Ncaph2*	1,97453914	4,02e-06
Charged multivesicular body protein 4c	*Chmp4c*	1,97369272	0,00015891
Fyve and coiled-coil domain containing 1	*Fyco1*	1,97259088	1,13e-05
Myosin, heavy polypeptide 9, non-muscle	*Myh9*	1,97254682	0,00011485
Protein-l-isoaspartate (d-aspartate) o-methyltransferase 1	*Pcmt1*	1,97174512	2,42e-05
Ras related protein 1b	*Rap1b*	1,97124059	0,00032551
Leucine rich repeat containing 20	*Lrrc20*	1,96948282	4,04e-05
Huntingtin interacting protein 1	*Hip1*	1,96913317	1,48e-05
Potassium channel tetramerisation domain containing 10	*Kctd10*	1,96846598	5,28e-06
Glutaminyl-trna synthase (glutamine-hydrolyzing)-like 1	*Qrsl1*	1,96802013	9,51e-05

Predicted gene 7040 /// prolactin family 2, subfamily c, member 2 /// prolactin family 2, subfamily c, member 3 /// prolactin family 2, subfamily c, member 4 /// prolactin family 2, subfamily c, member 5	*Gm7040 /// prl2c2 /// prl2c3 /// prl2c4 /// prl2c5*	1,96745478	8,56e-05
Proteasome (prosome, macropain) assembly chaperone 2	*Psmg2*	1,96709284	1,33e-05
Synovial sarcoma translocation, chromosome 18	*Ss18*	1,96683909	0,00574606
Eukaryotic translation initiation factor 2d	*Eif2d*	1,96645019	2,18e-05
G protein-coupled receptor, family c, group 5, member b	*Gprc5b*	1,96552599	2,48e-05
Glutathione peroxidase 8 (putative)	*Gpx8*	1,9644943	1,39e-06
Dna segment, chr 10, wayne state university 102, expressed	*D10wsu102e*	1,96385366	1,73e-05
Transaldolase 1	*Taldo1*	1,96281633	2,56e-06
Oncoprotein induced transcript 3	*Oit3*	1,96065627	0,00033648
Brain and reproductive organ-expressed protein	*Bre*	1,95968408	0,00021363
Glucosaminyl (n-acetyl) transferase 3, mucin type	*Gcnt3*	1,95875326	1,33e-05
Bcl2-associated athanogene 3	*Bag3*	1,95717189	2,65e-05
Neoplastic progression 2	*Npn2*	1,95480949	4,92e-05
Inositol (myo)-1(or 4)-monophosphatase 2	*Impa2*	1,95274897	6,90e-06
G two s phase expressed protein 1	*Gtse1*	1,95245989	3,42e-05
Protocadherin gamma subfamily a, 1 /// protocadherin gamma subfamily a, 10 /// protocadherin gamma subfamily a, 11 /// protocadherin gamma subfamily a, 12 /// protocadherin gamma subfamily a, 2 /// protocadherin gamma subfamily a, 3 /// protocadherin gamma subfamily a, 4 /// protocadherin gamma subfamily a, 5 /// protocadherin gamma subfamily a, 6 /// protocadherin gamma subfamily a, 7 /// protocadherin gamma subfamily a, 8 /// protocadherin gamma subfamily a, 9 /// protocadherin gamma subfamily b, 1 /// protocadherin gamma subfamily b, 2 /// protocadherin gamma subfamily b, 4 /// protocadherin gamma subfamily b, 5 /// protocadherin gamma subfamily b, 6 /// protocadherin gamma subfamily b, 7 /// protocadherin gamma subfamily b, 8 /// protocadherin gamma subfamily c, 3 /// protocadherin gamma subfamily c, 4 /// protocadherin gamma subfamily c, 5	*Pcdhga1 /// pcdhga10 /// pcdhga11 /// pcdhga12 /// pcdhga2 /// pcdhga3 /// pcdhga4 /// pcdhga5 /// pcdhga6 /// pcdhga7 /// pcdhga8 /// pcdhga9 /// pcdhgb1 /// pcdhgb2 /// pcdhgb4 /// pcdhgb5 /// pcdhgb6 /// pcdhgb7 /// pcdhgb8 /// pcdhgc3 /// pcdhgc4 /// pcdhgc5*	1,95118241	0,000744
Sorting nexin 12	*Snx12*	1,95022546	6,37e-05
Leucine carboxyl methyltransferase 1	*Lcmt1*	1,94823548	5,03e-06
Baculoviral iap repeat-containing 5	*Birc5*	1,94651539	5,26e-06
Reversion-inducing-cysteine-rich protein with kazal motifs	*Reck*	1,94484114	0,00117776
Plakophilin 1	*Pkp1*	1,9433897	0,00258752
Tyrosine 3-monooxygenase/tryptophan 5-monooxygenase activation protein, zeta polypeptide	*Ywhaz*	1,9420982	1,81e-06
Death-associated protein kinase 3	*Dapk3*	1,94113028	0,00553691
Potassium channel tetramerisation domain containing 2	*Kctd2*	1,93836114	6,48e-06
Dnaj (hsp40) homolog, subfamily c, member 14	*Dnajc14*	1,93800827	6,00e-06
Eph receptor a7	*Epha7*	1,93694036	0,00238751

Lactate dehydrogenase b	*Ldhb*	1,93656808	8,66e-05
Amyloid beta (a4) precursor protein-binding, family b, member 3	*Apbb3*	1,93619414	0,00040787
Bone morphogenetic protein 1	*Bmp1*	1,93478564	0,00034443
Fch and double sh3 domains 1	*Fchsd1*	1,93452423	0,00019962
Adrenergic receptor, beta 2	*Adrb2*	1,93438386	0,001184
Fanconi anemia, complementation group c	*Fancc*	1,93411653	1,35e-05
Myosin, light polypeptide 6, alkali, smooth muscle and non-muscle pseudogene /// myosin light polypeptide 6 alkali smooth muscle and non-muscle protein, pseudogene /// myosin light polypeptide 6-like /// myosin, light polypeptide 6, alkali, smooth muscle and non-muscle	*Gm5526 /// gm8894 /// loc102643184 /// myl6*	1,93342631	3,06e-06
Zinc finger matrin type 3	*Zmat3*	1,93307474	2,48e-05
Glutaminyl-peptide cyclotransferase (glutaminyl cyclase)	*Qpct*	1,93116133	8,26e-05
Mannan-binding lectin serine peptidase 1	*Masp1*	1,92910071	0,00010425
Was protein family, member 1	*Wasf1*	1,92884674	1,98e-05
Coagulation factor viii	*F8*	1,92829518	8,73e-06
Coiled-coil domain containing 93	*Ccdc93*	1,92534005	0,00016082
Tensin 1	*Tns1*	1,92430631	0,00015697
D-tyrosyl-trna deacylase 1	*Dtd1*	1,92404699	4,35e-06
Dynein light chain tctex-type 3	*Dynlt3*	1,92326527	0,00552465
Non-smc condensin i complex, subunit g	*Ncapg*	1,92241446	7,43e-06
Transforming growth factor beta 1 induced transcript 1	*Tgfb1i1*	1,92199589	0,00060122
Migration and invasion enhancer 1	*Mien1*	1,91856566	2,82e-05
Grainyhead-like 1 (drosophila)	*Grhl1*	1,91725263	0,00033453
P53 and dna damage regulated 1	*Pdrg1*	1,91608685	6,94e-06
Mitochondrial gtpase 1 homolog (s. Cerevisiae)	*Mtg1*	1,91587269	1,38e-05
Pq loop repeat containing 1	*Pqlc1*	1,91451351	1,20e-05
Platelet derived growth factor receptor, beta polypeptide	*Pdgfrb*	1,9141167	3,13e-05
Protocadherin alpha 1 /// protocadherin alpha 10 /// protocadherin alpha 11 /// protocadherin alpha 12 /// protocadherin alpha 2 /// protocadherin alpha 3 /// protocadherin alpha 4 /// protocadherin alpha 5 /// protocadherin alpha 6 /// protocadherin alpha 7 /// protocadherin alpha 8 /// protocadherin alpha 9 /// protocadherin alpha subfamily c, 1 /// protocadherin alpha subfamily c, 2	*Pcdha1 /// pcdha10 /// pcdha11 /// pcdha12 /// pcdha2 /// pcdha3 /// pcdha4 /// pcdha5 /// pcdha6 /// pcdha7 /// pcdha8 /// pcdha9 /// pcdhac1 /// pcdhac2*	1,91383703	1,63e-05
Caspase 12	*Casp12*	1,91334526	5,47e-06
Na+/k+ transporting atpase interacting 1	*Nkain1*	1,91324442	5,20e-06
Short stature homeobox 2	*Shox2*	1,9130595	2,48e-05
Centrosomal protein 112	*Cep112*	1,911885	1,77e-05
Stimulated by retinoic acid 13	*Stra13*	1,91083924	4,92e-05
Zinc finger protein 758	*Zfp758*	1,90899784	0,00206984
Receptor (calcitonin) activity modifying protein 2 /// vacuolar protein sorting 25 (yeast)	*Ramp2 /// vps25*	1,90846557	2,59e-06
Malic enzyme 2, nad(+)-dependent, mitochondrial	*Me2*	1,90740081	5,04e-06

Ankyrin repeat and socs box-containing 13	*Asb13*	1,90738024	0,00239436
Diaphanous homolog 3 (drosophila)	*Diap3*	1,90636967	9,06e-05
Synapse associated protein 1	*Syap1*	1,90587223	1,32e-05
Katanin p60 (atpase-containing) subunit a1	*Katna1*	1,90457622	2,48e-06
Protein tyrosine phosphatase, receptor type, a	*Ptpra*	1,90363994	1,24e-05
Ddb1 and cul4 associated factor 11	*Dcaf11*	1,90282752	0,00018727
Histocompatibility 13	*H13*	1,90221516	7,85e-06
Aldo-keto reductase family 1, member c12 /// aldo-keto reductase family 1, member c13	*Akr1c12 /// akr1c13*	1,90197454	0,00034139
Ras suppressor protein 1	*Rsu1*	1,90184914	1,15e-05
N(alpha)-acetyltransferase 10, nata catalytic subunit	*Naa10*	1,89889751	1,46e-05
Histone deacetylase 5	*Hdac5*	1,8982923	2,48e-05
Salvador homolog 1 (drosophila)	*Sav1*	1,89731526	0,00169417
N-myristoyltransferase 1	*Nmt1*	1,89637924	3,18e-06
Gats protein-like 2	*Gatsl2*	1,89626772	1,73e-05
Aldo-keto reductase family 1, member c13	*Akr1c13*	1,89625713	0,00014853
Dnaj (hsp40) homolog, subfamily c, member 9	*Dnajc9*	1,8961265	2,71e-06
Imp2 inner mitochondrial membrane peptidase-like (s. Cerevisiae)	*Immp2l*	1,89600848	2,34e-05
Aryl-hydrocarbon receptor	*Ahr*	1,89560469	0,00150161
Cysteine-rich c-terminal 1	*Crct1*	1,89477507	1,83e-05
Rab3a interacting protein	*Rab3ip*	1,89443804	1,64e-05
Phospholipase a2, group iie	*Pla2g2e*	1,89432391	1,27e-05
Niemann-pick type c1	*Npc1*	1,89420571	8,69e-06
Riken cdna 1700020i14 gene	*1700020i14rik*	1,89412373	5,29e-05
Dnaj (hsp40) homolog, subfamily b, member 5	*Dnajb5*	1,89320855	0,00012758
Myosin, light polypeptide 4	*Myl4*	1,89308278	0,00015881
Elongation factor 1 homolog (elf1, s. Cerevisiae)	*Elof1*	1,89179163	1,18e-05
Thioredoxin-like 1	*Txnl1*	1,89161045	1,65e-05
Maturin, neural progenitor differentiation regulator homolog (xenopus)	*Mturn*	1,89066538	0,0001208
Ring finger protein 144b	*Rnf144b*	1,88761668	3,66e-06
Proline/serine-rich coiled-coil 1	*Psrc1*	1,88736751	2,00e-05
Seh1-like (s. Cerevisiae	*Seh1l*	1,88718736	9,94e-05
Gtp cyclohydrolase 1	*Gch1*	1,88706501	5,87e-05
Early b cell factor 3	*Ebf3*	1,88673881	0,00536257
Predicted gene 5553 /// orm1-like 2 (s. Cerevisiae)	*Gm5553 /// ormdl2*	1,88389169	2,30e-05
Microsomal glutathione s-transferase 3	*Mgst3*	1,88354425	2,25e-05
Thiopurine methyltransferase	*Tpmt*	1,88248621	9,95e-05
Ngfi-a binding protein 2	*Nab2*	1,8810258	0,00131953
Endosulfine alpha	*Ensa*	1,88055841	4,90e-05
Phosphatidylinositol-4-phosphate 5-kinase, type 1 gamma	*Pip5k1c*	1,8803225	0,00039835
Dymeclin	*Dym*	1,87878734	1,16e-05
Chromodomain helicase dna binding protein 1-like	*Chd1l*	1,87763681	5,61e-05
Guanosine diphosphate (gdp) dissociation inhibitor 1	*Gdi1*	1,87762762	8,10e-05

Inositol polyphosphate-1-phosphatase	*Inpp1*	1,87757532	1,63e-05
Calcium/calmodulin-dependent protein kinase ii, delta	*Camk2d*	1,87730293	0,00018618
Cytochrome c oxidase assembly protein 16	*Cox16*	1,87702808	1,32e-05
Predicted gene 12693 /// rwd domain containing 1	*Gm12693 /// rwdd1*	1,87639045	0,00066046
Tetratricopeptide repeat domain 1	*Ttc1*	1,8762646	1,36e-05
Twist basic helix-loop-helix transcription factor 2	*Twist2*	1,8726078	4,21e-05
Cytoplasmic polyadenylation element binding protein 1	*Cpeb1*	1,87226731	0,00020268
Adaptor-related protein complex 2, alpha 2 subunit	*Ap2a2*	1,8721288	0,00016498
Dna primase, p49 subunit	*Prim1*	1,87166063	9,56e-06
Zinc finger protein 931	*Zfp931*	1,87160947	0,00033395
Mitochondrial ribosomal protein s16	*Mrps16*	1,87073223	6,62e-06
Mediator complex subunit 30	*Med30*	1,87037669	1,01e-05
Lim domain and actin binding 1	*Lima1*	1,86980875	8,67e-06
Chibby homolog 1 (drosophila)	*Cby1*	1,86929054	2,55e-05
Angiopoietin 1	*Angpt1*	1,86749783	0,0004692
Cdna sequence bc005624	*Bc005624*	1,86749158	0,00019819
Collagen, type vii, alpha 1	*Col7a1*	1,86664943	0,00042024
Proteasome (prosome, macropain) inhibitor subunit 1	*Psmf1*	1,86592579	5,85e-06
Malignant t cell amplified sequence 1	*Mcts1*	1,8659064	3,18e-06
Ganglioside-induced differentiation-associated protein 1-like 1	*Gdap1l1*	1,86588194	0,0007816
S-antigen, retina and pineal gland (arrestin)	*Sag*	1,86554945	0,00029511
Centrin 3	*Cetn3*	1,86547948	3,60e-06
Hect, uba and wwe domain containing 1	*Huwe1*	1,86298582	1,78e-05
Nicalin homolog (zebrafish)	*Ncln*	1,86243725	0,00043986
Signal transducer and activator of transcription 1	*Stat1*	1,86206256	0,00028404
Sirtuin 7	*Sirt7*	1,86174594	2,06e-05
Atpase, class vi, type 11c	*Atp11c*	1,86072715	0,00515454
Zinc finger, an1 type domain 2b	*Zfand2b*	1,85854472	0,00018742
Guanylate cyclase activator 1b	*Guca1b*	1,85808293	4,86e-05
Integrator complex subunit 3	*Ints3*	1,85752731	0,00072372
Component of oligomeric golgi complex 6	*Cog6*	1,85493192	6,77e-06
Mediator complex subunit 14	*Med14*	1,85479715	0,00051561
Parathymosin	*Ptms*	1,85476661	0,0036531
5',3'-nucleotidase, cytosolic	*Nt5c*	1,8544462	2,21e-05
Phosphodiesterase 3a, cgmp inhibited	*Pde3a*	1,85310838	9,29e-05
Arfgap with sh3 domain, ankyrin repeat and ph domain1	*Asap1*	1,85289042	2,37e-06
Prefoldin 1	*Pfdn1*	1,8525045	1,48e-05
Riken cdna 0610010b08 gene /// predicted gene 14295 /// predicted gene 14305 /// predicted gene 14308 /// predicted gene 14419 /// predicted gene 14430 /// predicted gene 14432 /// predicted gene 14434 /// predicted gene 2004 /// predicted gene 4724	*0610010b08rik /// gm14295 /// gm14305 /// gm14308 /// gm14419 /// gm14430 /// gm14432 ///*	1,85161862	0,00056186

	gm14434 /// gm2004 /// gm4724		
Hydroxysteroid (17-beta) dehydrogenase 4	*Hsd17b4*	1,85133946	5,52e-05
Sterol regulatory element binding factor 2	*Srebf2*	1,85026046	1,87e-05
Sec63-like (s. Cerevisiae)	*Sec63*	1,84978738	8,73e-06
Casein kinase 1, gamma 2	*Csnk1g2*	1,84976683	2,39e-05
Downstream of stk11	*Dos*	1,84936092	0,00151327
Mitochondrial ribosomal protein s25	*Mrps25*	1,84920861	2,20e-05
Myosin xviiia	*Myo18a*	1,84875746	0,00017742
Glyoxalase domain containing 4	*Glod4*	1,84721949	1,15e-05
Rab2b, member ras oncogene family	*Rab2b*	1,84714233	0,00023531
Lysophosphatidylcholine acyltransferase 1	*Lpcat1*	1,84710747	0,00147883
Pq loop repeat containing	*Pqlc3*	1,84657218	4,04e-05
Threonyl-trna synthetase 2, mitochondrial (putative)	*Tars2*	1,84610115	2,13e-05
Pituitary tumor-transforming gene 1	*Pttg1*	1,84590891	5,94e-06
Succinyl-coa glutarate-coa transferase	*Sugct*	1,84511891	1,36e-05
Spermatogenesis associated glutamate (e)-rich protein 4a	*Speer4a*	1,84476272	0,00695992
Riken cdna 1700037h04 gene	*1700037h04rik*	1,84400001	0,00515454
Fyve, rhogef and ph domain containing 6	*Fgd6*	1,84174742	0,00066336
Cortactin	*Cttn*	1,84115259	0,000649
Smad family member 2	*Smad2*	1,84079729	0,00020133
Phosphatidylserine decarboxylase, pseudogene 3	*Pisd-ps3*	1,84052153	7,88e-06
Alveolar soft part sarcoma chromosome region, candidate 1 (human)	*Aspscr1*	1,83996215	8,61e-06
G protein-coupled receptor kinase 4	*Grk4*	1,83894789	4,60e-05
Anaphase promoting complex subunit 16	*Anapc16*	1,8387825	7,31e-06
Colony stimulating factor 2 receptor, beta 2, low-affinity (granulocyte-macrophage)	*Csf2rb2*	1,83785952	0,00053599
Phosphorylase kinase alpha 2	*Phka2*	1,83715375	7,58e-06
Sh3-binding domain glutamic acid-rich protein like	*Sh3bgrl*	1,83694512	0,00349371
Rio kinase 3	*Riok3*	1,83418263	0,00011878
Erythrocyte protein band 4.1-like 4a	*Epb4.1l4a*	1,83416841	2,43e-05
Tubulin, alpha 4a	*Tuba4a*	1,83139569	3,51e-05
Protein inhibitor of activated stat 3	*Pias3*	1,83001686	1,92e-05
Solute carrier family 25, member 51	*Slc25a51*	1,82988065	0,00695315
Phosphatidylserine decarboxylase, pseudogene 1 /// phosphatidylserine decarboxylase, pseudogene 3	*Pisd-ps1 /// pisd-ps3*	1,82973128	1,47e-05
Dnaj (hsp40) homolog, subfamily c, member 19 /// dnaj (hsp40) homolog, subfamily c, member 19, pseudogene	*Dnajc19 /// dnajc19-ps*	1,82856212	4,02e-05
Dnaj (hsp40) homolog, subfamily b, member 6	*Dnajb6*	1,82681592	0,00011703
Thymopoietin	*Tmpo*	1,82636381	0,00751075
Sorbitol dehydrogenase	*Sord*	1,82474738	4,08e-05
Casp8 and fadd-like apoptosis regulator	*Cflar*	1,82443874	8,91e-06
Methionine sulfoxide reductase b1	*Msrb1*	1,82362574	0,00784485
Smad family member 7	*Smad7*	1,82360368	0,00095714
Dual specificity phosphatase 1	*Dusp1*	1,82351571	0,0012097

Electron transferring flavoprotein, dehydrogenase	*Etfdh*	1,82228244	0,0002593
Peroxisomal biogenesis factor 7	*Pex7*	1,82022414	9,45e-05
Mediator complex subunit 27	*Med27*	1,81996208	3,85e-05
Meiosis 1 associated protein	*M1ap*	1,81915672	8,32e-05
Trafficking protein particle complex 2	*Trappc2*	1,8184565	0,00087461
Parvin, alpha	*Parva*	1,81841995	2,56e-05
Formin binding protein 1	*Fnbp1*	1,81801523	9,89e-06
Zeta-chain (tcr) associated protein kinase	*Zap70*	1,81715753	3,58e-05
Regulator of cell cycle	*Rgcc*	1,81609011	0,00067056
Phosphatidylinositol transfer protein, alpha	*Pitpna*	1,81554958	5,69e-05
Oxysterol binding protein-like 1a	*Osbpl1a*	1,81551798	3,35e-05
Riken cdna 3830403n18 gene /// x-linked lymphocyte-regulated	*3830403n18rik /// xlr*	1,81387816	7,12e-05
Tetraspanin 5	*Tspan5*	1,81323641	7,96e-05
Sorting nexin 3	*Snx3*	1,81305455	6,77e-06
Nitrogen permease regulator-like 3	*Nprl3*	1,81292424	4,25e-05
Adp-ribosylation factor-like 6 interacting protein 1	*Arl6ip1*	1,8126088	1,41e-05
Dual specificity phosphatase 19	*Dusp19*	1,81220547	0,00018329
Ubiquitin protein ligase e3b	*Ube3b*	1,8114149	4,33e-05
Keratin 23	*Krt23*	1,81123157	8,35e-05
Histidyl-trna synthetase 2, mitochondrial (putative)	*Hars2*	1,80996624	7,15e-05
Farnesyl diphosphate farnesyl transferase 1	*Fdft1*	1,80863395	6,20e-06
Polymerase (dna directed), mu	*Polm*	1,80751813	0,00038779
Rho gtpase activating protein 17	*Arhgap17*	1,80621546	4,26e-05
Integrin beta 1 binding protein 1	*Itgb1bp1*	1,80572206	1,78e-05
Atpase, class ii, type 9b	*Atp9b*	1,8045927	9,75e-06
Runt-related transcription factor 1; translocated to, 1 (cyclin d-related)	*Runx1t1*	1,80384765	0,00241931
Solute carrier family 12, member 6	*Slc12a6*	1,80373352	1,74e-05
Mesenteric estrogen dependent adipogenesis	*Medag*	1,8029394	0,0001208
Run and sh3 domain containing 2	*Rusc2*	1,80070581	5,11e-05
Rab3b, member ras oncogene family	*Rab3b*	1,80011976	1,82e-05
Vesicle-associated membrane protein, associated protein b and c	*Vapb*	1,80002782	0,00321267
Purinergic receptor p2x, ligand-gated ion channel, 5	*P2rx5*	1,79947721	0,00012379
Zinc finger and btb domain containing 20	*Zbtb20*	1,79710012	0,00464988
Solute carrier family 12, member 2	*Slc12a2*	1,79650208	1,02e-05
Growth arrest and dna-damage-inducible 45 gamma	*Gadd45g*	1,79628533	5,25e-05
Transmembrane protein 45a	*Tmem45a*	1,79620404	0,00017721
D site albumin promoter binding protein	*Dbp*	1,79613277	7,81e-05
Dnaj (hsp40) homolog, subfamily c, member 18	*Dnajc18*	1,79455681	7,88e-05
Tetraspanin 9	*Tspan9*	1,79428963	2,72e-05
Solute carrier family 39 (zinc transporter), member 3	*Slc39a3*	1,79391584	3,95e-05
Latexin	*Lxn*	1,79385296	6,57e-06
Family with sequence similarity 50, member a	*Fam50a*	1,79235113	1,34e-05
Interleukin-1 receptor-associated kinase 1	*Irak1*	1,79093832	1,51e-05

Ras association (ralgds/af-6) domain family (n-terminal) member 8	*Rassf8*	1,79012335	6,42e-06
Wd repeat containing planar cell polarity effector	*Wdpcp*	1,78963045	0,00061828
Adenosine deaminase, trna-specific 3 /// secretory carrier membrane protein 4	*Adat3 /// scamp4*	1,78928035	0,00017552
Ik cytokine	*Ik*	1,78853785	6,79e-06
Methylcrotonoyl-coenzyme a carboxylase 1 (alpha)	*Mccc1*	1,78772656	4,05e-05
Chloride channel 4-2	*Clcn4-2*	1,78747127	6,89e-05
Endoplasmic reticulum chaperone sil1 homolog (s. Cerevisiae)	*Sil1*	1,78694168	7,04e-06
Creatine kinase, brain	*Ckb*	1,7857676	2,33e-05
Ral guanine nucleotide dissociation stimulator-like 3	*Rgl3*	1,78493322	0,00028429
Riken cdna 1110058l19 gene	*1110058l19rik*	1,78485378	5,78e-05
Adenosine deaminase, trna-specific 2	*Adat2*	1,78377998	0,00017987
Camp responsive element binding protein 3	*Creb3*	1,78372086	1,84e-05
Polymerase (rna) i polypeptide e	*Polr1e*	1,7832969	0,00013751
Methionine aminopeptidase 2	*Metap2*	1,78295602	0,00138414
Riken cdna 3110057o12 gene /// predicted gene 2011	*3110057o12rik /// gm2011*	1,78288694	0,0001262
Elk3, member of ets oncogene family	*Elk3*	1,7825918	3,04e-05
Leukotriene a4 hydrolase	*Lta4h*	1,78214818	7,87e-06
Myosin x	*Myo10*	1,78190646	5,47e-05
Rad51 homolog c	*Rad51c*	1,78130428	0,0022653
Elongator acetyltransferase complex subunit 2	*Elp2*	1,78086404	1,31e-05
Nucleolar and spindle associated protein 1	*Nusap1*	1,7797091	6,43e-05
Nedd4 family interacting protein 1	*Ndfip1*	1,77956645	0,0012203
Retinoic acid induced 14	*Rai14*	1,77920191	0,00039006
Activating signal cointegrator 1 complex subunit 2	*Ascc2*	1,77861152	3,36e-05
Solute carrier family 25, member 46	*Slc25a46*	1,77853995	0,0016517
Small proline-rich protein 2i	*Sprr2i*	1,77788001	0,00150101
Peptidylglycine alpha-amidating monooxygenase	*Pam*	1,77756572	0,00142281
Myosin, light polypeptide 6, alkali, smooth muscle and non-muscle pseudogene /// myosin light polypeptide 6 alkali smooth muscle and non-muscle protein, pseudogene /// myosin, light polypeptide 6, alkali, smooth muscle and non-muscle	*Gm5526 /// gm8894 /// myl6*	1,77693894	1,64e-05
Aha1, activator of heat shock protein atpase 2	*Ahsa2*	1,7762981	2,48e-05
Predicted gene 4887 /// twinfilin, actin-binding protein, homolog 1 (drosophila)	*Gm4887 /// twf1*	1,77610078	0,00947341
Interleukin 6 receptor, alpha	*Il6ra*	1,77563699	0,00032806
Nitric oxide synthase 1, neuronal	*Nos1*	1,77558992	0,00035435
Rho gdp dissociation inhibitor (gdi) alpha	*Arhgdia*	1,77546538	0,00104054
Solute carrier family 4 (anion exchanger), member 8	*Slc4a8*	1,77524386	0,0001915
Nuclear receptor subfamily 3, group c, member 1	*Nr3c1*	1,77515884	0,00015625
Ets variant 5	*Etv5*	1,77432111	0,00010353
Myosin ic	*Myo1c*	1,7730764	0,00037248
Jerky	*Jrk*	1,77287922	9,57e-06
Solute carrier family 46, member 1	*Slc46a1*	1,77282336	0,00110602

Adenylate cyclase 7	*Adcy7*	1,77241731	0,00023466
Hydroxyacid oxidase 1, liver	*Hao1*	1,7723014	0,00391993
Crystallin, zeta	*Cryz*	1,77138222	5,20e-06
Zinc finger protein 759	*Zfp759*	1,76982235	0,00054019
Casp2 and ripk1 domain containing adaptor with death domain	*Cradd*	1,76792311	0,00120206
Nuclear factor of kappa light polypeptide gene enhancer in b cells inhibitor, alpha	*Nfkbia*	1,7676123	0,00011443
Arginine/serine-rich coiled-coil 1	*Rsrc1*	1,76757407	0,00036328
Atpase, class v, type 10a	*Atp10a*	1,76703452	0,00419233
Protocadherin alpha 1 /// protocadherin alpha 10 /// protocadherin alpha 11 /// protocadherin alpha 12 /// protocadherin alpha 2 /// protocadherin alpha 4 /// protocadherin alpha 9 /// protocadherin alpha subfamily c, 1 /// protocadherin alpha subfamily c, 2	*Pcdha1 /// pcdha10 /// pcdha11 /// pcdha12 /// pcdha2 /// pcdha4 /// pcdha9 /// pcdhac1 /// pcdhac2*	1,76693946	0,00079675
Gamma-aminobutyric acid (gaba) a receptor-associated protein-like 1	*Gabarapl1*	1,76656963	1,60e-05
Mitogen-activated protein kinase kinase 2	*Map2k2*	1,76458771	0,00023654
Interferon-induced protein 35	*Ifi35*	1,764183	2,87e-05
Mitochondrial calcium uptake 1	*Micu1*	1,76392486	2,77e-05
Armadillo repeat containing, x-linked 5	*Armcx5*	1,76309948	0,00285018
Trna methyltransferase 1 like	*Trmt1l*	1,76293014	0,00010304
Yip1 domain family, member 5	*Yipf5*	1,76292751	0,0003164
Solute carrier family 29 (nucleoside transporters), member 3	*Slc29a3*	1,762762	1,50e-05
High mobility group nucleosomal binding domain 3	*Hmgn3*	1,76222267	9,87e-05
Polymerase (dna directed), kappa	*Polk*	1,76176311	0,00150659
Protocadherin beta 17	*Pcdhb17*	1,76136775	0,00032337
Testis expressed gene 261	*Tex261*	1,76105396	4,04e-05
Nuclear receptor subfamily 2, group c, member 1	*Nr2c1*	1,76097366	0,00025641
Transmembrane bax inhibitor motif containing 4	*Tmbim4*	1,76061323	4,61e-05
Required for meiotic nuclear division 1 pseudogene /// required for meiotic nuclear division 1 homolog (s. Cerevisiae)	*Gm5512 /// rmnd1*	1,76039741	3,04e-05
Fun14 domain containing 1	*Fundc1*	1,76011512	0,00122633
Choline kinase beta	*Chkb*	1,75974853	7,29e-06
Wd repeat domain 36	*Wdr36*	1,75893837	3,80e-05
Pleckstrin homology-like domain, family a, member 3	*Phlda3*	1,75782228	0,00012829
Lectin, galactose binding, soluble 8	*Lgals8*	1,75753954	0,00513337
Gata binding protein 4	*Gata4*	1,75738852	0,00031561
Family with sequence similarity 114, member a1	*Fam114a1*	1,75676555	2,12e-05
Myosin, light polypeptide 9, regulatory	*Myl9*	1,75497514	0,00016812
Biogenesis of lysosomal organelles complex-1, subunit 5, muted	*Bloc1s5*	1,75442815	5,89e-05
Glucosamine-6-phosphate deaminase 1	*Gnpda1*	1,75384537	1,89e-05
Phosphatidylinositol 4-kinase, catalytic, alpha polypeptide	*Pi4ka*	1,75344987	8,19e-06
Praja ring finger 1, e3 ubiquitin protein ligase	*Pja1*	1,75146946	0,00146874

Sh3-domain grb2-like 3	*Sh3gl3*	1,75049819	0,00556211
Transcription factor ap-2, alpha	*Tfap2a*	1,75013133	0,00034711
Nadh dehydrogenase (ubiquinone) 1 beta subcomplex, 7	*Ndufb7*	1,74817665	6,66e-05
Suppressor of cytokine signaling 1	*Socs1*	1,74815945	6,03e-05
Pdz domain containing ring finger 3	*Pdzrn3*	1,74761317	0,00031747
Hyaluronoglucosaminidase 1 /// n-acetyltransferase 6	*Hyal1 /// nat6*	1,7473769	5,45e-05
Isy1 splicing factor homolog (s. Cerevisiae)	*Isy1*	1,74554222	0,00030919
Transformed mouse 3t3 cell double minute 4	*Mdm4*	1,74423021	2,76e-05
Solute carrier family 39 (metal ion transporter), member 6	*Slc39a6*	1,74343096	0,00047505
Cask-interacting protein 2	*Caskin2*	1,74230298	5,87e-05
Regulation of nuclear pre-mrna domain containing 1a	*Rprd1a*	1,74197872	0,00219843
Protein tyrosine phosphatase, receptor type, t	*Ptprt*	1,74091041	0,00024242
Forkhead box p2	*Foxp2*	1,74037453	0,00108249
Fms-like tyrosine kinase 3 ligand /// ribosomal protein l13a	*Flt3l /// rpl13a*	1,73995106	0,00161043
Retinoic acid induced 2	*Rai2*	1,73981285	0,00025229
Uncharacterized loc100135765 /// phosphatidylinositol glycan anchor biosynthesis, class b	*Loc100135765 /// pigb*	1,73898722	0,00034072
Mckusick-kaufman syndrome	*Mkks*	1,73863891	4,00e-05
Myeloid/lymphoid or mixed-lineage leukemia (trithorax homolog, drosophila); translocated to, 3	*Mllt3*	1,73763648	4,41e-05
Ankyrin repeat and kh domain containing 1	*Ankhd1*	1,73734362	2,61e-05
Apolipoprotein o-like	*Apool*	1,73729062	2,47e-05
Family with sequence similarity 207, member a	*Fam207a*	1,73629107	7,05e-05
Ddb1 and cul4 associated factor 4	*Dcaf4*	1,73493898	0,00026611
Haus augmin-like complex, subunit 1	*Haus1*	1,73487307	2,29e-05
Riken cdna 6430548m08 gene	*6430548m08rik*	1,73455507	1,72e-05
Target of myb1-like 1 (chicken)	*Tom1l1*	1,73440119	0,00020704
Ras and rab interactor 2	*Rin2*	1,7343599	6,87e-05
Treacher collins franceschetti syndrome 1, homolog	*Tcof1*	1,73316505	0,00034995
Protein sfi1 homolog /// sfi1 homolog, spindle assembly associated (yeast)	*Loc100861749 /// sfi1*	1,73306652	6,61e-05
Asparagine-linked glycosylation 12 (alpha-1,6-mannosyltransferase)	*Alg12*	1,73287127	0,00027676
Zinc finger swim-type containing 7	*Zswim7*	1,73228176	7,76e-05
Vesicle transport through interaction with t-snares 1b	*Vti1b*	1,73150931	2,42e-05
Protein arginine n-methyltransferase 2	*Prmt2*	1,73148238	7,40e-05
Enolase 3, beta muscle	*Eno3*	1,7308036	6,23e-05
Aminolevulinic acid synthase 1	*Alas1*	1,72976645	2,38e-05
Fc fragment of igg binding protein	*Fcgbp*	1,72965123	0,00078875
Trafficking protein particle complex subunit 10-like /// trafficking protein particle complex 10	*Loc102641872 /// trappc10*	1,7294913	1,60e-05
Ral gef with ph domain and sh3 binding motif 2	*Ralgps2*	1,72929749	0,00265141
Acyl-coenzyme a oxidase 3, pristanoyl	*Acox3*	1,72926216	0,00086747
Protease-associated domain containing 1	*Pradc1*	1,7292047	4,51e-05
Max dimerization protein 4	*Mxd4*	1,72895637	9,04e-05

Male-specific lethal 1 homolog (drosophila)	*Msl1*	1,72839701	0,00012121
Riken cdna c920025e04 gene /// histocompatibility 2, t region locus 23 /// h-2 class i histocompatibility antigen, d-37 alpha chain-like	*C920025e04rik /// h2-t23 /// loc102641046*	1,72729659	0,00018131
Coatomer protein complex, subunit zeta 1	*Copz1*	1,72711367	6,78e-06
Negative elongation factor complex member c/d, th1l	*Nelfcd*	1,72668711	1,50e-05
Proline rich 14	*Prr14*	1,72569114	4,65e-05
Ankyrin repeat domain 6	*Ankrd6*	1,72566827	0,00351512
Patatin-like phospholipase domain containing 7	*Pnpla7*	1,72548378	0,00017049
Tubulin, beta 6 class v	*Tubb6*	1,72410289	1,31e-05
Inositol polyphosphate 5-phosphatase k	*Inpp5k*	1,72327957	2,21e-05
Pre b cell leukemia homeobox 3	*Pbx3*	1,72316396	1,09e-05
Tumor necrosis factor receptor superfamily, member 21	*Tnfrsf21*	1,72237017	0,00014688
Solute carrier family 35, member a4	*Slc35a4*	1,72227899	0,00027158
Glutathione s-transferase, mu 5	*Gstm5*	1,72161491	8,55e-05
3'-phosphoadenosine 5'-phosphosulfate synthase 1	*Papss1*	1,72069677	4,77e-05
Solute carrier organic anion transporter family, member 5a1	*Slco5a1*	1,720394	6,26e-05
Stomatin	*Stom*	1,7201485	0,00996776
Special at-rich sequence binding protein 2	*Satb2*	1,72007837	9,87e-05
Tissue factor pathway inhibitor	*Tfpi*	1,71978791	0,00897341
Kinesin family member 16b	*Kif16b*	1,71598691	0,00573541
Las1-like (s. Cerevisiae)	*Las1l*	1,71589803	2,55e-05
Atp synthase, h+ transporting, mitochondrial f1 complex, delta subunit	*Atp5d*	1,71523023	2,76e-05
Gem (nuclear organelle) associated protein 8	*Gemin8*	1,71483243	0,00051068
Nadh dehydrogenase (ubiquinone) fe-s protein 7	*Ndufs7*	1,71410635	4,88e-05
Pyruvate dehydrogenase kinase, isoenzyme 4	*Pdk4*	1,7138099	6,27e-05
Centrin 2	*Cetn2*	1,71281911	4,66e-05
Budding uninhibited by benzimidazoles 1 homolog, beta (s. Cerevisiae)	*Bub1b*	1,71131535	4,04e-05
Nuclear factor of activated t cells, cytoplasmic, calcineurin dependent 1	*Nfatc1*	1,71090006	0,00013443
Adenomatosis polyposis coli down-regulated 1	*Apcdd1*	1,7106333	0,00759188
Cxxc finger 1 (phd domain)	*Cxxc1*	1,71053846	2,55e-05
T cell leukemia translocation altered gene	*Tcta*	1,71028894	1,96e-05
Actin related protein 2/3 complex, subunit 4	*Arpc4*	1,70867373	8,72e-05
Non-smc condensin i complex, subunit d2	*Ncapd2*	1,70822064	8,35e-05
Delta/notch-like egf-related receptor	*Dner*	1,70763236	0,0074881
Forkhead box n1	*Foxn1*	1,70699841	0,00024476
Predicted gene 14277 /// small nuclear ribonucleoprotein d1	*Gm14277 /// snrpd1*	1,70695393	1,09e-05
Chondroitin polymerizing factor 2	*Chpf2*	1,70685362	0,00085073
Oral-facial-digital syndrome 1 gene homolog (human)	*Ofd1*	1,70666865	0,0010494
Udp-glucose pyrophosphorylase 2	*Ugp2*	1,70646641	3,07e-05
Sec16 homolog b (s. Cerevisiae)	*Sec16b*	1,70623542	0,00045761
Cellular repressor of e1a-stimulated genes 1	*Creg1*	1,70588116	0,00049409

Cyclin b2	*Ccnb2*	1,70580097	2,50e-05
Coiled-coil domain containing 71 like	*Ccdc71l*	1,70577256	0,00017438
Cyclin a2	*Ccna2*	1,70522718	0,00103975
Tao kinase 1	*Taok1*	1,70466687	3,55e-05
Family with sequence similarity 43, member a	*Fam43a*	1,70436983	4,78e-05
Calcium binding protein 39-like	*Cab39l*	1,70380813	0,00050875
Family with sequence similarity 64, member a	*Fam64a*	1,70376598	4,33e-05
Myotubularin related protein 9	*Mtmr9*	1,70329374	0,00117321
Phosphatidylserine decarboxylase /// phosphatidylserine decarboxylase, pseudogene 1 /// phosphatidylserine decarboxylase, pseudogene 3	*Pisd /// pisd-ps1 /// pisd-ps3*	1,70262169	1,92e-05
Imp1 inner mitochondrial membrane peptidase-like (s. Cerevisiae)	*Immp1l*	1,7008647	0,00077632
Proteasome (prosome, macropain) activator subunit 4	*Psme4*	1,69956388	0,00013222
Golgi associated, gamma adaptin ear containing, arf binding protein 1	*Gga1*	1,69897591	8,52e-05
Zinc finger protein 397	*Zfp397*	1,69881517	0,00013339
Melanoma antigen, family d, 2	*Maged2*	1,69880738	1,17e-05
Atpase, h+ transporting, lysosomal v0 subunit a1	*Atp6v0a1*	1,69736491	0,00010739
Netrin 4	*Ntn4*	1,69687266	0,00229246
Excision repair cross-complementing rodent repair deficiency complementation group 6 like	*Ercc6l*	1,69683113	2,43e-05
Fidgetin	*Fign*	1,69671402	0,0023225
Ubiquinol-cytochrome c reductase, complex iii subunit xi	*Uqcr11*	1,6962055	0,00021008
Cdna sequence bc022687	*Bc022687*	1,69615709	0,00035508
Phosphatidylinositol transfer protein, membrane-associated 2	*Pitpnm2*	1,69564039	0,00391273
Ellis van creveld gene syndrome	*Evc*	1,69547569	0,00284043
Riken cdna 2210418o10 gene	*2210418o10rik*	1,69425154	0,00276494
Idnk gluconokinase homolog (e. Coli)	*Idnk*	1,69420217	5,14e-05
Mitochondrial ribosomal protein l42	*Mrpl42*	1,69406276	2,43e-05
Polymerase (rna) iii (dna directed) polypeptide k	*Polr3k*	1,69274808	0,00045627
X-linked lymphocyte-regulated 3a /// x-linked lymphocyte-regulated 3b /// x-linked lymphocyte-regulated 3c	*Xlr3a /// xlr3b /// xlr3c*	1,6920725	0,00013127
Vacuolar protein sorting 28 (yeast)	*Vps28*	1,69205445	2,28e-05
Non-smc element 1 homolog (s. Cerevisiae)	*Nsmce1*	1,69033592	4,45e-05
Ww domain binding protein 2	*Wbp2*	1,68968799	0,00011899
Hyaluronan mediated motility receptor (rhamm)	*Hmmr*	1,68946575	6,83e-05
Sodium channel modifier 1	*Scnm1*	1,68926578	0,00023652
Riken cdna 2310030g06 gene	*2310030g06rik*	1,68898363	0,00093044
Riken cdna 4930430f08 gene	*4930430f08rik*	1,68887195	0,00113761
Sh3-binding kinase 1	*Sbk1*	1,68868053	0,00021658
Mesoderm induction early response 1, family member 2	*Mier2*	1,68847921	4,61e-05
Rho-associated coiled-coil containing protein kinase 2	*Rock2*	1,68712743	6,26e-05
Glutamate receptor, ionotropic, ampa1 (alpha 1)	*Gria1*	1,68657459	0,00049114

Riken cdna 2510002d24 gene	*2510002d24rik*	1,68516894	0,00037796
Guanine nucleotide binding protein, alpha 11	*Gna11*	1,68512558	3,84e-05
Polymerase (rna) ii (dna directed) polypeptide d	*Polr2d*	1,68450121	4,54e-05
Coenzyme q10 homolog a (yeast)	*Coq10a*	1,68364297	1,10e-05
Kinesin family member 21a	*Kif21a*	1,68242648	0,00012763
Cd14 antigen	*Cd14*	1,68215204	8,23e-06
Trna aspartic acid methyltransferase 1	*Trdmt1*	1,68109908	0,00819458
5'-nucleotidase domain containing 3	*Nt5dc3*	1,68103188	0,00013176
F-box protein 17	*Fbxo17*	1,68067808	0,00220535
Chromobox 8	*Cbx8*	1,67905601	0,00155359
Replication protein a1	*Rpa1*	1,67848318	0,00102596
Paired box 6	*Pax6*	1,67817891	0,00244386
Tata box binding protein (tbp)-associated factor, rna polymerase i, a	*Taf1a*	1,67764819	0,0001909
Abhydrolase domain containing 12	*Abhd12*	1,67640669	0,00101818
2,3-bisphosphoglycerate mutase	*Bpgm*	1,67639172	0,00645999
Tubulin, alpha 3a /// tubulin, alpha 3b	*Tuba3a /// tuba3b*	1,67636236	6,66e-05
A disintegrin and metallopeptidase domain 15 (metargidin)	*Adam15*	1,67602053	8,84e-05
Activating signal cointegrator 1 complex subunit 1	*Ascc1*	1,67572108	0,00104038
Synaptotagmin xi	*Syt11*	1,67528479	0,00099472
Nadh dehydrogenase (ubiquinone) 1 alpha subcomplex, 2	*Ndufa2*	1,67527112	3,49e-05
Phosphatidylinositol-5-phosphate 4-kinase, type ii, gamma	*Pip4k2c*	1,67460901	0,000649
Family with sequence similarity 83, member g	*Fam83g*	1,67326233	5,84e-05
Sh3-domain kinase binding protein 1	*Sh3kbp1*	1,67157676	4,18e-05
N-ethylmaleimide sensitive fusion protein	*Nsf*	1,6714461	0,00010518
Testis expressed gene 2	*Tex2*	1,67049444	0,00024326
Tdp-glucose 4,6-dehydratase	*Tgds*	1,67004809	0,00224586
Nucleoporin 37	*Nup37*	1,66950544	3,62e-05
6-phosphofructo-2-kinase/fructose-2,6-biphosphatase 1	*Pfkfb1*	1,66931461	0,0004126
Slc10a3-ubl4 readthrough /// ubiquitin-like 4	*Slc10a3-ubl4 /// ubl4*	1,66912608	6,40e-05
Nfu1 iron-sulfur cluster scaffold homolog (s. Cerevisiae)	*Nfu1*	1,66909726	6,22e-05
Rna binding motif protein 22	*Rbm22*	1,66899565	0,00013512
Gli pathogenesis-related 2	*Glipr2*	1,66817066	0,00011844
Calcium/calmodulin-dependent protein kinase ii gamma	*Camk2g*	1,66782177	7,53e-05
Dna fragmentation factor, alpha subunit	*Dffa*	1,66775138	0,00053122
Mitogen-activated protein kinase kinase kinase 1	*Map3k1*	1,66749315	0,00081812
Nadh dehydrogenase (ubiquinone) 1 beta subcomplex, 5	*Ndufb5*	1,66746643	2,66e-05
Sorbin and sh3 domain containing 3	*Sorbs3*	1,66649141	0,00161879
Transformation related protein 53	*Trp53*	1,66637266	0,00083681
Pdz and lim domain 5	*Pdlim5*	1,66535055	4,32e-05
Coronin 6	*Coro6*	1,66493425	0,00370325

Riken cdna 2700094k13 gene	*2700094k13rik*	1,66464274	8,10e-05
Glycoprotein (transmembrane) nmb	*Gpnmb*	1,66350502	1,60e-05
Sorbin and sh3 domain containing 1	*Sorbs1*	1,66287993	0,00298254
Churchill domain containing 1	*Churc1*	1,66278137	0,00024765
Procollagen c-endopeptidase enhancer protein	*Pcolce*	1,66276065	0,00025197
G protein-coupled receptor 89	*Gpr89*	1,66265342	3,09e-05
Nuclear factor i/a	*Nfia*	1,66247375	0,00043243
Myosin ie	*Myo1e*	1,66145905	0,00019796
Atp synthase, h+ transporting, mitochondrial f1 complex, epsilon subunit	*Atp5e*	1,66120577	0,00045609
Zinc fingers and homeoboxes 1	*Zhx1*	1,66114678	0,00042552
Secretion regulating guanine nucleotide exchange factor	*Sergef*	1,6606879	8,42e-05
Mevalonate kinase	*Mvk*	1,65980318	0,00016109
Ring finger protein 138	*Rnf138*	1,65941524	0,00392192
Hras-like suppressor	*Hrasls*	1,65870264	0,00044289
Antigen identified by monoclonal antibody ki 67	*Mki67*	1,65796813	1,47e-05
Deafness, autosomal dominant 5 (human)	*Dfna5*	1,6579035	0,00201699
Regulator of calcineurin 3	*Rcan3*	1,65739141	0,00036915
Zinc finger with ufm1-specific peptidase domain	*Zufsp*	1,65731481	2,10e-05
5'-nucleotidase domain containing 2	*Nt5dc2*	1,65695293	0,00021518
Mitochondrial fission regulator 2	*Mtfr2*	1,65638822	0,00273404
Ring finger protein 145	*Rnf145*	1,65611914	3,94e-05
Tubulin, delta 1	*Tubd1*	1,65571707	0,00227479
Inositol 1,3,4-triphosphate 5/6 kinase	*Itpk1*	1,65558749	6,07e-05
Midnolin	*Midn*	1,65426918	0,00023466
Arfgap with fg repeats 2	*Agfg2*	1,65388226	0,00020686
Centromere protein f	*Cenpf*	1,65345587	0,00011443
Glucosamine-6-phosphate deaminase 1 pseudogene /// glucosamine-6-phosphate deaminase 1	*Gm8615 /// gnpda1*	1,65181745	0,00013264
Spectrin repeat containing, nuclear envelope 2	*Syne2*	1,6516199	0,00049387
Neuronal pentraxin chromo domain /// neuronal pentraxin receptor	*Npcd /// nptxr*	1,65155884	0,00090869
Ribonuclease, rnase k	*Rnasek*	1,6515071	0,00016512
Aurora kinase b	*Aurkb*	1,65112889	0,00025078
Checkpoint kinase 2	*Chek2*	1,65109373	0,0007617
Cholecystokinin b receptor	*Cckbr*	1,65066946	0,00101118
Galanin receptor 3 /// glycine c-acetyltransferase (2-amino-3-ketobutyrate-coenzyme a ligase)	*Galr3 /// gcat*	1,65063289	9,88e-05
Chitinase domain containing 1	*Chid1*	1,6503318	9,37e-05
Septin 9	*Ix.09*	1,65020087	0,00071769
Exocyst complex component 7	*Exoc7*	1,65002308	3,76e-05
Methylmalonic aciduria (cobalamin deficiency) type b homolog (human)	*Mmab*	1,64990948	0,0004081
Cyclin-dependent kinase inhibitor 2d (p19, inhibits cdk4)	*Cdkn2d*	1,64975357	2,63e-05
Ddrgk domain containing 1	*Ddrgk1*	1,64956473	4,44e-05

Fos-like antigen 1	*Fosl1*	1,64925961	0,00081464
N-acetyl galactosaminidase, alpha	*Naga*	1,64814568	0,00024211
Ten1 telomerase capping complex subunit	*Ten1*	1,64779769	8,22e-05
Atpase, h+ transporting, lysosomal v1 subunit d	*Atp6v1d*	1,647748	6,11e-05
Carbohydrate (keratan sulfate gal-6) sulfotransferase 1	*Chst1*	1,64768519	0,00165146
Riken cdna 2700062c07 gene /// predicted gene 9182	*2700062c07rik /// gm9182*	1,64766756	0,00046985
Serine dehydratase-like	*Sdsl*	1,64691436	3,72e-05
Interferon regulatory factor 5	*Irf5*	1,64681022	0,00178751
Transmembrane protein 5	*Tmem5*	1,64634482	2,96e-05
Gdp-mannose pyrophosphorylase a	*Gmppa*	1,6462322	4,40e-05
Retinoblastoma-like 1 (p107)	*Rbl1*	1,64556614	0,00029081
Matrix metallopeptidase 11	*Mmp11*	1,64547577	0,0002073
Nerve growth factor receptor (tnfrsf16) associated protein 1	*Ngfrap1*	1,64519591	2,29e-05
Fragile x mental retardation syndrome 1	*Fmr1*	1,64472031	0,00497219
Sh3 domain ysc-like 1	*Sh3yl1*	1,64431627	5,22e-05
Bromodomain containing 8	*Brd8*	1,64396552	8,65e-05
Hect domain and ankyrin repeat containing, e3 ubiquitin protein ligase 1	*Hace1*	1,64199791	0,00161879
Epithelial membrane protein 2	*Emp2*	1,64142409	0,00023129
Zinc finger, cchc domain containing 18	*Zcchc18*	1,64080587	0,00331724
Tsc22 domain family, member 1	*Tsc22d1*	1,64043061	0,00015141
Wd repeat domain 13	*Wdr13*	1,64013893	1,64e-05
Emerin	*Emd*	1,63971256	0,0001695
Flightless i homolog (drosophila)	*Flii*	1,63915827	0,00017972
Phosphoribosyl pyrophosphate synthetase 2	*Prps2*	1,63811414	0,0036941
Insulin-like growth factor 1	*Igf1*	1,63808868	0,00023828
Chloride channel calcium activated 1	*Clca1*	1,63799059	0,0015992
Diazepam binding inhibitor	*Dbi*	1,63780818	3,09e-05
Centrosomal protein 290	*Cep290*	1,63776199	0,00096564
Phenylalkylamine ca2+ antagonist (emopamil) binding protein	*Ebp*	1,63756183	8,99e-05
Zinc finger and scan domain containing 21	*Zscan21*	1,6372491	0,00012009
Docking protein 1	*Dok1*	1,6370501	5,21e-05
N-acetyl transferase 1	*Nat1*	1,6367212	0,00265497
Lactamase, beta 2	*Lactb2*	1,63672038	0,00613684
Sec23b (s. Cerevisiae)	*Sec23b*	1,63671953	0,00323509
Morn repeat containing 4	*Morn4*	1,63666853	0,00113862
Nicolin 1	*Nicn1*	1,63621074	0,00040402
Extracellular matrix protein 1	*Ecm1*	1,63581078	0,00040787
Wd repeat and socs box-containing 2	*Wsb2*	1,63565635	0,0001208
Coatomer protein complex, subunit gamma 1	*Copg1*	1,63517243	9,88e-05
Myosin, light polypeptide 6, alkali, smooth muscle and non-muscle	*Myl6*	1,63487947	0,0003467
Phospholipase a2, group vi	*Pla2g6*	1,63450033	0,00114692
Structural maintenance of chromosomes 1a	*Smc1a*	1,63404501	3,10e-05

Transmembrane and coiled-coil domains 6	*Tmco6*	1,63399579	8,44e-05
Stam binding protein	*Stambp*	1,63271367	0,00682319
Ribonucleotide reductase m2 b (tp53 inducible)	*Rrm2b*	1,63251295	0,00032867
Ribonuclease/angiogenin inhibitor 1	*Rnh1*	1,63237534	2,58e-05
Josephin domain containing 2	*Josd2*	1,63200508	0,00122765
Ribosome binding protein 1	*Rrbp1*	1,63191569	0,00052211
Sperm specific antigen 2	*Ssfa2*	1,63160405	0,00018131
Mus musculus adult male lung cdna, riken full-length enriched library, clone:1200016e24 product:unclassifiable, full insert sequence. /// predicted gene, 20186	*Bc057675 /// gm20186*	1,63154073	0,00241646
Tudor domain containing 7	*Tdrd7*	1,63137559	9,95e-05
Guanine nucleotide binding protein, alpha o	*Gnao1*	1,63134068	0,00952001
Nudix (nucleoside diphosphate linked moiety x)-type motif 6	*Nudt6*	1,63022713	0,00085543
Importin 8	*Ipo8*	1,63014334	2,21e-05
Dual specificity phosphatase 11 (rna/rnp complex 1-interacting)	*Dusp11*	1,62968769	0,00135527
Citron	*Cit*	1,62945186	0,0068626
Predicted gene 9840 /// ring-box 1	*Gm9840 /// rbx1*	1,62919887	5,40e-05
Asialoglycoprotein receptor 1	*Asgr1*	1,62866931	0,00512208
Sh3-domain grb2-like endophilin b2	*Sh3glb2*	1,62807355	3,85e-05
Calcitonin receptor-like	*Calcrl*	1,62755106	0,00282269
Ankyrin repeat domain 13a	*Ankrd13a*	1,62704152	3,77e-05
Transcription elongation regulator 1 (ca150)	*Tcerg1*	1,62663482	0,00029423
Rna polymerase ii associated protein 3	*Rpap3*	1,62636692	0,0001406
Magnesium-dependent phosphatase 1	*Mdp1*	1,62613901	0,00015038
Serine (or cysteine) peptidase inhibitor, clade i, member 1	*Serpini1*	1,62541559	0,00046736
Nadh dehydrogenase (ubiquinone) 1 beta subcomplex, 2	*Ndufb2*	1,62536883	0,00033966
Cullin 2	*Cul2*	1,62530223	0,00135889
Atp synthase, h+ transporting, mitochondrial f1 complex, alpha subunit 1	*Atp5a1*	1,62473087	0,00010345
Gata binding protein 2	*Gata2*	1,62445695	0,00050524
Limb-bud and heart	*Lbh*	1,62418848	0,00207626
Centromere protein m	*Cenpm*	1,62408148	0,00084751
Transmembrane protein 159	*Tmem159*	1,62354757	0,0002219
Predicted gene 11554 /// predicted gene 11569 /// keratin associated protein 4-13	*Gm11554 /// gm11569 /// krtap4-13*	1,6230392	0,00242569
Alpha-methylacyl-coa racemase	*Amacr*	1,62241194	0,00010238
Solute carrier family 27 (fatty acid transporter), member 4	*Slc27a4*	1,6221216	7,64e-05
St8 alpha-n-acetyl-neuraminide alpha-2,8-sialyltransferase 2	*St8sia2*	1,62178889	0,00161173
Heat-responsive protein 12	*Hrsp12*	1,62172145	0,00112149
Raftlin family member 2	*Rftn2*	1,62134869	0,00021633
Secretogranin v	*Scg5*	1,6199135	0,007122

High mobility group at-hook 1 /// high mobility group at-hook i, related sequence 1	*Hmga1 /// hmga1-rs1*	1,61947709	0,00629013
Mitogen-activated protein kinase 3	*Mapk3*	1,61943479	0,0001891
Integral membrane protein 2c	*Itm2c*	1,6188293	7,46e-05
Hmg box domain containing 3	*Hmgxb3*	1,6185888	0,00014726
Riken cdna d930015e06 gene	*D930015e06rik*	1,61851749	8,54e-05
Sema domain, immunoglobulin domain (ig), tm domain, and short cytoplasmic domain	*Sema4f*	1,61830314	0,00564487
Hydroxysteroid dehydrogenase like 2	*Hsdl2*	1,6179807	0,00330788
Procollagen-proline, 2-oxoglutarate 4-dioxygenase (proline 4-hydroxylase), alpha 1 polypeptide	*P4ha1*	1,61792591	0,00171855
Cytochrome c oxidase assembly protein 19	*Cox19*	1,61725628	9,51e-05
G protein-coupled receptor kinase 5	*Grk5*	1,61706626	0,00168058
Sh3 domain binding glutamic acid-rich protein-like 3	*Sh3bgrl3*	1,61684913	7,10e-05
Unc-13 homolog b (c. Elegans)	*Unc13b*	1,61662503	0,00412602
Aminopeptidase-like 1	*Npepl1*	1,61537186	3,72e-05
Ribokinase	*Rbks*	1,6148204	0,00105645
Filamin, alpha	*Flna*	1,61395638	4,92e-05
Tripartite motif-containing 21	*Trim21*	1,61344784	0,00014467
Cytohesin 2	*Cyth2*	1,61337759	5,88e-05
Glucocorticoid induced transcript 1	*Glcci1*	1,61334243	0,00273936
Vacuolar protein sorting 33b (yeast)	*Vps33b*	1,61333805	0,00048739
Argininosuccinate lyase	*Asl*	1,6129553	0,00082205
Deoxyguanosine kinase	*Dguok*	1,61251306	0,00036476
Tnf receptor-associated factor 5	*Traf5*	1,61243912	5,85e-05
Thimet oligopeptidase 1	*Thop1*	1,61237343	0,0015323
Vesicular, overexpressed in cancer, prosurvival protein 1	*Vopp1*	1,61194813	0,00061828
Heparan sulfate (glucosamine) 3-o-sulfotransferase 1	*Hs3st1*	1,61179512	0,00067341
Asparagine-linked glycosylation 13	*Alg13*	1,61165277	0,00683466
Clpb caseinolytic peptidase b	*Clpb*	1,61068538	0,00121444
Methyl-cpg binding domain protein 1	*Mbd1*	1,61056872	7,03e-05
Sprouty-related, evh1 domain containing 2	*Spred2*	1,61051132	3,03e-05
Ubiquitin-conjugating enzyme e2e 2	*Ube2e2*	1,60850288	0,00019925
Crystallin, beta b1	*Crybb1*	1,60723163	0,00259916
Discs, large homolog 4 (drosophila)	*Dlg4*	1,60711041	0,0035767
Intraflagellar transport 80	*Ift80*	1,60548856	0,00012216
Immature colon carcinoma transcript 1	*Ict1*	1,6051103	5,27e-05
Polymerase i and transcript release factor	*Ptrf*	1,60464171	0,00432788
Kinesin family member 23	*Kif23*	1,60392585	0,00024657
Homeobox b6	*Hoxb6*	1,6034517	0,00069177
Required for meiotic nuclear division 5 homolog b (s. Cerevisiae)	*Rmnd5b*	1,60306871	4,14e-05
Selenoprotein k	*Selk*	1,60277522	6,25e-05
Riken cdna 6430706d22 gene /// riken cdna a730008h23 gene /// holliday junction recognition protein	*6430706d22rik /// a730008h23rik /// hjurp*	1,60198069	0,00017925

Calcium channel, voltage-dependent, t type, alpha 1g subunit	*Cacna1g*	1,60140129	0,00384282
Adenomatosis polyposis coli 2	*Apc2*	1,60109499	0,00391273
Solute carrier family 22 (organic cation transporter), member 4	*Slc22a4*	1,60039089	0,00078884
Predicted gene 12854 /// predicted gene 5068 /// s100 calcium binding protein a11 (calgizzarin)	*Gm12854 /// gm5068 /// s100a11*	1,60037216	7,53e-05
Ptk2 protein tyrosine kinase 2	*Ptk2*	1,60016383	0,00010075
Zinc finger protein 672	*Zfp672*	1,59936461	0,0003289
Solute carrier family 8 (sodium/lithium/calcium exchanger), member b1	*Slc8b1*	1,59933478	0,00018039
Ikbkb interacting protein	*Ikbip*	1,59908607	6,80e-05
Retinoblastoma binding protein 8	*Rbbp8*	1,59845565	0,00010745
Rho-associated coiled-coil containing protein kinase 1	*Rock1*	1,5977236	0,00191277
Mitochondrial ribosomal protein s18a	*Mrps18a*	1,59764584	3,40e-05
Ornithine decarboxylase antizyme 1 /// ornithine decarboxylase antizyme 1, pseudogene	*Oaz1 /// oaz1-ps*	1,59730576	0,00016059
Comm domain containing 1	*Commd1*	1,5968255	5,29e-05
Sar1 gene homolog a (s. Cerevisiae)	*Sar1a*	1,59670072	3,06e-05
P450 (cytochrome) oxidoreductase	*Por*	1,59623336	0,0004214
Coiled-coil domain containing 64	*Ccdc64*	1,59620704	0,00128457
Solute carrier family 2, (facilitated glucose transporter), member 8	*Slc2a8*	1,59529739	0,00058055
Atp-binding cassette, sub-family a (abc1), member 7	*Abca7*	1,59501811	0,00073603
Carboxypeptidase q	*Cpq*	1,59482203	3,84e-05
Phosphoglucomutase 1	*Pgm1*	1,59463116	9,09e-05
Dnaj (hsp40) homolog, subfamily b, member 2	*Dnajb2*	1,59336263	0,00010523
Period circadian clock 3	*Per3*	1,59332652	0,00105574
Solute carrier family 38, member 10	*Slc38a10*	1,5933065	0,00054256
Period circadian clock 2	*Per2*	1,59319148	0,00048245
La ribonucleoprotein domain family, member 1b	*Larp1b*	1,59290513	0,00016225
Acyl-coa thioesterase 13	*Acot13*	1,59265808	0,00038686
Transmembrane bax inhibitor motif containing 1	*Tmbim1*	1,59253474	0,00025556
Neurofilament, heavy polypeptide	*Nefh*	1,59226702	0,00198015
Pdz domain containing 11	*Pdzd11*	1,59206043	0,00079761
Budding uninhibited by benzimidazoles 1 homolog (s. Cerevisiae)	*Bub1*	1,5907517	0,00010884
Asparaginase like 1	*Asrgl1*	1,59044736	0,00013223
Progestin and adipoq receptor family member iv	*Paqr4*	1,5903949	0,00012522
Spectrin beta, non-erythrocytic 1	*Sptbn1*	1,59032626	0,00125854
Coiled-coil domain containing 159	*Ccdc159*	1,58932289	0,00064545
Zinc finger protein 36	*Zfp36*	1,58870918	0,00074446
Methyltransferase like 21a	*Mettl21a*	1,58867876	0,00817062
Von hippel-lindau binding protein 1	*Vbp1*	1,5883704	0,00144228
Talin 1	*Tln1*	1,58814811	0,00141052
Translocase of inner mitochondrial membrane 22	*Timm22*	1,58787468	8,94e-05
Sap30 binding protein	*Sap30bp*	1,58743844	0,00018108

Protein phosphatase 3, regulatory subunit b, alpha isoform (calcineurin b, type i)	*Ppp3r1*	1,58711137	0,00488818
Leiomodin 2 (cardiac)	*Lmod2*	1,58697186	0,00062623
Ubiquitin-conjugating enzyme e2 n-like /// ubiquitin-conjugating enzyme e2n	*Mcg1038069 /// ube2n*	1,58677884	0,00968858
Peptidase inhibitor 15	*Pi15*	1,58640462	0,00084185
Expressed sequence ai413582	*Ai413582*	1,58628592	0,0034131
Protein kinase c, alpha	*Prkca*	1,58620364	0,00013782
Rna 2',3'-cyclic phosphate and 5'-oh ligase	*Rtcb*	1,58603433	3,66e-05
Ribosome production factor 2 homolog (s. Cerevisiae)	*Rpf2*	1,58579309	0,00021834
Thymoma viral proto-oncogene 1	*Akt1*	1,58547132	0,00157084
Protein phosphatase 3, catalytic subunit, gamma isoform	*Ppp3cc*	1,58543774	0,00110602
Rab1, member ras oncogene family	*Rab1*	1,58537323	0,00205602
Tank-binding kinase 1	*Tbk1*	1,58533287	0,005699
Spindle apparatus coiled-coil protein 1	*Spdl1*	1,58498143	0,0011137
Glycolipid transfer protein	*Gltp*	1,58458004	0,00209294
Predicted gene 3650 /// spastic paraplegia 11	*Gm3650 /// spg11*	1,58451578	8,78e-05
Pleckstrin homology domain-containing, family a (phosphoinositide binding specific) member 3	*Plekha3*	1,58424935	0,00437
Ubiquitin-like modifier activating enzyme 7	*Uba7*	1,58419531	0,00061569
Signal peptidase complex subunit 1 homolog (s. Cerevisiae)	*Spcs1*	1,58406019	0,00928181
Thymosin, beta 4, x chromosome	*Tmsb4x*	1,58388471	2,41e-05
Thap domain containing, apoptosis associated protein 2	*Thap2*	1,5835536	0,00212076
Epidermal growth factor receptor pathway substrate 8	*Eps8*	1,58313401	0,00029767
Protein kinase, camp dependent regulatory, type i beta	*Prkar1b*	1,58292208	3,07e-05
Cytoplasmic polyadenylation element binding protein 4	*Cpeb4*	1,58153344	0,00320473
Polymerase (dna-directed), epsilon 4 (p12 subunit)	*Pole4*	1,58152381	0,00201088
Teashirt zinc finger family member 1	*Tshz1*	1,58096369	6,37e-05
Frizzled homolog 8 (drosophila)	*Fzd8*	1,58083627	0,00035559
Comm domain containing 6	*Commd6*	1,58064695	0,00015343
Phosphatidylglycerophosphate synthase 1	*Pgs1*	1,58055957	7,46e-05
Zinc finger protein 35	*Zfp35*	1,5801319	0,00033051
Map7 domain containing 1	*Map7d1*	1,57997576	0,00021374
Calsequestrin 2	*Casq2*	1,57993126	0,00282601
Bladder cancer associated protein homolog (human)	*Blcap*	1,57978277	0,00025208
Riken cdna 2310011j03 gene	*2310011j03rik*	1,57970084	2,71e-05
Polyadenylate-binding protein-interacting protein 2	*Paip2*	1,57910105	0,00019748
Pan2 polya specific ribonuclease subunit homolog (s. Cerevisiae)	*Pan2*	1,57900864	0,00403361
Eukaryotic translation initiation factor 4 gamma, 3	*Eif4g3*	1,57793043	0,0002544
Smad family member 4	*Smad4*	1,57685149	0,00016449
Lysosomal-associated protein transmembrane 4b	*Laptm4b*	1,5763611	0,00010016
Pih1 domain containing 1	*Pih1d1*	1,57630193	6,34e-05
Coiled-coil domain containing 53	*Ccdc53*	1,57598625	0,00066347

Carbohydrate sulfotransferase 11	*Chst11*	1,5754445	0,00587783
Lysophospholipase 1	*Lypla1*	1,57489668	0,00358651
Tripartite motif-containing 3	*Trim3*	1,57464639	0,00498788
Chemokine (c-x-c motif) ligand 12	*Cxcl12*	1,57451847	0,0003551
Oxidation resistance 1	*Oxr1*	1,57444909	7,59e-05
Anillin, actin binding protein	*Anln*	1,57419118	0,00047136
Citrate synthase	*Cs*	1,57366439	0,00012829
Nsfl1 (p97) cofactor (p47)	*Nsfl1c*	1,57336042	9,36e-05
Methylenetetrahydrofolate dehydrogenase (nadp+ dependent), methenyltetrahydrofolate cyclohydrolase, formyltetrahydrofolate synthase	*Mthfd1*	1,57310313	6,11e-05
Reticulon 4 interacting protein 1	*Rtn4ip1*	1,57280512	0,00011001
Eukaryotic translation initiation factor 1a, x-linked	*Eif1ax*	1,57267481	0,00166522
Pleckstrin homology domain containing, family b (evectins) member 2	*Plekhb2*	1,57242804	0,0011149
Polo-like kinase 2	*Plk2*	1,57230796	0,00012249
Estrogen receptor-binding fragment-associated gene 9	*Ebag9*	1,57175344	0,00085445
Tetraspanin 31	*Tspan31*	1,57082055	4,88e-05
Family with sequence similarity 53, member c	*Fam53c*	1,57072295	0,00010373
Anaphase-promoting complex subunit 5	*Anapc5*	1,57070871	0,00958648
Riken cdna 2210016l21 gene	*2210016l21rik*	1,56985745	0,00119584
Riken cdna 8430419l09 gene	*8430419l09rik*	1,56967852	0,00025263
Kinesin family member 5b	*Kif5b*	1,5693171	0,00014956
Isocitrate dehydrogenase 1 (nadp+), soluble	*Idh1*	1,56904533	0,00013113
Glycerophosphocholine phosphodiesterase gde1 homolog (s. Cerevisiae)	*Gpcpd1*	1,56863433	0,00410003
Tubulin, alpha 1a	*Tuba1a*	1,56822058	0,00019103
Cd24a antigen	*Cd24a*	1,56786042	0,00813973
Phospholipase b domain containing 2	*Plbd2*	1,56775031	0,00051589
Keratin 33b	*Krt33b*	1,56768788	0,00010542
Uncharacterized loc269472	*Loc269472*	1,56761804	0,00103246
Atp-binding cassette, sub-family c (cftr/mrp), member 5	*Abcc5*	1,56749341	0,00680331
Tubulin, beta 4b class ivb	*Tubb4b*	1,56696887	3,96e-05
Riken cdna 1110051m20 gene	*1110051m20rik*	1,5666705	5,65e-05
Asunder, spermatogenesis regulator	*Asun*	1,56618599	0,00038705
Fatty acid synthase	*Fasn*	1,56580001	0,00012121
Protein kinase d2	*Prkd2*	1,56570984	0,00049529
Activin a receptor, type 1	*Acvr1*	1,56546727	0,00266862
Run and sh3 domain containing 1	*Rusc1*	1,5652001	0,00223319
Cysteine-rich secretory protein 2	*Crisp2*	1,56506394	0,00040199
T cell receptor alpha variable 9d-3	*Trav9d-3*	1,56484998	0,00552409
Mitogen-activated protein kinase binding protein 1	*Mapkbp1*	1,56468569	0,00136922
B cell cll/lymphoma 7b	*Bcl7b*	1,56439128	0,00072085
Iq motif containing gtpase activating protein 1	*Iqgap1*	1,5640744	0,00010796
Fatty acid binding protein 4, adipocyte	*Fabp4*	1,56395091	0,00031819

Polymerase (dna directed), alpha 1	*Pola1*	1,56335213	0,00077803
Mediator complex subunit 16	*Med16*	1,56330913	0,00025661
Lactation elevated 1	*Lace1*	1,56307759	0,00074224
Ubiquitin specific peptidase 21	*Usp21*	1,56285016	0,00013768
5' nucleotidase, ecto	*Nt5e*	1,56279836	0,00623383
Potassium voltage-gated channel, shaker-related subfamily, beta member 3	*Kcnab3*	1,56262119	0,00156134
Small glutamine-rich tetratricopeptide repeat (tpr)-containing, alpha	*Sgta*	1,56157058	0,00012974
Mitogen-activated protein kinase kinase kinase 6	*Map3k6*	1,56108594	0,00040991
Regulator of g-protein signaling 20	*Rgs20*	1,56102372	0,00782543
Hairy/enhancer-of-split related with yrpw motif 1	*Hey1*	1,55979032	0,00026961
Ccr4-not transcription complex, subunit 2	*Cnot2*	1,5597896	0,00011072
Aconitase 2, mitochondrial	*Aco2*	1,55958936	6,90e-05
Major histocompatibility complex, class i-related	*Mr1*	1,55914903	0,00850478
S100 calcium binding protein a10 (calpactin)	*S100a10*	1,55854471	3,45e-05
Thioredoxin-related transmembrane protein 2	*Tmx2*	1,55831642	0,00025197
Serrate rna effector molecule homolog (arabidopsis)	*Srrt*	1,55787799	0,00055739
Ran gtpase activating protein 1	*Rangap1*	1,55786032	0,00010373
Staphylococcal nuclease and tudor domain containing 1	*Snd1*	1,55695234	3,06e-05
Microtubule-associated protein, rp/eb family, member 1	*Mapre1*	1,55667762	0,00282699
Zinc finger protein 54	*Zfp54*	1,5565252	0,00744143
Casein kinase 1, delta	*Csnk1d*	1,55633636	0,00018771
Kinetochore-localized astrin/spag5 binding	*Knstrn*	1,55605217	0,00126379
Cytoplasmic fmr1 interacting protein 1	*Cyfip1*	1,55605207	0,00035001
Sphingomyelin synthase 1	*Sgms1*	1,55528715	0,00018081
Sec24 related gene family, member d (s. Cerevisiae)	*Sec24d*	1,55444024	0,00014264
Interleukin 18	*Il18*	1,55377726	6,92e-05
Au rna binding protein/enoyl-coenzyme a hydratase	*Auh*	1,5537009	0,0036176
Farnesyltransferase, caax box, beta	*Fntb*	1,55352232	0,00300131
Myocyte enhancer factor 2c	*Mef2c*	1,55313898	0,00087005
Dehydrogenase/reductase (sdr family) member 1	*Dhrs1*	1,55310502	0,00151882
Solute carrier family 41, member 2	*Slc41a2*	1,5529264	0,00063124
Receptor accessory protein 3	*Reep3*	1,55289192	0,00027913
Dopa decarboxylase	*Ddc*	1,55268829	0,0015529
Establishment of cohesion 1 homolog 1 (s. Cerevisiae)	*Esco1*	1,55253481	0,00110385
Notch 4	*Notch4*	1,55249207	0,0023289
Oligosaccharyltransferase 4 homolog (s. Cerevisiae)	*Ost4*	1,5520209	0,00042281
Microtubule associated serine/threonine kinase-like	*Mastl*	1,5518193	0,00105114
Zinc finger protein 503	*Zfp503*	1,55176049	0,00717397
Emi domain containing 1	*Emid1*	1,55169206	0,00320982
Centromere protein a	*Cenpa*	1,55144407	0,00010332
Mediator complex subunit 23	*Med23*	1,55123534	0,00077436
Solute carrier family 41, member 3	*Slc41a3*	1,55116785	0,00432468

Sec31 homolog a (s. Cerevisiae)	*Sec31a*	1,55076653	0,00026345
Translocator protein	*Tspo*	1,55041138	0,00027973
Tuftelin 1	*Tuft1*	1,55027151	0,0001304
Calcineurin-like phosphoesterase domain containing 1	*Cpped1*	1,55021927	0,00183786
Filamin binding lim protein 1	*Fblim1*	1,54951853	0,00187458
Riken cdna 1810009n02 gene	*1810009n02rik*	1,54890862	0,0016163
1-acylglycerol-3-phosphate o-acyltransferase 3	*Agpat3*	1,54857303	0,00025042
Serine/threonine kinase 10	*Stk10*	1,54853596	0,00018505
High mobility group box transcription factor 1	*Hbp1*	1,54850323	0,00012109
Wd repeat domain 78	*Wdr78*	1,54847006	0,00066844
Ubiquitin specific peptidase 14	*Usp14*	1,54733496	8,81e-05
Syntrophin, acidic 1	*Snta1*	1,54668585	6,82e-05
Dynactin 1	*Dctn1*	1,54521208	0,00054859
Interferon, alpha-inducible protein 27 like 2a	*Ifi27l2a*	1,54501906	0,00025078
Riken cdna 1810030o07 gene	*1810030o07rik*	1,54424592	0,00272215
Methyltransferase like 23	*Mettl23*	1,54421511	0,00114744
N-deacetylase/n-sulfotransferase (heparan glucosaminyl) 1	*Ndst1*	1,54420899	4,22e-05
Sorting and assembly machinery component 50 homolog (s. Cerevisiae)	*Samm50*	1,54401352	0,00010181
St3 beta-galactoside alpha-2,3-sialyltransferase 1	*St3gal1*	1,5437939	0,00044193
Transcription elongation factor a (sii)-like 8	*Tceal8*	1,54362024	0,00470045
Protein kinase, amp-activated, gamma 1 non-catalytic subunit	*Prkag1*	1,54327415	0,00055636
Crystallin, alpha b	*Cryab*	1,54261016	0,00012009
Tumor necrosis factor, alpha-induced protein 8	*Tnfaip8*	1,54255983	0,00078653
G protein-coupled receptor 137b, pseudogene	*Gpr137b-ps*	1,54221371	0,00010009
Dynactin 2	*Dctn2*	1,5414252	0,00010075
Protein tyrosine phosphatase, receptor type, j	*Ptprj*	1,54098675	0,00758256
Relt-like 1	*Rell1*	1,54035826	0,00254647
Zinc finger, mym-type 3	*Zmym3*	1,54002448	0,00026985
Asparaginyl-trna synthetase	*Nars*	1,53988223	0,00019055
Transcriptional adaptor 1	*Tada1*	1,53966355	0,00968752
Pet112 homolog (s. Cerevisiae)	*Pet112*	1,53960814	0,00214147
Enoyl-coenzyme a, hydratase/3-hydroxyacyl coenzyme a dehydrogenase	*Ehhadh*	1,53950981	0,00134423
Seminal vesicle secretory protein 5	*Svs5*	1,53929223	0,00016309
Matrix metallopeptidase 19	*Mmp19*	1,53887905	0,00055685
Adipogenesis associated mth938 domain containing	*Aamdc*	1,53831902	0,00015539
Nucleoporin 107	*Nup107*	1,5382107	5,15e-05
Tubulin, gamma 1	*Tubg1*	1,53716024	0,00037508
Aldo-keto reductase family 1, member c12	*Akr1c12*	1,53693765	0,00012685
Guanosine monophosphate reductase 2	*Gmpr2*	1,53688819	0,00058614
Vimentin	*Vim*	1,53687258	4,04e-05
Zinc finger, ccch-type with g patch domain	*Zgpat*	1,53640954	7,46e-05
Septin 8	*Ix.08*	1,53593462	0,00025722

Erythrocyte protein band 4.1-like 2	*Epb4.1l2*	1,53543005	0,00152358
Mitogen-activated protein kinase kinase kinase 4	*Map3k4*	1,53522202	0,00047808
Thioredoxin reductase 2	*Txnrd2*	1,53440293	0,00233837
Mitochondrial translational initiation factor 2	*Mtif2*	1,53361475	0,00036723
Bpi fold containing family b, member 1	*Bpifb1*	1,5335655	0,00319857
Sema domain, transmembrane domain (tm), and cytoplasmic domain, (semaphorin) 6a	*Sema6a*	1,53354064	0,00825754
Achalasia, adrenocortical insufficiency, alacrimia	*Aaas*	1,53328373	0,0004915
Riken cdna 1810011o10 gene	*1810011o10rik*	1,53320946	0,00013539
Oxysterol binding protein-like 5	*Osbpl5*	1,53305745	0,00202598
Cold inducible rna binding protein	*Cirbp*	1,53294972	0,00022668
Riken cdna 6030458c11 gene	*6030458c11rik*	1,5325734	0,00020583
Kinesin family member 4	*Kif4*	1,53248684	0,00016356
Fucosidase, alpha-l- 1, tissue	*Fuca1*	1,5322795	0,00055276
St6 (alpha-n-acetyl-neuraminyl-2,3-beta-galactosyl-1,3)-n-acetylgalactosaminide alpha-2,6-sialyltransferase 6	*St6galnac6*	1,53187054	0,00169161
Carbohydrate sulfotransferase 10	*Chst10*	1,53181236	6,88e-05
Enhancer of polycomb homolog 1 (drosophila)	*Epc1*	1,53077897	0,00054444
Cell division cycle 34	*Cdc34*	1,5299129	0,00037774
X-ray repair complementing defective repair in chinese hamster cells 4	*Xrcc4*	1,52987171	0,00138635
F-box protein 6	*Fbxo6*	1,52969376	9,93e-05
Arachidonate 5-lipoxygenase activating protein	*Alox5ap*	1,52939909	0,0001788
Hig1 domain family, member 1c /// methyltransferase like 7a1 /// methyltransferase like 7a2 /// mettl7a2-higd1c readthrough transcript /// methyltransferase like 7a3	*Higd1c /// mettl7a1 /// mettl7a2 /// mettl7a2higd1c /// mettl7a3*	1,52931253	0,00175396
Dnaj (hsp40) homolog, subfamily c, member 24	*Dnajc24*	1,52876404	0,00760759
Late endosomal/lysosomal adaptor, mapk and mtor activator 2	*Lamtor2*	1,5287404	0,00015533
Phosphorylase kinase alpha 1	*Phka1*	1,52832967	0,00570677
Serine/arginine repetitive matrix 4	*Srrm4*	1,52831468	0,00234391
Suppressor of var1, 3-like 1 (s. Cerevisiae)	*Supv3l1*	1,52809612	7,74e-05
Coiled-coil domain containing 6	*Ccdc6*	1,52807014	6,85e-05
Scribbled homolog (drosophila)	*Scrib*	1,52805874	0,00282269
Lim motif-containing protein kinase 2	*Limk2*	1,52736534	0,00047433
Yeats domain containing 4	*Yeats4*	1,52701049	0,00010713
Inosine triphosphatase (nucleoside triphosphate pyrophosphatase)	*Itpa*	1,52679964	0,00046648
Spry domain containing 3	*Spryd3*	1,5261878	0,0004485
Leukemia inhibitory factor	*Lif*	1,52576173	0,00484346
3-oxoacid coa transferase 1	*Oxct1*	1,52572109	0,00015929
Mitochondrial ribosomal protein l33	*Mrpl33*	1,52528334	0,00103175
Coiled coil domain containing 88a	*Ccdc88a*	1,52486895	0,0001909
Riken cdna 2810417h13 gene	*2810417h13rik*	1,52451174	5,78e-05
C2 calcium-dependent domain containing 2-like	*C2cd2l*	1,52442505	0,00607751
Sec22 vesicle trafficking protein homolog b (s. Cerevisiae)	*Sec22b*	1,52429124	0,00012935

Tbc1 domain family, member 15	*Tbc1d15*	1,5240709	0,00411504
Nadh dehydrogenase (ubiquinone) 1 beta subcomplex, 9	*Ndufb9*	1,52338691	0,00036145
Eukaryotic translation initiation factor 4e binding protein 2	*Eif4ebp2*	1,52313789	0,00026985
Dual specificity phosphatase 14	*Dusp14*	1,52279793	0,00038701
Prion protein	*Prnp*	1,52275889	0,00016059
Musculoskeletal, embryonic nuclear protein 1	*Mustn1*	1,52231085	0,00224789
Autophagy related 12	*Atg12*	1,52225226	0,00027118
Prolyl endopeptidase-like	*Prepl*	1,52174369	0,00020171
Predicted gene 10193 /// zinc finger protein 706	*Gm10193 /// zfp706*	1,52157079	0,00085972
Hect domain and rld 4	*Herc4*	1,52108527	0,00103166
Maternal embryonic leucine zipper kinase	*Melk*	1,52065765	0,00300883
Protein regulator of cytokinesis 1	*Prc1*	1,52058377	8,89e-05
Transmembrane and coiled-coil domains 2	*Tmcc2*	1,52018281	0,00127535
Myeloid cell leukemia sequence 1	*Mcl1*	1,52008634	0,00268677
Plastin 3 (t-isoform)	*Pls3*	1,52005762	0,00030766
Dehydrodolichyl diphosphate synthase	*Dhdds*	1,51979408	0,00036358
Riken cdna 4932438a13 gene	*4932438a13rik*	1,5191771	0,0009318
Homeobox c6	*Hoxc6*	1,51884762	0,00914449
Fk506 binding protein 5	*Fkbp5*	1,5186897	0,00010761
Leucine-zipper-like transcriptional regulator, 1	*Lztr1*	1,51853963	0,00040615
Glypican 1	*Gpc1*	1,51793289	6,81e-05
Ubiquitin c	*Ubc*	1,51768685	0,00053094
Transcription factor 19	*Tcf19*	1,51767502	4,93e-05
Rotatin	*Rttn*	1,51740047	0,00024668
Fermitin family homolog 2 (drosophila)	*Fermt2*	1,51710355	0,00096276
Prominin 2	*Prom2*	1,51531761	0,00104512
Start domain containing 10	*Stard10*	1,51507493	0,00173462
Family with sequence similarity 73, member b	*Fam73b*	1,51456059	0,00016653
Predicted gene 12538 /// orm1-like 3 (s. Cerevisiae)	*Gm12538 /// ormdl3*	1,51427311	0,00017518
Cyclin d-type binding-protein 1	*Ccndbp1*	1,51384614	0,00029599
Choroidermia	*Chm*	1,513529	0,00020044
Ornithine decarboxylase antizyme 2	*Oaz2*	1,51341505	0,00022801
Leucine-rich repeats and wd repeat domain containing 1	*Lrwd1*	1,51319892	0,00021822
Dehydrogenase/reductase (sdr family) member 4	*Dhrs4*	1,51296311	0,00053599
Sub1 homolog (s. Cerevisiae)	*Sub1*	1,5127786	4,90e-05
Protein o-fucosyltransferase 2	*Pofut2*	1,51266367	0,00192371
Carbonic anhydrase 13	*Car13*	1,51209659	0,00097253
Tpx2, microtubule-associated protein homolog (xenopus laevis)	*Tpx2*	1,51087509	9,72e-05
Polymerase (rna) ii (dna directed) polypeptide e	*Polr2e*	1,51049565	8,36e-05
Protocadherin beta 14	*Pcdhb14*	1,51035233	0,00310511
Tumor susceptibility gene 101	*Tsg101*	1,51003597	0,00027481
Calpain 2	*Capn2*	1,50986148	0,00012655

Riken cdna d030029j20 gene	*D030029j20rik*	1,50952225	0,00185247
Trio and f-actin binding protein	*Triobp*	1,5094524	0,00367553
Phosphoseryl-trna kinase	*Pstk*	1,50939284	0,00023819
Potassium voltage gated channel, shab-related subfamily, member 1 /// protein kinase c and casein kinase substrate in neurons 2	*Kcnb1 /// pacsin2*	1,50937754	0,00011773
Endoplasmic reticulum lectin 1	*Erlec1*	1,50856006	0,00190256
Sema domain, immunoglobulin domain (ig), transmembrane domain (tm) and short cytoplasmic domain, (semaphorin) 4b	*Sema4b*	1,50765196	0,00159612
Cofilin 2, muscle	*Cfl2*	1,50756087	0,00124117
S-adenosylmethionine decarboxylase 1	*Amd1*	1,50726707	0,00014051
Calponin 3, acidic	*Cnn3*	1,50716596	0,00063058
Amplified in osteosarcoma	*Os9*	1,50701242	0,00300903
Retinol saturase (all trans retinol 13,14 reductase)	*Retsat*	1,50674148	0,0002769
Non-smc condensin i complex, subunit h	*Ncaph*	1,5066382	0,0002328
Cdna sequence bc018242	*Bc018242*	1,50641271	0,00536898
Caspase 8	*Casp8*	1,50567632	0,00801458
Oligophrenin 1	*Ophn1*	1,50547745	0,00050786
Zinc and ring finger 2	*Znrf2*	1,50504258	0,00020381
Ras-related c3 botulinum substrate 3	*Rac3*	1,50503522	0,00117374
Riken cdna 0610009o20 gene	*0610009o20rik*	1,50497856	0,00165115
Traf3 interacting protein 2	*Traf3ip2*	1,50472421	0,00039041
Rrs1 ribosome biogenesis regulator homolog pseudogene	*4632415l05rik*	1,504552	0,00569364
Dead (asp-glu-ala-asp) box polypeptide 50	*Ddx50*	1,50434621	8,23e-05
Inhibin beta-a	*Inhba*	1,50367737	0,00425161
Jumonji, at rich interactive domain 2	*Jarid2*	1,50342958	0,00103622
Protein tyrosine phosphatase, non-receptor type 2	*Ptpn2*	1,50312563	0,00513645
Adaptor protein, phosphotyrosine interaction, ph domain and leucine zipper containing 2	*Appl2*	1,50291377	0,00050658
Udp-n-acetyl-alpha-d-galactosamine:polypeptide n-acetylgalactosaminyltransferase 1	*Galnt1*	1,50269176	0,0001689
L antigen family, member 3	*Lage3*	1,501224	0,00011794
Plectin	*Plec*	1,50108166	0,00306773
Stanniocalcin 2	*Stc2*	1,50090234	0,00102835
High mobility group box 3	*Hmgb3*	1,50062532	0,00227913
Nudix (nucleoside diphosphate linked moiety x)-type motif 4	*Nudt4*	1,50055727	8,81e-05
Poly (adp-ribose) polymerase family, member 4	*Parp4*	1,50048281	0,00281973
Dead (asp-glu-ala-asp) box polypeptide 21	*Ddx21*	1,50037423	0,00020049
Mitochondrial rrna methyltransferase 1 homolog (s. Cerevisiae)	*Mrm1*	1,49982765	0,00027249
Immunoglobulin (cd79a) binding protein 1	*Igbp1*	1,49970492	0,00028494
N-acetylglucosamine kinase	*Nagk*	1,49950325	0,00063767
Hemochromatosis	*Hfe*	1,49892564	0,00038865
Ring finger protein 41	*Rnf41*	1,49858327	0,00095031
Tripartite motif-containing 34a	*Trim34a*	1,49697612	0,00754305

Surfeit gene 2	*Surf2*	1,49688186	0,00047433
Zinc finger prtoein 943	*Zfp943*	1,49664162	0,00078296
Calpain 5	*Capn5*	1,49660224	0,00146017
Endonuclease g	*Endog*	1,49592542	0,00472722
Tumor necrosis factor, alpha-induced protein 1 (endothelial)	*Tnfaip1*	1,49510635	0,00039512
Ankyrin repeat and socs box-containing 3	*Asb3*	1,49509907	0,00120682
Furry homolog-like (drosophila)	*Fryl*	1,49501023	0,00014144
Component of sp100-rs /// predicted pseudogene 15433 /// predicted gene 2666 /// predicted pseudogene 7609 /// uncharacterized loc100041903 /// uncharacterized loc100503923	*Csprs /// gm15433 /// gm2666 /// gm7609 /// loc100041903 /// loc100503923*	1,4949191	0,00565691
Retinoblastoma 1	*Rb1*	1,49489658	0,00215585
Centrosomal protein 350	*Cep350*	1,49463774	0,00151792
Zinc finger protein 39	*Zfp39*	1,49430814	0,00089558
Gtp binding protein 1	*Gtpbp1*	1,49407788	0,00029081
Vcp-interacting membrane protein	*Vimp*	1,49384314	0,0008094
Low density lipoprotein receptor	*Ldlr*	1,49378456	0,0002073
Tbc1 domain family, member 22a	*Tbc1d22a*	1,49291342	0,00015963
Pleckstrin homology domain containing, family g (with rhogef domain) member 2	*Plekhg2*	1,49276952	0,00718818
Rho gtpase activating protein 9	*Arhgap9*	1,49271914	0,00026923
Insulin-like growth factor 2, opposite strand	*Igf2os*	1,49182677	0,00201492
Aldolase a, fructose-bisphosphate	*Aldoa*	1,49177145	0,0038582
Cyclin-dependent kinase 5	*Cdk5*	1,49094566	0,00045561
Kdm3b lysine (k)-specific demethylase 3b	*Kdm3b*	1,49092297	0,00018659
Heat shock protein 2	*Hspa2*	1,4903547	0,00250243
Capicua homolog (drosophila)	*Cic*	1,49034317	0,00215012
Mitogen-activated protein kinase 8 interacting protein 1	*Mapk8ip1*	1,49007388	0,00899634
Tao kinase 3	*Taok3*	1,48994266	0,00817875
Signal transducer and activator of transcription 6	*Stat6*	1,48868095	0,00043109
Rfad1, flavin adenine dinucleotide synthetase, homolog (yeast) /// lens epithelial protein	*Flad1 /// lenep*	1,48866441	0,00315415
Plakophilin 4	*Pkp4*	1,48827666	0,00022376
Jnk1/mapk8-associated membrane protein	*Jkamp*	1,48820849	0,00068881
Sam domain, sh3 domain and nuclear localization signals, 1	*Samsn1*	1,48801928	0,00117374
Zinc finger protein 207	*Zfp207*	1,48765591	0,00229536
Coiled-coil domain containing 91	*Ccdc91*	1,48719093	0,00102676
Inversin	*Invs*	1,48704953	0,00372573
Aldehyde dehydrogenase 4 family, member a1	*Aldh4a1*	1,48688702	0,00755877
Nadh dehydrogenase (ubiquinone) 1 alpha subcomplex, 6 (b14)	*Ndufa6*	1,48685057	0,00028526
Discoidin domain receptor family, member 2	*Ddr2*	1,48673088	0,00716128
Major vault protein	*Mvp*	1,48613027	0,00593625
Regulatory factor x, 2 (influences hla class ii expression)	*Rfx2*	1,48610232	8,98e-05

Myeloid leukemia factor 1	*Mlf1*	1,4857753	0,00129553
Zinc finger, dhhc domain containing 3	*Zdhhc3*	1,48565448	0,00016216
Peroxiredoxin 4	*Prdx4*	1,48562841	8,80e-05
Chromobox 5	*Cbx5*	1,48558556	0,00020624
Eh domain binding protein 1	*Ehbp1*	1,48537903	0,00103975
Methylmalonyl coa epimerase	*Mcee*	1,48515172	0,00193202
Elongation protein 4 homolog (s. Cerevisiae)	*Elp4*	1,48471679	0,00357193
Interleukin 15	*Il15*	1,48457447	0,00038701
Small integral membrane protein 14	*Smim14*	1,48443358	0,00011127
Unc-45 homolog a (c. Elegans)	*Unc45a*	1,48422612	0,00489058
Predicted gene, 20559	*Gm20559*	1,48384506	0,00035809
Nol1/nop2/sun domain family member 6	*Nsun6*	1,48372	0,00207626
Thioredoxin-like 4a	*Txnl4a*	1,48333232	0,00036257
Sry (sex determining region y)-box 13	*Sox13*	1,48327257	0,00489897
Gram domain containing 3	*Gramd3*	1,48298741	0,00044871
Solute carrier family 19 (thiamine transporter), member 2	*Slc19a2*	1,48297196	0,00042047
Rap2c, member of ras oncogene family	*Rap2c*	1,48250155	0,00256787
Mannosidase, alpha, class 2c, member 1	*Man2c1*	1,48189657	0,00137762
Guanine nucleotide binding protein (g protein), alpha inhibiting 2	*Gnai2*	1,48188006	0,00027249
Hexamethylene bis-acetamide inducible 1	*Hexim1*	1,4816773	0,00028726
Gpi anchor attachment protein 1	*Gpaa1*	1,48129856	0,00312981
Protein phosphatase 1, regulatory (inhibitor) subunit 7	*Ppp1r7*	1,4811363	0,0001788
N-glycanase 1	*Ngly1*	1,48103238	0,00179724
Ww domain containing e3 ubiquitin protein ligase 2	*Wwp2*	1,48017554	0,00807197
Nipsnap homolog 3b (c. Elegans)	*Nipsnap3b*	1,47986766	0,00243507
Mitochondrial ribosomal protein l4	*Mrpl4*	1,47968117	0,0005427
Cell division cycle 42	*Cdc42*	1,47953076	0,00316303
Lamin a	*Lmna*	1,47943191	0,00041623
Myosin xix	*Myo19*	1,47868775	0,0009005
Nucleoporin 155	*Nup155*	1,47842323	0,00397872
A disintegrin and metallopeptidase domain 22	*Adam22*	1,4780841	0,00483075
Hairy and enhancer of split 6	*Hes6*	1,47804364	0,00206152
Histone deacetylase 9	*Hdac9*	1,47788875	0,00554966
Epithelial membrane protein 1	*Emp1*	1,47756667	0,00093585
Early endosome antigen 1	*Eea1*	1,47742883	0,0004454
Comm domain containing 5	*Commd5*	1,47700374	0,00369785
Isocitrate dehydrogenase 3 (nad+) alpha	*Idh3a*	1,47691189	0,00176213
Amyloid beta (a4) precursor protein-binding, family a, member 3	*Apba3*	1,4764386	0,00331408
Riken cdna 3110057o12 gene	*3110057o12rik*	1,47633045	0,00234929
Inositol polyphosphate-5-phosphatase e	*Inpp5e*	1,47583011	0,0014144
Unc-119 homolog b (c. Elegans)	*Unc119b*	1,4757359	0,00025259
Slit-robo rho gtpase activating protein 3	*Srgap3*	1,47509291	0,00019779
Phosphoprotein enriched in astrocytes 15a	*Pea15a*	1,47352737	9,82e-05

Beta-carotene oxygenase 2	*Bco2*	1,4733778	0,00141302
Elastin microfibril interfacer 1	*Emilin1*	1,47291897	0,00861529
Calmodulin 1 /// calmodulin 2 /// calmodulin 3	*Calm1 /// calm2 /// calm3*	1,47195344	0,0013555
Cysteine rich transmembrane bmp regulator 1 (chordin like)	*Crim1*	1,47152102	0,00215774
Solute carrier family 6 (neurotransmitter transporter), member 17	*Slc6a17*	1,47116461	0,00327286
Von willebrand factor a domain containing 5a	*Vwa5a*	1,47111564	0,00515749
Golgi autoantigen, golgin subfamily a, 3	*Golga3*	1,47098923	0,00058519
Golgi autoantigen, golgin subfamily a, 2	*Golga2*	1,47070856	0,0006033
Polyglutamine binding protein 1	*Pqbp1*	1,47063538	0,00015629
Secretory carrier membrane protein 3	*Scamp3*	1,47049368	8,86e-05
Map/microtubule affinity-regulating kinase 4	*Mark4*	1,47037907	0,00874848
Antizyme inhibitor 1	*Azin1*	1,46998069	0,00916981
Cap-gly domain containing linker protein family, member 4	*Clip4*	1,46961116	0,00056579
Snap-associated protein	*Snapin*	1,46845694	0,00017938
Gamma-glutamyl cyclotransferase	*Ggct*	1,46842303	0,00160661
Slit-robo rho gtpase activating protein 2	*Srgap2*	1,467885	0,0003649
Tetraspanin 6	*Tspan6*	1,4677141	0,00540521
Thymus, brain and testes associated	*Tbata*	1,46741768	0,00122456
Ankyrin repeat domain 46	*Ankrd46*	1,46708564	0,0003646
Mitochondrial ribosomal protein l13	*Mrpl13*	1,4667048	0,00010839
St3 beta-galactoside alpha-2,3-sialyltransferase 5	*St3gal5*	1,46656387	0,00231111
Riken cdna 4933413g19 gene /// forkhead box m1 /// phosphatidylethanolamine binding protein 1	*4933413g19rik /// foxm1 /// pebp1*	1,46649853	0,00632241
Cysteine and glycine-rich protein 1	*Csrp1*	1,46638523	0,00902664
Adp-ribosylation factor-like 1	*Arl1*	1,46616856	0,00022249
Endonuclease/exonuclease/phosphatase family domain containing 1	*Eepd1*	1,46497071	0,00277875
Arginyl aminopeptidase (aminopeptidase b)	*Rnpep*	1,46466251	0,00321232
Rap2b, member of ras oncogene family	*Rap2b*	1,46454322	0,00276494
Cd274 antigen	*Cd274*	1,46453378	0,00216391
Cadherin 4	*Cdh4*	1,46357778	0,00033453
Adp-ribosylation factor 3	*Arf3*	1,46319091	0,00857204
Riken cdna 0610010k14 gene /// ribonuclease, rnase k	*0610010k14rik /// rnasek*	1,46316667	0,00209576
Zinc finger protein 239	*Zfp239*	1,46302801	0,00034852
Breakpoint cluster region	*Bcr*	1,46265206	0,00207658
Rab4b, member ras oncogene family	*Rab4b*	1,46262379	0,00160754
Adducin 1 (alpha)	*Add1*	1,46248048	0,00332087
Mitochondrial ribosomal protein s34 /// nme/nm23 nucleoside diphosphate kinase 3	*Mrps34 /// nme3*	1,46225466	0,00194947
Transmembrane and coiled-coil domains 1	*Tmco1*	1,46176892	0,00932947
Inositol polyphosphate-5-phosphatase a	*Inpp5a*	1,46168575	0,00103214
Phosphatidylinositol glycan anchor biosynthesis, class k	*Pigk*	1,46108827	0,00826967

Adducin 2 (beta)	*Add2*	1,46097709	0,00334606
Phosducin-like 3	*Pdcl3*	1,46094355	0,00684954
5'-3' exoribonuclease 2	*Xrn2*	1,46083013	0,00130841
Transcription factor 3	*Tcf3*	1,46079715	0,00208449
Sorting nexin 29	*Snx29*	1,46026932	0,00041015
Nadh dehydrogenase (ubiquinone) 1 beta subcomplex, 10	*Ndufb10*	1,45916375	0,00114075
Hermansky-pudlak syndrome 3 homolog (human)	*Hps3*	1,45889872	0,00117562
Signal peptide peptidase like 2a	*Sppl2a*	1,45845918	0,00225228
E2f transcription factor 8	*E2f8*	1,45822979	0,00216148
Nadh dehydrogenase (ubiquinone) 1 alpha subcomplex, 8	*Ndufa8*	1,45797426	0,00022107
Kinesin family member c2	*Kifc2*	1,45794566	0,00102868
Exportin, trna (nuclear export receptor for trnas)	*Xpot*	1,45787907	0,00275666
Galactose-4-epimerase, udp	*Gale*	1,45752844	0,0002072
Calpastatin	*Cast*	1,4574606	0,00053484
Zinc finger protein 954	*Zfp954*	1,45718211	0,00122557
Udp-glucuronate decarboxylase 1	*Uxs1*	1,45708911	0,00049667
Cystatin e/m	*Cst6*	1,4570805	0,00609317
Nucleosome assembly protein 1-like 4	*Nap1l4*	1,45677447	7,77e-05
Vaccinia related kinase 2	*Vrk2*	1,45630385	0,0073124
Glucosamine (udp-n-acetyl)-2-epimerase/n-acetylmannosamine kinase	*Gne*	1,45609108	0,00461472
Catalase	*Cat*	1,4560884	0,00157302
Solute carrier family 23 (nucleobase transporters), member 1	*Slc23a1*	1,45569567	0,00417435
Zinc finger, miz-type containing 2	*Zmiz2*	1,45566465	0,00244918
Nmda receptor synaptonuclear signaling and neuronal migration factor	*Nsmf*	1,45541013	0,00088927
Lon peptidase 2, peroxisomal	*Lonp2*	1,4553374	0,00042872
Biliverdin reductase a	*Blvra*	1,45525395	0,00032306
Nuclear receptor subfamily 1, group d, member 1	*Nr1d1*	1,45444072	0,0039557
Immediate early response 5-like	*Ier5l*	1,45434254	0,00451918
Tetratricopeptide repeat domain 28	*Ttc28*	1,45422489	0,00705773
Arp2 actin-related protein 2	*Actr2*	1,45327475	0,00049011
Lyr motif containing 5	*Lyrm5*	1,45276736	0,00957156
Calmodulin binding transcription activator 2	*Camta2*	1,45273559	0,00071397
Jumonji domain containing 8	*Jmjd8*	1,45271609	0,00216276
Cysteine-rich hydrophobic domain 1	*Chic1*	1,45268264	0,00744143
F-box protein 3	*Fbxo3*	1,45266606	0,00367729
Utp6, small subunit (ssu) processome component, homolog (yeast)	*Utp6*	1,4523173	0,00135608
Iscu iron-sulfur cluster scaffold homolog (e. Coli)	*Iscu*	1,45216671	0,00017988
Nfkb inhibitor interacting ras-like protein 1	*Nkiras1*	1,4520974	0,00220136
Kringle containing transmembrane protein 1	*Kremen1*	1,45141817	0,0012006
Ecto-nox disulfide-thiol exchanger 2	*Enox2*	1,45124272	0,00387873

Retinoic acid receptor responder (tazarotene induced) 2	*Rarres2*	1,45115522	0,00037394
Asparagine-linked glycosylation 14	*Alg14*	1,45108884	0,00113322
Cdc42 small effector 1	*Cdc42se1*	1,45085162	0,00246176
Solute carrier family 25 (mitochondrial carrier, brain), member 14	*Slc25a14*	1,45016422	0,00030249
Ethanolamine kinase 1	*Etnk1*	1,44995091	0,00019347
Splicing factor 3a, subunit 1	*Sf3a1*	1,44978372	0,00035001
Peptidylprolyl isomerase (cyclophilin)-like 2	*Ppil2*	1,44933612	0,00262568
Tho complex 1	*Thoc1*	1,44905242	0,0002289
Promyelocytic leukemia	*Pml*	1,44901755	0,0001371
Neural cell adhesion molecule 1	*Ncam1*	1,44882476	0,00730816
Selenocysteine lyase	*Scly*	1,44775426	0,00759188
Signal recognition particle 19	*Srp19*	1,44765885	0,00476996
Mitochondrial ribosomal protein s21	*Mrps21*	1,44728619	0,00054824
Pyruvate dehydrogenase e1 alpha 1	*Pdha1*	1,4465762	0,00075027
Inner membrane protein, mitochondrial	*Immt*	1,44645375	0,00172305
Udp-n-acetyl-alpha-d-galactosamine:polypeptide n-acetylgalactosaminyltransferase 4	*Galnt4*	1,4459022	0,00127219
Family with sequence similarity 220, member a	*Fam220a*	1,44584546	0,00151645
Diazepam binding inhibitor-like 5	*Dbil5*	1,44582568	0,00419233
Propionyl coenzyme a carboxylase, beta polypeptide	*Pccb*	1,44462917	0,0015054
Activating transcription factor 7 interacting protein	*Atf7ip*	1,44413368	0,00338831
Uhrf1 (icbp90) binding protein 1-like	*Uhrf1bp1l*	1,44402124	0,00046176
Coiled-coil domain containing 34	*Ccdc34*	1,44395427	0,00027783
Letm1 domain containing 1	*Letmd1*	1,44376082	0,00290619
High mobility group at-hook 2	*Hmga2*	1,44311714	0,00110924
Cullin associated and neddylation disassociated 1	*Cand1*	1,44309127	0,00059082
Twist basic helix-loop-helix transcription factor 1	*Twist1*	1,44263382	0,00084656
Potassium intermediate/small conductance calcium-activated channel, subfamily n, member 4	*Kcnn4*	1,44248026	0,0054838
Ptk2 protein tyrosine kinase 2 beta	*Ptk2b*	1,44238905	0,00531517
Annexin a7	*Anxa7*	1,44223828	0,00015449
Cytochrome c-1	*Cyc1*	1,44219671	0,00025706
Ubiquitin specific peptidase 15	*Usp15*	1,44210707	0,00036121
Mediator complex subunit 15	*Med15*	1,44180057	0,0006066
Paternally expressed 12	*Peg12*	1,44015447	0,00018611
Sorting nexin 16	*Snx16*	1,43961954	0,00079238
A disintegrin and metallopeptidase domain 5	*Adam5*	1,43930238	0,00122391
Kn motif and ankyrin repeat domains 2	*Kank2*	1,43912873	0,00701192
Dihydrolipoamide s-acetyltransferase (e2 component of pyruvate dehydrogenase complex)	*Dlat*	1,43905886	0,00265878
Splicing factor 3b, subunit 5	*Sf3b5*	1,43838816	0,00036659
Phosphatase domain containing, paladin 1	*Pald1*	1,43838614	0,00091433
Bicaudal d homolog 2 (drosophila)	*Bicd2*	1,43838603	0,00111011
Sam domain and hd domain, 1	*Samhd1*	1,43806853	0,00187372
Aly/ref export factor	*Alyref*	1,43794994	0,00447012

Rap guanine nucleotide exchange factor (gef) 1	*Rapgef1*	1,43762083	0,00156444
Set and mynd domain containing 2	*Smyd2*	1,43747187	0,00072924
Synaptotagmin iv	*Syt4*	1,43746326	0,00386725
Polymerase (dna directed), epsilon 2 (p59 subunit)	*Pole2*	1,43740828	0,00026891
Malate dehydrogenase 1, nad (soluble)	*Mdh1*	1,43720571	0,00063325
Upf3 regulator of nonsense transcripts homolog b (yeast)	*Upf3b*	1,43673401	0,00033899
Archaelysin family metallopeptidase 2	*Amz2*	1,43641677	0,00194351
Atpase, h+ transporting, lysosomal v1 subunit c1	*Atp6v1c1*	1,43620914	0,00159899
Syntaxin 4a (placental)	*Stx4a*	1,43610973	0,00396025
Absent in melanoma 2	*Aim2*	1,43610743	0,00793365
Interferon activated gene 205 /// myeloid cell nuclear differentiation antigen	*Ifi205 /// mnda*	1,43581338	0,00050524
Actinin, alpha 1	*Actn1*	1,43525274	0,0002485
Solute carrier family 9 (sodium/hydrogen exchanger), member 8	*Slc9a8*	1,43491183	0,00186068
Makorin, ring finger protein, 1	*Mkrn1*	1,43469509	0,00268036
Eukaryotic translation initiation factor 4b	*Eif4b*	1,43453951	0,0002843
Mitogen-activated protein kinase kinase 3, opposite strand	*Map2k3os*	1,43452822	0,00030723
Biliverdin reductase b (flavin reductase (nadph))	*Blvrb*	1,43431045	0,00238834
Harvey rat sarcoma oncogene, subgroup r	*Rras*	1,43391177	0,00100833
Rogdi homolog (drosophila)	*Rogdi*	1,43303648	0,0011018
Exocyst complex component 6 /// sh3-domain grb2-like b1 (endophilin)	*Exoc6 /// sh3glb1*	1,43300133	0,00395224
Histone deacetylase 3	*Hdac3*	1,43291613	0,00013401
Mitochondrial ribosomal protein l23	*Mrpl23*	1,43249517	0,001088
Ankyrin repeat domain 24	*Ankrd24*	1,43237786	0,00108107
Solute carrier family 4 (anion exchanger), member 2	*Slc4a2*	1,43233699	0,00097122
Polymerase (dna-directed), delta 4	*Pold4*	1,43183985	0,0007317
Nudix (nucleoside diphosphate linked moiety x)-type motif 5	*Nudt5*	1,43180926	0,00210005
Rab5b, member ras oncogene family	*Rab5b*	1,43076485	0,00744219
Tetratricopeptide repeat domain 7b	*Ttc7b*	1,4301402	0,00756216
Cyclin d3	*Ccnd3*	1,42961817	0,0004965
Guanine nucleotide binding protein (g protein), beta 2	*Gnb2*	1,42952248	0,00070838
Excision repair cross-complementing rodent repair deficiency, complementation group 5	*Ercc5*	1,4295156	0,0054838
Nuf2, ndc80 kinetochore complex component, homolog (s. Cerevisiae)	*Nuf2*	1,42900626	0,00187049
Oral cancer overexpressed 1	*Oraov1*	1,42732326	0,00057714
Ubiquitin-conjugating enzyme e2e 3	*Ube2e3*	1,42730368	0,00032207
Charged multivesicular body protein 3	*Chmp3*	1,42708065	0,00256367
Family with sequence similarity 222, member b	*Fam222b*	1,42634411	0,00461472
Autophagy related 5	*Atg5*	1,42626862	0,00908386
Transmembrane protein 189	*Tmem189*	1,42528057	0,00065916
Two pore segment channel 2	*Tpcn2*	1,42515972	0,00101441
Run and fyve domain containing 1	*Rufy1*	1,42512396	0,00065173

Tubulin, gamma complex associated protein 2	*Tubgcp2*	1,42498233	0,0002118
4-nitrophenylphosphatase domain and non-neuronal snap25-like protein homolog 1 (c. Elegans)	*Nipsnap1*	1,42478266	0,00353489
Nadh dehydrogenase (ubiquinone) 1, subcomplex unknown, 1	*Ndufc1*	1,4241854	0,00063174
Protein phosphatase methylesterase 1	*Ppme1*	1,42368213	0,00034882
Ring finger protein 121	*Rnf121*	1,42272042	0,00372109
Actinin alpha 4	*Actn4*	1,42255943	0,00050928
Prostate tumor over expressed gene 1	*Ptov1*	1,42158471	0,00074952
Coiled-coil domain containing 28a	*Ccdc28a*	1,42135913	0,00326907
Protein tyrosine phosphatase, non-receptor type 5	*Ptpn5*	1,42119261	0,00861319
Riken cdna 1110038f14 gene	*1110038f14rik*	1,42025998	0,00038924
Interferon, alpha-inducible protein 27 like 2b	*Ifi27l2b*	1,4201493	0,00208223
Ring finger protein 123	*Rnf123*	1,41971684	0,00209576
Diacylglycerol kinase zeta	*Dgkz*	1,41960713	0,00417651
Integrin beta 4	*Itgb4*	1,41846706	0,00019337
Interleukin 15 receptor, alpha chain	*Il15ra*	1,41826389	0,00452999
Nadh dehydrogenase (ubiquinone) 1 beta subcomplex, 6	*Ndufb6*	1,41719792	0,00100326
Carnitine acetyltransferase	*Crat*	1,41697874	0,0040619
Phd finger protein 1	*Phf1*	1,41684758	0,00194351
Solute carrier family 25 (mitochondrial thiamine pyrophosphate carrier), member 19	*Slc25a19*	1,41643951	0,00522131
Chromatin accessibility complex 1	*Chrac1*	1,41598782	0,00028396
Rfng o-fucosylpeptide 3-beta-n-acetylglucosaminyltransferase	*Rfng*	1,41489966	0,00332683
E2f-associated phosphoprotein	*Eapp*	1,41479323	0,00480912
Otu deubiquitinase with linear linkage specificity	*Otulin*	1,41454927	0,00034909
Phosphatidic acid phosphatase type 2b	*Ppap2b*	1,41454126	0,00545386
Homeobox a7	*Hoxa7*	1,41411821	0,0041875
Cts telomere maintenance complex component 1	*Ctc1*	1,41398449	0,00057875
Uridine phosphorylase 1	*Upp1*	1,41332506	0,00571562
Tensin like c1 domain-containing phosphatase	*Tenc1*	1,41321493	0,0034564
Cytidine monophospho-n-acetylneuraminic acid synthetase	*Cmas*	1,41285927	0,00098755
Alpha-kinase 2	*Alpk2*	1,41179767	0,00840206
Thioredoxin domain containing 16	*Txndc16*	1,41156997	0,00455118
Dynamin 2	*Dnm2*	1,41130257	0,00217352
Calcineurin-like ef hand protein 1 /// predicted gene, 20056	*Chp1 /// gm20056*	1,4109868	0,00054421
Phospholipase a2 receptor 1	*Pla2r1*	1,41098334	0,00113342
M-phase phosphoprotein 8	*Mphosph8*	1,41096656	0,00021552
Coiled-coil-helix-coiled-coil-helix domain containing 6	*Chchd6*	1,41025217	0,00091504
Peptidase (mitochondrial processing) beta	*Pmpcb*	1,41009698	0,00837355
Swi5 recombination repair homolog (yeast)	*Swi5*	1,41002447	0,00055154
Glutathione synthetase	*Gss*	1,40993447	0,00044441
Succinate dehydrogenase complex, subunit c, integral membrane protein	*Sdhc*	1,40936515	0,00948976

Rab22a, member ras oncogene family	*Rab22a*	1,40934988	0,00266679
Riken cdna 1110032a03 gene	*1110032a03rik*	1,40929816	0,00965065
Membrane protein, palmitoylated	*Mpp1*	1,40916671	0,0009297
Transmembrane bax inhibitor motif containing 6	*Tmbim6*	1,40882736	0,00206478
Ranbp-type and c3hc4-type zinc finger containing 1	*Rbck1*	1,40848437	0,00111861
Replication termination factor 2 domain containing 1	*Rtfdc1*	1,40843416	0,00370325
Urocanase domain containing 1	*Uroc1*	1,40800219	0,00287133
Single immunoglobulin and toll-interleukin 1 receptor (tir) domain	*Sigirr*	1,4078644	0,00104701
Ribosomal protein l22 like 1	*Rpl22l1*	1,40771649	0,00043619
Ectonucleoside triphosphate diphosphohydrolase 6	*Entpd6*	1,40754894	0,00816157
Ctd (carboxy-terminal domain, rna polymerase ii, polypeptide a) small phosphatase 2	*Ctdsp2*	1,40702567	0,00034472
Apaf1 interacting protein	*Apip*	1,40700295	0,00061214
Cdna sequence bc003331	*Bc003331*	1,40699358	0,00246805
Pleckstrin homology domain containing, family a member 5	*Plekha5*	1,40678845	0,0017047
Nima (never in mitosis gene a)-related expressed kinase 4	*Nek4*	1,40666097	0,00367043
Acyl-coa thioesterase 9	*Acot9*	1,40648325	0,00028982
Acyl-coa thioesterase 10 /// acyl-coa thioesterase 9	*Acot10 /// acot9*	1,40647802	0,00023627
Adp-ribosylation factor 1	*Arf1*	1,4064747	0,00991416
Riken cdna 2310067b10 gene	*2310067b10rik*	1,40628627	0,00087096
Glutathione peroxidase 4	*Gpx4*	1,40612972	0,00044193
Phosphate cytidylyltransferase 1, choline, alpha isoform	*Pcyt1a*	1,4052581	0,00309698
Enhancer of zeste homolog 2 (drosophila)	*Ezh2*	1,40491517	0,00133725
Transmembrane 7 superfamily member 3	*Tm7sf3*	1,40383631	0,00978522
Cut-like homeobox 1	*Cux1*	1,40377615	0,00645718
Protein phosphatase 6, catalytic subunit	*Ppp6c*	1,40367012	0,00163784
Apoptosis enhancing nuclease	*Aen*	1,40332881	0,0017522
Scm-like with four mbt domains 1	*Sfmbt1*	1,40280077	0,00201029
Uridine-cytidine kinase 1	*Uck1*	1,40263139	0,00071934
Ino80 complex subunit b	*Ino80b*	1,40236681	0,00850425
Translocase of inner mitochondrial membrane 13	*Timm13*	1,40232848	0,00037487
Solute carrier family 25 (mitochondrial carrier, phosphate carrier), member 3	*Slc25a3*	1,4021832	0,00033453
Tcdd-inducible poly(adp-ribose) polymerase	*Tiparp*	1,40189698	0,00509205
Lyr motif containing 2	*Lyrm2*	1,40175012	0,00183536
Anaphase prompoting complex c subunit 15	*Anapc15*	1,40142662	0,00074953
Angiopoietin 4	*Angpt4*	1,40127708	0,00546102
Phosphomannomutase 2	*Pmm2*	1,40058161	0,00151382
Solute carrier family 22, member 23	*Slc22a23*	1,40033509	0,00076599
Kinesin family member 3c	*Kif3c*	1,40024883	0,00158988
Fanconi anemia, complementation group g	*Fancg*	1,4001991	0,00239707
Rab3a, member ras oncogene family	*Rab3a*	1,39979434	0,00640062
Rna binding motif protein, x-linked 2	*Rbmx2*	1,39972227	0,00461661

Fanconi anemia, complementation group i	*Fanci*	1,39889942	0,00083754
Dna methyltransferase (cytosine-5) 1	*Dnmt1*	1,39865817	0,0009069
Kelch-like 7	*Klhl7*	1,39863619	0,00545623
Adp-ribosylation factor gtpase activating protein 2	*Arfgap2*	1,39850167	0,00155094
Brick1, scar/wave actin-nucleating complex subunit	*Brk1*	1,39818946	0,00108332
Ww domain binding protein 1	*Wbp1*	1,39714449	0,00096564
Cdkn1a interacting zinc finger protein 1	*Ciz1*	1,39682297	0,0010691
Bromodomain adjacent to zinc finger domain, 2a	*Baz2a*	1,39550415	0,00058301
Vinculin	*Vcl*	1,39512793	0,00155039
Glial cells missing homolog 1 (drosophila)	*Gcm1*	1,39423498	0,00730718
Mif4g domain containing	*Mif4gd*	1,39395153	0,00229516
Proteasome (prosome, macropain) 26s subunit, non-atpase, 11	*Psmd11*	1,3938757	0,00170082
Transmembrane protein 205	*Tmem205*	1,39325894	0,00160305
Tumor suppressor candidate 2	*Tusc2*	1,39321934	0,00081765
Aminoacylase 1	*Acy1*	1,39318132	0,0032524
Transcription elongation factor a (sii)-like 1	*Tceal1*	1,39302116	0,00433875
Coiled-coil domain containing 65	*Ccdc65*	1,39216663	0,00092713
Tetratricopeptide repeat domain 4	*Ttc4*	1,39211688	0,00305901
Transmembrane protein 30a	*Tmem30a*	1,39211436	0,00684497
Trinucleotide repeat containing 6b	*Tnrc6b*	1,391747	0,00229536
Calcium and integrin binding 1 (calmyrin)	*Cib1*	1,39168907	0,00149434
Neuronal guanine nucleotide exchange factor	*Ngef*	1,39140195	0,0007951
Ribonucleotide reductase m1	*Rrm1*	1,39131994	0,00049506
Myb-related transcription factor, partner of profilin	*Mypop*	1,39120526	0,00257661
Dead (asp-glu-ala-asp) box polypeptide 41	*Ddx41*	1,39076897	0,00209686
Udp-n-acetyl-alpha-d-galactosamine:polypeptide n-acetylgalactosaminyltransferase 10	*Galnt10*	1,39040007	0,00072372
Yme1-like 1 (s. Cerevisiae)	*Yme1l1*	1,38991556	0,00643418
Vaccinia related kinase 3	*Vrk3*	1,38969156	0,00071821
Translin	*Tsn*	1,38961525	0,0094721
Erythroid differentiation regulatory factor 1	*Edrf1*	1,38936696	0,0021739
Dynactin 5	*Dctn5*	1,38901779	0,00208683
Tbc1 domain family, member 1	*Tbc1d1*	1,38896717	0,00790114
Family with sequence similarity 120, member b	*Fam120b*	1,3884743	0,0019914
Glycerophosphodiester phosphodiesterase domain containing 5	*Gdpd5*	1,38836646	0,00169161
Udp-n-acetylglucosamine pyrophosphorylase 1	*Uap1*	1,38830227	0,00088534
Rho guanine nucleotide exchange factor (gef) 25	*Arhgef25*	1,38820757	0,00784434
Traf type zinc finger domain containing 1	*Trafd1*	1,38786681	0,00315345
Signal recognition particle 14	*Srp14*	1,38779758	0,00061538
Roundabout homolog 3 (drosophila)	*Robo3*	1,38763023	0,0064082
Lim and senescent cell antigen-like domains 1	*Lims1*	1,38752145	0,00241841
Coagulation factor x	*F10*	1,38746848	0,00349155
Ubiquitin protein ligase e3 component n-recognin 1	*Ubr1*	1,38680177	0,00705404
N-methylpurine-dna glycosylase	*Mpg*	1,38636454	0,00265497

Nima (never in mitosis gene a)-related expressed kinase 8	*Nek8*	1,38601097	0,00159378
General transcription factor iiic, polypeptide 5	*Gtf3c5*	1,38568016	0,00137428
Nadh dehydrogenase (ubiquinone) fe-s protein 1	*Ndufs1*	1,38560187	0,00436043
Solute carrier family 35, member a5	*Slc35a5*	1,38555214	0,00251818
Aldo-keto reductase family 1, member e1	*Akr1e1*	1,38513517	0,00665432
Cytochrome p450, family 2, subfamily j, polypeptide 9	*Cyp2j9*	1,38488318	0,00083356
Membrane-associated ring finger (c3hc4) 6	*Iii.06*	1,38487476	0,00139968
Cdna sequence bc048403	*Bc048403*	1,38459705	0,00414133
Mitochondrial ribosomal protein l14	*Mrpl14*	1,38425769	0,00230272
Serine/threonine kinase 25 (yeast)	*Stk25*	1,38405497	0,0002762
Riken cdna 1700071k01 gene /// prohibitin	*1700071k01rik /// phb*	1,3836545	0,00132066
Myotubularin related protein 6	*Mtmr6*	1,38351383	0,00418584
Rna binding motif protein 14	*Rbm14*	1,3834749	0,00091066
Protein phosphatase 2a activator, regulatory subunit b	*Ppp2r4*	1,38317283	0,00894206
Dcp1 decapping enzyme homolog a (s. Cerevisiae)	*Dcp1a*	1,38317257	0,00043644
Wd repeat, sam and u-box domain containing 1	*Wdsub1*	1,38287548	0,00212222
Protein kinase, interferon inducible double stranded rna dependent activator	*Prkra*	1,38280798	0,00137052
Tubulin polyglutamylase complex subunit 1	*Tpgs1*	1,38267575	0,00450375
Interferon-induced protein with tetratricopeptide repeats 2	*Ifit2*	1,38261212	0,00055305
Cysteine-rich with egf-like domains 2	*Creld2*	1,3825009	0,00145352
Tlc domain containing 1	*Tlcd1*	1,38230248	0,00414834
Transcription factor a, mitochondrial	*Tfam*	1,38227323	0,00162368
Unc-51 like kinase 2	*Ulk2*	1,38137025	0,00083745
Siva1, apoptosis-inducing factor	*Siva1*	1,38122421	0,00075475
Zinc finger protein 219	*Zfp219*	1,38072173	0,00539899
Mitochondrial ribosomal protein s11	*Mrps11*	1,38045013	0,0020947
Phd finger protein 19	*Phf19*	1,38026702	0,00236016
Tubulin alpha, related sequence 1	*Tuba-rs1*	1,37995247	0,00993568
Swi/snf related, matrix associated, actin dependent regulator of chromatin, subfamily d, member 1	*Smarcd1*	1,37955582	0,00505576
Phosphatidic acid phosphatase type 2c	*Ppap2c*	1,37926776	0,00043012
Actin related protein 2/3 complex, subunit 5	*Arpc5*	1,37908837	0,00062801
Atp synthase, h+ transporting, mitochondrial f0 complex, subunit f2	*Atp5j2*	1,37902382	0,00293797
Integrator complex subunit 4	*Ints4*	1,37897349	0,0007385
Pleiotropic regulator 1, prl1 homolog (arabidopsis)	*Plrg1*	1,37897083	0,00056158
Lim and sh3 protein 1	*Lasp1*	1,37840586	0,00442356
Cytohesin 1	*Cyth1*	1,37807769	0,00181778
Methyl cpg binding protein 2	*Mecp2*	1,37796884	0,00919691
Heat repeat containing 6	*Heatr6*	1,37789531	0,00188045
Dehydrogenase/reductase (sdr family) member 7	*Dhrs7*	1,37789453	0,00101415
Riken cdna 4930429b21 gene	*4930429b21rik*	1,37774055	0,00719036
Zinc finger protein 386 (kruppel-like)	*Zfp386*	1,37757669	0,00078296

6-phosphofructo-2-kinase/fructose-2,6-biphosphatase 2	*Pfkfb2*	1,37639169	0,00293871
Steroid 5 alpha-reductase 3	*Srd5a3*	1,37637351	0,00155912
Major facilitator superfamily domain containing 1	*Mfsd1*	1,37571693	0,00146859
Centromere protein h	*Cenph*	1,37568097	0,00902867
Ecsit homolog (drosophila)	*Ecsit*	1,37565957	0,00490418
Dnaj (hsp40) homolog, subfamily b, member 1	*Dnajb1*	1,37563215	0,00209021
Zinc finger protein 277	*Zfp277*	1,37557802	0,00669478
Tsr2 20s rrna accumulation	*Tsr2*	1,37534164	0,0007097
Ataxin 2	*Atxn2*	1,37524345	0,00397883
Rex4, rna exonuclease 4 homolog (s. Cerevisiae)	*Rexo4*	1,37471851	0,00337182
Kelch-like 5	*Klhl5*	1,3741974	0,0009318
Ras, dexamethasone-induced 1	*Rasd1*	1,37397714	0,00131526
Glucose 6 phosphatase, catalytic, 3	*G6pc3*	1,37391657	0,00134672
Ubiquitin specific peptidase 5 (isopeptidase t)	*Usp5*	1,37359661	0,00178235
Protein phosphatase 4, regulatory subunit 2	*Ppp4r2*	1,37329233	0,00181878
Wolfram syndrome 1 homolog (human)	*Wfs1*	1,37292037	0,00036012
Cystatin c	*Cst3*	1,37287395	0,00046444
Vacuolar protein sorting 26 homolog b (yeast)	*Vps26b*	1,37277787	0,00304946
Pleckstrin and sec7 domain containing 3	*Psd3*	1,3724352	0,00083665
Lysophospholipase-like 1	*Lyplal1*	1,37232447	0,00377633
Proteasome maturation protein	*Pomp*	1,37228515	0,00068269
Polymerase (dna directed), iota	*Poli*	1,37170622	0,00279345
Hiv tat specific factor 1	*Htatsf1*	1,37166932	0,00168454
Ring finger protein 126	*Rnf126*	1,37133811	0,00325893
F-box protein 18	*Fbxo18*	1,37091266	0,00048274
Secretory blood group 1	*Sec1*	1,37060505	0,00620543
Helicase-like transcription factor	*Hltf*	1,37053851	0,00408454
Comm domain containing 9	*Commd9*	1,37029876	0,00089917
Slowmo homolog 2 (drosophila)	*Slmo2*	1,36990542	0,00695992
Flavin containing monooxygenase 5	*Fmo5*	1,36976202	0,00548828
E2f transcription factor 1	*E2f1*	1,36962757	0,00316084
Tropomyosin 3, gamma	*Tpm3*	1,36958304	0,00146729
Deformed epidermal autoregulatory factor 1 (drosophila)	*Deaf1*	1,36935502	0,00865533
Sar1 gene homolog b (s. Cerevisiae)	*Sar1b*	1,36932636	0,00469524
Adnp homeobox 2	*Adnp2*	1,36882199	0,00359623
Galactose-1-phosphate uridyl transferase	*Galt*	1,36844456	0,00723007
Kit ligand	*Kitl*	1,36826627	0,00496122
Protein phosphatase 6, regulatory subunit 2	*Ppp6r2*	1,36814094	0,00084701
N-myc downstream regulated gene 4	*Ndrg4*	1,36792001	0,00120479
Small g protein signaling modulator 3	*Sgsm3*	1,36778178	0,00032257
Forkhead box m1	*Foxm1*	1,36770485	0,00306147
Nadh dehydrogenase (ubiquinone) 1 alpha subcomplex, 12	*Ndufa12*	1,36757012	0,000649
Exocyst complex component 4	*Exoc4*	1,36742331	0,00151745

Brca1 associated protein	*Brap*	1,36742147	0,00737763
Macrophage stimulating 1 (hepatocyte growth factor-like)	*Mst1*	1,36696358	0,00154495
Vesicle-associated membrane protein 4	*Vamp4*	1,36660313	0,00951649
E2f transcription factor 5	*E2f5*	1,36612002	0,00787595
Extended synaptotagmin-like protein 1	*Esyt1*	1,36609986	0,00090769
Centriole, cilia and spindle associated protein	*Ccsap*	1,36609714	0,00261462
Nadh dehydrogenase (ubiquinone) 1 alpha subcomplex, 9	*Ndufa9*	1,36466075	0,00063215
Nadh dehydrogenase [ubiquinone] 1 subunit c2-like /// nadh dehydrogenase (ubiquinone) 1, subcomplex unknown, 2	*Loc102641347 /// ndufc2*	1,36455483	0,00351512
Golgi snap receptor complex member 1	*Gosr1*	1,36451589	0,00762965
Mitochondrial ribosomal protein l48	*Mrpl48*	1,36440761	0,00176029
Tocopherol (alpha) transfer protein	*Ttpa*	1,36365438	0,0015582
Branched chain ketoacid dehydrogenase kinase	*Bckdk*	1,36349627	0,00066855
Mevalonate (diphospho) decarboxylase	*Mvd*	1,36327331	0,00958593
Dopamine receptor d4	*Drd4*	1,36320956	0,00739845
Glutamic acid decarboxylase 2	*Gad2*	1,36309822	0,0005387
Protein inhibitor of activated stat 2	*Pias2*	1,36282228	0,00287183
Nuclear mitotic apparatus protein 1	*Numa1*	1,36275661	0,00151976
Fibroblast growth factor 18	*Fgf18*	1,36256269	0,00350918
Solute carrier family 39 (metal ion transporter), member 13	*Slc39a13*	1,36230089	0,00383471
Histone deacetylase 7	*Hdac7*	1,3622439	0,00667587
Phosphorylase kinase gamma 1	*Phkg1*	1,36156489	0,00275636
Mitochondrial ribosomal protein l54	*Mrpl54*	1,36142005	0,00294111
Interferon gamma receptor 1	*Ifngr1*	1,36028939	0,00049367
Inositol polyphosphate-4-phosphatase, type i	*Inpp4a*	1,36015935	0,00431617
Cdc42 binding protein kinase alpha	*Cdc42bpa*	1,36013135	0,0025466
Calcium/calmodulin-dependent protein kinase kinase 2, beta	*Camkk2*	1,36010419	0,00522944
Polymerase (dna-directed), delta interacting protein 3	*Poldip3*	1,35994535	0,00919995
Atpase, h+ transporting, lysosomal v1 subunit e1	*Atp6v1e1*	1,35979919	0,00169947
Cyclic nucleotide gated channel alpha 1	*Cnga1*	1,35959561	0,00498541
Ubiquitin associated domain containing 2	*Ubac2*	1,35892089	0,00099736
Dna primase, p58 subunit	*Prim2*	1,35886054	0,000646
Translocase of outer mitochondrial membrane 40 homolog-like (yeast)	*Tomm40l*	1,35783722	0,00197087
Sjogren's syndrome nuclear autoantigen 1	*Ssna1*	1,35762472	0,00048735
Microsomal glutathione s-transferase 2	*Mgst2*	1,35645392	0,0029667
Rab5c, member ras oncogene family	*Rab5c*	1,35631073	0,00298464
V-ral simian leukemia viral oncogene homolog a (ras related)	*Rala*	1,3557392	0,00328571
Translocated promoter region, nuclear basket protein	*Tpr*	1,35542004	0,00232105
Matrix metallopeptidase 17	*Mmp17*	1,35511799	0,0006706
Loricrin	*Lor*	1,35507516	0,00433239
Jade family phd finger 1	*Jade1*	1,35501302	0,00907162

Numb gene homolog (drosophila)	*Numb*	1,35457364	0,00513645
Heat shock protein 4 like	*Hspa4l*	1,35449504	0,00554926
Expressed sequence c78339	*C78339*	1,35446772	0,00048972
Integrator complex subunit 1	*Ints1*	1,3544415	0,00631492
Ring finger protein 167	*Rnf167*	1,35436127	0,00161281
Integrator complex subunit 9	*Ints9*	1,35420816	0,00720264
Aly/ref export factor /// aly/ref export factor 2	*Alyref /// alyref2*	1,35391068	0,00991729
Cyclin-dependent kinase 1	*Cdk1*	1,35375872	0,00054365
M-phase phosphoprotein 9	*Mphosph9*	1,35375164	0,00342175
Riken cdna 9030624j02 gene	*9030624j02rik*	1,35363831	0,00066198
Rna binding motif protein 8a	*Rbm8a*	1,3535044	0,00114647
Ubiquinol-cytochrome c reductase hinge protein	*Uqcrh*	1,35348615	0,00229277
Peroxisomal biogenesis factor 16	*Pex16*	1,35344964	0,00403909
Histone deacetylase 2	*Hdac2*	1,35338565	0,00208514
Sec24 related gene family, member c (s. Cerevisiae)	*Sec24c*	1,35337559	0,00121397
Cysteine and histidine rich 1	*Cyhr1*	1,35272391	0,00115429
Trab domain containing	*Trabd*	1,35261697	0,00495352
Thymidine kinase 1	*Tk1*	1,35233501	0,00128481
Pyridoxine 5'-phosphate oxidase	*Pnpo*	1,35222326	0,00383943
Glutaredoxin 2 (thioltransferase)	*Glrx2*	1,35190944	0,00157516
Slu7 splicing factor homolog (s. Cerevisiae)	*Slu7*	1,35180518	0,00965603
General transcription factor iii a	*Gtf3a*	1,35156799	0,00683814
Fk506 binding protein 1a	*Fkbp1a*	1,35123539	0,00202396
Fk506 binding protein 7	*Fkbp7*	1,3511533	0,00169772
Fyve, rhogef and ph domain containing 3	*Fgd3*	1,35084287	0,00893378
Replication factor c (activator 1) 2	*Rfc2*	1,35076296	0,00077659
Stabilin 1	*Stab1*	1,35076286	0,00385116
Tubby-like protein 3	*Tulp3*	1,35065889	0,00463605
Polymerase (dna directed), delta 2, regulatory subunit	*Pold2*	1,3505106	0,00231451
Atp-binding cassette, sub-family c (cftr/mrp), member 2	*Abcc2*	1,35049612	0,00310114
Arp3 actin-related protein 3	*Actr3*	1,35031179	0,00174472
Armadillo repeat containing 1	*Armc1*	1,34931959	0,00261972
Sec14-like 1 (s. Cerevisiae)	*Sec14l1*	1,34919463	0,0039557
Ubiquitin-conjugating enzyme e2t (putative)	*Ube2t*	1,34910168	0,00358
Myoneurin	*Mynn*	1,34910121	0,00560969
Protein inhibitor of activated stat 1	*Pias1*	1,3490447	0,00267759
Erbb receptor feedback inhibitor 1	*Errfi1*	1,34883705	0,00108107
Mitochondrial ribosomal protein s5	*Mrps5*	1,3488024	0,00614249
Myotubularin related protein 14	*Mtmr14*	1,34837633	0,00491227
Vitamin k epoxide reductase complex, subunit 1	*Vkorc1*	1,34835613	0,00099306
Thioredoxin reductase 3	*Txnrd3*	1,34781735	0,00291285
Zinc finger protein 87	*Zfp87*	1,34770221	0,00325399
Acyl-coenzyme a dehydrogenase family, member 10	*Acad10*	1,34728184	0,00100314
Geminin	*Gmnn*	1,34720035	0,00168454

Cholinergic receptor, muscarinic 3, cardiac	*Chrm3*	1,34701646	0,0004727
Fasciculation and elongation protein zeta 2 (zygin ii)	*Fez2*	1,34654615	0,00050245
Sam and sh3 domain containing 1	*Sash1*	1,34653255	0,00335704
K(lysine) acetyltransferase 7	*Kat7*	1,34641156	0,0079804
Male-specific lethal 3 homolog (drosophila)	*Msl3*	1,3459993	0,00066855
Ctd (carboxy-terminal domain, rna polymerase ii, polypeptide a) phosphatase, subunit 1	*Ctdp1*	1,34580966	0,00935869
Nth (endonuclease iii)-like 1 (e.coli)	*Nthl1*	1,34580461	0,00112873
Cox assembly mitochondrial protein 2	*Cmc2*	1,34492314	0,00239109
Rab11a, member ras oncogene family	*Rab11a*	1,3447351	0,00796815
Germ cell-less homolog 1 (drosophila)	*Gmcl1*	1,3442875	0,00422609
Vps9 domain containing 1	*Vps9d1*	1,34412013	0,00206024
Mediator complex subunit 22	*Med22*	1,3438771	0,00331579
Nudix (nucleoside diphosphate linked moiety x)-type motif 16	*Nudt16*	1,34355991	0,00952734
Death associated protein 3	*Dap3*	1,34343057	0,00091545
Transcription factor cp2	*Tfcp2*	1,34310415	0,00238834
Ring finger protein 157	*Rnf157*	1,3428983	0,00317977
Cleavage and polyadenylation specific factor 1	*Cpsf1*	1,34280598	0,0030092
Trans-2,3-enoyl-coa reductase	*Tecr*	1,34275508	0,00418089
Nascent polypeptide-associated complex alpha polypeptide	*Naca*	1,34256173	0,00076395
Huntingtin	*Htt*	1,34227253	0,00395224
Mitofusin 1	*Mfn1*	1,34208324	0,00164862
Grpe-like 2, mitochondrial	*Grpel2*	1,34201422	0,00172363
Nitric oxide associated 1	*Noa1*	1,34150844	0,00191845
Mob family member 4, phocein	*Mob4*	1,34128785	0,00880243
Polymerase (dna directed), beta	*Polb*	1,34127114	0,00143088
Lipin 3	*Lpin3*	1,34093366	0,00425092
Coiled-coil-helix-coiled-coil-helix domain containing 5	*Chchd5*	1,34083338	0,00619686
Myotubularin related protein 4	*Mtmr4*	1,34075439	0,00079068
Fanconi anemia, complementation group l	*Fancl*	1,34045421	0,00281881
E2f transcription factor 6	*E2f6*	1,34023989	0,00207574
Nucleoporin 43	*Nup43*	1,34023074	0,00160935
Cysteine rich, dpf motif domain containing 1	*Cdpf1*	1,33969349	0,00177085
Patatin-like phospholipase domain containing 2	*Pnpla2*	1,33968449	0,00436988
Glucosidase, alpha, acid	*Gaa*	1,33954259	0,00374298
Vang-like 1 (van gogh, drosophila)	*Vangl1*	1,33929168	0,00236001
Solute carrier family 39 (metal ion transporter), member 11	*Slc39a11*	1,33899294	0,0017669
Ash2 (absent, small, or homeotic)-like (drosophila)	*Ash2l*	1,33895066	0,00112471
Predicted gene, 17748 /// peptidyl prolyl isomerase h	*Gm17748 /// ppih*	1,33888524	0,00291272
Glucosamine (n-acetyl)-6-sulfatase	*Gns*	1,33851297	0,00104274
Dual oxidase maturation factor 1	*Duoxa1*	1,33846107	0,00560969
Profilin 2	*Pfn2*	1,33833138	0,00553428
Suppressor of cytokine signaling 5	*Socs5*	1,33825698	0,00365674

Haus augmin-like complex, subunit 4	*Haus4*	1,3381848	0,00174963
Predicted gene 13152	*Gm13152*	1,33775971	0,00893981
Fatty acid binding protein 5, epidermal	*Fabp5*	1,33751313	0,00774416
Loss of heterozygosity, 12, chromosomal region 1 homolog (human)	*Loh12cr1*	1,33598528	0,00223573
Major facilitator superfamily domain containing 3	*Mfsd3*	1,33586296	0,00650951
Solute carrier family 14 (urea transporter), member 1	*Slc14a1*	1,33527427	0,00096682
Mitochondrial ribosomal protein l35	*Mrpl35*	1,33479738	0,00438536
Pdz binding kinase	*Pbk*	1,33415875	0,00327163
Growth factor receptor bound protein 2	*Grb2*	1,33376711	0,0007824
Enolase 2, gamma neuronal	*Eno2*	1,33363235	0,0076496
Ras-related c3 botulinum substrate 1	*Rac1*	1,3335692	0,00305103
Vacuolar protein sorting 26 homolog a (yeast)	*Vps26a*	1,33317146	0,00054258
Triosephosphate isomerase 1	*Tpi1*	1,33231031	0,00107379
Eukaryotic translation initiation factor 2b, subunit 4 delta	*Eif2b4*	1,33211782	0,00545462
Adenosine monophosphate deaminase 3	*Ampd3*	1,33144015	0,00336167
Mad2 mitotic arrest deficient-like 1	*Mad2l1*	1,33083298	0,00106554
Thyroid hormone receptor alpha	*Thra*	1,33069258	0,00742942
Mis18 binding protein 1	*Mis18bp1*	1,3306531	0,00268677
Eukaryotic translation initiation factor 4, gamma 2	*Eif4g2*	1,33011535	0,00253679
Arp10 actin-related protein 10	*Actr10*	1,3300581	0,00325893
Microtubule-associated protein 4	*Map4*	1,32987265	0,00366439
Sprouty homolog 4 (drosophila)	*Spry4*	1,32987102	0,00432788
Adp-ribosylation factor-like 4c	*Arl4c*	1,3297901	0,0010052
Fic domain containing	*Ficd*	1,32976609	0,00923632
Solute carrier family 4 (anion exchanger), member 1	*Slc4a1*	1,32974785	0,00124039
Atp synthase, h+ transporting mitochondrial f1 complex, beta subunit	*Atp5b*	1,32971081	0,00229228
Acireductone dioxygenase 1	*Adi1*	1,32939115	0,00278303
Tripartite motif-containing 62	*Trim62*	1,32871883	0,00259503
Zinc finger and btb domain containing 45	*Zbtb45*	1,32842264	0,00135598
Cytochrome p450, family 11, subfamily a, polypeptide 1	*Cyp11a1*	1,32836438	0,00705773
Molybdenum cofactor sulfurase	*Mocos*	1,32831756	0,00669456
Translocase of inner mitochondrial membrane 17a	*Timm17a*	1,32798196	0,00396966
Secernin 2	*Scrn2*	1,32792942	0,00377677
Small nuclear ribonucleoprotein 27 (u4/u6.u5)	*Snrnp27*	1,32769556	0,00254443
Er degradation enhancer, mannosidase alpha-like 1	*Edem1*	1,32751009	0,00061585
Mitochondrial fission regulator 1-like	*Mtfr1l*	1,32720058	0,00117801
Lsm14 homolog b (scd6, s. Cerevisiae)	*Lsm14b*	1,32685111	0,00228668
Nlr family, apoptosis inhibitory protein 2	*Naip2*	1,32679938	0,00299058
Catenin, beta like 1	*Ctnnbl1*	1,32652003	0,00131526
Asp (abnormal spindle)-like, microcephaly associated (drosophila)	*Aspm*	1,3261432	0,00397733
Hydroxy-delta-5-steroid dehydrogenase, 3 beta- and steroid delta-isomerase 7	*Hsd3b7*	1,32590643	0,00084461

Biphenyl hydrolase-like (serine hydrolase, breast epithelial mucin-associated antigen)	*Bphl*	1,32538214	0,00591573
Purine rich element binding protein a	*Pura*	1,32535293	0,00820166
N-acetylneuraminic acid synthase (sialic acid synthase)	*Nans*	1,32534911	0,0034774
Snf8, escrt-ii complex subunit, homolog (s. Cerevisiae)	*Snf8*	1,32514866	0,0041785
Dynactin 3	*Dctn3*	1,32506994	0,00370257
H2a histone family, member v	*H2afv*	1,32492264	0,0018271
Wingless-type mmtv integration site family, member 5a	*Wnt5a*	1,32488887	0,0099207
Tubulin, gamma complex associated protein 4	*Tubgcp4*	1,3247779	0,0018366
Aurora kinase a	*Aurka*	1,32441759	0,00935397
Speg complex locus	*Speg*	1,32395374	0,00985276
Cytochrome c oxidase subunit viia 2	*Cox7a2*	1,32378791	0,00382598
N-6 adenine-specific dna methyltransferase 2 (putative)	*N6amt2*	1,32347908	0,00801956
Nadh dehydrogenase (ubiquinone) fe-s protein 3	*Ndufs3*	1,32333657	0,0041785
Ath1, acid trehalase-like 1 (yeast)	*Athl1*	1,32258445	0,00969983
Glutamate-cysteine ligase, modifier subunit	*Gclm*	1,32189372	0,00195219
Serum response factor binding protein 1	*Srfbp1*	1,32185855	0,00231561
Leukocyte receptor cluster (lrc) member 1	*Leng1*	1,32164396	0,00717368
Abhydrolase domain containing 16a	*Abhd16a*	1,3212273	0,00349414
Spastic paraplegia 20, spartin (troyer syndrome) homolog (human)	*Spg20*	1,32117029	0,00446874
Mpv17 mitochondrial inner membrane protein	*Mpv17*	1,32113946	0,00367508
Acetyl-coenzyme a acetyltransferase 2	*Acat2*	1,32051603	0,00107596
Sec1 family domain containing 1	*Scfd1*	1,31978078	0,00237424
Sec61 alpha 1 subunit (s. Cerevisiae)	*Sec61a1*	1,31970046	0,00416432
Electron transferring flavoprotein, beta polypeptide	*Etfb*	1,31955255	0,00316737
Atx1 (antioxidant protein 1) homolog 1 (yeast)	*Atox1*	1,31936529	0,00467958
Leukotriene c4 synthase	*Ltc4s*	1,31895046	0,0063076
Suppressor of defective silencing 3 homolog (s. Cerevisiae)	*Suds3*	1,31866405	0,00234391
Tar (hiv) rna binding protein 2	*Tarbp2*	1,31843558	0,00264826
Atp citrate lyase	*Acly*	1,31837211	0,00541149
Ras p21 protein activator 4	*Rasa4*	1,31823741	0,00414834
Riken cdna 5031439g07 gene	*5031439g07rik*	1,31795033	0,0028693
Biotinidase	*Btd*	1,31787007	0,00464004
Histone deacetylase 6	*Hdac6*	1,31778492	0,00224586
Schlafen 3	*Slfn3*	1,31754778	0,00997966
Coiled-coil-helix-coiled-coil-helix domain containing 2 /// predicted gene 13202 /// zinc finger, bed-type containing 5	*Chchd2 /// gm13202 /// zbed5*	1,31717906	0,00148674
Lysosomal-associated protein transmembrane 4a	*Laptm4a*	1,31646317	0,00309332
Cdc28 protein kinase regulatory subunit 2	*Cks2*	1,31624628	0,00247621
Ww domain containing adaptor with coiled-coil	*Wac*	1,31610842	0,00700356
Glucose-6-phosphate dehydrogenase 2 /// glucose-6-phosphate dehydrogenase x-linked	*G6pd2 /// g6pdx*	1,31610015	0,00891248
Splicing factor 3b, subunit 6	*Sf3b6*	1,31599757	0,00373324

Family with sequence similarity 21	*Fam21*	1,31597099	0,00625833
Proteasome (prosome, macropain) subunit, alpha type 1	*Psma1*	1,31589089	0,00383738
Solute carrier family 15, member 4	*Slc15a4*	1,31571466	0,00525411
Paxip1 associated glutamate rich protein 1a	*Pagr1a*	1,31560683	0,00833993
Small edrk-rich factor 2	*Serf2*	1,31543784	0,00516913
Sodium channel, voltage-gated, type i, alpha	*Scn1a*	1,31531297	0,00176433
Sh3 binding domain protein 5 like	*Sh3bp5l*	1,31519177	0,00255329
Biogenesis of lysosomal organelles complex-1, subunit 6, pallidin	*Bloc1s6*	1,3149992	0,00322294
Developmentally regulated gtp binding protein 1	*Drg1*	1,31485765	0,00241931
Calcium-binding tyrosine-(y)-phosphorylation regulated (fibrousheathin 2)	*Cabyr*	1,31416494	0,0043725
Zinc finger with krab and scan domains 5	*Zkscan5*	1,31258515	0,0050304
Cystatin b	*Cstb*	1,3124178	0,00322944
Lipin 1	*Lpin1*	1,31199226	0,00371082
Nephronophthisis 1 (juvenile) homolog (human)	*Nphp1*	1,31189797	0,00626874
3-hydroxyisobutyrate dehydrogenase	*Hibadh*	1,31146759	0,00784359
Mrs2 magnesium homeostasis factor homolog (s. Cerevisiae)	*Mrs2*	1,31143055	0,0070202
Amyloid beta (a4) precursor protein-binding, family b, member 1	*Apbb1*	1,31113909	0,00362909
Acyl-coenzyme a oxidase 1, palmitoyl	*Acox1*	1,31088211	0,00104335
Occludin/ell domain containing 1	*Ocel1*	1,31064261	0,00644585
Protein phosphatase 1, regulatory subunit 21	*Ppp1r21*	1,30984588	0,00308282
Polymerase (dna-directed), delta interacting protein 2	*Poldip2*	1,30954683	0,00350128
Ubiquitin specific peptidase 9, x chromosome	*Usp9x*	1,30901735	0,00660122
Keratin 36	*Krt36*	1,30892424	0,00387783
Pten induced putative kinase 1	*Pink1*	1,30856876	0,00255582
Heme binding protein 1	*Hebp1*	1,30835527	0,00288795
Catenin (cadherin associated protein), alpha 1	*Ctnna1*	1,30801684	0,00142882
Microsomal triglyceride transfer protein	*Mttp*	1,30774926	0,00449769
Serine/threonine kinase 11	*Stk11*	1,30749815	0,00431548
Atp synthase, h+ transporting, mitochondrial f0 complex, subunit b1	*Atp5f1*	1,30726702	0,00179895
Histocompatibility 2, t region locus 10 /// histocompatibility 2, t region locus 22 /// histocompatibility 2, t region locus 9	*H2-t10 /// h2-t22 /// h2-t9*	1,30672539	0,00359506
Histone deacetylase 1	*Hdac1*	1,3065813	0,00638011
Fragile x mental retardation gene 1, autosomal homolog	*Fxr1*	1,30643668	0,00483075
Ras-like without caax 1	*Rit1*	1,30520559	0,00199961
Engulfment and cell motility 2	*Elmo2*	1,30470601	0,0027479
Ceroid lipofuscinosis, neuronal 3, juvenile (batten, spielmeyer-vogt disease)	*Cln3*	1,30450872	0,00585036
Riken cdna 1810037i17 gene /// predicted gene 2036	*1810037i17rik /// gm2036*	1,30420003	0,00672941
Syntaxin binding protein 1	*Stxbp1*	1,30391858	0,00982635
Denn/madd domain containing 5a	*Dennd5a*	1,30385112	0,0010875

O-sialoglycoprotein endopeptidase	*Osgep*	1,30324105	0,00605046
Ctr9, paf1/rna polymerase ii complex component, homolog (s. Cerevisiae)	*Ctr9*	1,30320091	0,00262179
Nima (never in mitosis gene a)-related expressed kinase 2	*Nek2*	1,30302274	0,0068225
Mitochondrial translation optimization 1 homolog (s. Cerevisiae)	*Mto1*	1,30301262	0,00331727
Wnk lysine deficient protein kinase 1	*Wnk1*	1,30257723	0,00123371
Aspartyl aminopeptidase	*Dnpep*	1,30201121	0,00170612
Adenylosuccinate lyase	*Adsl*	1,30196918	0,0025513
Transmembrane protein 184b	*Tmem184b*	1,30190849	0,00154442
Casein kinase 1, alpha 1	*Csnk1a1*	1,30169828	0,00328754
Microphthalmia-associated transcription factor	*Mitf*	1,30152936	0,00536898
Replication protein a3	*Rpa3*	1,30122751	0,00782985
Mitochondrial ribosomal protein l15	*Mrpl15*	1,30073001	0,00155094
Predicted gene 10015 /// predicted gene 10145 /// predicted gene 10705 /// predicted gene 5858 /// ubiquitin-conjugating enzyme e2l 3	*Gm10015 /// gm10145 /// gm10705 /// gm5858 /// ube2l3*	1,30013769	0,00367182
Asparagine-linked glycosylation 5 (dolichyl-phosphate beta-glucosyltransferase)	*Alg5*	1,30009471	0,00149482
Cdna sequence bc017643	*Bc017643*	1,29937266	0,00391273
Zinc finger rna binding protein 2	*Zfr2*	1,29902417	0,00958836
Schlafen 8	*Slfn8*	1,29897085	0,00884021
Leucyl-trna synthetase	*Lars*	1,29879136	0,00242934
Capping protein (actin filament) muscle z-line, alpha 1	*Capza1*	1,29874869	0,00672285
Clathrin, light polypeptide (lca)	*Clta*	1,29869015	0,00262579
Zinc finger ccch type containing 10	*Zc3h10*	1,29859437	0,00476996
Solute carrier family 35, member f6	*Slc35f6*	1,29846429	0,00530349
Ribosomal protein s14	*Rps14*	1,29820252	0,00208514
Rna polymerase ii associated protein 1	*Rpap1*	1,29819001	0,00867657
Adp-ribosylation factor-like 16	*Arl16*	1,29816237	0,00279972
Keratin 16	*Krt16*	1,29799848	0,00290619
Synergin, gamma	*Synrg*	1,29758621	0,00749904
Hepatoma-derived growth factor, related protein 2	*Hdgfrp2*	1,29744373	0,00232634
Mitotic spindle organizing protein 2	*Mzt2*	1,29723414	0,00469752
Trna splicing endonuclease 15 homolog (s. Cerevisiae)	*Tsen15*	1,29703491	0,00552176
Rfad1, flavin adenine dinucleotide synthetase, homolog (yeast)	*Flad1*	1,29688905	0,00461472
Pescadillo homolog 1, containing brct domain (zebrafish)	*Pes1*	1,29570651	0,00434655
Sprouty protein with evh-1 domain 1, related sequence	*Spred1*	1,29564167	0,00276981
Tmem9 domain family, member b	*Tmem9b*	1,29557102	0,00452933
Regulation of nuclear pre-mrna domain containing 1b	*Rprd1b*	1,29526529	0,00730816
Proteasome (prosome, macropain) 26s subunit, atpase 3, interacting protein	*Psmc3ip*	1,29524595	0,0018758
Solute carrier family 35, member e4	*Slc35e4*	1,29487357	0,00899634
Apurinic/apyrimidinic endonuclease 2	*Apex2*	1,29473794	0,00674245

Riken cdna 4931406c07 gene	*4931406c07rik*	1,29461402	0,0036704
Proteasome (prosome, macropain) subunit, alpha type 2	*Psma2*	1,29400792	0,0037987
Er degradation enhancer, mannosidase alpha-like 2	*Edem2*	1,29391975	0,00696527
Mitochondrial ribosomal protein s24	*Mrps24*	1,29388735	0,0046047
Adp-ribosylation factor-like 5a	*Arl5a*	1,29348589	0,00198337
Filamin c, gamma	*Flnc*	1,29341745	0,00901275
Succinate-coa ligase, gdp-forming, alpha subunit	*Suclg1*	1,29278964	0,00206522
Defender against cell death 1	*Dad1*	1,2926727	0,00782985
Pleckstrin homology-like domain, family b, member 1	*Phldb1*	1,29247009	0,00946706
Ornithine decarboxylase antizyme 1	*Oaz1*	1,29238955	0,00362433
Vasoactive intestinal polypeptide	*Vip*	1,29202707	0,00382322
Tubulin, alpha 1b pseudogene /// tubulin, alpha 1b	*Gm5620 /// tuba1b*	1,2917548	0,00138688
Chaperonin containing tcp1, subunit 3 (gamma)	*Cct3*	1,29156964	0,00819458
Bud13 homolog (yeast)	*Bud13*	1,29127096	0,0068107
Kinesin family member 22	*Kif22*	1,29122531	0,00399587
Tax1 (human t cell leukemia virus type i) binding protein 3	*Tax1bp3*	1,29086865	0,0024029
Transmembrane protein 63a	*Tmem63a*	1,29070607	0,00845245
Platelet-activating factor acetylhydrolase 2	*Pafah2*	1,2906827	0,00299673
Autophagy related 3	*Atg3*	1,29064038	0,00533141
Cytochrome p450, family 2, subfamily b, polypeptide 13	*Cyp2b13*	1,29009859	0,00621996
Actin related protein 2/3 complex, subunit 3	*Arpc3*	1,28989335	0,00770516
D-2-hydroxyglutarate dehydrogenase	*D2hgdh*	1,28936055	0,00762973
Predicted gene, 21949 /// schwannomin interacting protein 1	*Gm21949 /// schip1*	1,28910962	0,00467343
Syntaxin 8	*Stx8*	1,28895663	0,00699057
Actin, beta	*Actb*	1,28890036	0,00286966
Endothelin converting enzyme 2	*Ece2*	1,28883338	0,0063824
Peroxisome proliferative activated receptor, gamma, coactivator 1 beta	*Ppargc1b*	1,28848282	0,0082213
Taspase, threonine aspartase 1	*Tasp1*	1,28808802	0,00998206
Williams beuren syndrome chromosome region 22	*Wbscr22*	1,28802507	0,00630526
Angiogenic factor with g patch and fha domains 1	*Aggf1*	1,2872336	0,00283042
Proliferating cell nuclear antigen	*Pcna*	1,28686492	0,00405245
Glucosidase beta 2	*Gba2*	1,28662766	0,00884436
Myc binding protein 2	*Mycbp2*	1,28660275	0,00350128
Serine (or cysteine) peptidase inhibitor, clade b, member 9b	*Serpinb9b*	1,28654588	0,00977186
Nuclear distribution gene c homolog (aspergillus)	*Nudc*	1,28654518	0,00483942
Acyl-coenzyme a dehydrogenase, very long chain	*Acadvl*	1,28617336	0,00650951
Stromal cell-derived factor 2-like 1	*Sdf2l1*	1,28597647	0,00288795
Trans-golgi network vesicle protein 23a	*Tvp23a*	1,28565622	0,00358255
Ornithine decarboxylase, structural 1	*Odc1*	1,28472038	0,00675055
Wd repeat domain 45b	*Wdr45b*	1,28401026	0,00259445
F-box protein 28	*Fbxo28*	1,28382056	0,00680542

Intraflagellar transport 20	*Ift20*	1,28361606	0,00311936
Single-stranded dna binding protein 1	*Ssbp1*	1,28351194	0,00238499
Rab7, member ras oncogene family	*Rab7*	1,2834163	0,0030057
Protein kinase, x-linked	*Prkx*	1,28202628	0,00958648
Thymidylate synthase /// thymidylate synthase, pseudogene	*Tyms /// tyms-ps*	1,28114555	0,00188481
Predicted gene 4736 /// predicted gene 8882 /// proline-rich protein bstni subfamily 1 /// proline-rich protein mp5	*Gm4736 /// gm8882 /// prb1 /// prpmp5*	1,28097284	0,0045084
Centrosomal protein 89	*Cep89*	1,28080302	0,00835379
Sphingosine phosphate lyase 1	*Sgpl1*	1,27959892	0,00993499
Daz associated protein 1	*Dazap1*	1,27944349	0,00989513
Nad(p)h dehydrogenase, quinone 2	*Nqo2*	1,27793797	0,00744285
Helicase with zinc finger domain	*Helz*	1,27740566	0,00564038
Arp8 actin-related protein 8	*Actr8*	1,27708016	0,00535282
Protein (peptidyl-prolyl cis/trans isomerase) nima-interacting 1	*Pin1*	1,27676216	0,00518314
Wap four-disulfide core domain 5	*Wfdc5*	1,27646909	0,00564363
Fucosidase, alpha-l- 2, plasma	*Fuca2*	1,27617222	0,00810042
Beta-site app cleaving enzyme 1	*Bace1*	1,27580426	0,00617449
Trna splicing endonuclease 34 homolog (s. Cerevisiae)	*Tsen34*	1,27566232	0,00217352
Vasohibin 2	*Vash2*	1,27538835	0,00339103
Coenzyme q4 homolog (yeast)	*Coq4*	1,2745957	0,00399587
Microspherule protein 1	*Mcrs1*	1,27448686	0,00207906
Brca1/brca2-containing complex, subunit 3	*Brcc3*	1,27445007	0,00377272
Small nuclear ribonucleoprotein 70 (u1)	*Snrnp70*	1,27403077	0,0034284
Platelet-activating factor acetylhydrolase, isoform 1b, subunit 1	*Pafah1b1*	1,27393417	0,00540161
F-box and wd-40 domain protein 2	*Fbxw2*	1,27370265	0,00244948
Cytoplasmic polyadenylation element binding protein 3	*Cpeb3*	1,27315115	0,00517764
Gelsolin	*Gsn*	1,27187326	0,00299673
Neuroguidin, eif4e binding protein	*Ngdn*	1,27184755	0,00464988
Kinesin family member 1c	*Kif1c*	1,27170433	0,00538098
Er membrane protein complex subunit 2	*Emc2*	1,27134356	0,00328571
Inositol polyphosphate phosphatase-like 1	*Inppl1*	1,27123684	0,00950279
Kinesin family member 1b	*Kif1b*	1,27116951	0,00383471
Dynein, axonemal, light chain 4	*Dnal4*	1,27050151	0,00856643
N-deacetylase/n-sulfotransferase (heparan glucosaminyl) 2	*Ndst2*	1,2693463	0,00469859
Phosphatidylinositol glycan anchor biosynthesis, class s	*Pigs*	1,26876717	0,00883188
Cdk5 regulatory subunit associated protein 3	*Cdk5rap3*	1,26786495	0,00229277
Predicted pseudogene 9769 /// prostaglandin e synthase 3 (cytosolic)	*Gm9769 /// ptges3*	1,26778766	0,00302228
Sap domain containing ribonucleoprotein	*Sarnp*	1,26647548	0,00831015
Rest corepressor 3	*Rcor3*	1,2659788	0,00854535
Carbamoyl-phosphate synthetase 2, aspartate transcarbamylase, and dihydroorotase	*Cad*	1,26477473	0,00640146

Cysteine sulfinic acid decarboxylase	*Csad*	1,26458218	0,00793139
Cdna sequence bc023829	*Bc023829*	1,26456405	0,0038582
Tel2, telomere maintenance 2, homolog (s. Cerevisiae)	*Telo2*	1,26396037	0,00881119
Nudix (nucleoside diphosphate linked moiety x)-type motif 1	*Nudt1*	1,26369474	0,0058462
Basic transcription factor 3	*Btf3*	1,26329236	0,00255833
S100 calcium binding protein a6 (calcyclin)	*S100a6*	1,26321971	0,0026829
Serine/threonine kinase 40	*Stk40*	1,26309723	0,00565768
Atp synthase, h+ transporting, mitochondrial f0 complex, subunit c3 (subunit 9)	*Atp5g3*	1,26236543	0,0027436
Dynein light chain roadblock-type 1	*Dynlrb1*	1,26189517	0,00587783
Predicted gene 8539 /// zinc finger and btb domain containing 8 opposite strand	*Gm8539 /// zbtb8os*	1,26120502	0,00838107
Melanoma inhibitory activity 3	*Mia3*	1,26061918	0,00639777
Suppression of tumorigenicity 13	*St13*	1,26045623	0,00644837
Annexin a2	*Anxa2*	1,26001883	0,00257891
Membrane-bound transcription factor peptidase, site 1	*Mbtps1*	1,25867515	0,00362909
Mitochondrial ribosomal protein s36	*Mrps36*	1,2583465	0,00255816
Acyl-coa synthetase family member 3	*Acsf3*	1,25805845	0,0085295
Canopy 2 homolog (zebrafish)	*Cnpy2*	1,2568544	0,00586918
Tubulin folding cofactor b	*Tbcb*	1,25579825	0,00689876
Centrin 1	*Cetn1*	1,2554023	0,00952001
Solute carrier family 25 (mitochondrial carrier, phosphate carrier), member 25	*Slc25a25*	1,25447048	0,00631358
Protein-tyrosine sulfotransferase 1	*Tpst1*	1,25446673	0,00331596
Zinc finger, dhhc domain containing 4	*Zdhhc4*	1,25357801	0,00568704
Beta-transducin repeat containing protein	*Btrc*	1,25351144	0,00641687
Hydroxysteroid (17-beta) dehydrogenase 2	*Hsd17b2*	1,25344534	0,00703335
Thioredoxin 2	*Txn2*	1,25314758	0,00503083
Hydroxymethylbilane synthase	*Hmbs*	1,25226861	0,00545858
Phosphomannomutase 1	*Pmm1*	1,25208302	0,00794215
Succinate dehydrogenase complex, subunit a, flavoprotein (fp)	*Sdha*	1,25093501	0,00957156
Ecdysoneless homolog (drosophila)	*Ecd*	1,25028745	0,00867205
Predicted gene 10362 /// 60s ribosomal protein l17-like /// 60s ribosomal protein l17-like /// ribosomal protein l17 /// ribosomal protein l17, pseudogene 10 /// ribosomal protein l17, pseudogene 9	*Gm10362 /// loc101056140 /// loc102641612 /// rpl17 /// rpl17-ps10 /// rpl17-ps9*	1,25024224	0,00294667
Proteasome (prosome, macropain) 26s subunit, non-atpase, 13	*Psmd13*	1,25019884	0,0068626
Taf12 rna polymerase ii, tata box binding protein (tbp)-associated factor	*Taf12*	1,24933721	0,00624151
Coatomer protein complex subunit alpha	*Copa*	1,24918236	0,00545462
Solute carrier family 44, member 1	*Slc44a1*	1,24802655	0,00980672
Hydroxyacyl-coenzyme a dehydrogenase	*Hadh*	1,24693397	0,00654375
Dnaj (hsp40) homolog, subfamily a, member 1	*Dnaja1*	1,24644766	0,00430296
Glial cell line derived neurotrophic factor	*Gdnf*	1,24642011	0,00498804

Membrane associated guanylate kinase, ww and pdz domain containing 1	*Magi1*	1,24618535	0,00739598
Mitochondrial amidoxime reducing component 2	*Iii.02*	1,24594104	0,00348955
Dynein cytoplasmic 1 intermediate chain 2	*Dync1i2*	1,24564047	0,0039557
Poly(rc) binding protein 2	*Pcbp2*	1,2453972	0,00437
--- /// heat shock protein 1 (chaperonin 10), pseudogene 2	*Hspe1-ps2 /// hspe1-ps2*	1,2450838	0,00588062
Intraflagellar transport 140	*Ift140*	1,24392195	0,00544694
3-hydroxy-3-methylglutaryl-coenzyme a synthase 1	*Hmgcs1*	1,24330699	0,00851544
Htra serine peptidase 1	*Htra1*	1,24177985	0,00303318
Uncharacterized loc102634352 /// snf2-related crebbp activator protein	*Loc102634352 /// srcap*	1,23932911	0,00926729
Acyl-coa thioesterase 8	*Acot8*	1,23856928	0,00503304
Riken cdna c330007p06 gene	*C330007p06rik*	1,23816719	0,00952343
Apolipoprotein a-i binding protein	*Apoa1bp*	1,23614313	0,00494214
Phosphatidylethanolamine binding protein 1	*Pebp1*	1,23563031	0,0043013
Negative elongation factor complex member b, cobra1	*Nelfb*	1,23518063	0,00850784
Uridine-cytidine kinase 1-like 1	*Uckl1*	1,23516234	0,00826859
Golgi to er traffic protein 4 homolog (s. Cerevisiae)	*Get4*	1,23464342	0,0082723
A kinase (prka) interacting protein 1	*Akip1*	1,23281635	0,00809443
Proteasome (prosome, macropain) activator subunit 2 (pa28 beta) /// protease (prosome, macropain) activator subunit 2b	*Psme2 /// psme2b*	1,23166236	0,00927153
Cdgsh iron sulfur domain 1	*Cisd1*	1,23091086	0,00984893
Par-6 family cell polarity regulator gamma	*Pard6g*	1,2302928	0,00994021
Valosin containing protein	*Vcp*	1,22954042	0,00550564
Amme chromosomal region gene 1-like	*Ammecr1l*	1,22847525	0,00695992
Transmembrane protein 41b	*Tmem41b*	1,22837525	0,00921647
Polypyrimidine tract binding protein 1	*Ptbp1*	1,22761474	0,00828297
Uncharacterized loc102643207 /// mitochondrial ribosomal protein l48	*Loc102643207 /// mrpl48*	1,22738256	0,00742942
Tyrosine 3-monooxygenase/tryptophan 5-monooxygenase activation protein, theta polypeptide	*Ywhaq*	1,2273074	0,00426057
Peroxiredoxin 1 pseudogene /// peroxiredoxin 1	*Gm21399 /// prdx1*	1,2269683	0,00859906
Cysteine-rich secretory protein lccl domain containing 1	*Crispld1*	1,22656083	0,00724376
Replication factor c (activator 1) 5	*Rfc5*	1,22639577	0,0098258
Yy1 associated factor 2	*Yaf2*	1,22613227	0,0043725
Ubiquitin associated domain containing 1	*Ubac1*	1,22394021	0,00942401
Macrophage migration inhibitory factor	*Mif*	1,22391009	0,00645859
Cell division cycle 45	*Cdc45*	1,22375326	0,0059314
Transmembrane and immunoglobulin domain containing 1	*Tmigd1*	1,2229615	0,00968053
Coatomer protein complex, subunit beta 2 (beta prime)	*Copb2*	1,22194474	0,00523193
Peptidase d	*Pepd*	1,22143252	0,00866841
Anaphase promoting complex subunit 4	*Anapc4*	1,22112118	0,00987219
Afg3-like aaa atpase 2	*Afg3l2*	1,22083665	0,00662571
Brca1 interacting protein c-terminal helicase 1, opposite strand	*Brip1os*	1,21907406	0,00631492

Secreted phosphoprotein 2	*Spp2*	1,21840341	0,00981578
Isocitrate dehydrogenase 3 (nad+), gamma	*Idh3g*	1,21295416	0,00814906
Actin related protein 2/3 complex, subunit 1a	*Arpc1a*	1,21229144	0,00989766
Mitochondrial ribosomal protein l10	*Mrpl10*	1,21051309	0,00782971
Centrobin, centrosomal brca2 interacting protein	*Cntrob*	1,20229624	0,00975398
**Gene.Title**	**Gene.Symbol**	**2^logFC**	**adj.p.val**
Matrix metallopeptidase 13	*Mmp13*	-213,53317	3,44E-12
Inactive X specific transcripts	*Xist*	-142,42203	6,45E-11
Inhibin beta-B	*Inhbb*	-125,63207	6,55E-10
Lysyl oxidase	*Lox*	-72,205729	3,23E-10
Gremlin 1	*Grem1*	-68,754314	2,71E-10
Fibronectin 1	*Fn1*	-55,407195	2,71E-10
Interleukin 13 receptor, alpha 1	*Il13ra1*	-53,469942	1,71E-09
Glucosamine-6-phosphate deaminase 2	*Gnpda2*	-51,206631	6,45E-11
Serine (or cysteine) peptidase inhibitor, clade E, member 2	*Serpine2*	-50,629947	6,45E-11
Proline rich 13	*Prr13*	-47,123873	2,33E-10
Trypsin 4 /// trypsin 5	*Try4 /// Try5*	-44,750827	4,21E-10
Lectin, galactoside binding-like	*Lgalsl*	-44,138611	1,14E-10
Regulator of calcineurin 2	*Rcan2*	-37,599922	2,03E-10
SRY (sex determining region Y)-box 11	*Sox11*	-36,640318	2,06E-10
Anthrax toxin receptor 1	*Antxr1*	-34,773312	2,71E-10
UDP-glcnac:betagal beta-1,3-N-acetylglucosaminyltransferase 9	*B3gnt9*	-31,127934	6,55E-10
Acyl-coa thioesterase 1	*Acot1*	-29,65227	6,30E-10
Serum amyloid A 3	*Saa3*	-29,557842	4,21E-10
A disintegrin-like and metallopeptidase (reprolysin type) with thrombospondin type 1 motif, 5 (aggrecanase-2)	*Adamts5*	-29,341216	2,20E-10
Clusterin	*Clu*	-28,19451	1,14E-10
Interleukin 13 receptor, alpha 2	*Il13ra2*	-27,080658	5,59E-09
Ceruloplasmin	*Cp*	-26,483137	3,42E-09
Nuclear factor I/B	*Nfib*	-26,442483	8,30E-10
Heparan sulfate 6-O-sulfotransferase 2	*Hs6st2*	-24,72591	8,37E-10
Transmembrane protein 176A	*Tmem176a*	-23,987612	5,89E-09
Transmembrane protein 176B	*Tmem176b*	-23,983272	4,85E-10
Ribonuclease, rnase A family 4	*Rnase4*	-23,807461	4,21E-09
Solute carrier family 25 (mitochondrial carrier ornithine transporter), member 15	*Slc25a15*	-23,295105	6,37E-10
Lysophosphatidic acid receptor 4	*Lpar4*	-22,849717	1,00E-07
Fibroblast growth factor 7	*Fgf7*	-22,310157	1,58E-09
ATP-binding cassette, sub-family G (WHITE), member 2	*Abcg2*	-22,059047	3,57E-09
Glucosaminyl (N-acetyl) transferase 2, I-branching enzyme	*Gcnt2*	-21,954323	2,23E-09
Solute carrier family 24 (sodium/potassium/calcium exchanger), member 3	*Slc24a3*	-21,926616	1,03E-08

Immunoglobulin superfamily, DCC subclass, member 4	*Igdcc4*	-21,43308	5,96E-10
Myogenic factor 5	*Myf5*	-21,099131	4,21E-10
Aldo-keto reductase family 1, member C18	*Akr1c18*	-20,912855	1,04E-09
Guanine deaminase	*Gda*	-20,632907	2,58E-09
Transmembrane protein 98	*Tmem98*	-20,555627	1,93E-07
Disabled 2, mitogen-responsive phosphoprotein	*Dab2*	-20,521364	8,89E-10
Cholinergic receptor, nicotinic, beta polypeptide 1 (muscle)	*Chrnb1*	-20,39487	1,25E-09
Sphingomyelin phosphodiesterase, acid-like 3B	*Smpdl3b*	-20,007048	2,02E-09
Brain derived neurotrophic factor	*Bdnf*	-19,836436	3,84E-09
Epidermal growth factor-containing fibulin-like extracellular matrix protein 1	*Efemp1*	-19,404751	1,88E-09
Protocadherin 7	*Pcdh7*	-19,087566	1,44E-09
Frizzled homolog 6 (Drosophila)	*Fzd6*	-18,606621	3,07E-09
Cadherin 15	*Cdh15*	-17,877482	1,58E-09
Protein kinase D1	*Prkd1*	-17,690578	2,20E-10
Metallothionein 2	*Mt2*	-16,984148	3,32E-10
Tetratricopeptide repeat domain 30A1	*Ttc30a1*	-16,949514	3,30E-07
PRKC, apoptosis, WT1, regulator	*Pawr*	-16,667418	3,03E-08
Insulin-like growth factor binding protein 5	*Igfbp5*	-16,645444	6,39E-10
Stearoyl-Coenzyme A desaturase 1	*Scd1*	-16,393254	3,75E-10
Fibronectin type III domain containing 4	*Fndc4*	-15,990521	1,55E-08
Leucine rich repeat protein 1, neuronal	*Lrrn1*	-15,89245	1,55E-08
Haptoglobin	*Hp*	-15,545423	4,21E-10
Collagen, type IV, alpha 1	*Col4a1*	-15,276089	2,36E-09
S100 calcium binding protein A8 (calgranulin A)	*S100a8*	-15,160143	3,73E-09
Collagen, type IV, alpha 2	*Col4a2*	-14,640044	5,99E-09
Receptor accessory protein 1	*Reep1*	-14,616622	5,64E-09
Nephronectin	*Npnt*	-13,783281	4,57E-08
Myc induced nuclear antigen	*Mina*	-13,669299	2,64E-09
Neuronal regeneration related protein	*Nrep*	-13,419839	2,49E-08
Carbonic anhydrase 3	*Car3*	-13,380518	2,54E-08
Coagulation factor III	*F3*	-13,262845	6,53E-08
Oncostatin M receptor	*Osmr*	-12,94217	3,07E-09
Carbonyl reductase 1	*Cbr1*	-12,91644	2,36E-09
Four and a half LIM domains 1	*Fhl1*	-12,520381	5,14E-09
B cell translocation gene 3 /// B-cell translocation gene 3 pseudogene	*Btg3 /// Gm7334*	-12,431184	7,58E-09
Cyclin-dependent kinase 14	*Cdk14*	-12,405975	1,01E-08
Forkhead box C2	*Foxc2*	-12,400636	9,51E-09
Signal-regulatory protein alpha	*Sirpa*	-12,322868	8,02E-10
Lipocalin 2	*Lcn2*	-12,286672	1,48E-08
Bradykinin receptor, beta 1	*Bdkrb1*	-12,282902	4,21E-10
Protocadherin 18	*Pcdh18*	-12,178454	5,93E-09
Matrix metallopeptidase 3	*Mmp3*	-12,037466	1,51E-08

Collapsin response mediator protein 1	*Crmp1*	-12,027358	9,88E-10
Acyl-coa thioesterase 1 /// acyl-coa thioesterase 2	*Acot1 /// Acot2*	-11,733357	6,00E-09
PDZ and LIM domain 3	*Pdlim3*	-11,668623	4,21E-10
Nidogen 1	*Nid1*	-11,455679	1,26E-07
ELAV (embryonic lethal, abnormal vision, Drosophila)-like 2 (Hu antigen B)	*Elavl2*	-11,416921	1,63E-08
Interleukin 1 receptor antagonist	*Il1rn*	-11,196726	2,90E-09
Desmocollin 2	*Dsc2*	-10,80115	6,55E-09
Titin	*Ttn*	-10,662864	1,44E-09
Pleckstrin	*Plek*	-10,536	9,45E-09
CXADR-like membrane protein	*Clmp*	-10,308937	7,70E-07
Connective tissue growth factor	*Ctgf*	-10,126952	6,52E-09
CD53 antigen	*Cd53*	-9,8147674	7,61E-08
RIKEN cdna 6720475J19 gene	*6720475J19Rik*	-9,7584683	1,24E-08
RIKEN cdna D430019H16 gene	*D430019H16Rik*	-9,0712486	2,12E-09
Histocompatibility 2, D region locus 1 /// histocompatibility 2, D region locus L	*H2-D1 /// H2-L*	-9,0134839	6,77E-09
Actin, alpha 2, smooth muscle, aorta	*Acta2*	-8,9503748	1,16E-08
Midkine	*Mdk*	-8,7477468	6,53E-08
Scavenger receptor class A, member 3	*Scara3*	-8,6740238	6,52E-09
Serine (or cysteine) peptidase inhibitor, clade B, member 2	*Serpinb2*	-8,520025	1,45E-07
Armadillo repeat containing, X-linked 4	*Armcx4*	-8,2608939	3,57E-09
CD9 antigen	*Cd9*	-8,187259	6,55E-10
Histocompatibility 2, D region locus 1	*H2-D1*	-8,1750962	5,46E-09
Thrombospondin 3	*Thbs3*	-8,1709349	2,04E-08
CCAAT/enhancer binding protein (C/EBP), delta	*Cebpd*	-8,0217563	9,22E-08
N-myc downstream regulated gene 1	*Ndrg1*	-8,0180733	2,37E-08
Iroquois related homeobox 3	*Irx3*	-7,8722666	2,79E-09
Matrix metallopeptidase 2	*Mmp2*	-7,8311422	2,18E-09
Starch binding domain 1	*Stbd1*	-7,8279143	3,07E-09
Chemokine (C-X-C motif) ligand 5	*Cxcl5*	-7,7666984	2,86E-08
Hyaluronan synthase 2	*Has2*	-7,7199024	5,45E-08
Ectonucleotide pyrophosphatase/phosphodiesterase 5	*Enpp5*	-7,5355376	1,44E-06
Netrin 1	*Ntn1*	-7,5276327	2,86E-08
Prostaglandin-endoperoxide synthase 2	*Ptgs2*	-7,5100753	1,66E-06
Histocompatibility 2, K1, K region	*H2-K1*	-7,4754257	8,81E-10
Paired box 7	*Pax7*	-7,3605056	1,62E-06
Atpase, Na+/K+ transporting, alpha 3 polypeptide	*Atp1a3*	-7,3082359	1,46E-05
Choline kinase alpha	*Chka*	-7,3036551	2,73E-08
Matrix metallopeptidase 23	*Mmp23*	-7,2987182	1,18E-07
Collagen, type III, alpha 1	*Col3a1*	-7,2899813	2,33E-09
SMT3 suppressor of mif two 3 homolog 3 (yeast)	*Sumo3*	-7,2843802	3,24E-09
Tumor protein D52-like 1	*Tpd52l1*	-7,2525191	4,91E-09
Family with sequence similarity 132, member A	*Fam132a*	-7,2202857	1,93E-07
Sphingosine kinase 1	*Sphk1*	-7,1806105	3,84E-08

Cyclin-dependent kinase inhibitor 2A	*Cdkn2a*	-7,1556195	1,37E-07
Ankyrin repeat domain 1 (cardiac muscle)	*Ankrd1*	-7,0441502	1,58E-09
Small nucleolar RNA host gene 1	*Snhg1*	-7,043527	2,33E-09
A disintegrin-like and metallopeptidase (reprolysin type) with thrombospondin type 1 motif, 1	*Adamts1*	-6,922291	7,60E-08
Cathepsin F	*Ctsf*	-6,8066507	1,72E-09
Tumor necrosis factor, alpha-induced protein 2	*Tnfaip2*	-6,662343	2,70E-07
Very low density lipoprotein receptor	*Vldlr*	-6,6544573	2,23E-06
Oligodendrocyte transcription factor 1	*Olig1*	-6,6102193	8,86E-09
Podocalyxin-like	*Podxl*	-6,6043692	1,45E-07
Collagen, type V, alpha 2	*Col5a2*	-6,5806915	3,99E-08
KDEL (Lys-Asp-Glu-Leu) endoplasmic reticulum protein retention receptor 3	*Kdelr3*	-6,5166688	7,75E-09
Interleukin 1 receptor accessory protein	*Il1rap*	-6,5070999	1,11E-06
Solute carrier family 44, member 2	*Slc44a2*	-6,4986194	5,95E-07
Nucleolar protein 3 (apoptosis repressor with CARD domain)	*Nol3*	-6,4182831	6,09E-07
Protein tyrosine phosphatase, receptor type, D	*Ptprd*	-6,3535944	1,52E-07
Transmembrane protein 121	*Tmem121*	-6,3221863	1,48E-08
Nuclear paraspeckle assembly transcript 1 (non-protein coding)	*Neat1*	-6,2828259	8,51E-07
Frizzled homolog 4 (Drosophila)	*Fzd4*	-6,2611551	9,60E-08
Procollagen C-endopeptidase enhancer 2	*Pcolce2*	-6,1889154	1,94E-09
Paired related homeobox 2	*Prrx2*	-6,1546609	5,51E-08
WAP four-disulfide core domain 2	*Wfdc2*	-6,1427031	2,62E-08
Abhydrolase domain containing 6	*Abhd6*	-6,1292932	2,08E-07
CDC42 effector protein (Rho gtpase binding) 3	*Cdc42ep3*	-6,0831126	1,01E-08
CDC42 effector protein (Rho gtpase binding) 5	*Cdc42ep5*	-6,049787	6,63E-08
Adenosine A2b receptor	*Adora2b*	-6,0169607	2,31E-08
Retinol binding protein 1, cellular	*Rbp1*	-5,9565427	1,48E-07
Coagulation factor XIII, A1 subunit	*F13a1*	-5,9425848	1,39E-06
Solute carrier family 40 (iron-regulated transporter), member 1	*Slc40a1*	-5,8807007	1,49E-07
Neural precursor cell expressed, developmentally down-regulated gene 9	*Nedd9*	-5,8592177	1,36E-07
Glycoprotein 49 A /// leukocyte immunoglobulin-like receptor, subfamily B, member 4	*Gp49a /// Lilrb4*	-5,780959	7,06E-07
Malignant T cell amplified sequence 2	*Mcts2*	-5,739085	1,99E-07
Poly (ADP-ribose) polymerase family, member 12	*Parp12*	-5,643457	2,04E-06
Membrane bound O-acyltransferase domain containing 2	*Mboat2*	-5,6153325	1,93E-07
Serum deprivation response	*Sdpr*	-5,5602266	2,29E-07
Sortilin-related VPS10 domain containing receptor 2	*Sorcs2*	-5,5115323	2,25E-08
Secretory leukocyte peptidase inhibitor	*Slpi*	-5,4858615	5,06E-08
SH3 domain binding glutamic acid-rich protein like 2	*Sh3bgrl2*	-5,4120912	3,46E-08
Family with sequence similarity 213, member A	*Fam213a*	-5,3798927	3,84E-06
Junction adhesion molecule 3	*Jam3*	-5,357589	6,22E-07
Zinc finger, DHHC domain containing 2	*Zdhhc2*	-5,34096	5,47E-06

Berardinelli-Seip congenital lipodystrophy 2 homolog (seipin)	*Bscl2*	-5,3351753	3,90E-08
Beta-1,4-N-acetyl-galactosaminyl transferase 1	*B4galnt1*	-5,2602608	6,05E-08
MARCKS-like 1	*Marcksl1*	-5,2300082	3,74E-08
Coxsackie virus and adenovirus receptor	*Cxadr*	-5,2271679	9,55E-07
Homeobox B9	*Hoxb9*	-5,1719859	4,66E-07
Acyl-coa thioesterase 4	*Acot4*	-5,0572196	5,95E-06
Integrin alpha 6	*Itga6*	-4,9577248	1,34E-07
MACRO domain containing 1	*Macrod1*	-4,9552556	6,24E-08
PTK7 protein tyrosine kinase 7	*Ptk7*	-4,9502057	3,73E-09
Myosin ID	*Myo1d*	-4,9104612	1,24E-05
Nuclear factor, interleukin 3, regulated	*Nfil3*	-4,885142	1,47E-08
Cdna sequence BC002189	*BC002189*	-4,8428062	4,63E-05
Tripartite motif-containing 47	*Trim47*	-4,8406718	4,08E-08
Family with sequence similarity 46, member C	*Fam46c*	-4,8116964	4,23E-07
Nidogen 2	*Nid2*	-4,8106016	5,39E-08
Plasminogen activator, tissue	*Plat*	-4,7763604	5,53E-07
Prostaglandin F receptor	*Ptgfr*	-4,7574681	6,20E-07
Zinc finger protein, multitype 2	*Zfpm2*	-4,7536062	1,49E-08
Scavenger receptor class A, member 5 (putative)	*Scara5*	-4,7377895	1,36E-07
Matrix metallopeptidase 10	*Mmp10*	-4,7316064	1,46E-07
Reticulon 2 (Z-band associated protein)	*Rtn2*	-4,7140536	1,37E-06
Kruppel-like factor 2 (lung)	*Klf2*	-4,7006379	7,11E-08
Cytochrome P450, family 4, subfamily v, polypeptide 3	*Cyp4v3*	-4,6938015	2,90E-07
ABRA C-terminal like /// predicted pseudogene 6314	*Abracl /// Gm6314*	-4,6533144	1,44E-08
Adenylate kinase 4	*Ak4*	-4,6191065	4,12E-08
Suppressor of cytokine signaling 3	*Socs3*	-4,5895309	2,51E-07
Solute carrier family 16 (monocarboxylic acid transporters), member 1	*Slc16a1*	-4,5698151	3,23E-07
Solute carrier family 16 (monocarboxylic acid transporters), member 2	*Slc16a2*	-4,5572521	1,34E-06
Sialic acid acetylesterase	*Siae*	-4,5205357	8,31E-07
Transforming growth factor, beta 2	*Tgfb2*	-4,4917354	8,60E-07
Gap junction protein, gamma 1	*Gjc1*	-4,4731206	2,71E-08
3-hydroxybutyrate dehydrogenase, type 1	*Bdh1*	-4,3618719	4,89E-08
GM2 ganglioside activator protein	*Gm2a*	-4,3377518	4,52E-07
S100 calcium binding protein A16	*S100a16*	-4,3261396	5,93E-09
Histone cluster 1, H4a /// histone cluster 1, H4b /// histone cluster 1, H4c /// histone cluster 1, H4d /// histone cluster 1, H4f /// histone cluster 1, H4h /// histone cluster 1, H4i /// histone cluster 1, H4j /// histone cluster 1, H4k /// histone cluster 1, H4m /// histone cluster 1, H4n /// histone cluster 2, H4 /// histone cluster 4, H4	*Hist1h4a /// Hist1h4b /// Hist1h4c /// Hist1h4d /// Hist1h4f /// Hist1h4h /// Hist1h4i /// Hist1h4j /// Hist1h4k /// Hist1h4m ///*	-4,32085	7,06E-06

	Hist1h4n /// Hist2h4 /// Hist4h4		
Distal-less homeobox 4	*Dlx4*	-4,3149691	4,53E-07
Decorin	*Dcn*	-4,314674	1,92E-06
Leptin receptor	*Lepr*	-4,2643964	2,75E-06
SRY (sex determining region Y)-box 12	*Sox12*	-4,248915	2,56E-07
Ena-vasodilator stimulated phosphoprotein	*Evl*	-4,2396419	9,89E-07
Estrogen related receptor, beta	*Esrrb*	-4,2380164	3,52E-06
Collectin sub-family member 12	*Colec12*	-4,2328207	1,71E-06
Junction adhesion molecule 2	*Jam2*	-4,2061606	1,51E-06
RIKEN cdna 5730408K05 gene /// microrna 5136	*5730408K05Rik /// Mir5136*	-4,1909173	5,21E-06
Periostin, osteoblast specific factor	*Postn*	-4,1774896	2,49E-08
Solute carrier family 25 (mitochondrial carnitine/acylcarnitine translocase), member 20	*Slc25a20*	-4,1440571	1,36E-07
Caspase 4, apoptosis-related cysteine peptidase	*Casp4*	-4,1403709	4,09E-08
Zinc finger protein of the cerebellum 2	*Zic2*	-4,120063	1,90E-07
Polycomb group ring finger 6	*Pcgf6*	-4,1161639	4,57E-08
Metallothionein 1	*Mt1*	-4,1156477	1,93E-07
RAR-related orphan receptor gamma	*Rorc*	-4,0972904	2,06E-06
Fibulin 1	*Fbln1*	-4,0834853	4,00E-07
Cytidine monophospho-N-acetylneuraminic acid hydroxylase	*Cmah*	-4,0774904	2,32E-06
Ankyrin repeat domain 10	*Ankrd10*	-4,0697923	3,67E-07
CKLF-like MARVEL transmembrane domain containing 7	*Cmtm7*	-4,0642102	1,25E-07
F-box protein 32	*Fbxo32*	-4,0613342	4,44E-06
Ribosomal protein S20	*Rps20*	-4,0600876	9,96E-07
Melanocyte proliferating gene 1	*Myg1*	-3,9952414	6,63E-08
Interferon gamma inducible protein 30	*Ifi30*	-3,9779674	2,04E-08
Acyl-coa thioesterase 2	*Acot2*	-3,9448483	4,57E-08
Ferredoxin 1-like /// Raver1-Fdx1l readthrough	*Fdx1l /// Raver1-fdx1l*	-3,904555	9,97E-08
Slit homolog 2 (Drosophila)	*Slit2*	-3,9042483	7,22E-06
Sushi-repeat-containing protein, X-linked 2	*Srpx2*	-3,8953438	2,83E-06
Growth factor receptor bound protein 14	*Grb14*	-3,8763708	1,41E-05
Transmembrane protein 38B	*Tmem38b*	-3,8720062	4,93E-06
Guanine nucleotide binding protein, alpha 14	*Gna14*	-3,8589139	7,63E-07
Alpha 1,4-galactosyltransferase	*A4galt*	-3,8514778	2,29E-06
Thrombospondin 1	*Thbs1*	-3,851401	2,89E-06
Paternally expressed 3	*Peg3*	-3,8383861	1,72E-08
Fibronectin type III domain containing 1	*Fndc1*	-3,8043144	3,87E-06
Solute carrier family 4 (anion exchanger), member 4	*Slc4a4*	-3,788449	4,85E-06
Collagen, type V, alpha 3	*Col5a3*	-3,776821	1,10E-06
Protease, serine 23	*Prss23*	-3,7643586	3,60E-06
Membrane protein, palmitoylated 7 (MAGUK p55 subfamily member 7)	*Mpp7*	-3,7532198	2,79E-06

N-acetylated alpha-linked acidic dipeptidase 2	*Naalad2*	-3,7478646	4,39E-06
Follistatin-like 1	*Fstl1*	-3,7321433	1,47E-08
Renin binding protein	*Renbp*	-3,7258488	3,83E-08
Ets variant 1 /// predicted gene 5454	*Etv1 /// Gm5454*	-3,7173855	2,33E-07
Serglycin	*Srgn*	-3,7171635	4,37E-06
Phosphoribosyl pyrophosphate synthetase 1-like 3	*Prps1l3*	-3,6855149	3,27E-07
Basonuclin 1	*Bnc1*	-3,679649	3,39E-07
Tribbles homolog 2 (Drosophila)	*Trib2*	-3,6675639	2,22E-07
Zinc finger protein 521	*Zfp521*	-3,6366736	1,81E-06
Ectonucleotide pyrophosphatase/phosphodiesterase 2	*Enpp2*	-3,6309122	2,63E-06
Tripartite motif-containing 30A	*Trim30a*	-3,6234602	3,51E-06
Reticulocalbin 1	*Rcn1*	-3,6223057	7,78E-08
Ring finger protein 11	*Rnf11*	-3,6070849	1,38E-05
NME/NM23 nucleoside diphosphate kinase 4	*Nme4*	-3,5858294	1,18E-05
Eukaryotic translation initiation factor 4E member 3	*Eif4e3*	-3,5762657	5,29E-07
Selenium binding protein 1	*Selenbp1*	-3,5688637	5,51E-06
Syntaxin 3	*Stx3*	-3,5493671	6,20E-07
Chloride channel 3	*Clcn3*	-3,5473415	1,79E-07
Brain expressed myelocytomatosis oncogene	*Bmyc*	-3,545725	1,59E-06
Insulin receptor substrate 1	*Irs1*	-3,5453103	3,67E-07
Integral membrane protein 2A	*Itm2a*	-3,5265346	0,00016653
Chromodomain protein, Y chromosome-like	*Cdyl*	-3,5206551	1,99E-06
RIKEN cdna 1810055G02 gene	*1810055G02Rik*	-3,5154313	1,07E-08
Component of oligomeric golgi complex 8	*Cog8*	-3,4876252	6,08E-08
Muscle, skeletal, receptor tyrosine kinase	*Musk*	-3,4850363	1,68E-07
Four jointed box 1 (Drosophila)	*Fjx1*	-3,4819938	2,01E-05
Deiodinase, iodothyronine, type II	*Dio2*	-3,4812541	6,00E-07
Schlafen 2	*Slfn2*	-3,479936	3,39E-06
Regulator of G-protein signaling 19	*Rgs19*	-3,4462342	4,71E-07
Actin-binding LIM protein 1	*Ablim1*	-3,4420939	9,86E-07
Calcium channel, voltage-dependent, L type, alpha 1C subunit	*Cacna1c*	-3,4355213	3,55E-07
Myomesin 2	*Myom2*	-3,4334165	9,68E-07
RIKEN cdna 1190002N15 gene	*1190002N15Rik*	-3,4253645	3,92E-06
Dapper homolog 1, antagonist of beta-catenin (xenopus)	*Dact1*	-3,423001	1,45E-06
Cyclin-dependent kinase inhibitor 2B (p15, inhibits CDK4)	*Cdkn2b*	-3,4214807	2,28E-05
Component of oligomeric golgi complex 8 /// peptide deformylase (mitochondrial)	*Cog8 /// Pdf*	-3,4129843	6,36E-08
PDZ and LIM domain 4	*Pdlim4*	-3,4115804	1,23E-07
Transmembrane protein 173	*Tmem173*	-3,4094963	1,47E-06
Prostaglandin reductase 1	*Ptgr1*	-3,4031754	3,00E-07
Ciliary neurotrophic factor /// zinc finger protein 91 /// Zfp91-Cntf readthrough transcript (NMD candidate)	*Cntf /// Zfp91 /// Zfp91Cntf*	-3,3889911	1,46E-07
Cysteine and glycine-rich protein 2	*Csrp2*	-3,3820934	7,46E-08
Histocompatibility 2, class II antigen A, alpha	*H2-Aa*	-3,3709734	1,14E-05

Inhibitor of growth family, member 1	*Ing1*	-3,363131	7,69E-08
Protocadherin 1	*Pcdh1*	-3,350499	1,63E-05
Growth arrest specific 5	*Gas5*	-3,3467138	9,49E-08
Ring finger protein 149	*Rnf149*	-3,3379417	7,21E-07
Coronin, actin binding protein, 2B	*Coro2b*	-3,330451	9,13E-05
Syndecan 4	*Sdc4*	-3,3236031	1,05E-06
B cell leukemia/lymphoma 3	*Bcl3*	-3,3179232	7,87E-06
Dnaj (Hsp40) homolog, subfamily B, member 9	*Dnajb9*	-3,3099388	1,50E-05
Cytochrome b-245, alpha polypeptide	*Cyba*	-3,3098179	1,97E-07
Small EDRK-rich factor 1	*Serf1*	-3,3097197	2,59E-07
Activating transcription factor 3	*Atf3*	-3,3052807	3,05E-07
Zinc finger protein 62	*Zfp62*	-3,2961066	5,18E-06
RIKEN cdna D030056L22 gene	*D030056L22Rik*	-3,2823294	4,13E-08
Histocompatibility 13	*H13*	-3,281641	1,99E-07
Family with sequence similarity 129, member A	*Fam129a*	-3,2804877	9,77E-06
Atpase, H+ transporting, lysosomal V0 subunit E2	*Atp6v0e2*	-3,2728209	7,60E-06
Solute carrier family 6 (neurotransmitter transporter, glycine), member 9	*Slc6a9*	-3,2723988	2,29E-07
Ubiquitin specific peptidase 17-like A	*Usp17la*	-3,2695589	1,55E-05
RIKEN cdna E330009J07 gene	*E330009J07Rik*	-3,2595629	1,08E-05
Transmembrane protein 132A	*Tmem132a*	-3,2489087	6,34E-07
RAD52 homolog (S. Cerevisiae)	*Rad52*	-3,248531	7,53E-06
Family with sequence similarity 122, member A	*Fam122a*	-3,2361126	8,54E-06
Eomesodermin homolog (Xenopus laevis)	*Eomes*	-3,2187681	1,17E-05
Rho guanine nucleotide exchange factor (GEF) 5	*Arhgef5*	-3,2187415	5,12E-07
Leukemia inhibitory factor receptor	*Lifr*	-3,2181088	6,71E-06
Zinc finger protein of the cerebellum 5	*Zic5*	-3,2113066	1,63E-06
Tissue inhibitor of metalloproteinase 3	*Timp3*	-3,2028526	6,39E-07
Par-3 family cell polarity regulator	*Pard3*	-3,1967077	1,83E-05
CD320 antigen	*Cd320*	-3,1958343	8,50E-08
Poliovirus receptor-related 2	*Pvrl2*	-3,1919216	7,43E-07
Translation machinery associated 16 homolog (S. Cerevisiae)	*Tma16*	-3,1913675	3,74E-07
Tubulin, beta 2B class IIB	*Tubb2b*	-3,1864181	1,31E-06
Synaptotagmin binding, cytoplasmic RNA interacting protein	*Syncrip*	-3,1752746	1,81E-06
Follistatin	*Fst*	-3,1740203	2,17E-07
Nuclear factor of kappa light polypeptide gene enhancer in B cells inhibitor, zeta	*Nfkbiz*	-3,1717511	7,97E-08
Prolactin family 7, subfamily c, member 1	*Prl7c1*	-3,1681457	7,47E-07
Phosphodiesterase 4B, camp specific	*Pde4b*	-3,149691	2,99E-06
Zinc finger protein 810	*Zfp810*	-3,1375508	5,97E-06
Mitochondrial ribosomal protein L43	*Mrpl43*	-3,1318002	2,19E-07
Zinc finger, DHHC domain containing 16	*Zdhhc16*	-3,1273375	7,47E-08
Aldehyde dehydrogenase 18 family, member A1	*Aldh18a1*	-3,1264395	2,08E-06
Baculoviral IAP repeat-containing 3	*Birc3*	-3,1194038	6,94E-06

Cystathionase (cystathionine gamma-lyase)	*Cth*	-3,0987831	0,00010665
Histocompatibility 2, D region locus 1 /// histocompatibility 2, K1, K region /// h-2 class I histocompatibility antigen, K-D alpha chain-like	*H2-D1 /// H2-K1 /// LOC101056305*	-3,0967233	9,59E-05
Lipoprotein lipase	*Lpl*	-3,0960704	8,19E-06
Zinc finger protein 91	*Zfp91*	-3,0936812	9,04E-05
Arrestin domain containing 4	*Arrdc4*	-3,0789014	3,70E-06
TM2 domain containing 2	*Tm2d2*	-3,0788298	3,29E-06
Myelin protein zero-like 2	*Mpzl2*	-3,0614159	2,29E-06
Complement component 3	*C3*	-3,0581766	7,43E-07
Tetratricopeptide repeat domain 13	*Ttc13*	-3,0572622	3,93E-08
Spla/ryanodine receptor domain and SOCS box containing 1	*Spsb1*	-3,0568164	9,59E-07
Integrator complex subunit 5	*Ints5*	-3,0559599	1,89E-07
Atpase, Na+/K+ transporting, beta 1 polypeptide	*Atp1b1*	-3,0416059	1,39E-05
Pseudouridine synthase 3	*Pus3*	-3,0408838	7,18E-06
CD59a antigen	*Cd59a*	-3,0348221	2,47E-06
SUMO-interacting motifs containing 1	*Simc1*	-3,0335084	2,95E-07
Ribosomal protein L22	*Rpl22*	-3,0269046	1,86E-07
Neuron specific gene family member 1	*Nsg1*	-3,025951	1,47E-06
Tribbles homolog 3 (Drosophila)	*Trib3*	-3,0101013	5,91E-07
Growth arrest specific 6	*Gas6*	-3,0030225	3,08E-05
Phosphoenolpyruvate carboxykinase 2 (mitochondrial)	*Pck2*	-2,9980923	1,88E-07
DNA segment, Chr 19, Brigham & Women's Genetics 1357 expressed	*D19Bwg1357e*	-2,9970499	8,54E-07
Tropomyosin 1, alpha	*Tpm1*	-2,9917688	1,60E-05
Zinc finger protein 703	*Zfp703*	-2,9896759	2,47E-06
CD1d1 antigen	*Cd1d1*	-2,9875229	5,26E-06
CCAAT/enhancer binding protein (C/EBP), gamma	*Cebpg*	-2,9818454	2,92E-07
Metastasis associated lung adenocarcinoma transcript 1 (non-coding RNA)	*Malat1*	-2,977205	0,00166522
NADH dehydrogenase (ubiquinone) 1 alpha subcomplex, assembly factor 4	*Ndufaf4*	-2,9770896	5,63E-07
Pentraxin related gene	*Ptx3*	-2,9761673	9,57E-06
Endothelin converting enzyme 1	*Ece1*	-2,9717876	1,63E-06
Family with sequence similarity 195, member A	*Fam195a*	-2,9549816	1,10E-06
Transmembrane protein 2	*Tmem2*	-2,9480345	3,44E-06
SMAD family member 1	*Smad1*	-2,9395256	5,12E-07
Suppressor of cytokine signaling 2	*Socs2*	-2,9363014	1,17E-05
Solute carrier family 25, member 28	*Slc25a28*	-2,9322853	6,01E-07
Serine (or cysteine) peptidase inhibitor, clade B, member 9c	*Serpinb9c*	-2,9216005	6,39E-07
STAM binding protein like 1	*Stambpl1*	-2,9205342	2,54E-07
Quaking	*Qk*	-2,9199444	0,000182
Oxidative stress induced growth inhibitor family member 2	*Osgin2*	-2,9098	1,67E-06
Phosphoribosylglycinamide formyltransferase	*Gart*	-2,9084275	1,26E-07
Exosome component 1	*Exosc1*	-2,8792985	3,16E-07

Son of sevenless homolog 1 (Drosophila)	*Sos1*	-2,8729444	0,00254443
Zinc finger and BTB domain containing 46	*Zbtb46*	-2,8670737	2,06E-07
Kruppel-like factor 9	*Klf9*	-2,8661574	4,63E-07
Transcription factor 7 like 2, T cell specific, HMG box	*Tcf7l2*	-2,8649841	4,16E-07
Platelet derived growth factor receptor, alpha polypeptide	*Pdgfra*	-2,8606526	8,38E-06
Ribosomal protein S6	*Rps6*	-2,8576161	3,86E-07
Serine palmitoyltransferase, long chain base subunit 2	*Sptlc2*	-2,8575359	2,23E-07
Intestinal cell kinase	*Ick*	-2,8560279	8,97E-07
Pantothenate kinase 1	*Pank1*	-2,8555084	7,77E-07
RAS p21 protein activator 2	*Rasa2*	-2,8390523	1,74E-05
Expressed sequence AI464131	*AI464131*	-2,838945	5,91E-07
Gap junction protein, beta 3	*Gjb3*	-2,8380223	9,94E-06
Peptide deformylase (mitochondrial)	*Pdf*	-2,8348689	7,17E-07
YTH domain family 2	*Ythdf2*	-2,8322903	4,71E-07
Guanine nucleotide binding protein (G protein), beta 4	*Gnb4*	-2,8220627	1,42E-05
Procollagen lysine, 2-oxoglutarate 5-dioxygenase 2	*Plod2*	-2,8215941	9,72E-07
Mitochondrial ribosomal protein L24	*Mrpl24*	-2,8212443	3,40E-07
Protein S (alpha)	*Pros1*	-2,8134557	2,90E-07
Acyl-coa synthetase long-chain family member 1	*Acsl1*	-2,8113495	7,69E-07
Epidermal growth factor receptor	*Egfr*	-2,8090294	8,23E-06
Neuropilin 1	*Nrp1*	-2,8046346	2,48E-06
URI1, prefoldin-like chaperone	*Uri1*	-2,7976304	1,03E-06
Phosphatidylinositol glycan anchor biosynthesis, class Q	*Pigq*	-2,7940169	2,15E-06
Predicted gene 13139	*Gm13139*	-2,7938064	8,70E-07
E26 avian leukemia oncogene 2, 3' domain	*Ets2*	-2,7758601	2,90E-07
Insulin-like growth factor binding protein 3	*Igfbp3*	-2,7741958	4,58E-06
Coiled-coil domain containing 80	*Ccdc80*	-2,7563342	9,86E-07
Glutamic pyruvate transaminase (alanine aminotransferase) 2	*Gpt2*	-2,7414893	5,20E-06
Spla/ryanodine receptor domain and SOCS box containing 4	*Spsb4*	-2,7362657	8,60E-06
Versican	*Vcan*	-2,7356903	8,73E-06
5'-nucleotidase, cytosolic II	*Nt5c2*	-2,7329358	2,98E-06
Cathepsin Z	*Ctsz*	-2,7304412	2,19E-07
Sulfide quinone reductase-like (yeast)	*Sqrdl*	-2,7283098	5,89E-07
Solute carrier family 20, member 2	*Slc20a2*	-2,7271153	1,78E-07
Presenilin 2	*Psen2*	-2,7183749	6,95E-06
Paired box 8	*Pax8*	-2,71497	1,65E-06
Dihydrolipoamide branched chain transacylase E2	*Dbt*	-2,7084896	7,59E-05
DNA segment, Chr 16, ERATO Doi 472, expressed	*D16Ertd472e*	-2,7045851	3,14E-06
Porcupine homolog (Drosophila)	*Porcn*	-2,7013688	3,33E-06
Sortilin 1	*Sort1*	-2,7010036	1,09E-05
Potassium inwardly-rectifying channel, subfamily J, member 15	*Kcnj15*	-2,6967151	5,35E-05
Solute carrier family 43, member 3	*Slc43a3*	-2,6806115	5,90E-06

Serine (or cysteine) peptidase inhibitor, clade H, member 1	*Serpinh1*	-2,677629	3,90E-07
PAX3 and PAX7 binding protein 1	*Paxbp1*	-2,6755826	8,59E-06
Proteoglycan 4 (megakaryocyte stimulating factor, articular superficial zone protein)	*Prg4*	-2,6647731	2,94E-05
Histone cluster 3, h2a	*Hist3h2a*	-2,6644495	3,53E-06
Ferric-chelate reductase 1	*Frrs1*	-2,6628341	3,11E-06
Transmembrane protein 203	*Tmem203*	-2,6610273	1,19E-06
Glycosyltransferase 25 domain containing 1	*Glt25d1*	-2,6593628	6,46E-07
Interleukin enhancer binding factor 3	*Ilf3*	-2,6586241	2,63E-06
Leucine zipper protein 1	*Luzp1*	-2,6487573	5,28E-06
Nuclear protein transcription regulator 1	*Nupr1*	-2,6478122	2,79E-06
Trna methyltransferase 1	*Trmt1*	-2,6391709	4,52E-07
Acyl-coa synthetase long-chain family member 6	*Acsl6*	-2,6342857	3,17E-05
Interferon-induced protein with tetratricopeptide repeats 1	*Ifit1*	-2,6295644	7,30E-06
Calcium and integrin binding family member 2	*Cib2*	-2,6273139	1,59E-06
Growth arrest specific 1	*Gas1*	-2,6235678	2,19E-07
Gene trap ROSA 26, Philippe Soriano	*Gt(ROSA)26Sor*	-2,6205531	3,04E-06
Alanyl (membrane) aminopeptidase	*Anpep*	-2,6177025	0,00784359
Ribosomal protein L5 /// ribisomal protein L5, pseudogene 2	*Rpl5 /// Rpl5-ps2*	-2,6163981	3,67E-07
Transmembrane protein 223	*Tmem223*	-2,6114084	7,44E-07
Selenophosphate synthetase 2	*Sephs2*	-2,6102694	2,18E-07
Pecanex-like 4 (Drosophila)	*Pcnxl4*	-2,6083119	6,87E-07
STEAP family member 4	*Steap4*	-2,6072279	1,18E-06
Yamaguchi sarcoma viral (v-yes) oncogene homolog 1	*Yes1*	-2,5919534	0,00032245
Cyclin-dependent kinase inhibitor 2C (p18, inhibits CDK4)	*Cdkn2c*	-2,5902519	1,67E-07
Fin bud initiation factor homolog (zebrafish)	*Fibin*	-2,5865649	3,85E-05
PHD finger protein 10	*Phf10*	-2,5859632	1,28E-07
Interleukin 1 receptor, type I	*Il1r1*	-2,582533	1,73E-05
Radical S-adenosyl methionine domain containing 2	*Rsad2*	-2,5825223	6,79E-05
Junction-mediating and regulatory protein	*Jmy*	-2,5820926	0,00069019
Desmin	*Des*	-2,5784903	1,27E-05
CDK2 associated, cullin domain 1	*Cacul1*	-2,5777279	1,93E-06
Interferon gamma inducible protein 47	*Ifi47*	-2,5749327	7,69E-07
Interleukin 6	*Il6*	-2,569988	2,81E-05
Lysyl oxidase-like 4	*Loxl4*	-2,5675619	3,02E-05
Zinc finger protein 7	*Zfp7*	-2,5668639	0,00016932
Chromatin licensing and DNA replication factor 1	*Cdt1*	-2,566117	7,21E-07
Small nucleolar RNA host gene 5	*Snhg5*	-2,5624974	1,07E-07
Nuclear import 7 homolog (S. Cerevisiae)	*Nip7*	-2,5567988	1,41E-07
Cadherin 11 pseudogene /// cadherin 11 pseudogene /// cadherin-11-like	*2610005L07Rik /// 6820431F20Rik /// LOC101056094*	-2,5547309	4,43E-05
Hydroxysteroid (17-beta) dehydrogenase 11	*Hsd17b11*	-2,5538639	1,11E-05

Fibroblast growth factor binding protein 1	*Fgfbp1*	-2,5533545	6,31E-05
Phosphoserine phosphatase	*Psph*	-2,5391372	3,11E-07
4-aminobutyrate aminotransferase	*Abat*	-2,5333424	1,90E-05
Prolyl-trna synthetase (mitochondrial)(putative)	*Pars2*	-2,5327579	2,32E-06
Melanoma cell adhesion molecule	*Mcam*	-2,5320075	7,15E-05
Homeobox B7 /// homeobox B8	*Hoxb7 /// Hoxb8*	-2,5304344	0,00123002
RNA binding motif protein, X linked-like-1	*Rbmxl1*	-2,5286595	2,26E-07
Glucuronyl C5-epimerase	*Glce*	-2,525659	6,20E-07
Cleavage and polyadenylation specific factor 2	*Cpsf2*	-2,5253337	9,27E-07
Predicted gene 2808 /// tubby like protein 4	*Gm2808 /// Tulp4*	-2,5241583	6,78E-06
Zinc and ring finger 1	*Znrf1*	-2,5220784	5,57E-05
Epidermal growth factor-containing fibulin-like extracellular matrix protein 2	*Efemp2*	-2,5215573	3,90E-07
Mitochondrial ribosomal protein S31	*Mrps31*	-2,5199215	7,19E-07
Hook homolog 2 (Drosophila)	*Hook2*	-2,5180781	1,39E-06
Apurinic/apyrimidinic endonuclease 1	*Apex1*	-2,5155258	1,88E-07
Sorting nexin 18	*Snx18*	-2,5147908	2,51E-06
Nuclear factor I/X	*Nfix*	-2,5141724	5,21E-06
RIKEN cdna A430005L14 gene	*A430005L14Rik*	-2,513365	9,34E-06
Fibrillin 1	*Fbn1*	-2,5128841	1,58E-05
ST3 beta-galactoside alpha-2,3-sialyltransferase 2	*St3gal2*	-2,5122867	5,15E-07
Major facilitator superfamily domain containing 6	*Mfsd6*	-2,5106805	8,89E-06
RAB20, member RAS oncogene family	*Rab20*	-2,5103257	1,77E-05
TGFB-induced factor homeobox 1	*Tgif1*	-2,5070981	1,69E-06
DNA-damage regulated autophagy modulator 1	*Dram1*	-2,4995019	7,39E-07
UDP-glcnac:betagal beta-1,3-N-acetylglucosaminyltransferase 3	*B3gnt3*	-2,4944699	5,50E-05
Glycerophosphodiester phosphodiesterase domain containing 1	*Gdpd1*	-2,4917834	5,33E-06
TNFRSF1A-associated via death domain	*Tradd*	-2,4890379	4,75E-05
Calcium homeostasis modulator 2	*Calhm2*	-2,4863059	1,73E-06
Niemann-Pick type C2	*Npc2*	-2,4859404	6,09E-07
Cytochrome c oxidase assembly factor 7	*Coa7*	-2,4820785	8,70E-07
OTU domain containing 4	*Otud4*	-2,4807824	5,26E-07
Cadherin 11 pseudogene	*6820431F20Rik*	-2,4756552	9,81E-05
Bassoon	*Bsn*	-2,4741726	0,00073603
RAB8B, member RAS oncogene family	*Rab8b*	-2,4729647	2,27E-06
Tubulin, beta 2a, pseudogene 2 /// tubulin, beta 2B class IIB	*Tubb2a-ps2 /// Tubb2b*	-2,4726169	3,31E-05
Gamma-glutamylamine cyclotransferase	*Ggact*	-2,4622574	6,61E-05
Arginine/serine rich protein 1	*Rsrp1*	-2,4567834	7,24E-06
Laminin, alpha 2	*Lama2*	-2,4560485	3,04E-05
Nuclear factor of activated T cells, cytoplasmic, calcineurin dependent 3	*Nfatc3*	-2,4551426	1,14E-05
Chac, cation transport regulator 1	*Chac1*	-2,4536164	5,05E-07
Excision repair cross-complementing rodent repair deficiency, complementation group 6 like 2	*Ercc6l2*	-2,4491792	2,78E-05

H19, imprinted maternally expressed transcript /// microrna 675	*H19 /// Mir675*	-2,4420521	1,19E-05
Pyruvate dehydrogenase kinase, isoenzyme 3	*Pdk3*	-2,4390925	3,07E-06
Integrin alpha 3	*Itga3*	-2,4335973	0,00044514
Thioredoxin interacting protein	*Txnip*	-2,4317654	1,25E-06
IMP3, U3 small nucleolar ribonucleoprotein, homolog (yeast)	*Imp3*	-2,429415	9,51E-07
Gap junction protein, beta 4	*Gjb4*	-2,4288556	1,72E-06
Rho GDP dissociation inhibitor (GDI) gamma	*Arhgdig*	-2,4244167	9,86E-07
Collagen, type V, alpha 1	*Col5a1*	-2,4231274	6,26E-07
Amyloid beta (A4) precursor-like protein 1	*Aplp1*	-2,4204372	0,00031039
Podoplanin	*Pdpn*	-2,4166932	6,79E-07
SR-related CTD-associated factor 8	*Scaf8*	-2,4125178	5,71E-06
Collagen, type I, alpha 2	*Col1a2*	-2,4124913	7,92E-06
Rho gtpase activating protein 24	*Arhgap24*	-2,4118653	0,00012471
C-type lectin domain family 4, member e	*Clec4e*	-2,4103024	4,81E-06
Predicted pseudogene 4024 /// transmembrane emp24-like trafficking protein 10 (yeast)	*Gm4024 /// Tmed10*	-2,407312	1,82E-05
RAD9 homolog A	*Rad9a*	-2,4000829	7,21E-07
Small nucleolar RNA host gene 8	*Snhg8*	-2,3941895	4,81E-07
Cyclin-dependent kinase 2 interacting protein	*Cinp*	-2,3929391	2,06E-07
Eukaryotic translation initiation factor 4E binding protein 1	*Eif4ebp1*	-2,3915195	1,40E-06
NLR family member X1	*Nlrx1*	-2,3898469	1,64E-05
Pellino 2	*Peli2*	-2,3826465	7,22E-06
Annexin A8	*Anxa8*	-2,3773154	2,02E-06
UDP-N-acetyl-alpha-D-galactosamine: polypeptide N-acetylgalactosaminyltransferase 7	*Galnt7*	-2,3744862	9,22E-06
Guanine nucleotide binding protein (G protein), gamma 11	*Gng11*	-2,3730811	5,71E-06
Carbonyl reductase 3	*Cbr3*	-2,3716975	1,18E-05
Tripartite motif-containing 16	*Trim16*	-2,3700099	0,0003993
Interferon induced transmembrane protein 10	*Ifitm10*	-2,3683657	1,09E-05
RAB29, member RAS oncogene family	*Rab29*	-2,3611645	1,74E-05
Biglycan	*Bgn*	-2,36015	2,47E-06
Ras association (ralgds/AF-6) domain family member 5	*Rassf5*	-2,3598857	4,70E-05
Exoribonuclease 1	*Eri1*	-2,3581132	5,25E-06
SWA-70 protein	*Swap70*	-2,3573247	0,00010184
ATP5S-like	*Atp5sl*	-2,3560865	9,95E-06
Fatty acid desaturase 1	*Fads1*	-2,3482547	1,18E-06
Atpase family, AAA domain containing 3A	*Atad3a*	-2,3453573	3,79E-06
CCAAT/enhancer binding protein (C/EBP), beta	*Cebpb*	-2,3441637	3,72E-06
Disabled 2 interacting protein	*Dab2ip*	-2,3383266	3,05E-06
Cap methyltransferase 2	*Cmtr2*	-2,3335218	0,0014371
Phosphogluconate dehydrogenase	*Pgd*	-2,3295004	1,21E-05
Family with sequence similarity 69, member B	*Fam69b*	-2,3261738	2,19E-06
RAR-related orphan receptor alpha	*Rora*	-2,3216209	1,19E-05

MARVEL (membrane-associating) domain containing 1	*Marveld1*	-2,3206383	1,99E-06
Late cornified envelope 1I	*Lce1i*	-2,3200705	0,00043013
Interferon induced transmembrane protein 6	*Ifitm6*	-2,3194967	8,65E-06
TSC22 domain family, member 3	*Tsc22d3*	-2,3170017	3,76E-05
RCE1 homolog, prenyl protein peptidase (S. Cerevisiae)	*Rce1*	-2,315945	2,91E-06
Cdna sequence U90926	*U90926*	-2,3149534	0,00059321
Latrophilin 1	*Lphn1*	-2,3122615	8,09E-05
Complement component 2 (within H-2S)	*C2*	-2,3103564	4,93E-06
DAZ interacting protein 1	*Dzip1*	-2,3092763	0,00013925
KTI12 homolog, chromatin associated (S. Cerevisiae)	*Kti12*	-2,3075359	5,77E-06
Mitogen-activated protein kinase 12	*Mapk12*	-2,3064229	1,63E-05
N-acylsphingosine amidohydrolase 2	*Asah2*	-2,3048493	2,31E-05
Phosphoserine aminotransferase 1	*Psat1*	-2,3043242	7,08E-06
Androgen receptor	*Ar*	-2,2931722	0,00021087
Breast cancer metastasis-suppressor 1	*Brms1*	-2,2907519	2,22E-05
Cullin 7	*Cul7*	-2,2888134	1,11E-05
RAN guanine nucleotide release factor	*Rangrf*	-2,2886087	1,34E-06
Ajuba LIM protein	*Ajuba*	-2,2884952	1,52E-06
Cathepsin H	*Ctsh*	-2,2866001	2,07E-06
Dynein light chain Tctex-type 1F	*Dynlt1f*	-2,278813	1,39E-05
Seven in absentia 1A	*Siah1a*	-2,2758438	3,70E-06
Ras homolog enriched in brain like 1	*Rhebl1*	-2,2756448	4,36E-06
Ubiquitin A-52 residue ribosomal protein fusion product 1 pseudogene	*Gm11517*	-2,2748756	4,70E-06
Myelocytomatosis oncogene	*Myc*	-2,2714558	9,73E-06
Interferon (alpha and beta) receptor 2	*Ifnar2*	-2,2711201	4,77E-07
Insulin-like growth factor binding protein 2	*Igfbp2*	-2,2679605	8,26E-05
Interferon activated gene 204	*Ifi204*	-2,2622831	0,00284675
Branched chain aminotransferase 1, cytosolic	*Bcat1*	-2,261935	4,06E-06
Palladin, cytoskeletal associated protein	*Palld*	-2,2571822	2,48E-06
Nicotinamide N-methyltransferase	*Nnmt*	-2,2568981	1,56E-05
Wilms tumour 1-associating protein	*Wtap*	-2,2561245	1,55E-06
Retinoblastoma binding protein 6	*Rbbp6*	-2,2464027	7,10E-05
Family with sequence similarity 20, member C	*Fam20c*	-2,2462234	8,26E-05
Delta-like 1 (Drosophila)	*Dll1*	-2,2455847	1,85E-06
Zinc finger protein 568	*Zfp568*	-2,2450826	0,00209686
Scleraxis	*Scx*	-2,2428582	4,57E-05
Tubulointerstitial nephritis antigen-like 1	*Tinagl1*	-2,2417629	0,0002533
Dnaj (Hsp40) homolog, subfamily C, member 11	*Dnajc11*	-2,2388193	8,40E-07
Zinc finger protein 91 /// Zfp91-Cntf readthrough transcript (NMD candidate)	*Zfp91 /// Zfp91Cntf*	-2,2373494	0,00010288
Family with sequence similarity 136, member A	*Fam136a*	-2,2367972	1,07E-06
Post-GPI attachment to proteins 2	*Pgap2*	-2,2353238	4,04E-06
Trophoblast glycoprotein	*Tpbg*	-2,2299448	7,24E-06

Solute carrier family 3 (activators of dibasic and neutral amino acid transport), member 2	*Slc3a2*	-2,2282425	6,64E-07
STT3, subunit of the oligosaccharyltransferase complex, homolog A (S. Cerevisiae)	*Stt3a*	-2,2264849	0,00074953
Influenza virus NS1A binding protein	*Ivns1abp*	-2,2262983	8,64E-06
Deoxyhypusine synthase	*Dhps*	-2,2254598	2,29E-05
Cyclin-dependent kinase 6	*Cdk6*	-2,2213463	0,00070081
YTH domain family 1	*Ythdf1*	-2,2185356	5,51E-06
SMEK homolog 1, suppressor of mek1 (Dictyostelium)	*Smek1*	-2,2177674	2,08E-06
Centromere protein V	*Cenpv*	-2,2175872	4,85E-06
Solute carrier family 29 (nucleoside transporters), member 1	*Slc29a1*	-2,2131474	2,79E-06
DEAD (Asp-Glu-Ala-Asp) box polypeptide 18	*Ddx18*	-2,2127262	0,00011771
Microfibrillar-associated protein 3	*Mfap3*	-2,2118992	1,72E-05
TATA box binding protein (Tbp)-associated factor, RNA polymerase I, D	*Taf1d*	-2,2104737	0,00359967
Crystallin, zeta (quinone reductase)-like 1	*Cryzl1*	-2,2088259	1,05E-06
BAI1-associated protein 2-like 1	*Baiap2l1*	-2,2079877	1,73E-06
G0/G1 switch gene 2	*G0s2*	-2,2027006	3,18E-06
ER lipid raft associated 1	*Erlin1*	-2,2008449	1,99E-06
Poly (A) polymerase alpha	*Papola*	-2,2000658	0,00298998
Leucine rich repeat containing 16A	*Lrrc16a*	-2,1995341	2,33E-05
Retinitis pigmentosa gtpase regulator	*Rpgr*	-2,1935775	5,86E-05
Solute carrier family 25, member 33	*Slc25a33*	-2,1907789	4,88E-06
UDP-Gal:betaglcnac beta 1,4-galactosyltransferase, polypeptide 4	*B4galt4*	-2,1885944	2,59E-05
SET domain containing 6	*Setd6*	-2,1870104	1,17E-05
Camello-like 1	*Cml1*	-2,1863182	0,00010932
General transcription factor IIA, 1-like	*Gtf2a1l*	-2,1861162	1,66E-05
Tubulin, alpha 8	*Tuba8*	-2,1845513	1,44E-05
Pinin	*Pnn*	-2,1841531	9,73E-05
TEA domain family member 2	*Tead2*	-2,1837944	1,31E-05
G protein-coupled receptor 124	*Gpr124*	-2,1822874	2,08E-05
Lysyl oxidase-like 1	*Loxl1*	-2,180683	4,85E-05
Proteasome (prosome, macropain) 26S subunit, non-atpase, 7	*Psmd7*	-2,180414	1,11E-06
CD276 antigen	*Cd276*	-2,1786606	0,00015038
Expressed sequence AI837181	*AI837181*	-2,1784216	2,04E-05
Activating transcription factor 4	*Atf4*	-2,1781216	7,26E-06
Adenylate kinase isoenzyme 6 /// TAF9 RNA polymerase II, TATA box binding protein (TBP)-associated factor	*Ak6 /// Taf9*	-2,1773115	1,55E-06
TEA domain family member 4	*Tead4*	-2,1773112	6,69E-06
Transcription factor 7 like 1 (T cell specific, HMG box)	*Tcf7l1*	-2,1747824	0,00059926
Cytochrome c oxidase subunit vib polypeptide 2	*Cox6b2*	-2,1722717	0,00036428
RIKEN cdna 4921524J17 gene	*4921524J17Rik*	-2,1716119	0,0003008
Cyclin J	*Ccnj*	-2,1672591	0,00026232
Odd-skipped related 1 (Drosophila)	*Osr1*	-2,1671262	0,00011683

RIKEN cdna 1700017B05 gene	*1700017B05Rik*	-2,1652431	7,70E-06
Mannan-binding lectin serine peptidase 1	*Masp1*	-2,1641861	1,37E-06
Solute carrier family 2 (facilitated glucose transporter), member 1	*Slc2a1*	-2,1626391	1,18E-06
A disintegrin and metallopeptidase domain 9 (meltrin gamma)	*Adam9*	-2,1611491	4,12E-05
Alpha glucosidase 2 alpha neutral subunit	*Ganab*	-2,1602599	4,00E-06
Zinc finger protein 131	*Zfp131*	-2,1582565	1,15E-05
Family with sequence similarity 60, member A	*Fam60a*	-2,1574236	4,01E-06
Membrane-associated ring finger (C3HC4) 5	*III.05*	-2,1572342	7,15E-07
Zinc finger protein 566	*Zfp566*	-2,1570831	1,57E-05
Endoplasmic reticulum metallopeptidase 1	*Ermp1*	-2,1549717	1,02E-05
Coagulation factor II (thrombin) receptor	*F2r*	-2,1548038	1,98E-05
Camp responsive element binding protein 3-like 1	*Creb3l1*	-2,153368	1,60E-05
Sema domain, immunoglobulin domain (Ig), short basic domain, secreted, (semaphorin) 3C	*Sema3c*	-2,1533006	0,00061747
Membrane-bound transcription factor peptidase, site 1	*Mbtps1*	-2,1532607	9,83E-07
Spermatogenesis associated 13	*Spata13*	-2,1529286	1,05E-05
Mannosidase, beta A, lysosomal	*Manba*	-2,1527592	0,0012599
Microtubule-associated protein 1 light chain 3 beta	*Map1lc3b*	-2,1522459	9,96E-07
Feminization 1 homolog b (C. Elegans)	*Fem1b*	-2,1514226	0,00013925
Barrier to autointegration factor 1	*Banf1*	-2,1499748	1,71E-06
Astrotactin 1	*Astn1*	-2,1435955	9,95E-05
Protein phosphatase 1, regulatory (inhibitor) subunit 14c	*Ppp1r14c*	-2,142892	6,57E-05
Latent transforming growth factor beta binding protein 3	*Ltbp3*	-2,1425737	1,39E-06
Tousled-like kinase 2 (Arabidopsis)	*Tlk2*	-2,1423213	2,12E-05
YY1 transcription factor	*Yy1*	-2,1406352	4,17E-06
Interferon gamma induced gtpase	*Igtp*	-2,1401186	0,00020653
Death-associated protein kinase 2	*Dapk2*	-2,1383017	3,43E-06
Predicted gene 5806 /// thymine DNA glycosylase pseudogene /// thymine DNA glycosylase	*Gm5806 /// Gm9855 /// Tdg*	-2,1381554	1,92E-05
Glutathione S-transferase, alpha 2 (Yc2)	*Gsta2*	-2,1373803	0,00014204
Cirrhosis, autosomal recessive 1A (human)	*Cirh1a*	-2,1370915	1,56E-06
RIKEN cdna 1190005I06 gene	*1190005I06Rik*	-2,1364077	1,01E-05
Caspase 7	*Casp7*	-2,1349297	8,65E-06
Leptin receptor overlapping transcript-like 1	*Leprotl1*	-2,1290083	5,19E-06
N-acylsphingosine amidohydrolase 1	*Asah1*	-2,1289917	3,52E-06
RIKEN cdna 0610012G03 gene	*0610012G03Rik*	-2,1277981	4,95E-05
Golgi associated, gamma adaptin ear containing, ARF binding protein 2	*Gga2*	-2,1243881	1,96E-06
Zinc finger protein OZF-like	*LOC102643033*	-2,1234813	0,0030023
RNA terminal phosphate cyclase-like 1	*Rcl1*	-2,1196073	5,19E-07
Coiled-coil domain containing 86	*Ccdc86*	-2,1152319	2,63E-05
Epithelial splicing regulatory protein 2	*Esrp2*	-2,1147001	0,00498451
Zinc finger protein 639	*Zfp639*	-2,1102885	6,20E-07

Rho gtpase activating protein 5	*Arhgap5*	-2,1097987	1,88E-05
Diacylglycerol O-acyltransferase 2	*Dgat2*	-2,1091764	2,60E-05
Synaptotagmin-like 2	*Sytl2*	-2,1069587	3,14E-05
Neuropilin 2	*Nrp2*	-2,1066985	6,26E-05
Isovaleryl coenzyme A dehydrogenase	*Ivd*	-2,1044897	1,72E-05
Coiled-coil domain containing 186	*Ccdc186*	-2,1037913	1,50E-05
Epiregulin	*Ereg*	-2,1017689	5,72E-05
Interleukin 4 receptor, alpha	*Il4ra*	-2,099489	6,63E-06
Tubulin polymerization-promoting protein family member 3	*Tppp3*	-2,0989606	9,72E-05
Metastasis associated 1	*Mta1*	-2,0981072	2,63E-05
Ribonuclease T2A /// ribonuclease T2B	*Rnaset2a /// Rnaset2b*	-2,0965163	3,42E-05
Protein C receptor, endothelial	*Procr*	-2,0950723	1,13E-05
Purine rich element binding protein B	*Purb*	-2,0928686	4,32E-05
Toll-like receptor 3	*Tlr3*	-2,092708	0,00012199
Caspase 3	*Casp3*	-2,0926138	5,07E-06
Regulator of chromosome condensation 2	*Rcc2*	-2,0912309	3,87E-06
Mannoside acetylglucosaminyltransferase 4, isoenzyme B	*Mgat4b*	-2,0903379	1,28E-05
Prolylcarboxypeptidase (angiotensinase C)	*Prcp*	-2,0876197	3,59E-05
Chemokine (C-C motif) ligand 25	*Ccl25*	-2,0852887	9,82E-06
Histocompatibility 2, Q region locus 4 /// histocompatibility 2, Q region locus 6 /// histocompatibility 2, Q region locus 8 /// histocompatibility 2, Q region locus 6-like	*H2-Q4 /// H2-Q6 /// H2-Q8 /// LOC68395*	-2,0841086	1,67E-05
Frequently rearranged in advanced T cell lymphomas 2	*Frat2*	-2,0811141	1,31E-05
Ezrin	*Ezr*	-2,0799211	3,16E-06
Transforming growth factor alpha	*Tgfa*	-2,0731377	0,00129175
Abhydrolase domain containing 5	*Abhd5*	-2,0729977	8,45E-07
Notch 3	*Notch3*	-2,0721406	0,00015064
Glutamate-rich WD repeat containing 1	*Grwd1*	-2,0707029	1,27E-05
Zinc finger protein 318	*Zfp318*	-2,0689384	0,00086502
Rosbin, round spermatid basic protein 1	*Rsbn1*	-2,0679548	3,40E-06
Inhibitor of Bruton agammaglobulinemia tyrosine kinase	*Ibtk*	-2,0645838	0,00175189
Glutathione S-transferase omega 1	*Gsto1*	-2,0618304	5,42E-06
Suppressor of variegation 4-20 homolog 1 (Drosophila)	*Suv420h1*	-2,0611595	1,50E-05
R-spondin homolog (Xenopus laevis)	*Rspo1*	-2,0607306	0,00156369
Dipeptidylpeptidase 3	*Dpp3*	-2,0587912	3,85E-06
Transforming growth factor, beta 3	*Tgfb3*	-2,0558948	9,73E-06
Alanyl-trna synthetase	*Aars*	-2,0548343	1,47E-05
Eukaryotic translation initiation factor 3, subunit A	*Eif3a*	-2,0544919	0,00013829
Exocyst complex component 8	*Exoc8*	-2,0542678	1,22E-05
Kelch-like 24	*Klhl24*	-2,0537105	0,00146583
Zinc finger homeobox 3	*Zfhx3*	-2,0534941	0,00010462
RNA binding motif protein 43	*Rbm43*	-2,0533033	0,00212817

Hypoxia inducible factor 1, alpha subunit	*Hif1a*	-2,0511216	0,00035895
Transmembrane protein 123	*Tmem123*	-2,050726	0,00018567
Lysophosphatidylcholine acyltransferase 3	*Lpcat3*	-2,0506452	2,04E-06
Male-specific lethal 2 homolog (Drosophila)	*Msl2*	-2,0500997	2,40E-05
Phospholipase A2, group XV	*Pla2g15*	-2,0495195	3,06E-06
Apolipoprotein B mrna editing enzyme, catalytic polypeptide 1	*Apobec1*	-2,0491901	1,26E-05
Dicer 1, ribonuclease type III	*Dicer1*	-2,0484952	6,28E-06
Cysteine rich protein 2	*Crip2*	-2,0483604	0,00017404
Interferon-related developmental regulator 2	*Ifrd2*	-2,047077	8,49E-06
Adenylate kinase 2	*Ak2*	-2,0422601	1,40E-05
Signal sequence receptor, beta	*Ssr2*	-2,0420662	2,06E-06
Glutathione reductase	*Gsr*	-2,038195	6,27E-05
Phosphatase domain containing, paladin 1 /// thrombospondin 1	*Pald1 /// Thbs1*	-2,0367303	5,73E-06
Casein kinase 2, alpha prime polypeptide	*Csnk2a2*	-2,0364723	2,43E-06
Protein tyrosine phosphatase-like (proline instead of catalytic arginine), member b	*Ptplb*	-2,0353381	1,35E-05
Syntrophin, basic 2	*Sntb2*	-2,0349153	0,00019014
CTF8, chromosome transmission fidelity factor 8	*Chtf8*	-2,0345736	2,79E-06
Zinc finger protein 148	*Zfp148*	-2,0329677	2,39E-05
Friend leukemia integration 1	*Fli1*	-2,0320602	0,00018975
Stearoyl-Coenzyme A desaturase 2	*Scd2*	-2,0318512	0,00297705
Cleavage stimulation factor, 3' pre-RNA subunit 2, tau	*Cstf2t*	-2,031472	3,44E-06
Nucleolar complex associated 2 homolog (S. Cerevisiae)	*Noc2l*	-2,0258986	9,75E-06
Cell division cycle associated 4	*Cdca4*	-2,0257326	6,40E-05
Mitochondrial ribosomal protein L20	*Mrpl20*	-2,0256694	4,06E-06
Nucleolar and coiled-body phosphoprotein 1	*Nolc1*	-2,0251417	6,50E-06
Phosphatidylinositol glycan anchor biosynthesis, class K	*Pigk*	-2,0237278	3,16E-06
SET domain, bifurcated 1	*Setdb1*	-2,0234214	1,17E-05
Ubia prenyltransferase domain containing 1	*Ubiad1*	-2,0206232	1,78E-05
Polypyrimidine tract binding protein 3	*Ptbp3*	-2,0181206	0,00128012
Nucleoplasmin 3 /// nucleoplasmin 3, pseudogene 1	*Npm3 /// Npm3-ps1*	-2,0177671	0,00022773
Transmembrane protein 50B	*Tmem50b*	-2,0173611	3,70E-06
Ubiquitin specific peptidase 1	*Usp1*	-2,0163958	0,00025424
Leprecan 1	*Lepre1*	-2,0155617	1,76E-06
High mobility group box 2	*Hmgb2*	-2,0128018	7,86E-06
Solute carrier family 1 (glutamate/neutral amino acid transporter), member 4	*Slc1a4*	-2,0111929	1,35E-05
Adiponectin receptor 1	*Adipor1*	-2,0103741	2,77E-05
N(alpha)-acetyltransferase 30, natc catalytic subunit	*Naa30*	-2,007971	0,00043271
Transmembrane protein 11	*Tmem11*	-2,0067936	8,55E-06
Ubiquitin-conjugating enzyme E2J 2	*Ube2j2*	-2,0058429	3,93E-06
Son DNA binding protein	*Son*	-2,0050959	2,86E-05
Vacuolar protein sorting 37A (yeast)	*Vps37a*	-2,0042718	2,43E-05

Transmembrane protein 161A	*Tmem161a*	-2,0032236	1,47E-06
RIKEN cdna 1700019G17 gene	*1700019G17Rik*	-2,0022217	9,88E-05
Predicted gene 1976	*Gm1976*	-2,0017988	0,00040135
Serine carboxypeptidase 1	*Scpep1*	-2,0014988	6,03E-05
Glutathione transferase zeta 1 (maleylacetoacetate isomerase)	*Gstz1*	-2,0000087	0,00025625
Cerebral cavernous malformation 2	*Ccm2*	-1,998478	1,57E-05
Phosphatidylinositol transfer protein, membrane-associated 1	*Pitpnm1*	-1,9978405	1,09E-05
Purinergic receptor P2X, ligand-gated ion channel, 3	*P2rx3*	-1,9969497	0,00059623
Islet cell autoantigen 1	*Ica1*	-1,9962745	0,00089558
Left right determination factor 1	*Lefty1*	-1,9960439	1,02E-05
Gem (nuclear organelle) associated protein 7	*Gemin7*	-1,9959682	5,50E-05
3-phosphoglycerate dehydrogenase	*Phgdh*	-1,9934173	2,82E-06
Killer cell lectin-like receptor subfamily G, member 1	*Klrg1*	-1,9932304	6,74E-05
RER1 retention in endoplasmic reticulum 1 homolog (S. Cerevisiae)	*Rer1*	-1,9925884	4,36E-06
T cell lymphoma invasion and metastasis 1	*Tiam1*	-1,9920433	0,00048613
Dual-specificity tyrosine-(Y)-phosphorylation regulated kinase 1a	*Dyrk1a*	-1,9907922	2,88E-06
Cytochrome b5 type B	*Cyb5b*	-1,9899438	1,96E-05
Chromodomain helicase DNA binding protein 1	*Chd1*	-1,9876171	4,45E-06
Predicted gene 5595	*Gm5595*	-1,9871537	0,00049622
Egl-9 family hypoxia-inducible factor 3	*Egln3*	-1,9846993	0,00048245
Listerin E3 ubiquitin protein ligase 1	*Ltn1*	-1,981966	0,00141013
CCR4 carbon catabolite repression 4-like (S. Cerevisiae)	*Ccrn4l*	-1,9806347	4,03E-05
Chemokine (C-C motif) ligand 5	*Ccl5*	-1,9801157	8,77E-05
RIKEN cdna 9430015G10 gene	*9430015G10Rik*	-1,9795589	0,00027118
Mastermind like 1 (Drosophila)	*Maml1*	-1,9788044	0,00014418
Programmed cell death 4	*Pdcd4*	-1,9782017	4,07E-06
Ephrin A1	*Efna1*	-1,9775069	6,79E-06
Dynein light chain LC8-type 2	*Dynll2*	-1,9773674	1,10E-05
Bicaudal D homolog 1 (Drosophila)	*Bicd1*	-1,9773049	5,89E-05
Mediator complex subunit 26	*Med26*	-1,9772637	3,62E-05
Solute carrier family 25, member 37	*Slc25a37*	-1,9771933	2,01E-05
Solute carrier organic anion transporter family, member 1a5	*Slco1a5*	-1,9768397	2,05E-05
Expressed sequence AW209491	*AW209491*	-1,9760025	1,90E-05
Ribosomal protein L14	*Rpl14*	-1,9733875	2,51E-05
RAD23a homolog (S. Cerevisiae)	*Rad23a*	-1,9727949	0,00025706
Phospholipase C, delta 1	*Plcd1*	-1,9721871	1,75E-05
Rho gtpase activating protein 29	*Arhgap29*	-1,9717006	0,00015709
Survival motor neuron domain containing 1	*Smndc1*	-1,9710302	9,76E-06
Ephrin B2	*Efnb2*	-1,9687752	2,18E-06
Interleukin-1 receptor-associated kinase 1 binding protein 1	*Irak1bp1*	-1,967957	1,77E-05
DEAD (Asp-Glu-Ala-Asp) box polypeptide 19b	*Ddx19b*	-1,9675151	2,02E-05

Sec61, alpha subunit 2 (S. Cerevisiae)	*Sec61a2*	-1,9669509	0,00032787
Immunity-related gtpase family M member 2	*Irgm2*	-1,966839	0,00028602
SWI/SNF related, matrix associated, actin dependent regulator of chromatin, subfamily e, member 1	*Smarce1*	-1,9660569	0,00012338
Interleukin 1 receptor-like 1	*Il1rl1*	-1,9659781	2,29E-05
Beta galactoside alpha 2,6 sialyltransferase 1	*St6gal1*	-1,9647787	5,60E-06
Interferon-related developmental regulator 1	*Ifrd1*	-1,9612988	9,20E-06
Ribosomal RNA processing 1 homolog B (S. Cerevisiae)	*Rrp1b*	-1,9612741	1,11E-05
Mitochondrial ribosomal protein L50	*Mrpl50*	-1,9599644	0,00014204
FK506 binding protein 10	*Fkbp10*	-1,9583838	0,00050638
UFM1-specific peptidase 1	*Ufsp1*	-1,9555194	3,25E-05
Zinc finger protein 930	*Zfp930*	-1,9534159	0,00954367
Zinc finger and BTB domain containing 12	*Zbtb12*	-1,9526047	0,00050003
Interferon (alpha and beta) receptor 1	*Ifnar1*	-1,9523812	1,45E-05
FK506 binding protein-like	*Fkbpl*	-1,9521554	1,41E-05
TAF5-like RNA polymerase II, p300/CBP-associated factor (PCAF)-associated factor	*Taf5l*	-1,9508759	6,78E-06
Apolipoprotein B receptor	*Apobr*	-1,9507387	1,09E-05
Atpase type 13A3	*Atp13a3*	-1,9503173	5,97E-05
Tankyrase, TRF1-interacting ankyrin-related ADP-ribose polymerase 2	*Tnks2*	-1,9492552	3,65E-06
Membrane-spanning 4-domains, subfamily A, member 4D	*Ms4a4d*	-1,948877	0,00012009
Plexin D1	*Plxnd1*	-1,9485929	9,82E-05
Tripartite motif-containing 8	*Trim8*	-1,944568	1,51E-05
Iroquois related homeobox 1 (Drosophila)	*Irx1*	-1,9436566	7,59E-06
Fc receptor, igg, alpha chain transporter	*Fcgrt*	-1,9423897	9,21E-05
Sperm autoantigenic protein 17	*Spa17*	-1,9405339	0,00047138
NFKB activating protein-like	*Nkapl*	-1,9387444	7,91E-05
WD repeat domain 20	*Wdr20*	-1,938121	6,26E-06
1-acylglycerol-3-phosphate O-acyltransferase 5 (lysophosphatidic acid acyltransferase, epsilon)	*Agpat5*	-1,9379353	0,00037774
TRAF-interacting protein with forkhead-associated domain	*Tifa*	-1,937698	0,00166849
SKI-like	*Skil*	-1,9367273	3,10E-06
Mitogen-activated protein kinase 11	*Mapk11*	-1,9361098	0,0001196
Poly(A) binding protein, cytoplasmic 4	*Pabpc4*	-1,9352449	9,38E-06
Membrane bound O-acyltransferase domain containing 1	*Mboat1*	-1,934377	3,15E-06
Torsin A interacting protein 2	*Tor1aip2*	-1,9336605	1,49E-05
ATP-binding cassette, sub-family G (WHITE), member 1	*Abcg1*	-1,9333346	0,00018954
NEDD4 binding protein 2-like 1	*N4bp2l1*	-1,9326119	0,0005183
Syndecan 1	*Sdc1*	-1,9299786	2,79E-06
Like-glycosyltransferase	*Large*	-1,9291926	0,00056362
Phosphatase and tensin homolog	*Pten*	-1,9282955	4,95E-05
Human immunodeficiency virus type I enhancer binding protein 2	*Hivep2*	-1,9256705	6,35E-06

Eukaryotic translation initiation factor 2, subunit 2 (beta)	*Eif2s2*	-1,9256109	0,00313708
RIKEN cdna 2310036O22 gene	*2310036O22Rik*	-1,9232018	8,02E-06
Transmembrane protein 60	*Tmem60*	-1,9217652	1,48E-05
Dual specificity phosphatase 16	*Dusp16*	-1,9209266	6,57E-06
ATP-binding cassette, sub-family E (OABP), member 1	*Abce1*	-1,9204339	1,49E-05
Phosphodiesterase 10A	*Pde10a*	-1,9201709	0,00137373
Suppressor of fused homolog (Drosophila)	*Sufu*	-1,9200926	2,83E-05
RAS p21 protein activator 3	*Rasa3*	-1,9194502	3,70E-06
Ras and Rab interactor 1	*Rin1*	-1,9168035	1,96E-05
WD repeat domain 43	*Wdr43*	-1,9165637	9,05E-05
Queuine trna-ribosyltransferase 1	*Qtrt1*	-1,9162744	1,73E-05
Aldo-keto reductase family 1, member B3 (aldose reductase) /// Akr1b3 pseudogene	*Akr1b3 /// Gm6644*	-1,9162606	1,23E-05
Kruppel-like factor 13	*Klf13*	-1,9151687	7,50E-05
RIKEN cdna 2510039O18 gene	*2510039O18Rik*	-1,9143506	2,45E-05
Cyclin L2	*Ccnl2*	-1,9138611	5,22E-05
Predicted gene 2701 /// NADH dehydrogenase [ubiquinone] 1 beta subcomplex subunit 1-like	*Gm2701 /// LOC102631912*	-1,9130628	2,78E-05
Bbsome interacting protein 1	*Bbip1*	-1,912649	0,00025681
Processing of precursor 4, ribonuclease P/MRP family, (S. Cerevisiae)	*Pop4*	-1,911846	2,72E-05
CD40 antigen	*Cd40*	-1,9091836	0,00501436
Soc-2 (suppressor of clear) homolog (C. Elegans)	*Shoc2*	-1,9061595	0,00538098
Small nucleolar RNA host gene 6	*Snhg6*	-1,90599	4,88E-06
3-phosphoglycerate dehydrogenase pseudogene /// 3-phosphoglycerate dehydrogenase	*Gm8096 /// Phgdh*	-1,9058078	3,02E-06
Collagen, type I, alpha 1	*Col1a1*	-1,905485	8,22E-05
Glycerol-3-phosphate acyltransferase, mitochondrial	*Gpam*	-1,9046473	7,44E-06
Phosphodiesterase 4D interacting protein (myomegalin)	*Pde4dip*	-1,9042666	0,00091495
ATP-binding cassette, sub-family C (CFTR/MRP), member 1	*Abcc1*	-1,9032049	0,00576044
Zinc finger protein 707	*Zfp707*	-1,9026689	5,07E-05
2-oxoglutarate and iron-dependent oxygenase domain containing 3	*Ogfod3*	-1,9023459	6,38E-05
NADH dehydrogenase (ubiquinone) flavoprotein 1	*Ndufv1*	-1,9002117	2,21E-05
Immediate early response 2	*Ier2*	-1,8989391	0,00010286
Lon peptidase 1, mitochondrial	*Lonp1*	-1,89844	1,67E-05
Histone cluster 1, H3a /// histone cluster 1, H3b /// histone cluster 1, H3c /// histone cluster 1, H3d /// histone cluster 1, H3e /// histone cluster 1, H3f /// histone cluster 1, H3g /// histone cluster 1, H3h /// histone cluster 1, H3i /// histone cluster 2, H3b /// histone cluster 2, H3c1 /// histone cluster 2, H3c2	*Hist1h3a /// Hist1h3b /// Hist1h3c /// Hist1h3d /// Hist1h3e /// Hist1h3f /// Hist1h3g /// Hist1h3h /// Hist1h3i /// Hist2h3b /// Hist2h3c1 /// Hist2h3c2*	-1,8983715	0,00012003

Atpase family, AAA domain containing 1	*Atad1*	-1,8963062	0,00032806
Secretory carrier membrane protein 5	*Scamp5*	-1,8953707	6,89E-05
Collagen, type VI, alpha 2	*Col6a2*	-1,8952227	0,00162614
NAD kinase	*Nadk*	-1,8948904	7,04E-06
Ubiquitin specific peptidase 38	*Usp38*	-1,8948844	0,00010841
Procollagen-lysine, 2-oxoglutarate 5-dioxygenase 1	*Plod1*	-1,8947583	7,59E-06
Bromodomain containing 7	*Brd7*	-1,8943473	1,44E-05
Glutaryl-Coenzyme A dehydrogenase	*Gcdh*	-1,8938528	7,84E-05
DNA cross-link repair 1A, PSO2 homolog (S. Cerevisiae)	*Dclre1a*	-1,8931896	0,00019256
Ribonuclease H2, subunit C	*Rnaseh2c*	-1,8922329	0,00017126
Solute carrier family 25 (mitochondrial carrier, palmitoylcarnitine transporter), member 29	*Slc25a29*	-1,8907505	2,60E-05
Cleavage and polyadenylation specific factor 7	*Cpsf7*	-1,8890977	6,19E-06
Transcription factor AP-2, gamma	*Tfap2c*	-1,8887532	0,002959
Cadherin 11 pseudogene	*2610005L07Rik*	-1,8881062	0,00012082
Multiple inositol polyphosphate histidine phosphatase 1	*Minpp1*	-1,8880124	0,00021378
Chloride channel 5	*Clcn5*	-1,8862205	7,70E-06
Dystroglycan 1	*Dag1*	-1,8861204	1,61E-05
Interactor of little elongation complex ELL subunit 1	*Ice1*	-1,8830364	1,44E-05
Ubiquitin specific peptidase 10	*Usp10*	-1,8825579	9,36E-05
Rbm14-Rbm4 readthrough /// RNA binding motif protein 4	*Rbm14-rbm4 /// Rbm4*	-1,8811303	4,26E-05
Pleckstrin homology domain containing, family F (with FYVE domain) member 2	*Plekhf2*	-1,8809733	0,00069496
Glutamine and serine rich 1	*Qser1*	-1,8804835	0,00016329
RIKEN cdna 2810004N23 gene	*2810004N23Rik*	-1,8801819	5,18E-06
Expressed sequence AI462493	*AI462493*	-1,8790461	6,41E-06
Methylthioadenosine phosphorylase	*Mtap*	-1,8787518	8,10E-05
Helicase, lymphoid specific	*Hells*	-1,8776583	0,00010505
Contactin associated protein-like 4	*Cntnap4*	-1,8767297	0,00035172
Peroxiredoxin 3	*Prdx3*	-1,8757572	9,77E-06
Lysophosphatidic acid receptor 1	*Lpar1*	-1,8740904	3,33E-05
Poly(A) binding protein, nuclear 1	*Pabpn1*	-1,8728842	0,00043953
Cache domain containing 1	*Cachd1*	-1,8725484	7,95E-06
Leucine-rich PPR-motif containing	*Lrpprc*	-1,8717353	4,28E-05
NIMA (never in mitosis gene a)-related expressed kinase 3	*Nek3*	-1,8709677	9,62E-05
Ski sarcoma viral oncogene homolog (avian)	*Ski*	-1,8706676	4,06E-06
Mitochondria localized glutamic acid rich protein	*Mgarp*	-1,870285	0,0001531
Ring finger and SPRY domain containing 1	*Rspry1*	-1,867422	2,42E-05
Hexokinase 2	*Hk2*	-1,8671849	2,86E-05
Golgi apparatus protein 1	*Glg1*	-1,8665387	1,18E-05
Epidermal growth factor receptor pathway substrate 15	*Eps15*	-1,8664199	2,28E-05
PHD finger protein 13	*Phf13*	-1,8658612	8,27E-06
Rap1 interacting factor 1 homolog (yeast)	*Rif1*	-1,8656855	0,00184519

Forkhead box F2	*Foxf2*	-1,865015	0,00014513
Fas apoptotic inhibitory molecule	*Faim*	-1,8649234	1,91E-05
CD2-associated protein	*Cd2ap*	-1,8645972	0,00141248
Galactose mutarotase	*Galm*	-1,8617269	0,00016161
Orosomucoid 1	*Orm1*	-1,8614926	0,00011127
Tyrosyl-trna synthetase	*Yars*	-1,8609408	5,26E-06
Phosphatidylinositol 3-kinase, C2 domain containing, alpha polypeptide	*Pik3c2a*	-1,8592128	0,0001208
Acyl-coa thioesterase 6	*Acot6*	-1,8590151	1,59E-05
Fatty acid desaturase 3	*Fads3*	-1,8589021	7,83E-06
Forkhead box N2	*Foxn2*	-1,8569667	9,67E-05
Myosin, light polypeptide kinase	*Mylk*	-1,8557178	0,00022638
Heat shock protein 5	*Hspa5*	-1,8550896	2,21E-05
NEDD4 binding protein 1	*N4bp1*	-1,8550537	3,47E-06
CD302 antigen	*Cd302*	-1,8547174	0,00026742
Patatin-like phospholipase domain containing 8	*Pnpla8*	-1,8540058	9,74E-05
Zinc finger and BTB domain containing 21	*Zbtb21*	-1,8537072	0,00038911
Bone morphogenetic protein receptor, type 1A	*Bmpr1a*	-1,8532193	5,75E-05
Endonuclease domain containing 1	*Endod1*	-1,852876	2,35E-05
MAK16 homolog (S. Cerevisiae)	*Mak16*	-1,8520222	1,42E-05
Platelet-derived growth factor, C polypeptide	*Pdgfc*	-1,8516917	4,90E-05
Zinc finger protein 428	*Zfp428*	-1,8484631	0,00021608
Heat shock protein family B (small), member 11	*Hspb11*	-1,8481158	5,27E-05
Predicted pseudogene 5963 /// ribosomal protein S21	*Gm5963 /// Rps21*	-1,846137	0,00274762
Chondroitin sulfate N-acetylgalactosaminyltransferase 1	*Csgalnact1*	-1,8459159	2,22E-05
GRP1 (general receptor for phosphoinositides 1)-associated scaffold protein	*Grasp*	-1,8444318	0,0002328
Frataxin	*Fxn*	-1,8421148	2,58E-05
U7 snrnp-specific Sm-like protein LSM10	*Lsm10*	-1,841545	9,38E-06
Zinc finger protein 777	*Zfp777*	-1,8410625	1,90E-05
Trna methyltransferase 10C	*Trmt10c*	-1,8408504	0,00080342
Heterogeneous nuclear ribonucleoprotein R	*Hnrnpr*	-1,8404329	0,00012582
Solute carrier family 7 (cationic amino acid transporter, y+ system), member 6	*Slc7a6*	-1,8368218	0,00028487
NIMA (never in mitosis gene a)-related expressed kinase 6	*Nek6*	-1,8358348	2,89E-05
Atlastin gtpase 3	*Atl3*	-1,8346097	0,00019664
Zinc finger protein 410	*Zfp410*	-1,8331035	1,29E-05
Protein tyrosine phosphatase, non-receptor type 22 (lymphoid)	*Ptpn22*	-1,8327064	0,0004772
SWI5 dependent recombination repair 1	*Sfr1*	-1,8324736	8,98E-05
Protein phosphatase 1, regulatory (inhibitor) subunit 14B	*Ppp1r14b*	-1,8319272	5,19E-06
WW, C2 and coiled-coil domain containing 2	*Wwc2*	-1,8307237	0,00018972
Peptidyl-trna hydrolase 2	*Ptrh2*	-1,830629	8,23E-06
HAUS augmin-like complex, subunit 6	*Haus6*	-1,830333	0,00026177

Ribosomal protein L31 /// ribosomal protein L31, pseudogene 1 2	*Rpl31 /// Rpl31-ps12*	-1,8300662	5,23E-05
Dnaj (Hsp40) homolog, subfamily A, member 2	*Dnaja2*	-1,8300209	0,00060813
Solute carrier family 39 (zinc transporter), member 14	*Slc39a14*	-1,8294374	1,55E-05
Mucolipin 1	*Mcoln1*	-1,8293349	1,20E-05
Fatty acyl coa reductase 1	*Far1*	-1,8287807	0,00042552
CD200 antigen	*Cd200*	-1,8279482	0,00355681
Phosphatidylinositol glycan anchor biosynthesis, class N	*Pign*	-1,8274115	0,00010389
Intercellular adhesion molecule 1	*Icam1*	-1,8268078	0,00035293
Translocase of inner mitochondrial membrane 44	*Timm44*	-1,8259004	5,51E-05
RIKEN cdna 2510009E07 gene	*2510009E07Rik*	-1,8247303	3,42E-05
Interleukin 18 receptor accessory protein	*Il18rap*	-1,8245764	6,03E-05
ER membrane protein complex subunit 8	*Emc8*	-1,824479	1,27E-05
Star-related lipid transfer (START) domain containing 5	*Stard5*	-1,8241153	5,55E-05
Zinc finger protein 422	*Zfp422*	-1,822821	0,00085972
Transforming, acidic coiled-coil containing protein 2	*Tacc2*	-1,8226144	1,35E-05
Adducin 3 (gamma)	*Add3*	-1,821377	3,13E-05
Sterile alpha motif and leucine zipper containing kinase AZK	*Zak*	-1,8213614	3,70E-05
Transducin-like enhancer of split 4, homolog of Drosophila E(spl)	*Tle4*	-1,8208163	9,93E-05
UDP-glcnac:betagal beta-1,3-N-acetylglucosaminyltransferase 1	*B3gnt1*	-1,8206219	0,00010863
Amyloid beta (A4) precursor protein	*App*	-1,8193812	1,39E-05
Mediator complex subunit 1	*Med1*	-1,8177513	0,00039331
Sjogren's syndrome/scleroderma autoantigen 1 homolog (human)	*Sssca1*	-1,8172249	3,38E-05
Angiomotin	*Amot*	-1,8170641	0,00022899
Inhibitor of kappab kinase beta	*Ikbkb*	-1,81581	2,78E-05
HIG1 domain family, member 2A	*Higd2a*	-1,815404	4,26E-05
Family with sequence similarity 32, member A	*Fam32a*	-1,8149988	1,92E-05
Placental growth factor	*Pgf*	-1,8147131	0,00061047
Thrombospondin, type I, domain 1	*Thsd1*	-1,8146347	0,00064027
Insulin degrading enzyme	*Ide*	-1,8105283	1,47E-05
Carbohydrate (N-acetylgalactosamine 4-0) sulfotransferase 14	*Chst14*	-1,8104507	7,60E-06
Angiopoietin-like 4	*Angptl4*	-1,8093563	0,00122369
Prokineticin receptor 1	*Prokr1*	-1,808593	7,50E-05
Sideroflexin 2	*Sfxn2*	-1,8085383	0,00013449
Fumarylacetoacetate hydrolase domain containing 1	*Fahd1*	-1,8070627	2,63E-05
Transmembrane 9 superfamily member 3	*Tm9sf3*	-1,8066763	0,00129859
RIKEN cdna 4932431P20 gene /// predicted gene, 21596 /// predicted gene 6115 /// high mobility group box 1	*4932431P20Rik /// Gm21596 /// Gm6115 /// Hmgb1*	-1,8064171	0,00245043
Interferon gamma receptor 2	*Ifngr2*	-1,8059897	2,89E-05
Adhesion molecule with Ig like domain 1	*Amigo1*	-1,8039335	0,00019495
Stromal cell derived factor 4	*Sdf4*	-1,803504	1,84E-05

Testis expressed gene 10	*Tex10*	-1,8014605	4,97E-06
Atpase, class VI, type 11A	*Atp11a*	-1,80026	1,42E-05
Cell division cycle associated 7	*Cdca7*	-1,7989106	0,00011531
Seven in absentia 2	*Siah2*	-1,7977982	1,47E-05
Max interacting protein 1	*Mxi1*	-1,7974838	1,70E-05
Transmembrane protein with EGF-like and two follistatin-like domains 1	*Tmeff1*	-1,7973388	0,0020038
Rhomboid, veinlet-like 3 (Drosophila)	*Rhbdl3*	-1,7963984	0,00016694
Receptor-associated protein of the synapse	*Rapsn*	-1,7962584	3,15E-05
F-box and WD-40 domain protein 4	*Fbxw4*	-1,795061	0,00011134
Secreted phosphoprotein 1	*Spp1*	-1,7950022	2,71E-05
Protein phosphatase 1M	*Ppm1m*	-1,7940256	0,00022991
Jun B proto-oncogene	*Junb*	-1,7934501	0,0008008
Glucuronidase, beta	*Gusb*	-1,7932789	0,00044017
P21 protein (Cdc42/Rac)-activated kinase 3	*Pak3*	-1,7928118	8,28E-05
WD repeat domain 74	*Wdr74*	-1,792057	2,01E-05
CD151 antigen	*Cd151*	-1,7912572	2,19E-05
Atpase, Na+/K+ transporting, alpha 2 polypeptide	*Atp1a2*	-1,7909946	0,00600169
RIKEN cdna 4930453N24 gene	*4930453N24Rik*	-1,7904757	0,00015251
TNF receptor associated factor 4	*Traf4*	-1,7898688	0,00022595
Mitogen-activated protein kinase 8 interacting protein 3	*Mapk8ip3*	-1,7877441	0,00025651
Toll-like receptor 2	*Tlr2*	-1,7876196	6,65E-05
Ladybird homeobox homolog 1 (Drosophila)	*Lbx1*	-1,7864236	0,00048071
Phospholipid transfer protein	*Pltp*	-1,7856687	0,00336225
Protein phosphatase 1, regulatory (inhibitor) subunit 8	*Ppp1r8*	-1,7852943	0,00071084
Zinc finger protein 330	*Zfp330*	-1,7850562	1,14E-05
REST corepressor 1	*Rcor1*	-1,7848784	1,64E-05
TSR1 20S rrna accumulation	*Tsr1*	-1,7841973	1,66E-05
Flap structure specific endonuclease 1	*Fen1*	-1,783593	2,66E-05
Guanine nucleotide binding protein-like 2 (nucleolar)	*Gnl2*	-1,7827391	0,00037893
Arfgap with rhogap domain, ankyrin repeat and PH domain 2	*Arap2*	-1,7816706	0,00015049
WD repeat domain containing 83	*Wdr83*	-1,7809481	0,0001442
Peroxiredoxin 5	*Prdx5*	-1,7803333	1,92E-05
Lymphotoxin B receptor	*Ltbr*	-1,7803093	0,00013327
Tetraspanin 12	*Tspan12*	-1,7794998	0,00026891
THAP domain containing 11	*Thap11*	-1,7789917	1,23E-05
Mediator complex subunit 8	*Med8*	-1,7782972	1,84E-05
ATM interactor	*Atmin*	-1,7777315	5,35E-05
Runt related transcription factor 1	*Runx1*	-1,7771212	6,79E-05
Tumor necrosis factor receptor superfamily, member 22	*Tnfrsf22*	-1,7751567	0,00369812
Oxysterol binding protein-like 11	*Osbpl11*	-1,7751558	0,00014418
High mobility group box transcription factor 1	*Hbp1*	-1,7751156	6,06E-05
Legumain	*Lgmn*	-1,7746697	4,33E-05

Fumarylacetoacetate hydrolase	*Fah*	-1,7741737	3,02E-05
Sperm flagellar 1	*Spef1*	-1,7741123	1,44E-05
Solute carrier family 7 (cationic amino acid transporter, y+ system), member 2	*Slc7a2*	-1,7724662	0,00079689
Eukaryotic translation initiation factor 4, gamma 1	*Eif4g1*	-1,7719065	0,00505066
Peroxiredoxin 2	*Prdx2*	-1,7716232	1,79E-05
Mitochondrial ribosomal protein L11	*Mrpl11*	-1,7711587	3,14E-05
SUMO/sentrin specific peptidase 6	*Senp6*	-1,7709077	0,00010713
Serine/arginine-rich splicing factor 3	*Srsf3*	-1,7705228	0,00300055
PHD finger protein 3	*Phf3*	-1,7691424	0,00724753
CLOCK interacting protein, circadian	*Cipc*	-1,7685465	1,05E-05
UDP-Gal:betagal beta 1,3-galactosyltransferase, polypeptide 6	*B3galt6*	-1,7681073	3,49E-05
Zinc finger, CCHC domain containing 14	*Zcchc14*	-1,768107	4,61E-05
Forkhead box P1	*Foxp1*	-1,7663387	2,65E-05
Arylsulfatase A	*Arsa*	-1,7660407	1,40E-05
Transmembrane protein 218	*Tmem218*	-1,765829	0,00036196
RIKEN cdna 1810009A15 gene /// uncharacterized LOC102308570	*1810009A15Rik /// LOC102308570*	-1,7658277	1,55E-05
M phase phosphoprotein 6	*Mphosph6*	-1,7656621	2,59E-05
Mutl homolog 3 (E coli)	*Mlh3*	-1,7656113	9,29E-05
Atpase, H+ transporting, lysosomal V1 subunit F	*Atp6v1f*	-1,7646158	2,10E-05
Zinc finger and BTB domain containing 8a	*Zbtb8a*	-1,7638086	0,00035912
Microrchidia 3	*Morc3*	-1,7636804	0,00144222
Dihydrolipoamide S-succinyltransferase (E2 component of 2-oxo-glutarate complex)	*Dlst*	-1,7632964	1,52E-05
--- /// RAB26, member RAS oncogene family, opposite strand	*2610019E17Rik /// Rab26os*	-1,7631063	0,00403316
Family with sequence similarity 178, member A	*Fam178a*	-1,7630166	0,00043287
Alcohol dehydrogenase, iron containing, 1	*Adhfe1*	-1,7584427	0,00034882
1-acylglycerol-3-phosphate O-acyltransferase 6 (lysophosphatidic acid acyltransferase, zeta)	*Agpat6*	-1,7579763	0,00061757
Eukaryotic translation elongation factor 1 delta (guanine nucleotide exchange protein)	*Eef1d*	-1,7578941	1,27E-05
Non-catalytic region of tyrosine kinase adaptor protein 1	*Nck1*	-1,7577384	0,00029038
Rho/rac guanine nucleotide exchange factor (GEF) 18	*Arhgef18*	-1,7575874	9,61E-05
Y box protein 3	*Ybx3*	-1,7562527	4,83E-05
Sirtuin 1	*Sirt1*	-1,7553491	0,00014513
Vacuole membrane protein 1	*Vmp1*	-1,7544198	4,70E-06
Coiled-coil domain containing 84	*Ccdc84*	-1,7534478	0,00036145
Serine/arginine-rich protein specific kinase 1	*Srpk1*	-1,75154	2,80E-05
Exostoses (multiple) 1	*Ext1*	-1,7514839	1,40E-05
Family with sequence similarity 192, member A	*Fam192a*	-1,7514241	1,60E-05
E74-like factor 2	*Elf2*	-1,7512071	0,00383655
Tryptophanyl-trna synthetase	*Wars*	-1,7510321	0,00015138
Telomeric repeat binding factor 2	*Terf2*	-1,7495151	5,09E-05
Heparan-alpha-glucosaminide N-acetyltransferase	*Hgsnat*	-1,7494355	3,47E-05

Predicted gene 10349 /// predicted pseudogene 9386 /// nuclear transport factor 2 /// nuclear transport factor 2, pseudogene 1	*Gm10349 /// Gm9386 /// Nutf2 /// Nutf2-ps1*	-1,7477325	2,22E-05
NOP56 ribonucleoprotein	*Nop56*	-1,7475036	1,82E-05
Interleukin 11	*Il11*	-1,7465893	0,00011771
Fibroblast growth factor (acidic) intracellular binding protein	*Fibp*	-1,7452654	4,58E-05
Prefoldin subunit 6	*Pfdn6*	-1,7449728	6,75E-05
Mucolipin 3	*Mcoln3*	-1,7442108	0,00032787
Histone cluster 1, h1c	*Hist1h1c*	-1,7436993	0,00299673
Ring finger protein 128	*Rnf128*	-1,7435021	0,00012702
Keratin 80	*Krt80*	-1,7433043	0,00032559
Cdna sequence BC003965	*BC003965*	-1,7431366	2,02E-05
Zinc finger protein 960 /// zinc finger protein 97	*Zfp960 /// Zfp97*	-1,7424854	0,00028449
Rho family gtpase 3	*Rnd3*	-1,7413087	6,97E-05
Collagen, type XII, alpha 1	*Col12a1*	-1,7383292	0,0023225
Gamma-glutamyl hydrolase	*Ggh*	-1,7377086	0,00095496
Sphingomyelin phosphodiesterase, acid-like 3A	*Smpdl3a*	-1,7376647	6,60E-05
Phosphatidylinositol 4-kinase type 2 alpha	*Pi4k2a*	-1,7354053	2,81E-05
Cytochrome c oxidase assembly protein 15	*Cox15*	-1,7346717	2,78E-05
HECT domain containing 1	*Hectd1*	-1,7340627	8,30E-05
Fatty acid desaturase 2	*Fads2*	-1,732399	1,61E-05
NTF2-related export protein 1	*Nxt1*	-1,7317228	1,34E-05
Metal response element binding transcription factor 2	*Mtf2*	-1,7312448	0,00383471
Ribosomal protein S6 kinase polypeptide 3	*Rps6ka3*	-1,7311745	0,00016389
Complement component 1, q subcomponent binding protein	*C1qbp*	-1,7302609	6,78E-06
Ubiquitin specific peptidase 11	*Usp11*	-1,7299344	0,00050492
Zinc finger protein 617	*Zfp617*	-1,7298008	2,39E-05
Solute carrier family 7 (cationic amino acid transporter, y+ system), member 11	*Slc7a11*	-1,7296196	0,00597696
Zinc finger protein 445	*Zfp445*	-1,7294833	0,00024753
UHRF1 (ICBP90) binding protein 1	*Uhrf1bp1*	-1,7288239	5,49E-05
POU domain, class 6, transcription factor 1	*Pou6f1*	-1,7282853	3,15E-05
Immediate early response 3	*Ier3*	-1,7273387	1,88E-05
Sel-1 suppressor of lin-12-like (C. Elegans)	*Sel1l*	-1,7271652	0,0001558
Target of EGR1, member 1 (nuclear)	*Toe1*	-1,727084	4,92E-05
CAP-GLY domain containing linker protein 1	*Clip1*	-1,726622	0,00012987
UTP11-like, U3 small nucleolar ribonucleoprotein, (yeast)	*Utp11l*	-1,7260043	0,0003272
Solute carrier family 39 (zinc transporter), member 7	*Slc39a7*	-1,7255014	1,06E-05
Solute carrier family 25, member 36	*Slc25a36*	-1,723691	0,00017372
Scavenger receptor class B, member 2	*Scarb2*	-1,7225509	2,17E-05
Myod family inhibitor domain containing	*Mdfic*	-1,7222644	0,0002504
RIKEN cdna 4632428N05 gene	*4632428N05Rik*	-1,7199632	0,00030148
Tropomodulin 3	*Tmod3*	-1,7195654	0,00144625

Receptor (TNFRSF)-interacting serine-threonine kinase 2	*Ripk2*	-1,7190745	4,77E-05
Nudix (nucleoside diphosphate linked moiety X)-type motif 14	*Nudt14*	-1,7180218	0,00068783
SPRY domain containing 7	*Spryd7*	-1,7178057	0,00184696
Methylenetetrahydrofolate dehydrogenase (NAD+ dependent), methenyltetrahydrofolate cyclohydrolase	*Mthfd2*	-1,7176719	4,14E-05
Centrosomal protein 68	*Cep68*	-1,7172285	1,69E-05
Phospholipid scramblase 1	*Plscr1*	-1,7155966	0,00096642
Peroxisomal biogenesis factor 6	*Pex6*	-1,7152908	6,32E-05
Prolactin family 6, subfamily a, member 1	*Prl6a1*	-1,7149968	0,00091113
Peroxisome proliferative activated receptor, gamma, coactivator-related 1	*Pprc1*	-1,7147696	2,40E-05
Anoctamin 10	*Ano10*	-1,7120055	2,86E-05
Integrin beta 6	*Itgb6*	-1,7117932	0,00072941
Ventral anterior homeobox 2	*Vax2*	-1,7116794	0,00018518
Carnitine palmitoyltransferase 2	*Cpt2*	-1,7114927	2,57E-05
TSR3 20S rrna accumulation	*Tsr3*	-1,7114158	0,00010376
OTU domain, ubiquitin aldehyde binding 2	*Otub2*	-1,7112972	0,00025423
Family with sequence similarity 118, member B	*Fam118b*	-1,7111051	0,00062093
Dual-specificity tyrosine-(Y)-phosphorylation regulated kinase 3	*Dyrk3*	-1,7107664	8,40E-05
RB1-inducible coiled-coil 1	*Rb1cc1*	-1,7105768	1,65E-05
Spindlin 1	*Spin1*	-1,7099823	8,88E-05
RIO kinase 2 (yeast)	*Riok2*	-1,7097521	1,40E-05
Cytochrome c oxidase subunit viiia	*Cox8a*	-1,7091714	5,46E-05
PDLIM1 interacting kinase 1 like	*Pdik1l*	-1,7090587	0,00160228
Fc receptor, ige, low affinity II, alpha polypeptide	*Fcer2a*	-1,7079448	0,00123895
Tenascin C	*Tnc*	-1,7073908	0,00186058
Solute carrier family 4, sodium bicarbonate cotransporter, member 7	*Slc4a7*	-1,7065006	0,00393314
High mobility group nucleosomal binding domain 1	*Hmgn1*	-1,7061968	1,05E-05
Transporter 1, ATP-binding cassette, sub-family B (MDR/TAP)	*Tap1*	-1,70599	0,00018166
Splicing factor 3b, subunit 2	*Sf3b2*	-1,7055168	5,56E-05
Kelch domain containing 3	*Klhdc3*	-1,7053407	0,00039514
Forkhead box Q1	*Foxq1*	-1,7041959	0,00619057
Arginine and glutamate rich 1	*Arglu1*	-1,7040414	0,00128754
Chitobiase, di-N-acetyl-	*Ctbs*	-1,7030775	0,00326176
Neuraminidase 1	*Neu1*	-1,7024114	0,00061396
GA repeat binding protein, alpha	*Gabpa*	-1,7020113	0,00096471
IAP promoted placental gene	*Ipp*	-1,701566	0,00161331
Transmembrane protein 179B	*Tmem179b*	-1,7014444	9,89E-05
Atpase, H+ transporting, lysosomal V1 subunit B2	*Atp6v1b2*	-1,700442	5,88E-05
Cytotoxic T lymphocyte-associated protein 2 beta	*Ctla2b*	-1,6999924	9,68E-06
Nuclear assembly factor 1 homolog (S. Cerevisiae)	*Naf1*	-1,6988905	0,00017755
SDA1 domain containing 1	*Sdad1*	-1,6981569	0,00082526

Coordinator of PRMT5, differentiation stimulator	*Coprs*	-1,6976787	2,29E-05
Lysosomal-associated membrane protein 3 /// peptidylprolyl isomerase D (cyclophilin D)	*Lamp3 /// Ppid*	-1,6975751	3,59E-05
Seven in absentia 1A /// seven in absentia 1B	*Siah1a /// Siah1b*	-1,6970483	0,00140315
Finkel-Biskis-Reilly murine sarcoma virus (FBR-musv) ubiquitously expressed (fox derived)	*Fau*	-1,6961939	2,00E-05
Translocase of outer mitochondrial membrane 20 homolog (yeast)	*Tomm20*	-1,6961271	0,00016344
Paraoxonase 2	*Pon2*	-1,6951933	0,00993476
Protein kinase C substrate 80K-H	*Prkcsh*	-1,6947975	4,08E-05
Iroquois related homeobox 5 (Drosophila)	*Irx5*	-1,6947744	0,000136
Solute carrier family 29 (nucleoside transporters), member 2	*Slc29a2*	-1,6944869	0,00032385
Butyrophilin, subfamily 1, member A1	*Btn1a1*	-1,6942355	0,00105503
CDK2-associated protein 2	*Cdk2ap2*	-1,6939759	3,59E-05
Nudix (nucleoside diphosphate linked moiety X)-type motif 22	*Nudt22*	-1,6929635	0,00039465
Cell division cycle 37-like 1	*Cdc37l1*	-1,6915591	0,00063215
Dnaj (Hsp40) homolog, subfamily C, member 27	*Dnajc27*	-1,6914915	0,00136812
Superoxide dismutase 2, mitochondrial	*Sod2*	-1,6909995	8,21E-06
Zinc finger, DHHC domain containing 12	*Zdhhc12*	-1,6890051	5,47E-05
Laminin, beta 2	*Lamb2*	-1,6886079	0,00019565
Carbohydrate (N-acetylgalactosamine 4-sulfate 6-O) sulfotransferase 15	*Chst15*	-1,6879774	0,00264945
Patched homolog 1	*Ptch1*	-1,6872684	0,00026611
Ly6/neurotoxin 1	*Lynx1*	-1,6866635	0,00011364
Nuclear factor, erythroid derived 2, like 2	*Nfe2l2*	-1,6862028	0,0018454
3-phosphoglycerate dehydrogenase pseudogene /// 3-phosphoglycerate dehydrogenase	*Gm6756 /// Phgdh*	-1,6856667	1,09E-05
Chaperonin containing Tcp1, subunit 8 (theta)	*Cct8*	-1,6855584	6,94E-05
Transmembrane protein 147	*Tmem147*	-1,6852609	2,94E-05
Leucine rich repeat containing 42	*Lrrc42*	-1,6847988	0,00053213
Kruppel-like factor 5	*Klf5*	-1,6847378	0,00040939
Growth arrest and DNA-damage-inducible, gamma interacting protein 1	*Gadd45gip1*	-1,684676	0,00018617
Xylosylprotein beta1,4-galactosyltransferase, polypeptide 7 (galactosyltransferase I)	*B4galt7*	-1,6836628	0,00027249
Keratin 10	*Krt10*	-1,6826202	0,00037366
5-methyltetrahydrofolate-homocysteine methyltransferase reductase	*Mtrr*	-1,6825436	3,04E-05
Structural maintenance of chromosomes 5	*Smc5*	-1,6819125	6,80E-05
Deoxynucleotidyltransferase, terminal, interacting protein 2	*Dnttip2*	-1,6812704	5,56E-05
SRY (sex determining region Y)-box 4	*Sox4*	-1,6809269	9,09E-05
Protein tyrosine phosphatase, non-receptor type 21	*Ptpn21*	-1,6789133	0,00077436
Growth hormone receptor	*Ghr*	-1,6780595	0,00010548
Tetratricopeptide repeat domain 3	*Ttc3*	-1,6762934	0,00069066
Serum amyloid A 1	*Saa1*	-1,6753299	0,00023652
Zinc finger protein 868	*Zfp868*	-1,6740142	0,00021008

Jun proto-oncogene	*Jun*	-1,6739944	0,0001581
Mitochondrial ribosomal protein S18B	*Mrps18b*	-1,6731052	3,77E-05
Ariadne homolog 2 (Drosophila)	*Arih2*	-1,6723043	1,68E-05
Nuclear factor of activated T cells 5	*Nfat5*	-1,6715933	0,00019307
Predicted gene 561	*Gm561*	-1,6711139	9,36E-05
Nucleoporin 50	*Nup50*	-1,6703428	0,00015566
Galactosidase, alpha	*Gla*	-1,6700646	1,70E-05
Autism susceptibility candidate 2	*Auts2*	-1,6692109	0,00350407
Zinc finger and BTB domain containing 22	*Zbtb22*	-1,6681093	2,98E-05
N-acetylneuraminic acid phosphatase	*Nanp*	-1,6678696	9,26E-05
Predicted gene 5633 /// predicted gene 9178 /// MRT4, mrna turnover 4, homolog (S. Cerevisiae)	*Gm5633 /// Gm9178 /// Mrto4*	-1,6677536	0,00012849
Zinc finger, HIT domain containing 2	*Znhit2*	-1,6666869	0,00021062
Oxidative stress responsive serine rich 1	*Oser1*	-1,6664609	2,45E-05
Zinc finger, AN1-type domain 2A	*Zfand2a*	-1,666269	5,43E-05
Transferrin receptor	*Tfrc*	-1,6660503	9,37E-05
Yes-associated protein 1	*Yap1*	-1,6659897	4,60E-05
Chemokine (C-X-C motif) ligand 10	*Cxcl10*	-1,6652132	0,00316737
Checkpoint kinase 1	*Chek1*	-1,6649808	0,00014204
Succinate dehydrogenase complex assembly factor 1	*Sdhaf1*	-1,6648324	3,77E-05
Embigin	*Emb*	-1,6634047	0,00219293
Eukaryotic translation initiation factor 2B, subunit 2 beta	*Eif2b2*	-1,6630545	0,00016812
Nuclear transcription factor, X-box binding-like 1	*Nfxl1*	-1,6628527	0,00287331
Bystin-like	*Bysl*	-1,6622731	0,00065736
Zinc finger protein 189	*Zfp189*	-1,6621048	0,00054019
Growth factor independent 1	*Gfi1*	-1,6612297	0,0001374
Kelch-like 26	*Klhl26*	-1,6608848	7,89E-05
CCR4-NOT transcription complex, subunit 1 /// predicted gene 6158	*Cnot1 /// Gm6158*	-1,660796	6,96E-05
Insulin receptor substrate 2	*Irs2*	-1,6603707	0,00052511
Interferon regulatory factor 2	*Irf2*	-1,6602652	0,00062565
Intersectin 2	*Itsn2*	-1,6596865	0,00285018
Serine hydroxymethyltransferase 2 (mitochondrial)	*Shmt2*	-1,6596211	0,00028676
T-box 15	*Tbx15*	-1,6595053	0,00912172
Upstream binding protein 1	*Ubp1*	-1,659428	0,00013836
Zinc finger protein 790	*Zfp790*	-1,6592074	0,00182811
Bleomycin hydrolase	*Blmh*	-1,6590463	5,33E-05
Yrdc domain containing (E.coli)	*Yrdc*	-1,6584285	0,00012841
PAP associated domain containing 4	*Papd4*	-1,6580877	0,00403509
Iron-sulfur cluster assembly 2 homolog (S. Cerevisiae)	*Isca2*	-1,6579463	5,33E-05
Ectonucleoside triphosphate diphosphohydrolase 7	*Entpd7*	-1,6577865	0,00324034
Discoidin, CUB and LCCL domain containing 2	*Dcbld2*	-1,657377	0,00120822
Sal-like 2 (Drosophila)	*Sall2*	-1,6570123	0,00040897
Protein tyrosine phosphatase 4a2	*Ptp4a2*	-1,6568537	0,0063532
Integrin beta 5	*Itgb5*	-1,6566354	0,00013564

Guanine nucleotide binding protein-like 3 (nucleolar)	*Gnl3*	-1,6562636	2,55E-05
Potassium channel tetramerisation domain containing 20	*Kctd20*	-1,6559291	0,00018256
Homeodomain interacting protein kinase 2	*Hipk2*	-1,6555474	4,79E-05
Lysocardiolipin acyltransferase 1	*Lclat1*	-1,6544211	4,12E-05
Zinc finger protein 51	*Zfp51*	-1,6542492	0,00479695
Lysm, putative peptidoglycan-binding, domain containing 2	*Lysmd2*	-1,6539949	0,00087848
Pescadillo homolog 1, containing BRCT domain (zebrafish)	*Pes1*	-1,653884	0,00031889
TAR DNA binding protein	*Tardbp*	-1,651699	0,00082195
Vasodilator-stimulated phosphoprotein	*Vasp*	-1,6506392	0,0001279
Cyclin-dependent kinase 11B	*Cdk11b*	-1,6504804	0,0003188
Collagen, type XVII, alpha 1	*Col17a1*	-1,650238	0,00374561
F-box and leucine-rich repeat protein 8	*Fbxl8*	-1,6494071	0,00215747
Zinc finger protein 68	*Zfp68*	-1,6491436	0,00039514
Atpase family, AAA domain containing 2B	*Atad2b*	-1,6490602	0,00013797
Terminal uridylyl transferase 1, U6 snrna-specific	*Tut1*	-1,648682	2,49E-05
Cullin 4A	*Cul4a*	-1,6484864	9,34E-05
RIKEN cdna 9130004C02 gene	*9130004C02Rik*	-1,6484848	0,00705679
RNA binding motif protein 39	*Rbm39*	-1,6483542	0,00012121
RIKEN cdna 1110054M08 gene	*1110054M08Rik*	-1,6481994	0,00664812
NADH dehydrogenase (ubiquinone) Fe-S protein 8	*Ndufs8*	-1,6476346	0,00015787
PRP4 pre-mrna processing factor 4 homolog B (yeast)	*Prpf4b*	-1,6472315	0,00015966
Proviral integration site 1	*Pim1*	-1,6469027	5,17E-05
Septin 2	*IX.02*	-1,6462992	9,66E-05
RIKEN cdna 2410016O06 gene	*2410016O06Rik*	-1,6462761	1,90E-05
Tripartite motif-containing 13	*Trim13*	-1,643792	0,00626451
General transcription factor II E, polypeptide 2 (beta subunit)	*Gtf2e2*	-1,6437868	0,00024553
Coiled-coil-helix-coiled-coil-helix domain containing 4	*Chchd4*	-1,6430659	0,00028754
Shugoshin-like 1 (S. Pombe)	*Sgol1*	-1,6429031	6,36E-05
Echinoderm microtubule associated protein like 5	*Eml5*	-1,6425798	0,00030249
MAU2 chromatid cohesion factor homolog (C. Elegans)	*Mau2*	-1,6423467	0,00025423
Rho gtpase activating protein 20	*Arhgap20*	-1,6423103	0,0027511
WW domain binding protein 1 like	*Wbp1l*	-1,6421543	6,79E-05
ATP synthase, H+ transporting, mitochondrial F1 complex, O subunit	*Atp5o*	-1,6418682	0,0006032
Receptor-like tyrosine kinase	*Ryk*	-1,6405796	1,51E-05
Guanine nucleotide binding protein (G protein), beta polypeptide 1-like	*Gnb1l*	-1,6405744	0,00031934
Eukaryotic translation elongation factor 1 gamma /// elongation factor 1-gamma-like	*Eef1g /// LOC101055956*	-1,6392231	1,47E-05
Ring finger protein 26	*Rnf26*	-1,6392044	2,70E-05
Translocase of outer mitochondrial membrane 70 homolog A (yeast)	*Tomm70a*	-1,6386319	0,00185585
HEAT repeat containing 1	*Heatr1*	-1,6382964	0,00770596
Cdna sequence BC027231	*BC027231*	-1,637374	0,0001302

Expressed sequence AU040320	*AU040320*	-1,6366502	0,00387527
Dual specificity phosphatase 13	*Dusp13*	-1,6365428	0,00017251
Arginine/serine-rich coiled-coil 2	*Rsrc2*	-1,635651	0,00104116
SR-related CTD-associated factor 4	*Scaf4*	-1,6336445	0,00155013
Discoidin, CUB and LCCL domain containing 1	*Dcbld1*	-1,6335513	0,00010735
Atlastin gtpase 2	*Atl2*	-1,6335373	0,00111368
Family with sequence similarity 117, member A	*Fam117a*	-1,6335135	0,00503395
Zinc finger, AN1-type domain 3	*Zfand3*	-1,6318197	0,00013925
Predicted gene 3776 /// glutathione S-transferase, alpha 1 (Ya) /// glutathione S-transferase, alpha 2 (Yc2)	*Gm3776 /// Gsta1 /// Gsta2*	-1,6312329	0,00084792
WD repeat domain 77	*Wdr77*	-1,6311202	0,00133308
Bromodomain containing 4	*Brd4*	-1,6304191	0,00350277
Intraflagellar transport 81	*Ift81*	-1,6299311	8,56E-05
SWI/SNF related, matrix associated, actin dependent regulator of chromatin, subfamily d, member 3	*Smarcd3*	-1,6297353	0,00036251
Expressed sequence AI503316 /// heterogeneous nuclear ribonucleoprotein U	*AI503316 /// Hnrnpu*	-1,6292074	0,00084056
Fasciculation and elongation protein zeta 1 (zygin I)	*Fez1*	-1,6290314	0,00159037
TATA box binding protein (Tbp)-associated factor, RNA polymerase I, C	*Taf1c*	-1,6288227	7,34E-05
Heat shock protein 1	*Hspb1*	-1,6286607	0,0015719
Zinc finger protein 292	*Zfp292*	-1,6281707	0,00022006
Ribosomal protein S9	*Rps9*	-1,6279827	0,00253964
RIKEN cdna 2810474O19 gene	*2810474O19Rik*	-1,6275299	0,00201029
Histone cluster 1, H3b /// histone cluster 1, H3c /// histone cluster 1, H3d /// histone cluster 1, H3e /// histone cluster 1, H3f /// histone cluster 2, H3b /// histone cluster 2, H3c1 /// histone cluster 2, H3c2	*Hist1h3b /// Hist1h3c /// Hist1h3d /// Hist1h3e /// Hist1h3f /// Hist2h3b /// Hist2h3c1 /// Hist2h3c2*	-1,626695	0,00127637
Rho gtpase activating protein 8	*Arhgap8*	-1,6266404	6,29E-05
Ring finger protein 113A1	*Rnf113a1*	-1,6252598	9,60E-05
Chloride channel, nucleotide-sensitive, 1A	*Clns1a*	-1,6251458	8,88E-05
Zinc finger protein 110	*Zfp110*	-1,6230165	0,0005911
Fibroblast growth factor 16	*Fgf16*	-1,6225068	0,00035781
Phosphatase, orphan 2	*Phospho2*	-1,6224271	0,00235015
Zinc finger protein 869	*Zfp869*	-1,6223945	0,00146425
DTW domain containing 1	*Dtwd1*	-1,621991	0,00038586
Lysosomal-associated membrane protein 2	*Lamp2*	-1,6218468	1,77E-05
RIKEN cdna D230025D16 gene	*D230025D16Rik*	-1,6217961	0,00020384
RIKEN cdna 1200015M12 gene /// RIKEN cdna A130040M12 gene	*1200015M12Rik /// A130040M12Rik*	-1,6210431	1,72E-05
Ribosomal L1 domain containing 1	*Rsl1d1*	-1,6205671	8,85E-05
Zinc finger protein 644	*Zfp644*	-1,6201798	0,00105247
Mesoderm development candidate 2	*Mesdc2*	-1,6200563	2,03E-05
GATA zinc finger domain containing 2A	*Gatad2a*	-1,6197474	0,00031785

Coiled-coil domain containing 50	*Ccdc50*	-1,6187775	0,00784987
GRAM domain containing 1A	*Gramd1a*	-1,6185009	0,00015574
Peroxisomal biogenesis factor 11 alpha	*Pex11a*	-1,6172594	0,00119403
Apolipoprotein D	*Apod*	-1,6166171	0,00244809
Purinergic receptor P2X, ligand-gated ion channel 4	*P2rx4*	-1,6160472	0,00011537
ELOVL family member 7, elongation of long chain fatty acids (yeast)	*Elovl7*	-1,6158231	0,00513337
Interleukin 17D	*Il17d*	-1,6153522	0,00021113
Thymosin, beta 10	*Tmsb10*	-1,6149752	0,00022581
Cdna sequence BC002059	*BC002059*	-1,6144678	0,00036991
Pelota homolog (Drosophila)	*Pelo*	-1,6135246	0,00077938
Synaptonemal complex central element protein 2	*Syce2*	-1,6133699	7,23E-05
Synaptotagmin XVII	*Syt17*	-1,6130957	0,00012009
Heterogeneous nuclear ribonucleoprotein U-like 2	*Hnrnpul2*	-1,6129811	2,22E-05
Nucleolin	*Ncl*	-1,6127657	5,46E-05
CCCTC-binding factor	*Ctcf*	-1,612256	0,0001105
Phospholipase A1 member A	*Pla1a*	-1,6121011	6,02E-05
LPS-induced TN factor	*Litaf*	-1,61145	9,22E-05
Leprecan-like 4	*Leprel4*	-1,6110822	0,00012759
Cardiotrophin-like cytokine factor 1	*Clcf1*	-1,6110601	0,00117959
Protein kinase domain containing, cytoplasmic	*Pkdcc*	-1,6107502	0,00324651
NOP58 ribonucleoprotein	*Nop58*	-1,6107403	0,00012551
Heterogeneous nuclear ribonucleoprotein A2/B1	*Hnrnpa2b1*	-1,6107302	9,67E-05
Nrde-2 necessary for RNA interference, domain containing	*Nrde2*	-1,6088905	6,10E-05
Ribosomal protein S6 kinase polypeptide 1	*Rps6ka1*	-1,6083645	0,00277542
RIKEN cdna 2310039H08 gene	*2310039H08Rik*	-1,6077698	0,00037166
Protein phosphatase 1, catalytic subunit, beta isoform	*Ppp1cb*	-1,6071978	0,00411528
Pseudouridine synthase 1	*Pus1*	-1,6064225	7,12E-05
Proteasome (prosome, macropain) 26S subunit, atpase, 4	*Psmc4*	-1,6063524	0,00038058
DBF4 homolog (S. Cerevisiae)	*Dbf4*	-1,604072	0,00018042
Potassium channel tetramerisation domain containing 5	*Kctd5*	-1,6034164	6,40E-05
DCN1, defective in cullin neddylation 1, domain containing 5 (S. Cerevisiae)	*Dcun1d5*	-1,6030109	0,00038754
Erythrocyte protein band 4.1-like 3	*Epb4.1l3*	-1,6028884	7,24E-05
Regulator of G-protein signaling 4	*Rgs4*	-1,6028263	0,00014946
Transcriptional regulator, SIN3B (yeast)	*Sin3b*	-1,6025148	0,00020361
Sin3 associated polypeptide	*Sap30*	-1,6024881	0,00016582
Ubiquitination factor E4A	*Ube4a*	-1,6016554	0,00213282
SWI/SNF-related, matrix-associated actin-dependent regulator of chromatin, subfamily a, containing DEAD/H box 1	*Smarcad1*	-1,6016258	0,00025804
Endothelin converting enzyme 2	*Ece2*	-1,6014467	5,98E-05
NOP2 nucleolar protein	*Nop2*	-1,6004698	0,0002728
Caspase 8 associated protein 2	*Casp8ap2*	-1,600105	0,00091433

A disintegrin-like and metallopeptidase (reprolysin type) with thrombospondin type 1 motif, 7	*Adamts7*	-1,6000736	0,00061957
Lysine rich nucleolar protein 1	*Knop1*	-1,5998158	4,63E-05
RIKEN cdna 1600012H06 gene	*1600012H06Rik*	-1,5996929	0,00019561
Sprouty homolog 1 (Drosophila)	*Spry1*	-1,5989888	0,00015574
Cathepsin A	*Ctsa*	-1,5989635	2,02E-05
RIKEN cdna 2010204K13 gene	*2010204K13Rik*	-1,5984936	0,0010052
Zinc finger protein, multitype 1	*Zfpm1*	-1,59836	0,00112212
Autophagy related 101	*Atg101*	-1,5983171	0,00013925
NEDD8 activating enzyme E1 subunit 1	*Nae1*	-1,5981574	2,05E-05
Protein kinase, cgmp-dependent, type I	*Prkg1*	-1,5978094	0,00414604
Seryl-aminoacyl-trna synthetase	*Sars*	-1,597359	0,00073612
Chitinase domain containing 1	*Chid1*	-1,5972823	0,00157771
Heme oxygenase (decycling) 1	*Hmox1*	-1,5965774	9,04E-05
Methyltransferase like 9	*Mettl9*	-1,5962826	0,00072435
Bone marrow stromal cell antigen 2	*Bst2*	-1,5957651	0,00319001
Family with sequence similarity 76, member B	*Fam76b*	-1,5956001	0,00626604
Rho/rac guanine nucleotide exchange factor (GEF) 2	*Arhgef2*	-1,5939824	0,00040544
Voltage-dependent anion channel 3	*Vdac3*	-1,5937697	2,65E-05
Kelch repeat and BTB (POZ) domain containing 2	*Kbtbd2*	-1,5936355	0,00018925
Dihydroorotate dehydrogenase	*Dhodh*	-1,5935299	0,00029781
ATP-binding cassette, sub-family B (MDR/TAP), member 10	*Abcb10*	-1,5932796	0,000767
GTP binding protein 4	*Gtpbp4*	-1,592716	0,00620963
Xylulokinase homolog (H. Influenzae)	*Xylb*	-1,592428	0,00710783
Solute carrier family 30 (zinc transporter), member 6	*Slc30a6*	-1,5917812	0,00014834
ESF1, nucleolar pre-rrna processing protein, homolog (S. Cerevisiae)	*Esf1*	-1,591155	6,75E-05
ADP-ribosylation factor-like 2 binding protein	*Arl2bp*	-1,5903187	2,56E-05
60S ribosomal protein L23-like /// 60S ribosomal protein L23-like /// ribosomal protein L23	*LOC100044627 /// LOC100862455 /// Rpl23*	-1,5895855	2,95E-05
RE1-silencing transcription factor	*Rest*	-1,5895713	0,00078573
Splicing factor 3b, subunit 3	*Sf3b3*	-1,5887219	0,00024381
Trans-acting transcription factor 3	*Sp3*	-1,5880342	0,00103841
Arsenic (+3 oxidation state) methyltransferase	*As3mt*	-1,5880304	0,00253236
Autocrine motility factor receptor	*Amfr*	-1,58783	0,00013645
Phosphoglycolate phosphatase	*Pgp*	-1,5877012	0,00037959
Cytochrome b5 reductase 1	*Cyb5r1*	-1,5873021	4,14E-05
AT rich interactive domain 4B (RBP1-like)	*Arid4b*	-1,5870294	0,00350918
Small proline-rich protein 2D	*Sprr2d*	-1,586344	0,00121184
Guanine nucleotide binding protein (G protein), gamma 10	*Gng10*	-1,5858999	0,00103461
Solute carrier family 30 (zinc transporter), member 4	*Slc30a4*	-1,5857875	0,00060497
Solute carrier family 31, member 1	*Slc31a1*	-1,5856256	0,0001031
Ring finger protein 2	*Rnf2*	-1,5848022	0,00079475

A disintegrin-like and metallopeptidase (reprolysin type) with thrombospondin type 1 motif, 4	*Adamts4*	-1,5845214	0,00018522
GTP binding protein 6 (putative)	*Gtpbp6*	-1,5840918	5,34E-05
S-adenosylhomocysteine hydrolase	*Ahcy*	-1,5834732	3,68E-05
VPS33B interacting protein, apical-basolateral polarity regulator, spe-39 homolog	*Vipas39*	-1,5823006	0,000182
B cell leukemia/lymphoma 6	*Bcl6*	-1,5822362	7,07E-05
Alpha-N-acetylglucosaminidase (Sanfilippo disease IIIB)	*Naglu*	-1,5820349	0,00057057
Early B cell factor 1	*Ebf1*	-1,5811952	0,00040544
Predicted gene 12942 /// zinc finger, MYM-type 6	*Gm12942 /// Zmym6*	-1,5811314	0,00037774
Matrix metallopeptidase 12	*Mmp12*	-1,5808387	0,00164862
Pyruvate dehydrogenase kinase, isoenzyme 1	*Pdk1*	-1,5804277	0,00022991
Transformer 2 beta homolog (Drosophila)	*Tra2b*	-1,579791	7,29E-05
Transcriptional adaptor 2B	*Tada2b*	-1,579124	0,00033345
Serine/threonine/tyrosine interaction protein	*Styx*	-1,5790565	0,00181844
Trna methyltransferase 11-2	*Trmt112*	-1,5789694	0,00014046
Glycolipid transfer protein domain containing 1	*Gltpd1*	-1,5789003	0,00018926
Cysteine-rich transmembrane module containing 1	*Cystm1*	-1,5775595	0,00069874
Ring finger protein, LIM domain interacting	*Rlim*	-1,5771479	0,00049507
Agrin	*Agrn*	-1,5771475	3,77E-05
MTERF domain containing 1	*Mterfd1*	-1,5769751	8,63E-05
Proline-rich nuclear receptor coactivator 1	*Pnrc1*	-1,576173	0,00022424
Ubiquinol-cytochrome c reductase, complex III subunit X	*Uqcr10*	-1,5758552	0,00016412
DEAD (Asp-Glu-Ala-Asp) box polypeptide 10	*Ddx10*	-1,5748652	0,00379235
Transmembrane protein 38A	*Tmem38a*	-1,5746983	0,00294162
UDP-glucose dehydrogenase	*Ugdh*	-1,5746845	0,00034694
Family with sequence similarity 160, member A2	*Fam160a2*	-1,5746706	0,00330528
UDP-glcnac:betagal beta-1,3-N-acetylglucosaminyltransferase 8	*B3gnt8*	-1,5744857	0,00375727
Arginine vasopressin-induced 1	*Avpi1*	-1,5732164	0,00031138
BCL2-associated athanogene 5	*Bag5*	-1,5731954	0,00014117
Transforming growth factor, beta receptor I	*Tgfbr1*	-1,5730722	0,00168646
Glomulin, FKBP associated protein	*Glmn*	-1,5724264	0,00127535
Proline-rich polypeptide 3	*Prr3*	-1,5717863	0,0016397
Hairy and enhancer of split 1 (Drosophila)	*Hes1*	-1,5715866	0,00016588
Spastin	*Spast*	-1,5715566	0,00010774
Family with sequence similarity 110, member C	*Fam110c*	-1,5708922	0,00338831
Discoidin domain receptor family, member 1	*Ddr1*	-1,5700127	0,00353513
Nuclear transcription factor-Y gamma	*Nfyc*	-1,5696277	3,04E-05
Dynein cytoplasmic 1 light intermediate chain 1	*Dync1li1*	-1,5694898	3,72E-05
Galactosidase, beta 1	*Glb1*	-1,5694741	0,00065602
Armadillo repeat containing, X-linked 3	*Armcx3*	-1,5693181	0,00878164
N(alpha)-acetyltransferase 40, natd catalytic subunit, homolog (S. Cerevisiae)	*Naa40*	-1,5689224	0,00040544

X-prolyl aminopeptidase (aminopeptidase P) 1, soluble	*Xpnpep1*	-1,5682598	0,00016033
Predicted gene 6750 /// predicted pseudogene 7931 /// predicted gene 9525 /// high mobility group nucleosomal binding domain 2	*Gm6750 /// Gm7931 /// Gm9525 /// Hmgn2*	-1,5670234	7,32E-05
Syntaxin 5A	*Stx5a*	-1,5664078	0,0002201
Scl/Tal1 interrupting locus	*Stil*	-1,5658908	0,0015582
Latent transforming growth factor beta binding protein 2	*Ltbp2*	-1,5654794	0,00392732
Zinc finger protein 287	*Zfp287*	-1,5644674	0,00041087
Tripartite motif-containing 25	*Trim25*	-1,5637133	0,00330788
GTP binding protein 2	*Gtpbp2*	-1,5634807	7,62E-05
XPA binding protein 2	*Xab2*	-1,562909	0,00193667
2'-deoxynucleoside 5'-phosphate N-hydrolase 1	*Dnph1*	-1,5628102	5,38E-05
Dishevelled, dsh homolog 1 (Drosophila)	*Dvl1*	-1,5625797	0,00080803
Acetyl-Coenzyme A acetyltransferase 1	*Acat1*	-1,5624396	0,00053755
Trafficking protein particle complex 5	*Trappc5*	-1,5623832	0,00019545
Low density lipoprotein receptor-related protein 2	*Lrp2*	-1,5623639	0,0032811
Serine (or cysteine) peptidase inhibitor, clade B, member 1b	*Serpinb1b*	-1,5620808	0,00109685
Homocysteine-inducible, endoplasmic reticulum stress-inducible, ubiquitin-like domain member 1	*Herpud1*	-1,5619283	0,00051491
Heterogeneous nuclear ribonucleoprotein H1	*Hnrnph1*	-1,5619047	0,00136186
Zinc finger protein 319	*Zfp319*	-1,5615805	9,00E-05
WD repeat domain 45	*Wdr45*	-1,5615231	9,56E-05
CCR4-NOT transcription complex, subunit 7	*Cnot7*	-1,5612462	6,16E-05
DNA segment, Chr 1, ERATO Doi 622, expressed	*D1Ertd622e*	-1,5605946	0,00031974
Cytochrome c oxidase assembly factor 6	*Coa6*	-1,5602601	0,00027664
Thymoma viral proto-oncogene 1 interacting protein	*Aktip*	-1,5601739	0,00017979
Copper chaperone for superoxide dismutase	*Ccs*	-1,5599392	6,92E-05
Natriuretic peptide receptor 2	*Npr2*	-1,5598492	0,0011614
MAP kinase-interacting serine/threonine kinase 1	*Mknk1*	-1,5598158	0,00092676
Solute carrier family 35, member B2	*Slc35b2*	-1,559041	6,60E-05
Insulin-like growth factor 2 mrna binding protein 2	*Igf2bp2*	-1,5586479	6,65E-05
Mitochondrial ribosomal protein S30	*Mrps30*	-1,5584129	0,00011525
Cullin 4B	*Cul4b*	-1,5581903	0,00074507
Cytochrome c oxidase subunit IV isoform 1	*Cox4i1*	-1,5581637	9,41E-05
Interferon regulatory factor 2 binding protein 1	*Irf2bp1*	-1,5580096	0,00115932
Insulin receptor	*Insr*	-1,5560514	0,00651917
SWI/SNF related, matrix associated, actin dependent regulator of chromatin, subfamily a, member 5	*Smarca5*	-1,5557941	0,00022651
Synaptopodin 2	*Synpo2*	-1,5556218	6,22E-05
Excision repair cross-complementing rodent repair deficiency, complementation group 1	*Ercc1*	-1,5554438	0,00015334
TBC1 domain family, member 8	*Tbc1d8*	-1,5554358	0,00161354
MYB binding protein (P160) 1a	*Mybbp1a*	-1,5554303	0,00246176
Ubiquitin specific peptidase 24	*Usp24*	-1,5553654	0,00023055
PC4 and SFRS1 interacting protein 1	*Psip1*	-1,5548455	0,00044193

Carnitine deficiency-associated gene expressed in ventricle 3	*Cdv3*	-1,5543881	0,00863341
Protein disulfide isomerase associated 5	*Pdia5*	-1,554181	0,00050797
Phosphatidylinositol glycan anchor biosynthesis, class T	*Pigt*	-1,5540167	0,00154221
Cytochrome P450, family 2, subfamily c, polypeptide 44	*Cyp2c44*	-1,5539661	0,00027488
Armadillo repeat containing 6	*Armc6*	-1,5535884	0,00061471
Zinc finger protein 282	*Zfp282*	-1,5534526	0,00103669
Protein phosphatase 1B, magnesium dependent, beta isoform	*Ppm1b*	-1,5532801	8,06E-05
Neurolysin (metallopeptidase M3 family)	*Nln*	-1,5529135	0,00018715
Apolipoprotein L 9a /// apolipoprotein L 9b	*Apol9a /// Apol9b*	-1,5528074	0,00112076
Nucleoporin 62	*Nup62*	-1,5527522	9,85E-05
Importin 7	*Ipo7*	-1,5524443	0,00059926
Zinc finger, AN1-type domain 5	*Zfand5*	-1,5519697	0,00029073
Tyrosyl-DNA phosphodiesterase 1	*Tdp1*	-1,5515163	0,00032245
Phosphoprotein associated with glycosphingolipid microdomains 1	*Pag1*	-1,5514846	0,00069949
CTAGE family, member 5	*Ctage5*	-1,551471	0,0005183
RIKEN cdna 1110037F02 gene	*1110037F02Rik*	-1,5513856	9,09E-05
Janus kinase 2	*Jak2*	-1,5511262	8,41E-05
Golgi autoantigen, golgin subfamily a, 7	*Golga7*	-1,5506964	0,00169451
Homeobox C4	*Hoxc4*	-1,5504027	0,00093316
Ras association (ralgds/AF-6) domain family member 1	*Rassf1*	-1,54938	0,00016233
Distal-less homeobox 1	*Dlx1*	-1,5491343	0,00295685
Trna nucleotidyl transferase, CCA-adding, 1	*Trnt1*	-1,5488686	0,00206131
Structural maintenance of chromosomes 3	*Smc3*	-1,5487926	0,00011419
T cell specific gtpase 1 /// T cell specific gtpase 2	*Tgtp1 /// Tgtp2*	-1,5483196	0,00511393
RIKEN cdna 6330509M05 gene /// solute carrier family 9 (sodium/hydrogen exchanger), member 7	*6330509M05Rik /// Slc9a7*	-1,5469798	0,00382254
MIS12 homolog (yeast)	*Mis12*	-1,5468822	0,00017326
SH3-domain binding protein 4	*Sh3bp4*	-1,5463492	0,00025432
Membrane-spanning 4-domains, subfamily A, member 4B	*Ms4a4b*	-1,5460955	0,0016836
Caveolin 1, caveolae protein	*Cav1*	-1,5460451	4,37E-05
Ubiquitin A-52 residue ribosomal protein fusion product 1	*Uba52*	-1,5460369	4,45E-05
Breast cancer anti-estrogen resistance 1	*Bcar1*	-1,5455806	0,00034882
Tripartite motif-containing 30A /// tripartite motif-containing 30D	*Trim30a /// Trim30d*	-1,5444575	0,00160174
Lysophosphatidylglycerol acyltransferase 1	*Lpgat1*	-1,544307	0,0038623
DNA segment, Chr 17, human D6S56E 5	*D17H6S56E-5*	-1,5442883	0,00017517
CTD nuclear envelope phosphatase 1 regulatory subunit 1	*Cnep1r1*	-1,5437151	3,72E-05
Serine/threonine kinase receptor associated protein	*Strap*	-1,5434359	0,00023918
Downstream neighbor of SON	*Donson*	-1,5429832	0,00057512
Zinc finger protein 955B	*Zfp955b*	-1,5426904	0,00304786

Protein-kinase, interferon-inducible double stranded RNA dependent inhibitor, repressor of (P58 repressor)	*Prkrir*	-1,5425866	0,00020066
Proline, glutamic acid and leucine rich protein 1	*Pelp1*	-1,5419286	0,00035375
Protein tyrosine phosphatase, receptor type, O	*Ptpro*	-1,5418063	0,00036139
Required for meiotic nuclear division 5 homolog A (S. Cerevisiae)	*Rmnd5a*	-1,5412556	0,00062039
CKLF-like MARVEL transmembrane domain containing 6	*Cmtm6*	-1,5399817	0,0008353
Ets2 repressor factor	*Erf*	-1,53982	0,0037098
LSM4 homolog, U6 small nuclear RNA associated (S. Cerevisiae)	*Lsm4*	-1,539085	0,0001219
Histocompatibility 2, class II, locus dma	*H2-dma*	-1,5380152	0,00231399
Solute carrier organic anion transporter family, member 3a1	*Slco3a1*	-1,537501	0,00042588
Polyhomeotic-like 2 (Drosophila)	*Phc2*	-1,5368125	0,00037404
Poliovirus receptor	*Pvr*	-1,5366522	9,67E-05
Protein kinase D3	*Prkd3*	-1,5365622	0,00012009
Polymerase (RNA) I polypeptide D	*Polr1d*	-1,5360775	0,00019561
Nuclear factor of activated T cells, cytoplasmic, calcineurin dependent 4	*Nfatc4*	-1,5360014	0,00140729
Plexin domain containing 1	*Plxdc1*	-1,5359795	7,59E-05
Centromere protein N	*Cenpn*	-1,5355461	0,000649
Cleavage and polyadenylation specific factor 3-like	*Cpsf3l*	-1,5355015	0,00010121
SNW domain containing 1	*Snw1*	-1,5352348	0,00017721
Transmembrane protein 53	*Tmem53*	-1,5341039	0,00031523
Small integral membrane protein 12	*Smim12*	-1,5340799	0,00013925
Small nuclear ribonucleoprotein 48 (U11/U12)	*Snrnp48*	-1,5336962	0,00025653
Glycoprotein galactosyltransferase alpha 1, 3	*Ggta1*	-1,5334589	6,22E-05
Ribosomal protein S6 kinase, polypeptide 4	*Rps6ka4*	-1,5327599	0,00123895
Large subunit gtpase 1 homolog (S. Cerevisiae)	*Lsg1*	-1,5315097	0,0006434
Synovial apoptosis inhibitor 1, synoviolin	*Syvn1*	-1,5314108	0,00106949
Fos-like antigen 2	*Fosl2*	-1,5306846	0,00377944
Atpase type 13A1	*Atp13a1*	-1,5305868	0,00036145
GLIS family zinc finger 2	*Glis2*	-1,5304863	9,81E-05
Multivesicular body subunit 12B	*Mvb12b*	-1,5300873	0,0001283
Microtubule-associated protein 1 light chain 3 alpha	*Map1lc3a*	-1,5299054	6,21E-05
Ectonucleotide pyrophosphatase/phosphodiesterase 3	*Enpp3*	-1,5294872	0,0016232
Mckusick-Kaufman syndrome	*Mkks*	-1,5287398	0,00386412
SFT2 domain containing 2	*Sft2d2*	-1,528457	0,00048544
Egl-9 family hypoxia-inducible factor 1	*Egln1*	-1,5276321	5,43E-05
Regulator of chromosome condensation (RCC1) and BTB (POZ) domain containing protein 2	*Rcbtb2*	-1,5270763	0,00942401
KDEL (Lys-Asp-Glu-Leu) containing 1	*Kdelc1*	-1,5268901	8,37E-05
Stress-induced phosphoprotein 1	*Stip1*	-1,5267179	0,0001249
Interleukin 33	*Il33*	-1,5260179	8,50E-05
GINS complex subunit 4 (Sld5 homolog)	*Gins4*	-1,5256982	0,00010221
General transcription factor II H, polypeptide 1	*Gtf2h1*	-1,5253671	0,00015993

NOP14 nucleolar protein	*Nop14*	-1,5252963	0,00034509
Mitochondrial ribosomal protein L37	*Mrpl37*	-1,5250668	4,05E-05
Enoyl Coenzyme A hydratase domain containing 3	*Echdc3*	-1,5240383	0,00262579
SET nuclear oncogene	*Set*	-1,5234237	5,65E-05
Ataxin 7-like 1	*Atxn7l1*	-1,5233955	0,00054824
Phenylalanyl-trna synthetase, alpha subunit	*Farsa*	-1,5220338	0,00011139
Casitas B-lineage lymphoma	*Cbl*	-1,5219564	0,00087005
Solute carrier family 19 (folate transporter), member 1	*Slc19a1*	-1,5219038	0,00023566
Regulator of chromosome condensation 1	*Rcc1*	-1,521769	0,00018884
Signal peptidase complex subunit 3 homolog (S. Cerevisiae)	*Spcs3*	-1,5217093	0,0018725
Son of sevenless homolog 2 (Drosophila)	*Sos2*	-1,5216326	0,00513381
Isoprenylcysteine carboxyl methyltransferase	*Icmt*	-1,5209236	0,00015123
Zinc finger protein 52	*Zfp52*	-1,5203608	0,00209686
Methyltransferase like 6	*Mettl6*	-1,5202713	0,00194351
TAF6-like RNA polymerase II, p300/CBP-associated factor (PCAF)-associated factor	*Taf6l*	-1,5196983	0,00046279
Mitochondrial ribosomal protein L34	*Mrpl34*	-1,5188728	0,00032516
ER membrane protein complex subunit 6	*Emc6*	-1,518745	0,00016797
Heat shock protein 3	*Hspb3*	-1,5186855	0,00025267
Cutc copper transporter homolog (E.coli)	*Cutc*	-1,5181796	0,00346746
Twisted gastrulation homolog 1 (Drosophila)	*Twsg1*	-1,5181275	0,00013646
Conserved helix-loop-helix ubiquitous kinase	*Chuk*	-1,5178294	0,00398308
Transmembrane protein 167	*Tmem167*	-1,5176239	0,0002807
Sterile alpha motif domain containing 8	*Samd8*	-1,51758	0,00089917
Forty-two-three domain containing 1	*Fyttd1*	-1,5173989	0,0007858
Family with sequence similarity 45, member A	*Fam45a*	-1,5168882	0,00029865
Superoxide dismutase 1, soluble	*Sod1*	-1,5168822	6,17E-05
Ftsj homolog 3 (E. Coli)	*Ftsj3*	-1,5168068	0,00012544
Mpv17 transgene, kidney disease mutant-like	*Mpv17l*	-1,5159717	0,00092442
YTH domain containing 1	*Ythdc1*	-1,5158204	0,00016621
PET100 homolog (S. Cerevisiae)	*Pet100*	-1,5153093	0,0031974
Expressed sequence AI314180	*AI314180*	-1,5151743	0,00032385
Chromodomain helicase DNA binding protein 7	*Chd7*	-1,5151176	0,00218315
Leucine rich repeat containing 41	*Lrrc41*	-1,5151029	8,98E-05
Myosin regulatory light chain interacting protein	*Mylip*	-1,514539	0,00035636
Expressed sequence AI596198	*AI596198*	-1,5126882	0,00047845
F-box protein 22	*Fbxo22*	-1,510696	0,00041087
Microtubule-associated protein, RP/EB family, member 2	*Mapre2*	-1,5105506	0,00587783
Transducin-like enhancer of split 3, homolog of Drosophila E(spl)	*Tle3*	-1,509958	0,00151792
DNA-damage inducible protein 2 /// regulatory solute carrier protein, family 1, member 1	*Ddi2 /// Rsc1a1*	-1,5085249	9,94E-05
Jun D proto-oncogene	*Jund*	-1,5078434	0,00093072
V-rel reticuloendotheliosis viral oncogene homolog A (avian)	*Rela*	-1,5075491	0,00044442

Bone morphogenetic protein receptor, type 1B	*Bmpr1b*	-1,5073987	0,00390023
HERPUD family member 2	*Herpud2*	-1,5073818	0,00027973
Myod family inhibitor	*Mdfi*	-1,5069805	0,00449462
Hippocampus abundant transcript-like 1	*Hiatl1*	-1,5067789	0,00453329
Leucine zipper, putative tumor suppressor 2	*Lzts2*	-1,5064816	0,00021579
Microtubule-associated protein 1S	*Map1s*	-1,5063622	0,00011683
Predicted pseudogene 5848 /// small nuclear ribonucleoprotein D2	*Gm5848 /// Snrpd2*	-1,505755	9,93E-05
Atpase, Ca++ transporting, cardiac muscle, slow twitch 2	*Atp2a2*	-1,5053198	5,88E-05
Dipeptidylpeptidase 6	*Dpp6*	-1,5051077	0,0081124
AF4/FMR2 family, member 4	*Aff4*	-1,5039593	0,00224768
Predicted gene, 21596 /// predicted gene 6115 /// high mobility group box 1 /// high mobility group protein B1-like	*Gm21596 /// Gm6115 /// Hmgb1 /// LOC102635075*	-1,5031386	0,00483942
E26 avian leukemia oncogene 1, 5' domain	*Ets1*	-1,5030763	0,00035606
Akirin 2	*Akirin2*	-1,5021957	0,00015766
CLP1, cleavage and polyadenylation factor I subunit	*Clp1*	-1,5018862	0,00041727
Mitochondrial ribosomal protein L21	*Mrpl21*	-1,499967	0,00109431
SAC3 domain containing 1	*Sac3d1*	-1,4999473	0,00016344
Progestin and adipoq receptor family member VII	*Paqr7*	-1,4998693	0,00028222
Zinc finger protein 105	*Zfp105*	-1,4995911	0,00089917
Speckle-type POZ protein	*Spop*	-1,4995115	0,00049011
Coiled-coil domain containing 58	*Ccdc58*	-1,499396	0,00012759
Ubiquinol-cytochrome c reductase, Rieske iron-sulfur polypeptide 1	*Uqcrfs1*	-1,4993942	0,00016747
Z-DNA binding protein 1	*Zbp1*	-1,4990131	0,00056033
Poliovirus receptor-related 3	*Pvrl3*	-1,4987482	0,00108705
GTP binding protein 3	*Gtpbp3*	-1,4986328	0,00024769
Dynactin 6	*Dctn6*	-1,4985188	0,00060206
Ssu72 RNA polymerase II CTD phosphatase homolog (yeast)	*Ssu72*	-1,4980409	0,00059919
ADP-ribosylation factor-like 14 effector protein	*Arl14ep*	-1,4980237	0,00239958
Human immunodeficiency virus type I enhancer binding protein 1	*Hivep1*	-1,4973087	0,00116956
Rho gtpase activating protein 21	*Arhgap21*	-1,4962956	0,00024548
Eph receptor A2	*Epha2*	-1,4962259	0,00036145
Solute carrier family 2 (facilitated glucose transporter), member 10	*Slc2a10*	-1,4957242	0,00813973
Pre B cell leukemia homeobox 1	*Pbx1*	-1,4956351	0,00332683
Leucine rich repeat containing 8 family, member C	*Lrrc8c*	-1,4955783	0,0011614
Zinc finger, MYM-type 5	*Zmym5*	-1,4952664	0,00029405
Signal transducing adaptor molecule (SH3 domain and ITAM motif) 1	*Stam*	-1,4945348	0,00195184
Elongation of very long chain fatty acids (FEN1/Elo2, SUR4/Elo3, yeast)-like 1	*Elovl1*	-1,4944936	0,00047301
Shroom family member 1	*Shroom1*	-1,4943869	0,00101697
Atpase, Na+/K+ transporting, beta 3 polypeptide	*Atp1b3*	-1,4943073	0,00106932
Protein tyrosine phosphatase, non-receptor type 6	*Ptpn6*	-1,4930416	0,00063388

ATP-binding cassette, sub-family B (MDR/TAP), member 1A	*Abcb1a*	-1,4925482	0,00793449
Polymerase (RNA) III (DNA directed) polypeptide E	*Polr3e*	-1,4925066	0,0004915
Predicted gene 4604 /// ribosomal protein L36	*Gm4604 /// Rpl36*	-1,4924654	0,00087345
Kruppel-like factor 10	*Klf10*	-1,4923209	0,0001212
Zinc finger protein 948	*Zfp948*	-1,4922226	0,0006434
U6 snrna biogenesis 1	*Usb1*	-1,4921668	0,00022006
Zinc finger protein 26	*Zfp26*	-1,491224	0,00075525
Cytochrome c oxidase subunit viia polypeptide 2-like	*Cox7a2l*	-1,491079	0,00012338
Basic leucine zipper and W2 domains 2	*Bzw2*	-1,4910212	7,51E-05
Anaphase promoting complex subunit 1	*Anapc1*	-1,4909772	0,0001229
Ribosomal protein S25	*Rps25*	-1,4905657	0,00041412
CD209g antigen	*Cd209g*	-1,4897439	0,00381138
Ribosomal protein L7-like 1	*Rpl7l1*	-1,4891121	7,95E-05
Translocase of inner mitochondrial membrane 10B	*Timm10b*	-1,4890563	0,00044519
Hermansky-Pudlak syndrome 4 homolog (human)	*Hps4*	-1,4883092	0,00073207
Meteorin, glial cell differentiation regulator-like	*Metrnl*	-1,4881997	0,00039907
Single-stranded DNA binding protein 2	*Ssbp2*	-1,4878188	0,00024946
Zinc finger protein 65 /// zinc finger protein 738	*Zfp65 /// Zfp738*	-1,4875205	0,00305244
FK506 binding protein 9	*Fkbp9*	-1,4868795	0,00016866
Mitochondrial ribosomal protein L16	*Mrpl16*	-1,4868622	0,00010157
Transmembrane protein 62	*Tmem62*	-1,4867622	0,00178235
Glia maturation factor, beta	*Gmfb*	-1,4858794	0,00594102
Eukaryotic translation initiation factor 3, subunit C	*Eif3c*	-1,4856557	0,00016647
X-linked inhibitor of apoptosis	*Xiap*	-1,4839054	0,00034952
Polymerase (RNA) I polypeptide B	*Polr1b*	-1,4838789	0,00043619
Cytotoxic granule-associated RNA binding protein 1	*Tia1*	-1,483184	0,00024053
Integrin beta 1 (fibronectin receptor beta)	*Itgb1*	-1,4830543	0,00092938
Cartilage associated protein	*Crtap*	-1,4817813	0,00016582
Predicted gene 6910 /// RING1 and YY1 binding protein	*Gm6910 /// Rybp*	-1,4816607	0,00067045
Mindbomb homolog 2 (Drosophila)	*Mib2*	-1,4813609	0,00092442
Phosphoribosyl pyrophosphate amidotransferase	*Ppat*	-1,4809665	0,00122391
Myosin, light polypeptide 1	*Myl1*	-1,4806379	0,00390013
Scaffold attachment factor B2	*Safb2*	-1,4806333	0,00123371
Major facilitator superfamily domain containing 5	*Mfsd5*	-1,4804435	0,00010195
Small nuclear ribonucleoprotein 40 (U5)	*Snrnp40*	-1,4802282	0,00820339
Ras-related GTP binding C	*Rragc*	-1,4800545	0,00012021
BMP2 inducible kinase	*Bmp2k*	-1,4798024	0,00104695
Ring finger and WD repeat domain 3	*Rfwd3*	-1,4795076	0,00011171
Luc7 homolog (S. Cerevisiae)-like	*Luc7l*	-1,4793491	0,00046263
ERO1-like (S. Cerevisiae)	*Ero1l*	-1,4793302	0,00123988
Ubiquitin specific peptidase 18	*Usp18*	-1,4791648	0,00010009
F-box and leucine-rich repeat protein 12	*Fbxl12*	-1,4791078	0,00229091
Solute carrier family 7 (cationic amino acid transporter, y+ system), member 5	*Slc7a5*	-1,4787905	0,00746782

Heterogeneous nuclear ribonucleoprotein D-like	*Hnrnpdl*	-1,4780413	0,0008847
AT rich interactive domain 5A (MRF1-like)	*Arid5a*	-1,477457	0,0004965
Glucose-fructose oxidoreductase domain containing 2	*Gfod2*	-1,4771513	0,00010075
Phosphodiesterase 1A, calmodulin-dependent	*Pde1a*	-1,4770928	0,00024171
DPH2 homolog (S. Cerevisiae)	*Dph2*	-1,476765	0,00079192
Mitochondrial ribosomal protein L46	*Mrpl46*	-1,4760085	0,00036166
Lectin, galactoside-binding, soluble, 3 binding protein	*Lgals3bp*	-1,4757653	0,00866235
Inositol monophosphatase domain containing 1	*Impad1*	-1,4754148	0,0003177
CGG triplet repeat binding protein 1	*Cggbp1*	-1,4753767	0,00012009
Solute carrier family 38, member 2	*Slc38a2*	-1,4748157	0,00013223
Low density lipoprotein receptor-related protein associated protein 1	*Lrpap1*	-1,4747809	0,00020473
CCR4-NOT transcription complex, subunit 6-like	*Cnot6l*	-1,4745292	0,0010713
Casitas B-lineage lymphoma-like 1	*Cbll1*	-1,4744621	0,0062996
Alcohol dehydrogenase 5 (class III), chi polypeptide	*Adh5*	-1,4742087	0,00032245
Multivesicular body subunit 12A	*Mvb12a*	-1,4739939	0,00303494
Protein disulfide isomerase associated 4	*Pdia4*	-1,4737863	0,00078354
Mago-nashi homolog, proliferation-associated (Drosophila)	*Magoh*	-1,472782	0,00010682
Protein tyrosine phosphatase, non-receptor type 14	*Ptpn14*	-1,4722898	0,0015415
DNA segment, Chr 8, ERATO Doi 738, expressed	*D8Ertd738e*	-1,4722684	0,00048972
Golgi coiled coil 1	*Gcc1*	-1,4717202	0,00358
Translocase of inner mitochondrial membrane 8A1	*Timm8a1*	-1,47113	0,00036145
Fibulin 2	*Fbln2*	-1,4707538	0,00015574
Retinol dehydrogenase 13 (all-trans and 9-cis)	*Rdh13*	-1,4693994	0,00043317
Vacuolar protein sorting 37C (yeast)	*Vps37c*	-1,4691235	0,00072116
RIKEN cdna 1200003I10 gene /// RIKEN cdna 1200015M12 gene /// RIKEN cdna A130040M12 gene	*1200003I10Rik /// 1200015M12Rik /// A130040M12Rik*	-1,4690754	9,42E-05
T cell, immune regulator 1, atpase, H+ transporting, lysosomal V0 protein A3	*Tcirg1*	-1,4683915	0,00094292
Keratinocyte associated protein 2	*Krtcap2*	-1,4683182	0,00053358
Ribosomal protein S5	*Rps5*	-1,4680526	8,12E-05
Oxidase assembly 1-like	*Oxa1l*	-1,467952	0,00041824
Mannosyl-oligosaccharide glucosidase	*Mogs*	-1,4665038	0,00041027
Zinc finger with KRAB and SCAN domains 6	*Zkscan6*	-1,4663205	0,00108537
PRP31 pre-mrna processing factor 31 homolog (yeast)	*Prpf31*	-1,4658403	0,0012266
Family with sequence similarity 111, member A	*Fam111a*	-1,4651558	0,00012987
EH domain binding protein 1-like 1	*Ehbp1l1*	-1,4648802	0,00157944
U1 small nuclear ribonucleoprotein C	*Snrpc*	-1,4648181	0,00051668
WD repeat domain, phosphoinositide interacting 2	*Wipi2*	-1,4647933	0,00029662
Mannoside acetylglucosaminyltransferase 1	*Mgat1*	-1,4646796	0,00057832
Branched chain aminotransferase 2, mitochondrial	*Bcat2*	-1,4645563	0,00162541
Fyn proto-oncogene	*Fyn*	-1,4635119	0,00218315
Nuclear receptor subfamily 4, group A, member 1	*Nr4a1*	-1,4634666	0,00723007
Glycoprotein m6b	*Gpm6b*	-1,4630029	0,00122836

ATP-binding cassette, sub-family F (GCN20), member 1	*Abcf1*	-1,4628087	0,00310114
Myomesin 1	*Myom1*	-1,4627323	0,00122391
Oxidoreductase like domain containing 1	*Oxld1*	-1,4626343	0,00424994
Intersectin 1 (SH3 domain protein 1A)	*Itsn1*	-1,4620968	0,00418135
Solute carrier family 25 (mitochondrial carrier, adenine nucleotide translocator), member 4	*Slc25a4*	-1,4618501	0,00014956
Rearranged L-myc fusion sequence	*Rlf*	-1,4617369	0,00051741
Eva-1 homolog A (C. Elegans)	*Eva1a*	-1,4615198	0,00274572
MIS18 kinetochore protein homolog A (S. Pombe)	*Mis18a*	-1,4606966	0,00214147
Multiple endocrine neoplasia 1	*Men1*	-1,4601297	0,00135889
Hephaestin	*Heph*	-1,4597022	0,00651391
Predicted pseudogene 10913 /// predicted gene 12447 /// predicted gene 12508 /// predicted pseudogene 8210 /// 60S ribosomal protein L29-like /// 60S ribosomal protein L29-like /// ribosomal protein L29	*Gm10913 /// Gm12447 /// Gm12508 /// Gm8210 /// LOC100503055 /// LOC102642233 /// Rpl29*	-1,4590454	0,00018891
RIKEN cdna 1700030K09 gene	*1700030K09Rik*	-1,4583058	0,00015566
Trans-acting transcription factor 1	*Sp1*	-1,4578601	0,00209576
ELK4, member of ETS oncogene family	*Elk4*	-1,4577277	0,00037434
Cell division cycle associated 5	*Cdca5*	-1,4569556	0,00065083
Tripartite motif-containing 44	*Trim44*	-1,4568267	0,00015908
Interleukin 6 signal transducer	*Il6st*	-1,4564983	0,00025836
UBX domain protein 1	*Ubxn1*	-1,4562245	0,00026511
ADP-dependent glucokinase	*Adpgk*	-1,4556923	0,00091433
UFM1-specific peptidase 2	*Ufsp2*	-1,4555025	0,00017917
Tumor suppressing subtransferable candidate 1	*Tssc1*	-1,4554173	0,00020433
Vacuolar protein sorting 35	*Vps35*	-1,4551128	0,00580272
ATP-binding cassette, sub-family B (MDR/TAP), member 9	*Abcb9*	-1,4550357	0,00108225
Zinc finger protein 451	*Zfp451*	-1,4549187	0,00141971
Forkhead box C1	*Foxc1*	-1,4548893	0,0010052
Integrin beta 7	*Itgb7*	-1,4548003	0,00050617
Enhancer of yellow 2 homolog (Drosophila)	*Eny2*	-1,4547685	0,00058983
Protein phosphatase 6, regulatory subunit 3	*Ppp6r3*	-1,454654	0,00156755
CUGBP, Elav-like family member 1	*Celf1*	-1,4537795	0,00011036
Glutamate oxaloacetate transaminase 2, mitochondrial	*Got2*	-1,4529374	0,00028055
Neuroblastoma ras oncogene	*Nras*	-1,4526412	0,00083244
Zinc finger protein 213	*Zfp213*	-1,4516538	0,00047504
NOP10 ribonucleoprotein	*Nop10*	-1,4516468	0,00060192
CCAAT/enhancer binding protein zeta	*Cebpz*	-1,4512627	0,0005907
Pseudouridylate synthase 7 homolog (S. Cerevisiae)	*Pus7*	-1,4510244	0,00057396
Phosphodiesterase 12	*Pde12*	-1,4501755	0,00362628
B cell translocation gene 2, anti-proliferative	*Btg2*	-1,4492717	0,00423181
Zinc finger protein 160	*Zfp160*	-1,4488918	0,00040222

Carbohydrate kinase domain containing	*Carkd*	-1,4486921	0,00061355
Interactor of little elongation complex ELL subunit 2	*Ice2*	-1,4482409	0,00238026
Ethylmalonic encephalopathy 1	*Ethe1*	-1,4479951	0,00054654
Origin recognition complex, subunit 6	*Orc6*	-1,4479601	0,00022002
Kelch-like 42	*Klhl42*	-1,4478309	0,0017153
Adaptor protein complex AP-1, gamma 1 subunit	*Ap1g1*	-1,447534	0,00157987
UDP-Gal:betaglcnac beta 1,3-galactosyltransferase, polypeptide 4	*B3galt4*	-1,4470906	0,00369078
PR domain containing 5	*Prdm5*	-1,4465167	0,00111717
Kelch domain containing 4	*Klhdc4*	-1,4465047	0,00079785
Zinc finger protein 260	*Zfp260*	-1,4463507	0,00019854
Degenerative spermatocyte homolog 1 (Drosophila)	*Degs1*	-1,4463218	0,00040147
Predicted pseudogene 10913 /// predicted gene 12508 /// predicted pseudogene 8210 /// 60S ribosomal protein L29-like /// 60S ribosomal protein L29-like /// ribosomal protein L29	*Gm10913 /// Gm12508 /// Gm8210 /// LOC100503055 /// LOC102642233 /// Rpl29*	-1,4462965	0,00023024
Receptor (TNFRSF)-interacting serine-threonine kinase 1	*Ripk1*	-1,4462658	0,00845245
Signal transducer and activator of transcription 3	*Stat3*	-1,4455503	0,0003188
Solute carrier family 6 (neurotransmitter transporter, taurine), member 6	*Slc6a6*	-1,4454558	0,00339103
Forkhead box O3	*Foxo3*	-1,4445675	0,00035375
RNA binding motif protein 15B	*Rbm15b*	-1,4445567	0,00195726
Ribosomal protein L13A	*Rpl13a*	-1,4442226	0,00021095
Pellino 1	*Peli1*	-1,4441638	0,00736814
V-crk sarcoma virus CT10 oncogene homolog (avian)	*Crk*	-1,4439834	0,00076759
Jagunal homolog 1 (Drosophila)	*Jagn1*	-1,4439309	0,00065174
Pro-platelet basic protein	*Ppbp*	-1,4435505	0,00153407
Transmembrane emp24-like trafficking protein 10 (yeast)	*Tmed10*	-1,4434627	0,00013239
Caspase 2	*Casp2*	-1,4433248	0,0006098
WD repeat domain 46	*Wdr46*	-1,442211	0,0050227
Baculoviral IAP repeat-containing 2	*Birc2*	-1,4415792	0,00049982
Meis homeobox 2	*Meis2*	-1,441066	0,00100935
Heat shock protein 2	*Hspb2*	-1,4409612	0,00377633
WNT1 inducible signaling pathway protein 1	*Wisp1*	-1,4409044	0,00021922
ADP-ribosylation factor 6	*Arf6*	-1,4404298	0,00260014
RIO kinase 1 (yeast)	*Riok1*	-1,4401898	0,00171381
Max binding protein	*Mnt*	-1,4401309	0,00192324
Predicted gene 5617	*Gm5617*	-1,4399752	0,00136977
Rho gtpase activating protein 35	*Arhgap35*	-1,4396985	0,00284092
Ring finger protein 166	*Rnf166*	-1,4395557	0,00035235
BCL2-like 2	*Bcl2l2*	-1,4391899	0,00248474
Ribosomal protein S6 kinase, polypeptide 2	*Rps6ka2*	-1,439138	0,00069205
Protein O-glucosyltransferase 1	*Poglut1*	-1,4377974	0,00037774

Tubulin cofactor A	*Tbca*	-1,4374214	0,00031673
Glutamate receptor, ionotropic, kainate 5 (gamma 2)	*Grik5*	-1,437412	0,00726158
RIKEN cdna 1700123O20 gene	*1700123O20Rik*	-1,4368696	0,00120099
UDP-N-acteylglucosamine pyrophosphorylase 1-like 1	*Uap1l1*	-1,4366978	0,00032555
Methionine adenosyltransferase II, alpha	*Mat2a*	-1,4364619	0,00381906
Lysosomal-associated membrane protein 1	*Lamp1*	-1,4362542	0,00012987
Zinc finger protein 1	*Zfp1*	-1,4360126	0,00461472
RNA binding motif protein 6	*Rbm6*	-1,435105	0,00449671
Serine/arginine-rich splicing factor 7	*Srsf7*	-1,4348434	0,00187036
UDP-Gal:betaglcnac beta 1,4-galactosyltransferase, polypeptide 6	*B4galt6*	-1,4344453	0,00574636
Cyclin B1 /// predicted gene 5593	*Ccnb1 /// Gm5593*	-1,4335593	0,00254443
Adaptor protein, phosphotyrosine interaction, PH domain and leucine zipper containing 1	*Appl1*	-1,4335062	0,00768923
OTU domain containing 6B	*Otud6b*	-1,4334249	0,00276753
Serine/threonine kinase 38	*Stk38*	-1,4333589	0,0003551
Lysine (K)-specific demethylase 1A	*Kdm1a*	-1,4333207	0,00024053
SET domain containing 4	*Setd4*	-1,4332346	0,0007407
Myeloblastosis oncogene-like 2	*Mybl2*	-1,4332284	0,00018645
A disintegrin and metallopeptidase domain 17	*Adam17*	-1,4326966	0,00097909
Acetyl-Coenzyme A acyltransferase 1A /// acetyl-Coenzyme A acyltransferase 1B	*Acaa1a /// Acaa1b*	-1,4325931	0,00333898
Phospholipase D2	*Pld2*	-1,43203	0,00137624
Nuclear casein kinase and cyclin-dependent kinase substrate 1	*Nucks1*	-1,4318605	0,00080352
Small nuclear RNA activating complex, polypeptide 2	*Snapc2*	-1,4318281	0,00249839
LEM domain containing 2	*Lemd2*	-1,4316356	0,0015415
RAB5A, member RAS oncogene family	*Rab5a*	-1,431619	0,00609317
Syncoilin	*Sync*	-1,4310379	0,00132066
Gem (nuclear organelle) associated protein 6 /// predicted pseudogene 6253	*Gemin6 /// Gm6253*	-1,4309571	0,00044631
Torsin family 2, member A	*Tor2a*	-1,4305876	0,00065211
Transmembrane protein 183A	*Tmem183a*	-1,4302316	0,00094257
Neuroplastin	*Nptn*	-1,4302115	0,00524193
Mitogen-activated protein kinase kinase 7	*Map2k7*	-1,4300164	0,00994279
BUD31 homolog (yeast)	*Bud31*	-1,4298757	0,00402002
Obg-like atpase 1	*Ola1*	-1,4292277	0,00073934
Cell cycle associated protein 1	*Caprin1*	-1,4287046	0,00680542
PRP19/PSO4 pre-mrna processing factor 19 homolog (S. Cerevisiae)	*Prpf19*	-1,4285331	0,00021905
Beta-1,3-glucuronyltransferase 3 (glucuronosyltransferase I)	*B3gat3*	-1,4282185	0,00028604
Branched chain ketoacid dehydrogenase E1, alpha polypeptide	*Bckdha*	-1,4271772	0,00340039
RIKEN cdna C330006A16 gene	*C330006A16Rik*	-1,4270175	0,00211695
Solute carrier family 35, member F5	*Slc35f5*	-1,426569	0,00054372
Succinate dehydrogenase complex assembly factor 2	*Sdhaf2*	-1,426497	0,00101837
Pericentriolar material 1	*Pcm1*	-1,4262373	0,00754337

RIKEN cdna E130309D02 gene	*E130309D02Rik*	-1,4260741	0,00055672
Pentatricopeptide repeat domain 3	*Ptcd3*	-1,425855	0,00036121
Camp-regulated phosphoprotein 19	*Arpp19*	-1,4256068	0,00087698
Dolichyl-di-phosphooligosaccharide-protein glycotransferase	*Ddost*	-1,4255638	0,00016267
Phospholipid scramblase 3	*Plscr3*	-1,4252835	0,00050638
Mitochondrial ubiquitin ligase activator of NFKB 1	*Mul1*	-1,4250715	0,00324234
Malignant fibrous histiocytoma amplified sequence 1	*Mfhas1*	-1,4248158	0,00065173
Splicing factor 1	*Sf1*	-1,4246165	0,00054405
V-Ki-ras2 Kirsten rat sarcoma viral oncogene homolog	*Kras*	-1,4244298	0,0009151
Cyclin D binding myb-like transcription factor 1	*Dmtf1*	-1,4240882	0,0003188
Arfgap with gtpase domain, ankyrin repeat and PH domain 1	*Agap1*	-1,4240124	0,00325024
Pumilio RNA-binding family member 1	*Pum1*	-1,4238256	0,00107713
Zinc finger, BED domain containing 3	*Zbed3*	-1,4237683	0,00336956
UHRF1 (ICBP90) binding protein 1-like	*Uhrf1bp1l*	-1,4237511	0,00123143
Ubiquitin carboxyl-terminal esterase L5	*Uchl5*	-1,4236815	0,00755827
Ribonuclease P 14 subunit	*Rpp14*	-1,4236177	0,00112888
Atpase inhibitory factor 1	*Atpif1*	-1,423328	0,00072311
Mitochondrial ribosomal protein L1	*Mrpl1*	-1,4233222	0,00684219
Phospholipase A2, activating protein	*Plaa*	-1,4233031	0,00142695
Synaptophysin-like protein	*Sypl*	-1,4231562	0,00057475
Syndecan binding protein	*Sdcbp*	-1,4228437	0,00275137
Kelch-like 2, Mayven	*Klhl2*	-1,4228124	0,00123371
Neogenin	*Neo1*	-1,4220981	0,00100427
Predicted gene 13889	*Gm13889*	-1,4217186	0,00079122
Poly (ADP-ribose) polymerase family, member 8	*Parp8*	-1,4213311	0,00122386
DEAD (Asp-Glu-Ala-Asp) box polypeptide 46	*Ddx46*	-1,4203937	0,00616303
PHD finger protein 5A	*Phf5a*	-1,4200122	0,00087363
DET1 and DDB1 associated 1	*Dda1*	-1,4199495	0,00186058
Male-specific lethal 3-like 2 (Drosophila)	*Msl3l2*	-1,4197621	0,00133519
Apelin	*Apln*	-1,419522	0,00560969
Vascular endothelial growth factor A	*Vegfa*	-1,4194377	0,00721515
RNA binding motif protein 15	*Rbm15*	-1,4193708	0,00904805
Recombination signal binding protein for immunoglobulin kappa J region	*Rbpj*	-1,4192554	0,00242578
40S ribosomal protein S16-like /// ribosomal protein S16 /// ribosomal protein S16, pseudogene 2	*LOC100862433 /// Rps16 /// Rps16-ps2*	-1,4192283	0,00013801
Heat shock protein 12B	*Hspa12b*	-1,419097	0,00089331
TNF receptor-associated factor 3	*Traf3*	-1,4186725	0,00166522
Mitochondrial ribosomal protein L36	*Mrpl36*	-1,4182388	0,00039639
Tripartite motif-containing 28	*Trim28*	-1,4180726	0,00211005
Tripartite motif-containing 59	*Trim59*	-1,4175483	0,00942401
KAT8 regulatory NSL complex subunit 1-like	*Kansl1l*	-1,4172302	0,00435077
Calcium channel, voltage-dependent, P/Q type, alpha 1A subunit	*Cacna1a*	-1,4172149	0,00386754
Zinc finger and BTB domain containing 2	*Zbtb2*	-1,4171484	0,00054419

RAB8A, member RAS oncogene family	*Rab8a*	-1,4169514	0,00061966
Family with sequence similarity 173, member A	*Fam173a*	-1,4168384	0,00046871
N-myc (and STAT) interactor	*Nmi*	-1,4160876	0,00080201
Growth arrest specific 5 /// small nucleolar RNA, C/D box 47	*Gas5 /// Snord47*	-1,4141607	0,00456467
Par-6 family cell polarity regulator alpha	*Pard6a*	-1,4131836	0,000649
5-hydroxytryptamine (serotonin) receptor 6	*Htr6*	-1,4131538	0,00611941
Uridine monophosphate synthetase	*Umps*	-1,4127096	0,00054464
Nuclear factor of kappa light polypeptide gene enhancer in B cells 1, p105	*Nfkb1*	-1,4125569	0,00127271
Ras responsive element binding protein 1	*Rreb1*	-1,4119987	0,00043162
LIM domain binding 1	*Ldb1*	-1,411806	0,00083081
Ubiquitin specific peptidase 3	*Usp3*	-1,4117937	0,00127271
General transcription factor IIIC, polypeptide 4	*Gtf3c4*	-1,4116556	0,00077018
Protein-tyrosine sulfotransferase 2	*Tpst2*	-1,4115731	0,00017721
RIKEN cdna 2010107E04 gene	*2010107E04Rik*	-1,4113499	0,00052169
Solute carrier family 25, member 47	*Slc25a47*	-1,411116	0,00643059
Peroxisomal membrane protein 4	*Pxmp4*	-1,4107988	0,00684219
Htra serine peptidase 2	*Htra2*	-1,4105548	0,0061732
DDHD domain containing 1	*Ddhd1*	-1,410524	0,00305065
Ephrin A4	*Efna4*	-1,4104733	0,00510244
Dipeptidylpeptidase 7	*Dpp7*	-1,4102752	0,00298711
SURP and G patch domain containing 1	*Sugp1*	-1,4100868	0,00038947
Asparagine synthetase	*Asns*	-1,4088828	0,00103915
Splicing regulatory glutamine/lysine-rich protein 1interacting protein 1	*Srek1ip1*	-1,4087553	0,00262943
B cell translocation gene 1, anti-proliferative	*Btg1*	-1,4079916	0,00298065
RIKEN cdna 9530068E07 gene	*9530068E07Rik*	-1,4075172	0,00411504
Heterogeneous nuclear ribonucleoprotein H3	*Hnrnph3*	-1,4070118	0,0007097
Dolichol kinase	*Dolk*	-1,4063815	0,00023024
YTH domain family 3	*Ythdf3*	-1,406191	0,00459718
GRAM domain containing 4	*Gramd4*	-1,4060669	0,00060775
MLX interacting protein	*Mlxip*	-1,4057131	0,00483942
RIKEN cdna 4833420G17 gene	*4833420G17Rik*	-1,4054944	0,00374577
Serine/arginine-rich splicing factor 6	*Srsf6*	-1,405379	0,00072702
DNA methyltransferase 3B	*Dnmt3b*	-1,4045716	0,00161215
Microsomal glutathione S-transferase 1	*Mgst1*	-1,4041339	0,0003934
Translocase of inner mitochondrial membrane 8B	*Timm8b*	-1,4040517	0,00124566
RAB34, member RAS oncogene family	*Rab34*	-1,4039587	0,00114963
Zinc finger protein 955A	*Zfp955a*	-1,4037734	0,00292083
Neural proliferation, differentiation and control 1	*Npdc1*	-1,4035445	0,00233752
N(alpha)-acetyltransferase 15, nata auxiliary subunit	*Naa15*	-1,4025608	0,00110086
Protein arginine N-methyltransferase 1	*Prmt1*	-1,4024721	0,00049681
Proline synthetase co-transcribed	*Prosc*	-1,4023317	0,00459631
Predicted gene, 21596 /// predicted gene 6115 /// high mobility group box 1	*Gm21596 /// Gm6115 /// Hmgb1*	-1,4022854	0,00079192

Proteasome (prosome, macropain) assembly chaperone 1	*Psmg1*	-1,4022792	0,00038634
Polymerase (RNA) I polypeptide C	*Polr1c*	-1,4020927	0,00066855
Ligand dependent nuclear receptor corepressor	*Lcor*	-1,4017703	0,00574953
EMG1 nucleolar protein homolog (S. Cerevisiae)	*Emg1*	-1,4015159	0,00082989
Reticulocalbin 3, EF-hand calcium binding domain	*Rcn3*	-1,4008248	0,00068291
Cyclin C	*Ccnc*	-1,4000852	0,00788659
Transmembrane protein 126A	*Tmem126a*	-1,399661	0,0015582
Pleckstrin homology domain containing, family H (with myth4 domain) member 1	*Plekhh1*	-1,3990474	0,0082723
Zinc finger protein 280D	*Zfp280d*	-1,3988992	0,0014949
Nodal modulator 1	*Nomo1*	-1,3986057	0,00096682
40S ribosomal protein S6-like /// ribosomal protein S6 /// ribosomal protein S6, pseudogene 4	*LOC102643254 /// Rps6 /// Rps6-ps4*	-1,3979588	0,00637796
Nuclear RNA export factor 1	*Nxf1*	-1,3965658	0,00144943
Cytokine induced apoptosis inhibitor 1	*Ciapin1*	-1,3953741	0,00066206
Ribosomal protein S19	*Rps19*	-1,3949942	0,00045183
Exosome component 5	*Exosc5*	-1,3945773	0,00182512
Leucine carboxyl methyltransferase 2	*Lcmt2*	-1,3942534	0,00148731
Cyclin-dependent kinase 9 (CDC2-related kinase)	*Cdk9*	-1,3942022	0,00053873
Ubiquilin 4	*Ubqln4*	-1,3927469	0,00270733
Nudix (nucleoside diphosphate linked moiety X)-type motif 19	*Nudt19*	-1,3926801	0,00047688
Obscurin-like 1	*Obsl1*	-1,3926573	0,00095698
Aurora kinase A interacting protein 1	*Aurkaip1*	-1,3925584	0,00079985
EPS8-like 2	*Eps8l2*	-1,3920184	0,00280647
Transmembrane protein 40	*Tmem40*	-1,3913881	0,0091889
SEC62 homolog (S. Cerevisiae)	*Sec62*	-1,3911061	0,00049431
Von Willebrand factor A domain containing 9	*Vwa9*	-1,3908459	0,00031428
DEAH (Asp-Glu-Ala-His) box polypeptide 30	*Dhx30*	-1,3904979	0,00248177
Non-catalytic region of tyrosine kinase adaptor protein 2	*Nck2*	-1,3902193	0,00127918
Predicted gene 7285 /// ribosomal protein S7	*Gm7285 /// Rps7*	-1,3893568	0,00210565
Smg-8 homolog, nonsense mediated mrna decay factor (C. Elegans)	*Smg8*	-1,3892643	0,00399224
Protein kinase C, beta	*Prkcb*	-1,3891937	0,00099484
Prefoldin 2	*Pfdn2*	-1,3890149	0,00226054
Protein phosphatase 1, regulatory (inhibitor) subunit 15A	*Ppp1r15a*	-1,3887416	0,00274762
Prolactin family 7, subfamily a, member 2	*Prl7a2*	-1,3886413	0,00218315
Kelch-like 9	*Klhl9*	-1,3884216	0,0032382
FAST kinase domains 2	*Fastkd2*	-1,3882827	0,00123419
Ribonuclease H1	*Rnaseh1*	-1,3882595	0,00187045
PHD finger protein 23	*Phf23*	-1,3879364	0,00086316
Zinc finger, ZZ domain containing 3	*Zzz3*	-1,3878622	0,00624921
Thyroid hormone receptor interactor 11	*Trip11*	-1,3877726	0,00076173
Protein phosphatase 2, regulatory subunit B', gamma	*Ppp2r5c*	-1,3876736	0,00795429

Mitochondrial fission process 1	*Mtfp1*	-1,3873039	0,00073207
PAK1 interacting protein 1	*Pak1ip1*	-1,3872946	0,00058301
Ribosomal protein S7	*Rps7*	-1,3870419	0,00051056
Glutamyl-trna synthetase 2 (mitochondrial)(putative)	*Ears2*	-1,3870052	0,00831015
Heme binding protein 2	*Hebp2*	-1,3865564	0,00918587
Methylmalonic aciduria (cobalamin deficiency) type A	*Mmaa*	-1,3857624	0,00952001
Transforming growth factor beta regulated gene 1	*Tbrg1*	-1,3852016	0,0005721
POZ (BTB) and AT hook containing zinc finger 1	*Patz1*	-1,3848074	0,00150371
Cyclin H	*Ccnh*	-1,38473	0,00930323
Acidic (leucine-rich) nuclear phosphoprotein 32 family, member A	*Anp32a*	-1,3845019	0,00193849
Myo-inositol 1-phosphate synthase A1	*Isyna1*	-1,3843555	0,00057615
NADH dehydrogenase (ubiquinone) 1 alpha subcomplex, assembly factor 1	*Ndufaf1*	-1,3838753	0,00077353
Predicted pseudogene 10913 /// predicted gene 11449 /// predicted gene 12508 /// predicted gene 13213 /// predicted pseudogene 8210 /// 60S ribosomal protein L29-like /// 60S ribosomal protein L29-like /// ribosomal protein L29	*Gm10913 /// Gm11449 /// Gm12508 /// Gm13213 /// Gm8210 /// LOC100503055 /// LOC102642233 /// Rpl29*	-1,3836333	0,00025042
V-maf musculoaponeurotic fibrosarcoma oncogene family, protein G (avian)	*Mafg*	-1,3830084	0,00390737
Missing oocyte, meiosis regulator, homolog (Drosophila)	*Mios*	-1,3828066	0,00523618
Mitochondrial ribosomal protein L15	*Mrpl15*	-1,3814584	0,00157771
Translocase of inner mitochondrial membrane 10	*Timm10*	-1,3812301	0,00488818
Zinc finger, MYND domain containing 19	*Zmynd19*	-1,3806345	0,00030994
Lipin 2	*Lpin2*	-1,3805234	0,00725129
Protein tyrosine phosphatase, non-receptor type 12	*Ptpn12*	-1,3798454	0,00101363
Solute carrier family 27 (fatty acid transporter), member 1	*Slc27a1*	-1,3794797	0,00123419
Glutamate-cysteine ligase, catalytic subunit	*Gclc*	-1,3793581	0,00362656
Collagen, type VI, alpha 1	*Col6a1*	-1,3792668	0,00828297
Rap1 gtpase-activating protein	*Rap1gap*	-1,3780535	0,00239958
Family with sequence similarity 65, member A	*Fam65a*	-1,3777678	0,00506012
Nuclear receptor co-repressor 1	*Ncor1*	-1,3777313	0,00173451
Fibrillarin	*Fbl*	-1,3775561	0,0008092
V-erb-b2 erythroblastic leukemia viral oncogene homolog 2, neuro/glioblastoma derived oncogene homolog (avian)	*Erbb2*	-1,377198	0,00914449
RRS1 ribosome biogenesis regulator homolog (S. Cerevisiae)	*Rrs1*	-1,3771758	0,00193445
Calmodulin-like 4	*Calml4*	-1,3771142	0,00727112
La ribonucleoprotein domain family, member 6	*Larp6*	-1,3771075	0,00844261
Phospholipid scramblase 2	*Plscr2*	-1,3770161	0,00181766
Family with sequence similarity 3, member C	*Fam3c*	-1,3767622	0,00364103
RWD domain containing 4A	*Rwdd4a*	-1,3763772	0,00212573

CDC16 cell division cycle 16	*Cdc16*	-1,3762925	0,0023501
G patch domain containing 4	*Gpatch4*	-1,3759992	0,00275137
Ataxia telangiectasia and Rad3 related	*Atr*	-1,3746862	0,00109098
Activin receptor IIA	*Acvr2a*	-1,3745861	0,00125411
5-hydroxymethylcytosine (hmc) binding, ES cell specific	*Hmces*	-1,3744602	0,00046875
Sorting nexin 9	*Snx9*	-1,3738016	0,00721217
Spinster homolog 1	*Spns1*	-1,3737078	0,00588763
COBW domain containing 1	*Cbwd1*	-1,3732248	0,00102868
WD repeat containing, antisense to Trp73	*Wrap73*	-1,3730975	0,00199976
RIKEN cdna 5430417L22 gene	*5430417L22Rik*	-1,3730423	0,00172213
Nucleoporin 133	*Nup133*	-1,3717135	0,00086316
Eukaryotic translation initiation factor 3, subunit I	*Eif3i*	-1,3710274	0,00099252
Mitogen-activated protein kinase kinase kinase kinase 4	*Map4k4*	-1,3709504	0,00611496
CWF19-like 1, cell cycle control (S. Pombe)	*Cwf19l1*	-1,370276	0,00636841
Kinesin family member 1B	*Kif1b*	-1,3695334	0,00635373
DEAD (Asp-Glu-Ala-Asp) box polypeptide 39	*Ddx39*	-1,369067	0,0091889
Reduced expression 2	*Rex2*	-1,3686344	0,00279972
Xanthine dehydrogenase	*Xdh*	-1,3684082	0,00214969
Eukaryotic translation initiation factor 3, subunit K	*Eif3k*	-1,3680018	0,00093145
Leucine rich repeat containing 1	*Lrrc1*	-1,3677854	0,00610652
Adipocyte plasma membrane associated protein	*Apmap*	-1,3676628	0,00362909
Snail family zinc finger 2	*Snai2*	-1,367357	0,00810768
Ecotropic viral integration site 5	*Evi5*	-1,3672181	0,00140985
Nuclear factor, erythroid derived 2, like 3	*Nfe2l3*	-1,3671036	0,00431783
Asteroid homolog 1 (Drosophila)	*Aste1*	-1,3668807	0,00558472
Complexin 2	*Cplx2*	-1,3668777	0,0015868
Ring finger protein 4	*Rnf4*	-1,3668108	0,00065738
MOB kinase activator 1B	*Mob1b*	-1,3667296	0,00055996
NHP2 ribonucleoprotein	*Nhp2*	-1,366569	0,00106581
Coiled-coil domain containing 23	*Ccdc23*	-1,3664759	0,00036145
Era (G-protein)-like 1 (E. Coli)	*Eral1*	-1,3655403	0,00827619
RIKEN cdna B230308N11 gene	*B230308N11Rik*	-1,3653189	0,00669654
RIKEN cdna 1110001J03 gene	*1110001J03Rik*	-1,3652436	0,00365674
Calumenin	*Calu*	-1,3647969	0,00121339
Solute carrier family 44, member 1	*Slc44a1*	-1,3646631	0,00686204
Ankyrin repeat domain 17	*Ankrd17*	-1,3636579	0,00190185
Peptidylprolyl isomerase B	*Ppib*	-1,3635457	0,00102032
Carbonic anhydrase 11	*Car11*	-1,3634487	0,0078093
Transforming growth factor, beta receptor II	*Tgfbr2*	-1,3630331	0,00197898
RNA-binding region (RNP1, RRM) containing 3	*Rnpc3*	-1,3628587	0,00793302
Moloney leukemia virus 10	*Mov10*	-1,3620052	0,0016849
WD repeat domain 4	*Wdr4*	-1,3616678	0,00416532
NECAP endocytosis associated 2	*Necap2*	-1,3613531	0,00182523

Death effector domain-containing	*Dedd*	-1,3609541	0,0054553
Histidine triad nucleotide binding protein 1	*Hint1*	-1,3606377	0,00047299
DEAD (Asp-Glu-Ala-Asp) box polypeptide 24	*Ddx24*	-1,360481	0,00063272
Glucocorticoid modulatory element binding protein 1	*Gmeb1*	-1,3602835	0,00257891
Thioredoxin domain containing 5	*Txndc5*	-1,3601116	0,00092484
LSM1 homolog, U6 small nuclear RNA associated (S. Cerevisiae)	*Lsm1*	-1,3597313	0,00206179
Transcription factor Dp 1	*Tfdp1*	-1,3591504	0,00947253
Mitochondrial ribosomal protein S9	*Mrps9*	-1,358806	0,00913704
Zinc finger protein 574	*Zfp574*	-1,3586953	0,00171956
Axin 1	*Axin1*	-1,3586159	0,00713702
NME/NM23 nucleoside diphosphate kinase 2	*Nme2*	-1,3581724	0,00060813
Annexin A4	*Anxa4*	-1,3578764	0,00396456
Procollagen-lysine, 2-oxoglutarate 5-dioxygenase 3	*Plod3*	-1,3575965	0,00156034
Transmembrane protein 258	*Tmem258*	-1,3574434	0,00382598
SUMO/sentrin specific peptidase 3	*Senp3*	-1,3570349	0,00154477
Minichromosome maintenance deficient 5, cell division cycle 46 (S. Cerevisiae)	*Mcm5*	-1,3569216	0,00161879
Protein tyrosine phosphatase, receptor type, K	*Ptprk*	-1,3565744	0,00073612
Yae1 domain containing 1	*Yae1d1*	-1,3565157	0,00084869
UPF2 regulator of nonsense transcripts homolog (yeast)	*Upf2*	-1,3565147	0,00092266
V-crk sarcoma virus CT10 oncogene homolog (avian)-like	*Crkl*	-1,3565021	0,0008008
Excision repaiross-complementing rodent repair deficiency, complementation group 8	*Ercc8*	-1,3563958	0,00061521
RNA methyltransferase like 1	*Rnmtl1*	-1,3560774	0,00052767
Paired related homeobox 1	*Prrx1*	-1,3559575	0,00046485
Thiamine triphosphatase	*Thtpa*	-1,3559145	0,00087553
MTERF domain containing 2	*Mterfd2*	-1,3557415	0,00349366
Myogenic differentiation 1	*Myod1*	-1,3557231	0,00332087
6-phosphogluconolactonase	*Pgls*	-1,3552691	0,00195022
Transmembrane protein 175	*Tmem175*	-1,3552261	0,00749904
RIKEN cdna 3930401B19 gene	*3930401B19Rik*	-1,3552157	0,00056579
Protein phosphatase 2 (formerly 2A), catalytic subunit, beta isoform	*Ppp2cb*	-1,3551805	0,00999505
CDC42 small effector 2	*Cdc42se2*	-1,3550928	0,00044536
DPH1 homolog (S. Cerevisiae) /// candidate tumor suppressor in ovarian cancer 2	*Dph1 /// Ovca2*	-1,3548741	0,00045761
Predicted gene 3258 /// suppressor of Ty 4A	*Gm3258 /// Supt4a*	-1,3546795	0,00072702
Glycine cleavage system protein H (aminomethyl carrier)	*Gcsh*	-1,3543018	0,00074896
Predicted gene 10071 /// ribosomal protein L13	*Gm10071 /// Rpl13*	-1,3542836	0,00064278
Craniofacial development protein 1	*Cfdp1*	-1,3540518	0,0014334
Methylthioribose-1-phosphate isomerase homolog (S. Cerevisiae)	*Mri1*	-1,3538078	0,00206327
Suppressor of Ty 20	*Supt20*	-1,3536243	0,00165223
OMA1 homolog, zinc metallopeptidase (S. Cerevisiae)	*Oma1*	-1,3531216	0,00337965

V-ral simian leukemia viral oncogene homolog B (ras related)	*Ralb*	-1,3530351	0,00109053
Interferon induced transmembrane protein 3	*Ifitm3*	-1,3528775	0,00151829
Poly (ADP-ribose) glycohydrolase	*Parg*	-1,3527618	0,00162433
Ras homolog gene family, member C	*Rhoc*	-1,3526325	0,00760977
Polymerase (RNA) II (DNA directed) polypeptide C	*Polr2c*	-1,352579	0,00067248
Metaxin 1	*Mtx1*	-1,3524581	0,00278967
Protein phosphatase 1, regulatory (inhibitor) subunit 15b	*Ppp1r15b*	-1,3518057	0,00078875
Iron responsive element binding protein 2	*Ireb2*	-1,35179	0,00520147
Transmembrane protein 243, mitochondrial	*Tmem243*	-1,3516857	0,00216361
Sodium channel, voltage-gated, type V, alpha	*Scn5a*	-1,3516061	0,00711798
DEAH (Asp-Glu-Ala-His) box polypeptide 9	*Dhx9*	-1,3512457	0,00686359
Regulating synaptic membrane exocytosis 2	*Rims2*	-1,3509803	0,00240092
Transcription elongation factor B (SIII), polypeptide 1	*Tceb1*	-1,3508528	0,00229047
SH3 domain containing ring finger 1	*Sh3rf1*	-1,3507767	0,00287706
F-box protein 33	*Fbxo33*	-1,3507426	0,0024164
PRP39 pre-mrna processing factor 39 homolog (yeast)	*Prpf39*	-1,35046	0,00364777
Eukaryotic translation initiation factor 5	*Eif5*	-1,3502969	0,00113766
Mitochondrial ribosomal protein S14	*Mrps14*	-1,3500457	0,00820457
Mitochondrial ribosomal protein L49	*Mrpl49*	-1,3495385	0,00244177
UDP-N-acetyl-alpha-D-galactosamine:polypeptide N-acetylgalactosaminyltransferase 11	*Galnt11*	-1,3493032	0,00206811
Family with sequence similarity 92, member A	*Fam92a*	-1,3488876	0,00052303
Serine (or cysteine) peptidase inhibitor, clade D, member 1	*Serpind1*	-1,3488252	0,00075179
Grpe-like 1, mitochondrial	*Grpel1*	-1,3484835	0,00096556
YLP motif containing 1	*Ylpm1*	-1,3481207	0,00187036
Kelch-like 21	*Klhl21*	-1,3478974	0,00193497
Activin receptor IIB	*Acvr2b*	-1,347673	0,00912922
NADH dehydrogenase (ubiquinone) 1 beta subcomplex 8	*Ndufb8*	-1,3472837	0,00437692
Glucosamine-phosphate N-acetyltransferase 1	*Gnpnat1*	-1,3466537	0,0016849
Troponin C, cardiac/slow skeletal	*Tnnc1*	-1,3456244	0,00209274
Dynein cytoplasmic 1 heavy chain 1	*Dync1h1*	-1,3453945	0,00209682
Thyroid hormone receptor associated protein 3	*Thrap3*	-1,3449113	0,00246176
Zinc finger protein of the cerebellum 4	*Zic4*	-1,3447572	0,00740171
Coenzyme Q9 homolog (yeast)	*Coq9*	-1,3445714	0,0022653
RIKEN cdna 0610031J06 gene	*0610031J06Rik*	-1,3445005	0,0007016
Crystallin, lambda 1	*Cryl1*	-1,3443448	0,00290463
THUMP domain containing 1	*Thumpd1*	-1,3439008	0,00366182
MMS19 (MET18 S. Cerevisiae)	*Mms19*	-1,343794	0,00235673
Mitochondrial ribosomal protein L53	*Mrpl57*	-1,3425892	0,00968752
Kxdl motif containing 1 /// ubiquitin A-52 residue ribosomal protein fusion product 1	*Kxd1 /// Uba52*	-1,3424518	0,00082683
Striatin, calmodulin binding protein 4	*Strn4*	-1,342079	0,0037554

NDC80 homolog, kinetochore complex component (S. Cerevisiae)	*Ndc80*	-1,3420624	0,00637796
Golgi autoantigen, golgin subfamily a, 5	*Golga5*	-1,3419078	0,0018826
Ribosomal protein L27	*Rpl27*	-1,3407693	0,00273577
Non imprinted in Prader-Willi/Angelman syndrome 2 homolog (human)	*Nipa2*	-1,3405447	0,00133308
Necdin-like 2	*Ndnl2*	-1,3402513	0,00381242
Transmembrane protein 165	*Tmem165*	-1,3396645	0,00487355
Malonyl-coa decarboxylase	*Mlycd*	-1,3394669	0,00184814
Translocase of outer mitochondrial membrane 40 homolog (yeast)	*Tomm40*	-1,3393517	0,00455609
Adenine phosphoribosyl transferase	*Aprt*	-1,3385585	0,00528173
CREB binding protein	*Crebbp*	-1,3384618	0,00192301
Zinc finger protein 746	*Zfp746*	-1,3382678	0,00130716
Eukaryotic translation initiation factor 1 /// predicted pseudogene 5471	*Eif1 /// Gm5471*	-1,3377613	0,00077188
Anti-silencing function 1B histone chaperone	*Asf1b*	-1,3373529	0,00134538
Ngg1 interacting factor 3-like 1 (S. Pombe)	*Nif3l1*	-1,3371044	0,00112291
Calcium modulating ligand	*Caml*	-1,3367828	0,00175775
THO complex 6 homolog (Drosophila)	*Thoc6*	-1,3366872	0,00248934
Predicted gene, 17748 /// peptidyl prolyl isomerase H	*Gm17748 /// Ppih*	-1,3366614	0,00332087
RAB12, member RAS oncogene family	*Rab12*	-1,3366018	0,00228856
Mitochondrial ribosomal protein L2	*Mrpl2*	-1,3364543	0,00231981
RAB9, member RAS oncogene family	*Rab9*	-1,336123	0,00327836
Vaccinia related kinase 1	*Vrk1*	-1,3360625	0,00166902
Testis development related protein	*Tdrp*	-1,3358878	0,00112633
Ribosomal protein S8 pseudogene /// 40S ribosomal protein S8-like /// ribosomal protein S8	*Gm15501 /// LOC101055915 /// Rps8*	-1,3352712	0,00159035
DDB1 and CUL4 associated factor 15	*Dcaf15*	-1,3352399	0,00342377
SURP and G patch domain containing 2	*Sugp2*	-1,3345941	0,00919651
Rab9 effector protein with kelch motifs	*Rabepk*	-1,3337619	0,00091988
Eukaryotic translation initiation factor 3, subunit J1 /// eukaryotic translation initiation factor 3, subunit J2	*Eif3j1 /// Eif3j2*	-1,3322151	0,00080381
Ring finger protein 44	*Rnf44*	-1,3320347	0,00331433
Cyclin M2	*Cnnm2*	-1,3318202	0,00319549
Huntingtin interacting protein 1 related	*Hip1r*	-1,3313762	0,00432788
Radixin	*Rdx*	-1,3307348	0,00403913
Trafficking protein particle complex 6A	*Trappc6a*	-1,3305005	0,00172527
Leucine rich repeat containing 2	*Lrrc2*	-1,3300732	0,00674245
Torsin family 4, member A	*Tor4a*	-1,329622	0,00359623
Aarf domain containing kinase 4	*Adck4*	-1,329298	0,00465753
Protein phosphatase 4, regulatory subunit 2	*Ppp4r2*	-1,3282616	0,00218315
Polymerase (DNA directed), beta	*Polb*	-1,3281328	0,00793149
Aconitase 1	*Aco1*	-1,3280347	0,00580272
Fas-associated factor 1	*Faf1*	-1,3276973	0,0014144
Proteasome (prosome, macropain) assembly chaperone 4	*Psmg4*	-1,3276711	0,00600423

Atpase, Na+/K+ transporting, alpha 1 polypeptide	*Atp1a1*	-1,326678	0,00086967
Meningioma expressed antigen 5 (hyaluronidase)	*Mgea5*	-1,3266474	0,00317703
Cholinergic receptor, nicotinic, alpha polypeptide 1 (muscle)	*Chrna1*	-1,3265625	0,0030439
Interferon regulatory factor 9	*Irf9*	-1,3264722	0,00609927
Heterogeneous nuclear ribonucleoprotein F	*Hnrnpf*	-1,3264348	0,00416612
Polypyrimidine tract binding protein 2	*Ptbp2*	-1,3263515	0,00867657
Potassium channel modulatory factor 1	*Kcmf1*	-1,3256361	0,0022963
Signal recognition particle receptor, B subunit	*Srprb*	-1,3254156	0,0016836
Serine/threonine kinase 19	*Stk19*	-1,3254065	0,00164393
Small integral membrane protein 7	*Smim7*	-1,3253468	0,00273404
G-rich RNA sequence binding factor 1	*Grsf1*	-1,3252173	0,00965863
Splicing factor proline/glutamine rich (polypyrimidine tract binding protein associated)	*Sfpq*	-1,325117	0,00107541
Tectonic family member 3	*Tctn3*	-1,3249514	0,00543821
Mannose-6-phosphate receptor, cation dependent	*M6pr*	-1,3249413	0,00525868
Family with sequence similarity 220, member A	*Fam220a*	-1,3236407	0,00211912
Zinc finger CCCH-type containing 18	*Zc3h18*	-1,3236085	0,00453016
Rala binding protein 1	*Ralbp1*	-1,3228891	0,00599674
Suppression of tumorigenicity 5	*St5*	-1,3222113	0,00508101
Nuclear receptor coactivator 6	*Ncoa6*	-1,3216515	0,00440435
Calcium/calmodulin-dependent protein kinase kinase 1, alpha	*Camkk1*	-1,3213661	0,00563905
Tetratricopeptide repeat, ankyrin repeat and coiled-coil containing 1	*Tanc1*	-1,3212214	0,00114985
RMI1, recq mediated genome instability 1, homolog (S. Cerevisiae)	*Rmi1*	-1,3210134	0,00135785
Tissue factor pathway inhibitor	*Tfpi*	-1,3209459	0,00133688
Heat shock protein 8	*Hspa8*	-1,3208261	0,00476492
Cyclin Pas1/PHO80 domain containing 1	*Cnppd1*	-1,3207001	0,00168646
Lipoyltransferase 1	*Lipt1*	-1,3203321	0,0036098
Arginine glutamic acid dipeptide (RE) repeats	*Rere*	-1,3191086	0,00554985
Regulator of G-protein signaling 3	*Rgs3*	-1,3189592	0,00256548
Heterogeneous nuclear ribonucleoprotein A3 pseudogene /// heterogeneous nuclear ribonucleoprotein A3	*Gm6793 /// Hnrnpa3*	-1,3179423	0,00218548
Coiled-coil domain containing 166	*Ccdc166*	-1,3174177	0,00820851
Transcription factor B2, mitochondrial	*Tfb2m*	-1,3172364	0,00130222
Sulfatase modifying factor 1	*Sumf1*	-1,3170449	0,00362656
Glutamine-rich 1	*Qrich1*	-1,3169632	0,00132902
Phospholipase D family, member 3	*Pld3*	-1,3169436	0,00600022
Predicted pseudogene 8069 /// translocase of outer mitochondrial membrane 5 homolog (yeast)	*Gm8069 /// Tomm5*	-1,3165018	0,00194985
Spermatogenesis associated 5	*Spata5*	-1,3163539	0,00844499
Mitogen-activated protein kinase kinase 4	*Map2k4*	-1,3158266	0,00451918
Cell division cycle 20	*Cdc20*	-1,3149905	0,00690341
SMAD family member 5	*Smad5*	-1,3149388	0,00495596
Adenosine monophosphate deaminase 2	*Ampd2*	-1,3142382	0,00564545

Ly1 antibody reactive clone	*Lyar*	-1,3139791	0,00509759
Ribosomal protein L14 /// ribosomal protein L14, pseudogene 1	*Rpl14 /// Rpl14-ps1*	-1,3135725	0,00216542
Small nuclear RNA activating complex, polypeptide 3	*Snapc3*	-1,3135402	0,00162604
Jun dimerization protein 2	*Jdp2*	-1,3127204	0,00210952
Exostoses (multiple) 2	*Ext2*	-1,3126028	0,00206213
Ubiquitin fusion degradation 1 like	*Ufd1l*	-1,312436	0,00492026
Ninjurin 1	*Ninj1*	-1,311904	0,00633542
Zinc finger protein 598	*Zfp598*	-1,3118126	0,00519463
Activator of basal transcription 1	*Abt1*	-1,3117281	0,00391993
Solute carrier family 22 (organic cation transporter), member 5	*Slc22a5*	-1,3114843	0,00241931
Ring finger and WD repeat domain 2	*Rfwd2*	-1,3112673	0,00258173
Dedicator of cytokinesis 5	*Dock5*	-1,3107247	0,00375784
Oxysterol binding protein	*Osbp*	-1,3103041	0,00118631
Ras homolog enriched in brain	*Rheb*	-1,3097625	0,00160982
Cellular retinoic acid binding protein II	*Crabp2*	-1,309286	0,00129859
BRISC and BRCA1 A complex member 1	*Babam1*	-1,3092284	0,00303338
Transcription factor 25 (basic helix-loop-helix)	*Tcf25*	-1,3091429	0,00412964
Eukaryotic translation elongation factor 1 beta 2	*Eef1b2*	-1,3088871	0,00188711
SET domain containing 3	*Setd3*	-1,3086155	0,00127547
RAB30, member RAS oncogene family	*Rab30*	-1,3084321	0,00218548
Polyhomeotic-like 1 (Drosophila)	*Phc1*	-1,3082457	0,00207626
Flt3 interacting zinc finger protein 1	*Fiz1*	-1,3082161	0,00204198
Transcription elongation factor B (SIII), polypeptide 3	*Tceb3*	-1,3081592	0,00659165
Transmembrane emp24 protein transport domain containing 9	*Tmed9*	-1,3075883	0,00783824
SAP30 binding protein	*Sap30bp*	-1,30741	0,00703044
Casein kinase 1, gamma 1	*Csnk1g1*	-1,3071138	0,00987942
Monoamine oxidase A	*Maoa*	-1,3070755	0,0017522
Transmembrane 4 superfamily member 1	*Tm4sf1*	-1,306832	0,0018366
V-myc myelocytomatosis viral related oncogene, neuroblastoma derived (avian)	*Mycn*	-1,306336	0,0042478
Protein phosphatase 3, catalytic subunit, beta isoform	*Ppp3cb*	-1,306151	0,00277875
Intercellular adhesion molecule 4, Landsteiner-Wiener blood group	*Icam4*	-1,3058289	0,0093523
WD repeat domain containing 82	*Wdr82*	-1,3056994	0,00667494
Atpase, H+ transporting, lysosomal V0 subunit C	*Atp6v0c*	-1,3051779	0,00258752
WW domain containing transcription regulator 1	*Wwtr1*	-1,3051408	0,00409497
Coiled-coil domain containing 88C	*Ccdc88c*	-1,3049583	0,00947783
Inositol (myo)-1(or 4)-monophosphatase 1	*Impa1*	-1,3044127	0,00672941
Ubiquitin domain containing 1	*Ubtd1*	-1,3043982	0,00693328
Ubiquinol-cytochrome c reductase, complex III subunit VII	*Uqcrq*	-1,3037967	0,00415781
V-raf-leukemia viral oncogene 1	*Raf1*	-1,3037794	0,00265497
Zinc finger and BTB domain containing 18	*Zbtb18*	-1,3035698	0,0024318
BTB and CNC homology 1	*Bach1*	-1,3033604	0,00435243

Glycyl-trna synthetase	*Gars*	-1,3026342	0,00437392
Nudix (nucleoside diphosphate linked moiety X)-type motif 21	*Nudt21*	-1,302414	0,00129276
Dynamin binding protein	*Dnmbp*	-1,3021222	0,00557044
NSA2 ribosome biogenesis homolog (S. Cerevisiae)	*Nsa2*	-1,3021052	0,00299058
Sorting nexin 5	*Snx5*	-1,3018595	0,00248297
PHD finger protein 20	*Phf20*	-1,3007603	0,00122682
Transmembrane protein 160	*Tmem160*	-1,3007256	0,00621886
Tight junction protein 2	*Tjp2*	-1,3005734	0,00810042
Structural maintenance of chromosomes 4	*Smc4*	-1,2999074	0,00425191
Histone cluster 1, h2bc /// histone cluster 1, h2be /// histone cluster 1, h2bg	*Hist1h2bc /// Hist1h2be /// Hist1h2bg*	-1,2987979	0,00410655
U2 small nuclear ribonucleoprotein B	*Snrpb2*	-1,2986471	0,00266679
Forkhead box J3	*Foxj3*	-1,2981995	0,00429576
Mitochondrial ribosomal protein S2	*Mrps2*	-1,2980735	0,00272887
Tetraspanin 3	*Tspan3*	-1,2980451	0,00123063
Expressed sequence AI646383	*AI646383*	-1,2980415	0,00609317
Heat shock protein 1 (chaperonin)	*Hspd1*	-1,2979654	0,0098692
Protein phosphatase 1A, magnesium dependent, alpha isoform	*Ppm1a*	-1,2979003	0,00135785
Protein phosphatase 1, catalytic subunit, alpha isoform	*Ppp1ca*	-1,2973889	0,00105979
Zinc finger protein 358	*Zfp358*	-1,2973617	0,00861418
Retinitis pigmentosa 9 (human)	*Rp9*	-1,2972694	0,00368255
Solute carrier family 5, member 4a	*Slc5a4a*	-1,2971036	0,00967531
Checkpoint with forkhead and ring finger domains	*Chfr*	-1,2970913	0,00697761
Peroxisome proliferator activated receptor alpha	*Ppara*	-1,2970431	0,00681956
Sorting nexin 1	*Snx1*	-1,2966004	0,00221532
Calcium/calmodulin-dependent protein kinase ID	*Camk1d*	-1,2965924	0,00779299
Coiled-coil domain containing 71	*Ccdc71*	-1,296323	0,008325
MAP/microtubule affinity-regulating kinase 3	*Mark3*	-1,2961938	0,00625051
Trimethylguanosine synthase homolog (S. Cerevisiae)	*Tgs1*	-1,2958578	0,00849747
Zinc finger protein 937	*Zfp937*	-1,2957811	0,00391993
DEAD (Asp-Glu-Ala-Asp) box polypeptide 20	*Ddx20*	-1,2956852	0,00572459
H2A histone family, member J	*H2afj*	-1,2951546	0,00703956
A kinase (PRKA) anchor protein 1	*Akap1*	-1,2944974	0,00828491
BRCA1/BRCA2-containing complex, subunit 3	*Brcc3*	-1,2944604	0,00430637
Ankyrin repeat and sterile alpha motif domain containing 3	*Anks3*	-1,293431	0,00465766
Predicted gene 13363 /// protein tyrosine phosphatase 4a1	*Gm13363 /// Ptp4a1*	-1,2933346	0,00503404
Phosphatidylserine synthase 2	*Ptdss2*	-1,2931598	0,00359623
Nucleophosmin 1	*Npm1*	-1,2917716	0,00920234
Aftiphilin	*Aftph*	-1,2914376	0,00125176
Programmed cell death 2	*Pdcd2*	-1,2913408	0,00459816
Guanine nucleotide binding protein (G protein), beta polypeptide 2 like 1	*Gnb2l1*	-1,2911748	0,00152358

Dnaj (Hsp40) homolog, subfamily C, member 13	*Dnajc13*	-1,2902246	0,00371811
Ribosomal protein S6 /// ribosomal protein S6, pseudogene 4	*Rps6 /// Rps6-ps4*	-1,2891263	0,00238389
Transmembrane protein 184C	*Tmem184c*	-1,2888437	0,00440142
Six transmembrane epithelial antigen of the prostate 1	*Steap1*	-1,2875608	0,00156211
Tight junction associated protein 1	*Tjap1*	-1,2872952	0,00647022
DEAD (Asp-Glu-Ala-Asp) box polypeptide 49	*Ddx49*	-1,2871154	0,00530349
Protein kinase, membrane associated tyrosine/threonine 1	*Pkmyt1*	-1,286965	0,00336167
Ring finger protein 8	*Rnf8*	-1,2868529	0,00224186
Calmodulin regulated spectrin-associated protein 1	*Camsap1*	-1,2866231	0,00739845
Complement component 1, r subcomponent A	*C1ra*	-1,2864829	0,00445643
EH-domain containing 4	*Ehd4*	-1,286318	0,00250828
Increased sodium tolerance 1 homolog (yeast)	*Ist1*	-1,2862283	0,00371082
Nuclear receptor coactivator 1	*Ncoa1*	-1,2860723	0,00447787
Cold shock domain containing E1, RNA binding	*Csde1*	-1,2854945	0,00290384
DEAD (Asp-Glu-Ala-Asp) box polypeptide 39B	*Ddx39b*	-1,285232	0,00317583
AT rich interactive domain 1A (SWI-like)	*Arid1a*	-1,2849504	0,00896808
SH3-domain GRB2-like B1 (endophilin)	*Sh3glb1*	-1,2849005	0,0047134
Predicted gene 10420 /// predicted gene 6139 /// predicted gene 8841 /// predicted gene 8842 /// ribosomal protein S2	*Gm10420 /// Gm6139 /// Gm8841 /// Gm8842 /// Rps2*	-1,2845977	0,00194666
Histone cluster 1, h2bc /// histone cluster 1, h2be /// histone cluster 1, h2bf /// histone cluster 1, h2bg /// histone cluster 1, h2bj /// histone cluster 1, h2bl /// histone cluster 1, h2bm /// histone cluster 1, h2bn /// histone cluster 1, h2bp /// histone cluster 1, h2bq /// histone cluster 1 h2br	*Hist1h2bc /// Hist1h2be /// Hist1h2bf /// Hist1h2bg /// Hist1h2bj /// Hist1h2bl /// Hist1h2bm /// Hist1h2bn /// Hist1h2bp /// Hist1h2bq /// Hist1h2br*	-1,2845948	0,00342875
Heme oxygenase (decycling) 2	*Hmox2*	-1,2840568	0,00285018
Translocase of inner mitochondrial membrane 23	*Timm23*	-1,2838018	0,00178957
Mitochondrial ribosomal protein S6	*Mrps6*	-1,2837837	0,00515454
Glutaminyl-trna synthetase	*Qars*	-1,2837155	0,00348603
Nuclear autoantigenic sperm protein (histone-binding)	*Nasp*	-1,2836996	0,00257993
TAP binding protein	*Tapbp*	-1,2834837	0,00919634
Gene trap locus 3	*Gtl3*	-1,2831649	0,00377121
AT rich interactive domain 5B (MRF1-like)	*Arid5b*	-1,2825715	0,00656953
Trna isopentenyltransferase 1	*Trit1*	-1,2821478	0,0041625
CAS1 domain containing 1	*Casd1*	-1,2819132	0,00383507
Transportin 2 (importin 3, karyopherin beta 2b)	*Tnpo2*	-1,2814198	0,00844793
Serpine1 mrna binding protein 1	*Serbp1*	-1,2810461	0,00460083
Jumping translocation breakpoint	*Jtb*	-1,2809629	0,00271118
Hyaluronoglucosaminidase 2	*Hyal2*	-1,2799986	0,00420874
Fat mass and obesity associated	*Fto*	-1,279337	0,00577733

Heterogeneous nuclear ribonucleoprotein L-like	*Hnrnpll*	-1,2790606	0,00474874
N-terminal Xaa-Pro-Lys N-methyltransferase 1	*Ntmt1*	-1,2787182	0,00890829
Natural killer tumor recognition sequence	*Nktr*	-1,2781775	0,0098407
Angiomotin-like 2	*Amotl2*	-1,2777194	0,00404085
DEAD (Asp-Glu-Ala-Asp) box polypeptide 19a /// DEAD (Asp-Glu-Ala-Asp) box polypeptide 19b	*Ddx19a /// Ddx19b*	-1,2770209	0,00845727
Ceroid-lipofuscinosis, neuronal 8	*Cln8*	-1,2766295	0,00581212
3-hydroxy-3-methylglutaryl-Coenzyme A reductase	*Hmgcr*	-1,2756734	0,00519203
Mitochondrial ribosomal protein S34	*Mrps34*	-1,2750963	0,00550333
Polycystic kidney disease 2	*Pkd2*	-1,2749734	0,00201029
Copine III	*Cpne3*	-1,2748139	0,00286492
Trafficking protein particle complex 12	*Trappc12*	-1,2740359	0,00250933
Lysine (K)-specific demethylase 2B	*Kdm2b*	-1,2739493	0,00564363
La ribonucleoprotein domain family, member 1	*Larp1*	-1,2738553	0,00211113
Upregulated during skeletal muscle growth 5	*Usmg5*	-1,2724119	0,00880746
Zinc finger protein 354B	*Zfp354b*	-1,2721526	0,00316229
Sema domain, immunoglobulin domain (Ig), short basic domain, secreted, (semaphorin) 3B	*Sema3b*	-1,2712656	0,00464004
Cytidine 5'-triphosphate synthase 2	*Ctps2*	-1,2702922	0,00427473
Tripartite motif-containing 27	*Trim27*	-1,2700776	0,00856457
Platelet-activating factor acetylhydrolase, isoform 1b, subunit 2	*Pafah1b2*	-1,2699855	0,00970412
Taurine upregulated gene 1	*Tug1*	-1,2680684	0,00334486
Dolichyl-phosphate (UDP-N-acetylglucosamine) acetylglucosaminephosphotransferase 1 (glcnac-1-P transferase)	*Dpagt1*	-1,2675105	0,00404687
KH domain containing, RNA binding, signal transduction associated 1	*Khdrbs1*	-1,2673234	0,00658988
Digeorge syndrome critical region gene 2	*Dgcr2*	-1,2667848	0,00751963
Ribosomal protein SA	*Rpsa*	-1,266516	0,00386772
Transmembrane protein 134	*Tmem134*	-1,2664549	0,00572271
Eukaryotic translation initiation factor 2B, subunit 1 (alpha)	*Eif2b1*	-1,2657599	0,00382598
B cell leukemia/lymphoma 10	*Bcl10*	-1,2655486	0,00694831
Brain abundant, membrane attached signal protein 1	*Basp1*	-1,2653039	0,00258824
Polymerase (RNA) II (DNA directed) polypeptide G	*Polr2g*	-1,2652343	0,00899818
MOB kinase activator 1A	*Mob1a*	-1,2648486	0,00919471
Laminin B1	*Lamb1*	-1,2648192	0,00984768
Zinc finger, CCHC domain containing 6	*Zcchc6*	-1,2633362	0,00967061
Small nuclear ribonucleoprotein polypeptide G	*Snrpg*	-1,262844	0,00472295
WD repeat domain, phosphoinositide interacting 1	*Wipi1*	-1,2613378	0,00965065
Protein phosphatase 3, catalytic subunit, alpha isoform	*Ppp3ca*	-1,2609396	0,00920234
STIP1 homology and U-Box containing protein 1	*Stub1*	-1,2604673	0,00452485
Sequestosome 1	*Sqstm1*	-1,2601815	0,00240092
GID complex subunit 4, VID24 homolog (S. Cerevisiae)	*Gid4*	-1,2598659	0,00933976
NOL1/NOP2/Sun domain family member 2	*Nsun2*	-1,2593897	0,00305857
WAS protein family, member 2	*Wasf2*	-1,2591478	0,00580697

Glutamyl-prolyl-trna synthetase	*Eprs*	-1,258936	0,00505066
Endoplasmic reticulum aminopeptidase 1	*Erap1*	-1,2575368	0,006245
Complement component factor h	*Cfh*	-1,2573938	0,00729833
Ubiquitin-conjugating enzyme E2I	*Ube2i*	-1,2570384	0,00486792
Ribosomal protein S18	*Rps18*	-1,2570026	0,00301573
Heterogeneous nuclear ribonucleoprotein U	*Hnrnpu*	-1,2556769	0,00412602
Transmembrane protein 234	*Tmem234*	-1,2548406	0,00691904
Tumor protein D52-like 2	*Tpd52l2*	-1,2548302	0,00354108
Homeodomain interacting protein kinase 1	*Hipk1*	-1,2541372	0,0067176
CKLF-like MARVEL transmembrane domain containing 3	*Cmtm3*	-1,253692	0,00309332
CUE domain containing 2	*Cuedc2*	-1,253594	0,00975669
RIKEN cdna 1110059E24 gene	*1110059E24Rik*	-1,2524145	0,00360978
Amyloid beta (A4) precursor-like protein 2	*Aplp2*	-1,2499177	0,00712605
Heat shock factor binding protein 1	*Hsbp1*	-1,2499086	0,00368697
Leucine rich repeat containing 59	*Lrrc59*	-1,2490071	0,00563905
Cell division cycle 5-like (S. Pombe)	*Cdc5l*	-1,2481402	0,00552176
Myosin VA	*Myo5a*	-1,2480049	0,0056851
Zinc metallopeptidase, STE24	*Zmpste24*	-1,2477531	0,00886977
Rho guanine nucleotide exchange factor (GEF) 12	*Arhgef12*	-1,2476985	0,00933976
RIKEN cdna 4933426M11 gene	*4933426M11Rik*	-1,2463169	0,00583159
Mature T cell proliferation 1	*Mtcp1*	-1,2453009	0,00850857
F-box and WD-40 domain protein 9	*Fbxw9*	-1,2452619	0,0037885
Ribosomal protein S28	*Rps28*	-1,244866	0,00358
AHA1, activator of heat shock protein atpase 1	*Ahsa1*	-1,2444935	0,00818232
Tissue inhibitor of metalloproteinase 1	*Timp1*	-1,2439144	0,00691157
Armadillo repeat containing 10	*Armc10*	-1,2436546	0,00844261
Ceramide synthase 2	*Cers2*	-1,2435895	0,00417842
Ring finger protein 220	*Rnf220*	-1,2434506	0,00782947
NADH dehydrogenase (ubiquinone) 1 alpha subcomplex, 4	*Ndufa4*	-1,2434103	0,00971267
CCR4-NOT transcription complex, subunit 6	*Cnot6*	-1,2430205	0,00878405
Prohibitin 2	*Phb2*	-1,2429736	0,00905146
Solute carrier family 41, member 1	*Slc41a1*	-1,2427921	0,00896726
Vang-like 2 (van gogh, Drosophila)	*Vangl2*	-1,242777	0,0047893
Nucleolar complex associated 4 homolog (S. Cerevisiae)	*Noc4l*	-1,2416448	0,00449462
Ribosomal protein S2	*Rps2*	-1,2408877	0,00505213
Ribosomal protein L11	*Rpl11*	-1,2398668	0,00424622
Secreted acidic cysteine rich glycoprotein	*Sparc*	-1,2396918	0,00361493
Spermidine/spermine N1-acetyl transferase 1	*Sat1*	-1,2391896	0,00887822
Ligase III, DNA, ATP-dependent	*Lig3*	-1,2391337	0,00425111
Retinoblastoma binding protein 4	*Rbbp4*	-1,23866	0,00572459
Mitochondrial ribosomal protein L44	*Mrpl44*	-1,2385716	0,00972162
Karyopherin (importin) beta 1	*Kpnb1*	-1,2385552	0,00674605

DEAD/H (Asp-Glu-Ala-Asp/His) box polypeptide 3, X-linked	*Ddx3x*	-1,2380816	0,00784432
WD repeat domain 61	*Wdr61*	-1,2380119	0,00993476
Ribosomal L24 domain containing 1	*Rsl24d1*	-1,2378915	0,00695677
WD repeat domain 75	*Wdr75*	-1,2374292	0,00762642
BTB (POZ) domain containing 1	*Btbd1*	-1,2373559	0,00843453
Chromatin target of PRMT1	*Chtop*	-1,2362104	0,00605997
Sestrin 3	*Sesn3*	-1,235405	0,00847816
Actin related protein 2/3 complex, subunit 1B /// predicted pseudogene 5637	*Arpc1b /// Gm5637*	-1,2339399	0,00722147
STE20-like kinase	*Slk*	-1,2334486	0,00761074
Ankyrin repeat and zinc finger domain containing 1	*Ankzf1*	-1,2307969	0,00999717
Tuberous sclerosis 1	*Tsc1*	-1,2303183	0,00818718
Solute carrier family 25, member 51	*Slc25a51*	-1,230027	0,00662571
Bromodomain and PHD finger containing, 1	*Brpf1*	-1,2291561	0,00639022
Heat shock protein 9	*Hspa9*	-1,2291101	0,00712605
RAB24, member RAS oncogene family	*Rab24*	-1,228365	0,00998206
Acyl-Coenzyme A dehydrogenase, medium chain	*Acadm*	-1,227889	0,00969837
Chloride intracellular channel 4 (mitochondrial)	*Clic4*	-1,2262202	0,00537209
PDZ and LIM domain 1 (elfin)	*Pdlim1*	-1,2256053	0,00895794
Ribosomal protein S6 kinase, polypeptide 2	*Rps6kb2*	-1,2253649	0,00908973
Ribosomal protein, large, P0	*Rplp0*	-1,2203392	0,00939832
Vacuolar protein sorting 54 (yeast)	*Vps54*	-1,21603	0,00888068
GAR1 ribonucleoprotein homolog (yeast)	*Gar1*	-1,2154116	0,00807736
Heat shock protein 90 alpha (cytosolic), class B member 1	*Hsp90ab1*	-1,2130992	0,00781176
